# Photoinduced carbonylative annulation access to β-lactams†

**DOI:** 10.1039/d5sc02418h

**Published:** 2025-04-25

**Authors:** Yuanrui Wang, Xin Qi, Zhi-Peng Bao, Xiao-Feng Wu

**Affiliations:** a Dalian National Laboratory for Clean Energy, Dalian Institute of Chemical Physics, Chinese Academy of Sciences Dalian Liaoning 116023 China xwu2020@dicp.ac.cn; b University of Chinese Academy of Sciences Beijing 100049 China; c Leibniz-Institut für Katalyse e.V. Albert-Einstein-Straße 29a Rostock 18059 Germany Xiao-Feng.Wu@catalysis.de

## Abstract

In radical carbonylation chemistry, orderly and sequential construction of C–C and C–N bonds with CO can effectively approach amide units and quickly incorporate a wide range of functional groups. However, this procedure remains underdeveloped for the synthesis of β-lactams. In general, especially for four-membered rings, end-to-end annulation is a thermodynamically unfavorable process compared to [2 + 2] cycloaddition. Here we developed a photoinduced radical relay carbonylative annulation (RRCA) strategy in which the key β-amino acyl radical intermediates exhibit superior capability of cyclization. This unique and underrated property is crucial in the process of successfully overcoming the tension of four-membered annulation for the synthesis of β-lactams. Mild conditions and wide substrate compatibility indicate the value of this method in the field of new drug discovery with special therapeutic effects. Particularly, embedding the amine group of the amino acid into the β-lactam skeleton further illustrates the utility of this methodology enabling late-stage modification of bioactive molecules.

## Abstract 2

A photoinduced radical relay carbonylative annulation (RRCA) strategy for the synthesis of β-lactams has been developed.

## Graphical abstract

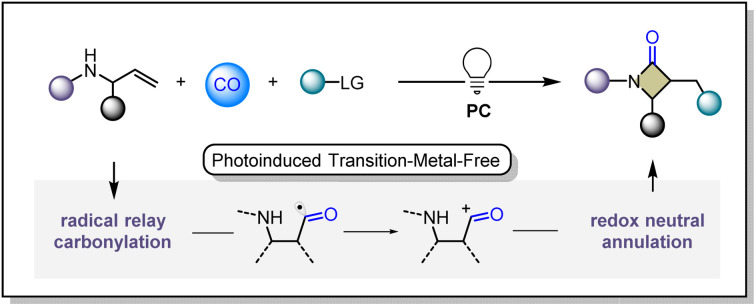

## Introduction

**Fig. 1:**
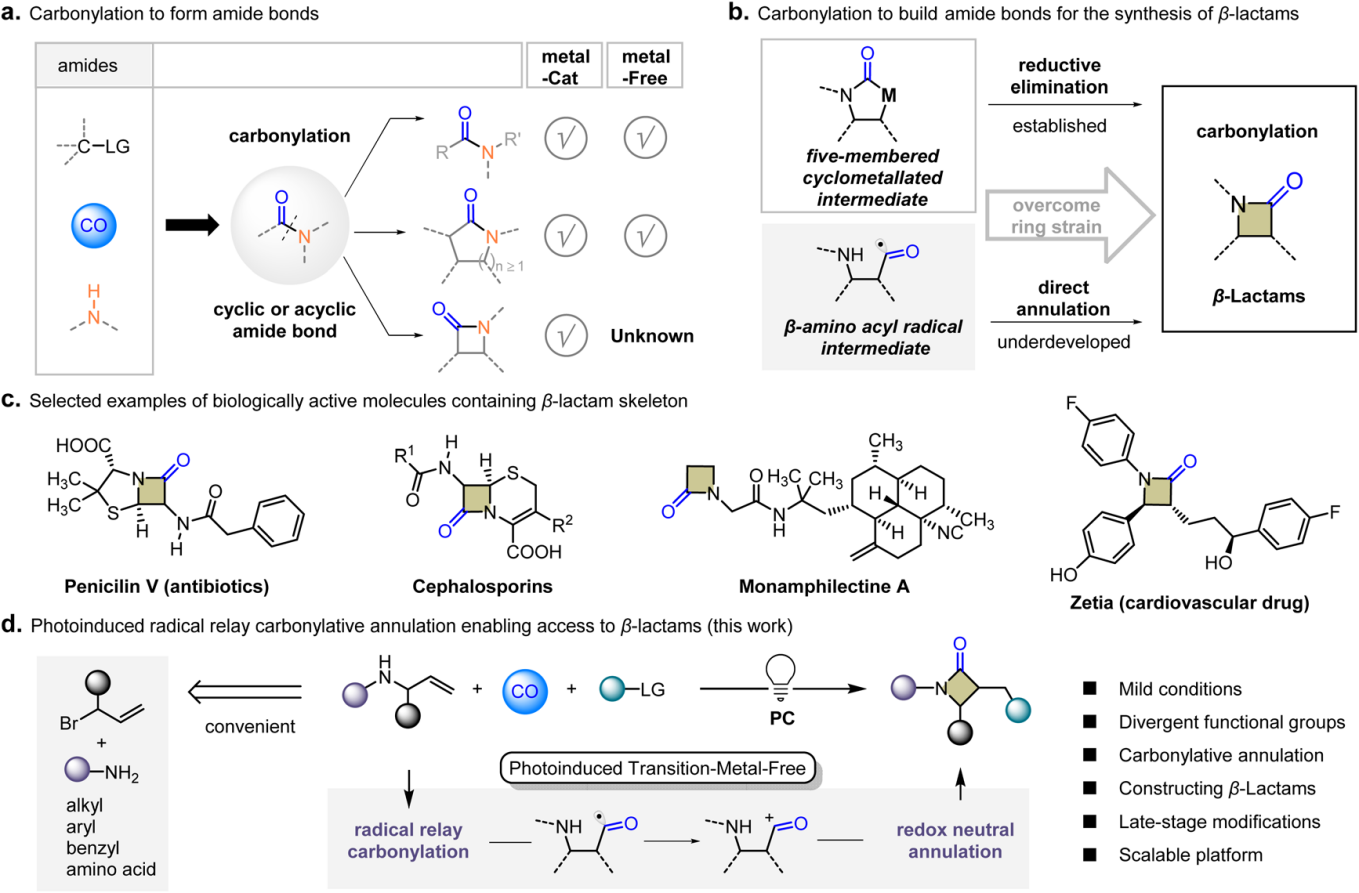
Background and carbonylative approaches for β-lactams. (a) Aminocarbonylation using CO to form amide bonds. (b) Our concept: β-amino acyl radical intermediates direct annulation. (c) Selected examples of biologically active molecules containing the β-lactam skeleton. (d) This work: visible-light-induced radical relay carbonylative annulation strategy to access β-lactams.

As an inexpensive and readily available bulk industrial raw material, CO is considered as an ideal carbonyl source for building amide bonds with amines.^1^ However, in contrast to rather well-established aminocarbonylation for building acyclic amide bonds, strategies to access structurally strained cyclic amides are scarce, especially for the four-membered β-lactams (Fig. 1a).^2^ Carbonylative four-membered annulation is a challenging retrosynthetic disconnection that is *de novo* introduction of the carbonyl group using CO.^3^ In 2016, Gaunt's group provided a general palladium-catalyzed β-C–H carbonylation of aliphatic amines to β-lactams.^4^ In this case, reductive elimination of the five-membered cyclometallated intermediate was suggested the key step (Fig. 1b). To the best of our knowledge, transition-metal-free carbonylative annulation to construct β-lactams is scarce and challenging because of the difficulty to balance CO insertion and annulation. Considering that photo-induced radical carbonylation has been successfully applied in practice to eliminate the dependence on transition metals.^5^ We hypothesize that the β-amino acyl radical can be obtained through radical tandem carbonylation with readily available allylamine. It is conceivable that β-lactams may be successfully obtained on the premise that the ring strain can be overcome. This transition-metal-free annulation would be a substantial advancement, providing a rapid access to β-lactams with divergent functional groups.

β-Lactam is one of the most coveted four-membered heterocycles containing amide bonds.^6^ As the core skeleton of antibiotics, such as penicillins and cephalosporins, the synthesis of β-lactam by chemical methods has been a long-lasting research hotspot.^7^ As a biologically active pharmacophore, it has received special attention in the pharmaceutical discovery (Fig. 1c).^8^ Characterization and structural analysis of β-lactams by infrared spectroscopy and X-ray crystallography show that the C–N bond between the nitrogen atom and carbonyl is quite different from other cyclic and acyclic amides.^9^ The angle strain greatly reduced the resonance between the lone pair of the N atom and the C=O double bond. Despite the strained four-membered ring, it is stable and contains a reactive amide bond. These unique properties also make it an interesting synthetic building block in diverse transformations.^10^ Some strategies have been developed to construct β-lactams from carbonyl-containing precursors, such as the classical Staudinger reaction that was reported as early as 1928, which is formal [2 + 2] cycloaddition of ketenes and imines.^11^ However, it is troubling that the construction of carbonyl-containing precursors is often tedious and accompanied by harsh conditions. Although the synthesis of β-lactams using CO as a carbonyl source has also attracted attention in carbonylation chemistry for a long time,^12^ most strategies are limited by the catalysis of transition metals and the diversity of substituents on the amine unit is mostly limited. It is urgent to develop a novel platform for the rapid construction of β-lactams that can introduce diverse functional groups while broadening the boundaries of the amine units in the skeleton.

Nowadays, photocatalysis has become a powerful tool in synthetic catalysis, introducing new reaction mechanisms and creating non-traditional catalytic modes.^13^ In fact, inspiring achievements have been made in the construction of small rings promoted by light excitation, especially in the scope of nitrogen-containing four-membered rings. Triplet energy transfer photocatalysis makes it possible to synthesize azetidines between imines and alkenes by [2 + 2]-cycloaddition (aza-Paternò–Büchi reaction).^14^ Radical strain–release of azabicyclo[1.1.0]butanes induced by organic photosensitizers has been reported to achieve the preparation of azetidines.^15^ However, visible-light mediated synthesis of β-lactams still has not received much attention. It is worth mentioning that Norrish-Yang type photocyclization of acrylamides is used to synthesize β-lactams,^16^ but limited substrate applicability hinders the development of diverse synthesis. Photoinduced intramolecular four-membered ring closure is also a very practical proposal, but it has always been restricted by the following problems: (a) the necessity of a leaving group increases the difficulty of substrate construction and the sensitivity of the functional group. (b) The high oxidation potential makes the formation of carbocation intermediates very difficult. (c) The existence of ring strain always makes the cyclization process uncertain and elusive.

Herein, we deliver a visible-light-induced radical relay carbonylative annulation (RRCA) strategy for the straightforward and efficient synthesis of β-lactams. Conversion of readily available amines and CO into value-added β-lactams is shown in Fig. 1d. The key to success is the β-amino acyl radical with low oxidation potential, which means it can be easily oxidized to the acyl cation. Due to the extremely strong electrophilic ability of the acyl cation, it quickly combines with nucleophiles. The efficiency of this process offers the possibility of breaking through the restriction of ring tension and connect end-to-end to form a four-membered ring. A variety of electrophilic radicals were investigated as candidates for functionalization, including the difluoroalkyl radical, trifluoromethyl radical, trichloromethyl radical, cyanomethylene radical and acylmethylene radical. The modular allylation process using allyl bromide is convenient for obtaining various types of allylic amines. General reaction conditions are compatible with a series of primary amines such as alkyl, aryl, and benzyl as the nitrogen unit source of β-lactams. Particularly, embedding amino acids derivatives abundant in natural products into β-lactam backbones is still an unexplored territory.

## Results and discussion

To verify the above assumptions about radical relay carbonylative β-lactamization, we first used allylated methyl 1-aminocyclohexane-1-carboxylate 1a as the template substrate and commercially available ethyl difluorobromoacetate 2a as the electrophilic radical precursor. Extensive screening of conditions showed that the target product 3a could be obtained in excellent yield (86%) by using 4CzIPN (2 mol%) as a photosensitizer, adding an ionic weak base Na_2_HPO_4_ (1.5 equiv.), using acetonitrile as a solvent, and fully irradiating the reaction system with blue light at room temperature under a CO atmosphere of 40 bar (Table 1, entry 1). The examination of photocatalysts showed that more than one organic photoredox catalyst can trigger and complete this procedure, but the results are not as satisfactory as 4CzIPN (Table 1, entries 2–5 and ESI Table S1†). The screening of bases, including common inorganic salts and organic bases, led us to find that weak alkalinity was crucial to obtain the product 3a with high yields (Table 1, entries 6–7 and ESI S2†). We suspect that a stronger base will formylate the amine 1a, blocking the cyclization process in the presence of CO. Other solvents, such as THF, provided only moderate yields of product 3a (46%), and if the reaction was carried out in methanol, only trace amounts of 3a were obtained (Table 1, entries 8–9 and ESI Table S3†). Understandably, protic polar solvents may act as competitive nucleophiles, with a significant inhibitory effect on the target transformation. Lowering the pressure mainly results in slower reaction rates and lower raw material conversion, resulting in unsatisfactory yields (Table 1, entry 10 and ESI Table S4†), which is consistent with the need for high pressure in sp2 carbon radicals capturing CO. The light-avoidance control experiment shows that light is essential (Table 1, entry 11). Without the addition of the photocatalyst 4CzIPN, the conversion from 1a to 3a cannot be achieved even with continuous light irradiation.

**Table 1:** Optimization of the reaction conditions^a^

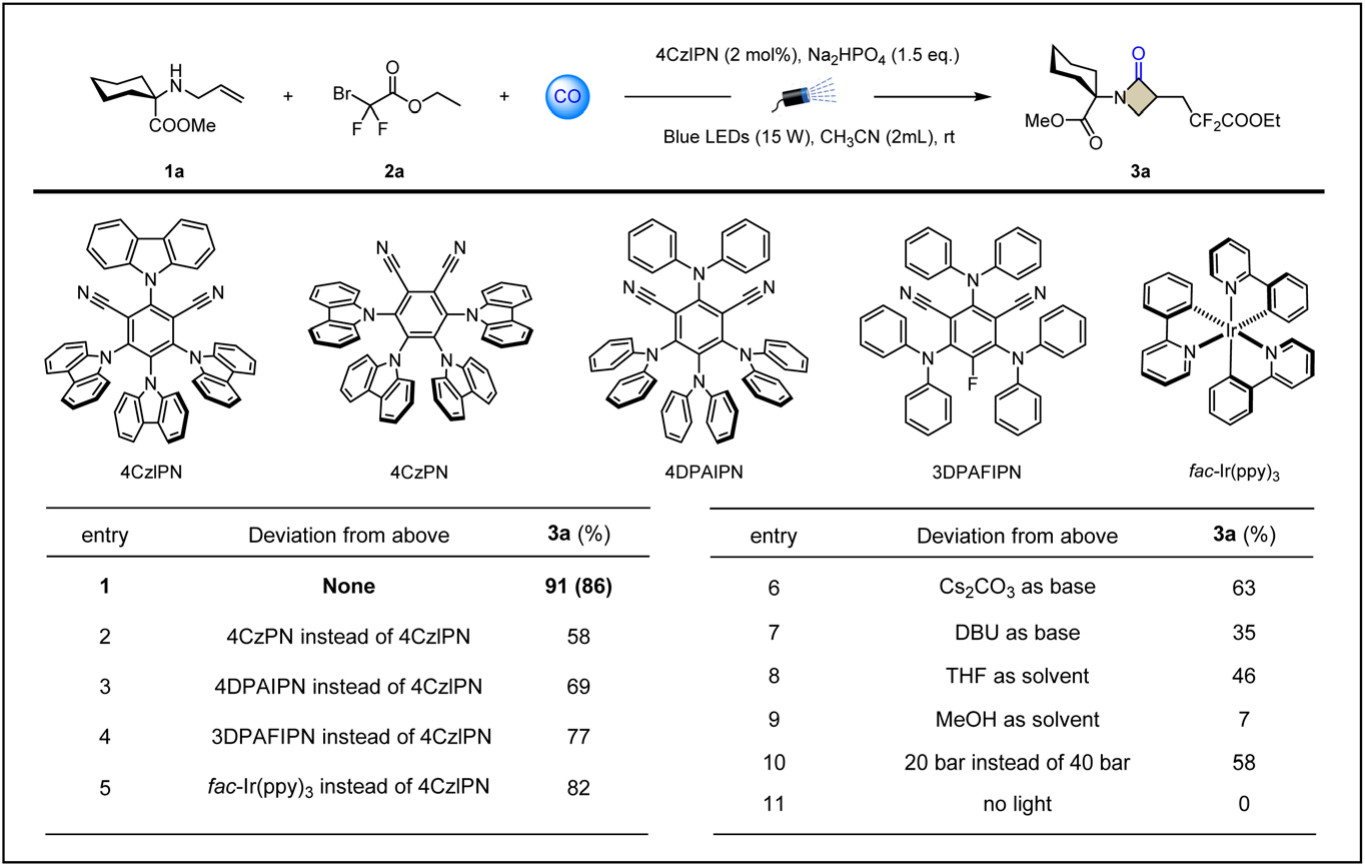

a Carbonylative cyclization to synthesize β-lactams. Reaction conditions: 1a (0.2 mmol), 2a (0.4 mmol), photocatalyst (2 mol%), base (1.5 equiv.) in solvent (2 mL) at room temperature for 20 h under CO (40 bar), 15 W blue LEDs. Yields were determined by GC-FID analysis using *n*-hexadecane as internal standard. Isolated yields given in brackets.

With the optimal conditions for visible-light-induced radical relay carbonylative annulation to β-lactams (Table 2). A set of disubstituted allylamines derived from natural and unnatural α-amino acids were examined in this platform. Designed carbonylative cyclization of α-cycloalkyl-substituted allylamines afforded bicyclic β-lactams in excellent yields (3b–3d). Two α-benzyl-substituted phenylalanine derivatives 3e and 3f were obtained in 70% and 68% yields, respectively. The conversion of methyl ester to ethyl ester and the spatial configuration had no negative impact. Surprisingly, when sterically bulky substituents, such as leucine derivatives, were introduced at the α-position, the target product 3g was still obtained successfully and maintained a high yield of 82%. Isopropyl and cycloalkyl-substituted substrates also gave 3h (from valine) and 3i smoothly as expected. The implementation of the α-phenyl-substituted case (3j) shows that the ring-closing process of acyl radical aromatic homolysis substitution has been effectively avoided. Derivatives of amino acids containing more complex functional groups such as aspartic acid, threonine, asparagine, and methionine can also be accurately converted into corresponding β-lactams (3k–3o). These cases are often difficult to be compatible in transition-metal-catalyzed systems. Incorporation of malonate substituents such as 3p provides a pathway for the synthesis of lactam backbones with multi-ester-based structures. The α-unsubstituted glycine derivative (3q–3r) and α-dimethyl-substituted 3s showed good yields, which is another indication that this model is less sensitive to steric resistance of the nitrogen α-position. According to this method, a compound 3t, containing the lactone-ring linked β-lactam-ring, was successfully prepared with a yield of 70%, which is difficult to obtain in other methods. Moreover, under standard conditions, allylamines derived from β-amino acids were also smoothly realized to generate target products 3u and 3v in moderate yields. Benzyl and adamantyl substituted allylamines have also been used to perform this carbonylative cyclization. Examples from 3w to 3y further expand the range of amines available in this method. Besides the nucleophilicity issue, the active benzylic C–H bond is partially responsible for the decreased yield. Benzaldehyde was detected in the case of using benzyl amine (3w) as the substrate. It's also worth mentioning that only a trace amount of the desired product was detected when *t*Bu or cyclohexyl amine analogues were tested.

**Table 2:** Scope of allylamines^a^^,^^b^

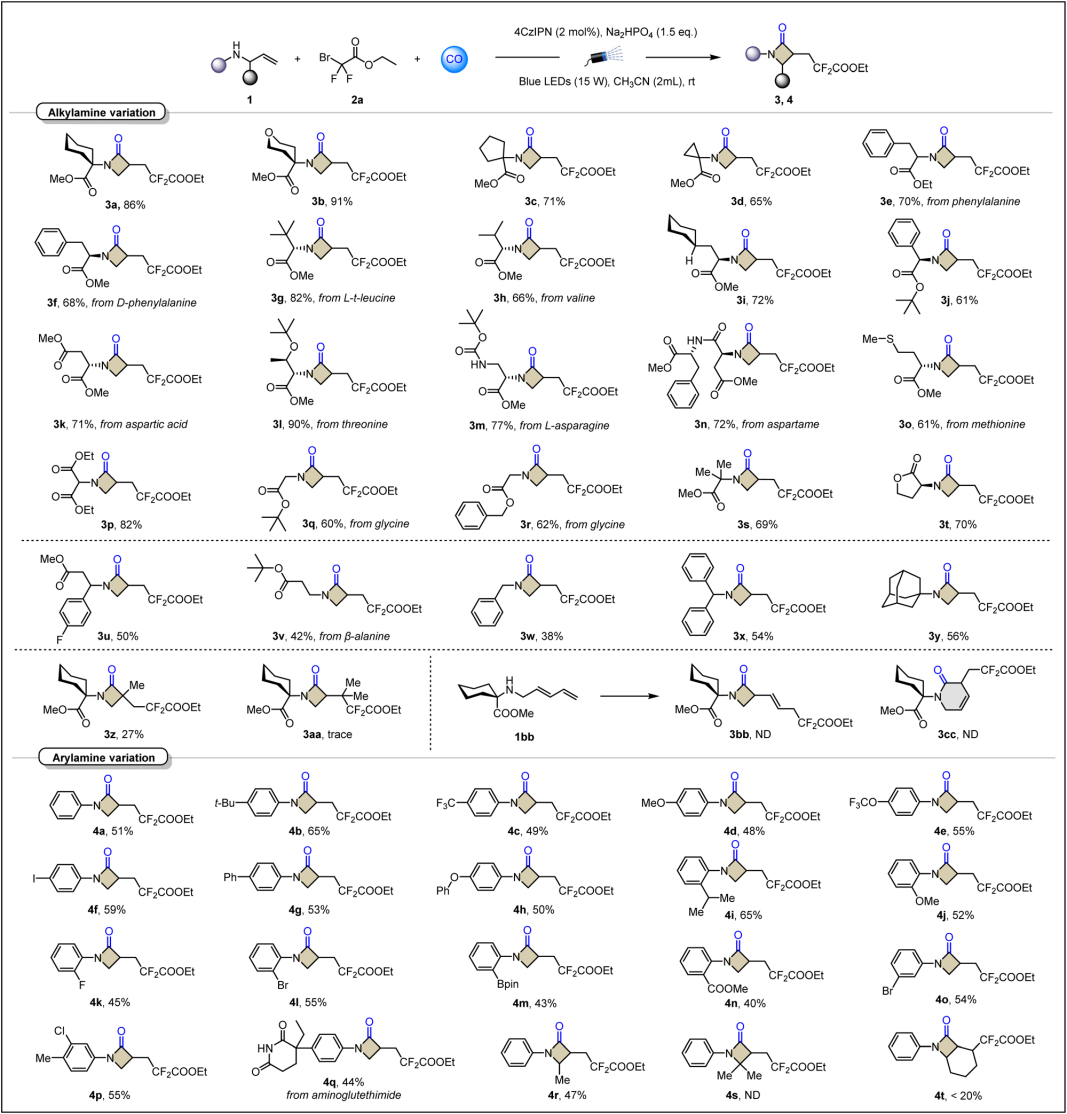

a Reaction conditions: 1 (0.2 mmol), 2a (0.4 mmol), 4CzIPN (2 mol%), base (1.5 equiv.) in CH_3_CN (2 mL) at room temperature for 20 h under CO (40 bar), 15 W blue LEDs.

b The dr. ratio was determined by ^1^H NMR. All yields are isolated yields.

For exploring the applicability of the olefin module, we tested the effect of steric hindrance on the rate of carbon radical capture of CO by introducing a methyl group on the inside of the olefin. The results showed that compared with secondary carbon radicals, tertiary carbon radicals are indeed more difficult to combine with CO even under higher pressures. Therefore, 3z was obtained in a yield of only 27%. When two methyl groups were introduced to the outside of the olefin, the situation became even worse, and only trace amounts of the target product 3aa were detected. The larger steric hindrance not only blocks the addition olefins, but also hinders the insertion of CO. In the case of 1,3-diene, the negative impact of the conjugation effect on the radical addition was also obvious. The raw material 1bb was completely recovered, and no products of either the four-membered ring β-lactam 3bb or the six-membered ring 3cc were detected.

To further showcase the utility of this carbonylative annulation access to β-lactams, we have expanded the class of amines to aromatic amines, and a series of aryl-substituted allylamines were prepared and investigated. The lower oxidation potential of arylamines makes the reaction more unpredictable. Fortunately, various substituent investigations confirmed that arylamines could also work in this scheme. The substitution of the para-substituent does not show a significant difference between the electron-withdrawing group and the electron-donating group (4a–4h). This may be attributed to the fact that nucleophilicity is not the determining factor for the completion of cyclization in this reaction system. It is noteworthy that the iodine functional group, which is sensitive to palladium metal, can be retained intact (4f). This is undoubtedly a good opportunity for the carbonylation transformation of iodoaniline. Subsequent testing of *ortho*-substituents also showed promising results. Generally, it is difficult to overcome large steric hindrance during coupling reactions. However, the isopropyl group at the *ortho*-position completed the β-lactamization more efficiently (4i). Analogous to the case of iodine, the presence of boronate ester also preserves active sites for further coupling (4m). The *meta*-position and multiple substitution investigations were also completed, and the designed product was obtained in good yield (4o and 4p). Examples of natural product β-lactamizative modifications demonstrate the utility of this approach (4q). We also find that the steric hindrance of the allyl site directly determines whether the cyclization can proceed. Increasing the single methyl substitution (4r) to the gem-dimethyl group (4s) completely blocks efficient conversion. In addition, cyclic internal olefin is difficult to exploit to construct bicyclic β-lactam (4t).

Next, we turned our investigation to radical precursors (Table 3). The scope of 2-bromo-2,2-difluoroacetamide derivatives was explored as satisfactory radical precursors for this platform, concluding alkylamine (5a), arylamine (5b), benzylamine (5c), heterocyclic benzylamine (5d) and morpholine (5e). It is noteworthy that some heterocyclic compounds that often exhibit radical sensitivity are still suitable under these standard conditions, such as 5d containing thiophene. In addition, this platform is not limited to ethyl difluorobromoacetate. As an extension of the ester group, other skeletons including diacetone-β-d-fructose (5g and 5l), vitamin E (5i), isoborneol (5j), testosterone (5k), glycine (5n) and menthol (5q) have also been shown to smoothly give the desired cyclization products with remarkable yields. The compatibility of these complex bioactive molecules demonstrates the potential value of the carbonylative cyclization to synthesize β-lactams. Then, we turned our investigation to a broader class of electron-deficient radical precursors. Less commonly used difluoroalkyl ketones (6a) and (6b) difluoroalkyl phosphates also unexpectedly achieved the desired transformations, albeit in only moderate yields. α-Bromo ketones often show excessive activity, and facile hydrogenation limits their use as radical precursors to produce α-carbonyl carbon radicals for functionalization of olefins. We successfully used them as synthons for the phenylketone functional group (6c). Bromoacetonitrile also lived up to expectations by bringing the cyanomethylene group into the β-lactam skeleton (6d). Carbon tetrachloride and Togni reagent (II) also achieved the synthesis of trichloromethyl and trifluoromethyl modified β-lactams in good yields (6e and 6f, respectively). However, the reaction failed when 5-(*p*-tolyl)-5*H*-thianthren-5-ium trifluoromethanesulfonate was tested as an aryl radical precursor under our standard conditions, and no improvement was obtained when iridium photocatalysts were tested.

**Table 3:** Scope of radical precursors^a^^,^^b^^,^^c^^,^^d^

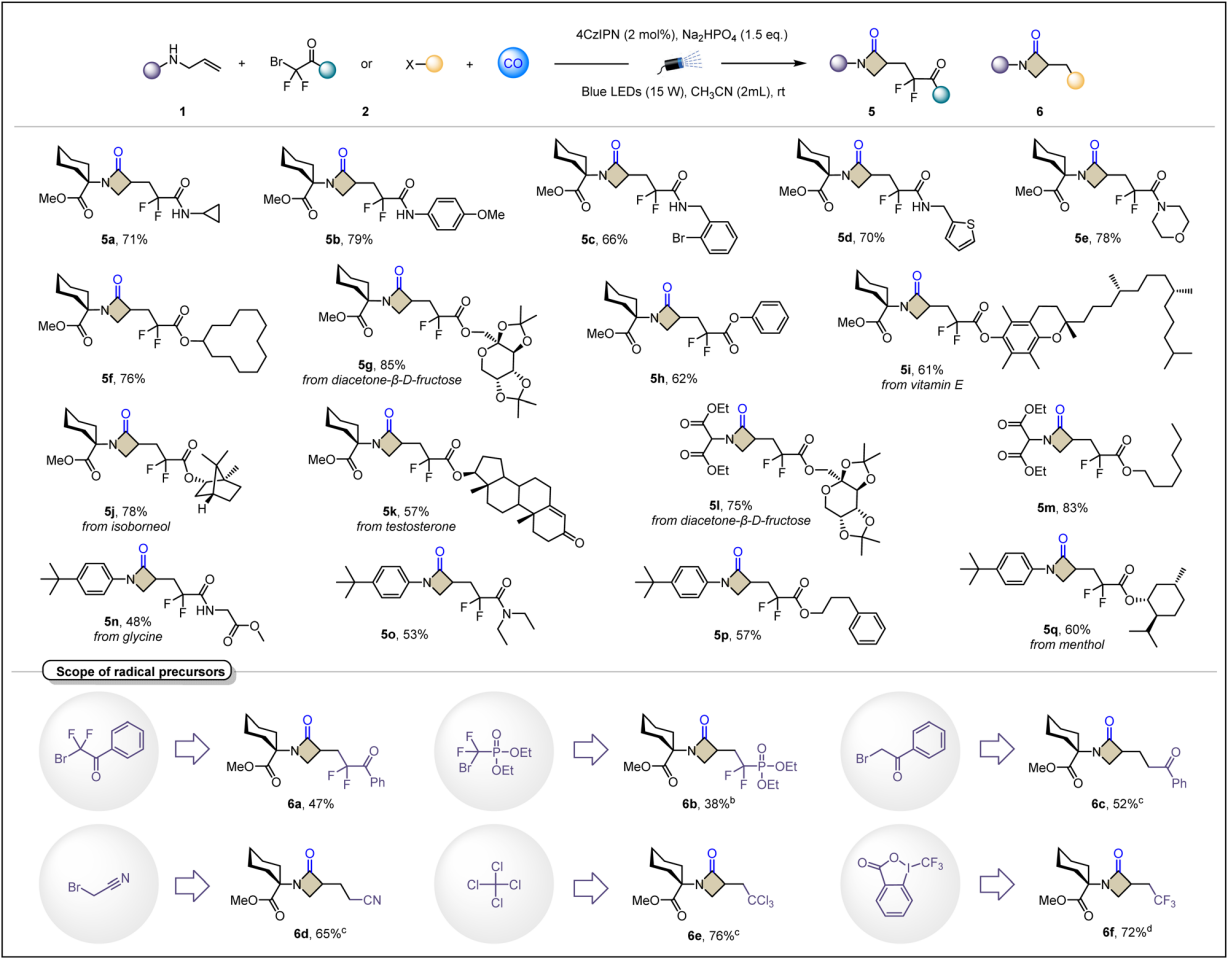

a Reaction conditions: 1 (0.2 mmol), 2 (0.3 mmol), 4CzIPN (2 mol%), base (1.5 equiv.) in CH_3_CN (2 mL) at room temperature for 20 h under CO (40 bar), 15 W blue LEDs.

b Radical precursor (0.4 mmol), 36 h.

c Radical precursor (0.6 mmol), 24 h.

d Togni reagent (II) (0.5 mmol), 24 h. The dr. ratio was determined by ^1^H NMR. All yields are isolated yields.

**Fig. 2:**
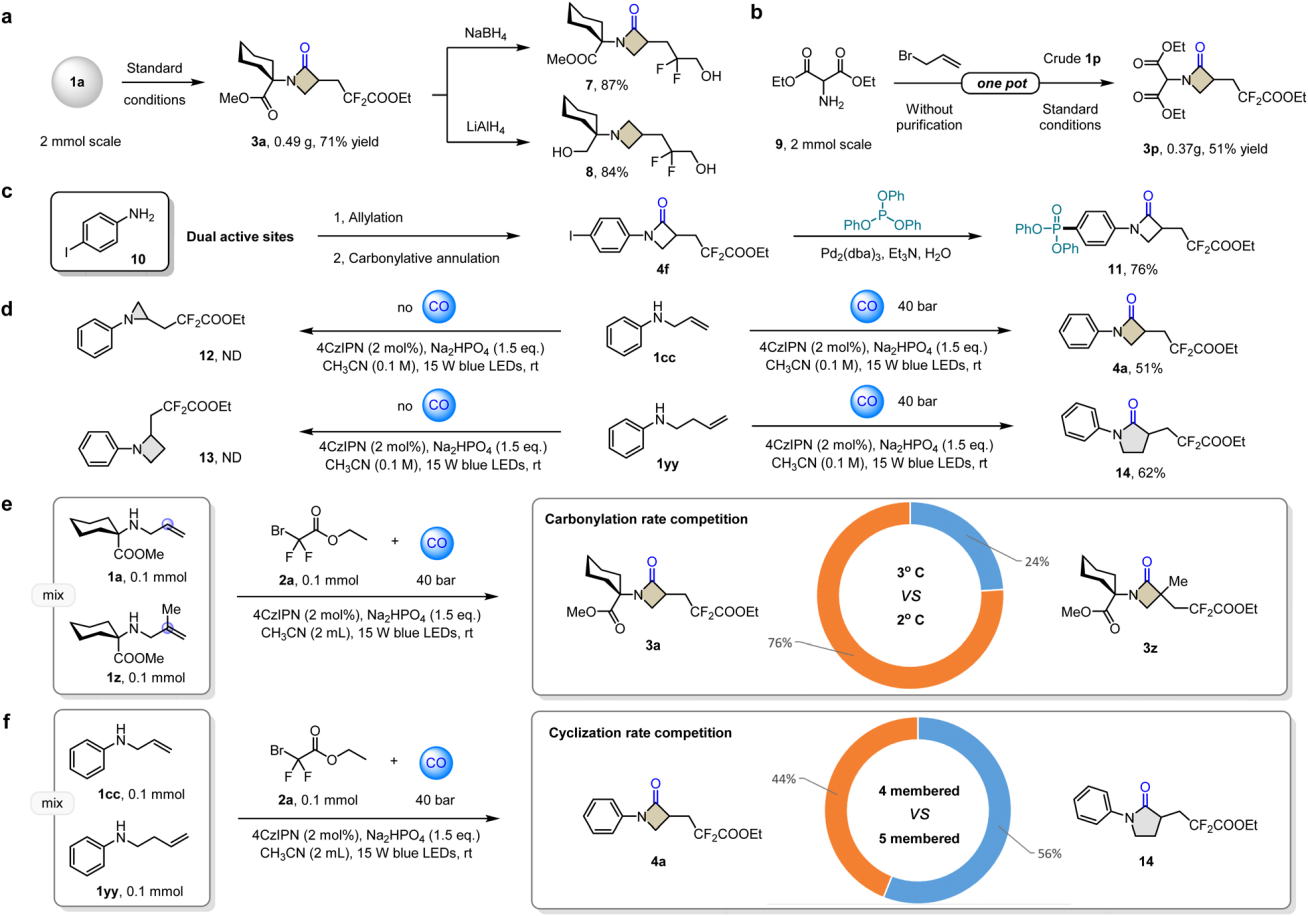
Derivatization and competition experiment. (a) Scale-up and selective reduction of compounds 3a. (b) One-pot scale–up reaction. (c) Transformation of 4f. (d) Comparison of carbonylative and non-carbonylative cyclization. (e) Competition between the carbonylation of secondary and tertiary carbon radicals. (f) Competition between the four-membered and five-membered cyclization.

The template reaction was still feasible while scaled up to 2 mmol, outputting 0.49 g expected product 3a in 71% yield (Fig. 2a). Diversity transformations started with 3a validate the synthetic utility of this method. Considering that 3a contains three different carbonyl groups, selective reduction will lead to unexpected structures. When NaBH_4_ was used, only the difluoroester group was reduced, the β-lactam skeleton 7 with a difluoroalcohol group attached was obtained in 87% yield. It is well known that lactams can be used to prepare the azetidine scaffold. While in the absence of LiAlH_4_, all carbonyl groups are uniformly reduced without distinction. We also successfully obtained the expected azetidine derivative 8 in 84% yield. To overcome the cumbersome problem of multi-step synthesis, one-pot scale–up reactions have also been attempted (Fig. 2b), such as *in situ* allylation of diethyl 2-aminomalonate 9 with allyl bromide, without purification and separation, and with directly adding the photocatalyst and transferring to light reaction conditions in a CO atmosphere. After full conversion, 0.37 g 3p was successfully isolated in 54% yield. 4-Iodoaniline has two active sites in carbonylation chemistry. We imagine that if the conventional reaction sequence can be changed and the active carbon–iodine bonds are retained, then diverse couplings in late-stage can be achieved. Under palladium catalysis, 4f can be coupled with triphenylphosphite to obtain β-lactam-containing aryl phosphate 11, which is difficult to obtain by other methods.

Non-carbonylation control experiments can highlight the differences in ring-forming abilities between different ring systems and different hybrid forms of carbon (Fig. 2c). Under standard conditions, allylamine 1cc cannot directly give nitrogen three-membered ring 12, but under a CO atmosphere, β-lactam 4a can be obtained. Comparing the results of homoallylamine 1yy, the non-carbonyl azetidine 13 was still not produced, but the γ-lactam 14 was produced as expected in the CO atmosphere (Fig. 2d). These results demonstrate the superior cyclization ability of the sp-hybridized acyl radical compared to the carbon radical (sp^2^). When 1a, α-methyl allylamine (1z) and 2a were reacted in a 1 : 1 : 1 ratio, products 3a and 3z account for 76% and 24% respectively (Fig. 2e). This indicates that the secondary carbon radical has a stronger ability to capture CO than the tertiary carbon radical, which may be attributed to the faster reverse reaction rate of decarbonylation of the tertiary carbon acyl radical. When allylamine 1cc and homoallylamine 1yy were mixed in equal amounts and reacted, the ratio of 44% to 56% between 4a and 14 indicated that there was no significant difference between the 4-membered cyclization and the 5-membered cyclization in the carbonylative cyclization (Fig. 2f). This result seems unusual in the well-known guidelines governing the ease of ring-closing reactions.

**Fig. 3:**
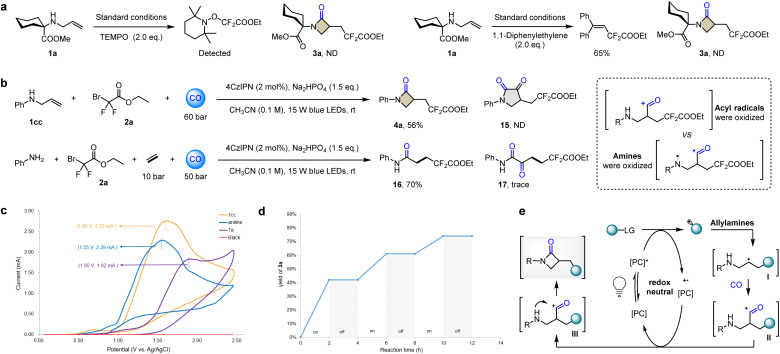
Mechanistic studies of photoinduced carbonylative annulation to β-lactams. (a) Radical inhibition experiment. (b) Elevated pressure experiments and control experiments of intermolecular amine carbonylation. (c) Evaluation of the redox potentials of different types of amines by cyclic voltammetry in acetonitrile. (d) Light off/on experiments. (e) Possible mechanism.

To obtain more clear understanding of this possible radical carbonylative annulation process, we designed and performed a series of mechanistic experiments. When additional stoichiometric amounts of the radical scavenger TEMPO (2,2,6,6-tetramethylpiperidine-1-oxyl) or 1,1-diphenylethene were added to the model reaction of 1a under standard conditions, the formation of the desired β-lactam 3a was completely suppressed, and the starting material 1a was fully recovered (Fig. 3a). The radical adducts were detected or isolated to verify the presence of radical species in the reaction process. Then, we increased the CO pressure to attempt to confirm the presence of double carbonylation species. If the double carbonylative product becomes the main product, it means that the amine in the substrate has been oxidized to a nitrogen radical.^17^ According to the existing research conclusions, the competition between single carbonylation and double carbonylation is reliable evidence for which of the acyl radical and amine nucleophile reacts preferentially through single-electron oxidation. And this doubt mainly arises from aromatic amines, so we use 1cc as the model substrate for testing. In this type of catalytic cycle, if the acyl radical is first oxidized, the acyl cation species can only be quenched by the amine nucleophile to obtain a single carbonylation product. For the template substrate 1cc, 60 bar CO pressure slightly increased the yield of 4a from 51% to 56% (Fig. 3b). This single carbonylation β-lactam 4a was the only product detected and isolated. Considering that 15 may be unstable, this would interfere with the analysis of the reaction pathway. We use ethylene and aniline to replace 1cc, and the intermolecular reaction avoids the emergence of unstable factors in the product. The single carbonyl amidation 16 shows an overwhelming advantage and was isolated in 70% yield. Even though double carbonylation 17 was detected, it was only in trace amounts (Fig. 3b).

To further illustrate how the redox potential regulates the selectivity of intermediate oxidation, we performed cyclic voltammetry experiments on three types of amines: phenylallylamine (orange line; Fig. 3c), aniline (blue line; Fig. 3c) and alkylallylamine (purple line; Fig. 3c). Phenylallylamine showed an oxidation potential peak at *E*_p_ = 1.65 V *vs.* Ag/AgCl, which is close to the oxidation potential of aniline (*E*_p_ = 1.55 V *vs.* Ag/AgCl). Obviously, in agreement with our elevated pressure experimental studies (Fig. 3b), the nucleophile amine cannot be oxidized before the acyl radical. The photocatalyst in the oxidized state preferentially oxidizes the acyl radicals to acyl cations. In fact, luminescence quenching experiments with organic photosensitizers support that 4CzIPN can complete this catalytic cycle.^18^ Compared with the oxidation potential of the acyl radical to the acyl cation,^19^ undoubtedly, *N*-alkyl alkylamines require a higher oxidation potential (1a, purple line; Fig. 3c, *E*_p_ = 1.95 V *vs.* Ag/AgCl). Based on the above results, it can be inferred that nitrogen radical species probably did not appear in this reaction system.^20^

Then we performed on-off light experiments. As shown in Fig. 3d, when the reaction was under continuous irradiation with blue light, the yield of 3a gradually increased. Once the light was turned off, the yield remained constant. These results could support the necessity of light and identify the catalytic cycle of the photosensitizer as the main pathway in the current system, rather than the radical chain process. Based on the above accumulation, we proposed a possible mechanism (Fig. 3e). The excited photosensitizer single electron reduced the radical precursor to generate an electrophilic fluoroalkyl reactive radical species. Allylamine acts as the radical acceptor, and the double bond is added to generate the corresponding alkyl carbon radical I. Under a certain pressure atmosphere, CO was captured to produce key β-aminoacyl radical intermediates II, which could be oxidized rapidly by the oxidation state photocatalyst to form an acyl cation III. Subsequently, the nucleophilic addition of the intramolecular amine quenching reaction completes the cyclization and obtains β-lactams.

## Conclusions

In conclusion, we have demonstrated a diversity-oriented radical relay carbonylative annulation strategy to access densely functionalized β-lactams from abundant substituted allylamines. Importantly, a wide range of primary amines, including alkyl, aryl, benzyl, and especially amino acid derivatives, can all serve as the nitrogen source, expanding the synthetic repertoire for β-lactam manufacturing. In addition, the compatibility of electron-deficient radicals also makes it possible to introduce fluoroalkyl, trichloromethyl, cyano, and ketone groups into the β-lactam skeleton. In this mild photocatalytic synthetic platform, the efficient, and selective radical relay process revealed that carbonylative annulation is compatible with radical photochemistry. The end-to-end connection pattern of acyl radicals and nucleophiles updates the construction mode of the β-lactam skeleton and discloses the uniqueness of carbonyl groups in the 4-membered strained ring formation process. It is conceivable that this method is very promising in synthetic β-lactams, especially combining amino acid fragments and β-lactam skeletons will attract widespread attention in the field of new drug discovery.

## Data availability

The data supporting this article have been included as part of the ESI.†

## Author contributions

Y. W. designed and carried out most of the reactions and analyzed the data. X.-F. W. designed and supervised the project. Y. W., X. Q. and Z.-P. B. provided raw material support. X.-F. W. and Y. W. wrote and revised the manuscript.

## Conflicts of interest

There are no conflicts to declare.

## Supplementary Material

SC-016-D5SC02418H-s001
