# Supplementary material for: Photoinduced carbonylative annulation access to β-lactams
Source: Chem Sci. 2025 Apr 25;16(22):9872–80. doi: 10.1039/d5sc02418h (PMC12046347; doi:10.1039/d5sc02418h)
Supplement: SC-016-D5SC02418H-s001 [file SC-016-D5SC02418H-s001.pdf]

Supplementary Information (SI) for Chemical Science.
This journal is © The Royal Society of Chemistry 2025

Supporting Information

Table of Contents

1. General information....S2
2. Reaction optimization....S3
3. Synthesis of the starting materials....S6
4. List of the starting materials....S14
5. Scale-up and derivatization....S15
6. Investigation of reaction mechanism.....S17
7. Cyclic Voltammograms S18
8. Characterization of Products....S19
9. Copy of NMR Spectrum....S45
10. References....S211

1. General Information

Unless otherwise noted, all reactions were carried out under a carbon monoxide or nitrogen atmosphere. All reagents were from commercial sources, all solvents are extra dry solvents and used as received without further purification. Column chromatography was performed on silica gel (200-300 meshes) using petroleum ether (b.p. 60-90 ^{o}C) and ethyl acetate as the eluents. ^{1}H and ^{13}C NMR spectra were taken on Bruker AVANCE III 400 MHz or 700 MHz spectrometers and spectral data were reported in ppm relative to tetramethylsilane (TMS) as the internal standard and $CDCl_3$ or DMSO-D_6 as solvent. All coupling constants (J) are reported in Hz with the following abbreviations: s = singlet, d = doublet, dd = double doublet, t = triplet, dt = double triplet, q = quatriplet, m = multiplet, br = broad. Gas chromatography (GC) analyses were performed on an Agilent HP-7890A instrument with a FID detector and HP-5 capillary column (polydimethylsiloxane with 5% phenyl groups, 30 m, 0.32 mm i.d. 0.25 μm film thickness) using argon as carrier gas. Gas chromatography mass spectrometer (GC-MS) analyses were performed on a Shimadzu QP2020 NX instrument. High resolution mass spectra (HRMS) were recorded on Agilent 8890-7250 and Agilent Q-TOF 6540. DFT calculation was carried out by Gaussian 09. Because of the high toxicity of carbon monoxide, all of the reactions should be performed in an autoclave. The laboratory should be well-equipped with a CO detector and alarm system.

2. Reaction Optimization

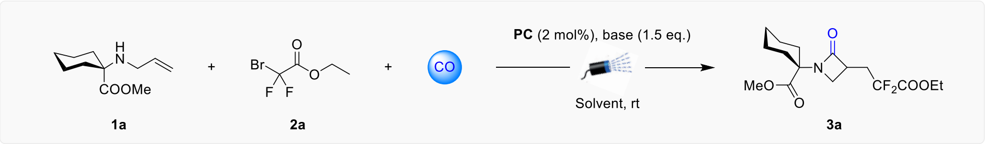

A 4 mL screw-cap vial was charged with **1a** (0.2 mmol), **2a** (0.4 mmol), photocatalyst (2 mol%), base (0.3 mmol), and solvent (2 mL) under N_2 atmosphere. The vial was closed with a Teflon septum and cap and connected to the atmosphere via a needle. The closed autoclave was flushed two times with N_2 (~ 5 bar), and a pressure of 40 bar CO were charged. The autoclave was then placed on a magnetic stirrer. The reaction mixture was stirred while being irradiated with 15 W blue light at room temperature for 20 h. After irradiation, the light was turned off and the pressure was released carefully. The mixture was concentrated under vacuum. The crude product was purified by column chromatography (PE/EA =10/1 to 5/1) on silica gel to afford the corresponding products. Note: Because of the high toxicity of carbon monoxide, all the reactions should be performed in an autoclave. The laboratory should be well-equipped with a CO detector and alarm system.

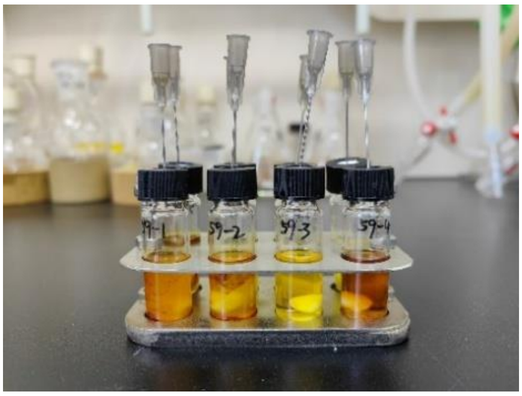

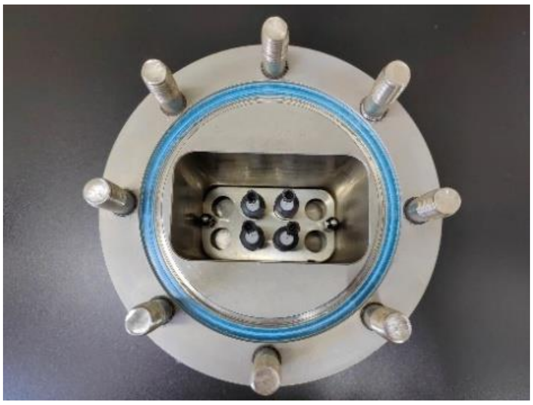

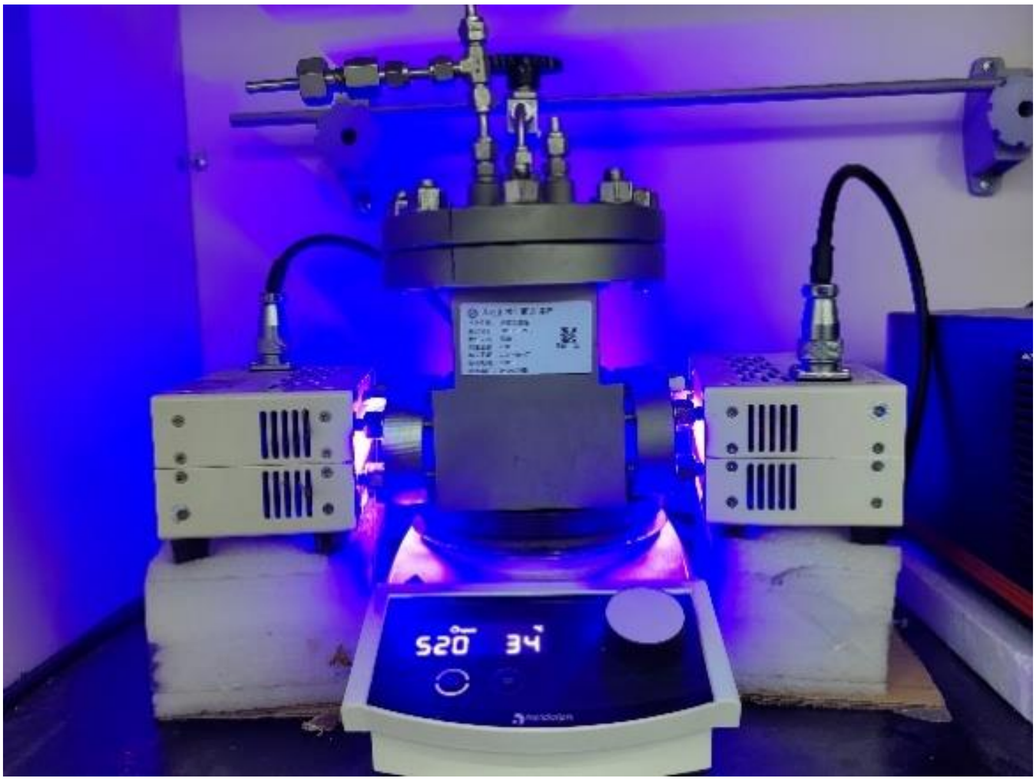

Supplementary Table 1. Screening of photocatalyst

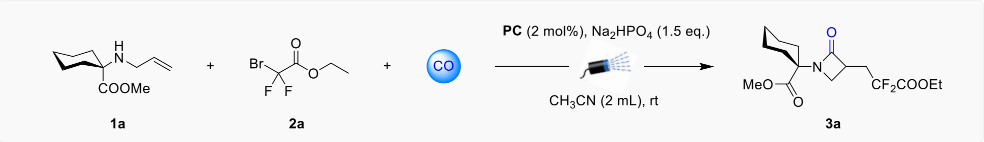

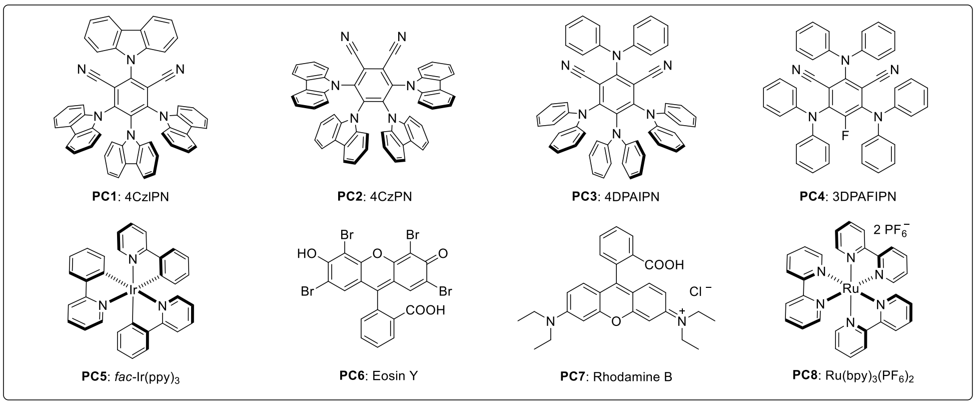

entry	PC	base	solvent	**3a** (%)[a]
**1**	**PC1**	**Na_2HPO_4**	**CH_3CN**	**91 (86)**
2	PC2	Na_2HPO_4	CH_3CN	58
3	PC3	Na_2HPO_4	CH_3CN	69
4	PC4	Na_2HPO_4	CH_3CN	77
5	PC5	Na_2HPO_4	CH_3CN	82
6	PC6	Na_2HPO_4	CH_3CN	24
7	PC7	Na_2HPO_4	CH_3CN	6
8	PC8	Na_2HPO_4	CH_3CN	36

[a] yields were determined by GC-FID analysis using n-hexadecane as internal standard. Isolated yields given in brackets.

Supplementary Table 2. Screening of base

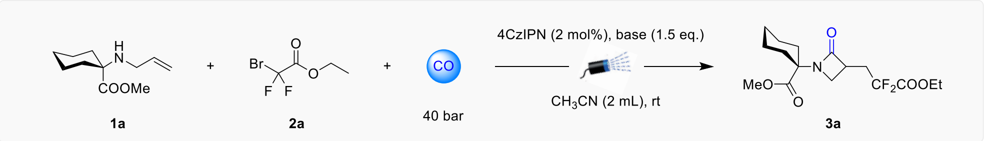

entry	PC	base	solvent	**3a** (%)[a]
1	PC1	$NaHCO_3$	CH_3CN	83
2	PC1	Na_2CO_3	CH_3CN	76
3	PC1	K_2CO_3	CH_3CN	71
4	PC1	Cs_2CO_3	CH_3CN	63
5	PC1	Et_3N	CH_3CN	86
6	PC1	DBU	CH_3CN	35

[a] yields were determined by GC-FID analysis using n-hexadecane as internal standard.

Supplementary Table 3. Screening of solvents

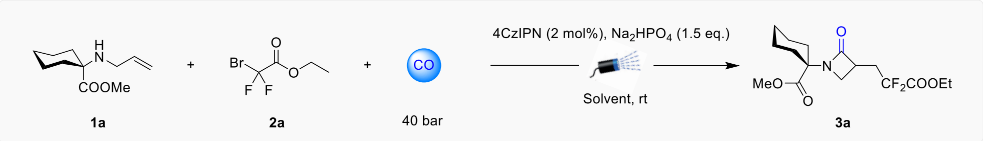

entry	PC	base	solvent	**3a** (%)[a]
1	PC1	Na_2HPO_4	THF	46
2	PC1	Na_2HPO_4	DMF	70
3	PC1	Na_2HPO_4	DCE	50
4	PC1	Na_2HPO_4	Toluene	63
5	PC1	Na_2HPO_4	Acetone	82
6	PC1	Na_2HPO_4	MeOH	7

[a] yields were determined by GC-FID analysis using n-hexadecane as internal standard.

Supplementary Table 4. Screening of the pressure of CO

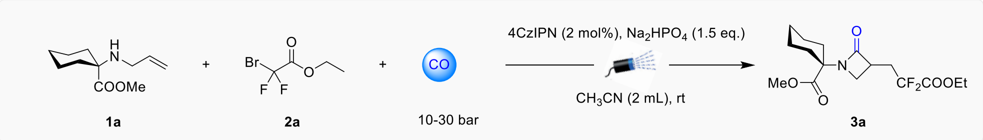

entry	PC	base	solvent	pressure of CO	**3a** (%)[a]
1	PC1	Na_2HPO_4	CH_3CN	30 bar	75
2	PC1	Na_2HPO_4	CH_3CN	20 bar	58
3	PC1	Na_2HPO_4	CH_3CN	10 bar	36

[a] yields were determined by GC-FID analysis using n-hexadecane as internal standard.

3. Synthesis of the starting materials

General Procedure A:

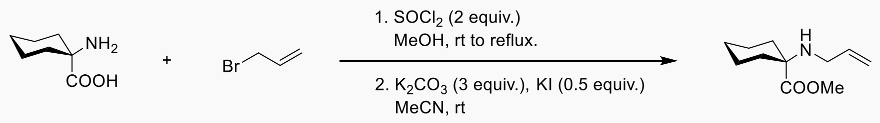

To a stirred solution of 1-aminocyclohexanecarboxylic acid (5 mmol, 1.0 equiv.) in MeOH (15 mL) was added $SOCl_2$ (10 mmol, 2.0 equiv.) slowly at ambient temperature (*note: this addition was very exothermic*). The solution was heated to reflux and stirred for another 2 h at this temperature. Methanol was removed in vacuo and the crude material was dissolved in MeCN (15 mL), K_2CO_3 (15 mmol, 3.0 equiv.) and KI (2.5 mmol, 0.5 equiv.) were added. Then, allyl bromide (5 mmol, 1.0 equiv.) was added at room temperature and stirred overnight. The reaction mixture was then filtered and washed with DCM. The filtrate was then concentrated at reduced pressure. The aimed allylamine was purified by flash chromatography (silica gel, petroleum ether/ethyl acetate = 10:1).

General Procedure B:

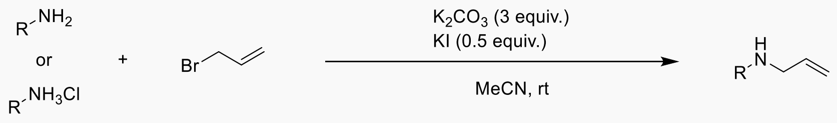

To a solution of amine or amine hydrochloride (5 mmol, 1 equiv.), K_2CO_3 (15 mmol, 3.0 equiv.) and KI (2.5 mmol, 0.5 equiv.) in MeCN (15 mL). Allyl bromide (5 mmol, 1.0 equiv.) was added at room temperature and stirred overnight. The reaction mixture was then filtered and washed with DCM. The filtrate was then concentrated at reduced pressure. The aimed allylamine was purified by flash chromatography (silica gel, petroleum ether/ethyl acetate = 10:1 to 1:1).

General Procedure C:

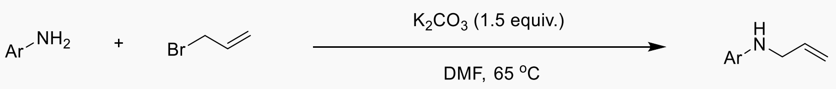

To a solution of aniline (6 mmol, 2.0 equiv) in DMF (15 mL) was added K_2CO_3 (4.5 mmol, 1.2 equiv) and allyl bromide (3 mmol, 1.0 equiv). The reaction mixture was stirred at 65 °C overnight and then cooled to room temperature. The reaction was quenched with H_2O (10 mL). The mixture was extracted with ethyl acetate (15 mL × 3). The combined organic layer was washed with brine (15 mL), dried over Na_2SO_4, filtered, and concentrated in vacuo. The crude product was purified by flash chromatography to afford aimed allylamine.

Methyl 1-(allylamino)cyclohexane-1-carboxylate (1a)

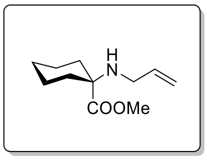

1a was prepared according to the **GP1** in 74% yield.

^{1}H NMR (400 MHz, $CDCl_3$) δ 5.93 – 5.83 (m, 1H), 5.22 – 5.00 (m, 2H), 3.71 (s, 3H), 3.09 – 3.03 (m, 2H), 1.98 – 1.88 (m, 2H), 1.72 – 1.65 (m, 2H), 1.56 – 1.36 (m, 7H).

^{13}C NMR (101 MHz, $CDCl_3$) δ 176.6, 136.9, 115.7, 61.7, 51.6, 46.2, 33.4, 25.7, 22.0.

HRMS (ESI-TOF) m/z: $[M+H]^+$ Calcd. for $C_{11}H_{20}NO_2$ 198.1489; found: 198.1497.

Methyl 4-(allylamino)tetrahydro-2*H*-pyran-4-carboxylate (1b)

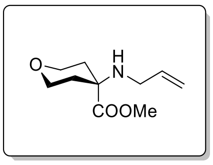

1b was prepared according to the **GP2** in 87% yield.

^{1}H NMR (400 MHz, $CDCl_3$) δ 5.97 – 5.77 (m, 1H), 5.24 – 5.03 (m, 2H), 3.93 – 3.84 (m, 2H), 3.74 (s, 3H), 3.61 – 3.54 (m, 2H), 3.09 – 3.05 (m, 2H), 2.09 – 2.01 (m, 2H), 1.68 – 1.62 (m, 2H), 1.52 (s, 1H).

^{13}C NMR (101 MHz, CDCl3) δ 175.6, 136.6, 115.8, 63.7, 59.3, 51.8, 46.0, 33.4.

HRMS (ESI-TOF) m/z: $[M+H]^+$ Calcd. for $C_{11}H_{18}NO_3$ 200.1281; found: 200.1289.

Methyl 1-(allylamino)cyclopentane-1-carboxylate (1c)

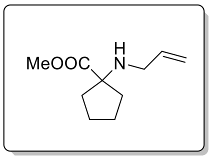

1c was prepared according to the **GP2** in 84% yield.

^{1}H NMR (400 MHz, $CDCl_3$) δ 5.94 – 5.81 (m, 1H), 5.21 – 5.01 (m, 2H), 3.71 (s, 3H), 3.08 (d, *J* = 6.0 Hz, 2H), 2.08 – 1.99 (m, 2H), 1.83 – 1.65 (m, 7H).

^{13}C NMR (101 MHz, $CDCl_3$) δ 177.4, 136.8, 115.7, 70.0, 51.8, 48.0, 36.6, 24.6.

HRMS (ESI-TOF) m/z: $[M+H]^+$ Calcd. for $C_{10}H_{18}NO_2$ 184.1332; found: 184.1338.

Methyl 1-(allylamino)cyclopropane-1-carboxylate (1d)

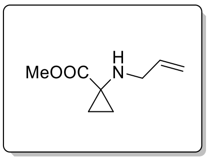

1d was prepared according to the **GP2** in 65% yield.

^{1}H NMR (400 MHz, $CDCl_3$) δ 5.99 – 5.77 (m, 1H), 5.22 – 5.01 (m, 2H), 3.69 (s, 3H), 3.34 (dd, *J* = 6.0, 1.2 Hz, 2H), 2.14 (s, 1H), 1.30 – 1.19 (m, 2H), 1.05 – 0.96 (m, 2H).

^{13}C NMR (101 MHz, $CDCl_3$) δ 175.7, 136.8, 115.7, 51.9, 50.4, 40.7, 17.5.

HRMS (ESI-TOF) m/z: $[M+H]^+$ Calcd. for $C_8H_{14}NO_2$ 156.1019; found: 156.1017.

Ethyl allylphenylalaninate (1e)

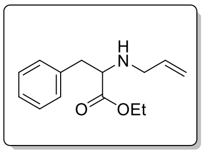

1e was prepared according to the **GP2** in 79% yield.

^{1}H NMR (400 MHz, CDCl$_3$) δ 7.30 – 7.25 (m, 2H), 7.23 – 7.17 (m, 3H), 5.88 – 5.74 (m, 1H), 5.16 – 5.03 (m, 2H), 4.09 (q, *J* = 7.1 Hz, 2H), 3.53 (t, *J* = 6.9 Hz, 1H), 3.31 – 3.23 (m, 1H), 3.15 – 3.08 (m, 1H), 3.01 – 2.89 (m, 2H), 1.63 (s, 1H), 1.15 (t, *J* = 7.1 Hz, 3H).

^{13}C NMR (101 MHz, CDCl$_3$) δ 174.6, 137.3, 136.2, 129.2, 128.4, 126.7, 116.4, 62.1, 60.6, 50.6, 39.8, 14.2.

HRMS (ESI-TOF) m/z: [M+H]$^+$ Calcd. for $C_{14}H_{20}NO_2$ 234.1489; found: 234.1496.

Methyl allyl-*D*-phenylalaninate (1f)

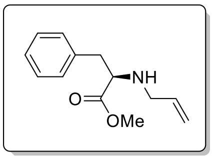

1f was prepared according to the **GP2** in 76% yield.

^{1}H NMR (400 MHz, CDCl$_3$) δ 7.30 – 7.25 (m, 2H), 7.23 – 7.15 (m, 3H), 5.86 – 5.73 (m, 1H), 5.16 – 5.00 (m, 2H), 3.63 (s, 3H), 3.55 (t, *J* = 6.8 Hz, 1H), 3.29 – 3.20 (m, 1H), 3.15 – 3.06 (m, 1H), 2.95 (d, *J* = 6.9 Hz, 2H), 1.64 (s, 1H).

^{13}C NMR (101 MHz, CDCl$_3$) δ 175.0, 137.2, 136.1, 129.2, 128.4, 126.7, 116.4, 62.1, 51.6, 50.6, 39.7.

HRMS (ESI-TOF) m/z: [M+H]$^+$ Calcd. for $C_{13}H_{18}NO_2$ 220.1332; found: 220.1340.

Methyl (*S*)-2-(allylamino)-3,3-dimethylbutanoate (1g)

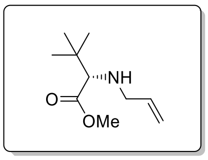

1g was prepared according to the **GP2** in 91% yield.

^{1}H NMR (400 MHz, CDCl$_3$) δ 5.91 – 5.75 (m, 1H), 5.21 – 5.02 (m, 2H), 3.71 (s, 3H), 3.28 – 3.20 (m, 1H), 3.05 – 2.97 (m, 1H), 2.91 (s, 1H), 1.64 (s, 1H), 0.96 (s, 9H).

^{13}C NMR (101 MHz, CDCl$_3$) δ 175.7, 136.7, 116.2, 69.6, 51.5, 51.0, 33.9, 26.7.

HRMS (ESI-TOF) m/z: [M+H]$^+$ Calcd. for $C_{10}H_{20}NO_2$ 186.1489; found: 186.1492.

Methyl allyl-*L*-valinate (1h)

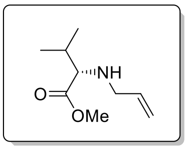

1h was prepared according to the **GP2** in 82% yield.

^{1}H NMR (400 MHz, CDCl$_3$) δ 5.91 – 5.78 (m, 1H), 5.20 – 5.05 (m, 2H), 3.72 (s, 3H), 3.27 (dd, *J* = 13.9, 5.8 Hz, 1H), 3.11 – 2.97 (m, 2H), 1.97 – 1.85 (m, 1H), 1.61 (s, 1H), 0.99 – 0.90 (m, 6H).

^{13}C NMR (101 MHz, CDCl$_3$) δ 175.7, 136.6, 116.2, 66.4, 51.4, 51.2, 31.6, 19.2, 18.7.
HRMS (ESI-TOF) m/z: $[M+H]^+$ Calcd. for $C_9H_{18}NO_2$ 172.1332; found: 172.1335.

Methyl (*R*)-2-(allylamino)-3-cyclohexylpropanoate (1i)

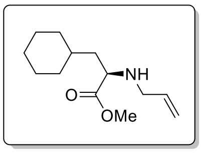

1i was prepared according to the **GP2** in 75% yield.
^{1}H NMR (400 MHz, CDCl$_3$) δ 5.90 – 5.77 (m, 1H), 5.22 – 5.02 (m, 2H), 3.77 – 3.65 (m, 3H), 3.36 – 3.29 (m, 1H), 3.29 – 3.20 (m, 1H), 3.14 – 3.05 (m, 1H), 1.75 – 1.56 (m, 6H), 1.50 – 1.36 (m, 3H), 1.28 – 1.10 (m, 3H), 0.96 – 0.83 (m, 2H).
^{13}C NMR (101 MHz, CDCl$_3$) δ 176.5, 136.4, 116.3, 58.4, 51.5, 50.8, 41.4, 34.2, 33.4, 33.0, 26.5, 26.2, 26.1.
HRMS (ESI-TOF) m/z: $[M+H]^+$ Calcd. for $C_{13}H_{24}NO_2$ 226.1802; found: 226.1807.

***tert*-Butyl (*R*)-2-(allylamino)-2-phenylacetate (1j)**

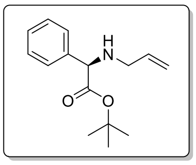

1j was prepared according to the **GP2** in 72% yield.
^{1}H NMR (400 MHz, CDCl$_3$) δ 7.38 – 7.23 (m, 5H), 5.95 – 5.83 (m, 1H), 5.20 – 5.06 (m, 2H), 4.27 (s, 1H), 3.25 – 3.12 (m, 2H), 2.08 (s, 1H), 1.39 (s, 9H).
^{13}C NMR (101 MHz, CDCl$_3$) δ 172.3, 138.7, 136.3, 128.5, 127.7, 127.3, 116.5, 81.4, 65.1, 50.2, 27.9.
HRMS (ESI-TOF) m/z: $[M+H]^+$ Calcd. for $C_{15}H_{22}NO_2$ 248.1645; found: 248.1652.

Dimethyl allyl-*L*-aspartate (1k)

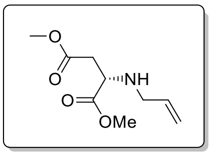

1k was prepared according to the **GP2** in 65% yield.
^{1}H NMR (400 MHz, CDCl$_3$) δ 5.93 – 5.73 (m, 1H), 5.24 – 5.04 (m, 2H), 3.75 (s, 3H), 3.69 (s, 3H), 3.68 – 3.64 (m, 1H), 3.36 – 3.28 (m, 1H), 3.22 – 3.15 (m, 1H), 2.79 – 2.62 (m, 2H), 1.92 (s, 1H).
^{13}C NMR (101 MHz, CDCl$_3$) δ 174.1, 171.3, 136.1, 116.6, 56.6, 52.1, 51.8, 50.6, 37.9.
HRMS (ESI-TOF) m/z: $[M+H]^+$ Calcd. for $C_9H_{16}NO_4$ 202.1074; found: 202.1080.

Methyl N-allyl-O-(*tert*-butyl)-*L*-threoninate (1l)

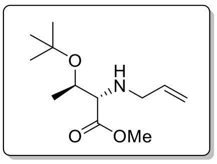

1l was prepared according to the **GP2** in 85% yield.

^{1}H NMR (400 MHz, CDCl$_3$) δ 5.93 – 5.77 (m, 1H), 5.24 – 5.02 (m, 2H), 4.01 – 3.91 (m, 1H), 3.72 (s, 3H), 3.34 (dd, *J* = 13.9, 5.6 Hz, 1H), 3.14 (d, *J* = 4.0 Hz, 1H), 3.07 (dd, *J* = 13.9, 6.4 Hz, 1H), 1.91 (s, 1H), 1.23 (d, *J* = 6.2 Hz, 3H), 1.14 (s, 9H).
^{13}C NMR (101 MHz, CDCl$_3$) δ 174.2, 136.7, 116.1, 73.7, 68.4, 66.1, 51.5, 51.0, 28.3, 20.4.
HRMS (ESI-TOF) m/z: [M+H]$^+$ Calcd. for $C_{12}H_{24}NO_3$ 230.1751; found: 230.1758.

Methyl (*S*)-2-(allylamino)-3-((*tert*-butoxycarbonyl)amino)propanoate (1m)

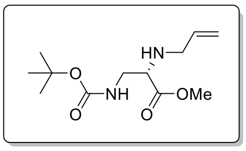

1m was prepared according to the **GP2** in 68% yield.
^{1}H NMR (400 MHz, CDCl$_3$) δ 5.90 – 5.76 (m, 1H), 5.25 – 5.07 (m, 3H), 3.74 (s, 3H), 3.50 – 3.34 (m, 2H), 3.34 – 3.23 (m, 2H), 3.16 (dd, *J* = 13.9, 5.9 Hz, 1H), 1.86 (s, 1H), 1.44 (s, 9H).
^{13}C NMR (101 MHz, CDCl$_3$) δ 173.8, 155.8, 136.0, 116.6, 79.4, 60.0, 52.1, 50.5, 42.5, 28.3.
HRMS (ESI-TOF) m/z: [M+H]$^+$ Calcd. for $C_{12}H_{23}N_2O_4$ 259.1652; found: 259.1655.

Methyl (*S*)-3-(allylamino)-4-(((*R*)-1-methoxy-1-oxo-3-phenylpropan-2-yl)amino)-4-oxobutanoate (1n)

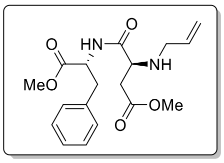

1n was prepared according to the **GP1** in 73% yield.
^{1}H NMR (400 MHz, CDCl$_3$) δ 7.75 (d, *J* = 8.3 Hz, 1H), 7.31 – 7.21 (m, 3H), 7.14 (d, *J* = 6.8 Hz, 2H), 5.84 – 5.71 (m, 1H), 5.18 – 5.01 (m, 2H), 4.92 – 4.78 (m, 1H), 3.69 (d, *J* = 16.7 Hz, 6H), 3.45 (dd, *J* = 8.0, 4.1 Hz, 1H), 3.19 – 3.04 (m, 4H), 2.73 (dd, *J* = 16.6, 4.1 Hz, 1H), 2.55 (dd, *J* = 16.6, 8.0 Hz, 1H), 1.82 (s, 1H).
^{13}C NMR (101 MHz, CDCl$_3$) δ 172.4, 172.0, 171.8, 136.0, 135.8, 129.3, 128.6, 127.1, 116.5, 58.0, 52.7, 52.3, 51.9, 50.3, 38.0, 35.8.
HRMS (ESI-TOF) m/z: [M+H]$^+$ Calcd. for $C_{18}H_{25}N_2O_5$ 349.1758; found: 349.1762.

Methyl allyl-*L*-methioninate (1o)

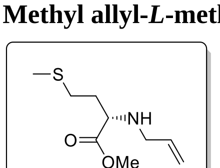

1o was prepared according to the **GP2** in 82% yield.
^{1}H NMR (400 MHz, CDCl$_3$) δ 5.90 – 5.78 (m, 1H), 5.22 – 5.05 (m, 2H), 3.74 (s, 3H), 3.40 (dd, *J* = 7.6, 5.7 Hz, 1H), 3.32 – 3.23 (m, 1H), 3.15 – 3.07 (m, 1H), 2.64 – 2.53 (m, 2H), 2.10 (s, 3H), 2.01 – 1.77 (m, 2H), 1.68 (s, 1H).
^{13}C NMR (101 MHz, CDCl$_3$) δ 175.6, 136.3, 116.4, 59.4, 51.8, 50.7, 32.9, 30.5, 15.4.
HRMS (ESI-TOF) m/z: [M+H]$^+$ Calcd. for $C_9H_{18}NO_2S$ 204.1053; found: 204.1055.

Diethyl 2-(allylamino)malonate (1p)

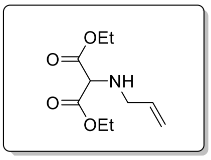

1p was prepared according to the **GP2** in 80% yield.

^{1}H NMR (400 MHz, CDCl$_3$) δ 5.93 – 5.79 (m, 1H), 5.28 – 5.08 (m, 2H), 4.32 – 4.15 (m, 4H), 4.06 (s, 1H), 3.27 (d, *J* = 6.1 Hz, 2H), 2.19 (s, 1H), 1.29 (t, *J* = 7.1 Hz, 6H).

^{13}C NMR (101 MHz, CDCl$_3$) δ 168.6, 135.4, 117.3, 64.0, 61.8, 50.3, 14.1.

HRMS (ESI-TOF) m/z: [M+H]$^+$ Calcd. for $C_{10}H_{18}NO_4$ 216.1230; found: 216.1234.

***tert*-Butyl allylglycinate (1q)**

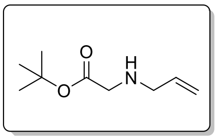

1q was prepared according to the **GP2** in 55% yield.

^{1}H NMR (400 MHz, CDCl$_3$) δ 5.96 – 5.77 (m, 1H), 5.23 – 5.06 (m, 2H), 3.30 (s, 2H), 3.26 (d, *J* = 6.0 Hz, 2H), 1.76 (s, 1H), 1.47 (s, 9H).

^{13}C NMR (101 MHz, CDCl$_3$) δ 171.7, 136.2, 116.5, 81.2, 51.8, 50.8, 28.1.

HRMS (ESI-TOF) m/z: [M+H]$^+$ Calcd. for $C_9H_{18}NO_2$ 172.1331; found: 172.1334.

Benzyl allylglycinate (1r)

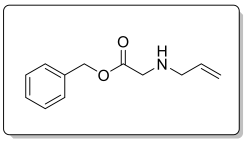

1r was prepared according to the **GP2** in 61% yield.

^{1}H NMR (400 MHz, CDCl$_3$) δ 7.40 – 7.30 (m, 5H), 5.92 – 5.79 (m, 1H), 5.25 – 5.00 (m, 4H), 3.45 (s, 2H), 3.26 (d, *J* = 6.0 Hz, 2H), 1.78 (s, 1H).

^{13}C NMR (101 MHz, CDCl$_3$) δ 172.4, 136.1, 135.6, 128.6, 128.4, 128.4, 116.6, 66.5, 51.8, 50.0.

HRMS (ESI-TOF) m/z: [M+H]$^+$ Calcd. for $C_{12}H_{16}NO_2$ 206.1176; found: 206.1180.

Methyl 2-(allylamino)-2-methylpropanoate (1s)

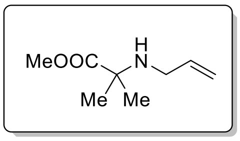

1s was prepared according to the **GP2** in 78% yield.

^{1}H NMR (400 MHz, CDCl$_3$) δ 5.98 – 5.82 (m, 1H), 5.25 – 5.03 (m, 2H), 3.71 (s, 3H), 3.18 – 3.07 (m, 2H), 1.84 (s, 1H), 1.33 (s, 6H).

^{13}C NMR (101 MHz, CDCl$_3$) δ 177.4, 136.6, 116.0, 58.9, 51.9, 47.3, 25.3.

HRMS (ESI-TOF) m/z: [M+H]$^+$ Calcd. for $C_8H_{16}NO_2$ 158.1176; found: 158.1176.

(*S*)-3-(Allylamino)dihydrofuran-2(3*H*)-one (1t)

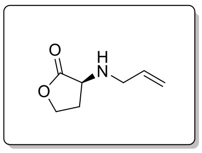

1t was prepared according to the **GP2** in 67% yield.

^{1}H NMR (400 MHz, CDCl$_3$) δ 5.94 – 5.82 (m, 1H), 5.29 – 5.10 (m, 2H), 4.41 (t, *J* = 8.9 Hz, 1H), 4.27 – 4.14 (m, 1H), 3.64 – 3.55 (m, 1H), 3.37 (d, *J* = 6.0 Hz, 2H), 2.58 – 2.45 (m, 1H), 2.18 – 2.02 (m, 1H), 1.93 (s, 1H).

^{13}C NMR (101 MHz, CDCl$_3$) δ 177.4, 135.8, 116.8, 65.7, 55.5, 50.5, 30.5.

HRMS (ESI-TOF) m/z: [M+H]$^+$ Calcd. for $C_7H_{12}NO_2$ 142.0863; found: 142.0870.

Methyl 3-(allylamino)-3-(4-fluorophenyl)propanoate (1u)

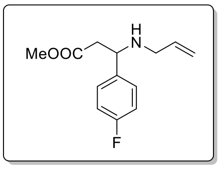

1u was prepared according to the **GP2** in 56% yield.

^{1}H NMR (400 MHz, CDCl$_3$) δ 7.30 (dd, *J* = 8.6, 5.5 Hz, 2H), 7.07 – 6.98 (m, 2H), 5.92 – 5.78 (m, 1H), 5.18 – 5.01 (m, 2H), 4.12 (dd, *J* = 8.3, 5.6 Hz, 1H), 3.64 (s, 3H), 3.13 – 2.96 (m, 2H), 2.70 (dd, *J* = 15.6, 8.4 Hz, 1H), 2.59 (dd, *J* = 15.6, 5.5 Hz, 1H), 1.81 (s, 1H).

^{13}C NMR (101 MHz, CDCl$_3$) δ 172.0, 162.1 (d, *J* = 245.2 Hz), 138.2 (d, *J* = 3.1 Hz), 136.5, 128.6 (d, *J* = 8.0 Hz), 116.0, 115.4 (d, *J* = 21.3 Hz), 58.0, 51.6, 49.7, 42.8.

^{19}F NMR (376 MHz, CDCl$_3$) δ -115.23.

HRMS (ESI-TOF) m/z: [M+H]$^+$ Calcd. for $C_{13}H_{17}FNO_2$ 238.1238; found: 238.1246.

***tert*-Butyl 3-(allylamino)propanoate (1v)**

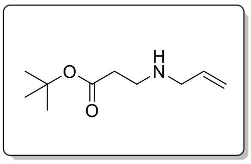

1v was prepared according to the **GP2** in 51% yield.

^{1}H NMR (400 MHz, CDCl$_3$) δ 5.96 – 5.82 (m, 1H), 5.24 – 5.04 (m, 2H), 3.31 – 3.20 (m, 2H), 2.84 (t, *J* = 6.5 Hz, 2H), 2.44 (t, *J* = 6.5 Hz, 2H), 1.81 (s, 1H), 1.45 (s, 9H).

^{13}C NMR (101 MHz, CDCl$_3$) δ 172.2, 136.7, 115.9, 80.5, 52.2, 44.6, 35.8, 28.1.

HRMS (ESI-TOF) m/z: [M+H]$^+$ Calcd. for $C_{10}H_{20}NO_2$ 186.1489; found: 186.1493.

Methyl 1-((2-methylallyl)amino)cyclohexane-1-carboxylate (1z)

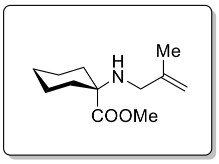

1z was prepared according to the **GP1** in 82% yield.

^{1}H NMR (400 MHz, CDCl$_3$) δ 4.89 (s, 1H), 4.79 (s, 1H), 3.70 (s, 3H), 2.95 (s, 2H), 1.96 – 1.82 (m, 2H),

1.79 – 1.64 (m, 5H), 1.61 – 1.52 (m, 2H), 1.49 – 1.35 (m, 5H).

^{13}C NMR (101 MHz, CDCl$_3$) δ 176.8, 144.5, 110.4, 61.6, 51.5, 49.3, 33.4, 25.8, 21.9, 21.1.

HRMS (ESI-TOF) m/z: [M+H]$^+$ Calcd. for $C_{12}H_{22}NO_2$ 212.1645; found: 212.1648.

Methyl 1-((3-methylbut-2-en-1-yl)amino)cyclohexane-1-carboxylate (1aa)

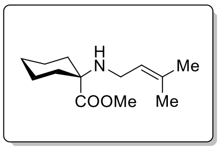

1aa was prepared according to the **GP1** in 59% yield.

^{1}H NMR (400 MHz, CDCl$_3$) δ 5.26 – 5.18 (m, 1H), 3.71 (s, 3H), 2.99 (d, *J* = 6.9 Hz, 2H), 1.99 – 1.88 (m, 2H), 1.75 – 1.63 (m, 5H), 1.61 (s, 3H), 1.57 – 1.48 (m, 2H), 1.46 – 1.35 (m, 5H).

^{13}C NMR (101 MHz, CDCl$_3$) δ 176.7, 134.6, 122.7, 61.9, 51.6, 41.1, 33.4, 25.7, 25.7, 22.1, 17.7.

HRMS (ESI-TOF) m/z: [M+H]$^+$ Calcd. for $C_{13}H_{24}NO_2$ 226.1802; found: 226.1804.

4. List of the starting materials

1a, **1k**, **1n**, **1z**, **1aa**, **1bb** were synthesized according to **GP1**[1]. **1b**-**1j**, **1l**, **1m**, **1o**-**1y** were synthesized according to **GP2**[2]. **1cc**-**1xx** were synthesized according to **GP3**[3] and the resultant data are matched with the literature.

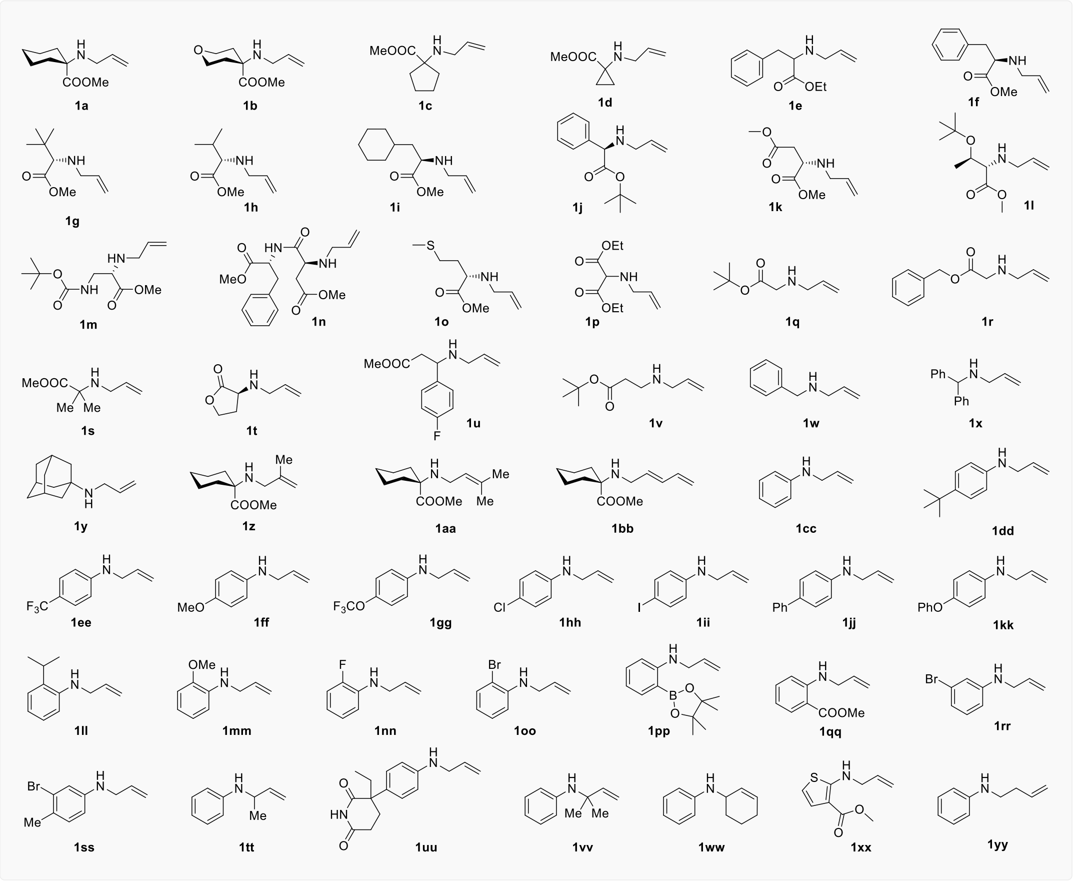

Supplementary Figure 1. List of the starting materials.

5. Scale-up and derivatization

5.1 Reaction scalep-up

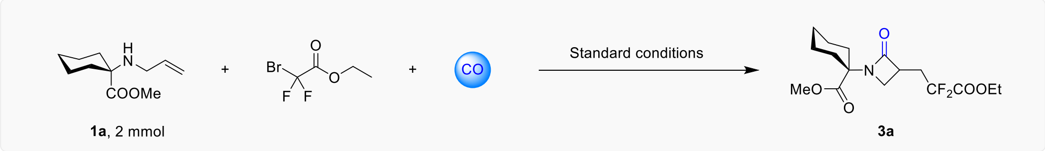

A 25 mL round-bottom flask was charged with **1a** (2 mmol), **2a** (4 mmol), photocatalyst (2 mol%), base (3 mmol), and solvent (15mL) under N_2 atmosphere. The flask was closed with a rubber stopper and connected to the atmosphere via a needle. The closed autoclave was flushed two times with N_2 (~ 5 bar), and a pressure of 40 bar CO were charged. The autoclave was then placed on a magnetic stirrer. The reaction mixture was stirred while being irradiated with 15 W blue light at room temperature for 24 h. After irradiation, the light was turned off and the pressure was released carefully. The mixture was concentrated under vacuum. The crude product was purified by column chromatography (PE/EA =10/1 to 5/1) on silica gel to afford the corresponding products **3a** (0.49 g, 71% yield).

5.2 One-pot allylation and carbonylative annulation to β-lactams **3p**

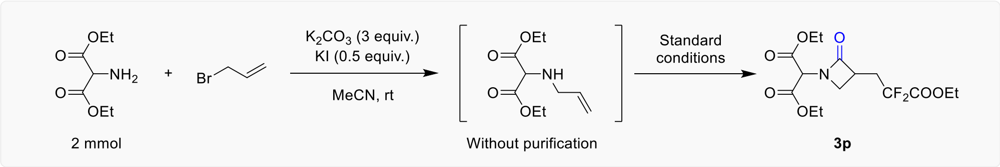

To a solution of amine (2 mmol, 1.0 equiv.) and potassium carbonate (6 mmol, 3.0 equiv.) in MeCN (15 mL), drops of allyl bromide (2 mmol, 1.0 equiv.) was added at room temperature and stirred overnight. The reaction mixture was was directly used for the following process without purification.

2a (2.0 equiv.), photocatalyst (2 mol%) was added into above mixture under N_2 atmosphere. The vial was then placed into autoclave. The closed autoclave was flushed two times with N_2 (~ 5 bar), and a pressure of 40 bar CO were charged. The autoclave was then placed on a magnetic stirrer. The reaction mixture was stirred while being irradiated with 15 W blue light at room temperature for 24 h. After irradiation, the light was turned off and the pressure was released carefully. The mixture was concentrated under vacuum. The crude product was purified by column chromatography (PE/EA = 5/1) on silica gel to afford the corresponding products **3p** (0.37g, 51%).

5.2 Transformations of compounds **3a**

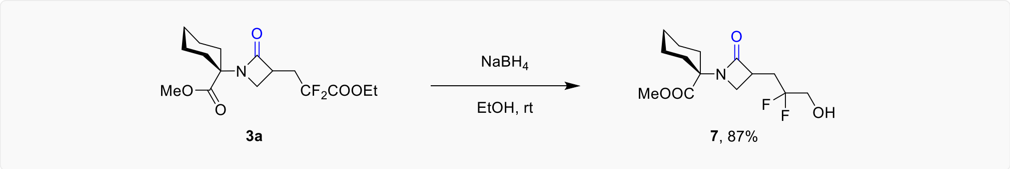

[4]To a solution of **3a** (0.3 mmol, 1 equiv) in ethanol (4 mL) at room temperature was added $NaBH_4$ (0.33 mmol, 12 mg, 1.1 equiv). The mixture was stirred for 0.5 h. After stirring for 0.5 h, the solution was diluted with ethyl acetate and washed with H_2O and brine. The organic layer was dried over Na_2SO_4, filtered, and concentrated. The residue was purified with flash column chromatography (PE/EA = 2/1) to give **7** as a colorless oil (79.4 mg, 87% yield).

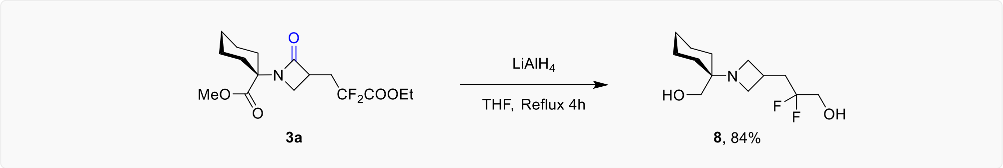

[5]To a stirred cold suspension (ice bath) of **3a** (0.3 mmol) in dry THF (1 mL) was added $LiAlH_4$ (0.6 mL, 1.0 mol/L in THF) slowly under N_2. The mixture was slowly warmed to the refluxing temperature for 4 h. The mixture was then cooled down to room temperature and quenched by addition of MeOH (1 mL) followed by addition of aq. solution of NaOH (10%, 1.0 mL). After the mixture was filtered through a pad of celite, THF was evaporated from the filtrate in vacuum. The resultant mixture was extracted with CH_2Cl_2 (3 × 5 mL). The combined organic layers were washed with brine (2 × 5 mL) and dried over $MgSO_4$. The solvent was removed in vacuum and the residue was purified by flash chromatography afford the desired product **8** (66.2 mg, 84%).

5.3 Transformations of compounds **4f**

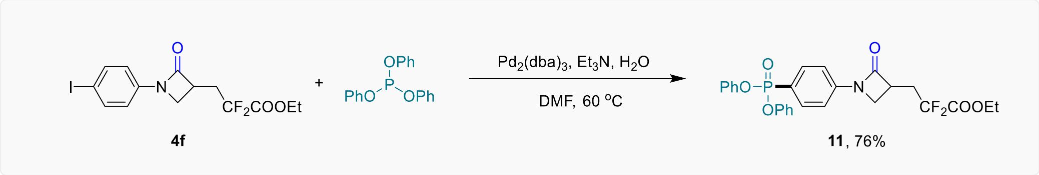

[6]Pure **4f** (0.2 mmol) and $Pd_2(dba)_3$ (4.6 mg, 5.0 mol%) were added into a 25 mL Schlenk tube. Then DMF (0.5 mL), H_2O (7.5 µL, 0.4 mmol), Et_3N (58 µL, 0.4 mmol) and triphenyl phosphite (56 µL, 0.2 mmol) were sequentially added under N_2 atmosphere. After the addition of all substances, the tube was heated at 60 °C for 2 h. Then 3 mL of water was added in the reaction mixture and extracted three times with ethyl acetate (3×4 mL). The organic layers were combined and washed with brine, dried over Na_2SO_4, and evaporated under vacuum. Pure **11** was obtained by flash chromatography (78.3 mg, 76%).

6. Control experiments

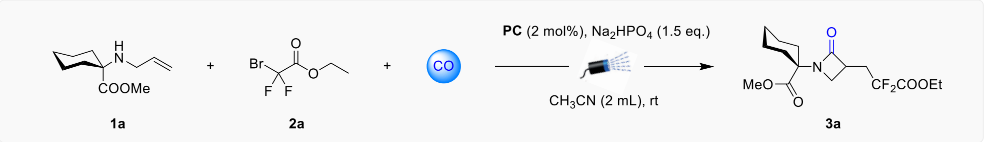

A 4 mL screw-cap vial was charged with **1a** (0.2 mmol), **2a** (0.4 mmol), photocatalyst (2 mol%), base (0.3 mmol), TEMPO (0.4 mmol) or 1,1-Diphenylethylene (0.4 mmol) and solvent (2 mL) under N_2 atmosphere. The vial was closed with a Teflon septum and cap and connected to the atmosphere via a needle. The closed autoclave was flushed two times with N_2 (~ 5 bar), and a pressure of 40 bar CO were charged. The autoclave was then placed on a magnetic stirrer. The reaction mixture was stirred while being irradiated with 15 W blue light at room temperature for 24 h. After irradiation, the light was turned off and the pressure was released carefully. After completion of the reaction, the solvent was concentrated under vacuum. After the reaction was completed, yields were determined by GC-FID analysis using n-hexadecane as internal standard.

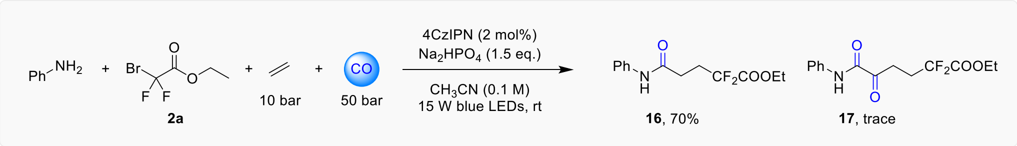

A 4 mL screw-cap vial was charged with aniline (0.2 mmol), **2a** (0.4 mmol), photocatalyst (2 mol%), base (0.3 mmol) and solvent (2 mL) under N_2 atmosphere. The vial was closed with a Teflon septum and cap and connected to the atmosphere via a needle. The closed autoclave was flushed two times with N_2 (~ 5 bar), and a pressure of 10 bar CO ethylene 50 bar CO were charged. The autoclave was then placed on a magnetic stirrer. The reaction mixture was stirred while being irradiated with 15 W blue light at room temperature for 24 h. After the reaction was completed, mixture was analyzed by GC-MS. Product **16** (38.1 mg, 70%) was afforded by flash chromatography.

7. Cyclic Voltammograms

The cyclic voltammetry was carried out with an Admiral Squidsta Plus - SUI v2.0 Beat workstation. All CV measurements were conducted in CH_3CN using Ag/AgCl as a reference and 1 mM nBu_4NBF_4 supporting electrolyte. The scan rate was 100 mV/s.

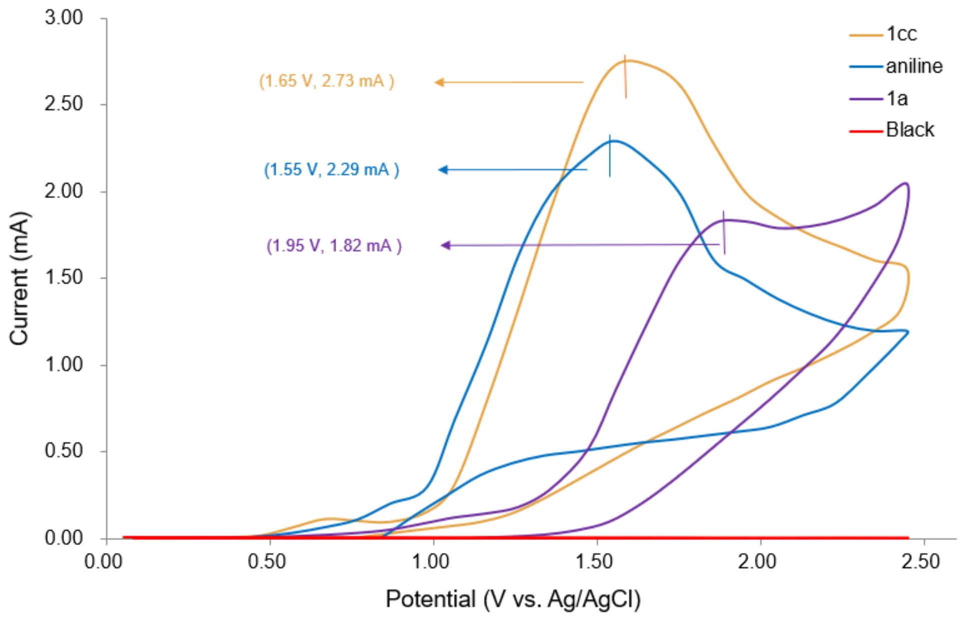

Supplementary Figure 2. Cyclic Voltammogram Curves.

8. Characterization of Products.

Methyl-1-(3-(3-ethoxy-2,2-difluoro-3-oxopropyl)-2-oxoazetidin-1-yl) cyclohexane-1-carboxylate (3a)

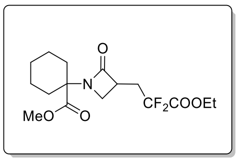

59.5 mg, colorless oil, yield: 86%. Eluent: pentane/ethyl acetate = 10/1.

^{1}H NMR (400 MHz, CDCl$_3$) δ 4.34 (q, *J* = 7.1 Hz, 2H), 3.73 (s, 1H), 3.49 (t, *J* = 5.5 Hz, 1H), 3.40 – 3.34 (m, 1H), 3.20 – 3.12 (m, 1H), 2.77 – 2.61 (m, 1H), 2.44 – 2.24 (m, 2H), 2.13 – 2.04 (m, 1H), 1.88 – 1.75 (m, 2H), 1.72 – 1.59 (m, 3H), 1.50 – 1.27 (m, 6H).

^{13}C NMR (101 MHz, CDCl$_3$) δ 173.5, 168.9, 163.6 (t, *J* = 32.4 Hz), 115.3 (t, *J* = 251.1 Hz), 63.3, 63.2, 52.6, 44.3, 41.4, 33.9 (t, *J* = 23.8 Hz), 31.2, 31.2, 24.7, 21.8, 21.7, 13.9.

^{19}F NMR (376 MHz, CDCl$_3$) δ -104.47 (d, *J* = 260.8 Hz, 1F), -106.94 (d, *J* = 260.8 Hz, 1F).

HRMS (ESI-TOF) m/z: $[M+H]^+$ Calcd. for $C_{16}H_{24}F_2NO_5$ 348.1617; found: 348.1620.

Methyl-4-(3-(3-ethoxy-2,2-difluoro-3-oxopropyl)-2-oxoazetidin-1-yl)tetrahydro-2H-pyran-4-carboxylate (3b)

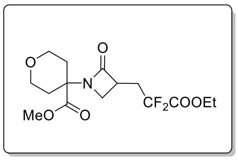

63.3 mg, colorless oil, yield: 91%. Eluent: pentane/ethyl acetate = 2/1.

^{1}H NMR (400 MHz, CDCl$_3$) δ 4.34 (q, *J* = 6.6 Hz, 2H), 3.90 – 3.81 (m, 2H), 3.81 – 3.76 (m, 3H), 3.60 (dd, *J* = 25.4, 11.9 Hz, 2H), 3.54 – 3.47 (m, 1H), 3.45 – 3.38 (m, 1H), 3.22 – 3.15 (m, 1H), 2.78 – 2.60 (m, 1H), 2.48 – 2.29 (m, 1H), 2.25 (d, *J* = 13.8 Hz, 1H), 2.22 – 2.09 (m, 2H), 2.04 (d, *J* = 14.0 Hz, 1H), 1.39 – 1.34 (m, 3H).

^{13}C NMR (101 MHz, CDCl$_3$) δ 172.1, 169.2, 163.5 (t, *J* = 32.3 Hz), 115.2 (t, *J* = 250.9 Hz), 63.5, 63.4, 63.2, 60.8, 52.9, 44.5, 41.6, 33.7 (t, *J* = 23.9 Hz), 31.1, 31.0, 13.9.

^{19}F NMR (376 MHz, CDCl$_3$) δ -104.5 (d, *J* = 261.6 Hz, 1F), -106.9 (d, *J* = 261.6 Hz, 1F).

HRMS (ESI-TOF) m/z: $[M+H]^+$ Calcd. for $C_{15}H_{22}F_2NO_6$ 350.1410; found: 350.1417.

Methyl-1-(3-(3-ethoxy-2,2-difluoro-3-oxopropyl)-2-oxoazetidin-1-yl)cyclopentane-1-carboxylate (3c)

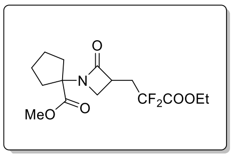

47.2 mg, colorless oil, yield: 71%. Eluent: pentane/ethyl acetate = 10/1.

^{1}H NMR (400 MHz, CDCl$_3$) δ 4.38 – 4.28 (m, 2H), 3.74 (s, 3H), 3.52 – 3.43 (m, 1H), 3.37 – 3.29 (m, 1H), 3.18 – 3.11 (m, 1H), 2.75 – 2.59 (m, 1H), 2.45 – 2.21 (m, 3H), 2.14 – 1.98 (m, 2H), 1.89 – 1.68 (m, 4H), 1.36 (td, *J* = 7.1, 2.6 Hz, 3H).

^{13}C NMR (101 MHz, CDCl$_3$) δ 173.5, 168.6, 163.6 (t, *J* = 32.6 Hz), 115.3 (t, *J* = 250.8 Hz),69.0, 63.2, 52.7, 44.9, 41.2 (t, *J* = 3.3 Hz), 35.4, 35.2, 33.8 (t, *J* = 23.9 Hz), 24.1, 24.1, 13.9.
^{19}F NMR (376 MHz, CDCl$_3$) δ -104.5 (d, *J* = 260.8 Hz, 1F), -107.0 (d, *J* = 260.7 Hz, 1F).
HRMS (ESI-TOF) m/z: $[M+H]^+$ Calcd. for $C_{15}H_{22}F_2N_2O_6$ 334.1461; found: 334.1468.

Methyl-1-(3-(3-ethoxy-2,2-difluoro-3-oxopropyl)-2-oxoazetidin-1-yl)cyclopropane-1-carboxylate (3d)

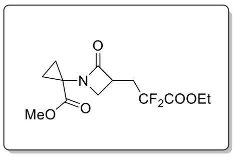

39.5 mg, colorless oil, yield: 65%. Eluent: pentane/ethyl acetate = 10/1.
^{1}H NMR (400 MHz, CDCl$_3$) δ 4.39 – 4.30 (m, 2H), 3.73 (s, 3H), 3.54 (t, *J* = 5.5 Hz, 1H), 3.42 – 3.35 (m, 1H), 3.27 – 3.22 (m, 1H), 2.75 – 2.58 (m, 1H), 2.45 – 2.27 (m, 1H), 1.58 – 1.47 (m, 2H), 1.43 – 1.29 (m, 5H).
^{13}C NMR (101 MHz, CDCl$_3$) δ 171.7, 168.7, 163.5 (t, *J* = 32.4 Hz), 115.2 (t, *J* = 250.9 Hz), 63.2, 52.7, 46.6, 42.7, 34.8, 33.6 (t, *J* = 24.0 Hz), 16.8, 16.3, 13.9.
^{19}F NMR (376 MHz, CDCl$_3$) δ -104.6 (d, *J* = 261.0 Hz, 1F), -106.9 (d, *J* = 261.0 Hz, 1F).
HRMS (ESI-TOF) m/z: $[M+H]^+$ Calcd. for $C_{13}H_{18}F_2NO_5$ 306.1148; found: 306.1151.

Ethyl-3-(1-(1-ethoxy-1-oxo-3-phenylpropan-2-yl)-2-oxoazetidin-3-yl)-2,2-difluoropropanoate (3e)

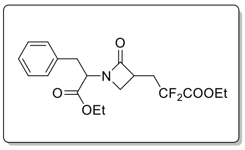

53.8 mg, colorless oil, yield: 70%. Eluent: pentane/ethyl acetate = 10/1.
Inseparable mixture of diastereomers. dr = 1:1.
^{1}H NMR (400 MHz, CDCl$_3$) δ 7.35 – 7.29 (m, 2H), 7.26 (t, *J* = 3.1 Hz, 1H), 7.22 (dd, *J* = 13.0, 4.9 Hz, 2H), 4.73 – 4.61 (m, 1H), 4.36 – 4.27 (m, 2H), 4.25 – 4.17 (m, 2H), 3.61 (t, *J* = 5.5 Hz, 0.5H), 3.44 (t, *J* = 5.4 Hz, 0.5H), 3.34 – 3.22 (m, 2.5H), 3.08 – 2.96 (m, 1.5H), 2.68 – 2.52 (m, 0.5H), 2.50 – 2.19 (m, 1H), 1.88 – 1.70 (m, 0.5H), 1.34 (t, *J* = 7.1 Hz, 3H), 1.30 – 1.23 (m, 3H).
^{13}C NMR (101 MHz, CDCl$_3$) δ 169.9, 169.8, 168.9, 168.0, 163.6 (t, *J* = 32.5 Hz), 136.2, 136.0, 128.8, 128.8, 128.7, 128.6, 127.23, 127.21, 115.1 (t, *J* = 249.3 Hz), 63.2, 63.1, 61.7, 61.7, 55.1, 54.7, 45.3, 45.1, 42.8, 42.6, 35.9, 35.6, 33.7 (t, *J* = 23.9 Hz), 14.1, 13.9.
^{19}F NMR (376 MHz, CDCl$_3$) δ -104.2 – -107.4 (m, 2F).
HRMS (ESI-TOF) m/z: $[M+H]^+$ Calcd. for $C_{19}H_{24}F_2NO_5$ 384.1617; found: 384.1620.

Ethyl-2,2-difluoro-3-(1-((*R*)-1-methoxy-1-oxo-3-phenylpropan-2-yl)-2-oxoazetidin-3-yl)propanoate (3f)

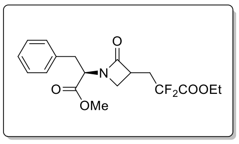

49.9 mg, colorless oil, yield: 68%. Eluent: pentane/ethyl acetate = 5/1.

Inseparable mixture of diastereomers. dr = 1:1.

^{1}H NMR (400 MHz, CDCl$_3$) δ 7.39 – 7.15 (m, 5H), 4.77 – 4.62 (m, 1H), 4.40 – 4.24 (m, 2H), 3.76 (s, 3H), 3.59 (t, *J* = 5.5 Hz, 0.5H), 3.44 (t, *J* = 5.7 Hz, 0.5H), 3.35 – 3.21 (m, 2.5H), 3.10 – 2.96 (m, 1.5H), 2.68 – 2.52 (m, 0.5H), 2.49 – 2.19 (m, 1H), 1.87 – 1.74 (m, 0.5H), 1.34 (t, *J* = 7.1 Hz, 3H).

^{13}C NMR (101 MHz, CDCl$_3$) δ 170.4, 170.3, 168.2, 168.0, 163.5 (t, *J* = 32.4 Hz), 136.1, 136.0, 128.8, 128.8, 128.6, 127.3, 127.2, 115.1 (t, *J* = 250.9 Hz), 63.2, 63.1, 55.1, 54.6, 52.6, 52.5, 45.3, 45.1, 42.8, 42.6, 35.8, 35.6, 33.7 (t, *J* = 23.4 Hz), 13.9.

^{19}F NMR (376 MHz, CDCl$_3$) δ -104.2 – -107.4 (m, 2F).

HRMS (ESI-TOF) m/z: [M+H]$^+$ Calcd. for $C_{18}H_{22}F_2NO_5$ 370.1461; found: 370.1466.

Methyl-(2*S*)-2-(3-(3-ethoxy-2,2-difluoro-3-oxopropyl)-2-oxoazetidin-1-yl)-3,3-dimethylbutanoate (3g)

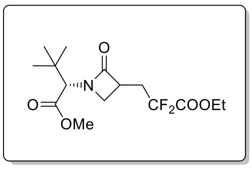

55.2 mg, colorless oil, yield: 82%. Eluent: pentane/ethyl acetate = 5/1.

Inseparable mixture of diastereomers. dr = 1.5:1.

^{1}H NMR (400 MHz, CDCl$_3$) δ 4.34 (q, *J* = 7.1 Hz, 2H), 4.28 (s, 1H), 4.01 (t, *J* = 5.9 Hz, 0.4H), 3.80 – 3.71 (m, 3.6H), 3.69 – 3.63 (m, 0.6H), 3.53 – 3.39 (m, 1.4H), 2.82 – 2.56 (m, 1H), 2.42 – 2.23 (m, 1H), 1.36 (t, *J* = 7.1 Hz, 3H), 1.04 (s, 9H).

^{13}C NMR (101 MHz, CDCl$_3$) δ 169.8, 169.6, 168.7, 168.4, 163.6 (t, *J* = 32.4 Hz), 115.1 (t, *J* = 250.9 Hz), 63.2, 63.2, 61.9, 61.7, 51.8, 51.7, 48.5, 48.3, 43.3, 43.1, 35.4, 35.4, 34.1 (t, *J* = 24.0 Hz), 33.6 (t, *J* = 23.9 Hz)27.4, 27.4, 13.9.

^{19}F NMR (376 MHz, CDCl$_3$) δ -104.1 – -107.3 (m, 2F).

HRMS (ESI-TOF) m/z: [M+H]$^+$ Calcd. for $C_{18}H_{22}F_2NO_5$ 336.1676; found: 336.1672.

Methyl-(2*S*)-2-(3-(3-ethoxy-2,2-difluoro-3-oxopropyl)-2-oxoazetidin-1-yl)-3-methylbutanoate (3h)

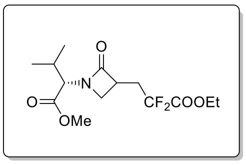

42.6 mg, colorless oil, yield: 66%. Eluent: pentane/ethyl acetate = 5/1.

Inseparable mixture of diastereomers. dr = 1:1.

^{1}H NMR (400 MHz, CDCl$_3$) δ 4.34 (q, *J* = 7.1 Hz, 2H), 4.18 (t, *J* = 7.8 Hz, 1H), 3.83 (t, *J* = 5.7 Hz, 0.5H), 3.77 – 3.72 (m, 3H), 3.62 (t, *J* = 5.5 Hz, 0.5H), 3.53 – 3.43 (m, 1.5H), 3.26 (dd, *J* = 6.0, 1.7 Hz, 0.5H), 2.79 – 2.56 (m, 1H), 2.43 – 2.25 (m, 1H), 2.24 – 2.13 (m, 1H), 1.36 (t, *J* = 7.1 Hz, 3H), 1.01 – 0.95 (m, 6H).

^{13}C NMR (101 MHz, CDCl$_3$) δ 170.5, 170.3, 168.5, 168.2, 163.6 (t, *J* = 32.4 Hz), 115.1 (t, *J* = 250.9 Hz), 63.2, 63.2, 59.5, 52.1, 52.0, 46.3, 46.2, 43.0 (t, *J* = 7.1 Hz), 34.1 (t, *J* = 24.0 Hz), 33.7 (t, *J* = 24.0 Hz), 29.4, 19.2, 19.2, 19.2, 13.9.

^{19}F NMR (376 MHz, CDCl$_3$) δ -104.1 – -107.3 (m, 2F).

HRMS (ESI-TOF) m/z: [M+H]$^+$ Calcd. for $C_{14}H_{22}F_2NO_5$ 322.1461; found: 322.1465.

Ethyl-3-(1-((R)-3-cyclohexyl-1-methoxy-1-oxopropan-2-yl)-2-oxoazetidin-3-yl)-2,2-difluoropropanoate (3i)

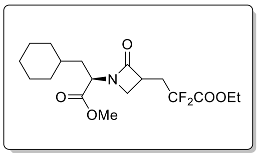

54.5 mg, colorless oil, yield: 72%. Eluent: pentane/ethyl acetate = 5/1.

Inseparable mixture of diastereomers. dr = 1:1.

^{1}H NMR (400 MHz, CDCl$_3$) δ 4.53 – 4.42 (m, 1H), 4.35 (q, *J* = 6.9 Hz, 2H), 3.77 – 3.67 (m, 3.5H), 3.51 (t, *J* = 5.3 Hz, 0.5H), 3.48 – 3.35 (m,1.5H), 3.16 (d, *J* = 5.7 Hz, 0.5H), 2.78 – 2.61 (m, 1H), 2.47 – 2.19 (m, 1H), 1.81 (d, *J* = 12.6 Hz, 1H), 1.76 – 1.60 (m, 6H), 1.37 (t, *J* = 7.1 Hz, 3H), 1.29 – 1.15 (m, 4H), 1.06 – 0.85 (m, 2H).

^{13}C NMR (101 MHz, CDCl$_3$) δ 171.5, 171.4, 168.3, 168.0, 163.6 (t, *J* = 32.4 Hz), 115.2 (t, *J* = 250.8 Hz), 63.21, 63.16, 52.3, 51.5, 51.3, 44.7, 44.5, 42.6, 42.5, 36.7, 36.5, 34.5, 34.4, 33.9 (t, *J* = 24.0 Hz), 33.7 (t, *J* = 23.9 Hz), 33.5, 33.4, 31.9, 31.8, 26.3, 26.1, 25.9, 25.8, 13.9.

^{19}F NMR (376 MHz, CDCl$_3$) δ -104.1 – -107.3 (m, 2F).

HRMS (ESI-TOF) m/z: $[M+H]^+$ Calcd. for $C_{14}H_{22}F_2NO_5$ 376.1930; found: 376.1937.

Ethyl-3-(1-((*R*)-2-(tert-butoxy)-2-oxo-1-phenylethyl)-2-oxoazetidin-3-yl)-2,2-difluoropropanoate (3j)

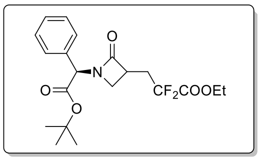

48.6 mg, colorless oil, yield: 61%. Eluent: pentane/ethyl acetate = 5/1.

Inseparable mixture of diastereomers. dr = 1:1.

^{1}H NMR (400 MHz, CDCl$_3$) δ 7.46 – 7.32 (m, 3H), 7.30 – 7.23 (m, 2H), 5.47 (s, 1H), 4.36 – 4.24 (m, 2H), 3.85 (t, *J* = 5.6 Hz, 0.5H), 3.55 – 3.41 (m, 1H), 3.35 – 3.23 (m, 1H), 2.90 (dd, *J* = 5.9, 2.2 Hz, 0.5H), 2.80 – 2.52 (m, 1H), 2.50 – 2.34 (m, 0.5H), 2.24 – 2.06 (m, 0.5H), 1.45 (s, 9H), 1.38 – 1.30 (m, 3H).

^{13}C NMR (101 MHz, CDCl$_3$) δ 168.44, 168.40, 167.6, 167.5, 163.6 (t, *J* = 32.4 Hz),133.8, 133.7, 129.12, 129.07, 128.7, 128.6, 128.04, 127.98, 115.2 (t, *J* = 250.9 Hz), 82.9, 82.8, 63.2, 58.2, 58.1, 45.3, 45.2, 42.5, 42.5, 33.8 (t, *J* = 24.0 Hz), 33.6 (t, *J* = 23.9 Hz), 27.93, 27.92, 13.91, 13.88.

^{19}F NMR (376 MHz, CDCl$_3$) δ -104.3 – -107.3 (m, 2F).

HRMS (ESI-TOF) m/z: $[M+H]^+$ Calcd. for $C_{20}H_{26}F_2NO_5$ 398.1774; found: 398.1772.

Dimethyl-(2S)-2-(3-(3-ethoxy-2,2-difluoro-3-oxopropyl)-2-oxoazetidin-1-yl)succinate (3k)

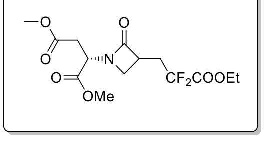

50.1 mg, colorless oil, yield: 71%. Eluent: pentane/ethyl acetate = 5/1.

Inseparable mixture of diastereomers. dr = 1:1.

^{1}H NMR (400 MHz, CDCl$_3$) δ 4.77 – 4.67 (m, 1H), 4.34 (q, *J* = 7.1 Hz, 2H), 3.78 (s, 3H), 3.72 (s, 3H), 3.69 (t, *J* = 5.6 Hz, 0.5H), 3.55 (t, *J* = 5.5 Hz, 0.5H), 3.51 – 3.39 (m, 1H), 3.37 (d, *J* = 5.7 Hz, 0.5H), 3.25 – 3.19 (m, 0.5H), 3.00 – 2.84 (m, 2H), 2.74 – 2.58 (m, 1H), 2.45 – 2.24 (m, 1H), 1.36 (t, *J* = 7.1 Hz, 3H).
^{13}C NMR (101 MHz, CDCl$_3$) δ 170.4, 169.3, 169.24, 168.21, 168.0, 163.5 (t, *J* = 32.3 Hz), 115.1 (t, *J* = 251.0 Hz), 63.2, 52.9, 52.3, 52.2, 50.8, 50.6, 46.0, 46.0, 43.1, 34.6, 34.5, 33.6 (t, *J* = 23.9 Hz), 13.91.
^{19}F NMR (376 MHz, CDCl$_3$) δ -104.2 – -107.3 (m, 2F).
HRMS (ESI-TOF) m/z: [M+H]$^+$ Calcd. for $C_{14}H_{20}F_2NO_7$ 352.1202; found: 352.1206.

Methyl-(2*S*,3*R*)-3-(tert-butoxy)-2-(3-(3-ethoxy-2,2-difluoro-3-oxopropyl)-2-oxoazetidin-1-yl)butanoate (3l)

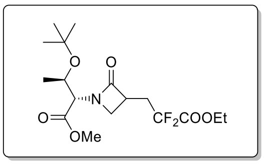

68.4 mg, colorless oil, yield: 90%. Eluent: pentane/ethyl acetate = 3/1.
Inseparable mixture of diastereomers. dr = 1:1.
^{1}H NMR (400 MHz, CDCl$_3$) δ 4.39 – 4.26 (m, 4H), 4.00 (t, *J* = 5.9 Hz, 0.4H), 3.81 – 3.75 (m, 0.4H), 3.72 (s, 3H), 3.67 – 3.62 (m, 0.6H), 3.51 – 3.41 (m, 1.4H), 2.79 – 2.59 (m, 1H), 2.50 – 2.26 (m, 1H), 1.42 – 1.33 (m, 3H), 1.20 (s, 3H), 1.11 (s, 9H).
^{13}C NMR (101 MHz, CDCl$_3$) δ 169.8, 169.6, 169.4, 163.7 (t, *J* = 32.5 Hz), 115.3 (t, *J* = 250.7 Hz), 74.1, 74.1, 68.24, 68.16, 63.2, 63.1, 59.0, 58.9, 52.1, 48.6, 48.5, 43.6, 43.5, 34.3 (t, *J* = 24.0 Hz), 33.9 (t, *J* = 24.0 Hz), 28.4, 21.2, 13.9.
^{19}F NMR (376 MHz, CDCl$_3$) δ -104.5 – -107.2 (m, 2F).
HRMS (ESI-TOF) m/z: [M+H]$^+$ Calcd. for $C_{17}H_{28}F_2NO_6$ 380.1879; found: 380.1885.

Ethyl-3-(1-((*S*)-3-((tert-butoxycarbonyl)amino)-1-methoxy-1-oxopropan-2-yl)-2-oxoazetidin-3-yl)-2,2-difluoropropanoate (3m)

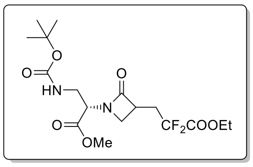

62.8 mg, colorless oil, yield: 77%. Eluent: pentane/ethyl acetate = 3/1.
Inseparable mixture of diastereomers. dr = 1:1.
^{1}H NMR (400 MHz, CDCl$_3$) δ 5.24 (d, *J* = 29.8 Hz, 1H), 4.42 – 4.29 (m, 3H), 3.77 (s, 3H), 3.72 – 3.55 (m, 3H), 3.45 (s, 1H), 3.33 (dd, *J* = 19.9, 4.7 Hz, 1H), 2.74 – 2.56 (m, 1H), 2.51 – 2.28 (m, 1H), 1.43 (s, 9H), 1.36 (t, *J* = 7.1 Hz, 3H).
^{13}C NMR (101 MHz, CDCl$_3$) δ 168.85, 168.83, 168.64, 168.58, 163.5 (t, *J* = 32.4 Hz), 155.8, 115.1 (t, *J* = 251.0 Hz), 79.8, 63.2, 63.2, 55.9, 55.6, 52.7, 52.7, 45.9, 45.8, 43.1, 40.1, 39.9, 33.6 (t, *J* = 24.0 Hz), 33.5 (t, *J* = 23.9 Hz), 28.3, 28.2, 13.9.
^{19}F NMR (376 MHz, CDCl$_3$) δ -104.3 – -107.2 (m, 2F).
HRMS (ESI-TOF) m/z: [M+Na]$^+$ Calcd. for $C_{17}H_{26}F_2N_2O_7Na$ 431.1600; found: 431.1607.

Methyl-(3*S*)-3-(3-(3-ethoxy-2,2-difluoro-3-oxopropyl)-2-oxoazetidin-1-yl)-4-(((*R*)-1-methoxy-1-oxo-3-phenylpropan-2-yl)amino)-4-oxobutanoate (3n)

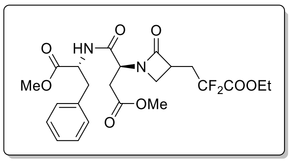

72.1 mg, colorless oil, yield: 72%. Eluent: pentane/ethyl acetate = 3/1.

Inseparable mixture of diastereomers. dr = 1:1.

^{1}H NMR (400 MHz, CDCl$_3$) δ 7.33 – 7.24 (m, 3H), 7.21 – 7.12 (m, 2H), 6.79 (dd, *J* = 16.9, 6.7 Hz, 1H), 4.87 – 4.77 (m, 1H), 4.61 (dd, *J* = 7.7, 6.6 Hz, 1H), 4.34 (q, *J* = 7.2 Hz, 2H), 3.75 (s, 3H), 3.67 (d, *J* = 3.9 Hz, 3H), 3.45 (t, *J* = 5.6 Hz, 0.5H), 3.40 – 3.33 (m, 0.5H), 3.30 – 3.17 (m, 0.5H), 3.12 – 2.95 (m, 2.5H), 2.94 – 2.83 (m, 1H), 2.81 – 2.67 (m, 1H), 2.66 – 2.47 (m, 1H), 2.33 – 2.06 (m, 1H), 1.37 (t, *J* = 7.1 Hz, 3H).

^{13}C NMR (101 MHz, CDCl$_3$) δ 171.5, 170.7, 170.6, 168.22, 168.21, 168.1, 167.8, 163.5 (t, J = 32.4 Hz), 135.8, 135.7, 129.18, 129.16, 128.70, 128.66, 127.3, 115.0 (t, J = 251.4 Hz), 63.3, 53.4, 53.2, 52.54, 52.52, 52.3, 52.21, 52.17, 45.5, 45.1, 42.9, 37.6, 37.5, 33.6, 33.4 (t, J = 23.9 Hz), 13.9.

^{19}F NMR (376 MHz, CDCl$_3$) δ -104.1 – -107.0 (m, 2F).

HRMS (ESI-TOF) m/z: [M+H]$^+$ Calcd. for $C_{23}H_{29}F_2N_2O_8$ 499.1886; found: 499.1892.

Methyl-(2*S*)-2-(3-(3-ethoxy-2,2-difluoro-3-oxopropyl)-2-oxoazetidin-1-yl)-4-(methylthio)butanoate (3o)

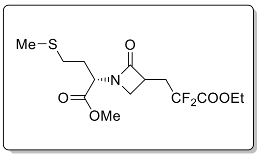

43.4 mg, colorless oil, yield: 61%. Eluent: pentane/ethyl acetate = 10/1.

Inseparable mixture of diastereomers. dr = 1:1.

^{1}H NMR (400 MHz, CDCl$_3$) δ 4.59 – 4.45 (m, 1H), 4.34 (q, *J* = 7.1 Hz, 2H), 3.76 (s, 3H), 3.68 (t, *J* = 5.5 Hz, 0.5H), 3.54 (t, *J* = 5.4 Hz, 0.5H), 3.51 – 3.41 (m, 1H), 3.37 (d, *J* = 5.5 Hz, 0.5H), 3.21 (d, *J* = 4.2 Hz, 0.5H), 2.77 – 2.45 (m, 3H), 2.44 – 2.29 (m, 1H), 2.24 – 2.15 (m, 1H), 2.11 (s, 3H), 2.10 – 1.93 (m, 1H), 1.37 (t, *J* = 7.1 Hz, 3H).

^{13}C NMR (101 MHz, CDCl$_3$) δ 170.6, 170.5, 168.5, 168.3, 163.5 (t, J = 32.3 Hz), 115.1 (t, J = 251.0 Hz), 63.3, 63.2, 53.4, 53.0, 52.6, 45.2, 45.0, 42.9, 42.8, 33.8 (t, J = 23.9 Hz), 33.7 (t, J = 23.9 Hz), 30.7, 29.0, 28.8, 15.50, 15.46, 13.9.

^{19}F NMR (376 MHz, CDCl$_3$) δ -104.1 – -107.3 (m, 2F).

HRMS (ESI-TOF) m/z: [M+H]$^+$ Calcd. for $C_{14}H_{22}F_2NO_5S$ 354.1181; found: 354.1173.

Diethyl 2-(3-(3-ethoxy-2,2-difluoro-3-oxopropyl)-2-oxoazetidin-1-yl)malonate (3p)

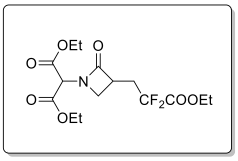

59.7 mg, colorless oil, yield: 82%. Eluent: pentane/ethyl acetate = 5/1.

^{1}H NMR (400 MHz, CDCl$_3$) δ 5.18 (s, 1H), 4.41 – 4.20 (m, 6H), 3.82 (t, *J* = 5.8 Hz, 1H), 3.57 – 3.47 (m, 2H), 2.78 – 2.60 (m, 1H), 2.50 – 2.30 (m, 1H), 1.40 – 1.28 (m, 9H).
^{13}C NMR (101 MHz, CDCl$_3$) δ 168.1, 164.9, 164.7, 163.5 (t, J = 32.3 Hz), 115.0 (t, J = 251.1 Hz), 63.2, 62.6, 62.6, 56.7, 46.4, 43.7 (t, J = 3.4 Hz), 33.7 (t, J = 24.0 Hz), 14.0, 13.9.
^{19}F NMR (376 MHz, CDCl$_3$) δ -104.7 (d, J = 261.9 Hz, 1F), -106.8 (d, J = 261.7 Hz, 1F).
HRMS (ESI-TOF) m/z: $[M+H]^+$ Calcd. for $C_{15}H_{22}F_2NO_7$ 366.1359; found: 366.1369.

Mthyl-3-(1-(2-(tert-butoxy)-2-oxoethyl)-2-oxoazetidin-3-yl)-2,2-difluoropropanoate (3q)

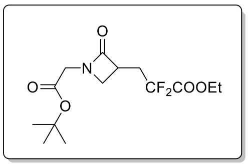

38.6 mg, colorless oil, yield: 60%. Eluent: pentane/ethyl acetate = 5/1.
^{1}H NMR (400 MHz, CDCl$_3$) δ 4.41 – 4.23 (m, 2H), 3.94 (dd, *J* = 17.9, 3.1 Hz, 1H), 3.80 (dd, *J* = 17.9, 3.0 Hz, 1H), 3.65 – 3.56 (m, 1H), 3.54 – 3.43 (m, 1H), 3.31 (s, 1H), 2.78 – 2.60 (m, 1H), 2.51 – 2.28 (m, 1H), 1.47 (d, *J* = 3.0 Hz, 9H), 1.39 – 1.32 (m, 3H).
^{13}C NMR (101 MHz, CDCl$_3$) δ 168.2, 167.0, 163.5 (t, J = 32.2 Hz), 115.1 (t, J = 250.8 Hz), 82.6, 63.2, 46.9, 43.9, 43.7, 33.8 (t, J = 24.0 Hz), 28.0, 13.9.
^{19}F NMR (376 MHz, CDCl$_3$) δ -104.7 (d, J = 261.1 Hz, 1F), -106.9 (d, J = 260.9 Hz, 1F).
HRMS (ESI-TOF) m/z: $[M+H]^+$ Calcd. for $C_{14}H_{22}F_2NO_5$ 322.1461; found: 322.1462.

Mthyl-3-(1-(2-(benzyloxy)-2-oxoethyl)-2-oxoazetidin-3-yl)-2,2-difluoropropanoate (3r)

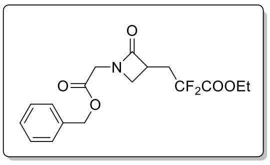

44.3 mg, colorless oil, yield: 62%. Eluent: pentane/ethyl acetate = 5/1.
^{1}H NMR (400 MHz, CDCl$_3$) δ 7.42 – 7.30 (m, 5H), 5.17 (s, 2H), 4.33 (q, *J* = 7.1 Hz, 2H), 4.09 (d, *J* = 18.1 Hz, 1H), 3.95 (d, *J* = 18.1 Hz, 1H), 3.61 (t, *J* = 5.4 Hz, 1H), 3.54 – 3.44 (m, 1H), 3.30 (dd, *J* = 5.5, 2.2 Hz, 1H), 2.76 – 2.57 (m, 1H), 2.43 – 2.24 (m, 1H), 1.35 (t, *J* = 7.1 Hz, 3H)..
^{13}C NMR (101 MHz, CDCl$_3$) δ 168.3, 167.8, 163.5 (t, *J* = 32.4 Hz), 134.9, 128.7, 128.7, 128.5, 115.1 (t, *J* = 250.9 Hz), 67.4, 63.2, 47.0, 44.1 (t, *J* = 3.4 Hz), 43.0, 33.7 (t, *J* = 24.0 Hz), 13.9.
^{19}F NMR (376 MHz, CDCl$_3$) δ -104.6 (d, J = 261.3 Hz, 1F), -106.9 (d, J = 261.5 Hz, 1F).
HRMS (ESI-TOF) m/z: $[M+H]^+$ Calcd. for $C_{17}H_{20}F_2NO_5$ 356.1304; found: 356.1295.

Ethyl-2,2-difluoro-3-(1-(1-methoxy-2-methyl-1-oxopropan-2-yl)-2-oxoazetidin-3-yl)propanoate (3s)

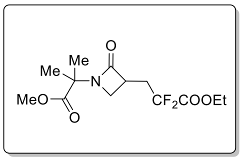

42.4 mg, colorless oil, yield: 69%. Eluent: pentane/ethyl acetate = 5/1.

^{1}H NMR (400 MHz, CDCl$_3$) δ 4.34 (q, *J* = 7.1 Hz, 2H), 3.75 (s, 3H), 3.52 (t, *J* = 5.5 Hz, 1H), 3.38 – 3.29 (m, 1H), 3.19 (dd, *J* = 5.4, 1.9 Hz, 1H), 2.76 – 2.53 (m, 1H), 2.45 – 2.25 (m, 1H), 1.60 (s, 3H), 1.53 (s, 3H), 1.36 (t, *J* = 7.1 Hz, 3H).
^{13}C NMR (101 MHz, CDCl$_3$) δ 173.5, 167.9, 163.6 (t, *J* = 32.5 Hz), 115.3 (t, *J* = 251.2 Hz), 63.2, 59.2, 52.7, 44.6, 41.4 (t, *J* = 3.3 Hz), 33.7 (t, *J* = 23.9 Hz), 24.1, 24.0, 13.9.
^{19}F NMR (376 MHz, CDCl$_3$) δ -104.5 (d, J = 260.7 Hz, 1F), -107.0 (d, J = 260.7 Hz, 1F).
HRMS (ESI-TOF) m/z: $[M+H]^+$ Calcd. for $C_{13}H_{20}F_2NO_5$ 308.1304; found: 308.1297.

Ethyl-2,2-difluoro-3-(2-oxo-1-((*S*)-2-oxotetrahydrofuran-3-yl)azetidin-3-yl)propanoate (3t)

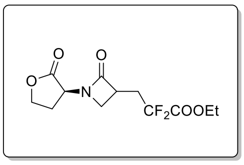

40.5 mg, colorless oil, yield: 70%. Eluent: pentane/ethyl acetate = 1/1.
Inseparable mixture of diastereomers. dr = 1:1.
^{1}H NMR (400 MHz, CDCl$_3$) δ 4.67 – 4.51 (m, 1H), 4.51 – 4.43 (m, 1H), 4.38 – 4.23 (m, 3H), 3.66 – 3.44 (m, 2H), 3.31 – 3.22 (m, 1H), 2.76 – 2.51 (m, 2H), 2.50 – 2.28 (m, 2H), 1.36 (t, *J* = 7.1 Hz, 3H).
^{13}C NMR (101 MHz, CDCl$_3$) δ 172.4, 172.2, 168.4, 168.3, 163.5 (t, *J* = 32.3 Hz), 115.0 (t, *J* = 251.1 Hz), 65.6, 65.6, 63.30, 63.28, 50.5, 50.2, 44.6, 44.4, 43.4, 33.7 (t, *J* = 24.0 Hz), 33.6 (t, *J* = 24.0 Hz), 26.9, 26.8, 13.9.
^{19}F NMR (376 MHz, CDCl$_3$) δ -104.1 – -107.2 (m, 2F).
HRMS (ESI-TOF) m/z: $[M+H]^+$ Calcd. for $C_{12}H_{16}F_2NO_5$ 292.0991; found: 292.0989.

Ethyl-2,2-difluoro-3-(1-(1-(4-fluorophenyl)-3-methoxy-3-oxopropyl)-2-oxoazetidin-3-yl)propanoate (3u)

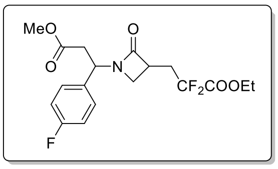

38.5 mg, colorless oil, yield: 50%. Eluent: pentane/ethyl acetate = 5/1.
Inseparable mixture of diastereomers. dr = 1:1.
^{1}H NMR (400 MHz, CDCl$_3$) δ 7.29 – 7.25 (m, 2H), 7.06 (t, *J* = 8.6 Hz, 2H), 5.06 – 4.90 (m, 1H), 4.37 – 4.25 (m, 2H), 3.69 (d, *J* = 1.0 Hz, 3H), 3.46 – 3.32 (m, 2H), 3.31 – 3.17 (m, 1H), 3.10 (d, *J* = 3.3 Hz, 0.5H), 3.00 (dd, *J* = 5.6, 1.9 Hz, 0.5H), 2.88 – 2.79 (m, 1H), 2.72 – 2.55 (m, 1H), 2.34 – 2.13 (m, 1H), 1.38 – 1.31 (m, 3H).
^{13}C NMR (101 MHz, CDCl$_3$) δ 170.68, 170.66, 167.4, 163.5 (t, *J* = 32.1 Hz), 162.5 (d, *J* = 247.5 Hz), 134.1 (d, *J* = 3.3 Hz), 134.0 (d, *J* = 3.3 Hz), 128.70 (d, *J* = 8.2 Hz), 128.69 (d, *J* = 8.2 Hz), 116.0 (d, *J* = 21.6 Hz), 115.1 (t, *J* = 252.1 Hz), 63.2, 54.2, 53.7, 52.08, 52.06, 45,0, 44.6, 42.3, 42.3, 38.0, 37.9, 33.7 (t, *J* = 24.0 Hz), 33.6 (t, *J* = 24.0 Hz), 13.9.
^{19}F NMR (376 MHz, CDCl$_3$) δ -104.2 – -107.3 (m, 2F), -113.5 (s, 1F).
HRMS (ESI-TOF) m/z: $[M+H]^+$ Calcd. for $C_{18}H_{21}F_3NO_5$ 388.1366; found: 388.1372.

Mthyl-3-(1-(3-(tert-butoxy)-3-oxopropyl)-2-oxoazetidin-3-yl)-2,2-difluoropropanoate (3v)

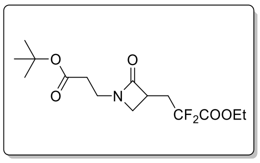

28.2 mg, colorless oil, yield: 42%. Eluent: pentane/ethyl acetate = 5/1.

^{1}H NMR (400 MHz, CDCl$_3$) δ 4.33 (q, *J* = 7.1 Hz, 2H), 3.53 – 3.43 (m, 3H), 3.41 – 3.33 (m, 1H), 3.15 (dd, *J* = 5.9, 1.7 Hz, 1H), 2.73 – 2.57 (m, 1H), 2.48 (t, *J* = 6.5 Hz, 2H), 2.37 – 2.21 (m, 1H), 1.46 (s, 9H), 1.36 (t, *J* = 7.1 Hz, 3H).

^{13}C NMR (101 MHz, CDCl$_3$) δ 170.5, 167.7, 163.6 (t, *J* = 32.5 Hz), 115.2 (t, *J* = 252.4 Hz), 81.3, 63.2, 46.6, 43.1, 37.8, 34.2, 33.8 (t, *J* = 24.0 Hz), 28.0, 13.9.

^{19}F NMR (376 MHz, CDCl$_3$) δ -104.6 (d, J = 261.0 Hz, 1F), -106.9 (d, J = 261.1 Hz, 1F).

HRMS (ESI-TOF) m/z: [M+H]$^+$ Calcd. for $C_{15}H_{24}F_2NO_5$ 336.1617; found: 336.1609.

Mthyl-3-(1-benzyl-2-oxoazetidin-3-yl)-2,2-difluoropropanoate (3w)

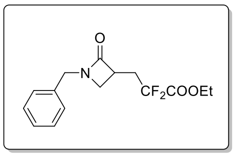

22.3 mg, colorless oil, yield: 38%. Eluent: pentane/ethyl acetate = 10/1.

^{1}H NMR (400 MHz, CDCl$_3$) δ 7.39 – 7.29 (m, 3H), 7.25 – 7.21 (m, 2H), 4.38 (s, 2H), 4.32 (q, *J* = 7.1 Hz, 2H), 3.47 – 3.40 (m, 1H), 3.40 – 3.34 (m, 1H), 3.01 (dd, *J* = 6.0, 2.0 Hz, 1H), 2.78 – 2.61 (m, 1H), 2.39 – 2.18 (m, 1H), 1.34 (t, *J* = 7.2 Hz, 3H).

^{13}C NMR (101 MHz, CDCl$_3$) δ 167.7, 163.6 (t, *J* = 32.5 Hz), 135.2, 128.9, 128.2, 127.9, 115.2 (t, *J* = 251.0 Hz), 63.2, 46.1, 45.6, 43.2, 33.8 (t, *J* = 24.0 Hz), 13.9.

^{19}F NMR (376 MHz, CDCl$_3$) δ -104.5 (d, J = 261.4 Hz, 1F), -106.8 (d, J = 261.4 Hz, 1F).

HRMS (ESI-TOF) m/z: [M+H]$^+$ Calcd. for $C_{15}H_{18}F_2NO_3$ 298.1249; found: 298.1250.

Ethyl-3-(1-benzhydryl-2-oxoazetidin-3-yl)-2,2-difluoropropanoate (3x)

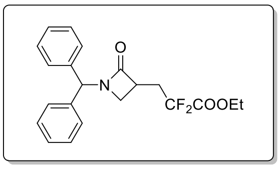

40.5 mg, colorless oil, yield: 54%. Eluent: pentane/ethyl acetate = 5/1.

^{1}H NMR (400 MHz, CDCl$_3$) δ 7.41 – 7.27 (m, 6H), 7.22 – 7.14 (m, 4H), 6.15 (s, 1H), 4.31 (q, *J* = 7.1 Hz, 2H), 3.52 – 3.39 (m, 2H), 3.07 (d, *J* = 4.4 Hz, 1H), 2.85 – 2.56 (m, 1H), 2.47 – 2.16 (m, 1H), 1.34 (t, *J* = 7.1 Hz, 3H).

^{13}C NMR (101 MHz, CDCl$_3$) δ 167.7, 163.6 (t, *J* = 32.4 Hz), 138.5, 138.4, 128.81, 128.75, 128.1, 128.0, 127.9, 127.8, 115.2 (t, *J* = 251.4 Hz), 63.2, 59.2, 45.1, 42.3, 33.9 (t, *J* = 24.0 Hz), 13.9.

^{19}F NMR (376 MHz, CDCl$_3$) δ -104.5 (d, J = 261.5 Hz, 1F), -107.0 (d, J = 261.5 Hz, 1F).

HRMS (ESI-TOF) m/z: [M+H]$^+$ Calcd. for $C_{21}H_{22}F_2NO_3$ 374.1562; found: 374.1559.

Ethyl-3-(1-((3*s*,5*s*,7*s*)-adamantan-1-yl)-2-oxoazetidin-3-yl)-2,2-difluoropropanoate (3y)

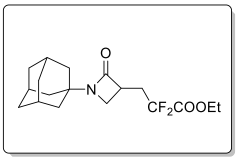

38.4 mg, colorless oil, yield: 56%. Eluent: pentane/ethyl acetate = 5/1.

^{1}H NMR (400 MHz, CDCl$_3$) δ 4.33 (q, *J* = 7.2 Hz, 2H), 3.40 (t, *J* = 5.5 Hz, 1H), 3.24 – 3.15 (m, 1H), 3.09 – 2.98 (m, 1H), 2.74 – 2.54 (m, 1H), 2.33 – 2.14 (m, 1H), 2.09 (s, 3H), 1.95 (d, *J* = 2.9 Hz, 6H), 1.73 – 1.62 (m, 6H), 1.36 (t, *J* = 7.2 Hz, 3H).

^{13}C NMR (101 MHz, CDCl$_3$) δ 166.6, 163.7 (t, *J* = 32.5 Hz), 115.2 (t, *J* = 251.4 Hz), 63.2, 53.9, 41.8, 40.7, 36.1, 34.0 (t, *J* = 23.8 Hz), 29.0, 14.0.

^{19}F NMR (376 MHz, CDCl$_3$) δ -104.5 (d, J = 260.0 Hz, 1F), -107.0 (d, J = 259.9 Hz, 1F).

HRMS (ESI-TOF) m/z: $[M+H]^+$ Calcd. for $C_{18}H_{26}F_2NO_3$ 342.1875; found: 342.1872.

Methyl-1-(3-(3-ethoxy-2,2-difluoro-3-oxopropyl)-3-methyl-2-oxoazetidin-1-yl)cyclohexane-1-carboxylate (3z)

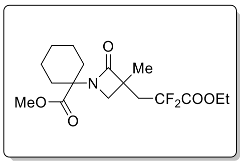

19.7 mg, colorless oil, yield: 27%. Eluent: pentane/ethyl acetate = 5/1.

^{1}H NMR (400 MHz, CDCl$_3$) δ 4.34 (q, J = 7.1 Hz, 2H), 3.73 (s, 3H), 3.41 (d, J = 5.5 Hz, 1H), 3.12 (d, J = 5.5 Hz, 1H), 2.60 – 2.36 (m, 2H), 2.27 – 2.11 (m, 2H), 1.86 – 1.74 (m, 2H), 1.70 – 1.63 (m, 3H), 1.47 – 1.42 (m, 4H), 1.39 – 1.24 (m, 5H).

^{13}C NMR (101 MHz, CDCl$_3$) δ 173.6, 172.0, 163.9 (t, J = 32.5 Hz), 115.9 (t, J = 251.4 Hz), 63.2, 62.9, 52.5, 50.8, 48.7, 37.8 (t, J = 22.8 Hz), 31.3, 24.74, 21.73, 19.3, 13.9.

^{19}F NMR (376 MHz, CDCl$_3$) δ -102.3 (d, J = 263.6 Hz, 1F), -103.5 (d, J = 263.6 Hz, 1F).

HRMS (ESI-TOF) m/z: $[M+H]^+$ Calcd. for $C_{17}H_{26}F_2NO_5$ 362.1774; found: 362.1777.

Ethyl-2,2-difluoro-3-(2-oxo-1-phenylazetidin-3-yl)propanoate (4a)

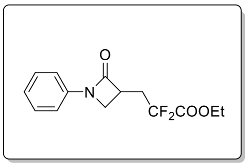

28.9 mg, colorless oil, yield: 51%. Eluent: pentane/ethyl acetate = 10/1.

^{1}H NMR (400 MHz, CDCl$_3$) δ 7.38 – 7.31 (m, 4H), 7.15 – 7.07 (m, 1H), 4.36 (q, *J* = 7.1 Hz, 2H), 3.87 (t, *J* = 5.9 Hz, 1H), 3.63 – 3.56 (m, 1H), 3.52 (dd, *J* = 6.2, 2.6 Hz, 1H), 2.85 – 2.68 (m, 1H), 2.50 – 2.33 (m, 1H), 1.37 (t, *J* = 7.1 Hz, 3H).

^{13}C NMR (101 MHz, CDCl$_3$) δ 164.7, 163.5 (t, *J* = 32.3 Hz), 138.0, 129.2, 124.3, 116.4, 115.2 (t, *J* = 250.8 Hz), 63.3, 45.1, 42.3, 33.8 (t, *J* = 23.9 Hz), 14.0.

^{19}F NMR (376 MHz, CDCl$_3$) δ -104.4 (d, *J* = 261.9 Hz, 1F), -106.8 (d, *J* = 262.1 Hz, 1F).

HRMS (ESI-TOF) m/z: $[M+H]^+$ Calcd. for $C_{14}H_{16}F_2NO_3$ 284.1093; found: 284.1099.

Ethyl-3-(1-(4-(tert-butyl)phenyl)-2-oxoazetidin-3-yl)-2,2-difluoropropanoate (4b)

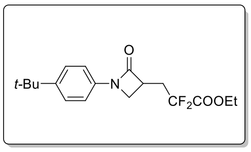

44.1 mg, colorless oil, yield: 65%. Eluent: pentane/ethyl acetate = 10/1.

^{1}H NMR (400 MHz, $CDCl_3$) δ 7.38 – 7.34 (m, 2H), 7.30 – 7.26 (m, 2H), 4.36 (q, *J* = 7.1 Hz, 2H), 3.85 (t, *J* = 5.8 Hz, 1H), 3.62 – 3.53 (m, 1H), 3.49 (dd, *J* = 6.1, 2.4 Hz, 1H), 2.84 – 2.67 (m, 1H), 2.48 – 2.30 (m, 1H), 1.37 (t, *J* = 7.1 Hz, 3H), 1.30 (s, 9H).

^{13}C NMR (101 MHz, $CDCl_3$) δ 164.4, 163.5 (t, *J* = 32.4 Hz), 147.3, 135.6, 126.0, 116.1, 115.2, (t, *J* = 250.6 Hz), 63.3, 45.1, 42.3, 34.4, 33.9 (t, *J* = 24.0 Hz), 31.4, 14.0.

^{19}F NMR (376 MHz, $CDCl_3$) δ -104.44 (d, *J* = 261.9 Hz, 1F), -106.83 (d, *J* = 262.0 Hz, 1F).

HRMS (ESI-TOF) m/z: $[M+H]^+$ Calcd. for $C_{18}H_{24}F_2NO_3$ 340.1719; found: 340.1720.

Ethyl-2,2-difluoro-3-(2-oxo-1-(4-(trifluoromethyl)phenyl)azetidin-3-yl)propanoate (4c)

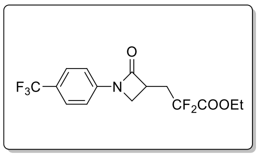

34.3 mg, colorless oil, yield: 49%. Eluent: pentane/ethyl acetate = 50/1.

^{1}H NMR (400 MHz, $CDCl_3$) δ 7.60 (d, *J* = 8.5 Hz, 2H), 7.43 (d, *J* = 8.5 Hz, 2H), 4.37 (q, *J* = 7.1 Hz, 2H), 3.92 (t, *J* = 6.0 Hz, 1H), 3.70 – 3.62 (m, 1H), 3.58 (dd, *J* = 6.2, 2.7 Hz, 1H), 2.86 – 2.67 (m, 1H), 2.53 – 2.37 (m, 1H), 1.38 (t, *J* = 7.1 Hz, 3H).

^{13}C NMR (101 MHz, $CDCl_3$) δ 165.05, 163.40 (t, *J* = 32.3 Hz), 140.60, 126.57 (q, *J* = 3.8 Hz), 126.05 (q, *J* = 32.9 Hz), 123.99 (q, *J* = 271.6 Hz), 116.27, 115.0 (t, *J* = 250. Hz), 63.39, 45.25, 42.78, 33.67 (t, *J* = 24.0 Hz), 13.93.

^{19}F NMR (376 MHz, $CDCl_3$) δ -62.10 (s, 3F), -104.4 (d, *J* = 263.0 Hz, 1F), -106.8 (d, *J* = 263.0 Hz, 1F).

HRMS (ESI-TOF) m/z: $[M+H]^+$ Calcd. for $C_{15}H_{15}F_5NO_3$ 352.0967; found: 352.0971.

Ethyl-2,2-difluoro-3-(1-(4-methoxyphenyl)-2-oxoazetidin-3-yl)propanoate (4d)

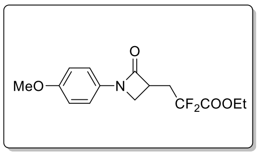

29.9 mg, colorless oil, yield: 48%. Eluent: pentane/ethyl acetate = 10/1.

^{1}H NMR (400 MHz, $CDCl_3$) δ 7.31 – 7.27 (m, 2H), 6.91 – 6.84 (m, 2H), 4.36 (q, *J* = 7.1 Hz, 2H), 3.83 (t, *J* = 5.8 Hz, 1H), 3.79 (s, 3H), 3.61 – 3.53 (m, 1H), 3.48 (dd, *J* = 6.1, 2.3 Hz, 1H), 2.84 – 2.69 (m, 1H), 2.49 – 2.33 (m, 1H), 1.37 (t, *J* = 7.1 Hz, 3H).

^{13}C NMR (101 MHz, $CDCl_3$) δ 164.1, 163.5 (t, *J* = 32.4 Hz), 156.3, 131.7, 117.6, 115.2(t, *J* = 250.7 Hz), 114.4, 63.3, 55.5, 45.2, 42.3, 42.3, 33.9 (t, *J* = 24.0 Hz), 14.0.

^{19}F NMR (376 MHz, $CDCl_3$) δ -104.5 (d, *J* = 261.8 Hz, 1F), -106.9 (d, *J* = 261.8 Hz, 1F).

HRMS (ESI-TOF) m/z: $[M+H]^+$ Calcd. for $C_{15}H_{18}F_2NO_4$ 314.1198; found: 314.1204.

Ethyl-2,2-difluoro-3-(2-oxo-1-(4-(trifluoromethoxy)phenyl)azetidin-3-yl)propanoate (4e)

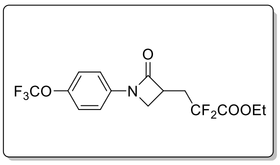

40.6 mg, colorless oil, yield: 55%. Eluent: pentane/ethyl acetate = 10/1.

^{1}H NMR (400 MHz, CDCl$_3$) δ 7.39 – 7.35 (m, 2H), 7.20 (d, *J* = 8.4 Hz, 2H), 4.36 (q, *J* = 7.1 Hz, 2H), 3.88 (t, *J* = 5.9 Hz, 1H), 3.67 – 3.59 (m, 1H), 3.54 (dd, *J* = 6.1, 2.6 Hz, 1H), 2.85 – 2.68 (m, 1H), 2.51 – 2.35 (m, 1H), 1.38 (t, *J* = 7.1 Hz, 3H).

^{13}C NMR (101 MHz, CDCl$_3$) δ 164.6, 163.4 (t, *J* = 32.3 Hz), 145.2 (q, *J* = 2.0 Hz), 136.7, 122.2, 117.4, 120.5 (q, *J* = 257.0 Hz), 115.1 (t, *J* = 250.8 Hz), 63.4, 45.3, 42.7 (t, *J* = 3.4 Hz), 33.7 (t, *J* = 24.0 Hz), 13.9.

^{19}F NMR (376 MHz, CDCl$_3$) δ -58.2 (s, 3F), -104.4 (d, *J* = 262.6 Hz, 1F), -106.8 (d, *J* = 262.6 Hz, 1F).

HRMS (ESI-TOF) m/z: $[M+H]^+$ Calcd. for $C_{15}H_{15}F_5NO_4$ 368.0916; found: 368.0921.

Ethyl-2,2-difluoro-3-(1-(4-iodophenyl)-2-oxoazetidin-3-yl)propanoate (4f)

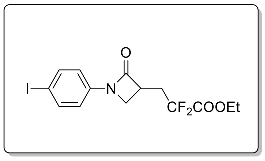

48 mg, colorless oil, yield: 59%. Eluent: pentane/ethyl acetate = 10/1.

^{1}H NMR (400 MHz, CDCl$_3$) δ 7.64 (d, *J* = 8.7 Hz, 2H), 7.11 (d, *J* = 8.7 Hz, 2H), 4.36 (q, *J* = 7.1 Hz, 2H), 3.85 (t, *J* = 5.9 Hz, 1H), 3.64 – 3.57 (m, 1H), 3.50 (dd, *J* = 6.2, 2.6 Hz, 1H), 2.83 – 2.67 (m, 1H), 2.51 – 2.34 (m, 1H), 1.37 (t, *J* = 7.1 Hz, 3H).

^{13}C NMR (101 MHz, CDCl$_3$) δ 164.7, 163.4 (t, *J* = 32.4 Hz), 138.1, 137.6, 118.2, 115.1 (t, *J* = 250.8 Hz), 87.3, 63.4, 45.1, 42.7 (t, *J* = 3.4 Hz), 33.7 (t, *J* = 24.0 Hz), 14.0.

^{19}F NMR (376 MHz, CDCl$_3$) δ -104.4 (d, *J* = 262.6 Hz, 1F), -106.8 (d, *J* = 262.5 Hz, 1F).

HRMS (ESI-TOF) m/z: $[M+H]^+$ Calcd. for $C_{14}H_{15}F_2INO_3$ 410.0059; found: 410.0052.

Ethyl-3-(1-([1,1'-biphenyl]-4-yl)-2-oxoazetidin-3-yl)-2,2-difluoropropanoate (4g)

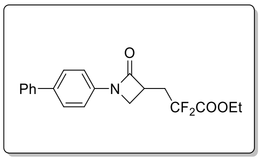

38.1 mg, colorless oil, yield: 53%. Eluent: pentane/ethyl acetate = 10/1.

^{1}H NMR (400 MHz, CDCl$_3$) δ 7.56 (t, *J* = 7.6 Hz, 4H), 7.45 – 7.39 (m, 4H), 7.33 (t, *J* = 7.3 Hz, 1H), 4.36 (q, *J* = 7.1 Hz, 2H), 3.89 (t, *J* = 5.8 Hz, 1H), 3.66 – 3.58 (m, 1H), 3.55 (dd, *J* = 6.1, 2.3 Hz, 1H), 2.87 – 2.70 (m, 1H), 2.52 – 2.36 (m, 1H), 1.38 (t, *J* = 7.1 Hz, 3H).

^{13}C NMR (101 MHz, CDCl$_3$) δ 164.7, 163.5 (t, *J* = 32.3 Hz), 140.3, 137.25, 137.18, 128.9, 127.9, 127.3, 126.8, 116.8, 115.2 (t, *J* = 250.7 Hz), 63.4, 45.2, 42.5, 33.8 (t, *J* = 24.0 Hz), 14.0.

^{19}F NMR (376 MHz, CDCl$_3$) δ -104.4 (d, *J* = 262.1 Hz, 1F), -106.8 (d, *J* = 262.1 Hz, 1F).

HRMS (ESI-TOF) m/z: $[M+H]^+$ Calcd. for $C_{20}H_{20}F_2NO_3$ 360.1406; found: 360.1403.

Ethyl-2,2-difluoro-3-(2-oxo-1-(4-phenoxyphenyl)azetidin-3-yl)propanoate (4h)

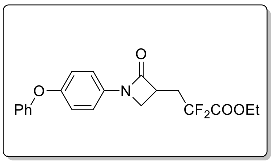

37.0 mg, colorless oil, yield: 50%. Eluent: pentane/ethyl acetate = 10/1.

^{1}H NMR (400 MHz, CDCl$_3$) δ 7.35 – 7.29 (m, 4H), 7.09 (t, *J* = 7.4 Hz, 1H), 7.03 – 6.94 (m, 4H), 4.36 (q, *J* = 7.1 Hz, 2H), 3.86 (t, *J* = 5.8 Hz, 1H), 3.64 – 3.56 (m, 1H), 3.51 (dd, *J* = 6.1, 2.4 Hz, 1H), 2.85 – 2.68 (m, 1H), 2.53 – 2.32 (m, 1H), 1.37 (t, *J* = 7.1 Hz, 3H).

^{13}C NMR (101 MHz, CDCl$_3$) δ 164.3, 163.5 (t, *J* = 32.3 Hz), 157.5, 153.4, 133.9, 129.8, 123.2, 120.0, 118.4, 117.8, 115.2, 63.3, 45.3, 42.5, 33.8 (t, *J* = 23.9 Hz), 14.0.

^{19}F NMR (376 MHz, CDCl$_3$) δ -104.4 (d, *J* = 262.1 Hz, 1F), -106.8 (d, *J* = 262.2 Hz, 1F).

HRMS (ESI-TOF) m/z: $[M+H]^+$ Calcd. for $C_{20}H_{20}F_2NO_4$ 376.1355; found: 376.1364.

Ethyl-2,2-difluoro-3-(1-(2-isopropylphenyl)-2-oxoazetidin-3-yl)propanoate (4i)

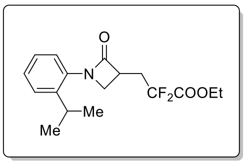

41.5 mg, colorless oil, yield: 65%. Eluent: pentane/ethyl acetate = 10/1.

^{1}H NMR (400 MHz, CDCl$_3$) δ 7.35 – 7.31 (m, 1H), 7.28 – 7.23 (m, 2H), 7.22 – 7.17 (m, 1H), 4.36 (q, *J* = 7.1 Hz, 2H), 3.92 (t, *J* = 5.8 Hz, 1H), 3.65 – 3.58 (m, 2H), 3.25 – 3.12 (m, 1H), 2.89 – 2.72 (m, 1H), 2.53 – 2.36 (m, 1H), 1.38 (t, *J* = 7.1 Hz, 3H), 1.25 (t, *J* = 6.4 Hz, 6H).

^{13}C NMR (101 MHz, CDCl$_3$) δ 166.4, 163.6 (t, *J* = 32.4 Hz), 143.6, 134.3, 127.7, 126.6, 126.5, 124.5, 115.2 (t, *J* = 251.1 Hz), 63.3, 49.6, 42.7 (t, *J* = 3.4 Hz), 34.0 (t, *J* = 23.9 Hz), 28.4, 23.6, 23.6, 14.0.

^{19}F NMR (376 MHz, CDCl$_3$) δ -104.4 (d, *J* = 261.8 Hz, 1F), -106.8 (d, *J* = 261.8 Hz, 1F).

HRMS (ESI-TOF) m/z: $[M+H]^+$ Calcd. for $C_{17}H_{22}F_2NO_3$ 326.1562; found: 326.1559.

Ethyl-2,2-difluoro-3-(1-(2-methoxyphenyl)-2-oxoazetidin-3-yl)propanoate (4j)

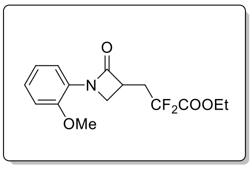

32.6 mg, colorless oil, yield: 52%. Eluent: pentane/ethyl acetate = 5/1.

^{1}H NMR (400 MHz, CDCl$_3$) δ 7.88 (dd, *J* = 7.9, 1.6 Hz, 1H), 7.13 – 7.05 (m, 1H), 6.97 – 6.86 (m, 2H), 4.36 (q, *J* = 7.1 Hz, 2H), 4.14 (dd, *J* = 7.0, 5.6 Hz, 1H), 3.85 – 3.78 (m, 4H), 3.60 – 3.53 (m, 1H), 2.86 – 2.68 (m, 1H), 2.52 – 2.33 (m, 1H), 1.38 (t, *J* = 7.1 Hz, 3H).

^{13}C NMR (101 MHz, CDCl$_3$) δ 166.4, 163.6 (t, *J* = 32.4 Hz), 150.2, 126.8, 125.5, 122.1, 121.0, 115.3 (t, *J* = 251.1 Hz), 111.8, 63.2, 55.6, 50.3, 43.9, 34.0 (t, *J* = 23.9 Hz), 14.0.

^{19}F NMR (376 MHz, CDCl$_3$) δ -104.4 (d, *J* = 260.8 Hz, 1F), -106.9 (d, *J* = 260.8 Hz, 1F).

HRMS (ESI-TOF) m/z: $[M+H]^+$ Calcd. for $C_{15}H_{18}F_2NO_4$ 314.1198; found: 314.1196.

Ethyl-2,2-difluoro-3-(1-(2-fluorophenyl)-2-oxoazetidin-3-yl)propanoate (4k)

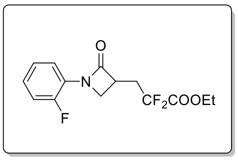

27.1 mg, colorless oil, yield: 45%. Eluent: pentane/ethyl acetate = 10/1.

^{1}H NMR (400 MHz, $CDCl_3$) δ 8.01 – 7.95 (m, 1H), 7.13 – 7.04 (m, 3H), 4.36 (q, *J* = 7.1 Hz, 2H), 4.17 – 4.11 (m, 1H), 3.84 – 3.78 (m, 1H), 3.69 – 3.62 (m, 1H), 2.84 – 2.69 (m, 1H), 2.53 – 2.37 (m, 1H), 1.38 (t, *J* = 7.1 Hz, 3H).

^{13}C NMR (101 MHz, $CDCl_3$) δ 165.8, 163.5 (t, *J* = 32.3 Hz), 152.2 (d, *J* = 245.4 Hz), 125.9 (d, *J* = 10.4 Hz), 125.1 (d, *J* = 7.4 Hz), 124.7 (d, *J* = 3.5 Hz), 121.6 (d, *J* = 2.3 Hz), 116.3 (d, *J* = 19.4 Hz), 115.1 (t, *J* = 251.8 Hz), 63.3, 49.2 (d, *J* = 6.6 Hz), 44.4, 33.8 (t, *J* = 24.0 Hz), 13.9.

^{19}F NMR (376 MHz, $CDCl_3$) δ -104.5 (d, *J* = 262.2 Hz, 1F), -106.7 (d, *J* = 262.2 Hz, 1F), -129.9 (S, 3F).

HRMS (ESI-TOF) m/z: $[M+H]^+$ Calcd. for $C_{14}H_{15}F_3NO_3$ 302.0999; found: 302.0999.

Ethyl-3-(1-(2-bromophenyl)-2-oxoazetidin-3-yl)-2,2-difluoropropanoate (4l)

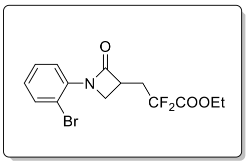

39.4 mg, colorless oil, yield: 55%. Eluent: pentane/ethyl acetate = 10/1.

^{1}H NMR (400 MHz, $CDCl_3$) δ 7.75 (dd, *J* = 8.1, 1.4 Hz, 1H), 7.55 (dd, *J* = 8.1, 1.1 Hz, 1H), 7.34 – 7.27 (m, 1H), 7.12 – 7.04 (m, 1H), 4.37 (q, *J* = 7.1 Hz, 2H), 4.23 (t, *J* = 6.0 Hz, 1H), 3.93 (dd, *J* = 6.4, 2.6 Hz, 1H), 3.69 – 3.56 (m, 1H), 2.88 – 2.70 (m, 1H), 2.56 – 2.39 (m, 1H), 1.38 (t, *J* = 7.1 Hz, 3H).

^{13}C NMR (101 MHz, $CDCl_3$) δ 166.7, 163.5 (t, *J* = 32.3 Hz), 135.9, 134.1, 128.2, 127.5, 125.4, 115.1 (t, *J* = 251.2 Hz), 114.5, 63.3, 50.1, 43.6 (t, *J* = 3.4 Hz), 33.9 (t, *J* = 24.0 Hz), 14.0.

^{19}F NMR (376 MHz, $CDCl_3$) δ -104.4 (d, *J* = 262.1 Hz, 1F), -106.7 (d, *J* = 262.0 Hz, 1F).

HRMS (ESI-TOF) m/z: $[M+H]^+$ Calcd. for $C_{14}H_{15}BrF_2NO_3$ 362.0198; found: 362.0198.

Ethyl-2,2-difluoro-3-(2-oxo-1-(2-(4,4,5,5-tetramethyl-1,3,2-dioxaborolan-2-yl)phenyl)azetidin-3-yl)propanoate (4m)

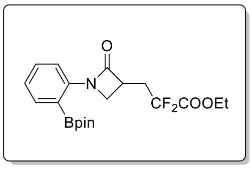

35.2 mg, colorless oil, yield: 43%. Eluent: pentane/ethyl acetate = 10/1.

^{1}H NMR (400 MHz, $CDCl_3$) δ 7.73 (dd, *J* = 7.5, 1.3 Hz, 1H), 7.46 – 7.40 (m, 1H), 7.29 (d, *J* = 7.7 Hz, 1H), 7.23 – 7.18 (m, 1H), 4.36 (q, *J* = 7.1 Hz, 2H), 3.96 (t, *J* = 5.6 Hz, 1H), 3.72 (dd, *J* = 5.8, 2.3 Hz, 1H), 3.58 – 3.49 (m, 1H), 2.85 – 2.68 (m, 1H), 2.61 – 2.45 (m, 1H), 1.41 – 1.32 (m, 15H).

^{13}C NMR (101 MHz, $CDCl_3$) δ 166.2, 163.7 (t, *J* = 32.5 Hz), 140.6, 136.1, 131.6, 125.6, 121.5, 115.4 (t, *J* = 249.7 Hz), 84.1, 63.2, 48.2, 42.5, 33.7 (t, *J* = 23.9 Hz), 25.1, 25.0, 14.0.

^{19}F NMR (376 MHz, $CDCl_3$) δ -104.4 (d, *J* = 260.8 Hz, 1F), -107.0 (d, *J* = 260.8 Hz, 1F).

HRMS (ESI-TOF) m/z: $[M+H]^+$ Calcd. for $C_{20}H_{27}BF_2NO_5$ 410.1945; found: 410.1953.

Methyl-2-(3-(3-ethoxy-2,2-difluoro-3-oxopropyl)-2-oxoazetidin-1-yl)benzoate (4n)

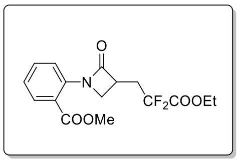

27.2 mg, colorless oil, yield: 40%. Eluent: pentane/ethyl acetate = 10/1.

^{1}H NMR (400 MHz, $CDCl_3$) δ 7.79 – 7.74 (m, 1H), 7.53 – 7.48 (m, 1H), 7.45 – 7.41 (m, 1H), 7.27 – 7.21 (m, 1H), 4.36 (q, *J* = 7.1 Hz, 2H), 3.96 (t, *J* = 5.8 Hz, 1H), 3.90 (s, 3H), 3.66 (dd, *J* = 5.9, 2.7 Hz, 1H), 3.63 – 3.57 (m, 1H), 2.83 – 2.69 (m, 1H), 2.58 – 2.40 (m, 1H), 1.38 (t, *J* = 7.1 Hz, 3H).

^{13}C NMR (101 MHz, $CDCl_3$) δ 166.9, 165.9, 163.6 (t, *J* = 32.4 Hz), 135.6, 132.5, 130.7, 125.5, 124.2, 122.0, 115.2 (t, *J* = 251.0 Hz), 63.3, 52.5, 47.9, 42.8 (t, *J* = 3.4 Hz), 33.6 (t, *J* = 24.0 Hz), 13.9.

^{19}F NMR (376 MHz, $CDCl_3$) δ -104.5 (d, *J* = 261.6 Hz, 1F), -106.8 (d, *J* = 261.6 Hz, 1F).

HRMS (ESI-TOF) m/z: $[M+H]^+$ Calcd. for $C_{16}H_{18}F_2NO_5$ 342.1148; found: 342.1152.

Ethyl-3-(1-(3-bromophenyl)-2-oxoazetidin-3-yl)-2,2-difluoropropanoate (4o)

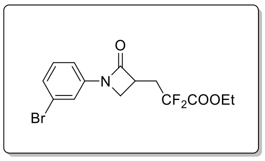

38.5 mg, colorless oil. yield: 54%. Eluent: pentane/ethyl acetate = 10/1.

^{1}H NMR (400 MHz, $CDCl_3$) δ 7.49 (t, *J* = 1.8 Hz, 1H), 7.31 – 7.27 (m, 1H), 7.26 – 7.17 (m, 2H), 4.36 (q, *J* = 7.1 Hz, 2H), 3.87 (t, *J* = 5.9 Hz, 1H), 3.67 – 3.58 (m, 1H), 3.52 (dd, *J* = 6.2, 2.6 Hz, 1H), 2.84 – 2.68 (m, 1H), 2.51 – 2.33 (m, 1H), 1.38 (t, *J* = 7.1 Hz, 3H).

^{13}C NMR (101 MHz, $CDCl_3$) δ 164.8, 163.4 (t, *J* = 32.3 Hz), 139.1, 130.6, 127.2, 123.0, 119.3, 115.0 (t, *J* = 251.3 Hz), 115.0, 63.4, 45.3, 42.6 (t, *J* = 3.5 Hz), 33.7 (t, *J* = 24.0 Hz), 14.0.

^{19}F NMR (376 MHz, $CDCl_3$) δ -104.4 (d, *J* = 262.7 Hz, 1F), -106.8 (d, *J* = 262.8 Hz, 1F).

HRMS (ESI-TOF) m/z: $[M+H]^+$ Calcd. for $C_{14}H_{15}BrF_2NO_3$ 362.0198; found: 362.0198.

Ethyl-3-(1-(3-chloro-4-methylphenyl)-2-oxoazetidin-3-yl)-2,2-difluoropropanoate (4p)

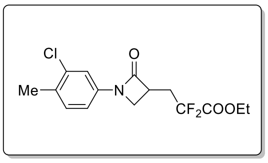

36.2 mg, colorless oil. yield: 55%. Eluent: pentane/ethyl acetate = 10/1.

^{1}H NMR (400 MHz, $CDCl_3$) δ 7.33 (d, *J* = 1.4 Hz, 1H), 7.20 – 7.14 (m, 2H), 4.36 (q, *J* = 7.1 Hz, 2H), 3.84 (t, *J* = 5.9 Hz, 1H), 3.64 – 3.56 (m, 1H), 3.50 (dd, *J* = 6.1, 2.6 Hz, 1H), 2.84 – 2.68 (m, 1H), 2.51 – 2.37 (m, 1H), 2.33 (s, 3H), 1.38 (t, *J* = 7.1 Hz, 3H).

^{13}C NMR (101 MHz, $CDCl_3$) δ 164.6, 163.5 (t, *J* = 32.3 Hz), 136.8, 134.8, 131.9, 131.3, 116.9, 115.1 (t, *J* = 251.8 Hz), 114.7, 63.3, 45.2, 42.5 (t, *J* = 3.4 Hz), 33.8 (t, *J* = 24.0 Hz), 19.5, 14.0.

^{19}F NMR (376 MHz, $CDCl_3$) δ -104.5 (d, *J* = 262.5 Hz, 1F), -106.8 (d, *J* = 262.4 Hz, 1F).

HRMS (ESI-TOF) m/z: $[M+H]^+$ Calcd. for $C_{15}H_{17}ClF_2NO_3$ 332.0860; found: 332.0862.

Ethyl-3-(1-(4-(3-ethyl-2,6-dioxopiperidin-3-yl)phenyl)-2-oxoazetidin-3-yl)-2,2-difluoropropanoate (4q)

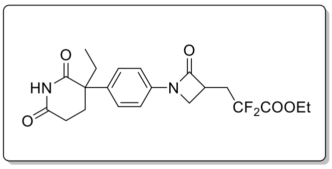

37.6 mg, colorless oil, yield: 44%. Eluent: pentane/ethyl acetate = 10/1.

^{1}H NMR (400 MHz, CDCl$_3$) δ 8.05 (s, 1H), 7.35 (d, *J* = 8.7 Hz, 2H), 7.26 (d, *J* = 8.4 Hz, 2H), 4.36 (q, *J* = 7.1 Hz, 2H), 3.88 (t, *J* = 5.9 Hz, 1H), 3.65 – 3.59 (m, 1H), 3.53 (d, *J* = 5.9 Hz, 1H), 2.85 – 2.69 (m, 1H), 2.65 – 2.57 (m, 1H), 2.50 – 2.32 (m, 3H), 2.27 – 2.17 (m, 1H), 2.08 – 1.97 (m, 1H), 1.95 – 1.85 (m, 1H), 1.37 (t, *J* = 7.1 Hz, 3H), 0.86 (t, *J* = 7.4 Hz, 3H).

^{13}C NMR (101 MHz, CDCl$_3$) δ 175.0, 172.1, 164.7, 163.5 (t, *J* = 32.4 Hz), 137.3, 134.5, 127.2, 116.9, 115.1 (t, *J* = 251.3 Hz), 63.4, 50.7, 45.1, 42.6, 33.8 (t, *J* = 23.9 Hz), 32.9, 29.2, 27.0, 14.0, 9.0.

^{19}F NMR (376 MHz, CDCl$_3$) δ -104.4 (dd, *J* = 262.5, 4.1 Hz, 1F), -106.8 (d, *J* = 262.5 Hz, 1F).

HRMS (ESI-TOF) m/z: [M+H]$^+$ Calcd. for $C_{21}H_{25}F_2N_2O_5$ 423.1726; found: 423.1724.

Ethyl-2,2-difluoro-3-(2-methyl-4-oxo-1-phenylazetidin-3-yl)propanoate (4r)

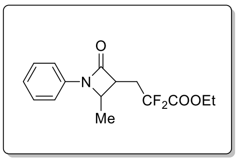

29.6 mg, colorless oil, yield: 50%. Eluent: pentane/ethyl acetate = 10/1.

Inseparable mixture of diastereomers. dr = 1.5:1.

^{1}H NMR (400 MHz, CDCl$_3$) δ 7.39 – 7.31 (m, 4H), 7.14 – 7.08 (m, 1H), 4.43 – 4.30 (m, 2.4H), 4.06 – 3.99 (m, 0.6H), 3.72 – 3.65 (m, 0.4H), 3.16 – 3.07 (m, 0.6H), 2.77 – 2.55 (m, 1H), 2.54 – 2.33 (m, 1H), 1.55 (d, *J* = 6.1 Hz, 1.8H), 1.46 (s, 1.2H), 1.41 – 1.34 (m, 3H).

^{13}C NMR (101 MHz, CDCl$_3$) δ 164.9, 164.6, 137.2, 137.1, 129.3, 124.1, 124.2, 117.1, 115.3 (t, *J* = 251.7 Hz), 63.3, 54.8, 50.7, 50.2, 45.5, 17.7, 13.9, 163.5 (t, *J* = 32.4 Hz), 33.4 (t, *J* = 23.9 Hz), 29.0 (t, *J* = 24.3 Hz).

^{19}F NMR (376 MHz, CDCl$_3$) δ -103.2 – -107.4 (m, 2F).

HRMS (ESI-TOF) m/z: [M+H]$^+$ Calcd. for $C_{15}H_{18}F_2NO_3$ 298.1249; found: 298.1251.

Methyl-1-(3-(3-(cyclopropylamino)-2,2-difluoro-3-oxopropyl)-2-oxoazetidin-1-yl)cyclohexane-1-carboxylate (5a)

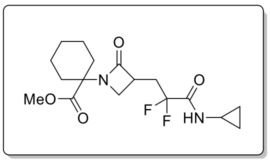

50.6 mg, colorless oil, yield: 71%. Eluent: pentane/ethyl acetate = 3/1.

^{1}H NMR (400 MHz, CDCl$_3$) δ 6.73 (s, 1H), 3.73 (s, 3H), 3.47 (t, *J* = 5.5 Hz, 1H), 3.34 (dd, *J* = 6.7, 4.2 Hz, 1H), 3.15 (dd, *J* = 5.5, 2.2 Hz, 1H), 2.83 – 2.74 (m, 1H), 2.67 – 2.52 (m, 1H), 2.52 – 2.36 (m, 1H), 2.24 (d, *J* = 13.8 Hz, 1H), 2.12 (d, *J* = 14.0 Hz, 1H), 1.87 – 1.76 (m, 2H), 1.66 (d, *J* = 9.4 Hz, 3H), 1.48 – 1.26 (m, 3H), 0.88 – 0.81 (m, 2H), 0.64 – 0.58 (m, 2H).

^{13}C NMR (101 MHz, CDCl$_3$) δ 173.5, 169.0, 165.0 (t, *J* = 28.4 Hz), 117.0 (t, *J* = 253.2 Hz), 63.2, 52.6, 44.4, 41.6 (t, *J* = 3.6 Hz), 33.6 (t, *J* = 24.3 Hz), 31.2, 31.2, 24.7, 22.6, 21.74, 21.69, 6.4.
^{19}F NMR (376 MHz, CDCl$_3$) δ -105.0 (d, J = 255.2 Hz, 1F), -105.8 (d, J = 255.2 Hz, 1F).
HRMS (ESI-TOF) m/z: $[M+H]^+$ Calcd. for $C_{17}H_{25}F_2N_2O_4$ 359.1777; found: 359.1786.

Methyl-1-(3-(2,2-difluoro-3-((4-methoxyphenyl)amino)-3-oxopropyl)-2-oxoazetidin-1-yl)cyclohexane-1-carboxylate (5b)

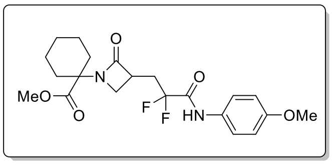

67.3 mg, colorless oil, yield: 79%. Eluent: pentane/ethyl acetate = 3/1.
^{1}H NMR (400 MHz, CDCl$_3$) δ 8.27 (s, 1H), 7.53 – 7.45 (m, 2H), 6.93 – 6.85 (m, 2H), 3.80 (s, 3H), 3.73 (s, 3H), 3.50 (t, *J* = 5.5 Hz, 1H), 3.44 – 3.36 (m, 1H), 3.17 (dd, *J* = 5.5, 2.3 Hz, 1H), 2.78 – 2.45 (m, 2H), 2.25 (d, *J* = 13.8 Hz, 1H), 2.13 (d, *J* = 14.2 Hz, 1H), 1.88 – 1.76 (m, 2H), 1.66 (d, *J* = 9.3 Hz, 3H), 1.48 – 1.26 (m, 3H).
^{13}C NMR (101 MHz, CDCl$_3$) δ 173.5, 169.0, 161.3 (t, *J* = 28.3 Hz), 157.3, 129.0, 122.0, 117.2 (t, *J* = 254.1 Hz), 114.3, 63.3, 55.5, 52.7, 44.4, 41.6, 33.9 (t, *J* = 24.5 Hz), 31.24, 31.21, 24.7, 21.8, 21.7.
^{19}F NMR (376 MHz, CDCl$_3$) δ -104.0 (d, J = 253.9 Hz, 1F), -104.8 (d, J = 253.9 Hz, 1F).
HRMS (ESI-TOF) m/z: $[M+H]^+$ Calcd. for $C_{21}H_{27}F_2N_2O_5$ 425.1883; found: 425.1892.

Methyl-1-(3-(3-((2-bromobenzyl)amino)-2,2-difluoro-3-oxopropyl)-2-oxoazetidin-1-yl)cyclohexane-1-carboxylate (5c)

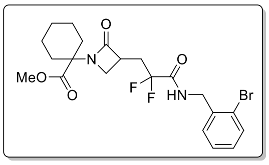

64.5 mg, colorless oil, yield: 66%. Eluent: pentane/ethyl acetate = 3/1.
^{1}H NMR (400 MHz, CDCl$_3$) δ 7.57 (d, *J* = 7.9 Hz, 1H), 7.38 (d, *J* = 7.6 Hz, 1H), 7.30 (dd, *J* = 12.5, 5.0 Hz, 1H), 7.18 (t, *J* = 7.6 Hz, 1H), 7.03 (s, 1H), 4.57 (d, *J* = 6.0 Hz, 2H), 3.72 (s, 3H), 3.39 – 3.25 (m, 2H), 3.05 (d, *J* = 3.1 Hz, 1H), 2.71 – 2.54 (m, 1H), 2.52 – 2.34 (m, 1H), 2.22 (d, *J* = 13.8 Hz, 1H), 2.06 (d, *J* = 13.8 Hz, 1H), 1.84 – 1.71 (m, 2H), 1.70 – 1.58 (m, 3H), 1.49 – 1.24 (m, 3H).
^{13}C NMR (101 MHz, CDCl$_3$) δ 173.5, 168.9, 163.6 (t, *J* = 28.8 Hz), 135.8, 133.0, 130.6, 129.8, 127.9, 123.8, 117.1 (t, *J* = 253.1 Hz), 63.2, 52.6, 44.2, 43.8, 41.6, 41.5, 33.7 (t, *J* = 24.2 Hz), 31.21, 31.17, 24.7, 21.74, 21.68.
^{19}F NMR (376 MHz, CDCl$_3$) δ -105.3 (d, J = 255.2 Hz, 1F), -106.0 (d, J = 254.9 Hz, 1F).
HRMS (ESI-TOF) m/z: $[M+H]^+$ Calcd. for $C_{21}H_{26}BrF_2N_2O_4$ 487.1039; found: 487.1044.

Methyl-1-(3-(2,2-difluoro-3-oxo-3-((thiophen-2-ylmethyl)amino)propyl)-2-oxoazetidin-1-yl)cyclohexane-1-carboxylate (5d)

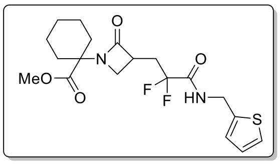

58.2 mg, colorless oil, yield: 70%. Eluent: pentane/ethyl acetate = 3/1.

^{1}H NMR (400 MHz, CDCl$_3$) δ 7.27 – 7.25 (m, 1H), 7.03 – 7.00 (m, 1H), 6.99 – 6.95 (m, 1H), 6.88 (s, 1H), 4.67 (d, *J* = 5.8 Hz, 2H), 3.73 (s, 3H), 3.41 (t, *J* = 5.5 Hz, 1H), 3.37 – 3.31 (m, 1H), 3.11 (dd, *J* = 5.5, 2.3 Hz, 1H), 2.70 – 2.54 (m, 1H), 2.54 – 2.37 (m, 1H), 2.24 (d, *J* = 13.7 Hz, 1H), 2.10 (d, *J* = 13.9 Hz, 1H), 1.84 – 1.75 (m, 2H), 1.71 – 1.61 (m, 3H), 1.47 – 1.25 (m, 3H).

^{13}C NMR (101 MHz, CDCl$_3$) δ 173.5, 168.9, 163.4 (t, *J* = 28.9 Hz), 138.9, 127.1, 126.9, 125.8, 117.1 (t, *J* = 253.1 Hz), 63.3, 52.6, 44.3, 41.6, 38.2, 33.7 (t, *J* = 24.2 Hz), 31.2, 31.2, 24.7, 21.8, 21.7.

^{19}F NMR (376 MHz, CDCl$_3$) δ -105.6 (d, J = 7.7 Hz, 2F).

HRMS (ESI-TOF) m/z: [M+H]$^+$ Calcd. for $C_{19}H_{25}F_2N_2O_4S$ 415.1498; found: 415.1506.

Methyl-1-(3-(2,2-difluoro-3-morpholino-3-oxopropyl)-2-oxoazetidin-1-yl)cyclohexane-1-carboxylate (5e)

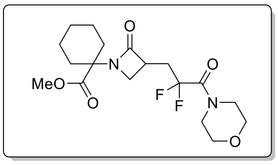

60.3 mg, colorless oil, yield: 78%. Eluent: pentane/ethyl acetate = 2/1.

^{1}H NMR (400 MHz, CDCl$_3$) δ 3.79 – 3.68 (m, 9H), 3.68 – 3.62 (m, 2H), 3.48 (dd, *J* = 6.5, 4.3 Hz, 1H), 3.45 – 3.36 (m, 1H), 3.23 – 3.14 (m, 1H), 2.74 (dd, *J* = 36.9, 15.9 Hz, 1H), 2.60 – 2.41 (m, 1H), 2.27 (d, *J* = 13.5 Hz, 1H), 2.14 (d, *J* = 13.7 Hz, 1H), 1.88 – 1.76 (m, 2H), 1.66 (d, *J* = 10.8 Hz, 3H), 1.50 – 1.27 (m, 3H).

^{13}C NMR (101 MHz, CDCl$_3$) δ 173.6, 169.3, 161.4 (t, *J* = 29.1 Hz), 118.9 (t, *J* = 255.0 Hz), 66.7, 66.6, 63.2, 52.6, 46.4 (t, *J* = 6.1 Hz), 44.7, 43.3, 41.8, 41.7, 34.1 (t, *J* = 23.6 Hz), 31.22, 31.21, 24.7, 21.74, 21.68.

^{19}F NMR (376 MHz, CDCl$_3$) δ -98.3 (d, J = 281.8 Hz, 1F), -99.1 (d, J = 282.0 Hz, 1F).

HRMS (ESI-TOF) m/z: [M+H]$^+$ Calcd. for $C_{18}H_{27}F_2N_2O_5$ 389.1883; found: 389.1893.

(1*R*,2*R*,5*R*)-2-isopropyl-5-methylcyclohexyl(2*r*,4*S*)-6-oxo-7-(2,2,2-trifluoroethyl)-5-oxaspiro[3.4]octane-2-carboxylate (5f)

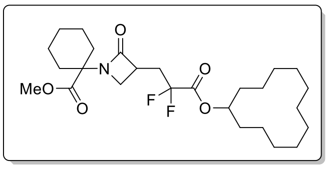

73.5 mg, colorless oil, yield: 76%. Eluent: pentane/ethyl acetate = 10/1.

^{1}H NMR (400 MHz, CDCl$_3$) δ 5.20 – 5.08 (m, 1H), 3.73 (s, 3H), 3.49 (t, *J* = 5.4 Hz, 1H), 3.42 – 3.28 (m, 1H), 3.17 (d, *J* = 5.3 Hz, 1H), 2.77 – 2.56 (m, 1H), 2.43 – 2.22 (m, 2H), 2.09 (d, *J* = 13.8 Hz, 1H), 1.89 – 1.76 (m, 4H), 1.73 – 1.53 (m, 5H), 1.46 – 1.28 (m, 21H).

^{13}C NMR (101 MHz, CDCl$_3$) δ 173.4, 168.9, 163.3 (t, *J* = 32.1 Hz), 115.4 (t, *J* = 250.9 Hz), 76.2, 63.3, 52.6, 44.3, 41.41, 41.38, 33.8 (t, *J* = 23.9 Hz), 31.2, 28.8, 28.7, 24.7, 24.0, 23.8, 23.2, 23.2, 23.1, 23.0, 21.8, 21.7, 20.7.

^{19}F NMR (376 MHz, CDCl$_3$) δ -104.7 (d, *J* = 259.4 Hz, 1F), -107.0 (d, *J* = 259.4 Hz, 1F).

HRMS (ESI-TOF) m/z: [M+H]$^+$ Calcd. for $C_{26}H_{42}F_2NO_5$ 486.3026; found: 486.3032.

Methyl-1-(3-(2,2-difluoro-3-oxo-3-(((3a*S*,5a*R*,8a*R*,8b*S*)-2,2,7,7-tetramethyltetrahydro-3a*H*-bis([1,3]dioxolo)[4,5-*b*:4',5'-*d*]pyran-3a-yl)methoxy)propyl)-2-oxoazetidin-1-yl)cyclohexane-1-carboxylate (5g)

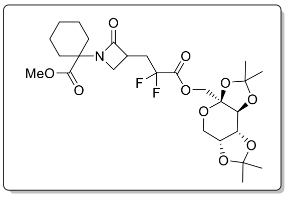

95.4 mg, colorless oil, yield: 85%. Eluent: pentane/ethyl acetate = 3/1.

Inseparable mixture of diastereomers. dr = 1:1.

^{1}H NMR (400 MHz, CDCl$_3$) δ 4.63 (d, *J* = 7.9 Hz, 1H), 4.57 – 4.49 (m, 1H), 4.33 (d, *J* = 2.4 Hz, 1H), 4.27 – 4.15 (m, 2H), 3.93 (d, *J* = 12.8 Hz, 1H), 3.77 (d, *J* = 13.0 Hz, 1H), 3.73 (s, 3H), 3.51 – 3.45 (m, 1H), 3.44 – 3.34 (m, 1H), 3.19 – 3.10 (m, 1H), 2.80 – 2.59 (m, 1H), 2.49 – 2.34 (m, 1H), 2.29 (d, *J* = 13.8 Hz, 1H), 2.08 (d, *J* = 13.9 Hz, 1H), 1.87 – 1.76 (m, 2H), 1.66 (d, *J* = 9.9 Hz, 3H), 1.55 (s, 3H), 1.51 – 1.26 (m, 12H).

^{13}C NMR (101 MHz, CDCl$_3$) δ 173.4, 168.7, 163.1 (t, *J* = 32.8 Hz), 115.4 (t, *J* = 251.8 Hz), 109.3, 109.2, 100.8, 70.7, 70.2, 70.2, 69.9, 66.6, 63.3, 61.4, 52.6, 44.3, 44.2, 41.4, 34.0 (t, *J* = 23.9 Hz), 31.2, 26.5, 25.9, 25.0, 24.7, 24.0, 21.8, 21.7.

^{19}F NMR (376 MHz, CDCl$_3$) δ -104.2 – -106.5 (m, 2F).

HRMS (ESI-TOF) m/z: [M+H]$^+$ Calcd. for $C_{26}H_{38}F_2NO_{10}$ 562.2458; found: 562.2460.

Methyl-1-(3-(2,2-difluoro-3-oxo-3-phenoxypropyl)-2-oxoazetidin-1-yl)cyclohexane-1-carboxylate (5h)

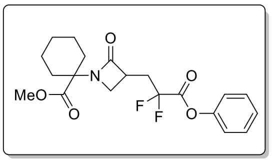

49.3 mg, colorless oil, yield: 62%. Eluent: pentane/ethyl acetate = 5/1.

^{1}H NMR (400 MHz, CDCl$_3$) δ 7.43 (t, *J* = 7.9 Hz, 2H), 7.31 (t, *J* = 7.4 Hz, 1H), 7.18 (d, *J* = 7.9 Hz, 2H), 3.74 (s, 3H), 3.54 (t, *J* = 5.5 Hz, 1H), 3.51 – 3.42 (m, 1H), 3.23 (dd, *J* = 5.4, 1.9 Hz, 1H), 2.98 – 2.75 (m, 1H), 2.61 – 2.43 (m, 1H), 2.32 (d, *J* = 13.7 Hz, 1H), 2.09 (d, *J* = 13.9 Hz, 1H), 1.90 – 1.76 (m, 2H), 1.67 (d, *J* = 9.8 Hz, 3H), 1.51 – 1.26 (m, 3H).

^{13}C NMR (101 MHz, CDCl$_3$) δ 173.5, 168.7, 162.1 (t, *J* = 33.6 Hz), 149.7, 129.8, 126.9, 120.8, 115.4 (t, *J* = 251.2 Hz), 63.4, 52.7, 44.3, 41.4, 34.0 (t, *J* = 23.7 Hz), 31.3, 31.2, 24.7, 21.8, 21.7.

^{19}F NMR (376 MHz, CDCl$_3$) δ -104.0 (d, *J* = 262.6 Hz, 1F), -106.4 (d, *J* = 262.6 Hz, 1F).

HRMS (ESI-TOF) m/z: [M+H]$^+$ Calcd. for $C_{20}H_{24}F_2NO_5$ 396.1617; found: 396.1617.

Methyl-1-(3-(2,2-difluoro-3-oxo-3-(((*S*)-2,5,7,8-tetramethyl-2-((4*R*,8*R*)-4,8,12-trimethyltridecyl)chroman-6-yl)oxy)propyl)-2-oxoazetidin-1-yl)cyclohexane-1-carboxylate (5i)

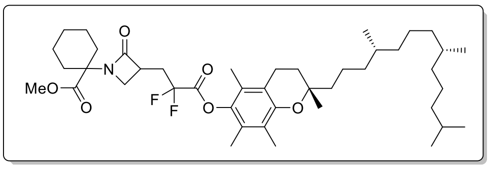

89.3 mg, colorless oil, yield: 61%. Eluent: pentane/ethyl acetate = 5/1.

^{1}H NMR (400 MHz, CDCl$_3$) δ 3.74 (s, 3H), 3.59 – 3.47 (m, 2H), 3.27 (d, *J* = 3.9 Hz, 1H), 2.96 – 2.79 (m, 1H), 2.64 – 2.46 (m, 3H), 2.32 (d, *J* = 13.7 Hz, 1H), 2.14 – 2.07 (m, 4H), 2.02 (s, 3H), 1.98 (s, 3H), 1.88 – 1.75 (m, 4H), 1.67 (d, *J* = 10.0 Hz, 3H), 1.57 – 1.24 (m, 21H), 1.16 – 1.04 (m, 6H), 0.89 – 0.82 (m, 13H).
^{13}C NMR (101 MHz, CDCl$_3$) δ 173.5, 168.9, 162.3 (t, *J* = 32.7 Hz), 150.1, 139.5, 126.1, 124.5, 123.6, 117.8, 115.8 (t, *J* = 251.4 Hz), 75.3, 63.4, 52.6, 44.6, 41.4, 39.4, 37.5, 37.44, 37.42, 37.3, 33.84 (t, *J* = 23.4 Hz), 32.81, 32.7, 31.28, 31.26, 31.0, 28.0, 24.8, 24.7, 24.4, 22.7, 22.6, 21.8, 21.7, 21.0, 20.6, 19.8, 19.7, 12.8, 11.9, 11.8.
^{19}F NMR (376 MHz, CDCl$_3$) δ -103.9 (d, *J* = 264.3 Hz, 1F), -106.4 (d, *J* = 264.3 Hz, 1F).
HRMS (ESI-TOF) m/z: $[M+H]^+$ Calcd. for $C_{43}H_{68}F_2NO_6$ 732.5009; found: 732.5013.

Methyl-1-(3-(2,2-difluoro-3-oxo-3-(((1*R*,2*R*,4*R*)-1,7,7-trimethylbicyclo[2.2.1]heptan-2-yl)oxy)propyl)-2-oxoazetidin-1-yl)cyclohexane-1-carboxylate (5j)

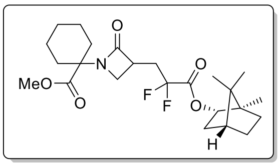

71.2 mg, colorless oil, yield: 78%. Eluent: pentane/ethyl acetate = 10/1.
Inseparable mixture of diastereomers. dr = 1:1.
^{1}H NMR (400 MHz, CDCl$_3$) δ 4.81 (d, *J* = 4.5 Hz, 1H), 3.73 (s, 3H), 3.48 (t, *J* = 5.2 Hz, 1H), 3.40 – 3.30 (m, 1H), 2.72 – 2.56 (m, 1H), 2.42 – 2.24 (m, 2H), 2.09 (d, *J* = 13.8 Hz, 1H), 1.88 – 1.77 (m, 5H), 1.74 – 1.57 (m, 5H), 1.49 – 1.26 (m, 3H), 1.21 – 1.09 (m, 2H), 0.99 (s, 3H), 0.88 (s, 3H), 0.86 (s, 3H).
^{13}C NMR (101 MHz, CDCl$_3$) δ 173.5, 168.9, 163.2 (t, *J* = 32.3 Hz), 115.4 (t, *J* = 251.8 Hz), 84.1, 84.0, 63.3, 52.6, 49.1, 49.1, 47.0, 45.0, 44.4, 41.4, 38.4, 38.3, 33.8 (t, *J* = 23.9 Hz), 33.4, 31.2, 26.9, 24.7, 21.8, 21.7, 20.0, 19.7, 11.3, 11.3.
^{19}F NMR (376 MHz, CDCl$_3$) δ -104.3 – -107.6 (m, 2F).
HRMS (ESI-TOF) m/z: $[M+H]^+$ Calcd. for $C_{24}H_{36}F_2NO_5$ 456.2556; found: 456.2548.

Methyl-1-(3-(3-(((10*R*,13*S*,17*S*)-10,13-dimethyl-3-oxo-2,3,6,7,8,9,10,11,12,13,14,15,16,17-tetradecahydro-1H-cyclopenta[a]phenanthren-17-yl)oxy)-2,2-difluoro-3-oxopropyl)-2-oxoazetidin-1-yl)cyclohexane-1-carboxylate (5k)

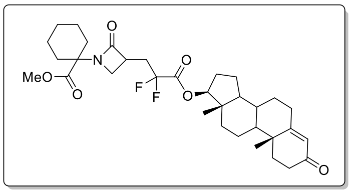

66.9 mg, colorless oil, yield: 57%. Eluent: pentane/ethyl acetate = 5/1.
Inseparable mixture of diastereomers. dr = 1:1.
^{1}H NMR (400 MHz, CDCl$_3$) δ 5.73 (s, 1H), 4.81 – 4.70 (m, 1H), 3.74 (s, 3H), 3.52 – 3.45 (m, 1H), 3.41 – 3.31 (m, 1H), 3.18 – 3.14 (m, 1H), 2.75 – 2.59 (m, 1H), 2.44 – 2.21 (m, 7H), 2.12 – 2.00 (m, 2H), 1.90 – 1.79 (m, 4H), 1.73 – 1.57 (m, 8H), 1.49 – 1.28 (m, 6H), 1.20 (s, 3H), 1.16 – 1.03 (m, 2H), 1.00 – 0.91 (m, 1H), 0.89 (s, 3H).
^{13}C NMR (101 MHz, CDCl$_3$) δ 199.3, 173.4, 170.6, 168.9, 163.6 (t, *J* = 32.4 Hz), 124.0, 115.4 (t, *J* = 250.9 Hz), 85.3, 85.2, 63.3, 53.6, 52.6, 50.1, 44.3, 42.9, 42.8, 41.4, 38.6, 36.5, 35.7, 35.3, 33.9, 33.8 (t, *J*

= 23.7 Hz), 32.6, 31.4, 31.2, 27.20, 27.17, 24.7, 23.4, 21.8, 21.7, 20.4, 17.4, 12.0.
^{19}F NMR (376 MHz, CDCl$_3$) δ -104.0 – -107.3 (m, 1F).
HRMS (ESI-TOF) m/z: [M+H]$^+$ Calcd. for $C_{33}H_{46}F_2NO_6$ 590.3288; found: 590.3290.

Diethyl-2-(3-(2,2-difluoro-3-oxo-3-(((3a*S*,5a*R*,8a*R*,8b*S*)-2,2,7,7-tetramethyltetrahydro-3a*H*-bis([1,3]dioxolo)[4,5-b:4',5'-d]pyran-3a-yl)methoxy)propyl)-2-oxoazetidin-1-yl)malonate (5l)

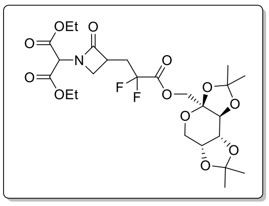

87.1 mg, colorless oil, yield: 75%. Eluent: pentane/ethyl acetate = 5/1.
Inseparable mixture of diastereomers. dr = 1:1.
^{1}H NMR (400 MHz, CDCl$_3$) δ 5.18 (s, 1H), 4.63 (dd, *J* = 7.9, 2.5 Hz, 1H), 4.53 (dd, *J* = 11.5, 6.9 Hz, 1H), 4.35 – 4.14 (m, 7H), 3.93 (dd, *J* = 12.9, 1.3 Hz, 1H), 3.86 – 3.73 (m, 2H), 3.58 – 3.47 (m, 2H), 2.79 – 2.62 (m, 1H), 2.51 – 2.35 (m, 1H), 1.55 (s, 3H), 1.48 (s, 3H), 1.41 (s, 3H), 1.36 – 1.29 (m, 9H).
^{13}C NMR (101 MHz, CDCl$_3$) δ 168.1, 164.8, 164.8, 163.0 (t, *J* = 32.6 Hz), 115.1 (t, *J* = 251.2 Hz), 109.3, 109.2, 100.8, 70.7, 70.2, 69.9, 66.7, 66.65, 62.62, 62.56, 61.4, 56.7, 46.4, 43.7, 33.8 (t, *J* = 23.9 Hz), 26.5, 25.8, 25.0, 24.0, 14.0.
^{19}F NMR (376 MHz, CDCl$_3$) δ -104.2 – -106.7 (m, 2F).
HRMS (ESI-TOF) m/z: [M+H]$^+$ Calcd. for $C_{25}H_{36}F_2NO_{16}$ 580.2200; found: 580.2196.

Diethyl-2-(3-(2,2-difluoro-3-(heptyloxy)-3-oxopropyl)-2-oxoazetidin-1-yl)malonate (5m)

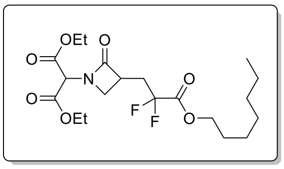

72.3 mg, colorless oil, yield: 83%. Eluent: pentane/ethyl acetate = 10/1.
^{1}H NMR (400 MHz, CDCl$_3$) δ 5.18 (s, 1H), 4.33 – 4.20 (m, 6H), 3.82 (t, *J* = 5.9 Hz, 1H), 3.57 – 3.48 (m, 2H), 2.77 – 2.59 (m, 1H), 2.49 – 2.31 (m, 1H), 1.76 – 1.67 (m, 2H), 1.41 – 1.25 (m, 14H), 0.89 (t, *J* = 6.6 Hz, 3H).
^{13}C NMR (101 MHz, CDCl$_3$) δ 168.2, 164.9, 164.8, 163.6 (t, *J* = 32.3 Hz), 115.1 (t, *J* = 251.1 Hz), 67.3, 62.62, 62.57, 56.7, 46.5, 43.8, 33.7 (t, *J* = 24.0 Hz), 31.6, 28.8, 28.2, 25.6, 22.5, 14.0.
^{19}F NMR (376 MHz, CDCl$_3$) δ -104.6 (d, *J* = 261.7 Hz, 1F), -106.8 (d, *J* = 261.7 Hz, 1F).
HRMS (ESI-TOF) m/z: [M+H]$^+$ Calcd. for $C_{20}H_{32}F_2NO_7$ 436.2141; found: 436.2138.

Methyl-(3-(1-(4-(tert-butyl)phenyl)-2-oxoazetidin-3-yl)-2,2-difluoropropanoyl)glycinate (5n)

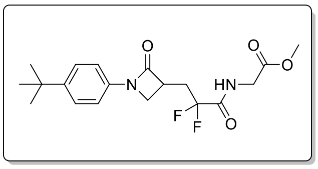

36.6 mg, colorless oil, yield: 48%. Eluent: pentane/ethyl acetate = 20/1.
^{1}H NMR (400 MHz, CDCl$_3$) δ 7.38 – 7.33 (m, 2H), 7.29 – 7.26 (m, 2H), 7.07 (s, 1H), 4.18 – 4.03 (m, 2H), 3.85 (t, *J* = 5.9 Hz, 1H), 3.80 (s, 3H), 3.66 – 3.57 (m, 1H), 3.49 (dd, *J* = 6.2, 2.6 Hz, 1H), 2.84 – 2.65

(m, 1H), 2.57 – 2.41 (m, 1H), 1.30 (s, 10H).
^{13}C NMR (101 MHz, CDCl$_3$) δ 169.1, 164.6, 163.8 (t, *J* = 32.1 Hz), 147.3, 135.6, 126.0, 116.9 (t, *J* = 250.8 Hz), 116.1, 52.8, 45.1, 42.4, 41.0, 34.4, 33.7 (t, *J* = 24.3 Hz), 31.4.
^{19}F NMR (376 MHz, CDCl$_3$) δ -104.9 (d, *J* = 257.3 Hz, 1F), -105.9 (d, *J* = 257.2 Hz, 1F).
HRMS (ESI-TOF) m/z: $[M+H]^+$ Calcd. for $C_{19}H_{25}F_2N_2O_4$ 383.1777; found: 383.1783.

3-(1-(4-(*tert*-Butyl)phenyl)-2-oxoazetidin-3-yl)-*N*,*N*-diethyl-2,2-difluoropropanamide (5o)

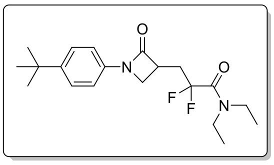

38.9 mg, colorless oil, yield: 53%. Eluent: pentane/ethyl acetate = 5/1.
^{1}H NMR (400 MHz, CDCl$_3$) δ 7.37 – 7.34 (m, 2H), 7.31 – 7.27 (m, 2H), 3.83 (t, *J* = 5.8 Hz, 1H), 3.64 – 3.57 (m, 1H), 3.57 – 3.50 (m, 3H), 3.40 (q, *J* = 7.1 Hz, 2H), 2.87 – 2.72 (m, 1H), 2.63 – 2.46 (m, 1H), 1.30 (s, 9H), 1.22 (t, *J* = 7.0 Hz, 3H), 1.17 (t, *J* = 7.1 Hz, 3H).
^{13}C NMR (101 MHz, CDCl$_3$) δ 165.3, 162.1 (t, *J* = 28.7 Hz), 147.0, 135.7, 126.0, 119.0 (t, *J* = 255.8 Hz), 116.1, 45.7, 43.0, 41.8, 41.5, 34.43, 34.35 (t, *J* = 24.2 Hz), 31.4, 14.3, 12.3.
^{19}F NMR (376 MHz, CDCl$_3$) δ -98.8 (d, *J* = 279.8 Hz, 1F), -99.6 (d, *J* = 279.8 Hz, 1F).
HRMS (ESI-TOF) m/z: $[M+H]^+$ Calcd. for $C_{20}H_{29}F_2N_2O_2$ 367.2192; found: 367.2202.

3-Phenylpropyl 3-(1-(4-(*tert*-butyl)phenyl)-2-oxoazetidin-3-yl)-2,2-difluoropropanoate (5p)

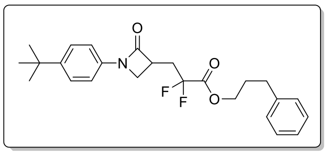

48.9 mg, colorless oil, yield: 57%. Eluent: pentane/ethyl acetate = 5/1.
^{1}H NMR (400 MHz, CDCl$_3$) δ 7.36 (d, *J* = 8.5 Hz, 2H), 7.31 – 7.25 (m, 4H), 7.22 – 7.16 (m, 3H), 4.29 (t, *J* = 6.5 Hz, 2H), 3.82 (t, *J* = 5.8 Hz, 1H), 3.59 – 3.52 (m, 1H), 3.47 (dd, *J* = 6.1, 2.4 Hz, 1H), 2.82 – 2.66 (m, 3H), 2.45 – 2.27 (m, 1H), 2.13 – 2.01 (m, 2H), 1.30 (s, 10H).
^{13}C NMR (101 MHz, CDCl$_3$) δ 164.4, 163.6 (t, *J* = 32.4 Hz), 147.3, 140.5, 135.6, 128.6, 128.4, 126.3, 126.0, 116.1, 115.2 (t, *J* = 251.0 Hz), 66.4, 45.1, 42.3, 34.5, 33.8 (t, *J* = 23.9 Hz), 31.9, 31.4, 29.7.
^{19}F NMR (376 MHz, CDCl$_3$) δ -104.3 (d, *J* = 262.3 Hz, 1F), -106.7 (d, *J* = 262.3 Hz, 1F).
HRMS (ESI-TOF) m/z: $[M+H]^+$ Calcd. for $C_{25}H_{30}F_2NO_3$ 430.2188; found: 430.2196.

(1*R*,2*S*,5*R*)-2-isopropyl-5-methylcyclohexyl-3-(1-(4-(*tert*-butyl)phenyl)-2-oxoazetidin-3-yl)-2,2-difl uoropropanoate (5q)

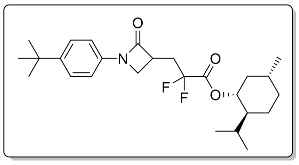

53.9 mg, colorless oil, yield: 60%. Eluent: pentane/ethyl acetate = 10/1.
Inseparable mixture of diastereomers. dr = 1:1.
^{1}H NMR (400 MHz, CDCl$_3$) δ 7.38 – 7.34 (m, 2H), 7.30 – 7.26 (m, 2H), 4.89 – 4.79 (m, 1H), 3.84 (t, *J* = 5.5 Hz, 1H), 3.61 – 3.54 (m, 1H), 3.54 – 3.49 (m, 1H), 2.84 – 2.67 (m, 1H), 2.46 – 2.29 (m, 1H),

2.07 – 2.00 (m, 1H), 1.89 – 1.81 (m, 1H), 1.77 – 1.67 (m, 2H), 1.55 – 1.47 (m, 2H), 1.30 (s, 10H), 1.15 – 1.01 (m, 2H), 0.97 – 0.90 (m, 7H), 0.81 – 0.74 (m, 3H).

^{13}C NMR (101 MHz, CDCl$_3$) δ 164.5, 163.2 (t, *J* = 32.1 Hz), 147.3, 135.6, 126.0, 116.1, 115.3 (t, *J* = 251.1 Hz), 77.9, 46.8, 46.8, 45.1, 42.3, 40.34, 40.31, 34.4, 34.0, 33.8 (t, *J* = 23.8 Hz), 31.4, 31.4, 26.2, 23.3, 23.3, 21.9, 20.7, 16.2, 16.1.

^{19}F NMR (376 MHz, CDCl$_3$) δ -104.2 – -107.2 (m, 2F).

HRMS (ESI-TOF) m/z: [M+H]$^+$ Calcd. for $C_{26}H_{38}F_2NO_3$ 450.2814; found: 450.2823.

Methyl-1-(3-(2,2-difluoro-3-oxo-3-phenylpropyl)-2-oxoazetidin-1-yl)cyclohexane-1-carboxylate (6a)

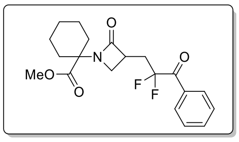

35.9 mg, colorless oil, yield: 47%. Eluent: pentane/ethyl acetate = 10/1.

^{1}H NMR (400 MHz, CDCl$_3$) δ 8.10 (d, *J* = 7.5 Hz, 2H), 7.69 – 7.62 (m, 1H), 7.51 (t, *J* = 7.8 Hz, 2H), 3.73 (s, 3H), 3.51 (t, *J* = 5.4 Hz, 1H), 3.48 – 3.41 (m, 1H), 3.24 – 3.18 (m, 1H), 2.90 – 2.74 (m, 1H), 2.59 – 2.42 (m, 1H), 2.29 (d, *J* = 13.8 Hz, 1H), 2.12 (d, *J* = 14.0 Hz, 1H), 1.87 – 1.77 (m, 2H), 1.66 (d, *J* = 9.6 Hz, 3H), 1.49 – 1.28 (m, 3H).

^{13}C NMR (101 MHz, CDCl$_3$) δ 188.7 (t, *J* = 30.8 Hz), 173.6, 169.2, 134.6, 131.6, 130.2 (t, *J* = 3.2 Hz), 128.8, 119.1 (t, *J* = 254.2 Hz), 63.3, 52.6, 44.6, 41.6, 33.4 (t, *J* = 23.4 Hz), 31.3, 24.7, 21.8, 21.7.

^{19}F NMR (376 MHz, CDCl$_3$) δ -98.8 (d, *J* = 288.2 Hz, 1F), -100.2 (d, *J* = 288.2 Hz, 1F).

HRMS (ESI-TOF) m/z: [M+H]$^+$ Calcd. for $C_{20}H_{24}F_2NO_4$ 380.1688; found: 380.1695.

Methyl-1-(3-(2-(diethoxyphosphoryl)-2,2-difluoroethyl)-2-oxoazetidin-1-yl)cyclohexane-1-carboxylate (6b)

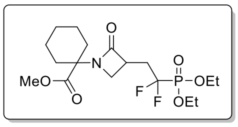

31.5 mg, colorless oil, yield: 38%. Eluent: pentane/ethyl acetate = 2/1.

^{1}H NMR (400 MHz, CDCl$_3$) δ 4.33 – 4.24 (m, 4H), 3.73 (s, 3H), 3.53 – 3.45 (m, 2H), 3.20 – 3.15 (m, 1H), 2.75 – 2.57 (m, 1H), 2.46 – 2.23 (m, 2H), 2.11 (d, *J* = 14.0 Hz, 1H), 1.87 – 1.76 (m, 2H), 1.71 – 1.62 (m, 3H), 1.52 – 1.24 (m, 9H).

^{13}C NMR (101 MHz, CDCl$_3$) δ 173.5, 169.2, 123.7, 121.6, 121.1, 120.1 (td, *J* = 260.5, 215.6 Hz), 119.0, 118.6, 116.4, 64.7 (d, *J* = 6.7 Hz), 64.6 (d, *J* = 6.9 Hz), 63.2, 52.6, 44.7, 41.1 (d, *J* = 4.7 Hz), 33.4 (td, *J* = 21.2, 15.4 Hz), 24.7, 21.8, 21.7, 16.4, 16.4.

^{19}F NMR (376 MHz, CDCl$_3$) δ -110.7 (dd, *J* = 297.4, 107.3 Hz, 1F), -112.2 (dd, *J* = 297.4, 105.8 Hz, 1F).

^{31}P NMR (162 MHz, CDCl$_3$) δ 6.3 (t, *J* = 106.6 Hz).

HRMS (ESI-TOF) m/z: [M+H]$^+$ Calcd. for $C_{17}H_{29}F_2NO_6P$ 412.1695; found: 412.1693.

Methyl-1-(2-oxo-3-(3-oxo-3-phenylpropyl)azetidin-1-yl)cyclohexane-1-carboxylate (6c)

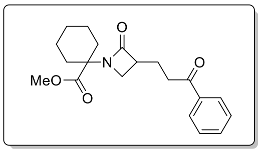

35.5 mg, colorless oil, yield: 52%. Eluent: pentane/ethyl acetate = 10/1.

^{1}H NMR (400 MHz, CDCl$_3$) δ 8.02 – 7.96 (m, 2H), 7.56 (t, *J* = 7.4 Hz, 1H), 7.46 (t, *J* = 7.6 Hz, 2H), 3.72 (s, 3H), 3.41 (t, *J* = 5.3 Hz, 1H), 3.32 – 3.16 (m, 3H), 3.03 (dd, *J* = 5.2, 2.5 Hz, 1H), 2.29 – 2.11 (m, 4H), 1.86 – 1.76 (m, 2H), 1.65 (d, *J* = 9.7 Hz, 3H), 1.49 – 1.40 (m, 2H), 1.36 – 1.26 (m, 1H).

^{13}C NMR (101 MHz, CDCl$_3$) δ 199.5, 173.7, 171.1, 136.8, 133.1, 128.6, 128.1, 63.0, 52.5, 47.1, 43.7, 35.7, 31.3, 24.8, 23.5, 21.83, 21.77.

HRMS (ESI-TOF) m/z: $[M+H]^+$ Calcd. for $C_{20}H_{26}NO_4$ 344.1856; found: 344.1865.

Methyl-1-(3-(2-cyanoethyl)-2-oxoazetidin-1-yl)cyclohexane-1-carboxylate (6d)

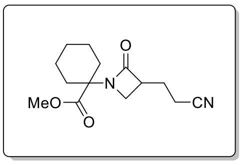

34.3 mg, colorless oil, yield: 65%. Eluent: pentane/ethyl acetate = 10/1.

^{1}H NMR (400 MHz, CDCl$_3$) δ 3.74 (s, 3H), 3.46 (t, *J* = 5.4 Hz, 1H), 3.28 – 3.20 (m, 1H), 3.13 – 3.07 (m, 1H), 2.66 – 2.49 (m, 2H), 2.32 (d, *J* = 13.9 Hz, 1H), 2.22 – 2.03 (m, 3H), 1.88 – 1.76 (m, 2H), 1.67 (d, *J* = 10.0 Hz, 3H), 1.47 – 1.29 (m, 3H).

^{13}C NMR (101 MHz, CDCl$_3$) δ 173.6, 169.7, 119.1, 63.3, 52.6, 46.5, 43.3, 31.2, 31.1, 25.0, 24.7, 21.8, 21.7, 15.2.

HRMS (ESI-TOF) m/z: $[M+H]^+$ Calcd. for $C_{14}H_{21}N_2O_3$ 265.1547; found: 265.1547.

Methyl-1-(2-oxo-3-(2,2,2-trichloroethyl)azetidin-1-yl)cyclohexane-1-carboxylate (6e)

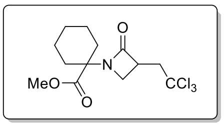

51.5 mg, colorless oil, yield: 76%. Eluent: pentane/ethyl acetate = 10/1.

^{1}H NMR (400 MHz, CDCl$_3$) δ 3.75 (s, 3H), 3.67 – 3.57 (m, 2H), 3.39 (dd, *J* = 5.6, 2.4 Hz, 1H), 3.31 (dd, *J* = 15.0, 1.9 Hz, 1H), 3.04 (dd, *J* = 15.0, 11.2 Hz, 1H), 2.33 (d, *J* = 13.8 Hz, 1H), 2.08 (d, *J* = 13.9 Hz, 1H), 1.90 – 1.78 (m, 2H), 1.72 – 1.63 (m, 3H), 1.50 – 1.29 (m, 3H).

^{13}C NMR (101 MHz, CDCl$_3$) δ 173.5, 168.5, 98.4, 63.5, 54.0, 52.7, 46.0, 45.6, 31.3, 31.2, 24.7, 21.81, 21.7.

HRMS (ESI-TOF) m/z: $[M+H]^+$ Calcd. for $C_{13}H_{19}Cl_3NO_3$ 342.0425; found: 342.0430.

Methyl-1-(2-oxo-3-(2,2,2-trifluoroethyl)azetidin-1-yl)cyclohexane-1-carboxylate (6f)

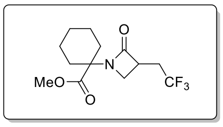

42.2 mg, colorless oil, yield: 72%. Eluent: pentane/ethyl acetate = 10/1.

^{1}H NMR (400 MHz, $CDCl_3$) δ 3.74 (s, 3H), 3.51 (t, *J* = 5.5 Hz, 1H), 3.41 – 3.33 (m, 1H), 3.18 (dd, *J* = 5.5, 2.0 Hz, 1H), 2.79 – 2.63 (m, 1H), 2.48 – 2.28 (m, 2H), 2.08 (d, *J* = 14.0 Hz, 1H), 1.89 – 1.77 (m, 2H), 1.67 (d, *J* = 9.9 Hz, 3H), 1.48 – 1.29 (m, 3H).
^{13}C NMR (101 MHz, $CDCl_3$) δ 173.4, 168.2, 126.4 (q, *J* = 276.5 Hz), 63.4, 52.6, 43.9, 41.7 (q, *J* = 2.5 Hz), 33.3 (q, *J* = 29.8 Hz), 31.2, 31.2, 24.6, 21.8, 21.7.
^{19}F NMR (376 MHz, $CDCl_3$) δ -65.6.
HRMS (ESI-TOF) m/z: $[M+H]^+$ Calcd. for $C_{13}H_{19}F_3NO_3$ 294.1312; found: 294.1321.

A methyl 1-(3-(2,2-difluoro-3-hydroxypropyl)-2-oxoazetidin-1-yl)cyclohexane-1-carboxylate (7)

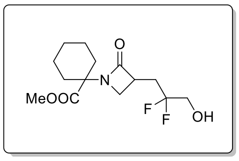

79.4 mg, colorless oil, yield: 87%. Eluent: pentane/ethyl acetate = 2/1.
^{1}H NMR (400 MHz, $CDCl_3$) δ 3.89 – 3.70 (m, 6H), 3.52 (t, *J* = 5.5 Hz, 1H), 3.42 – 3.34 (m, 1H), 3.12 (dd, *J* = 5.4, 2.5 Hz, 1H), 2.59 – 2.42 (m, 1H), 2.33 – 2.19 (m, 2H), 2.08 (d, J = 14.1 Hz, 1H), 1.89 – 1.78 (m, 2H), 1.67 (d, *J* = 12.0 Hz, 3H), 1.48 – 1.28 (m, 3H).
^{13}C NMR (101 MHz, $CDCl_3$) δ 173.4, 171.0, 122.6 (t, *J* = 243.0 Hz), 63.6 (t, *J* = 33.4 Hz), 63.4, 52.7, 41.6 (dd, *J* = 7.0, 2.5 Hz), 44.3, 33.0 (t, *J* = 25.3 Hz), 31.2, 24.6, 21.8, 21.7.
^{19}F NMR (376 MHz, $CDCl_3$) δ -103.2 (d, *J* = 253.2 Hz, 1F), -108.6 (d, *J* = 253.1 Hz, 1F).
HRMS (ESI-TOF) m/z: [M+H]+ Calcd. for $C_{14}H_{22}F_2NO_4$ 306.1511; found: 306.1521.

2,2-difluoro-3-(1-(1-(hydroxymethyl)cyclohexyl)azetidin-3-yl)propan-1-ol (8)

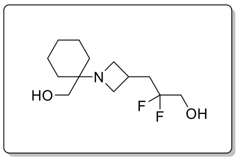

66.2 mg, colorless oil, yield: 84%. Eluent: pentane/ethyl acetate = 1/1.
^{1}H NMR (400 MHz, $CDCl_3$) δ 4.16 (s, 4H), 3.86 – 3.59 (m, 4H), 3.49 – 3.39 (m, 2H), 2.80 (dd, J = 11.4, 3.9 Hz, 1H), 2.68 (dd, *J* = 11.3, 7.7 Hz, 1H), 2.14 – 2.03 (m, 1H), 2.01 – 1.88 (m, 2H), 1.54 – 1.38 (m, 8H).
^{13}C NMR (101 MHz, $CDCl_3$) δ 123.8 (t, *J* = 242.9 Hz), 67.2, 64.4, 63.8 (t, *J* = 32.6 Hz), 56.0, 45.0, 34.4, 32.9 (t, *J* = 24.1 Hz), 32.0, 31.6, 25.8, 21.5, 21.4.
^{19}F NMR (376 MHz, $CDCl_3$) δ -104.0 (d, *J* = 250.0 Hz, 1F), -105.9 (d, *J* = 250.0 Hz, 1F).
HRMS (ESI-TOF) m/z: [M+H]+ Calcd. for $C_{13}H_{24}F_2NO_2$ 264.1770; found: 264.1774.

Ethyl 3-(1-(4-(diphenoxyphosphoryl)phenyl)-2-oxoazetidin-3-yl)-2,2-difluoropropanoate (11)

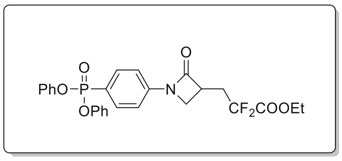

78.3 mg, colorless oil, yield: 76%. Eluent: pentane/ethyl acetate = 3/1.

^{1}H NMR (400 MHz, CDCl$_3$) δ 7.95 – 7.89 (m, 2H), 7.46 – 7.39 (m, 2H), 7.28 (t, *J* = 7.9 Hz, 4H), 7.21 – 7.10 (m, 6H), 4.35 (q, *J* = 7.1 Hz, 2H), 3.90 (t, *J* = 6.0 Hz, 1H), 3.69 – 3.59 (m, 1H), 3.59 – 3.52 (m, 1H), 2.84 – 2.66 (m, 1H), 2.53 – 2.35 (m, 1H), 1.36 (t, *J* = 7.1 Hz, 3H).
^{13}C NMR (101 MHz, CDCl$_3$) δ 165.2, 163.4 (t, *J* = 32.2 Hz), 150.3 (d, *J* = 7.4 Hz), 141.8 (d, *J* = 3.6 Hz), 133.9 (d, *J* = 11.3 Hz), 129.8, 125.2 (d, *J* = 0.9 Hz), 121.7 (d, *J* = 197.9 Hz), 120.6 (d, *J* = 4.5 Hz), 116.2 (d, *J* = 16.3 Hz), 115.0 (t, *J* = 251.3 Hz), 63.4, 45.2, 42.8 (t, *J* = 3.3 Hz), 33.6 (t, *J* = 24.0 Hz), 13.9.
^{19}F NMR (376 MHz, CDCl$_3$) δ -104.4 (d, *J* = 263.1 Hz, 1F), -106.7 (d, *J* = 263.2 Hz, 1F).
^{31}P NMR (162 MHz, CDCl$_3$) δ 11.3.
HRMS (ESI-TOF) m/z: [M+H]+ Calcd. for $C_{26}H_{25}F_2NO_6P$ 516.1382; found: 516.1374.

Ethyl-2,2-difluoro-3-(2-oxo-1-phenylpyrrolidin-3-yl)propanoate (14)

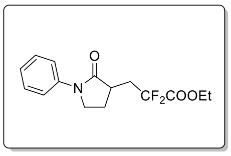

36.5 mg, colorless oil, yield: 62%. Eluent: pentane/ethyl acetate = 10/1.
^{1}H NMR (400 MHz, CDCl$_3$) δ 7.61 (d, *J* = 7.9 Hz, 2H), 7.37 (t, *J* = 8.0 Hz, 2H), 7.16 (t, *J* = 7.4 Hz, 1H), 4.35 (q, *J* = 7.1 Hz, 2H), 3.88 – 3.74 (m, 2H), 3.02 – 2.86 (m, 2H), 2.59 – 2.50 (m, 1H), 2.22 – 2.05 (m, 1H), 2.01 – 1.89 (m, 1H), 1.37 (t, *J* = 7.1 Hz, 3H).
^{13}C NMR (101 MHz, CDCl$_3$) δ 173.4, 163.9 (t, *J* = 32.5 Hz), 139.2, 128.9, 124.8, 119.8, 115.9 (t, *J* = 250.5 Hz), 63.1, 46.8, 38.0, 36.1 (t, *J* = 23.0 Hz), 26.2, 14.0.
^{19}F NMR (376 MHz, CDCl$_3$) δ -103.5 (d, *J* = 259.2 Hz, 1F), -107.1 (d, *J* = 259.1 Hz, 1F).
HRMS (ESI-TOF) m/z: [M+H]+ Calcd. for $C_{15}H_{18}F_2NO_3$ 298.1249; found: 298.1252.

Ethyl-2,2-difluoro-5-oxo-5-(phenylamino)pentanoate (16)

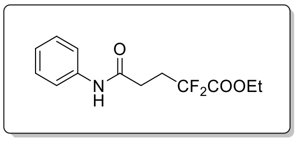

38.1 mg, colorless oil, yield: 70%. Eluent: pentane/ethyl acetate = 5/1.
^{1}H NMR (400 MHz, CDCl$_3$) δ 7.94 (s, 1H), 7.46 (d, *J* = 7.9 Hz, 2H), 7.33 – 7.21 (m, 2H), 7.08 (t, *J* = 7.2 Hz, 1H), 4.29 (q, *J* = 7.1 Hz, 2H), 2.59 – 2.42 (m, 4H), 1.31 (t, *J* = 7.1 Hz, 3H).
^{13}C NMR (101 MHz, CDCl$_3$) δ 169.1, 164.0 (t, *J* = 32.7 Hz), 137.7, 129.0, 124.5, 120.2, 115.6 (t, *J* = 250.3 Hz), 63.2, 30.0 (t, *J* = 23.8 Hz), 29.1, 13.9.
^{19}F NMR (376 MHz, CDCl$_3$) δ -106.5 (s, 2F).
HRMS (ESI-TOF) m/z: [M+H]+ Calcd. for $C_{13}H_{16}F_2NO_3$ 272.1093; found: 272.1097.

9. Copy of NMR Spectrum.

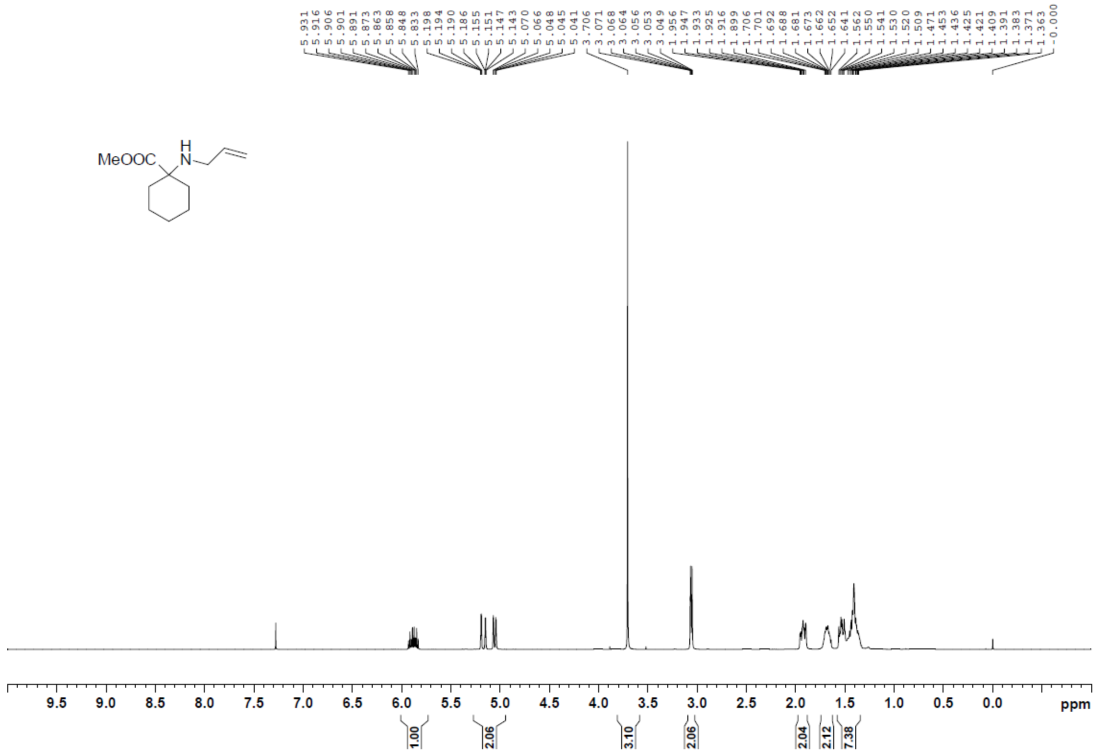

Supplementary Figure 3. ^{1}H NMR spectrum of **1a** in $CDCl_3$ (400 MHz)

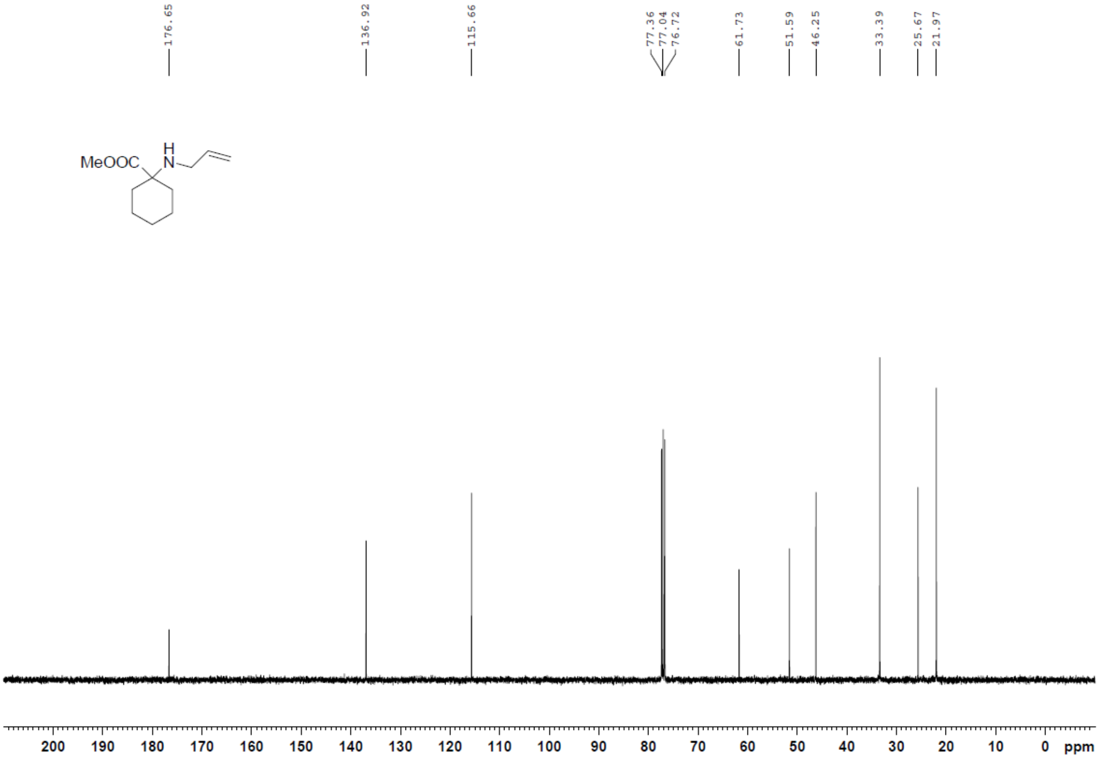

Supplementary Figure 4. ^{13}C NMR spectrum of **1a** in $CDCl_3$ (101 MHz)

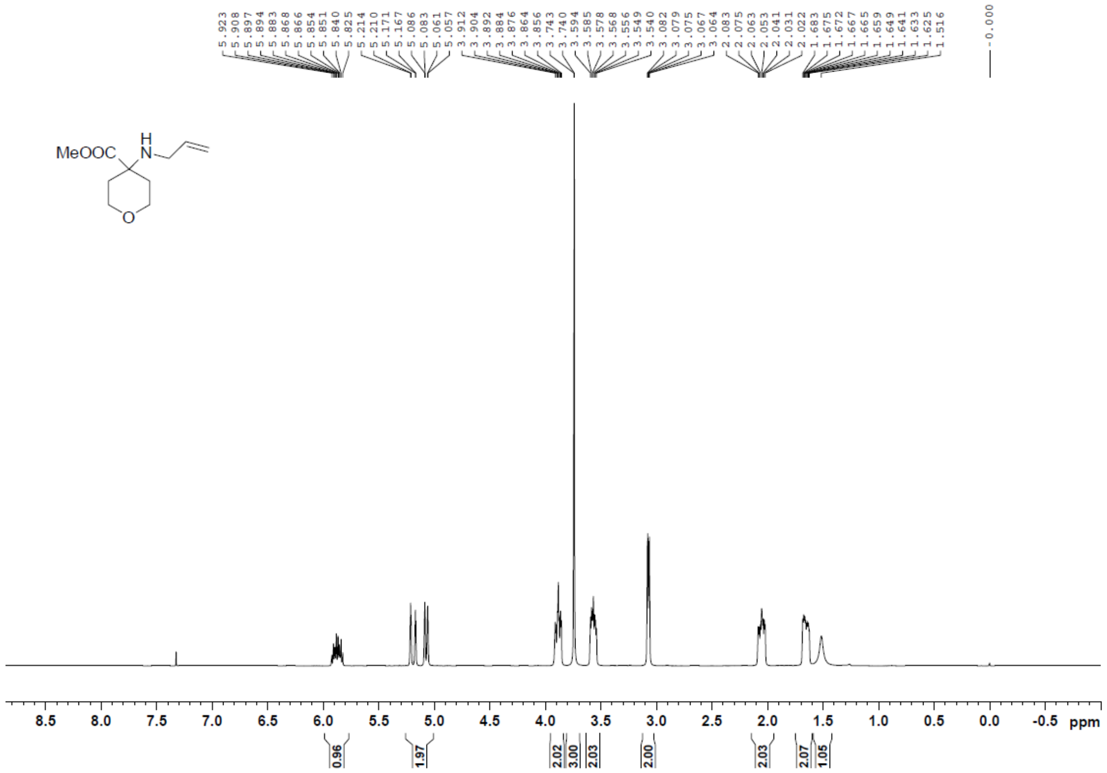

Supplementary Figure 5. ^{1}H NMR spectrum of **1b** in $CDCl_3$ (400 MHz)

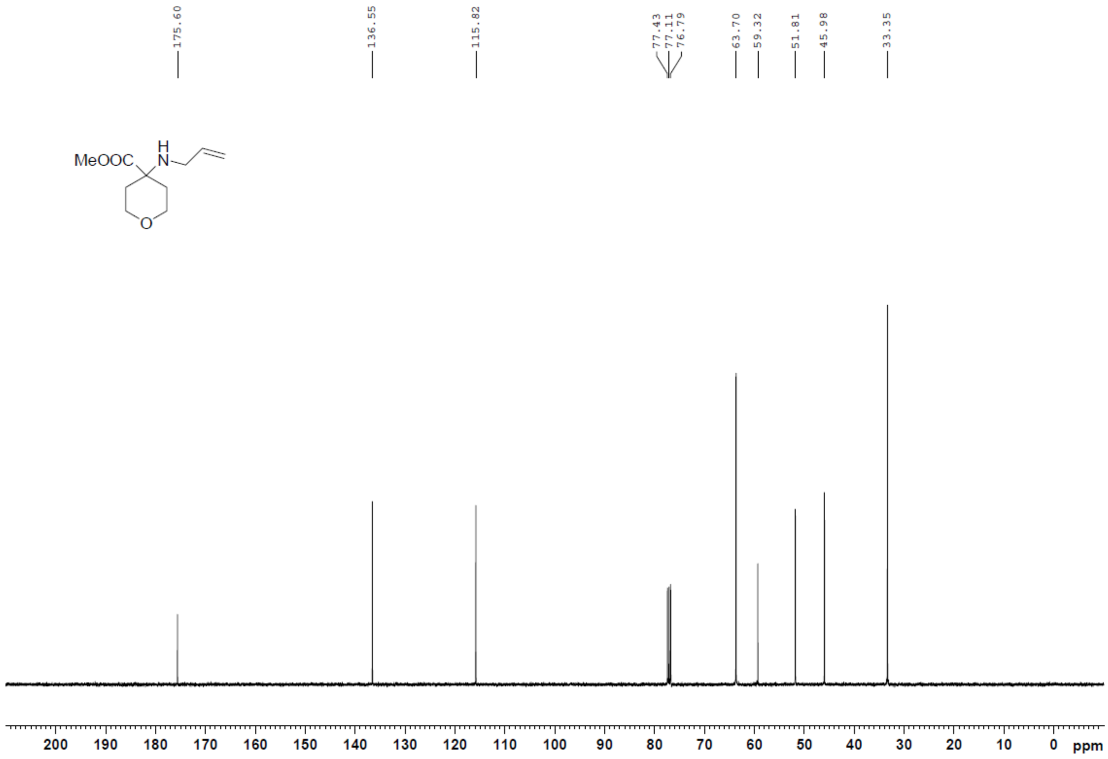

Supplementary Figure 6. ^{13}C NMR spectrum of **1b** in $CDCl_3$ (101 MHz)

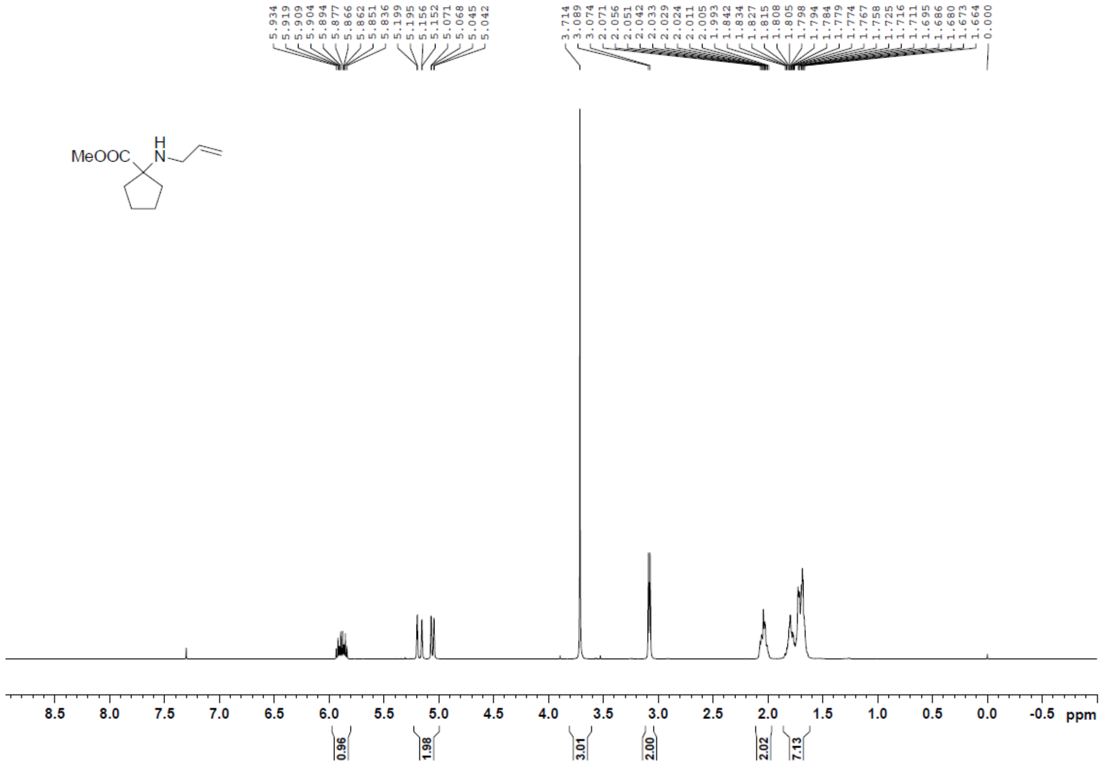

Supplementary Figure 7. ^{1}H NMR spectrum of **1c** in $CDCl_3$ (400 MHz)

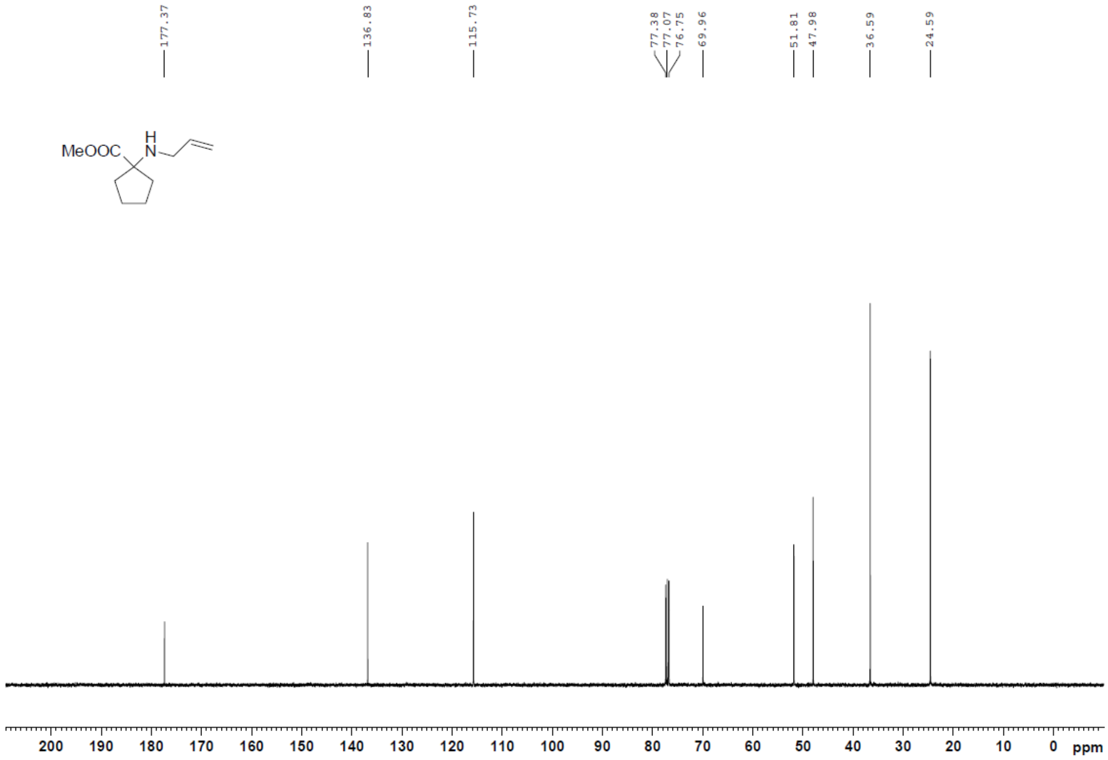

Supplementary Figure 8. ^{13}C NMR spectrum of **1c** in $CDCl_3$ (101 MHz)

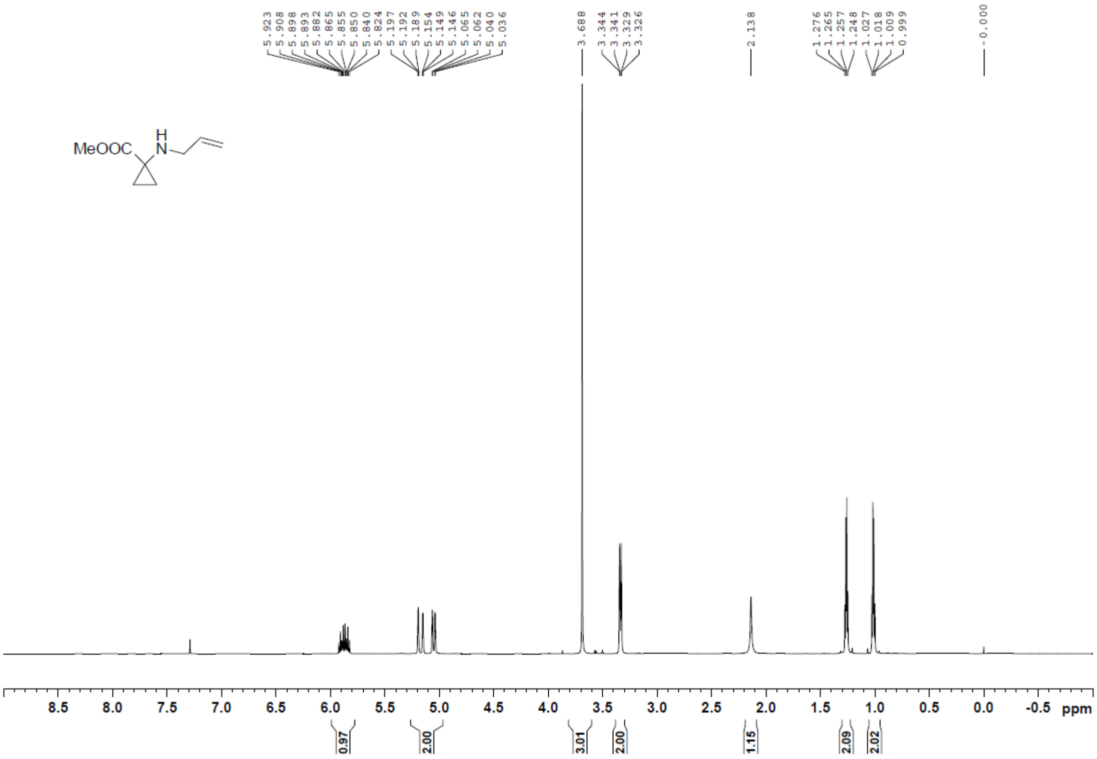

Supplementary Figure 9. ^{1}H NMR spectrum of **1d** in $CDCl_3$ (400 MHz)

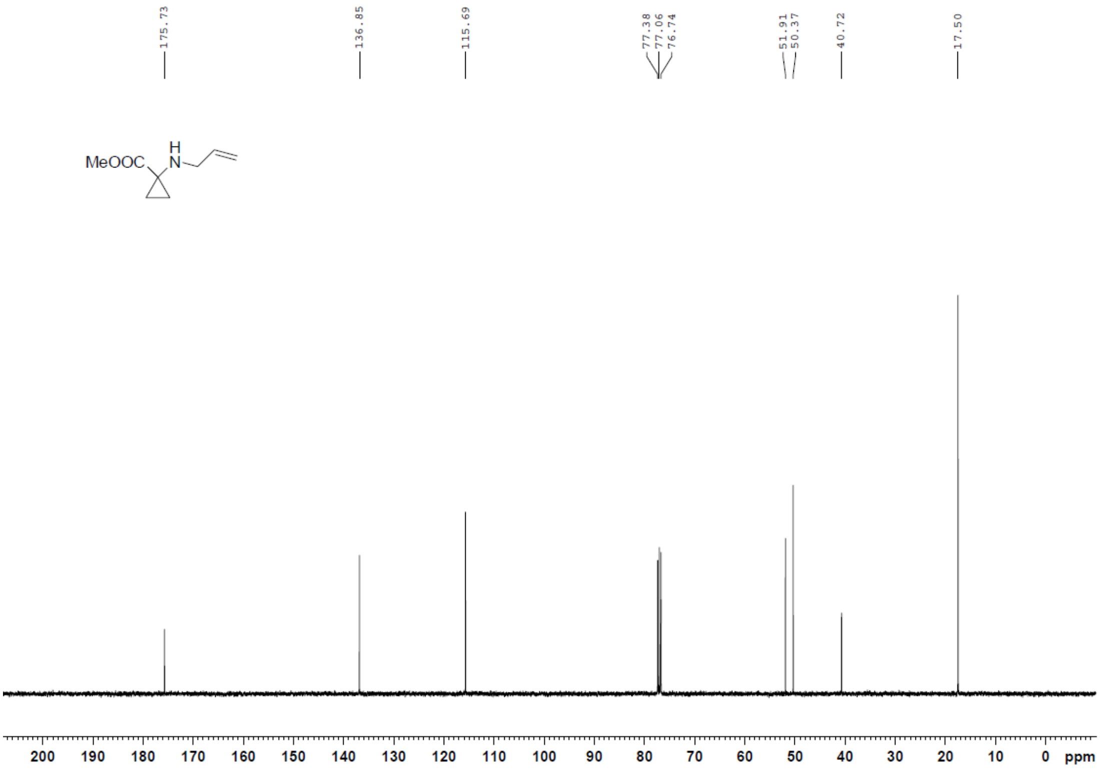

Supplementary Figure 10. ^{13}C NMR spectrum of **1d** in $CDCl_3$ (101 MHz)

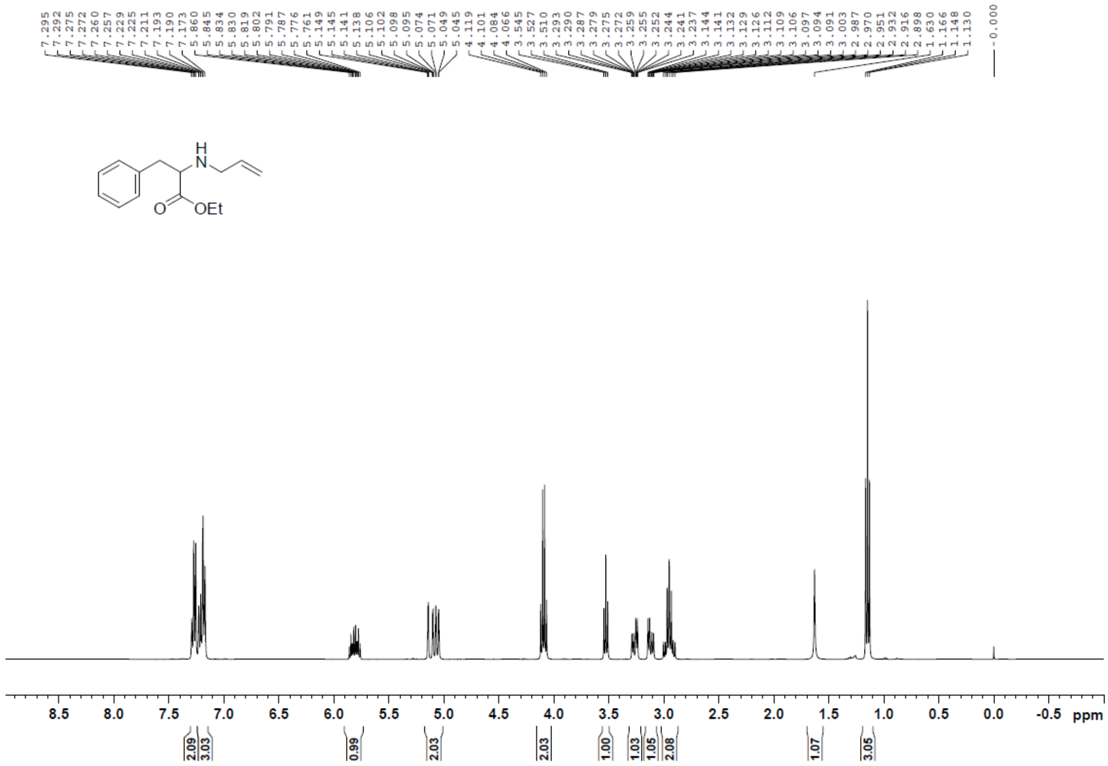

Supplementary Figure 11. ^{1}H NMR spectrum of **1e** in $CDCl_3$ (400 MHz)

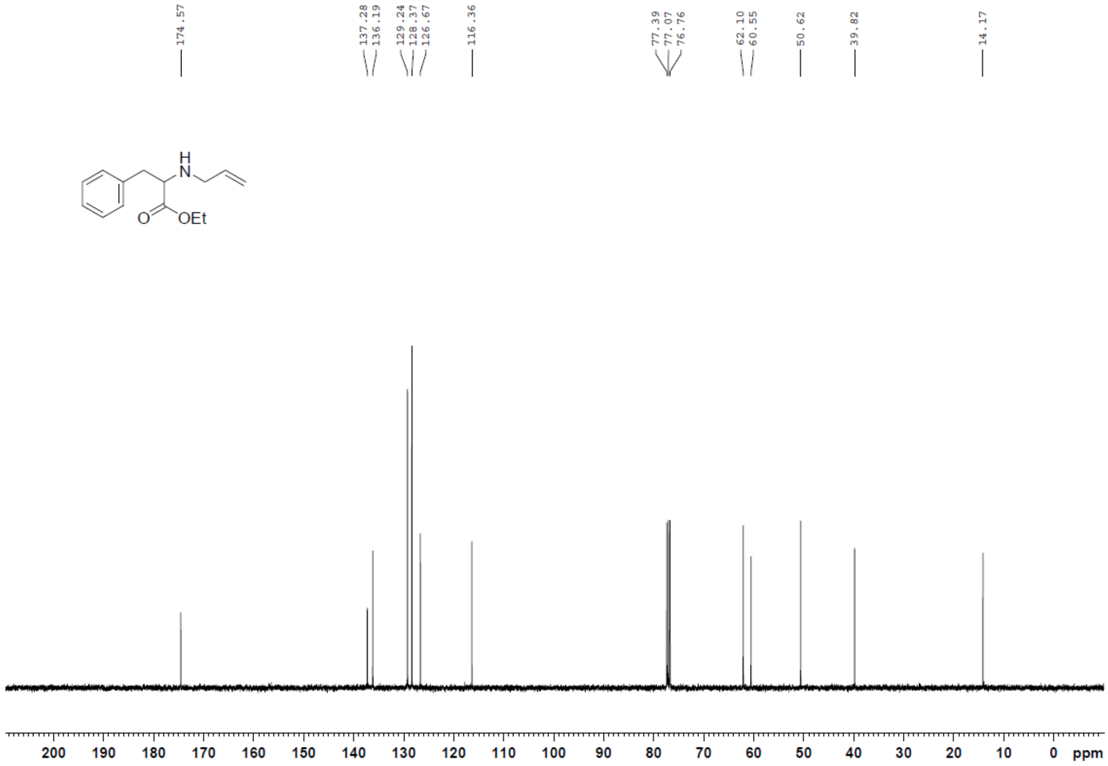

Supplementary Figure 12. ^{13}C NMR spectrum of **1e** in $CDCl_3$ (101 MHz)

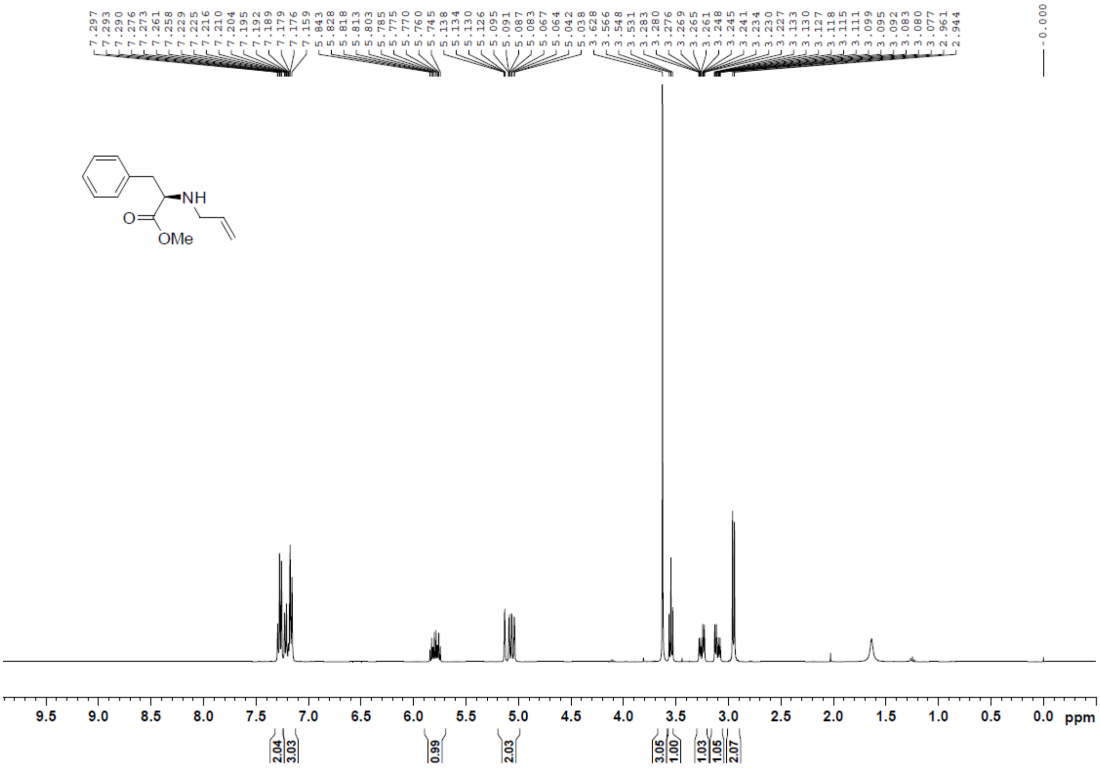

Supplementary Figure 13. ^{1}H NMR spectrum of **1f** in $CDCl_3$ (400 MHz)

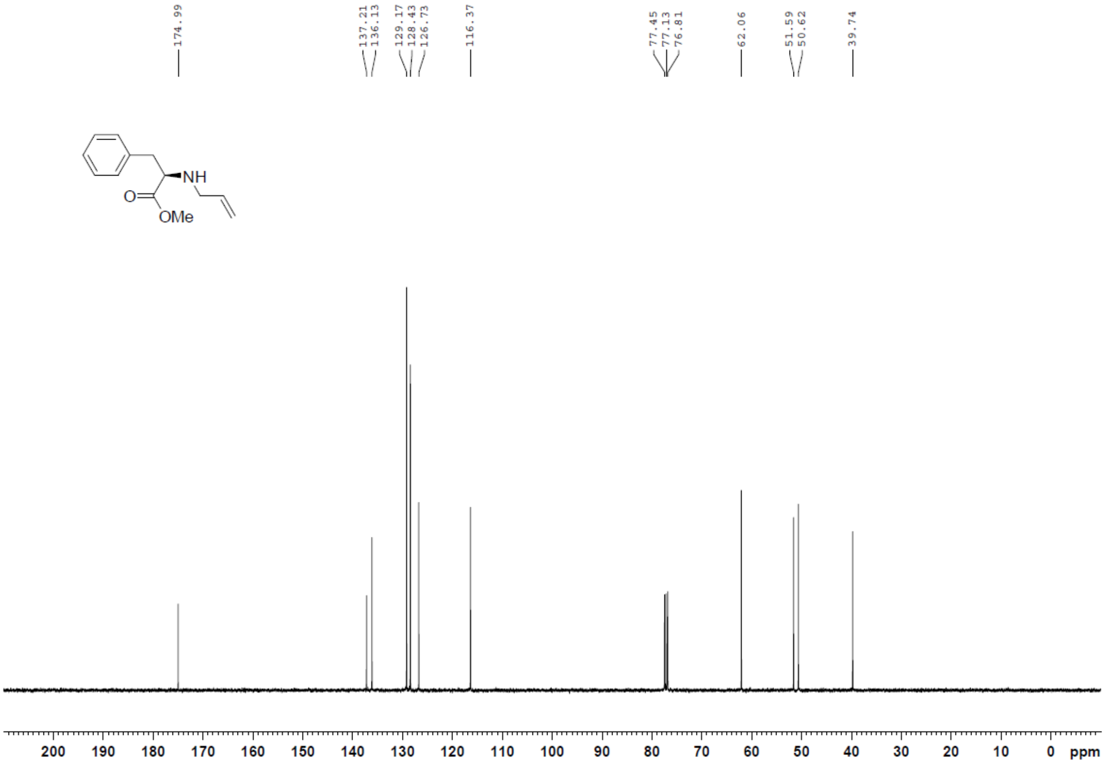

Supplementary Figure 14. ^{13}C NMR spectrum of **1f** in $CDCl_3$ (101 MHz)

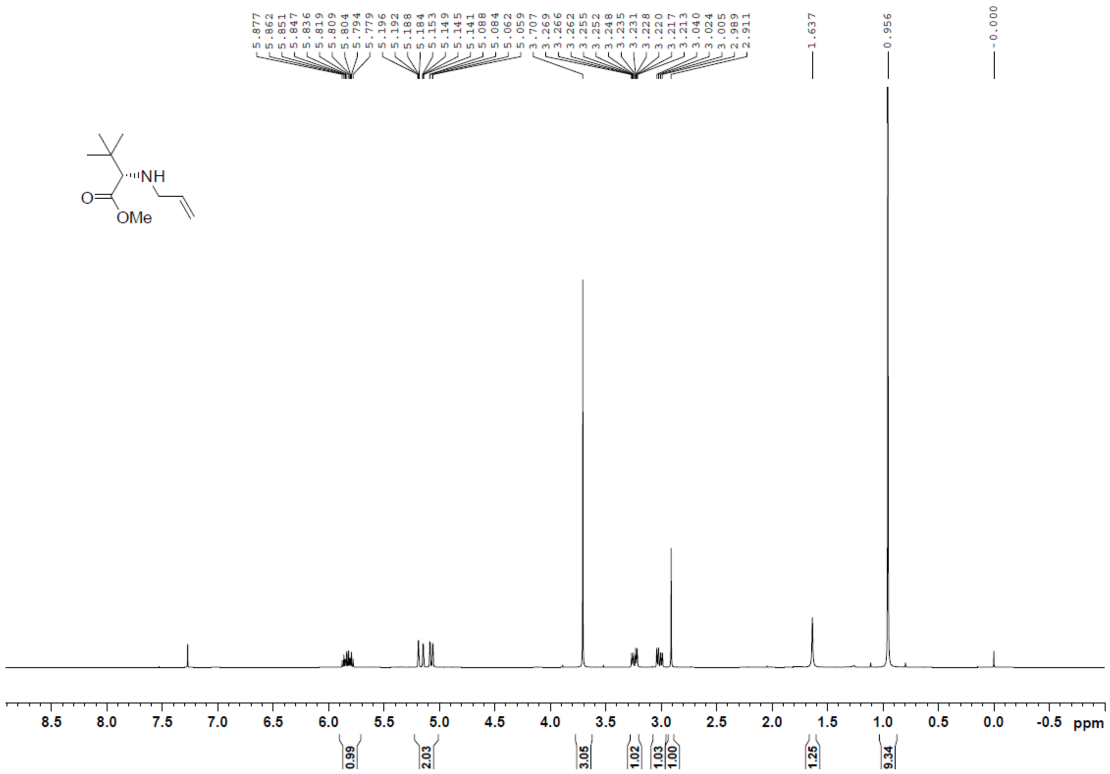

Supplementary Figure 15. ^{1}H NMR spectrum of **1g** in $CDCl_3$ (400 MHz)

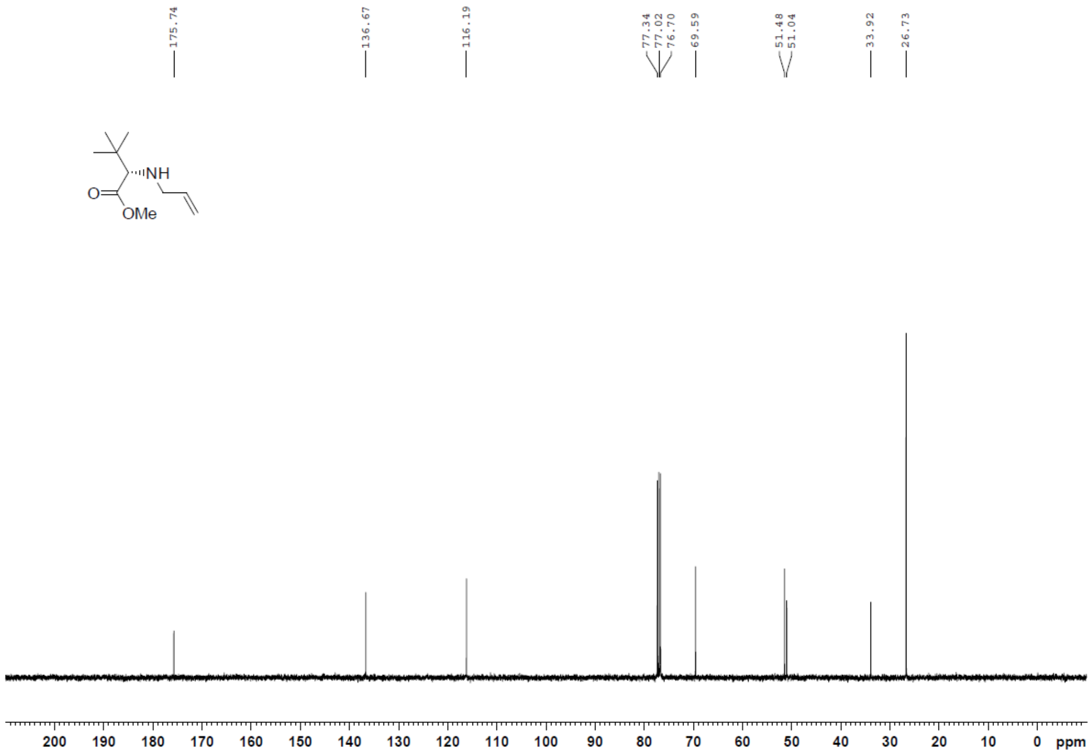

Supplementary Figure 16. ^{13}C NMR spectrum of **1g** in $CDCl_3$ (101 MHz)

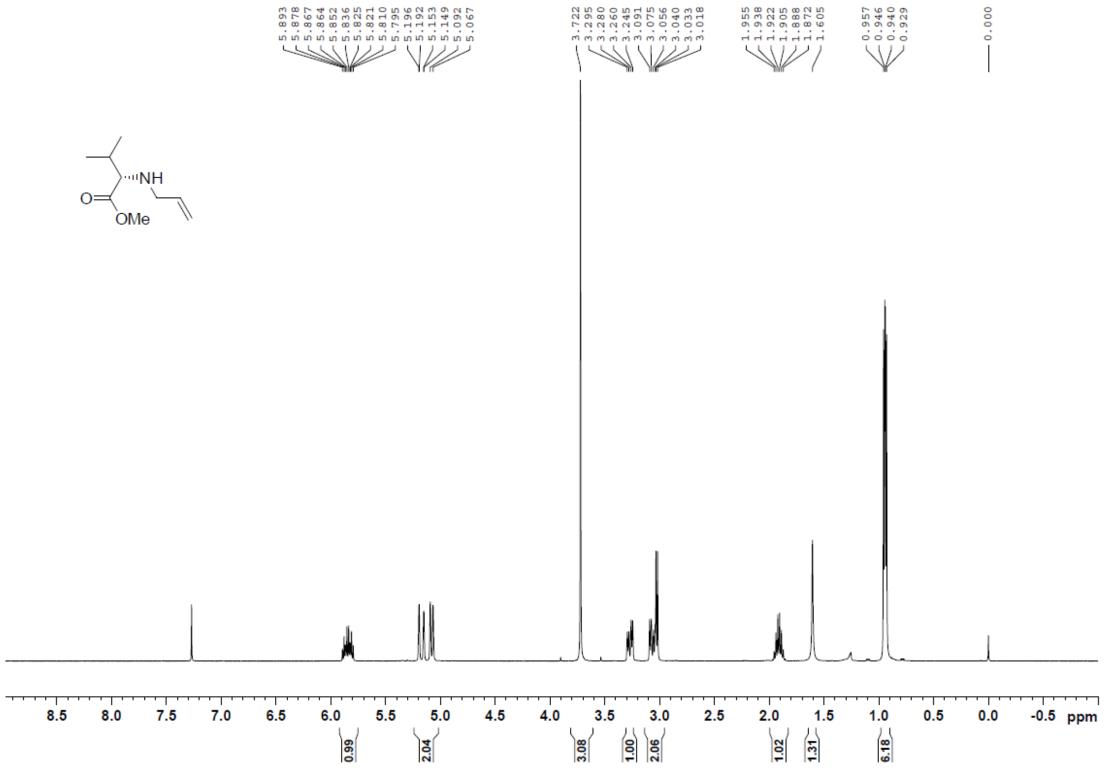

Supplementary Figure 17. ^{1}H NMR spectrum of **1h** in $CDCl_3$ (400 MHz)

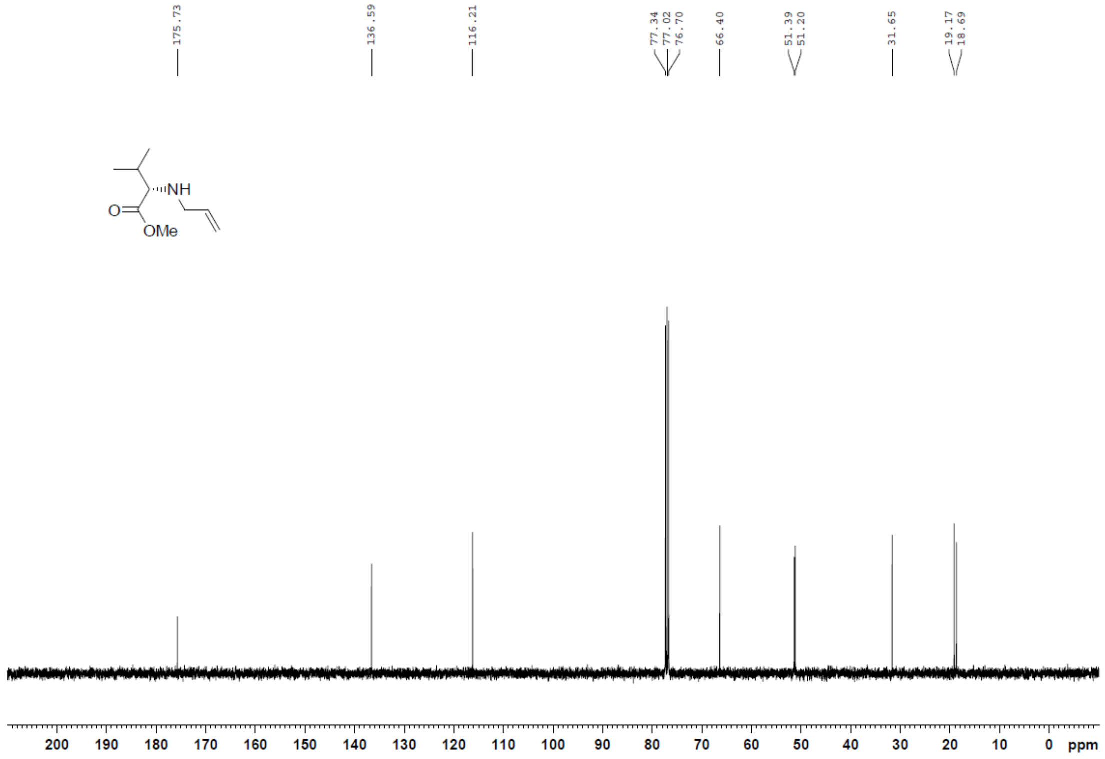

Supplementary Figure 18. ^{13}C NMR spectrum of **1h** in $CDCl_3$ (101 MHz)

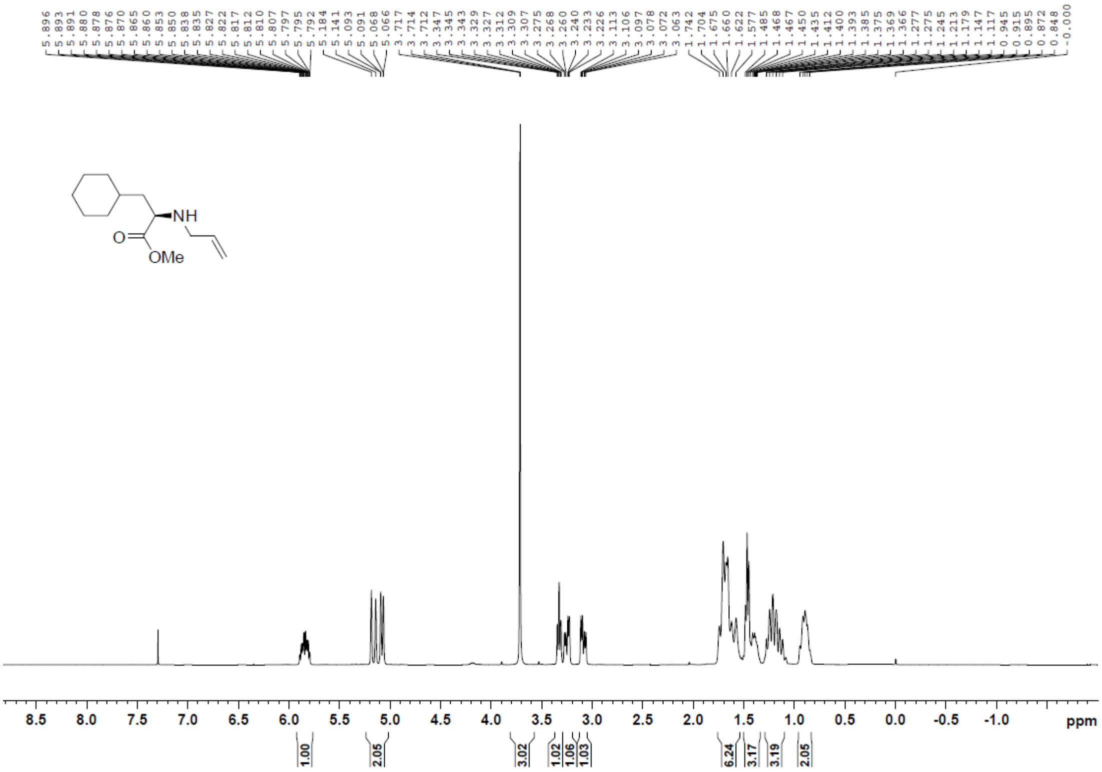

Supplementary Figure 19. ^{1}H NMR spectrum of **1i** in $CDCl_3$ (400 MHz)

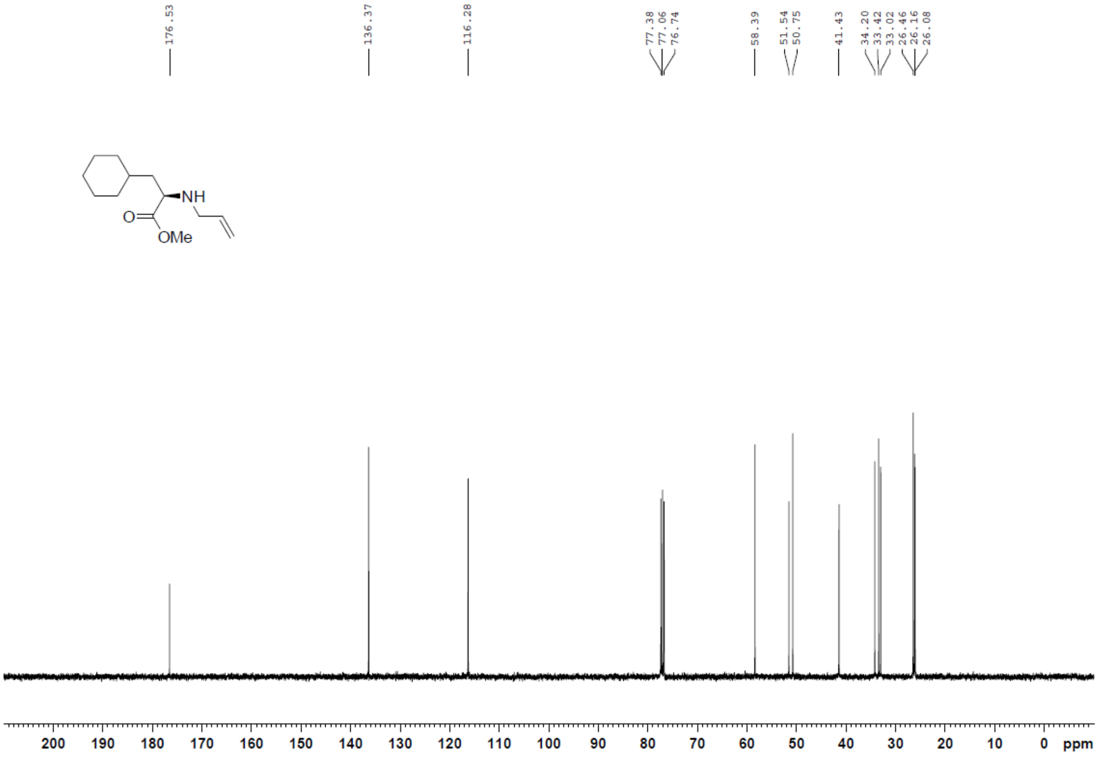

Supplementary Figure 20. ^{13}C NMR spectrum of **1i** in $CDCl_3$ (101 MHz)

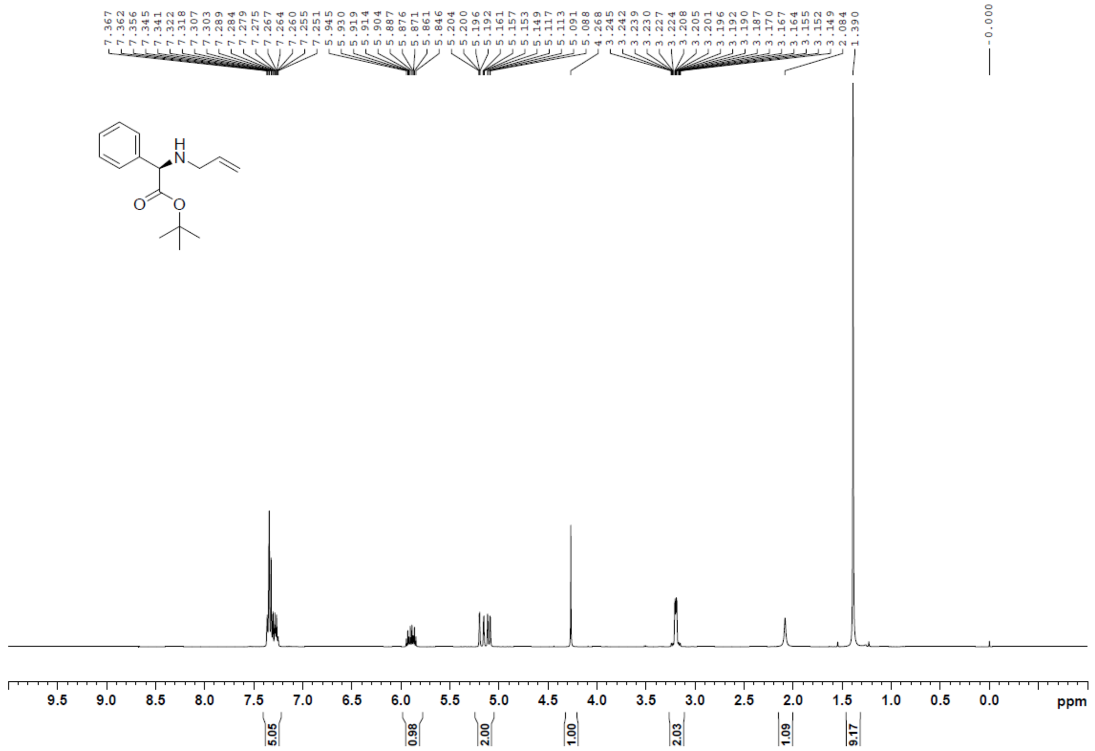

Supplementary Figure 21. ^{1}H NMR spectrum of **1j** in $CDCl_3$ (400 MHz)

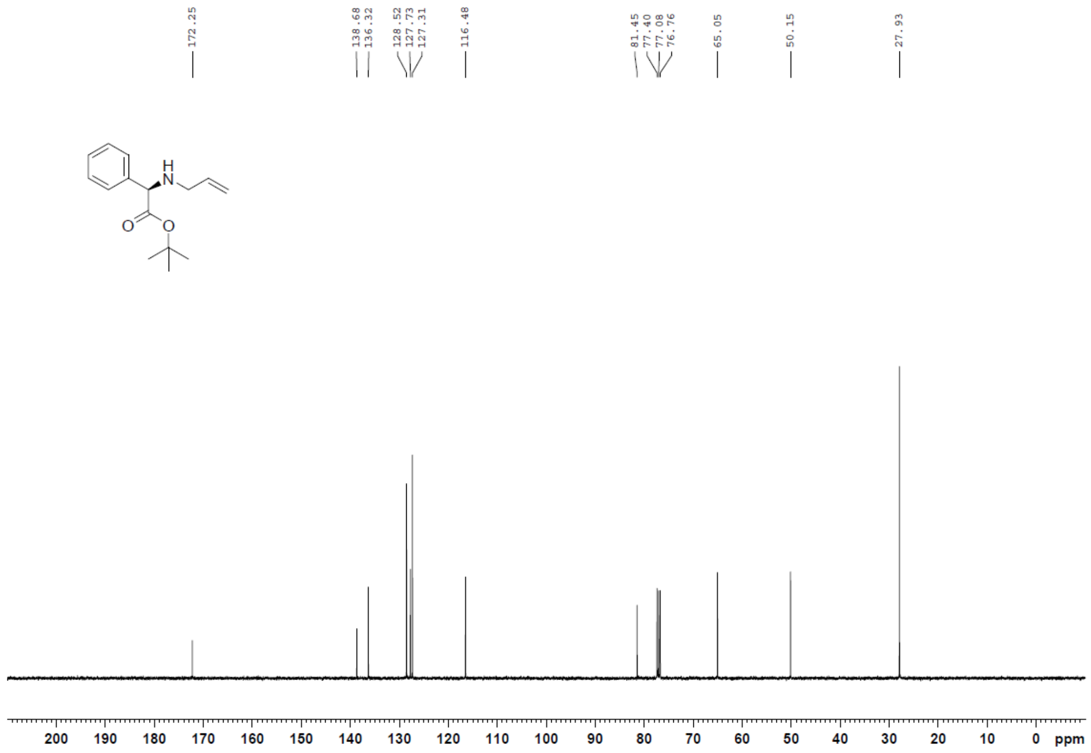

Supplementary Figure 22. ^{13}C NMR spectrum of **1j** in $CDCl_3$ (101 MHz)

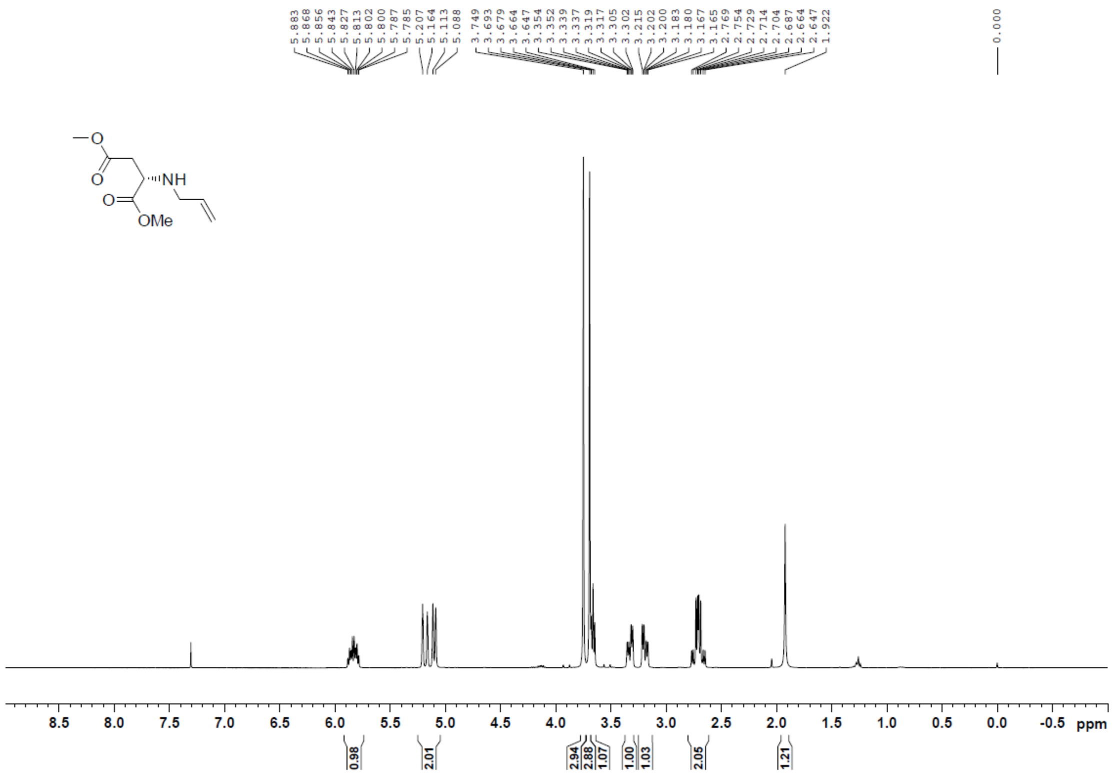

Supplementary Figure 23. ^{1}H NMR spectrum of **1k** in $CDCl_3$ (400 MHz)

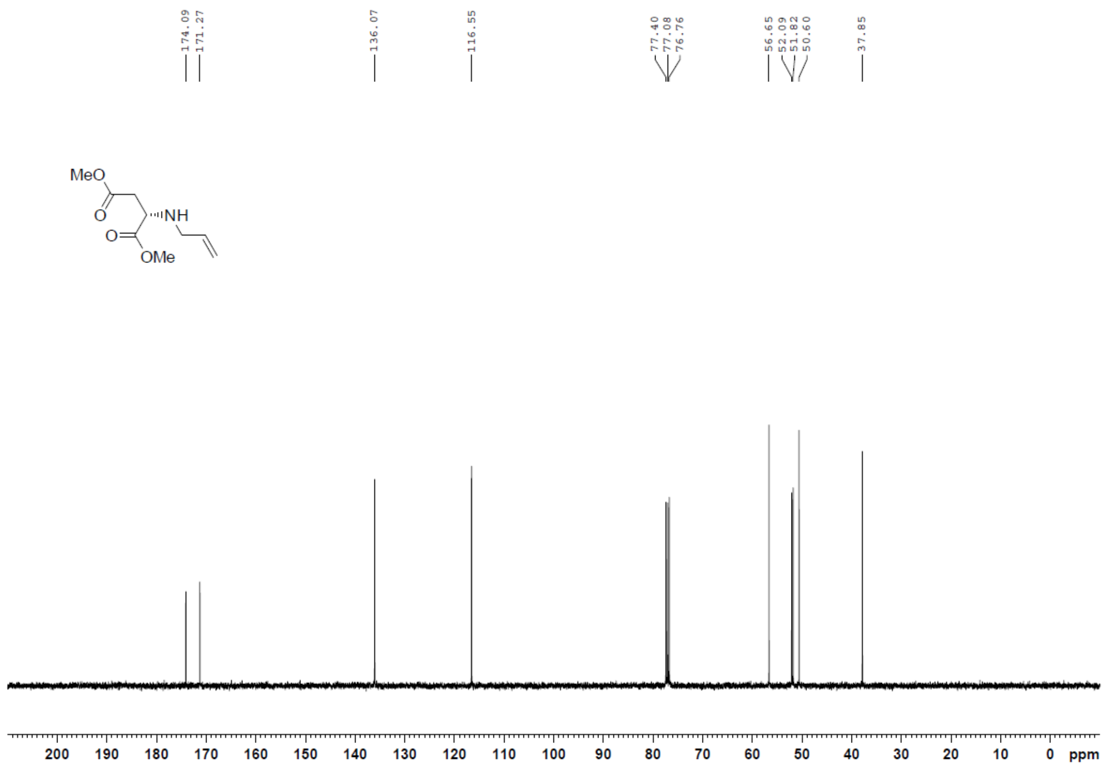

Supplementary Figure 24. ^{13}C NMR spectrum of **1k** in $CDCl_3$ (101 MHz)

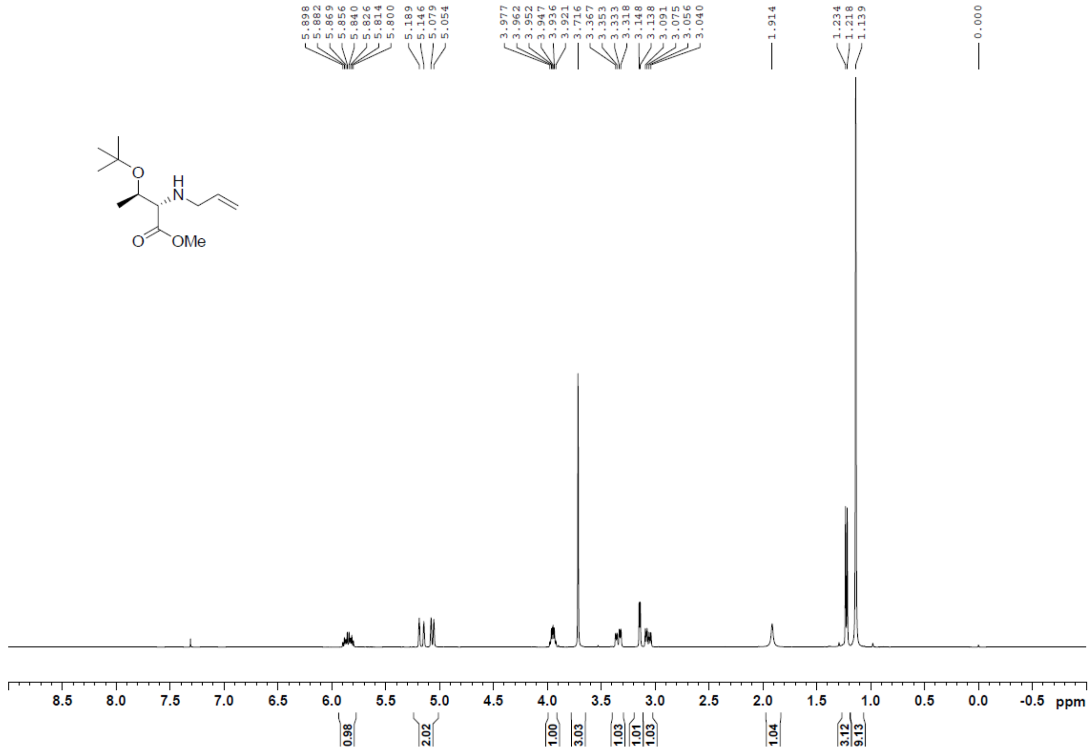

Supplementary Figure 25. ^{1}H NMR spectrum of **1l** in $CDCl_3$ (400 MHz)

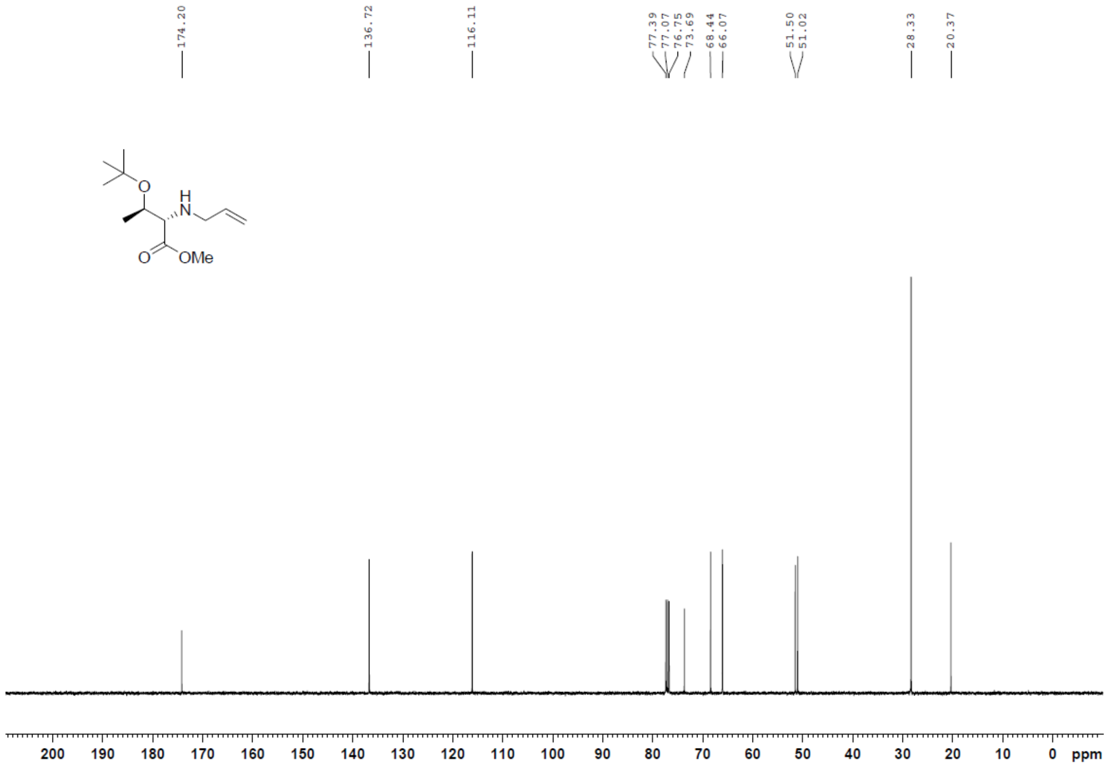

Supplementary Figure 26. ^{13}C NMR spectrum of **1l** in $CDCl_3$ (101 MHz)

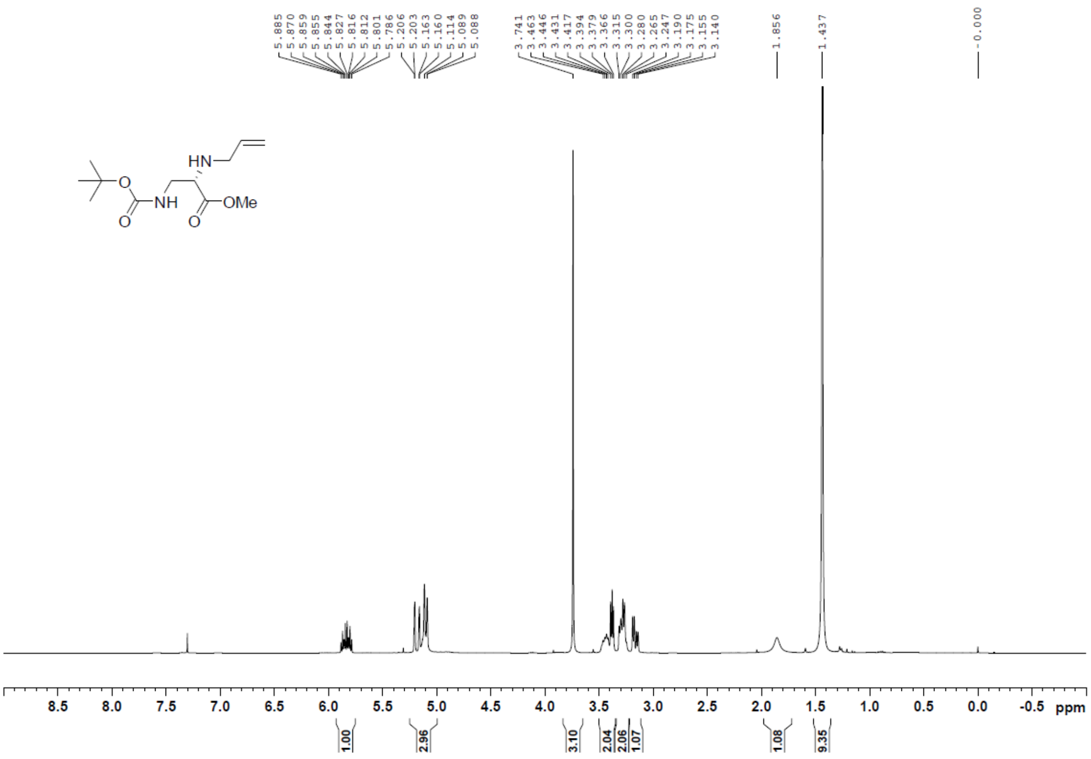

Supplementary Figure 27. ^{1}H NMR spectrum of **1m** in $CDCl_3$ (400 MHz)

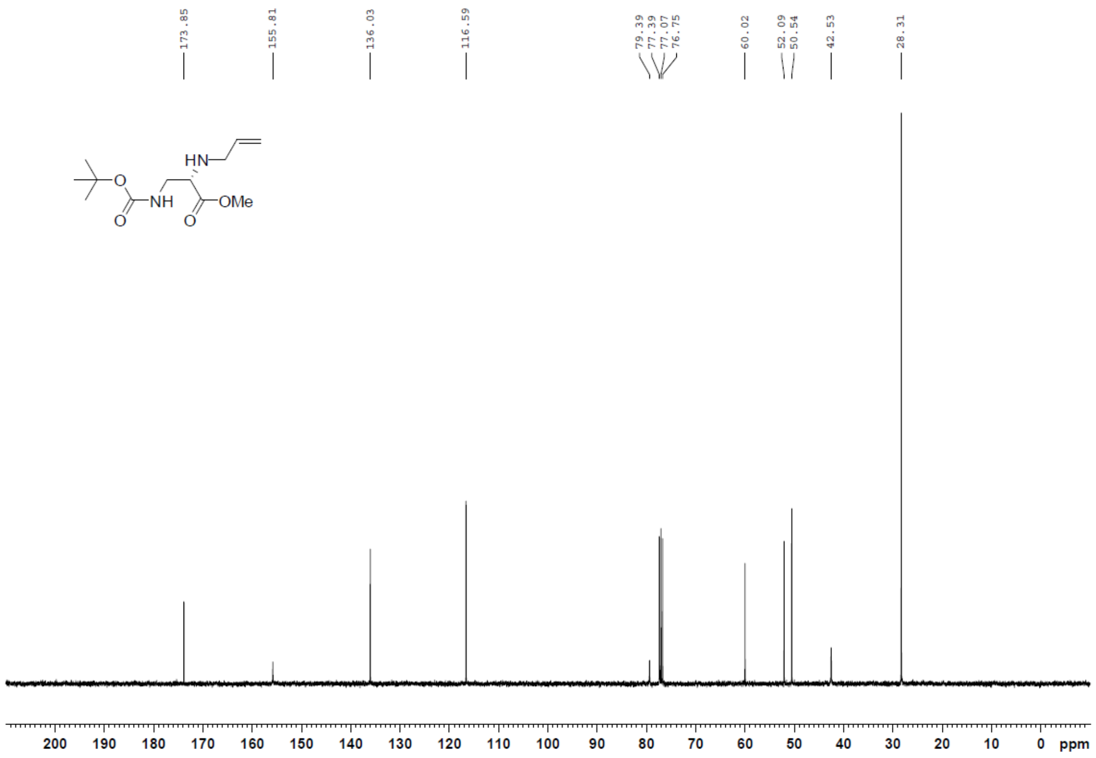

Supplementary Figure 28. ^{13}C NMR spectrum of **1m** in $CDCl_3$ (101 MHz)

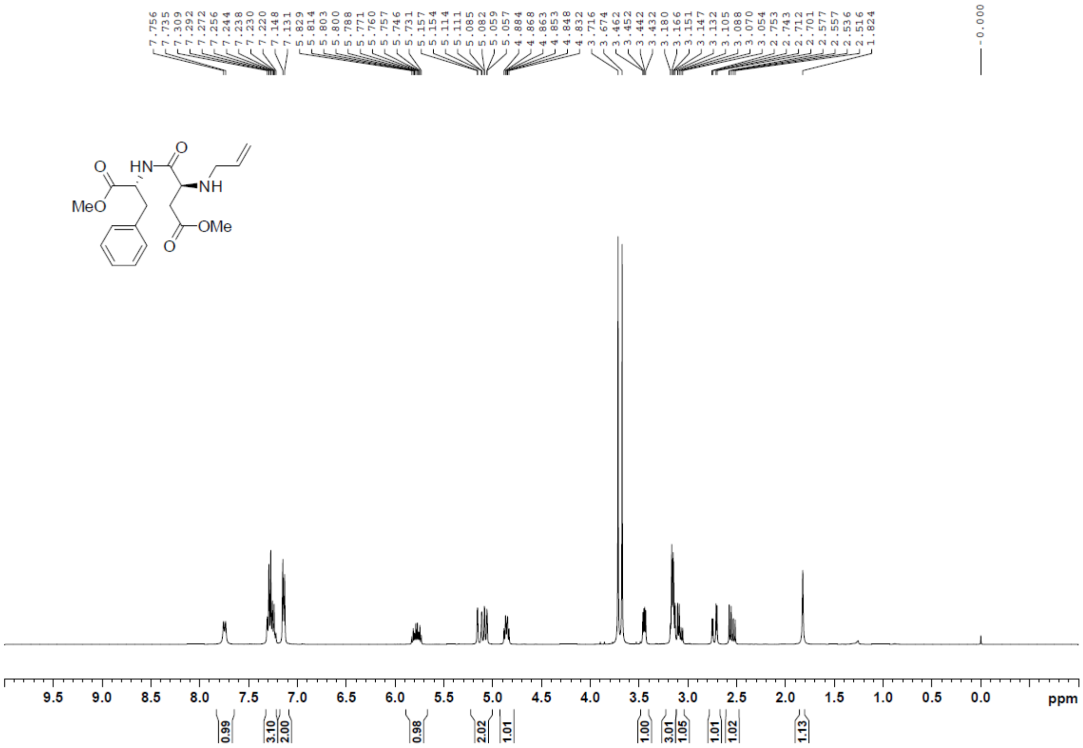

Supplementary Figure 29. ^{1}H NMR spectrum of **1n** in $CDCl_3$ (400 MHz)

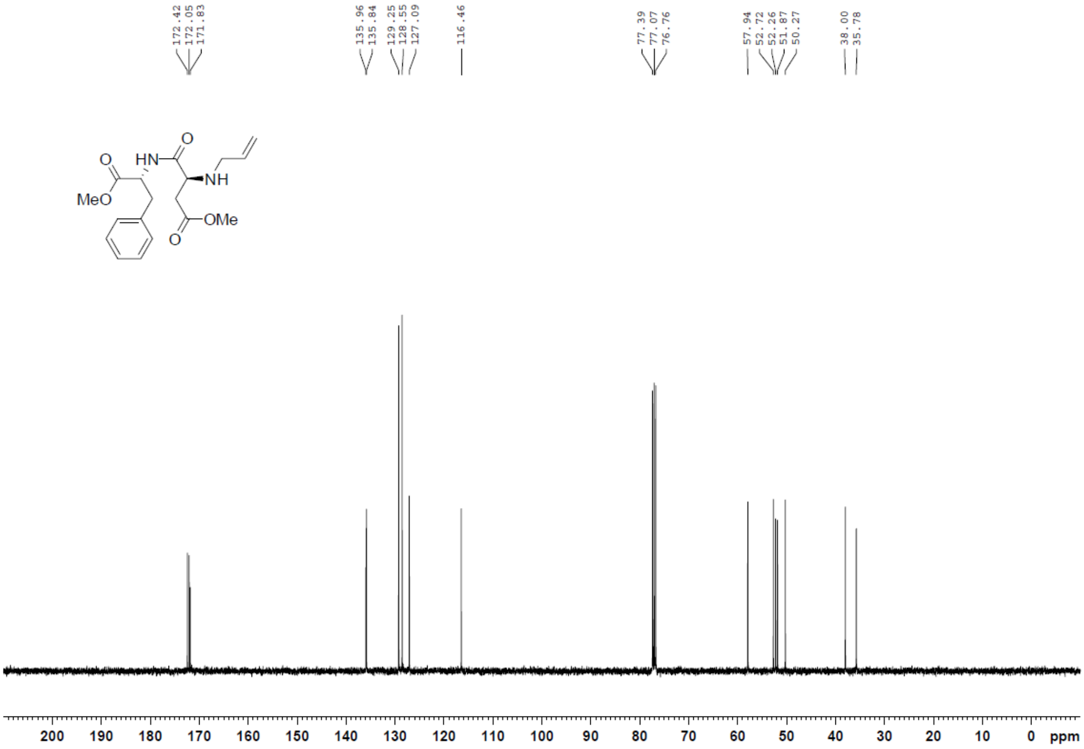

Supplementary Figure 30. ^{13}C NMR spectrum of **1n** in $CDCl_3$ (101 MHz)

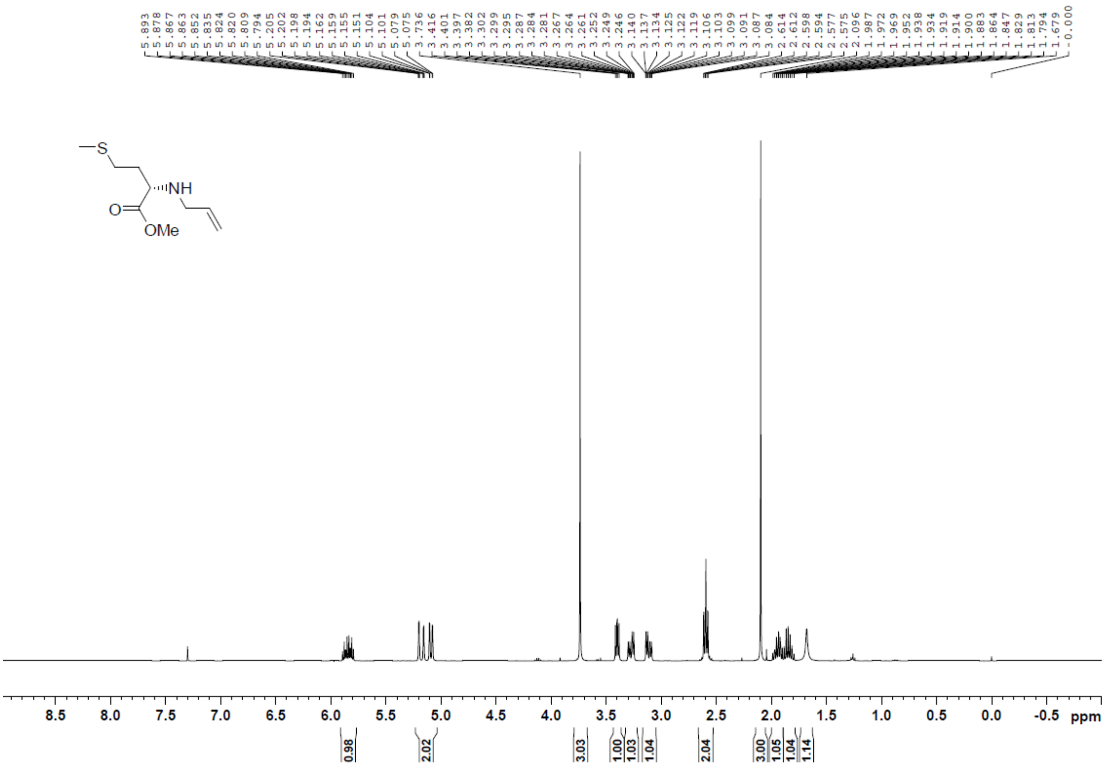

Supplementary Figure 31. ^{1}H NMR spectrum of **1o** in $CDCl_3$ (400 MHz)

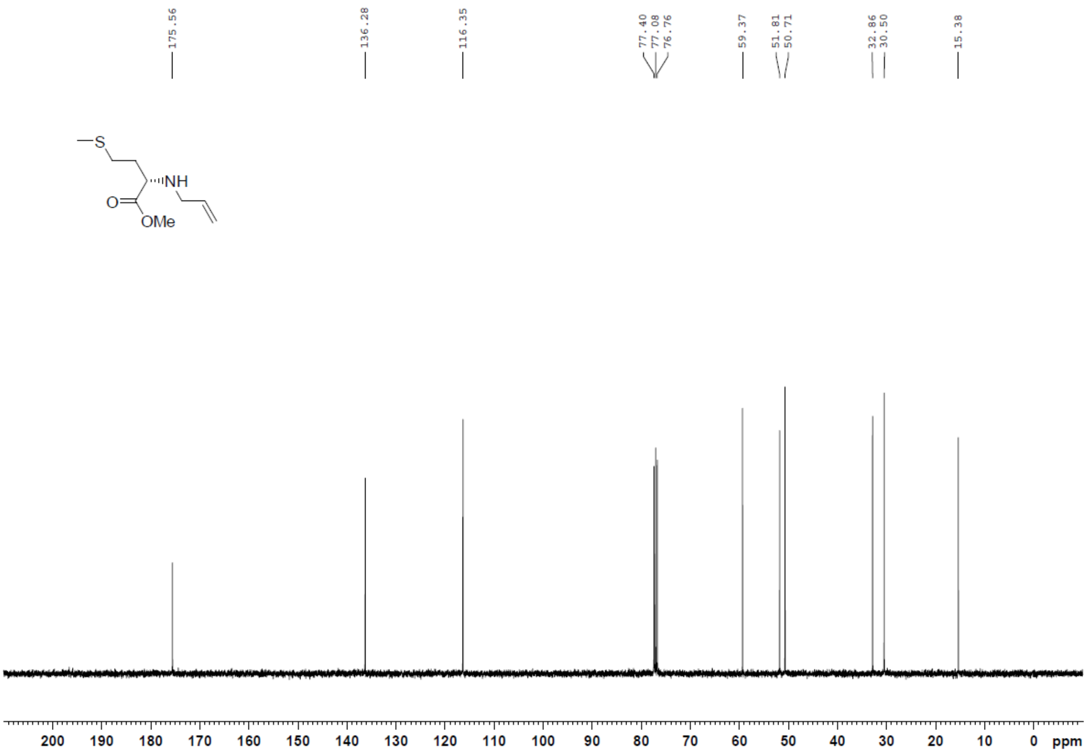

Supplementary Figure 32. ^{13}C NMR spectrum of **1o** in $CDCl_3$ (101 MHz)

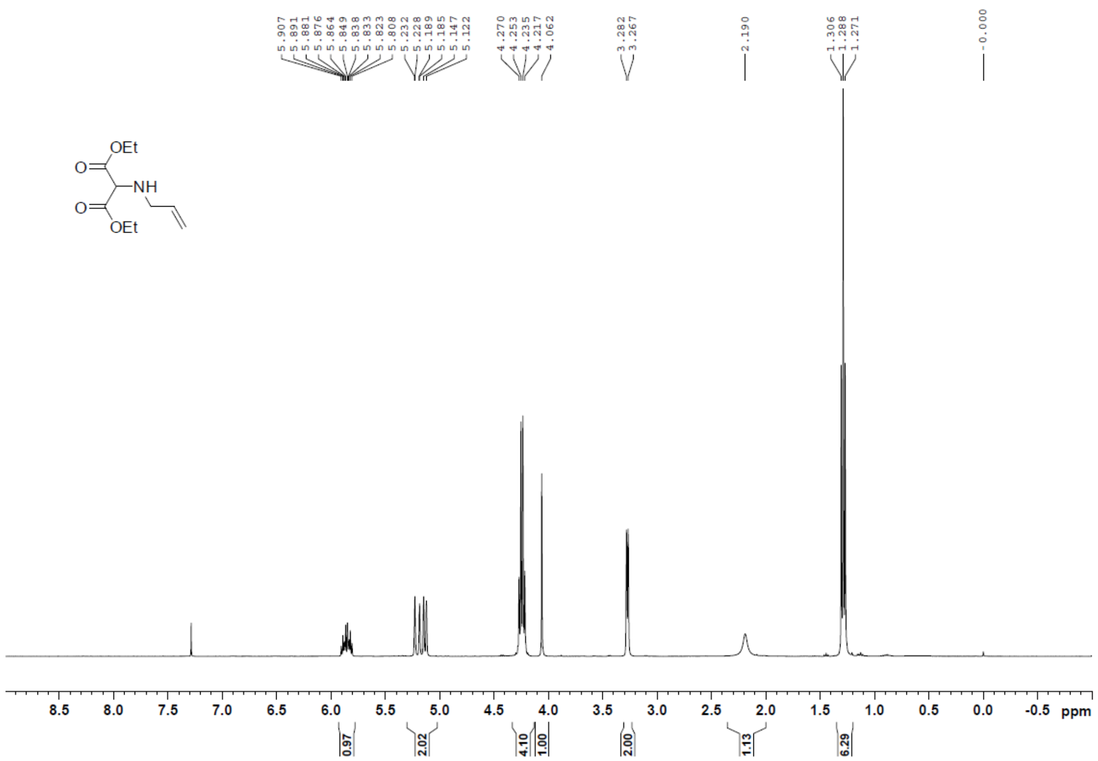

Supplementary Figure 33. ^{1}H NMR spectrum of **1p** in $CDCl_3$ (400 MHz)

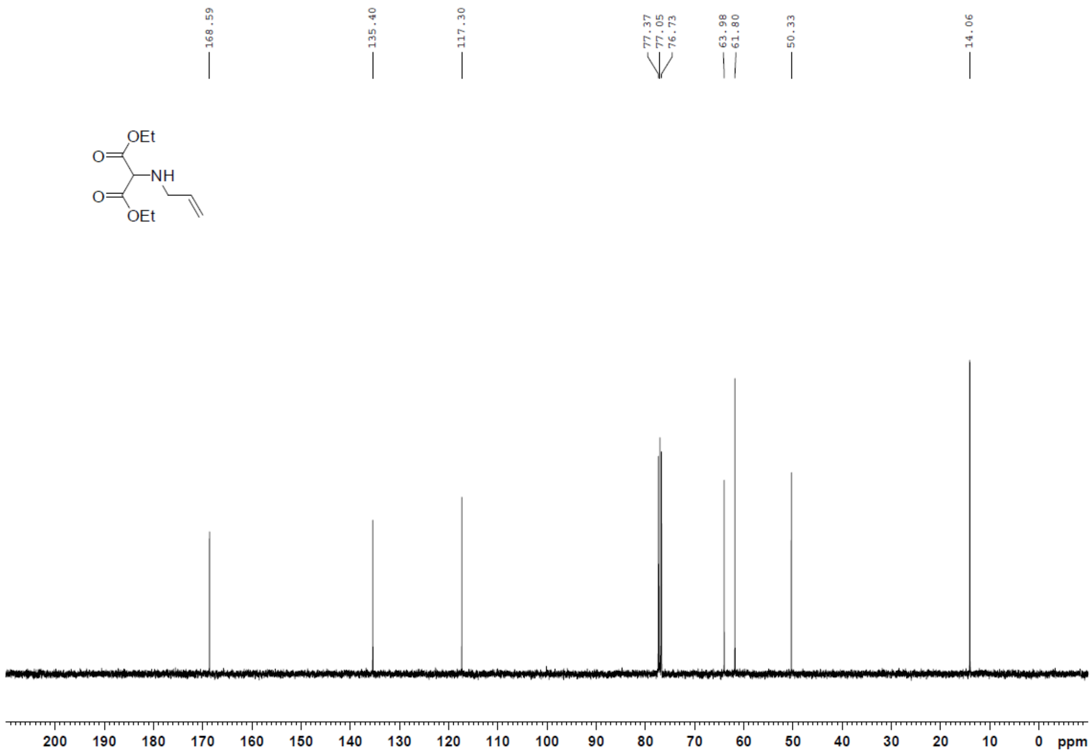

Supplementary Figure 34. ^{13}C NMR spectrum of **1p** in $CDCl_3$ (101 MHz)

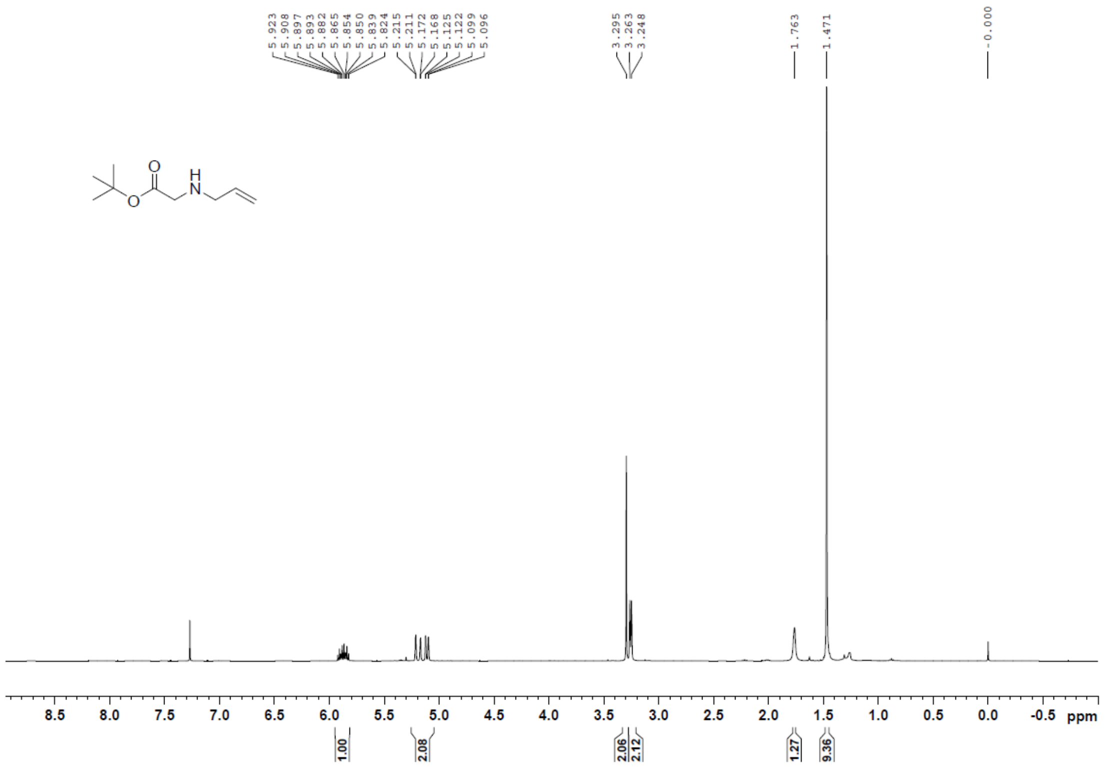

Supplementary Figure 35. ^{1}H NMR spectrum of **1q** in $CDCl_3$ (400 MHz)

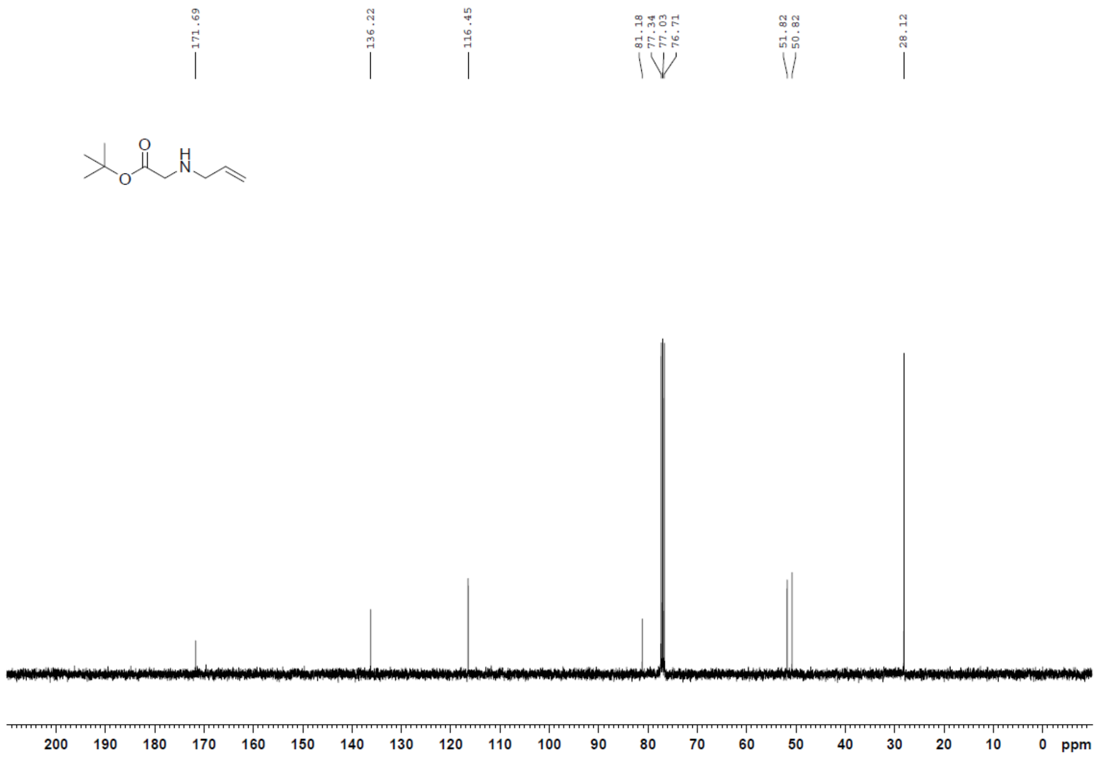

Supplementary Figure 36. ^{13}C NMR spectrum of **1q** in $CDCl_3$ (101 MHz)

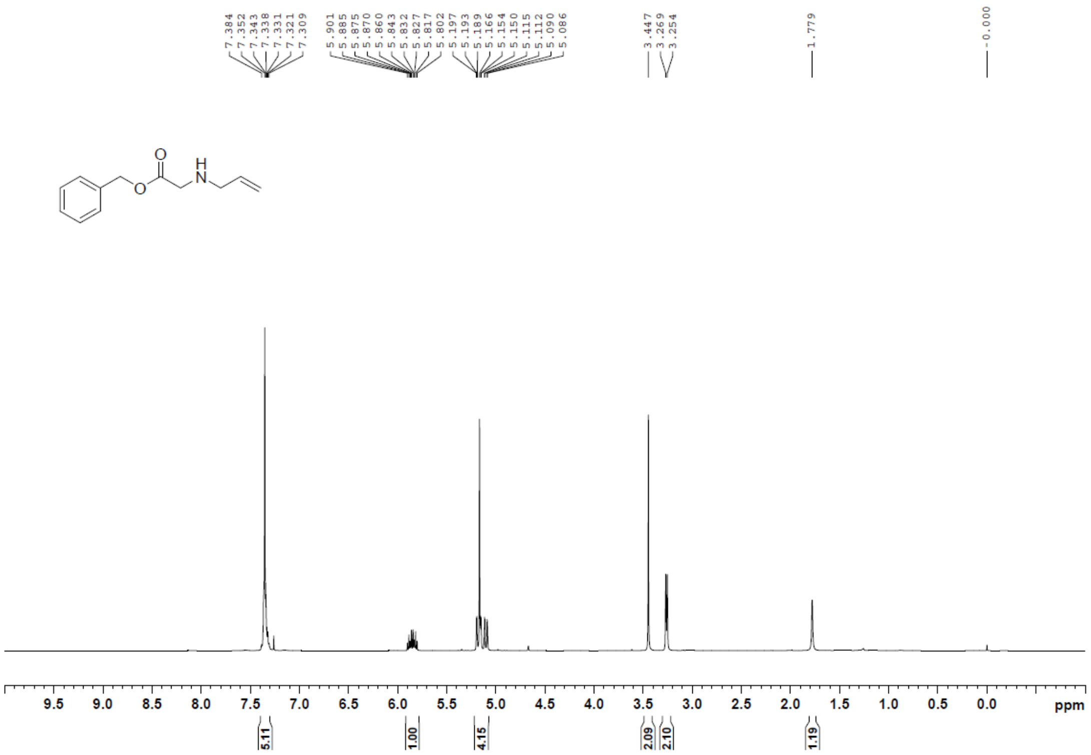

Supplementary Figure 37. ^{1}H NMR spectrum of **1r** in $CDCl_3$ (400 MHz)

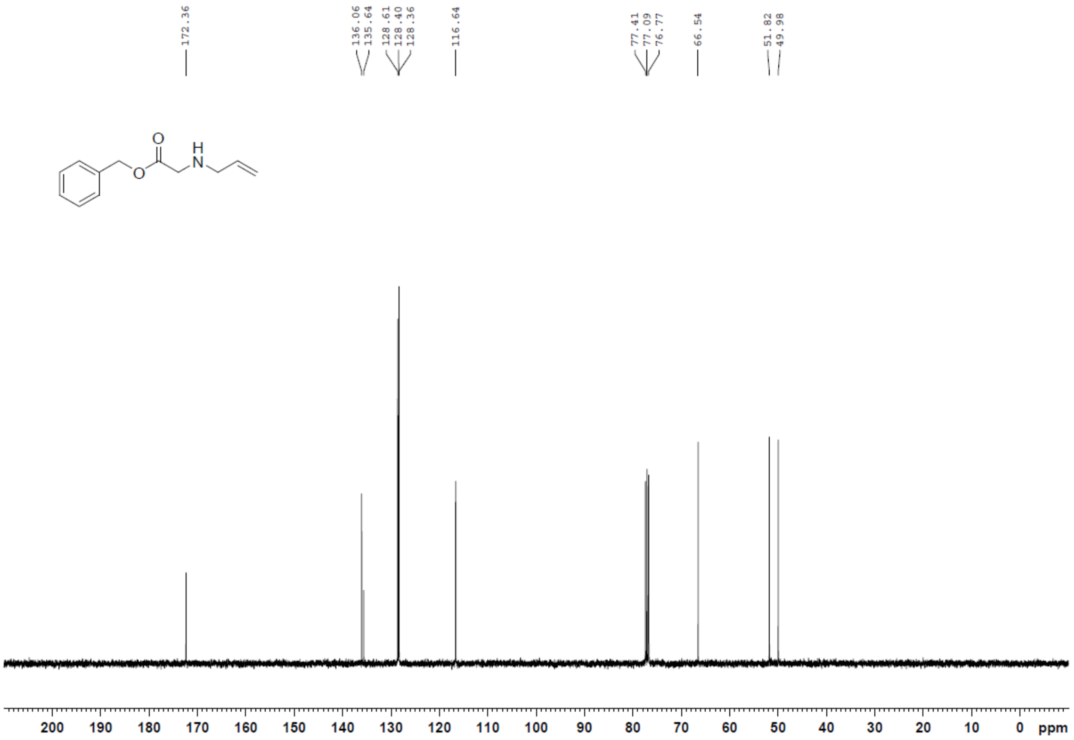

Supplementary Figure 38. ^{13}C NMR spectrum of **1r** in $CDCl_3$ (101 MHz)

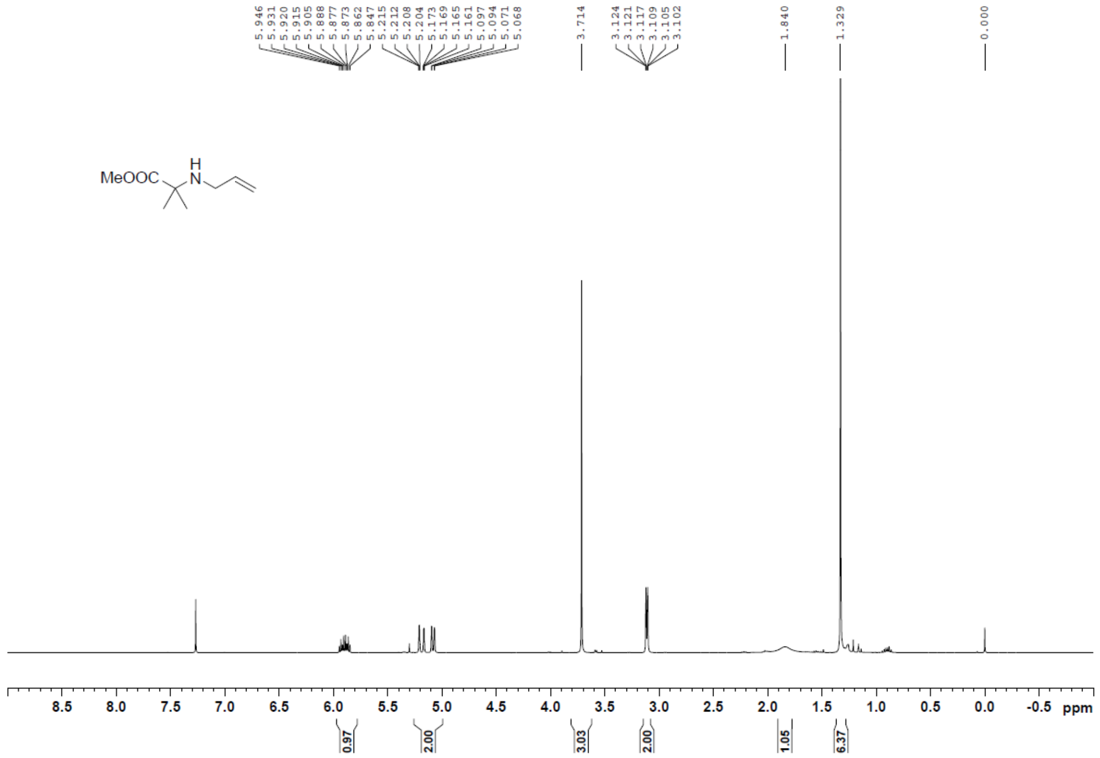

Supplementary Figure 39. ^{1}H NMR spectrum of **1s** in $CDCl_3$ (400 MHz)

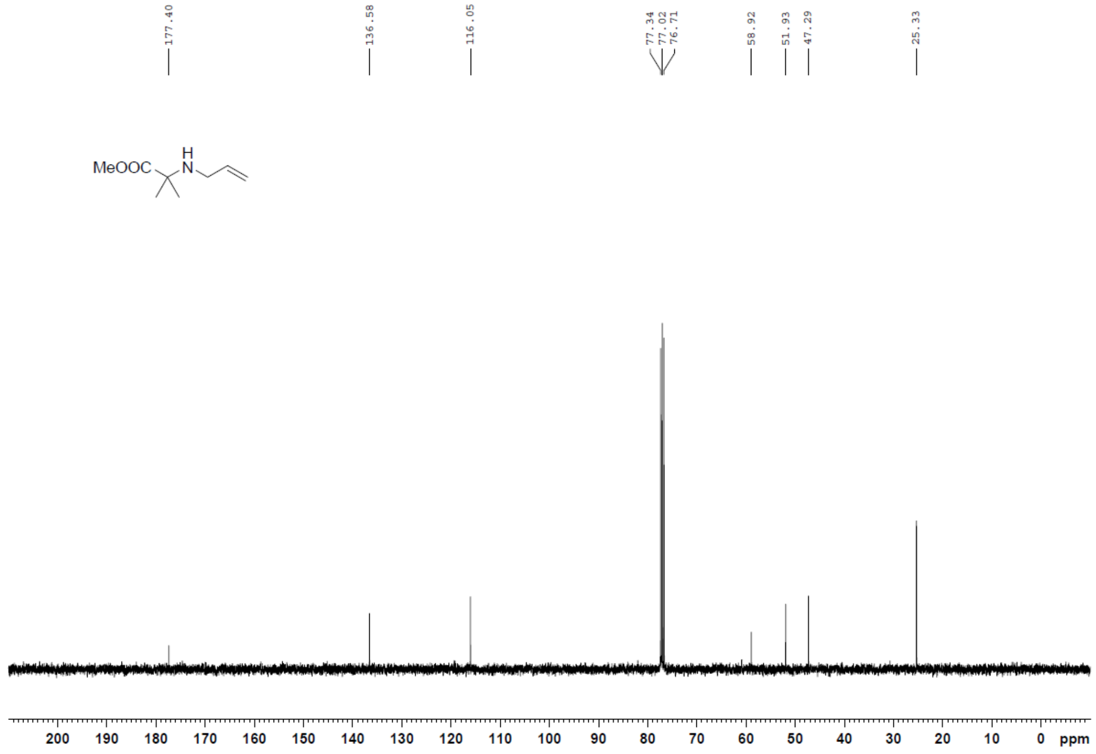

Supplementary Figure 40. ^{13}C NMR spectrum of **1s** in $CDCl_3$ (101 MHz)

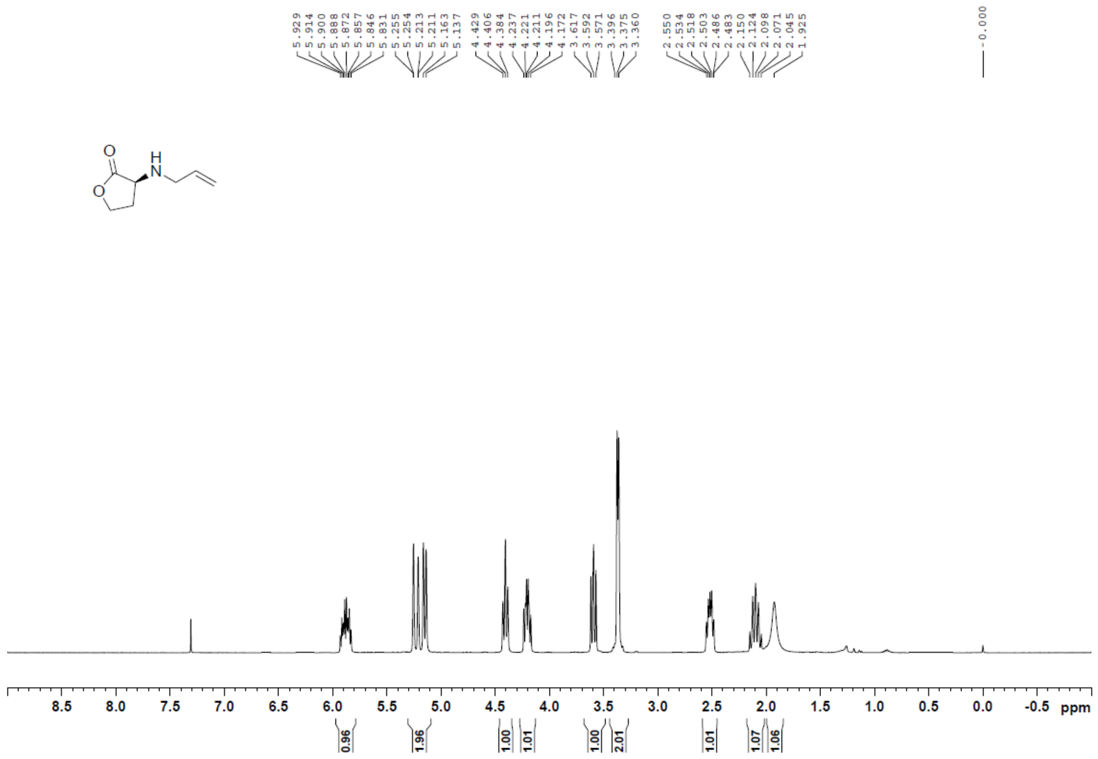

Supplementary Figure 41. ^{1}H NMR spectrum of **1t** in $CDCl_3$ (400 MHz)

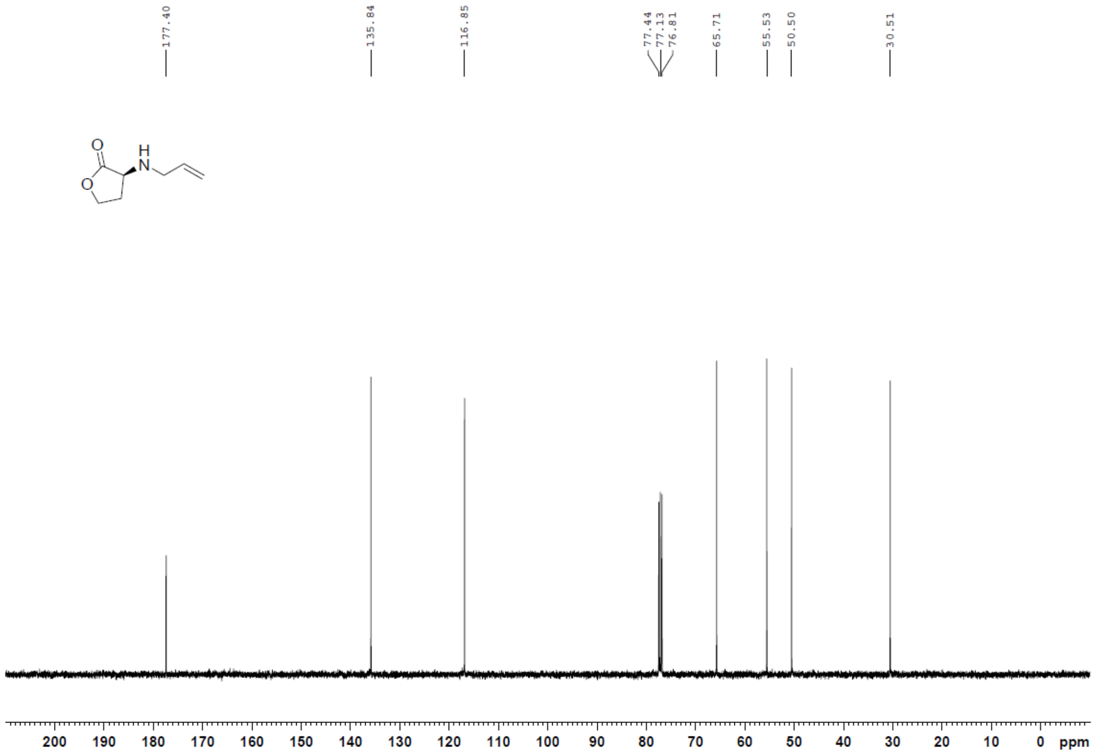

Supplementary Figure 42. ^{13}C NMR spectrum of **1t** in $CDCl_3$ (101 MHz)

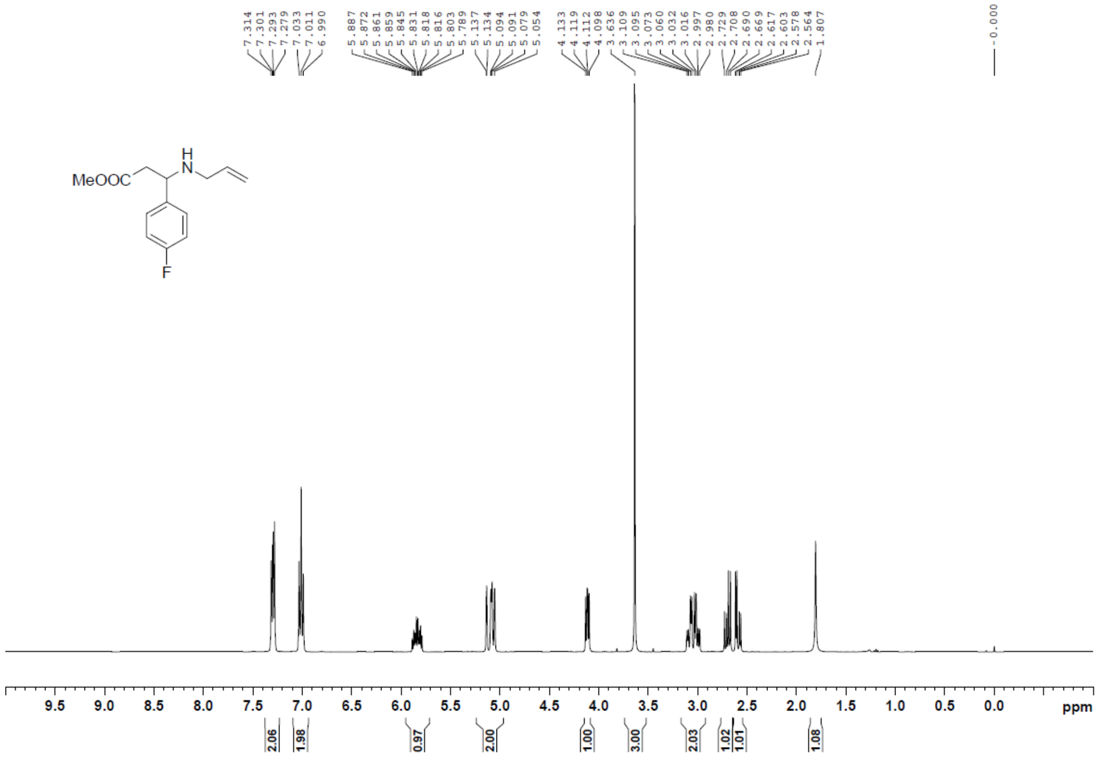

Supplementary Figure 43. ^{1}H NMR spectrum of **1u** in $CDCl_3$ (400 MHz)

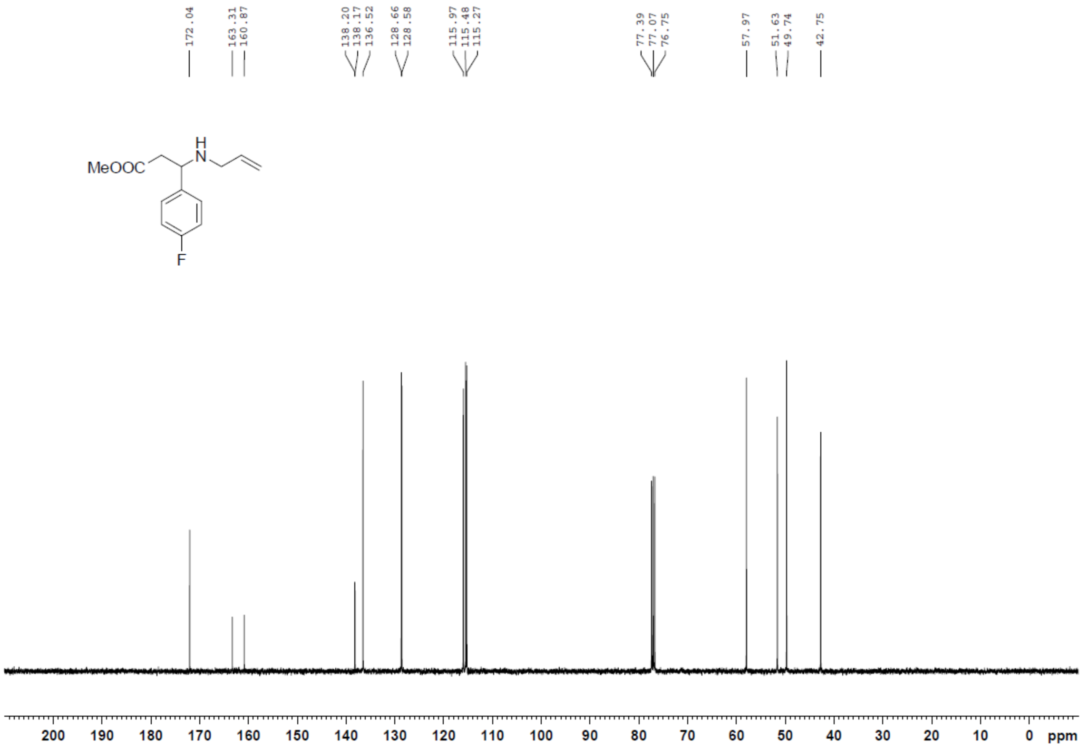

Supplementary Figure 44. ^{13}C NMR spectrum of **1u** in $CDCl_3$ (101 MHz)

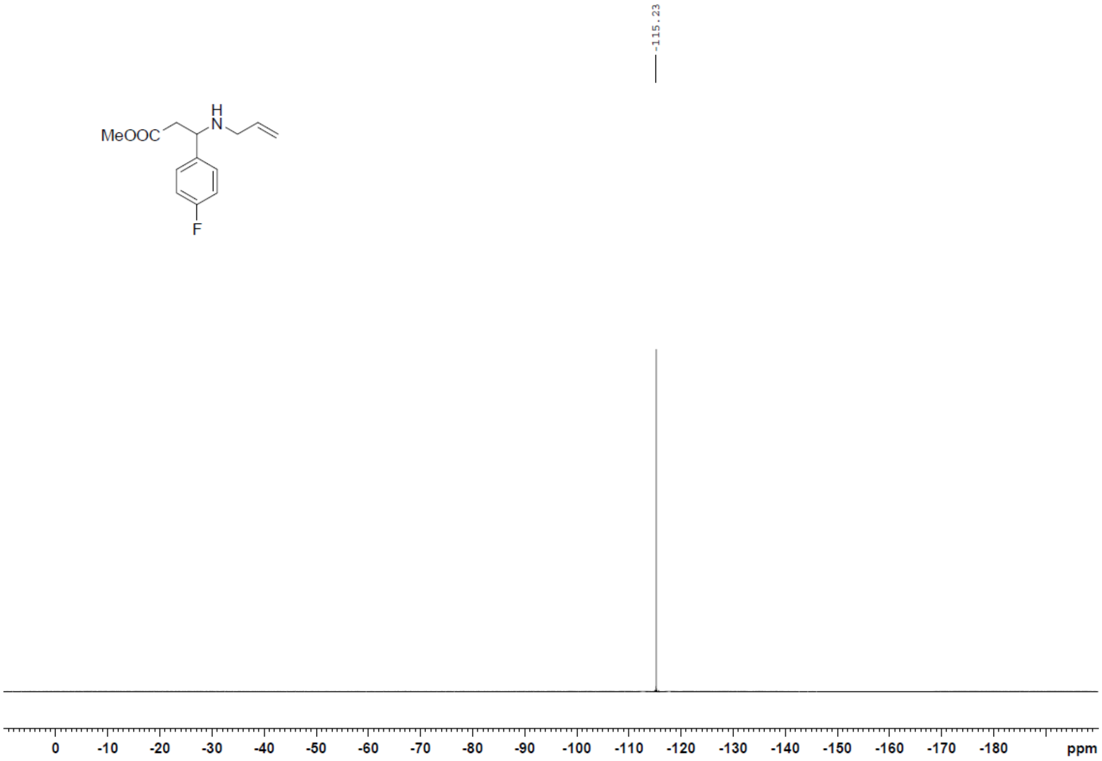

Supplementary Figure 45. ^{19}F NMR spectrum of **1u** in $CDCl_3$ (376 MHz)

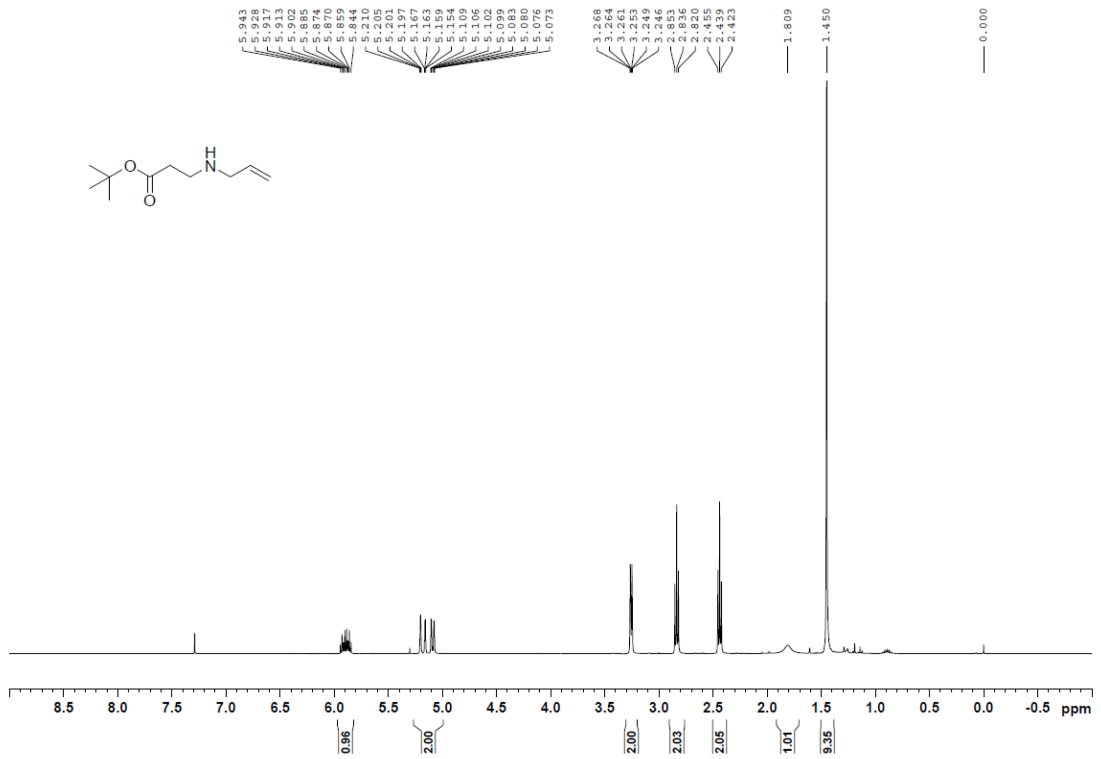

Supplementary Figure 46. ^{1}H NMR spectrum of **1v** in $CDCl_3$ (400 MHz)

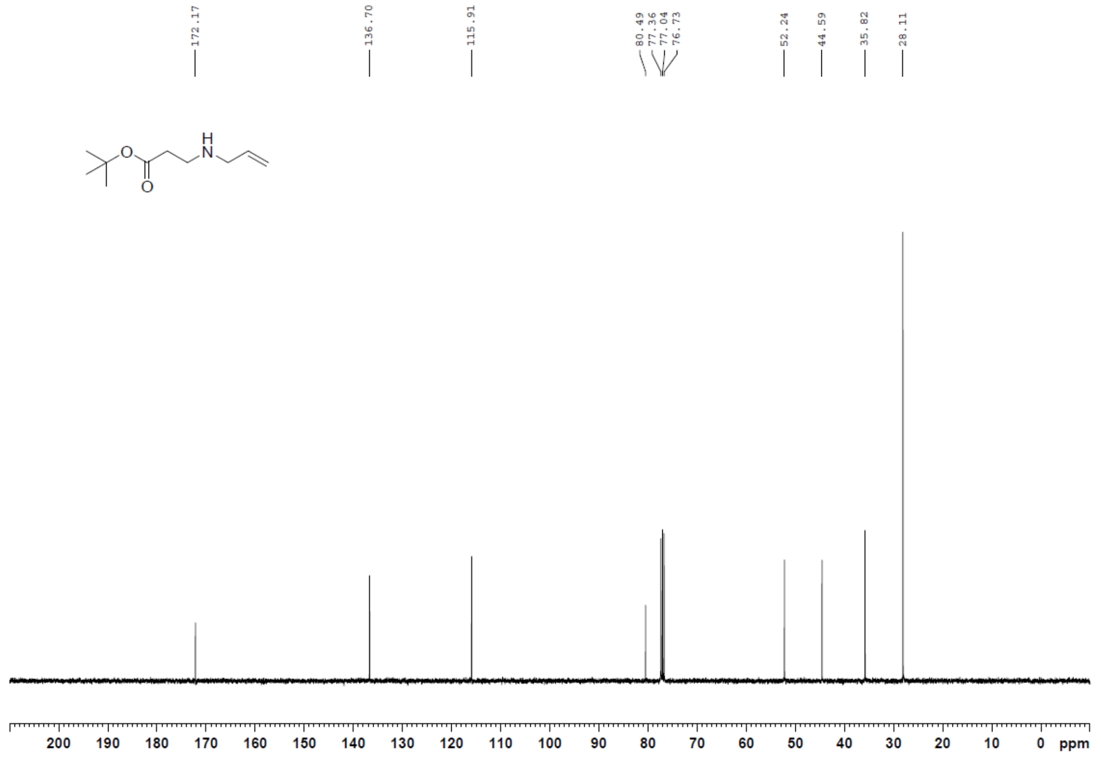

Supplementary Figure 47. ^{13}C NMR spectrum of **1v** in $CDCl_3$ (101 MHz)

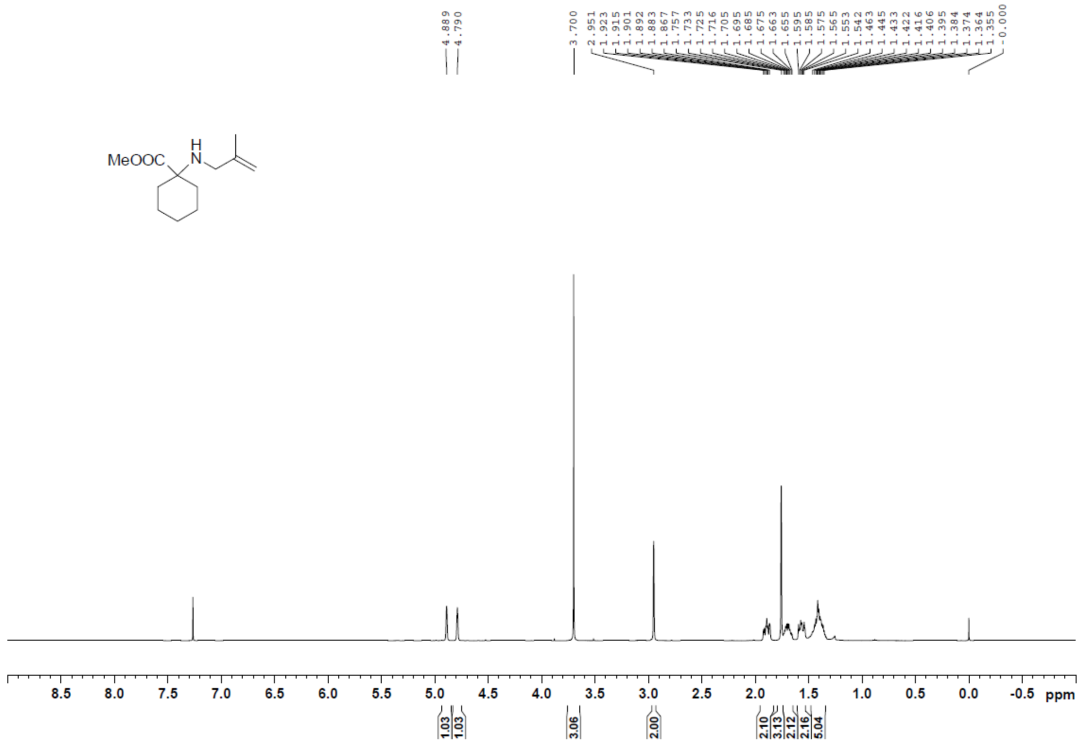

Supplementary Figure 48. ^{1}H NMR spectrum of **1z** in $CDCl_3$ (400 MHz)

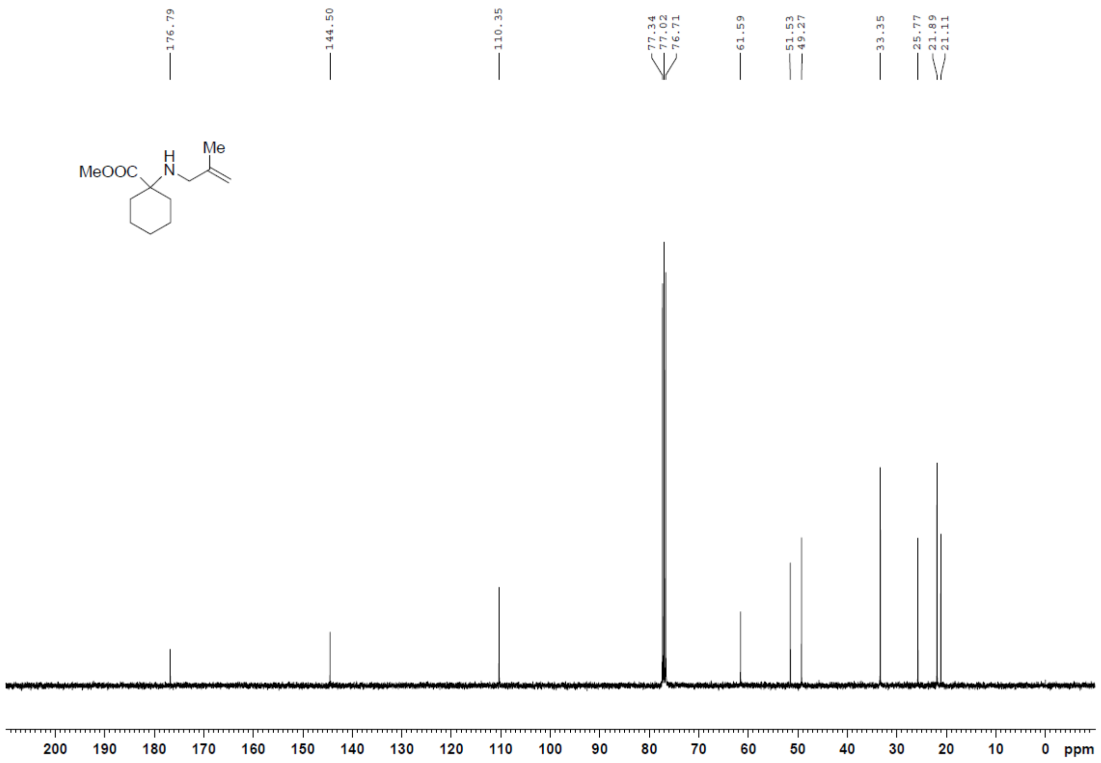

Supplementary Figure 49. ^{13}C NMR spectrum of **1z** in $CDCl_3$ (101 MHz)

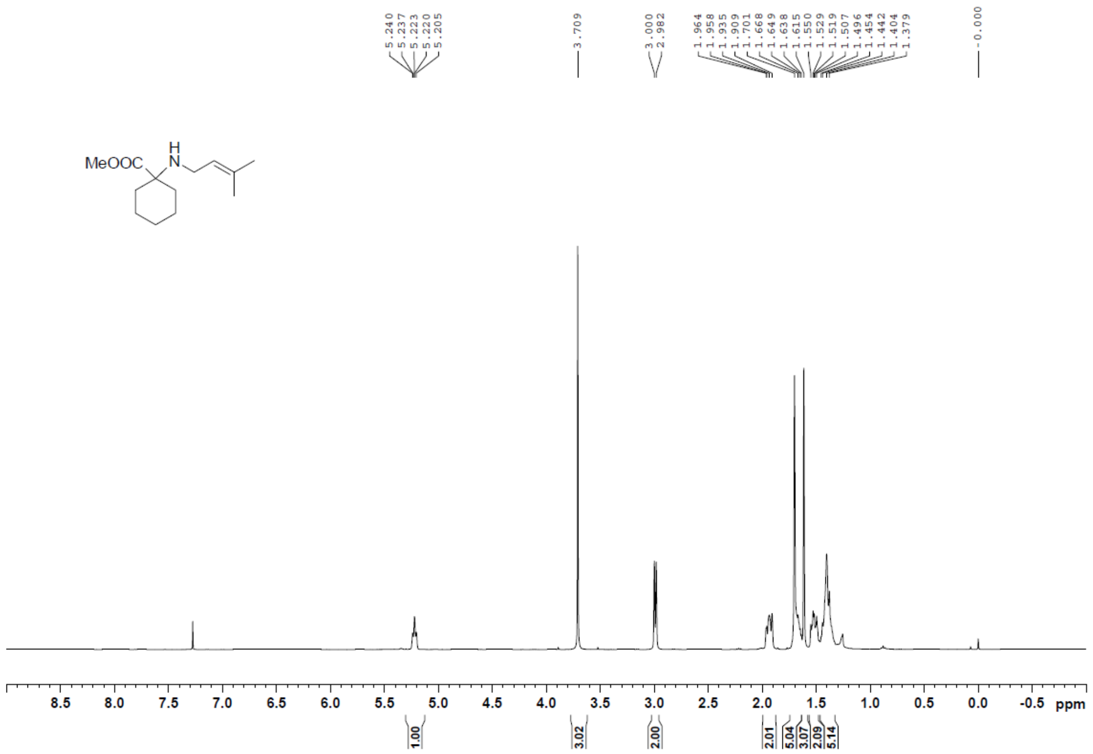

Supplementary Figure 50. ^{1}H NMR spectrum of **1aa** in $CDCl_3$ (400 MHz)

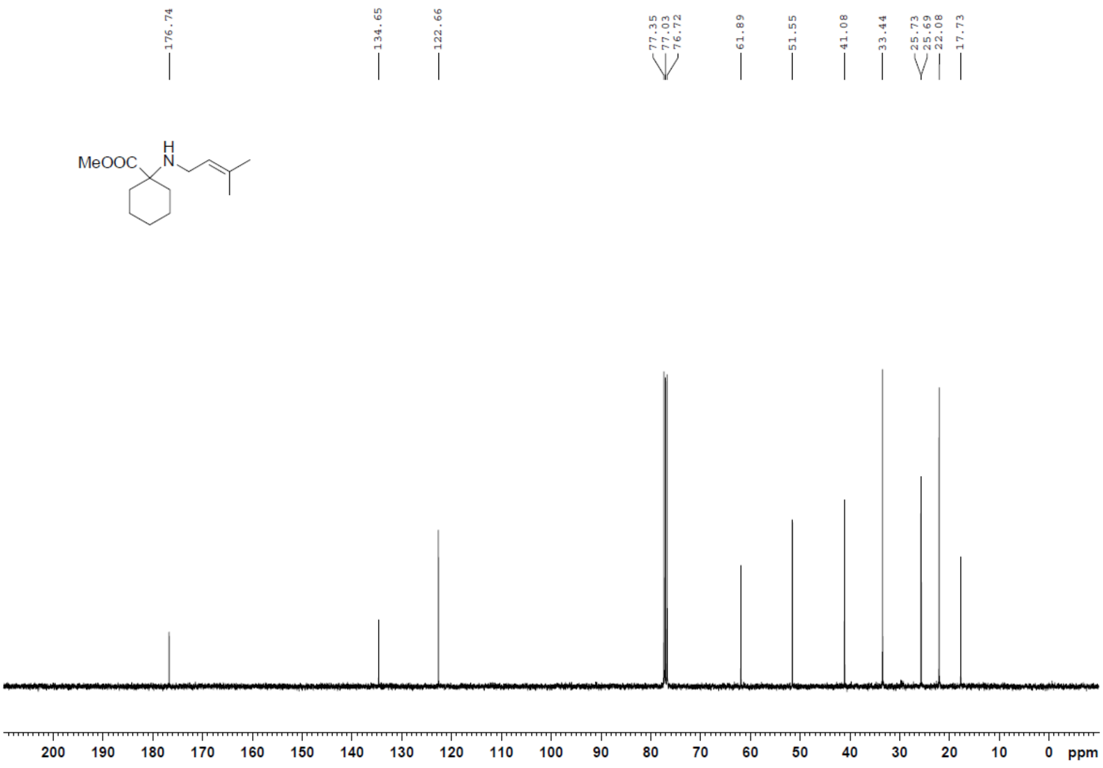

Supplementary Figure 51. ^{13}C NMR spectrum of **1aa** in $CDCl_3$ (101 MHz)

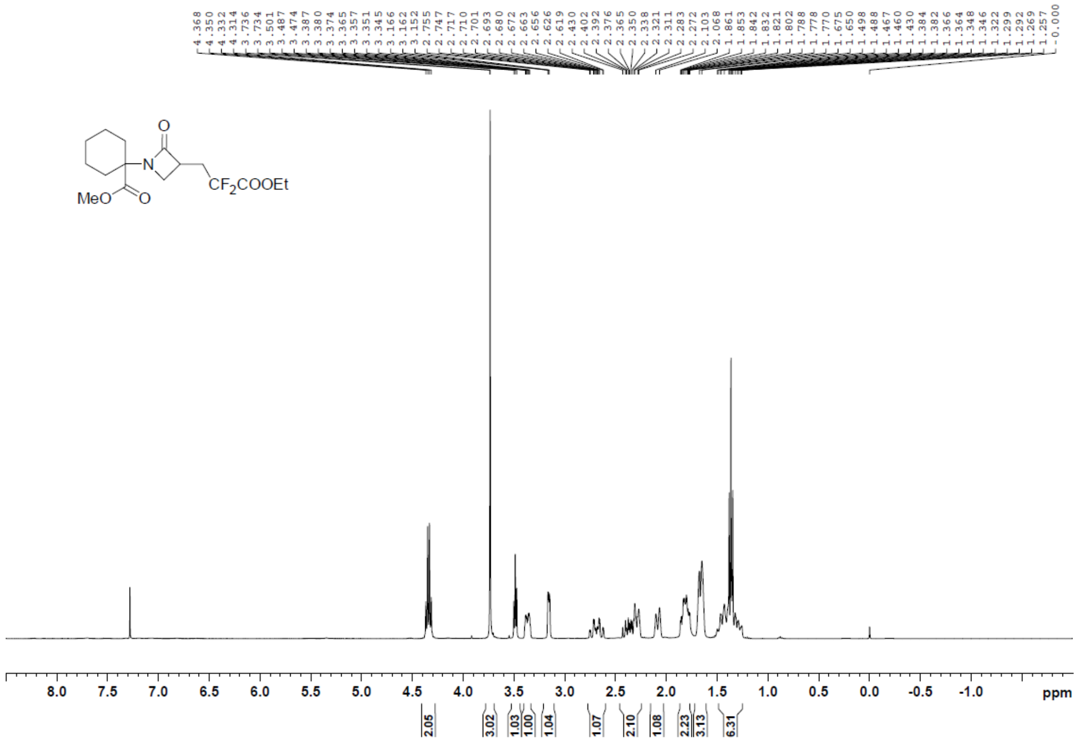

Supplementary Figure 52. ^{1}H NMR spectrum of **3a** in $CDCl_3$ (400 MHz)

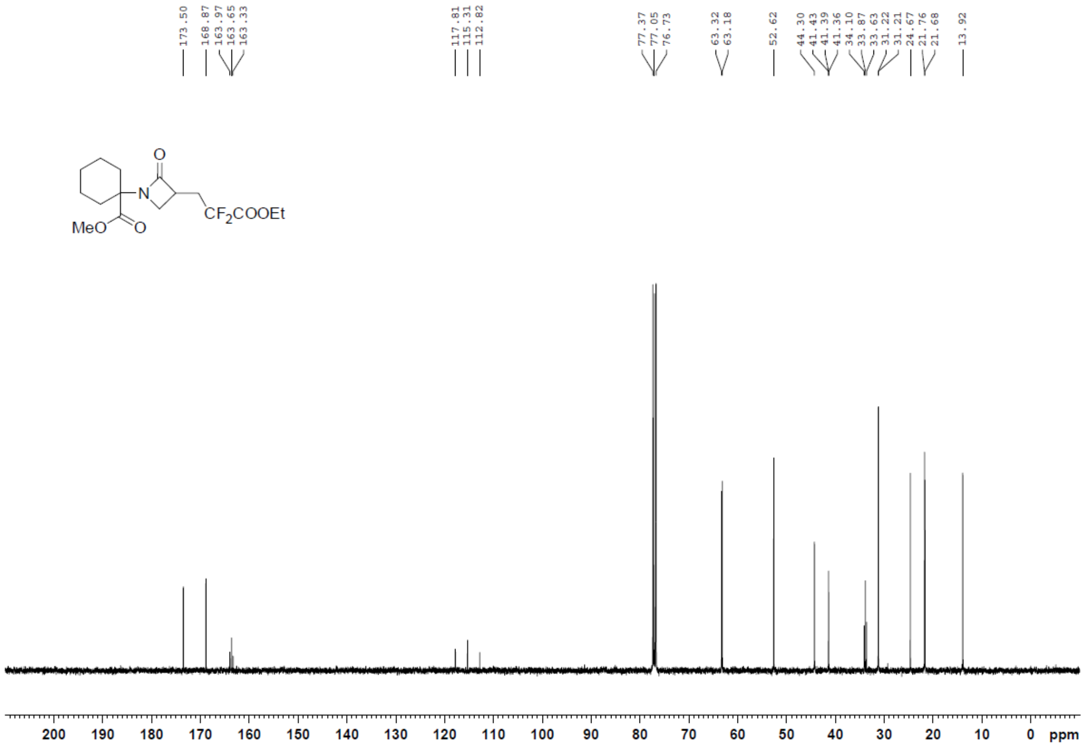

Supplementary Figure 53. ^{13}C NMR spectrum of **3a** in $CDCl_3$ (101 MHz)

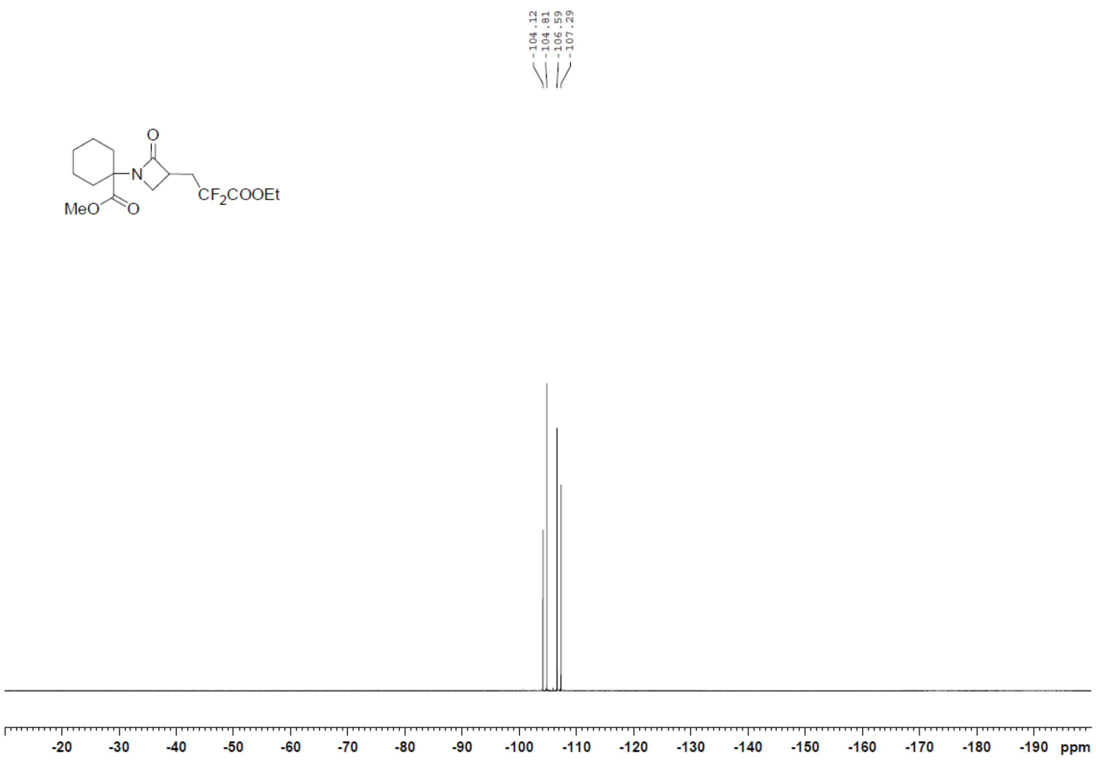

Supplementary Figure 54. ^{19}F NMR spectrum of **3a** in $CDCl_3$ (376 MHz)

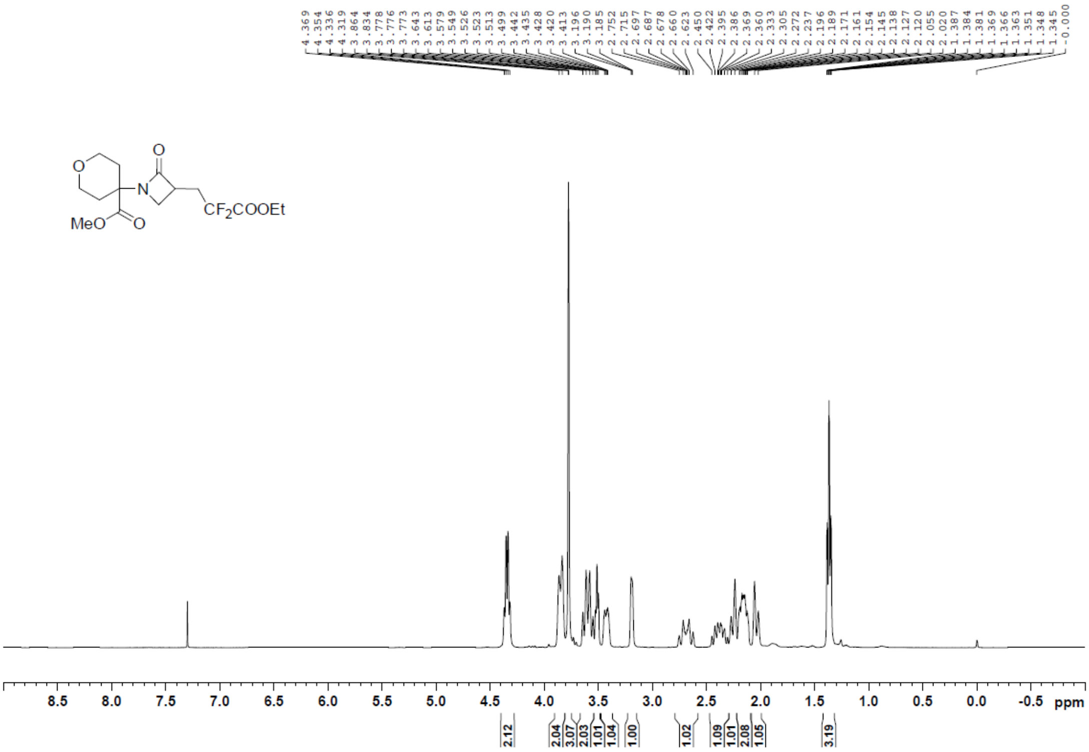

Supplementary Figure 55. ^{1}H NMR spectrum of **3b** in $CDCl_3$ (400 MHz)

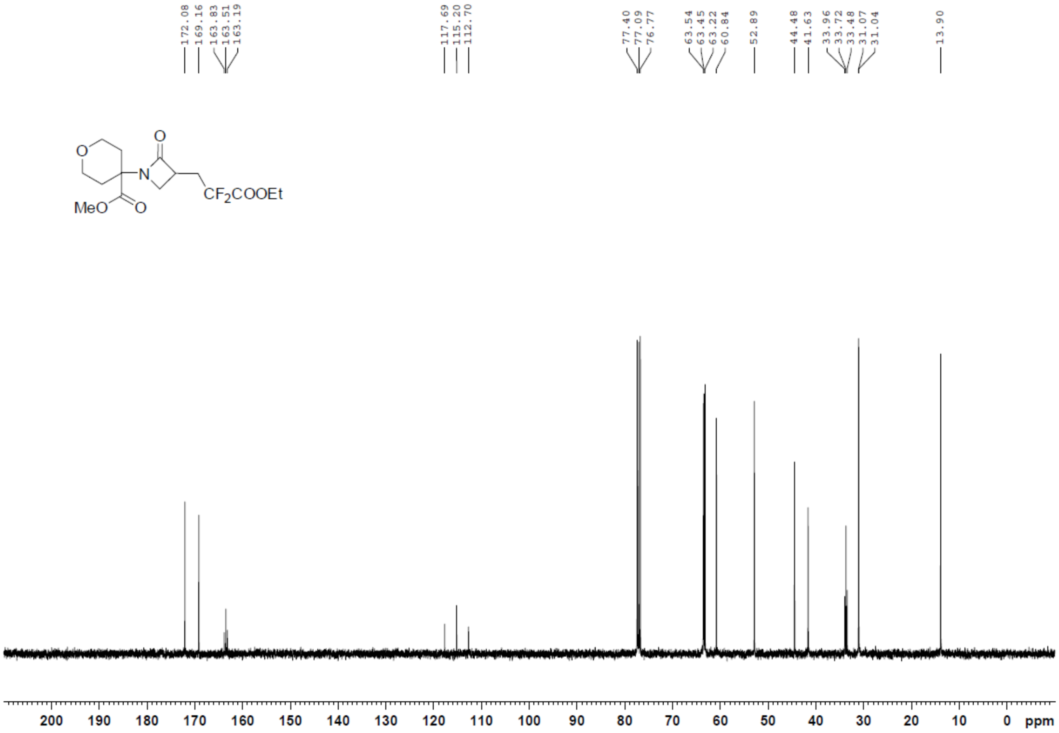

Supplementary Figure 56. ^{13}C NMR spectrum of **3b** in $CDCl_3$ (101 MHz)

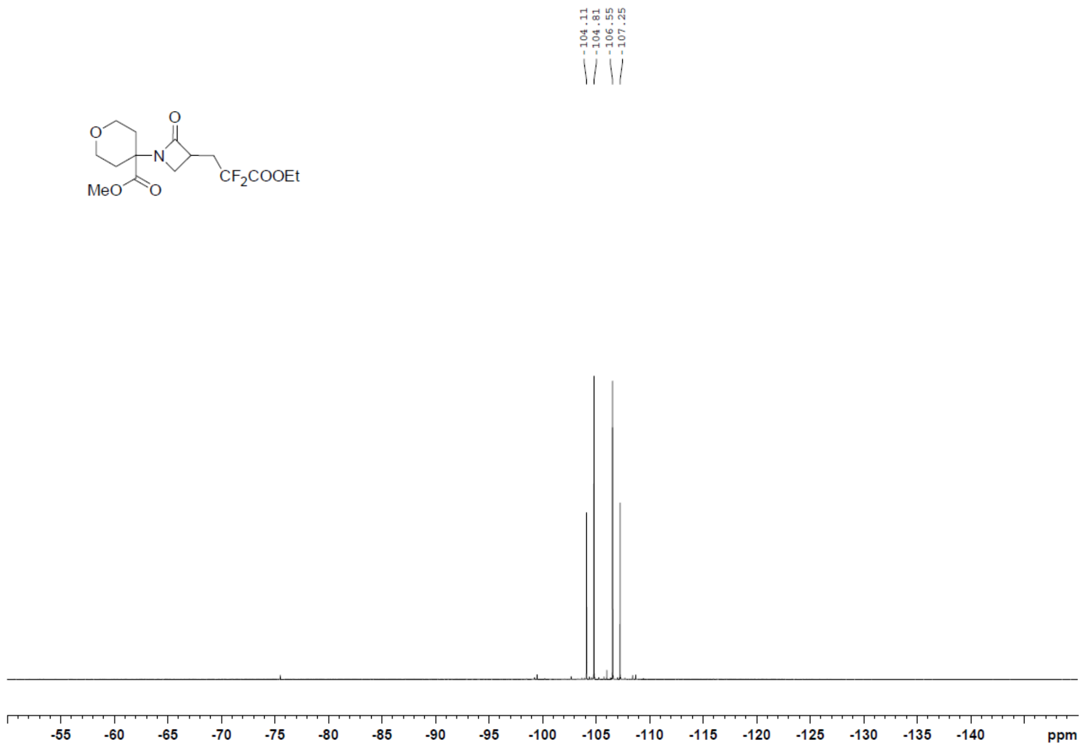

Supplementary Figure 57. ^{19}F NMR spectrum of **3b** in $CDCl_3$ (376 MHz)

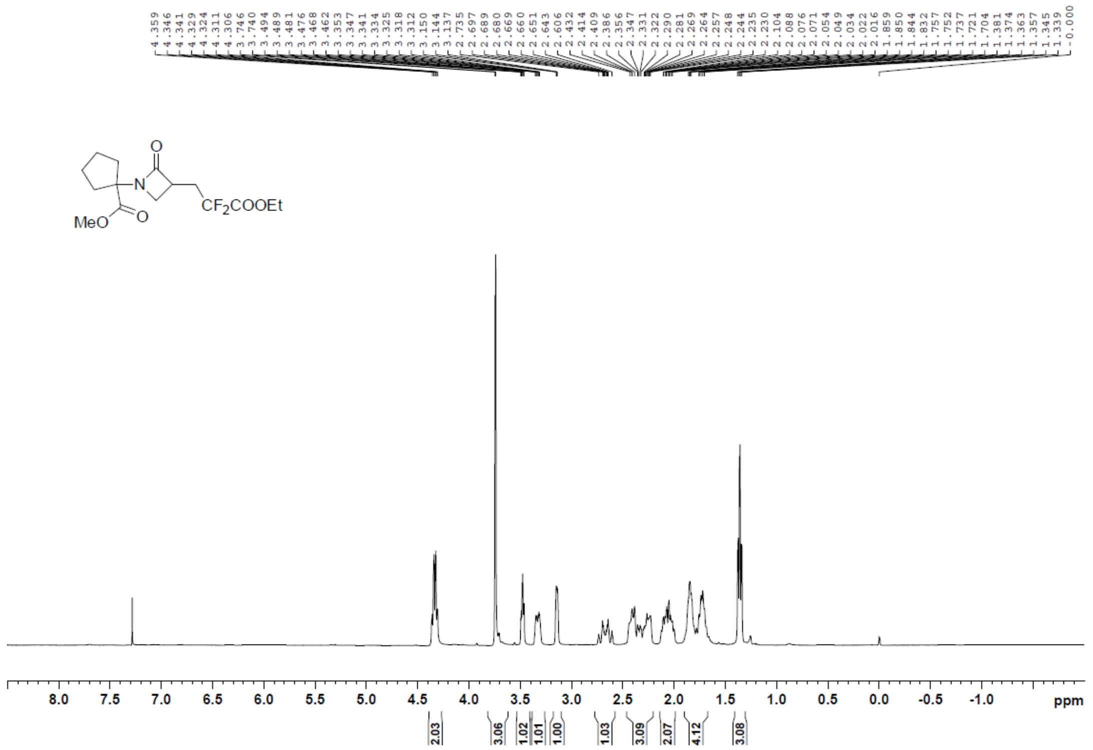

Supplementary Figure 58. ^{1}H NMR spectrum of **3c** in $CDCl_3$ (400 MHz)

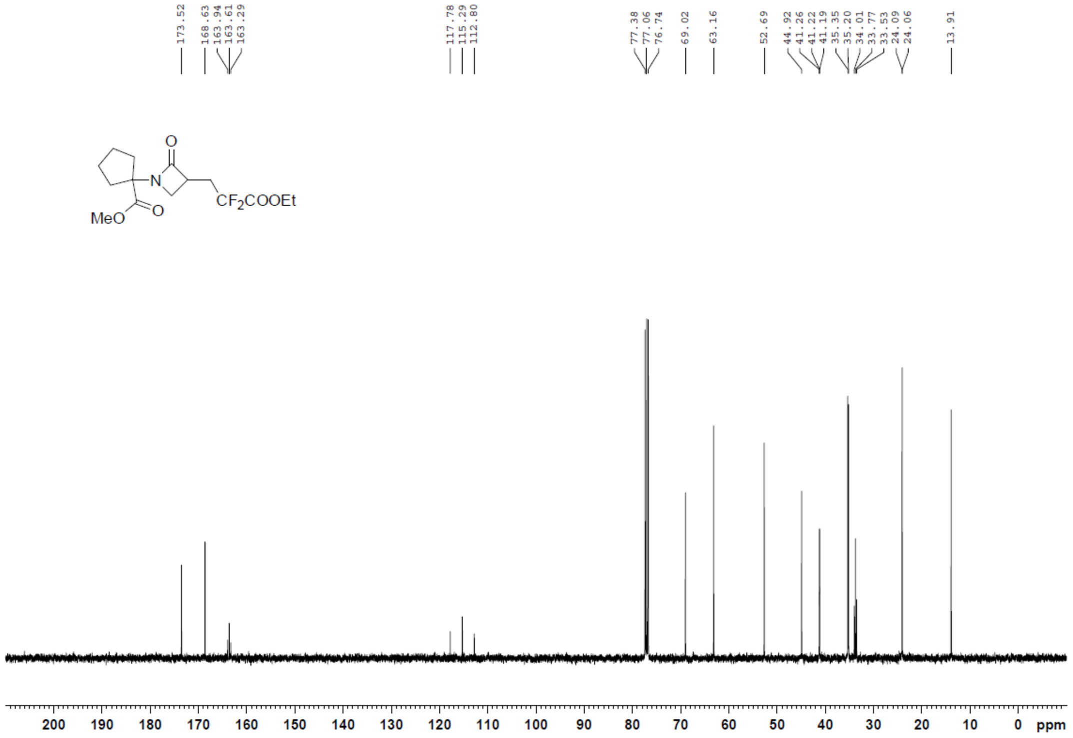

Supplementary Figure 59. ^{13}C NMR spectrum of **3c** in $CDCl_3$ (101 MHz)

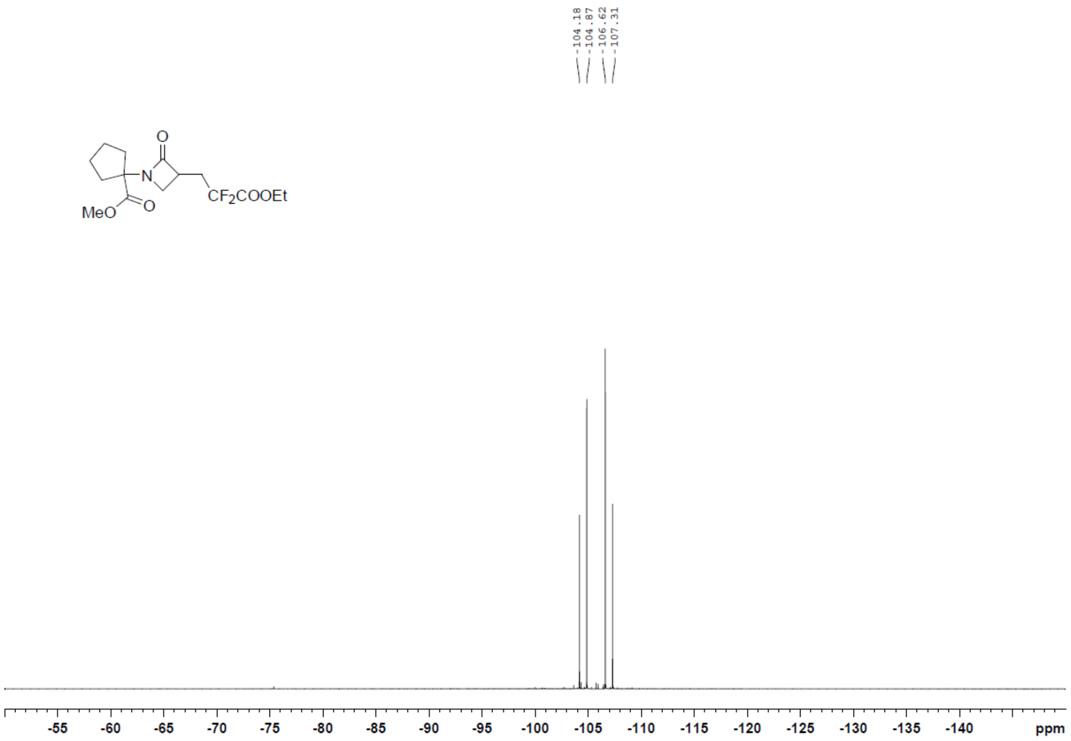

Supplementary Figure 60. ^{19}F NMR spectrum of **3c** in $CDCl_3$ (376 MHz)

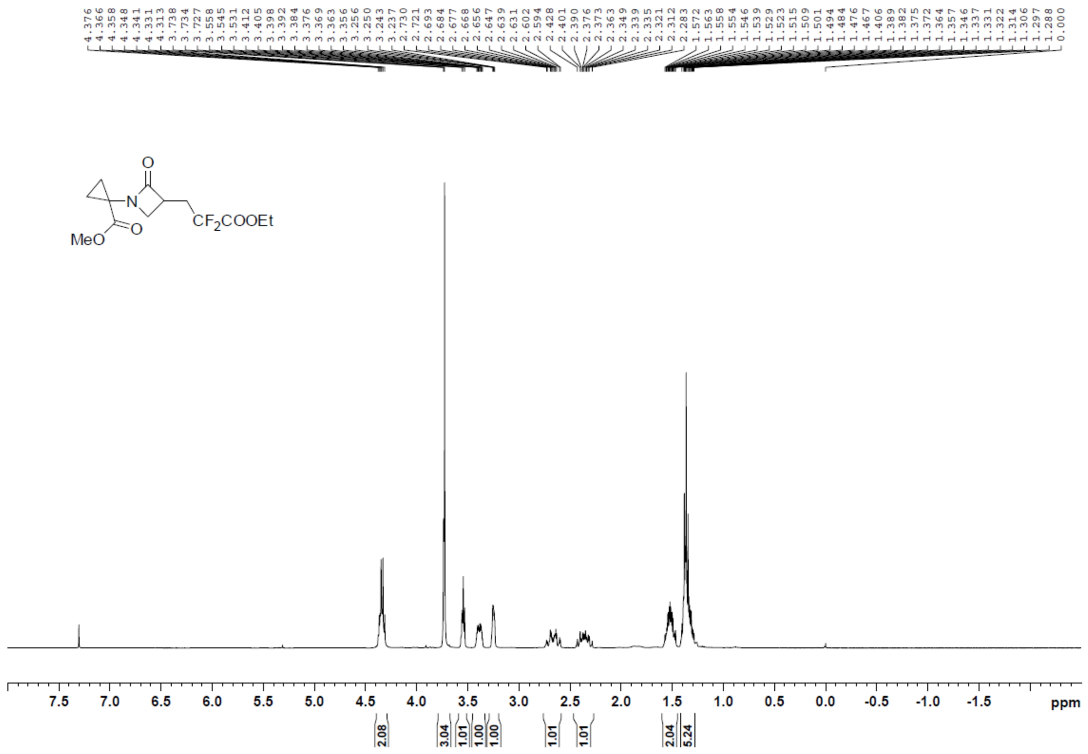

Supplementary Figure 61. ^{1}H NMR spectrum of **3d** in $CDCl_3$ (400 MHz)

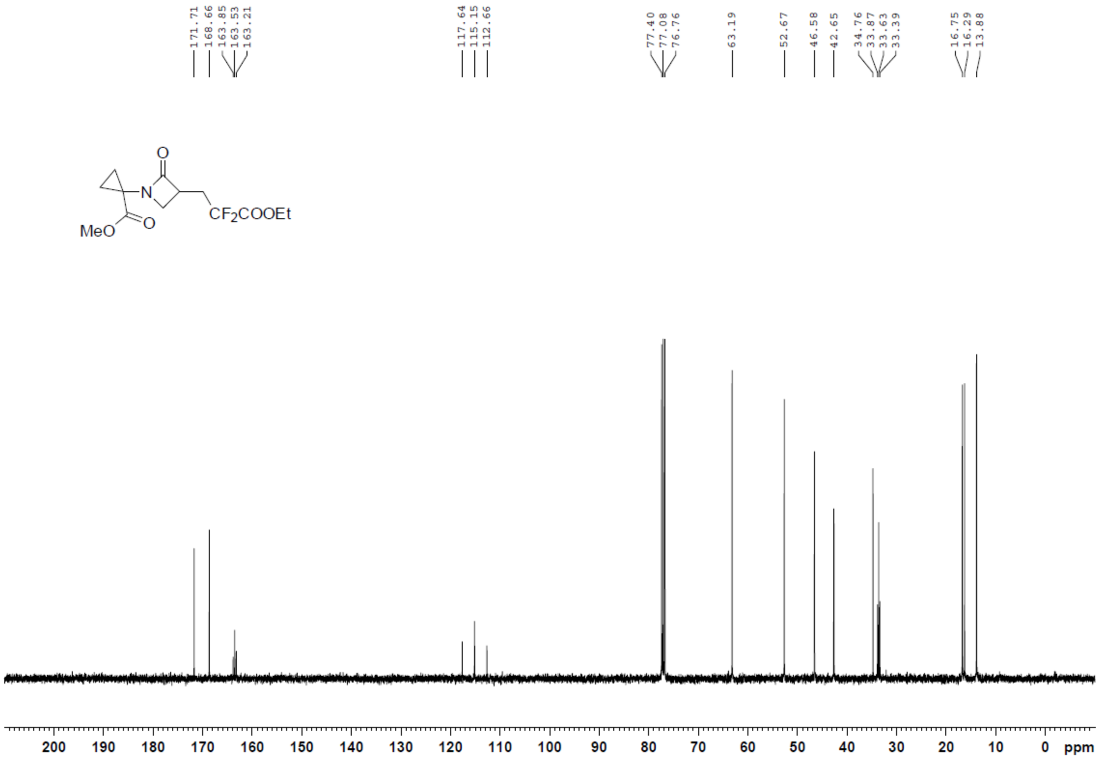

Supplementary Figure 62. ^{13}C NMR spectrum of **3d** in $CDCl_3$ (101 MHz)

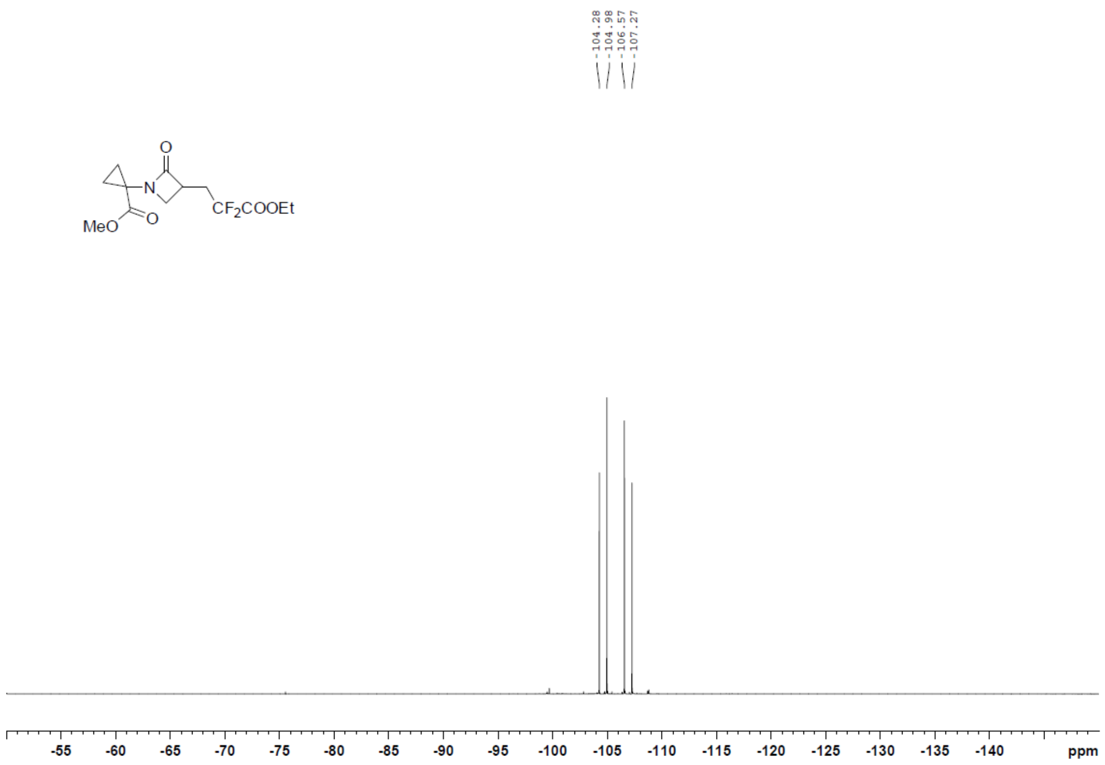

Supplementary Figure 63. ^{19}F NMR spectrum of **3d** in $CDCl_3$ (376 MHz)

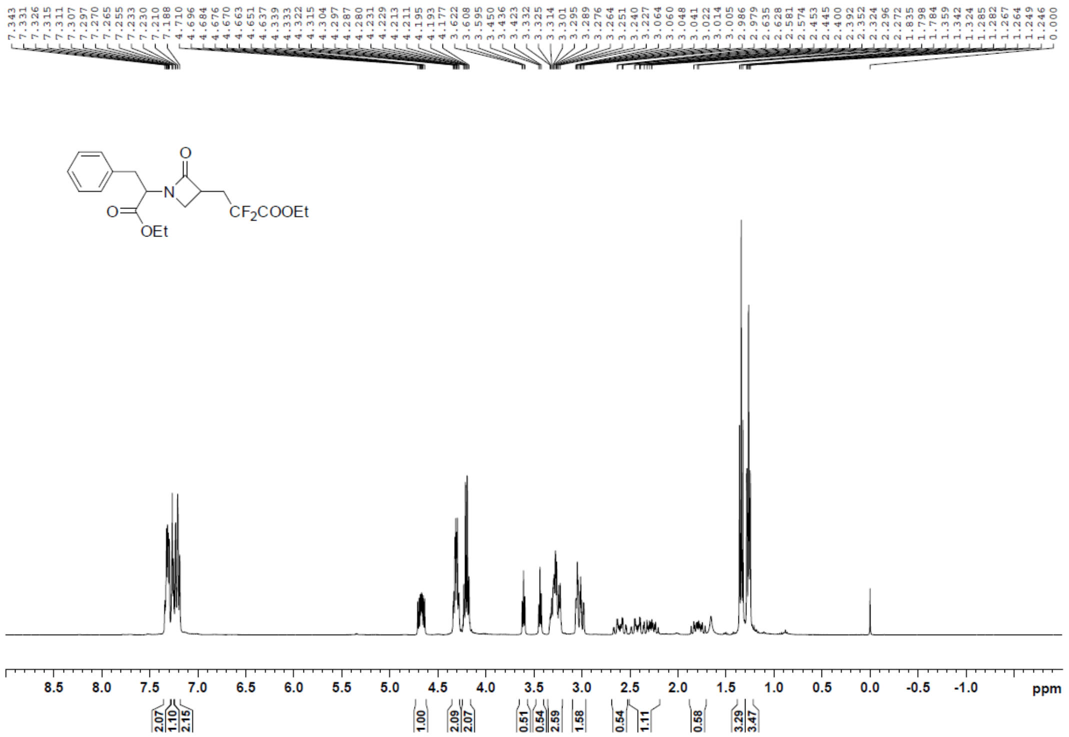

Supplementary Figure 64. ^{1}H NMR spectrum of **3e** in $CDCl_3$ (400 MHz)

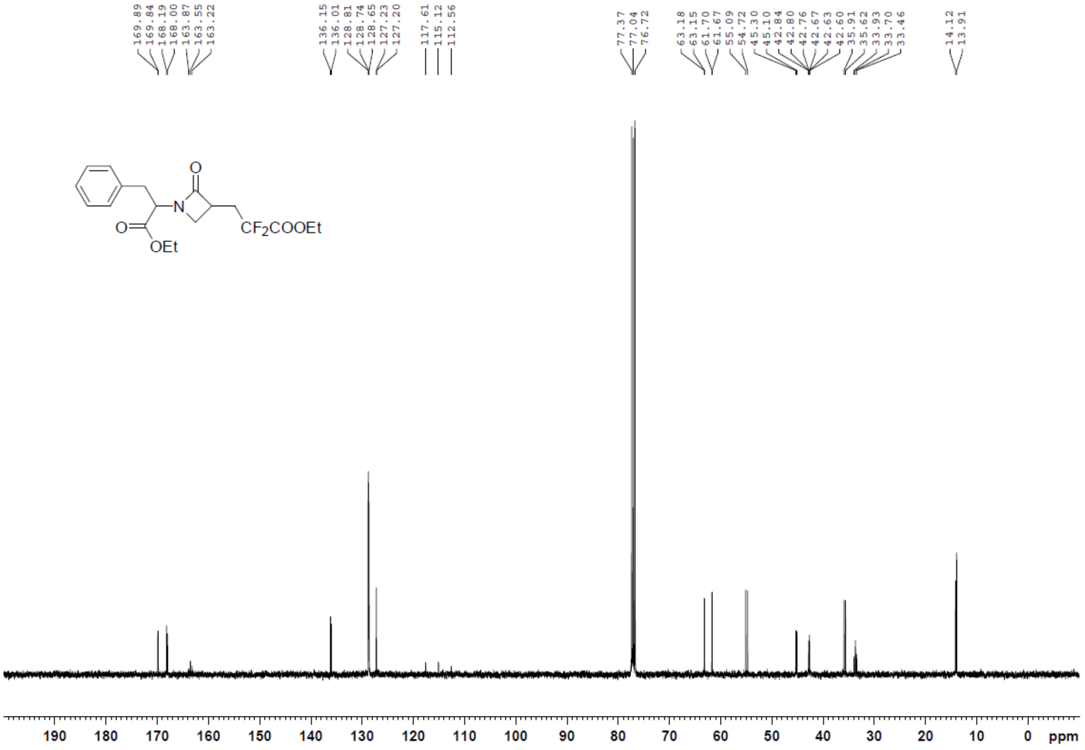

Supplementary Figure 65. ^{13}C NMR spectrum of **3e** in $CDCl_3$ (101 MHz)

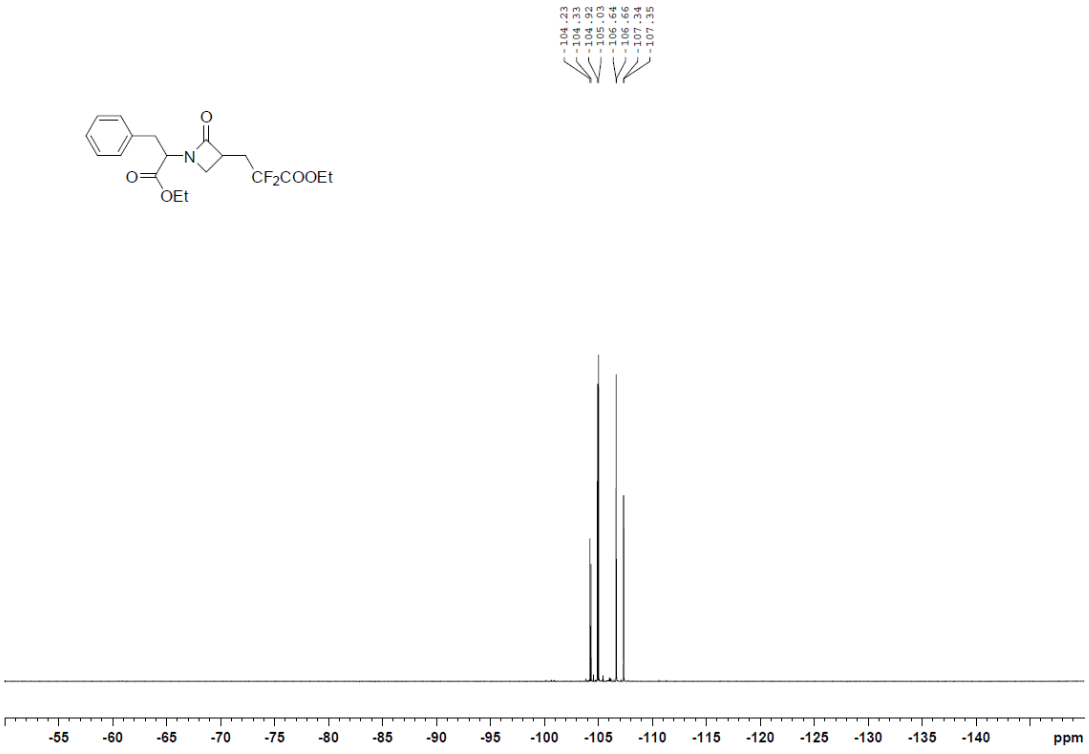

Supplementary Figure 66. ^{19}F NMR spectrum of **3e** in $CDCl_3$ (376 MHz)

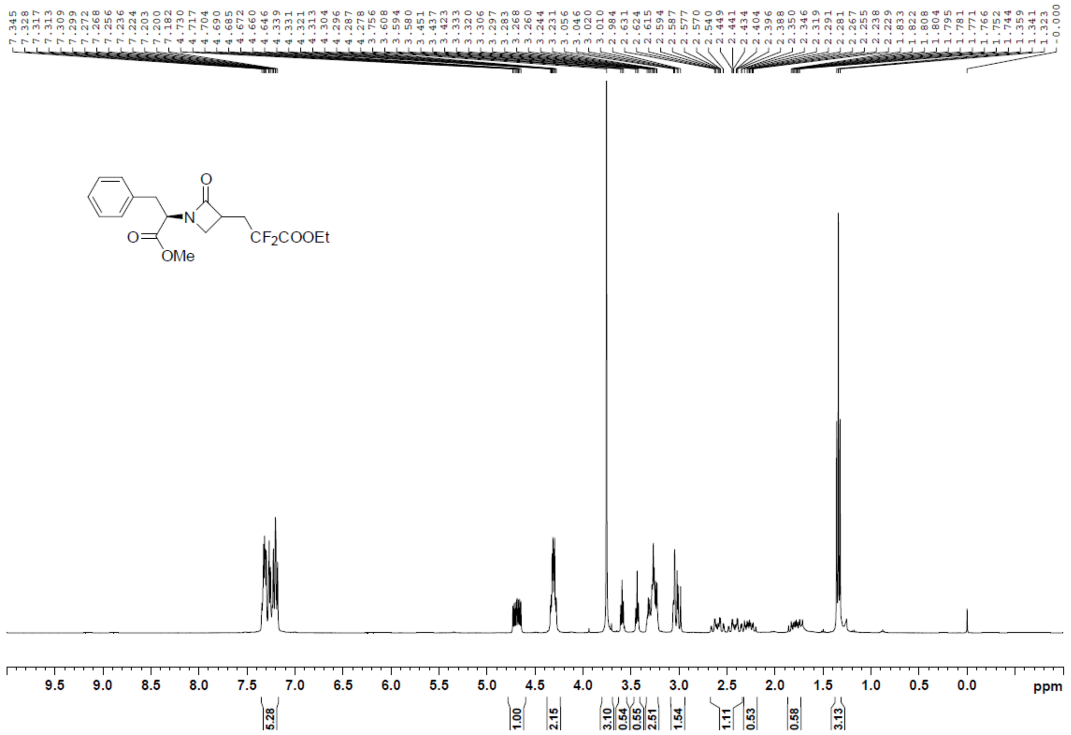

Supplementary Figure 67. ^{1}H NMR spectrum of **3f** in $CDCl_3$ (400 MHz)

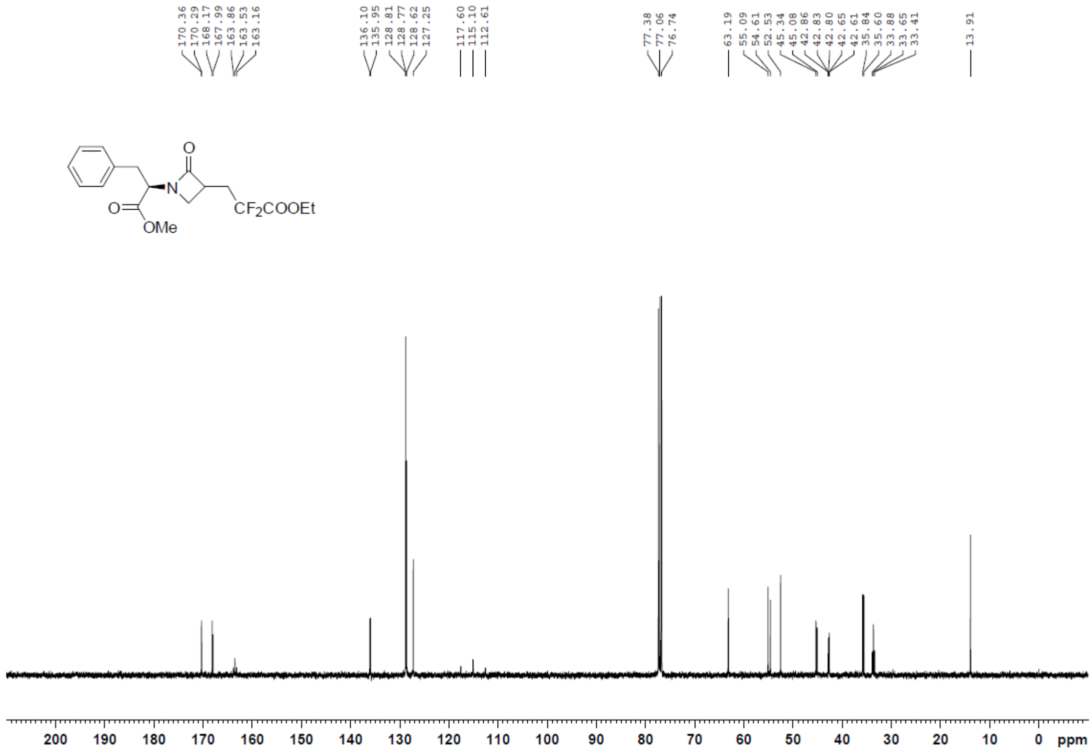

Supplementary Figure 68. ^{13}C NMR spectrum of **3f** in $CDCl_3$ (101 MHz)

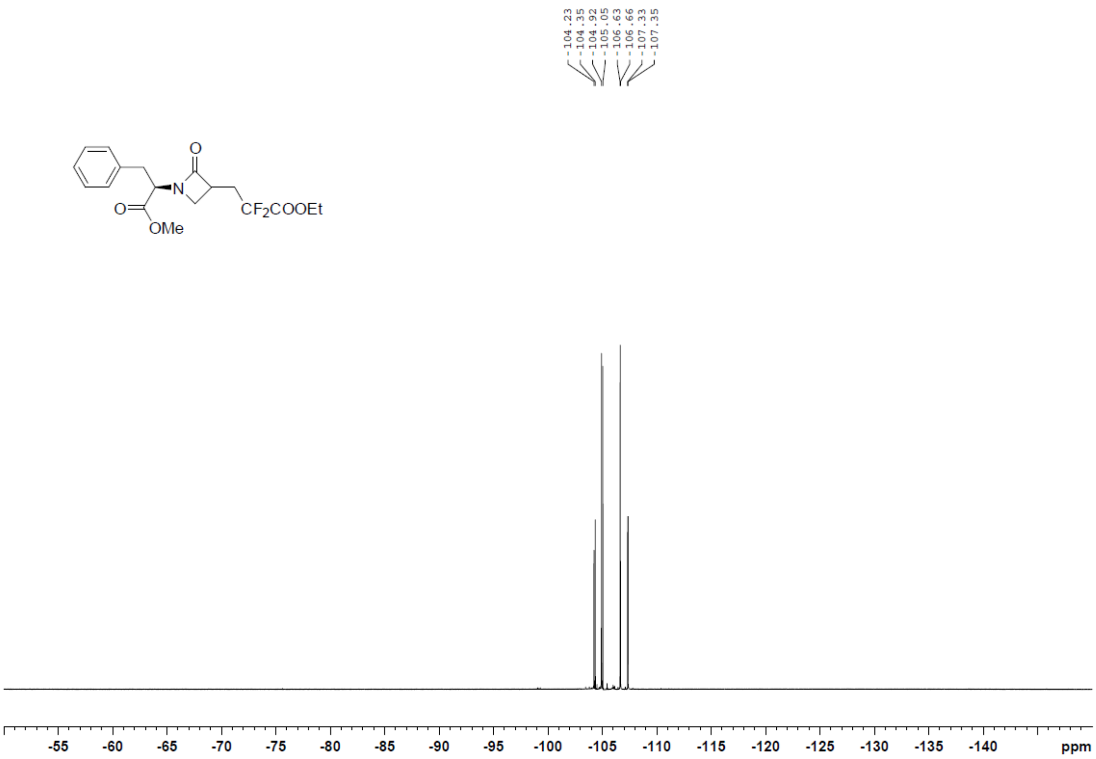

Supplementary Figure 69. ^{19}F NMR spectrum of **3f** in $CDCl_3$ (376 MHz)

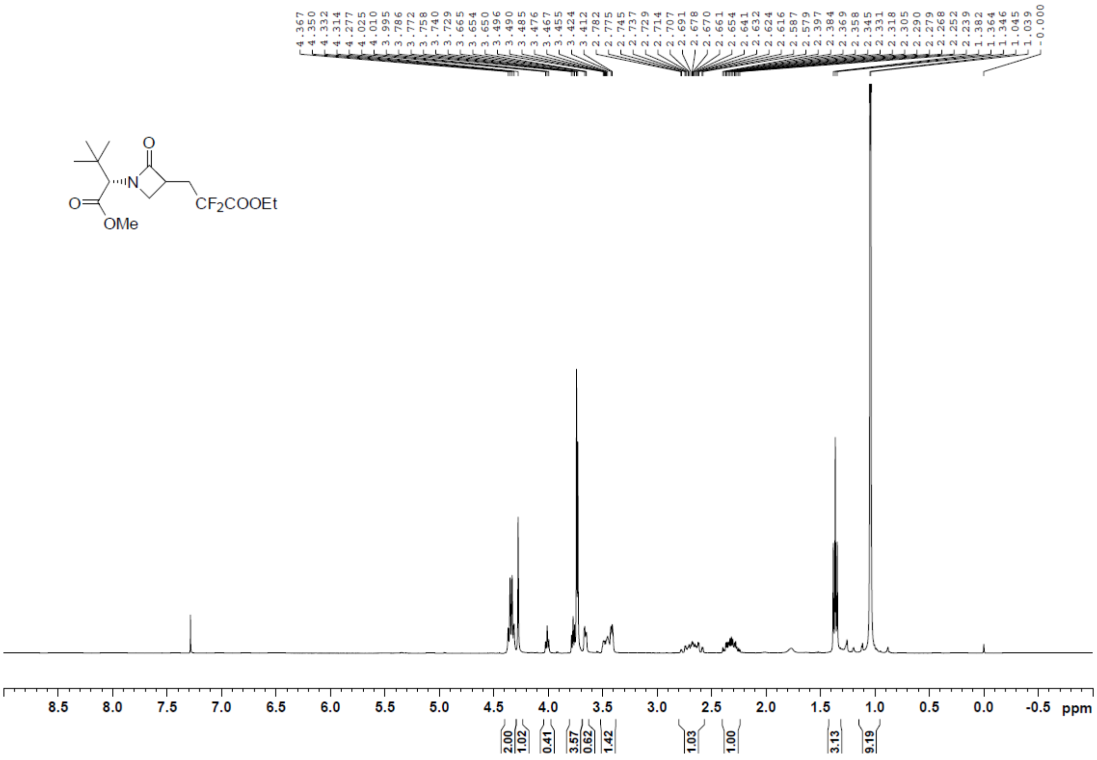

Supplementary Figure 70. ^{1}H NMR spectrum of **3g** in $CDCl_3$ (400 MHz)

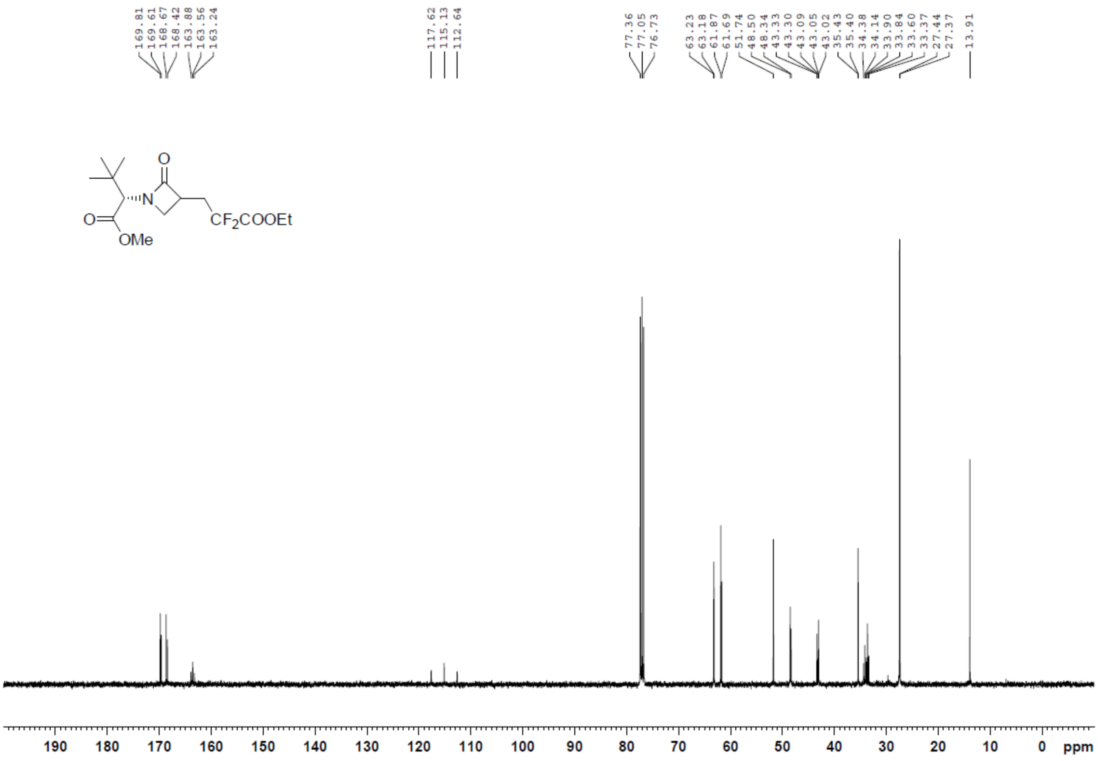

Supplementary Figure 71. ^{13}C NMR spectrum of **3g** in $CDCl_3$ (101 MHz)

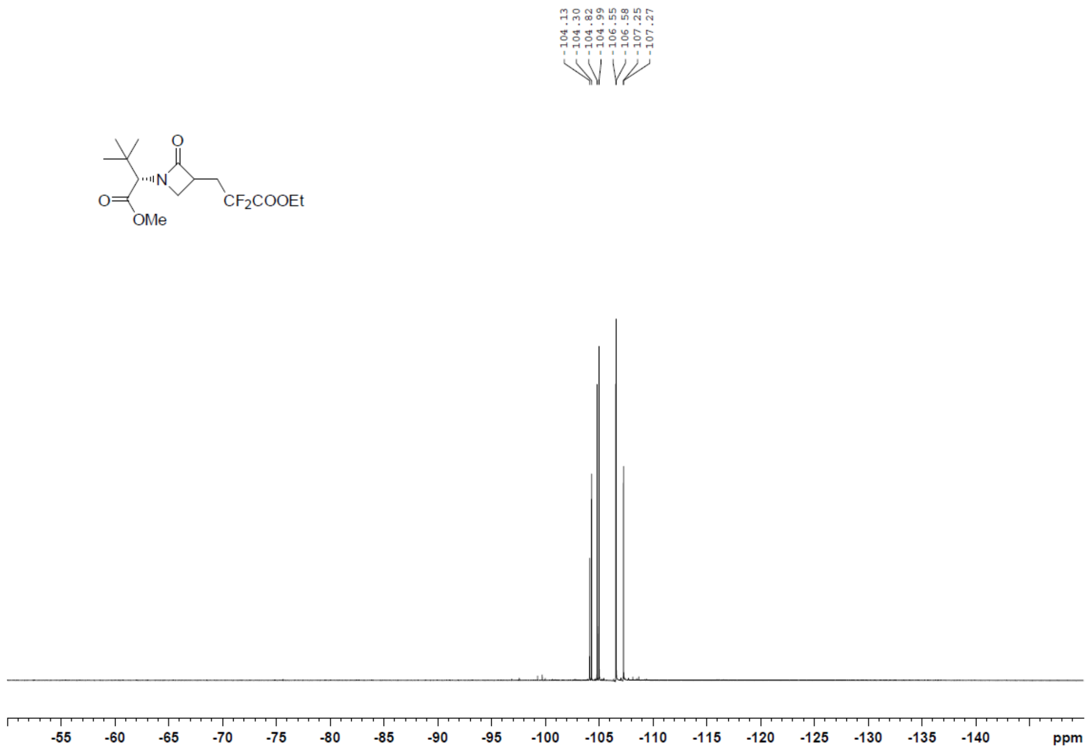

Supplementary Figure 72. ^{19}F NMR spectrum of **3g** in $CDCl_3$ (376 MHz)

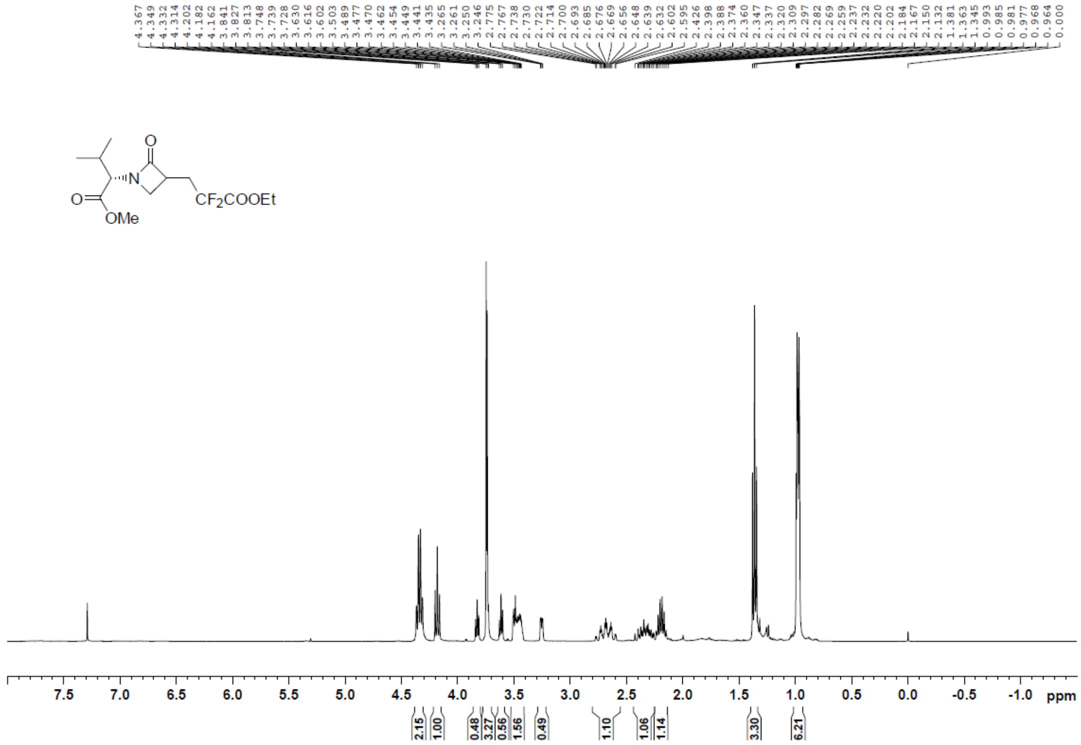

Supplementary Figure 73. ^{1}H NMR spectrum of **3h** in $CDCl_3$ (400 MHz)

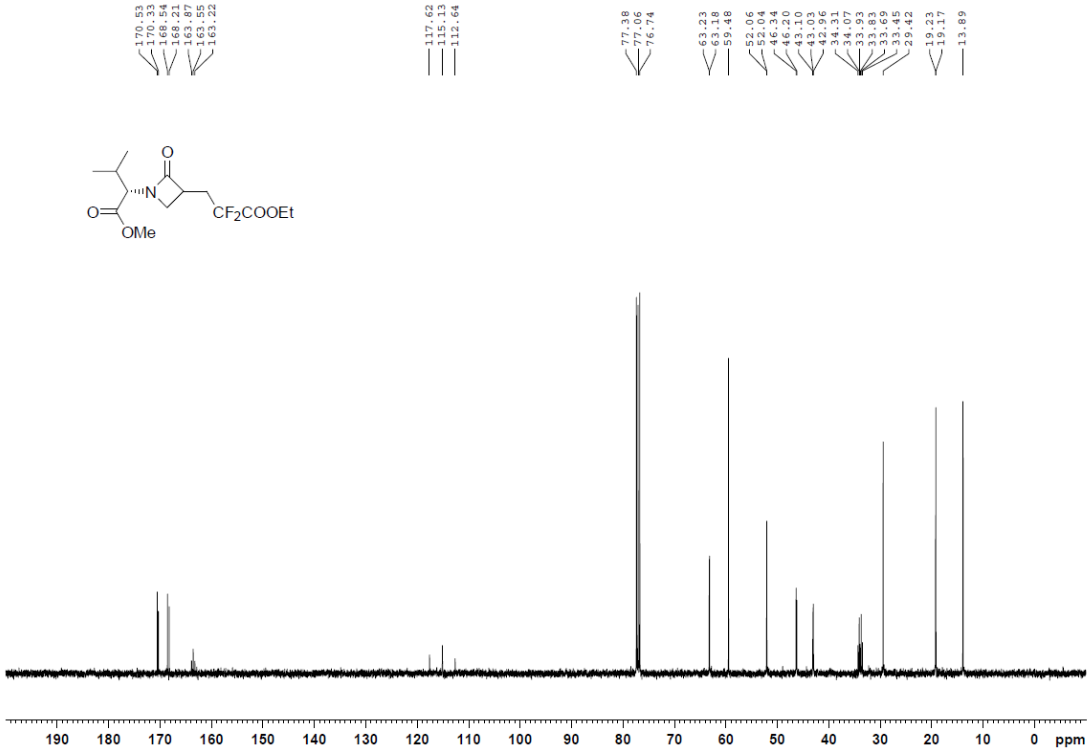

Supplementary Figure 74. ^{13}C NMR spectrum of **3h** in $CDCl_3$ (101 MHz)

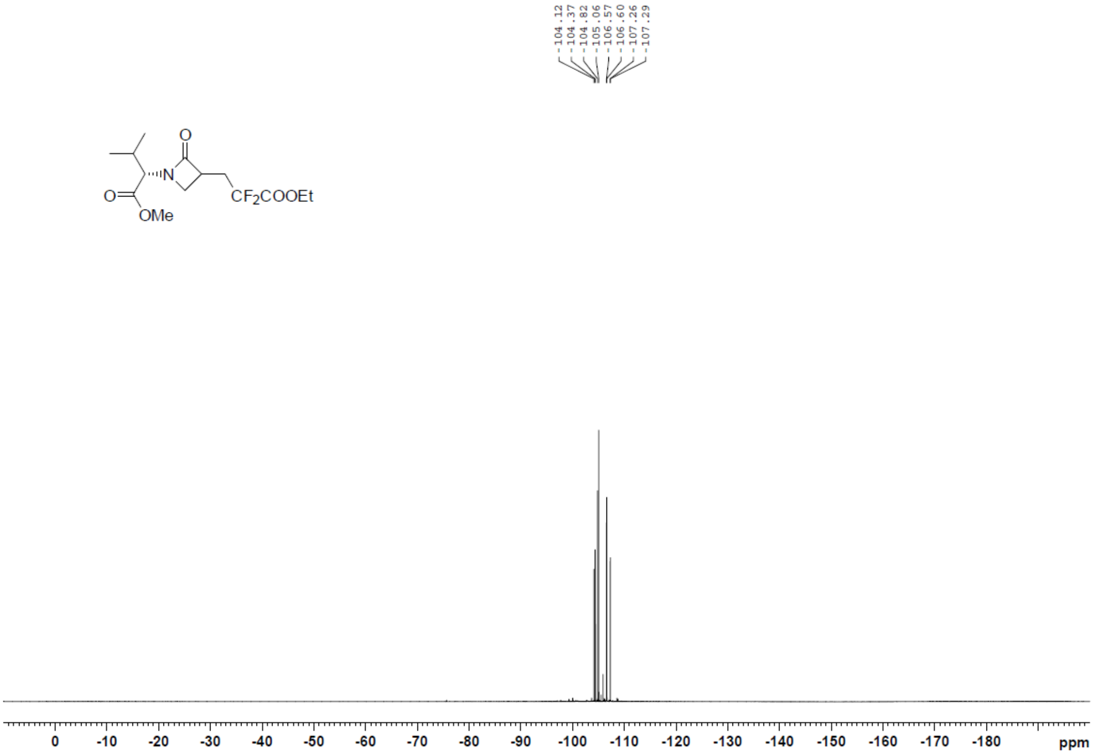

Supplementary Figure 75. ^{19}F NMR spectrum of **3h** in $CDCl_3$ (376 MHz)

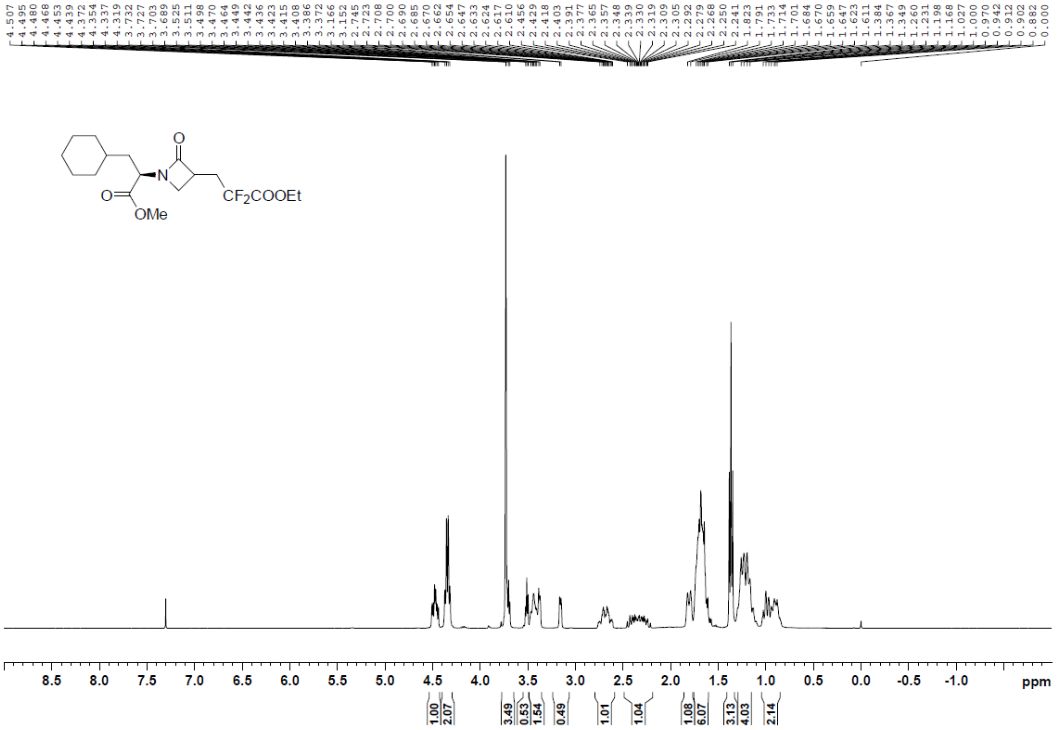

Supplementary Figure 76. ^{1}H NMR spectrum of **3i** in $CDCl_3$ (400 MHz)

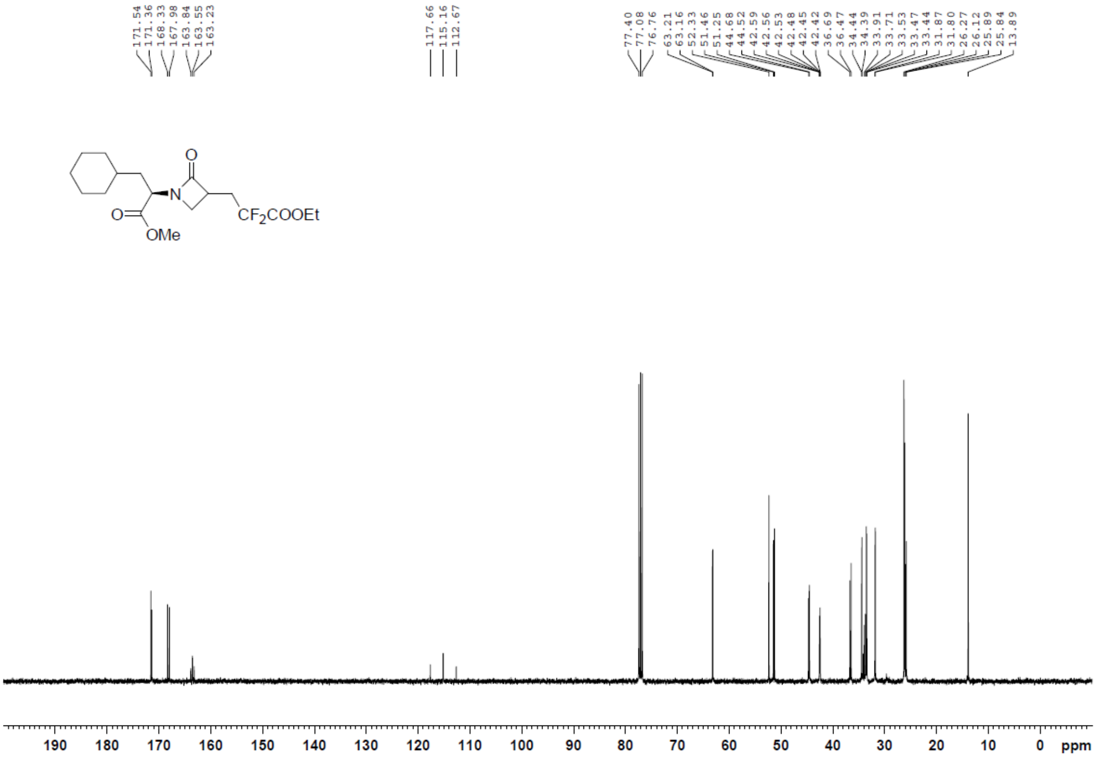

Supplementary Figure 77. ^{13}C NMR spectrum of **3i** in $CDCl_3$ (101 MHz)

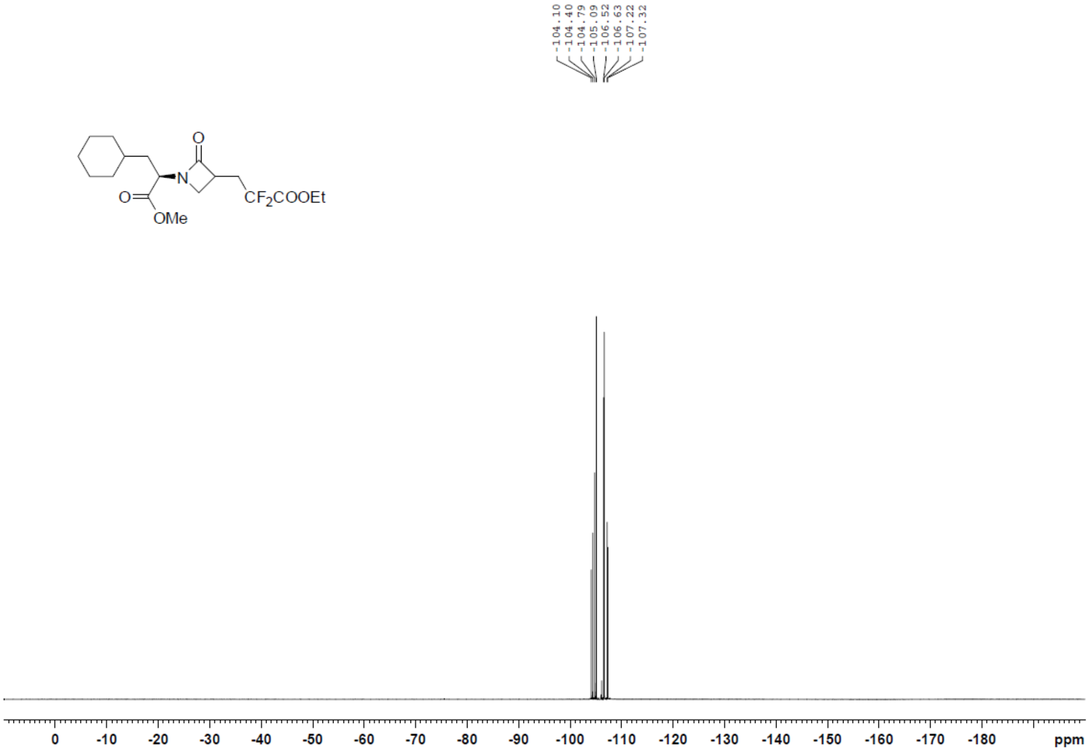

Supplementary Figure 78. ^{19}F NMR spectrum of **3i** in $CDCl_3$ (376 MHz)

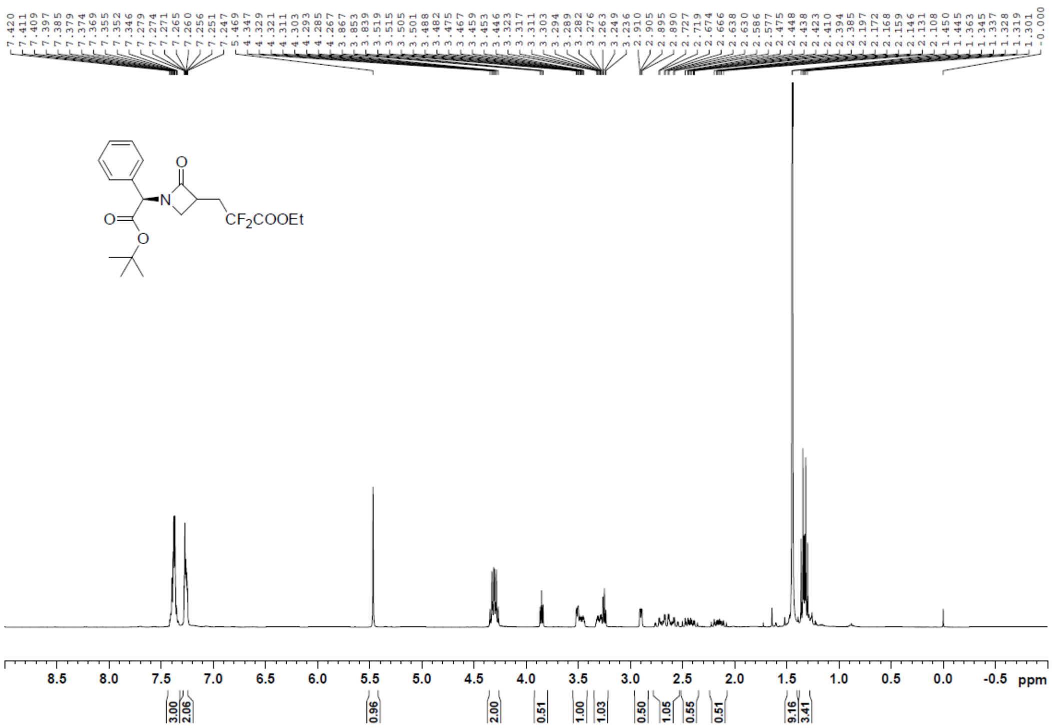

Supplementary Figure 79. ^{1}H NMR spectrum of **3j** in $CDCl_3$ (400 MHz)

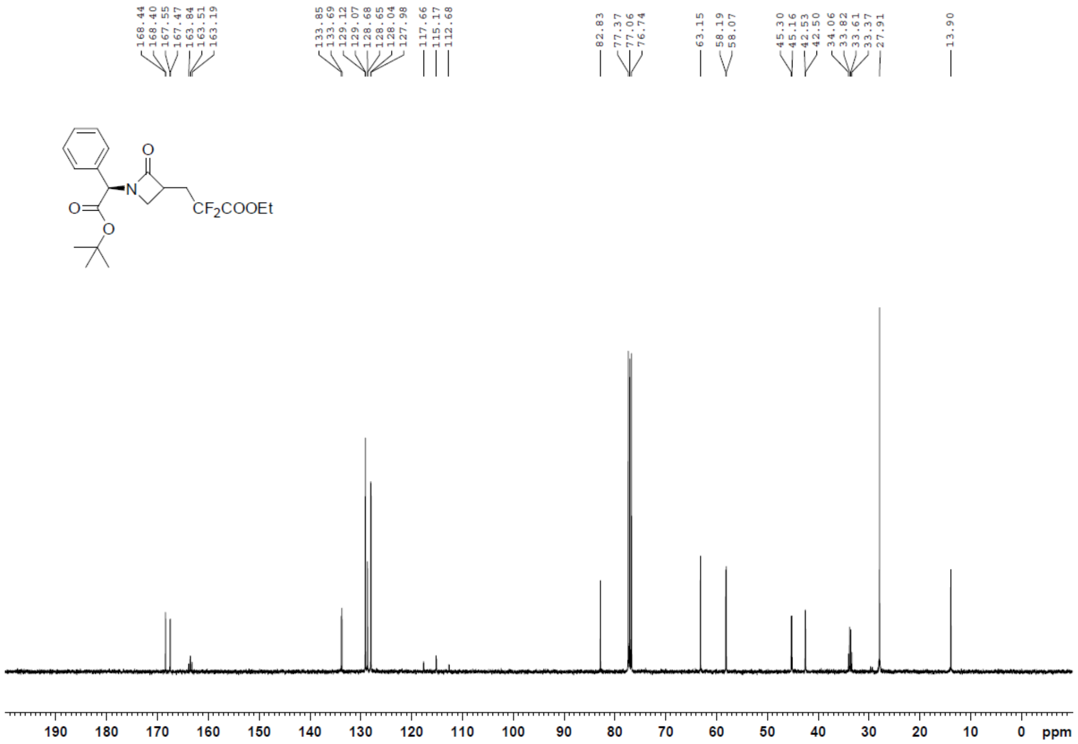

Supplementary Figure 80. ^{13}C NMR spectrum of **3j** in $CDCl_3$ (101 MHz)

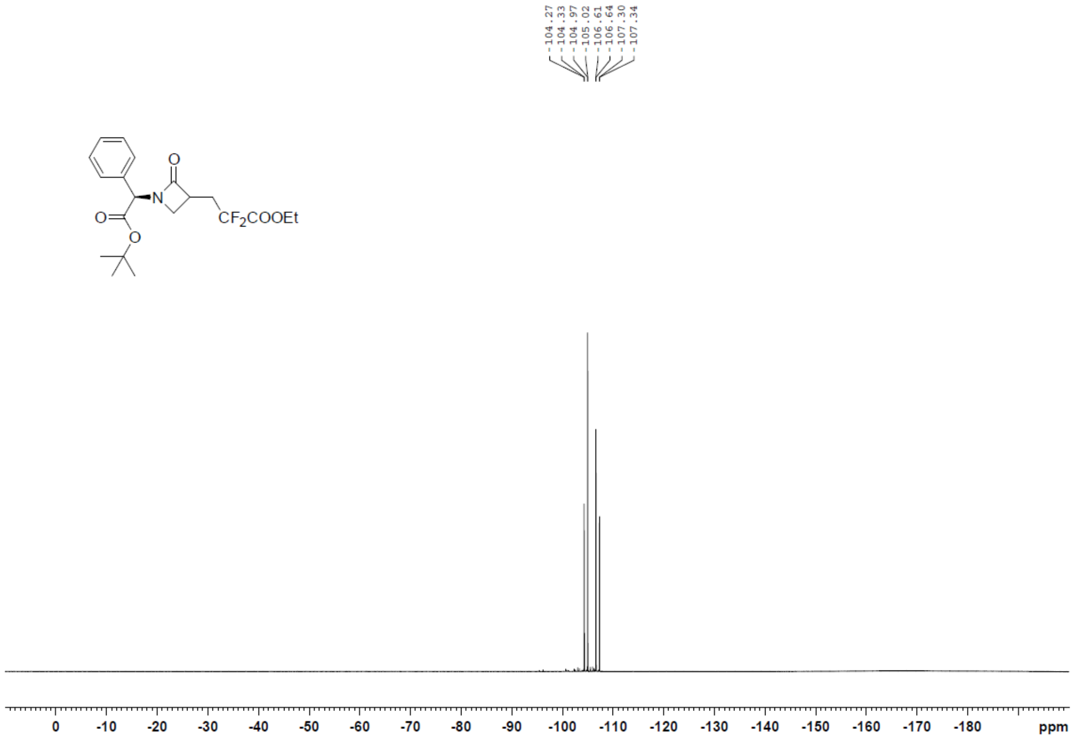

Supplementary Figure 81. ^{19}F NMR spectrum of **3j** in $CDCl_3$ (376 MHz)

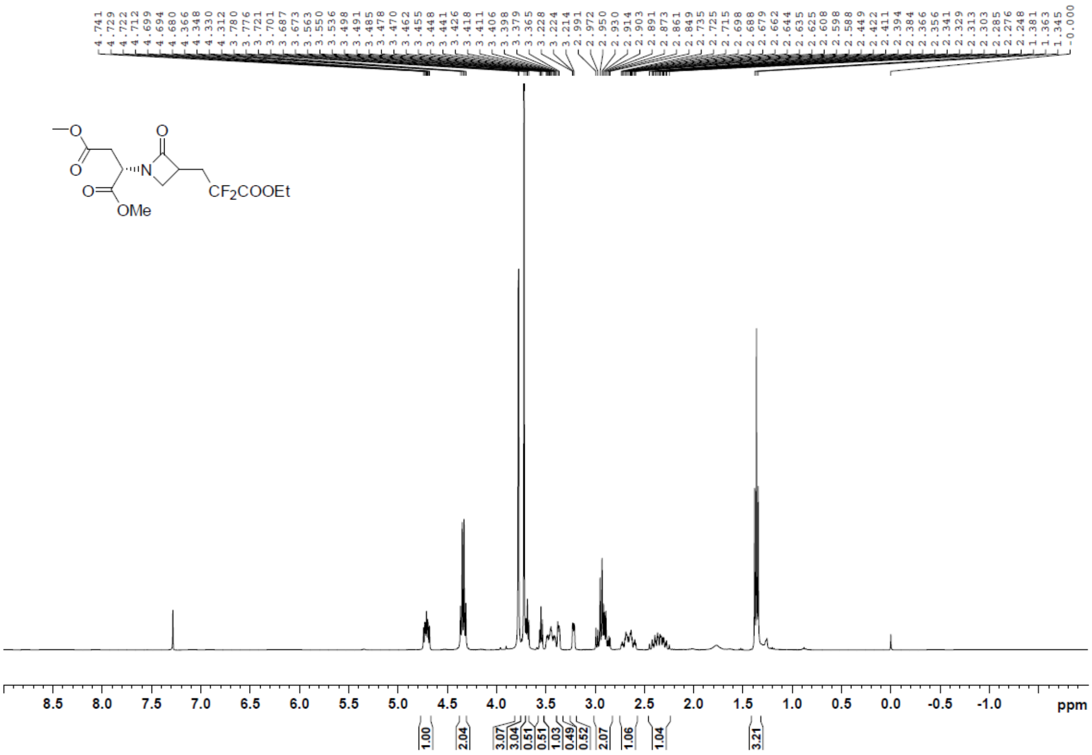

Supplementary Figure 82. ^{1}H NMR spectrum of **3k** in $CDCl_3$ (400 MHz)

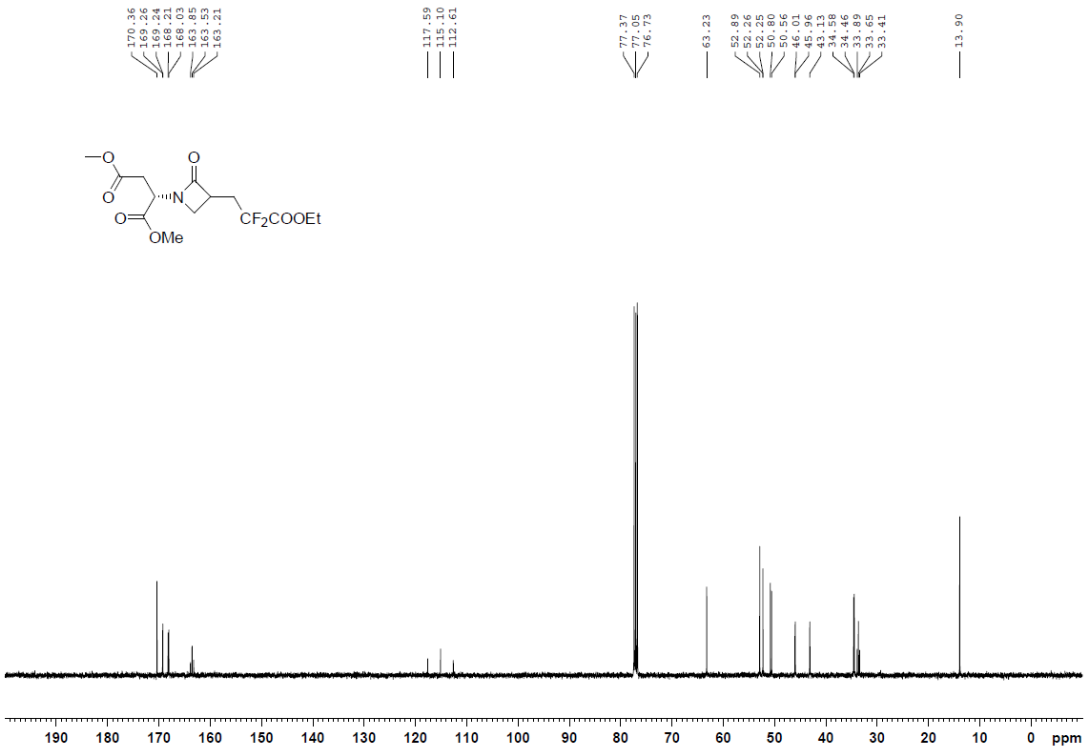

Supplementary Figure 83. ^{13}C NMR spectrum of **3k** in $CDCl_3$ (101 MHz)

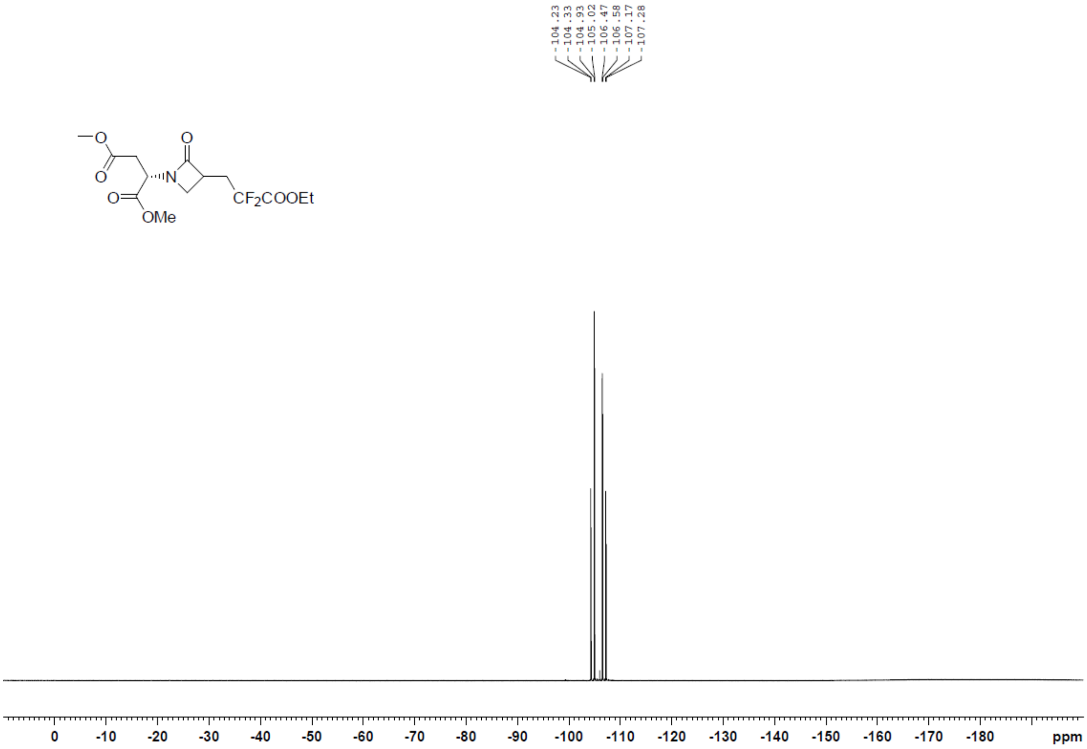

Supplementary Figure 84. ^{19}F NMR spectrum of **3k** in $CDCl_3$ (376 MHz)

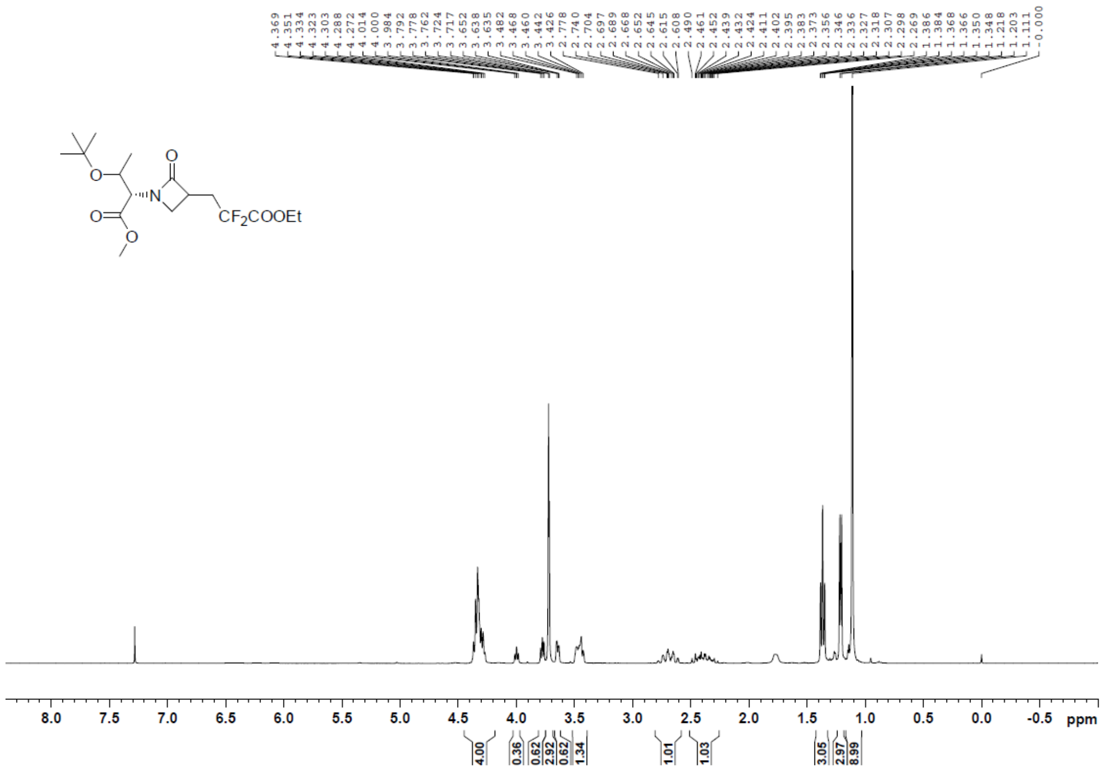

Supplementary Figure 85. ^{1}H NMR spectrum of **3l** in $CDCl_3$ (400 MHz)

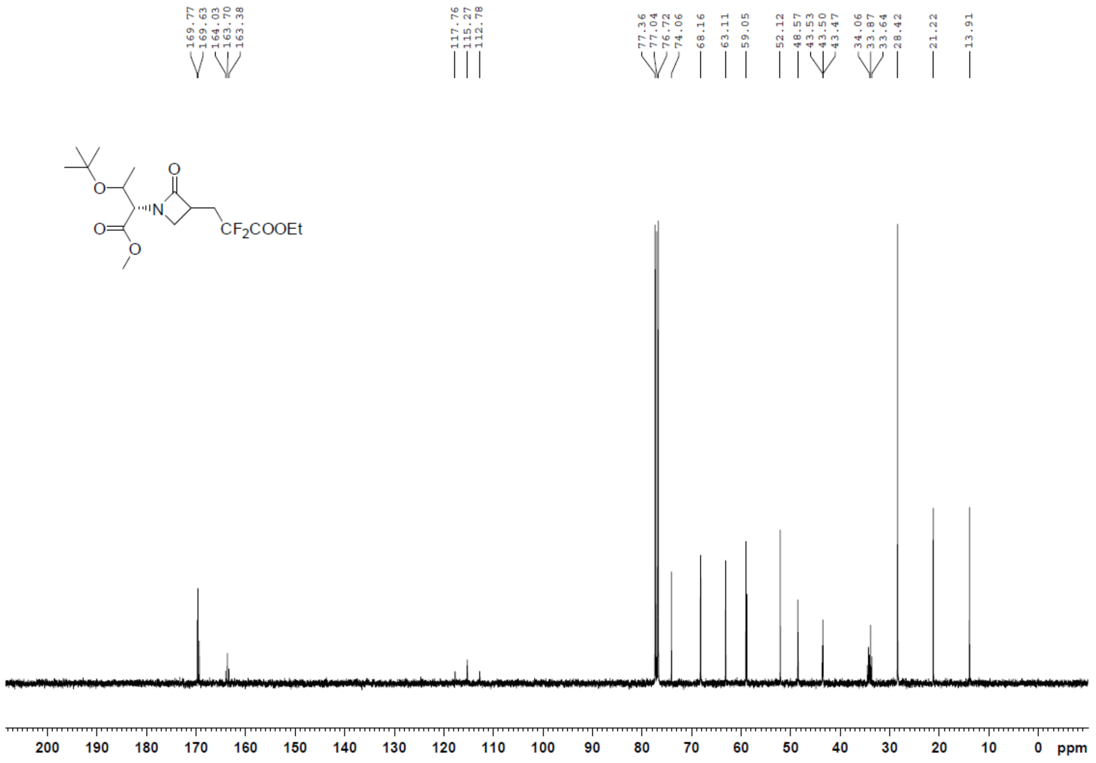

Supplementary Figure 86. ^{13}C NMR spectrum of **3l** in $CDCl_3$ (101 MHz)

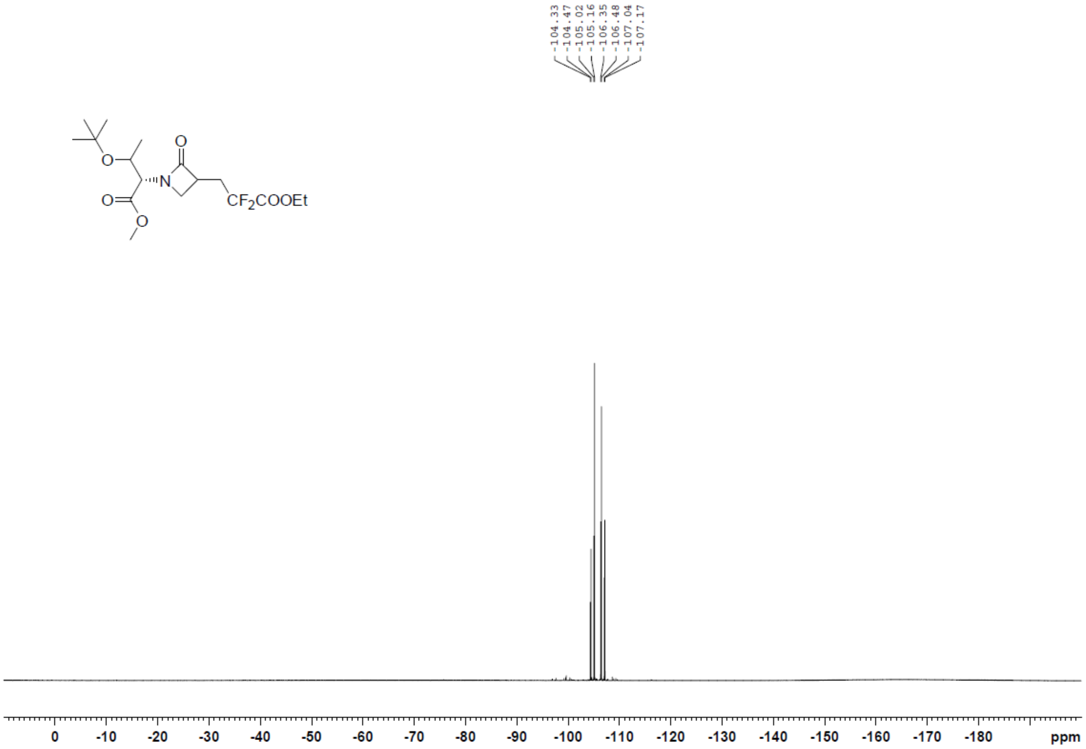

Supplementary Figure 87. ^{19}F NMR spectrum of **3l** in $CDCl_3$ (376 MHz)

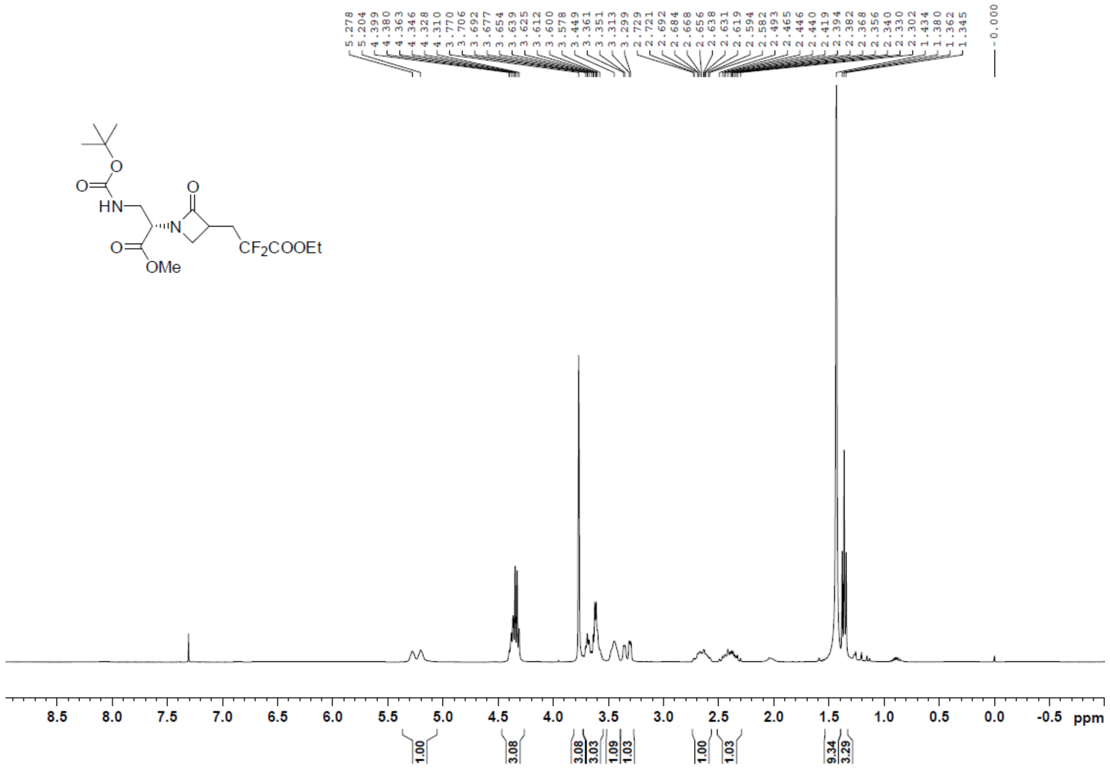

Supplementary Figure 88. ^{1}H NMR spectrum of **3m** in $CDCl_3$ (400 MHz)

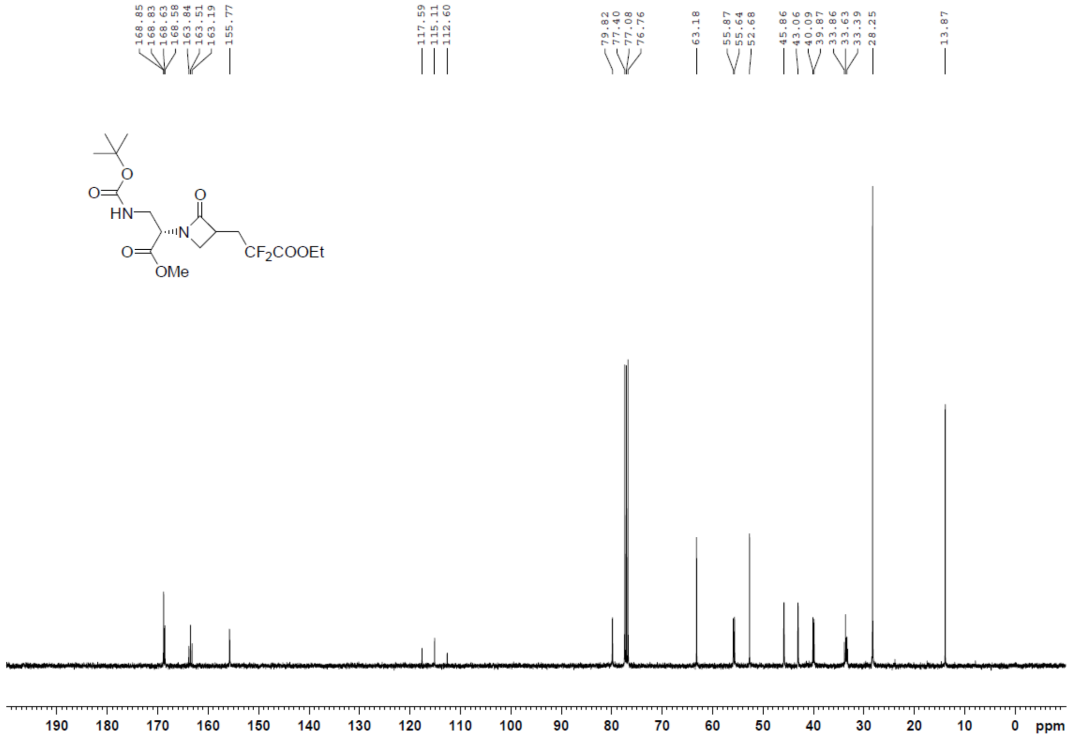

Supplementary Figure 89. ^{13}C NMR spectrum of **3m** in $CDCl_3$ (101 MHz)

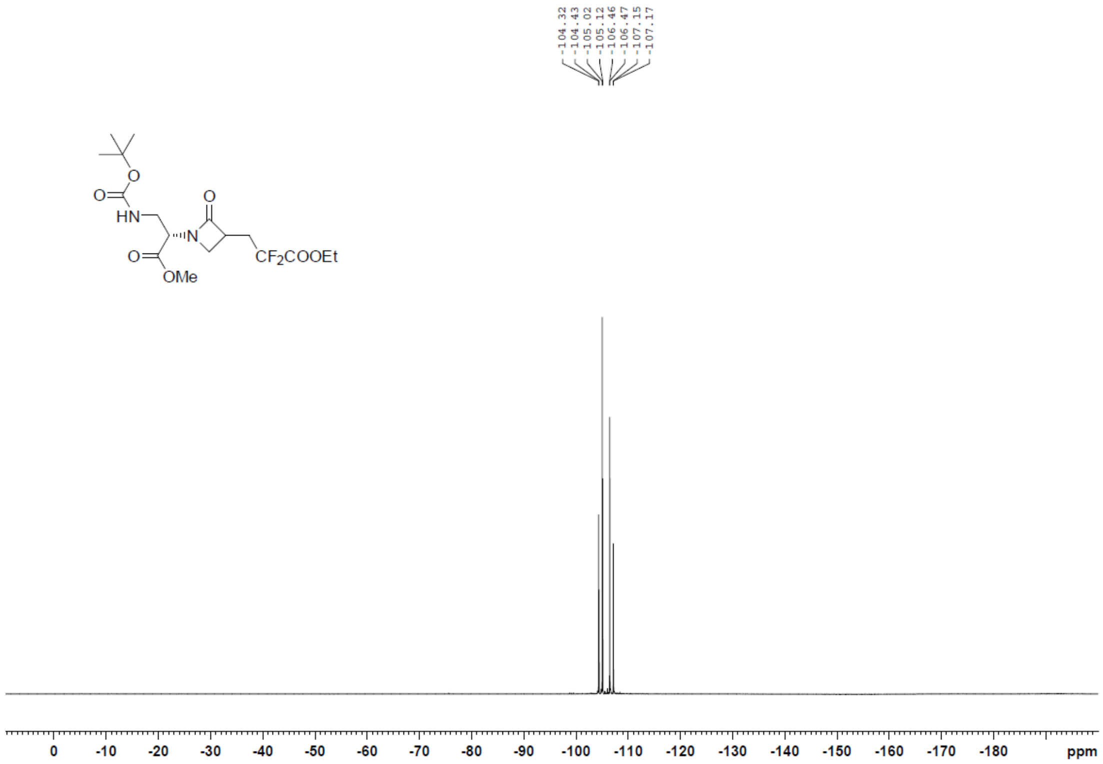

Supplementary Figure 90. ^{19}F NMR spectrum of **3m** in $CDCl_3$ (376 MHz)

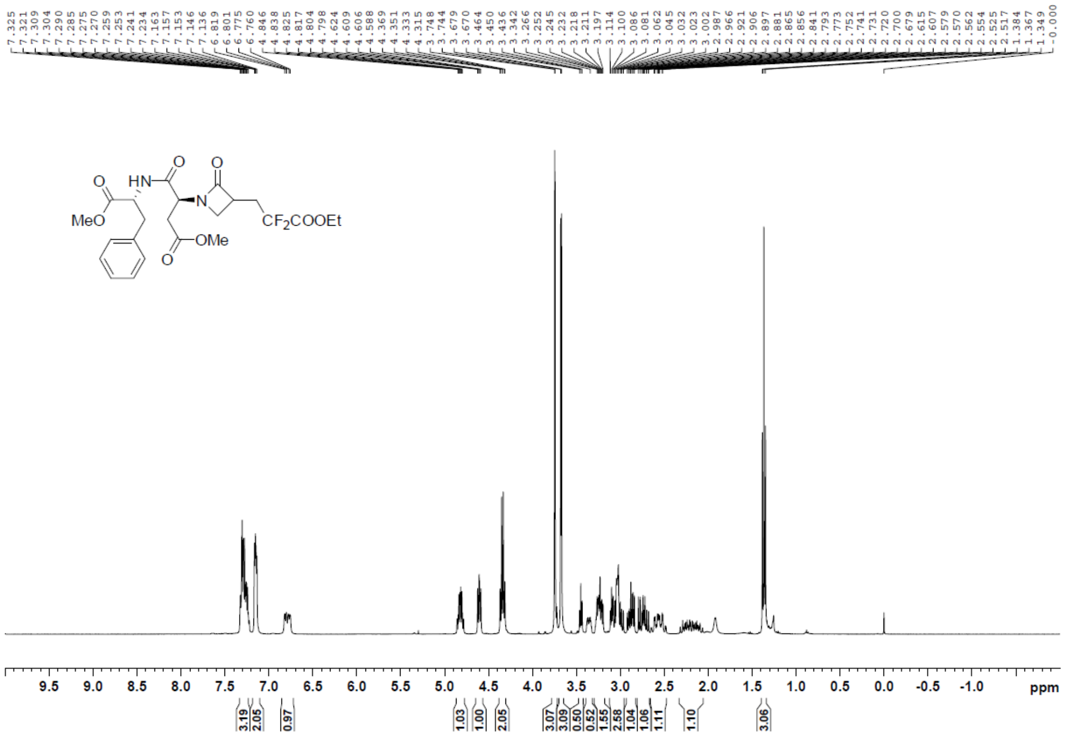

Supplementary Figure 91. ^{1}H NMR spectrum of **3n** in $CDCl_3$ (400 MHz)

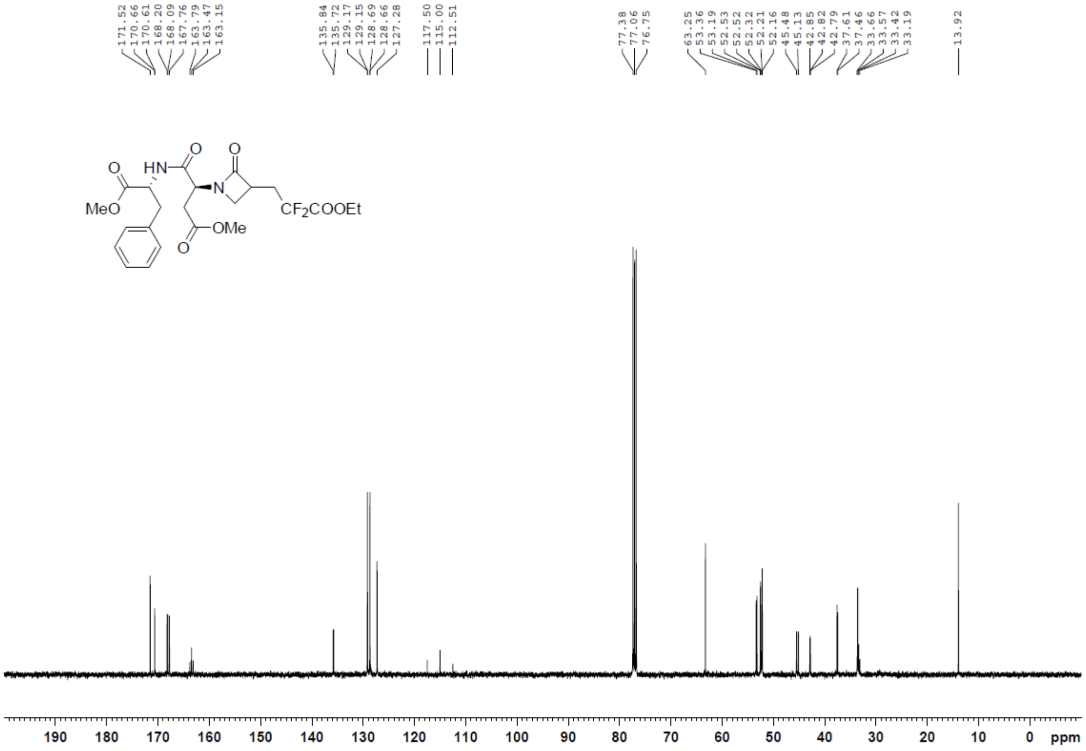

Supplementary Figure 92. ^{13}C NMR spectrum of **3n** in $CDCl_3$ (101 MHz)

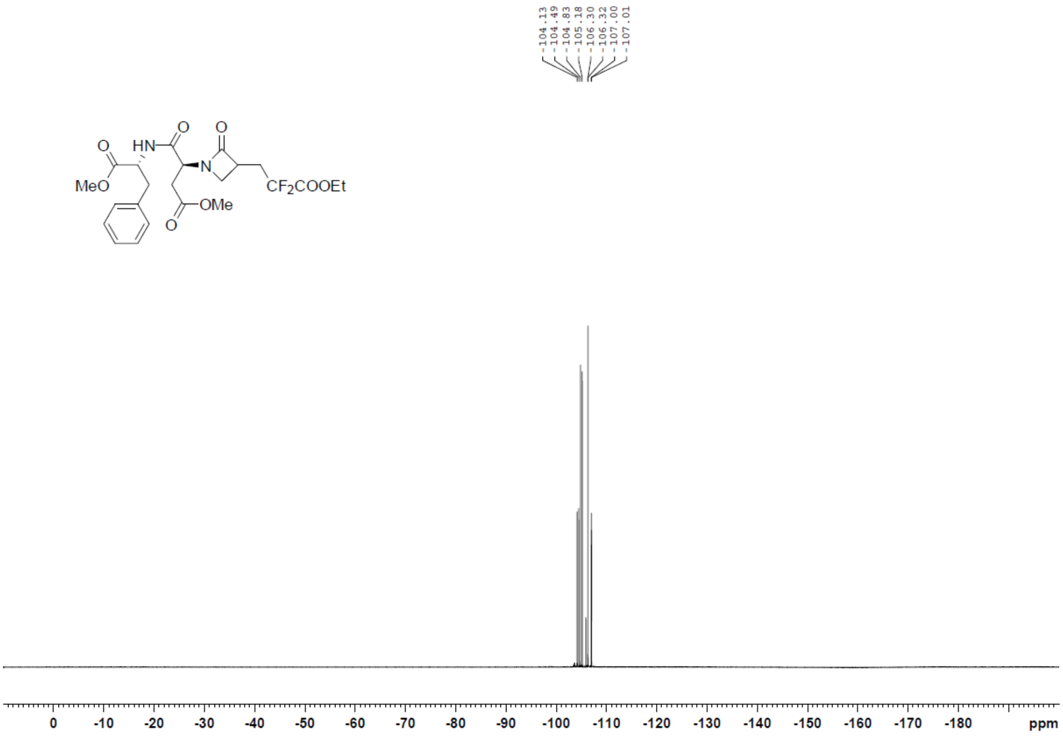

Supplementary Figure 93. ^{19}F NMR spectrum of **3n** in $CDCl_3$ (376 MHz)

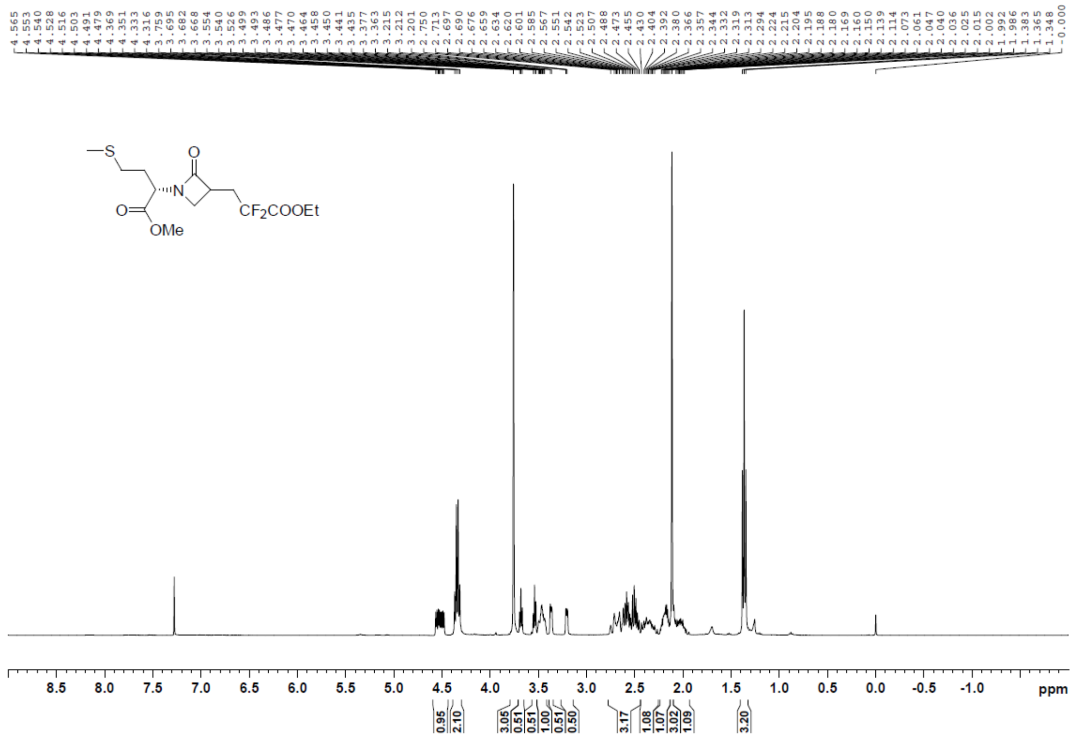

Supplementary Figure 94. ^{1}H NMR spectrum of **3o** in $CDCl_3$ (400 MHz)

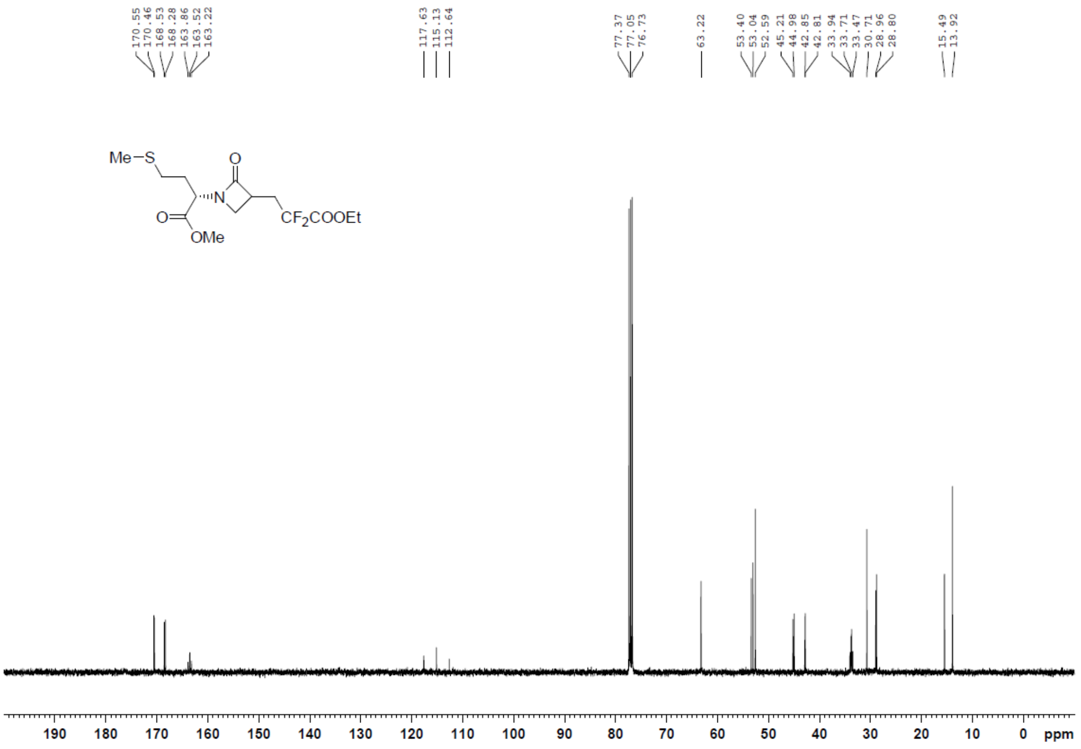

Supplementary Figure 95. ^{13}C NMR spectrum of **3o** in $CDCl_3$ (101 MHz)

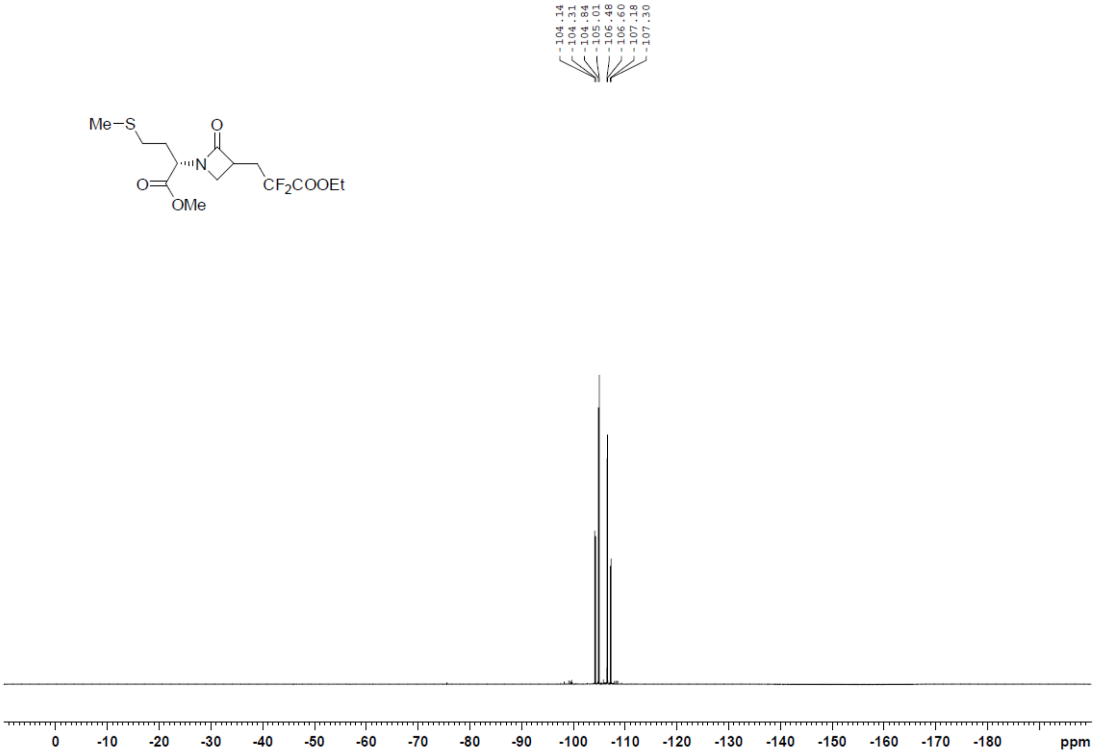

Supplementary Figure 96. ^{19}F NMR spectrum of **3o** in $CDCl_3$ (376 MHz)

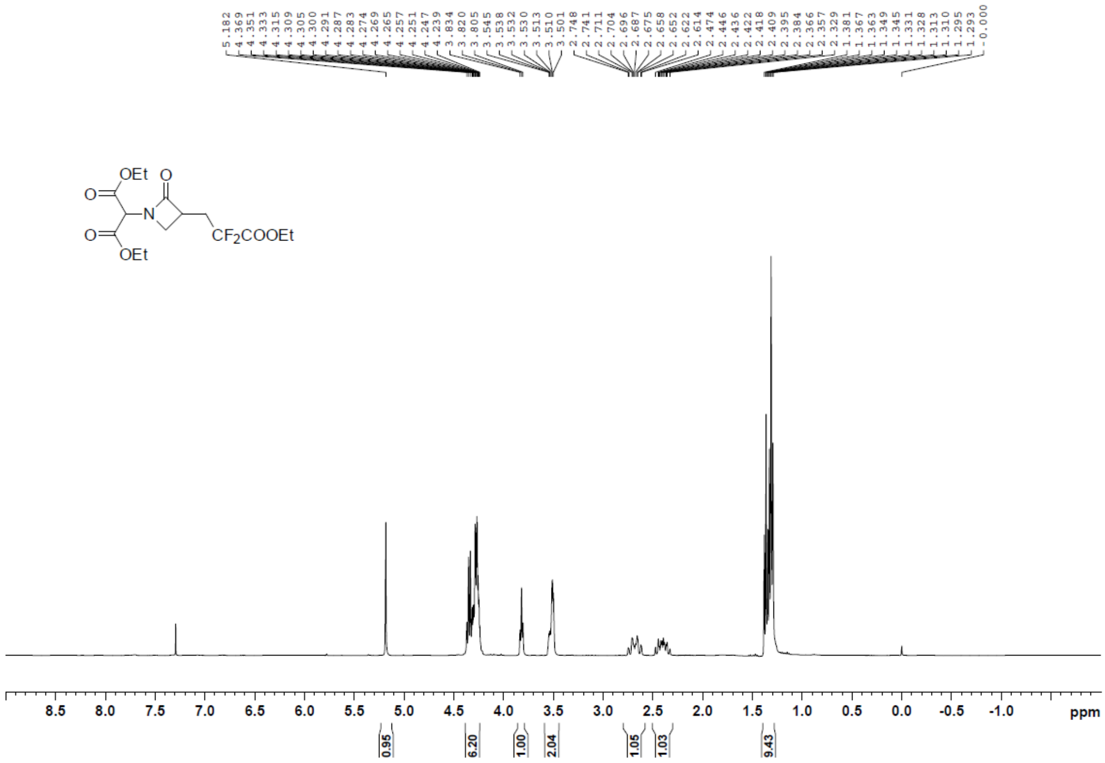

Supplementary Figure 97. ^{1}H NMR spectrum of **3p** in $CDCl_3$ (400 MHz)

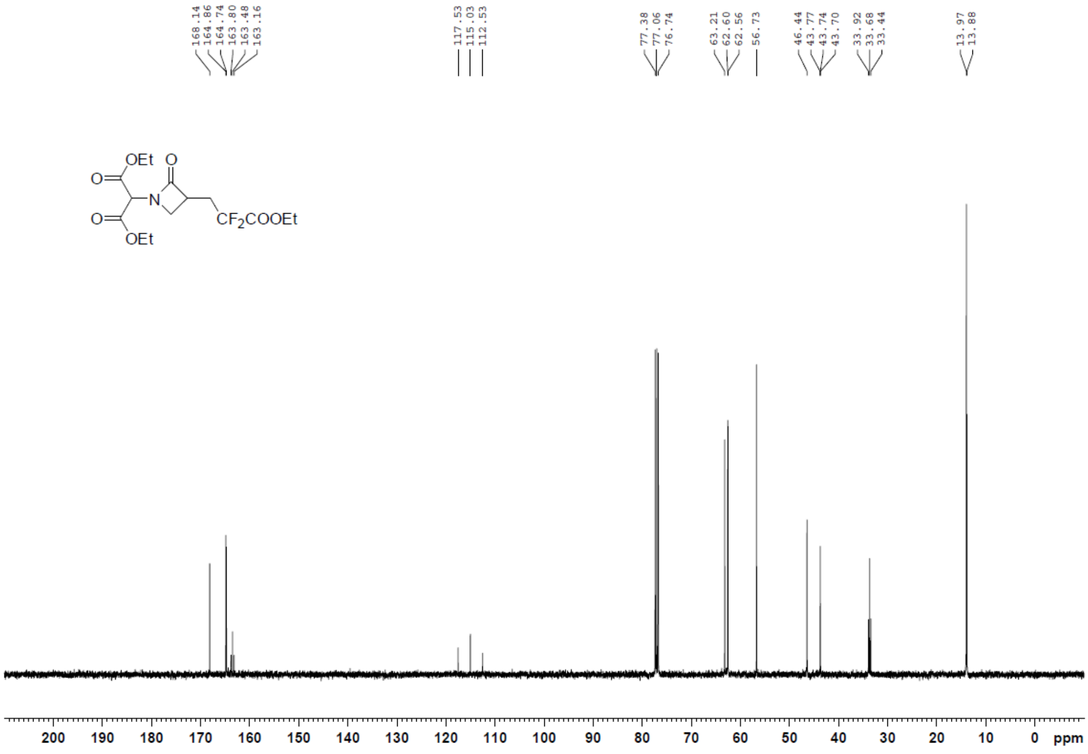

Supplementary Figure 98. ^{13}C NMR spectrum of **3p** in $CDCl_3$ (101 MHz)

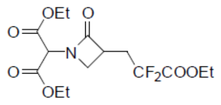

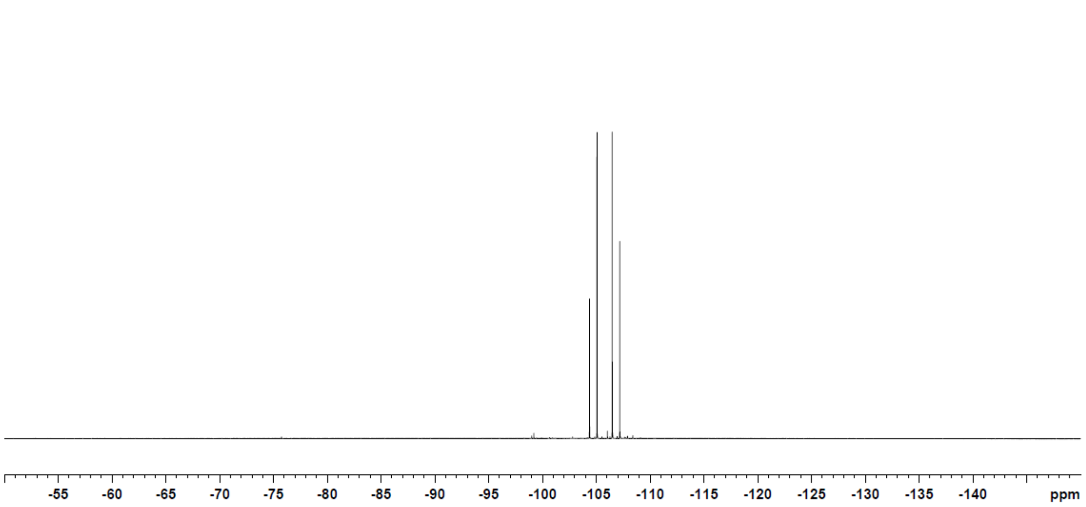

Supplementary Figure 99. ^{19}F NMR spectrum of **3p** in $CDCl_3$ (376 MHz)

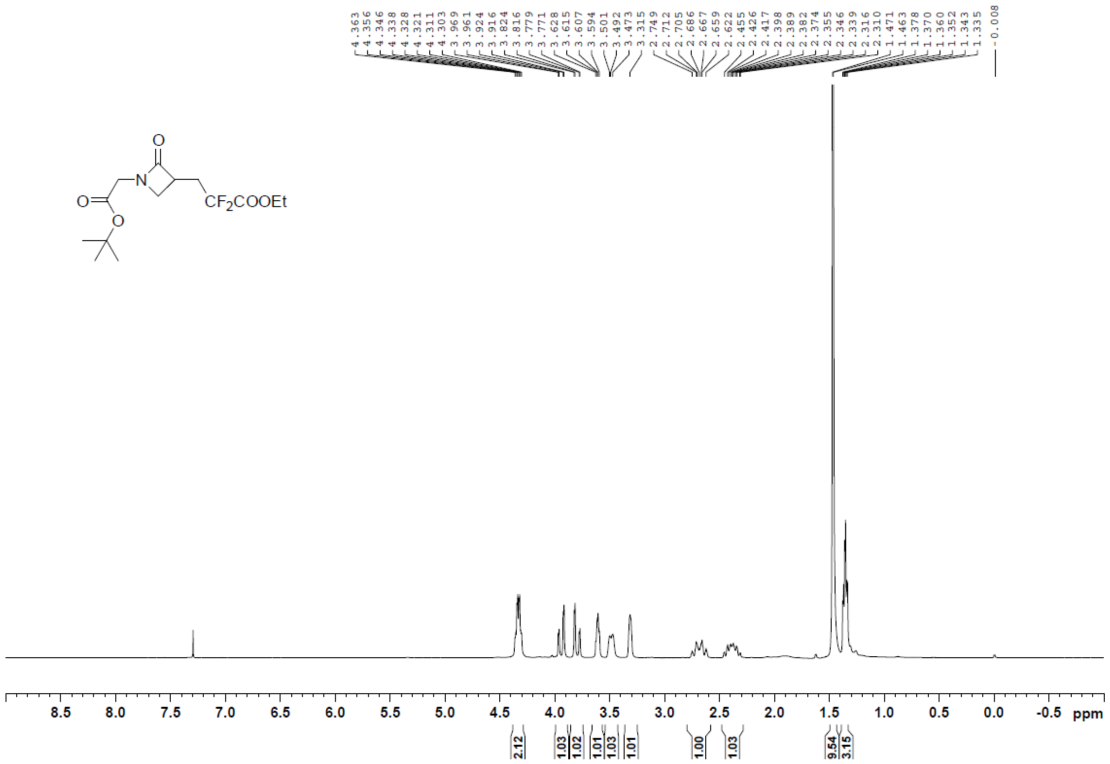

Supplementary Figure 100. ^{1}H NMR spectrum of **3q** in $CDCl_3$ (400 MHz)

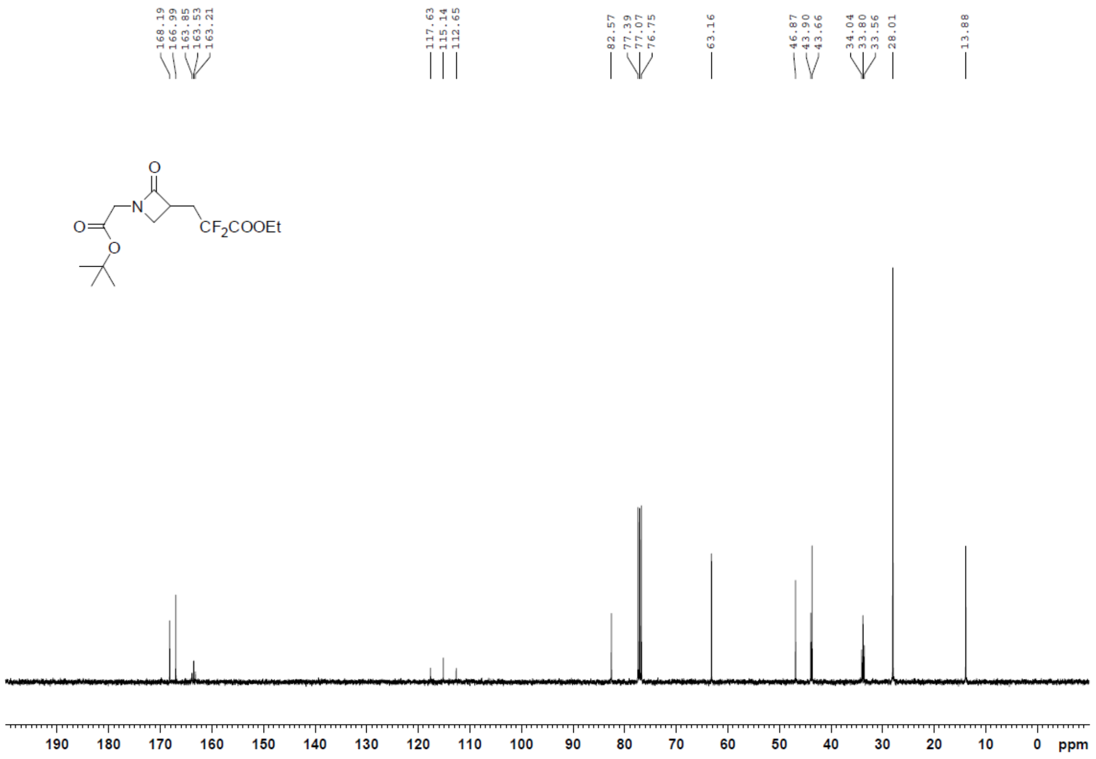

Supplementary Figure 101. ^{13}C NMR spectrum of **3q** in $CDCl_3$ (101 MHz)

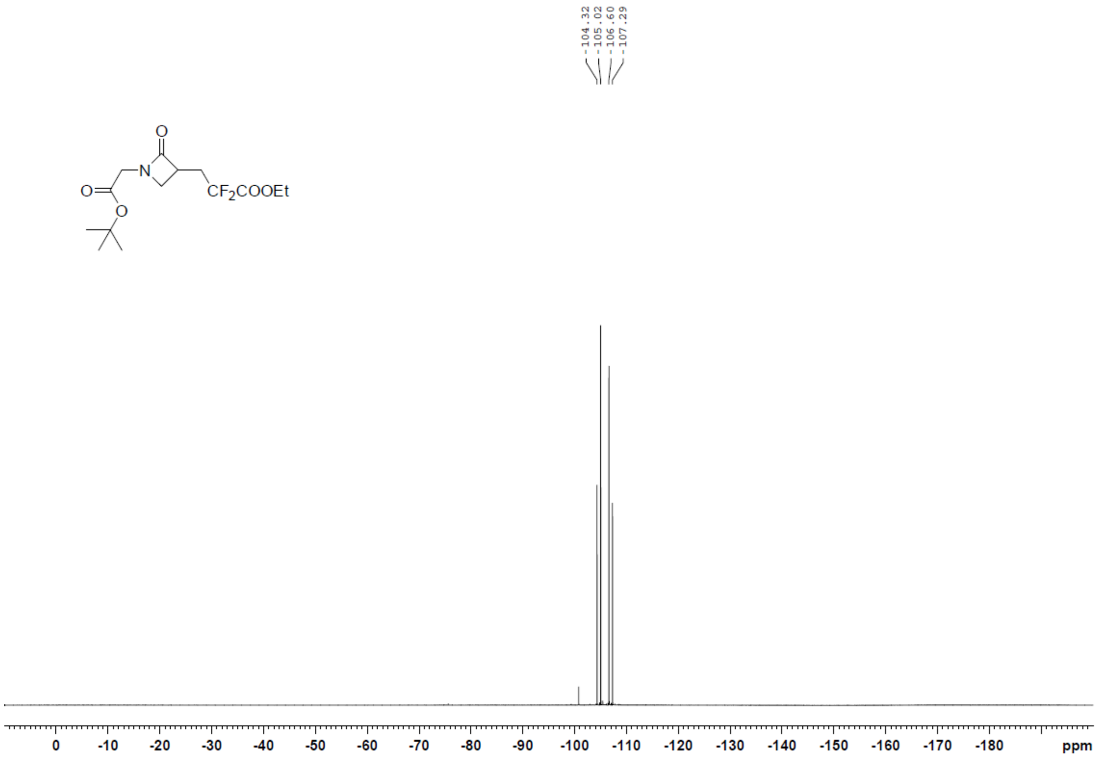

Supplementary Figure 102. ^{19}F NMR spectrum of **3q** in $CDCl_3$ (376 MHz)

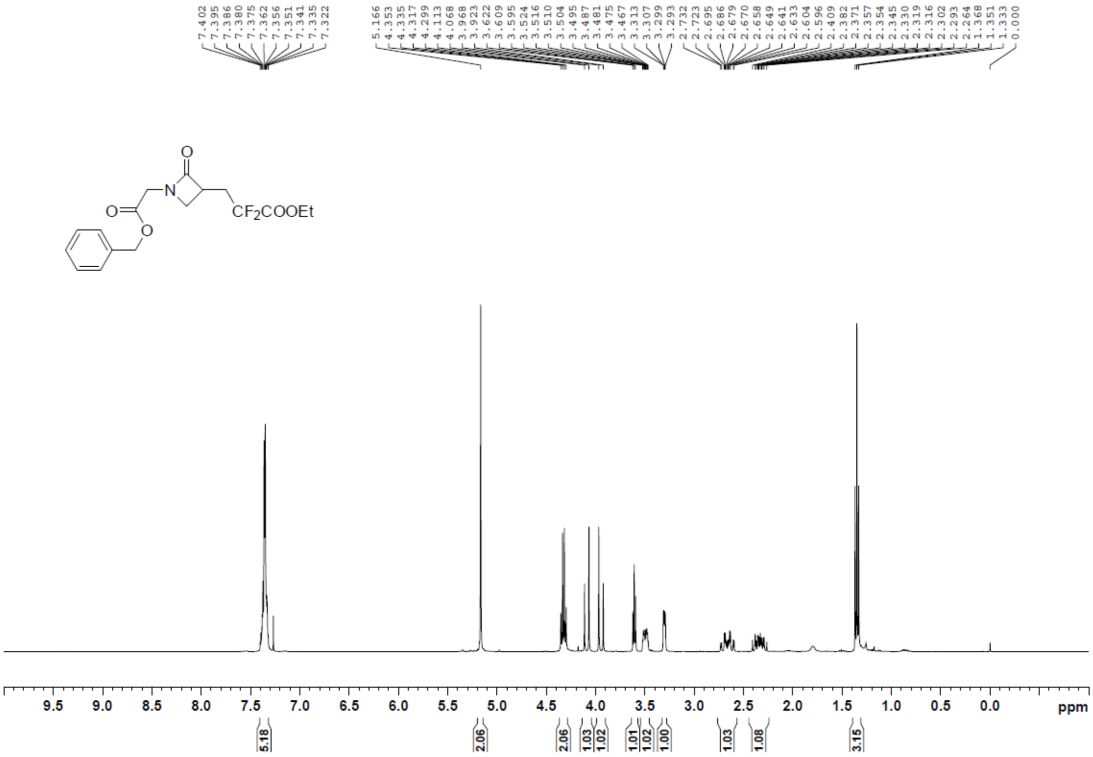

Supplementary Figure 103. ^{1}H NMR spectrum of **3r** in $CDCl_3$ (400 MHz)

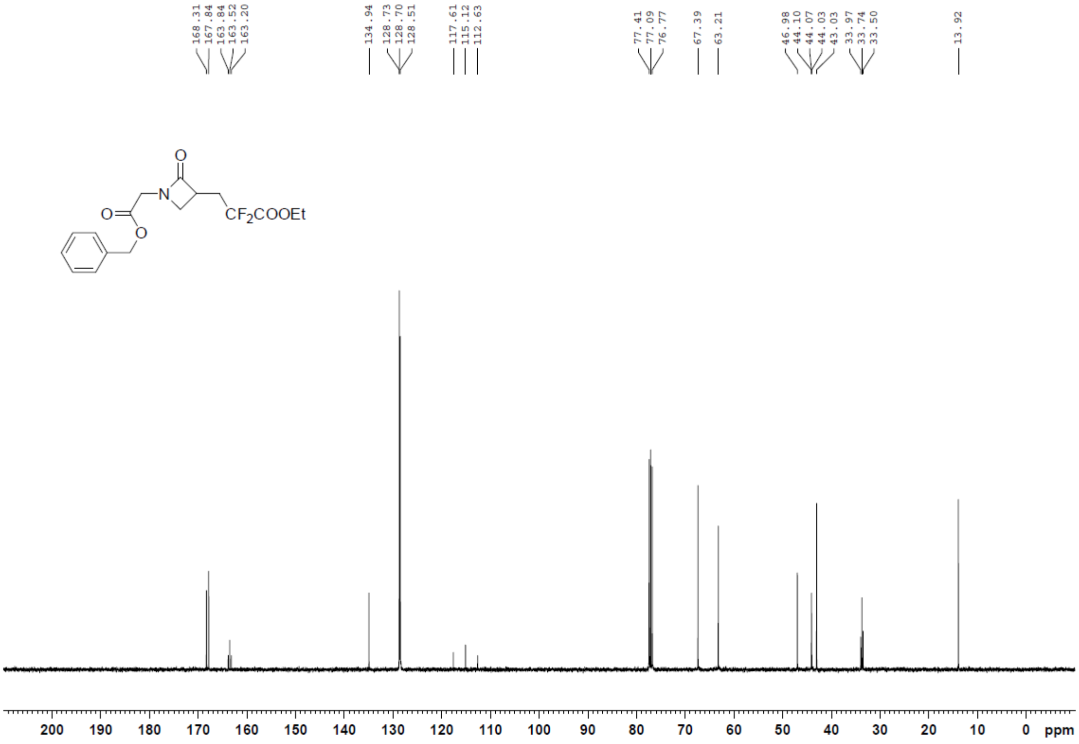

Supplementary Figure 104. ^{13}C NMR spectrum of **3r** in $CDCl_3$ (101 MHz)

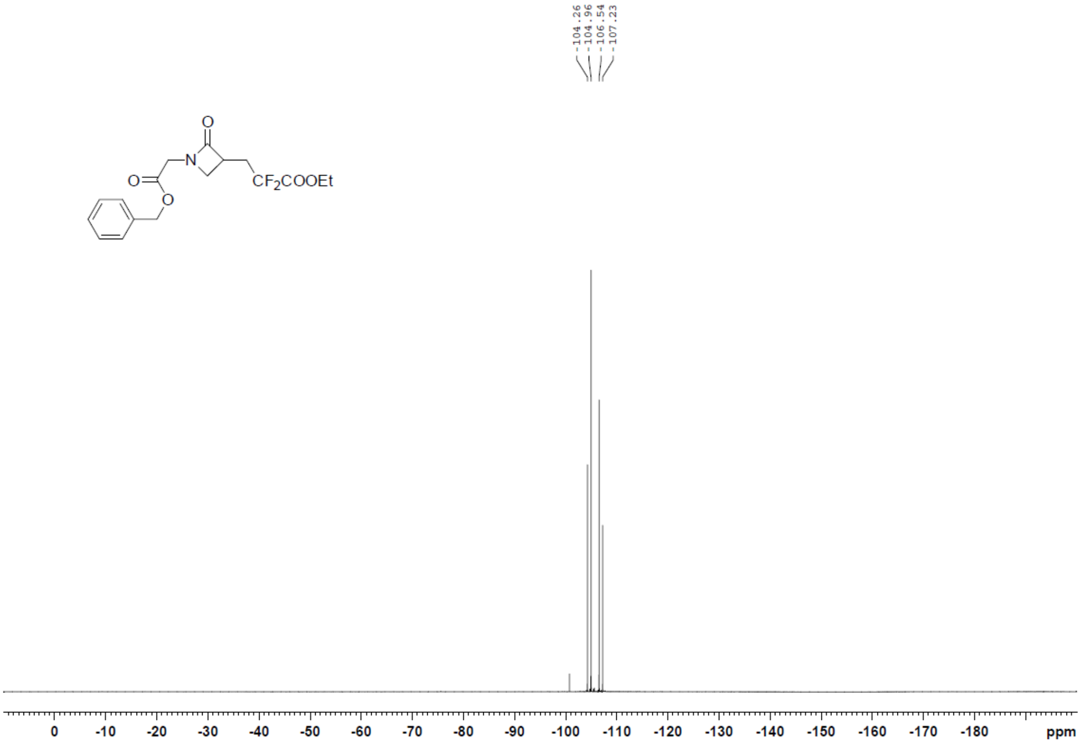

Supplementary Figure 105. ^{19}F NMR spectrum of **3r** in $CDCl_3$ (376 MHz)

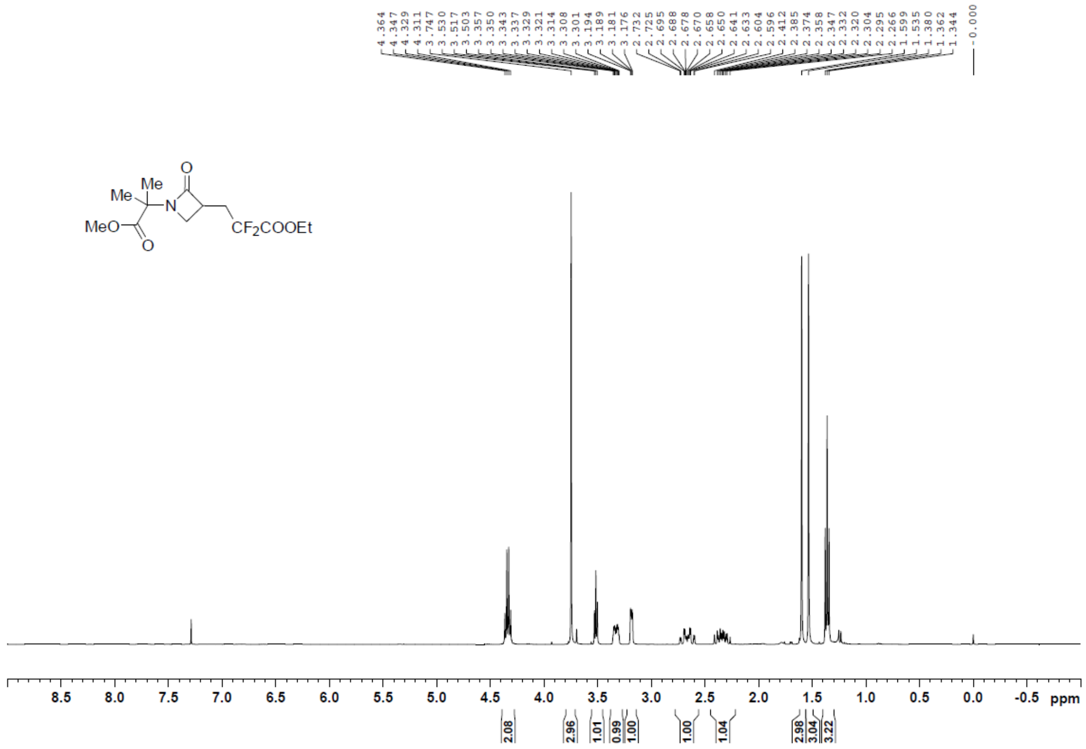

Supplementary Figure 106. ^{1}H NMR spectrum of **3s** in $CDCl_3$ (400 MHz)

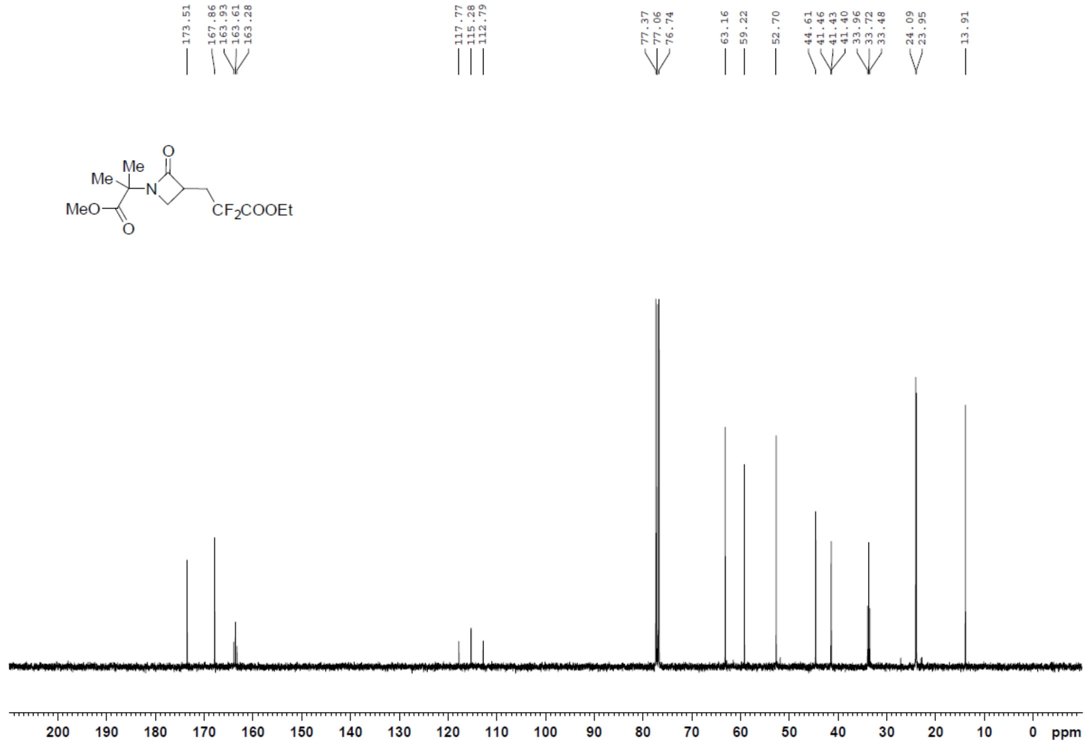

Supplementary Figure 107. ^{13}C NMR spectrum of **3s** in $CDCl_3$ (101 MHz)

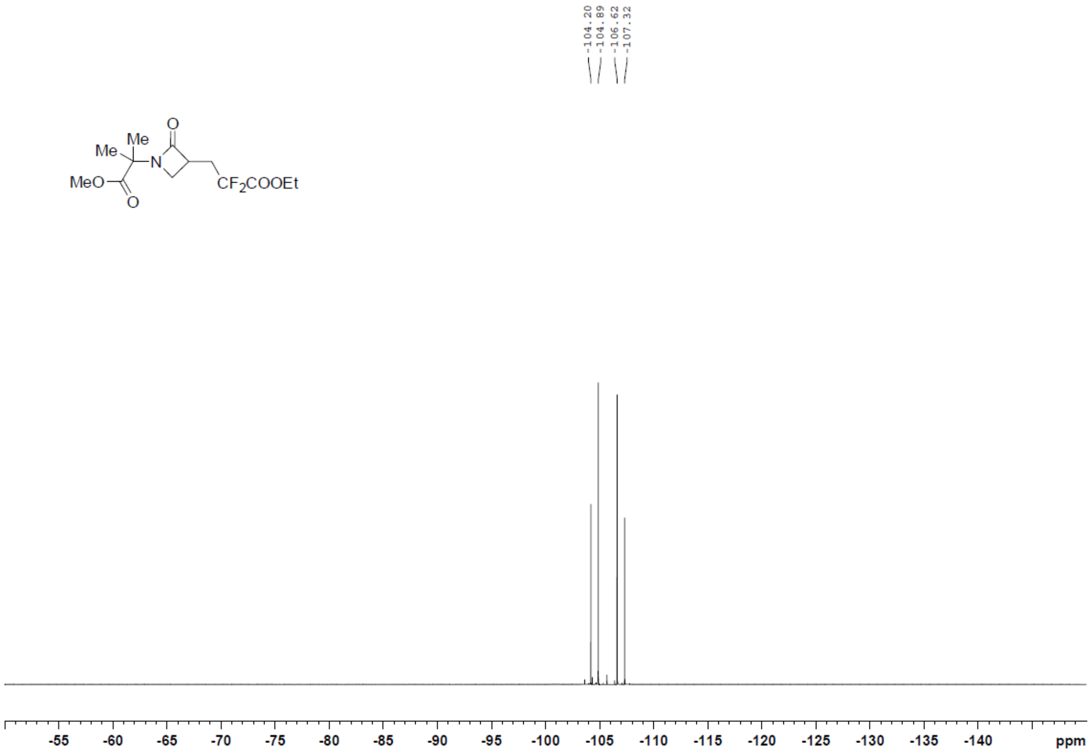

Supplementary Figure 108. ^{19}F NMR spectrum of **3s** in $CDCl_3$ (376 MHz)

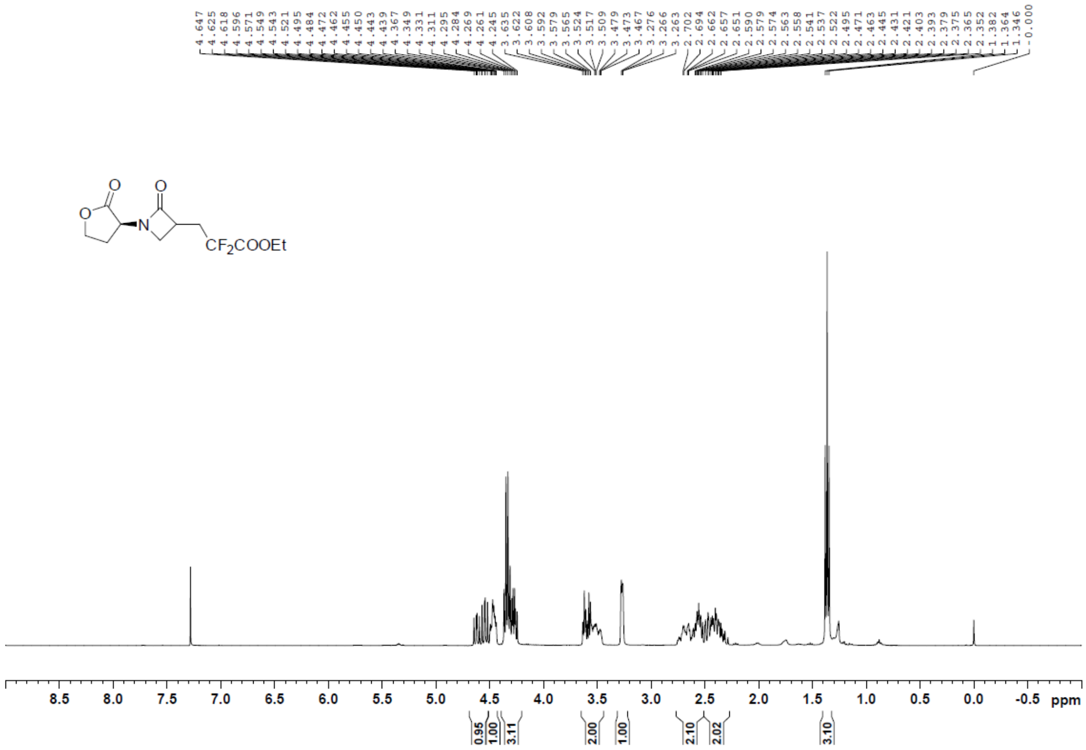

Supplementary Figure109. ^{1}H NMR spectrum of **3t** in $CDCl_3$ (400 MHz)

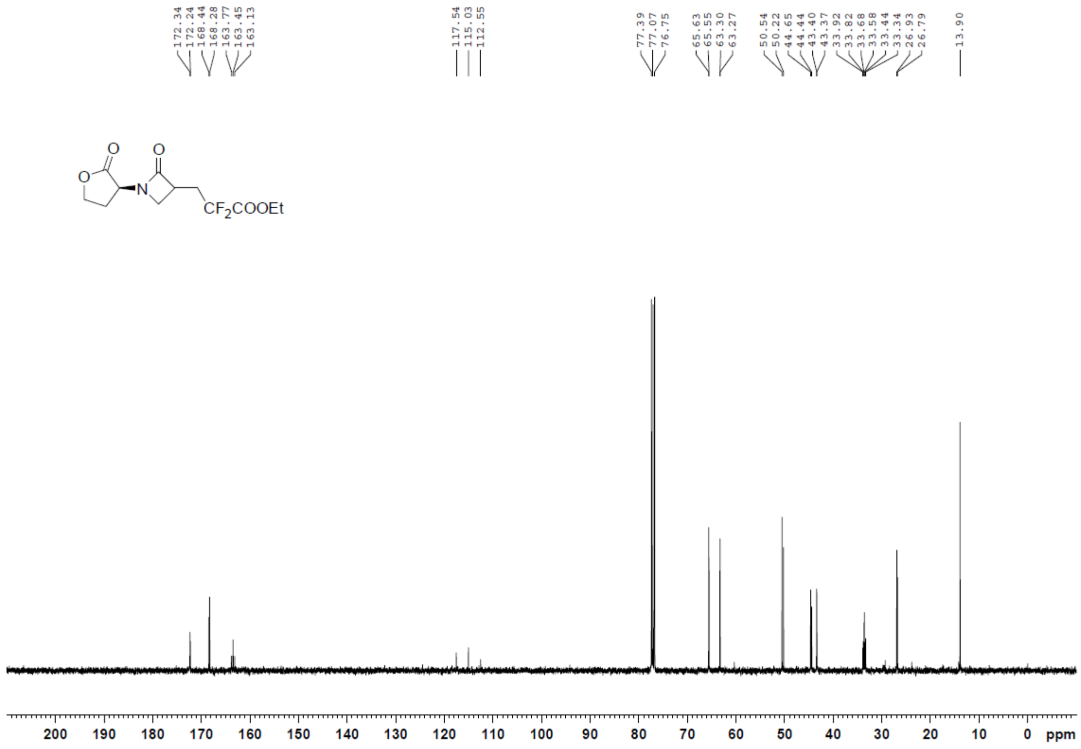

Supplementary Figure 110. ^{13}C NMR spectrum of **3t** in $CDCl_3$ (101 MHz)

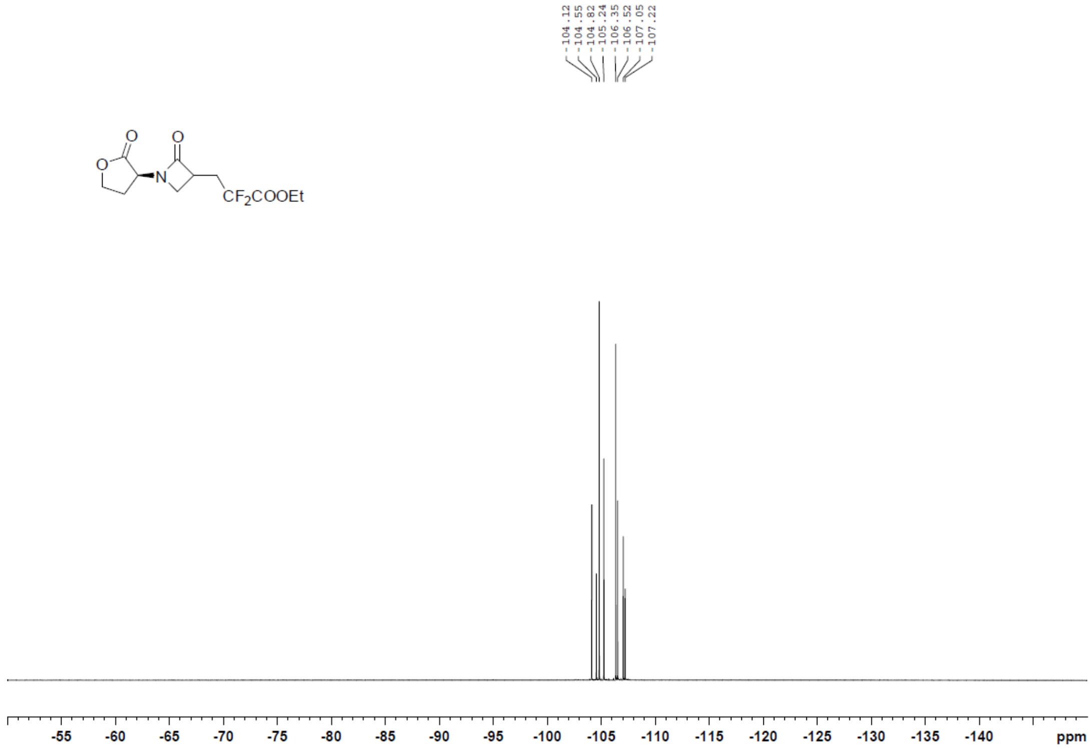

Supplementary Figure 111. ^{19}F NMR spectrum of **3t** in $CDCl_3$ (376 MHz)

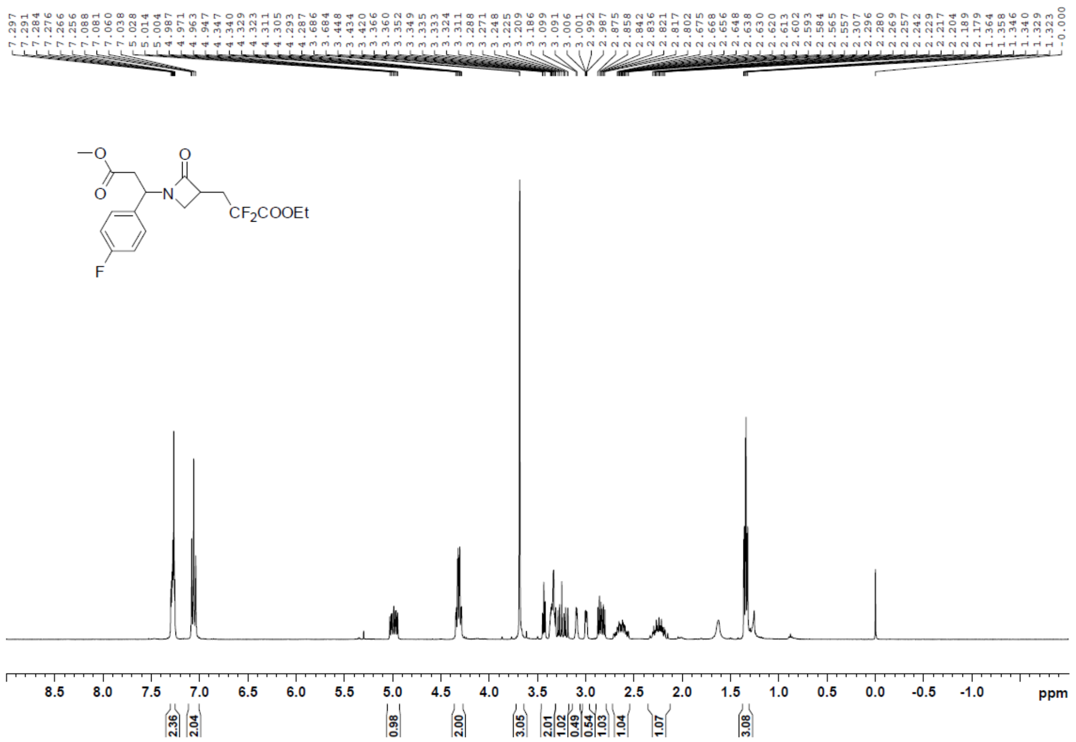

Supplementary Figure 112. ^{1}H NMR spectrum of **3u** in $CDCl_3$ (400 MHz)

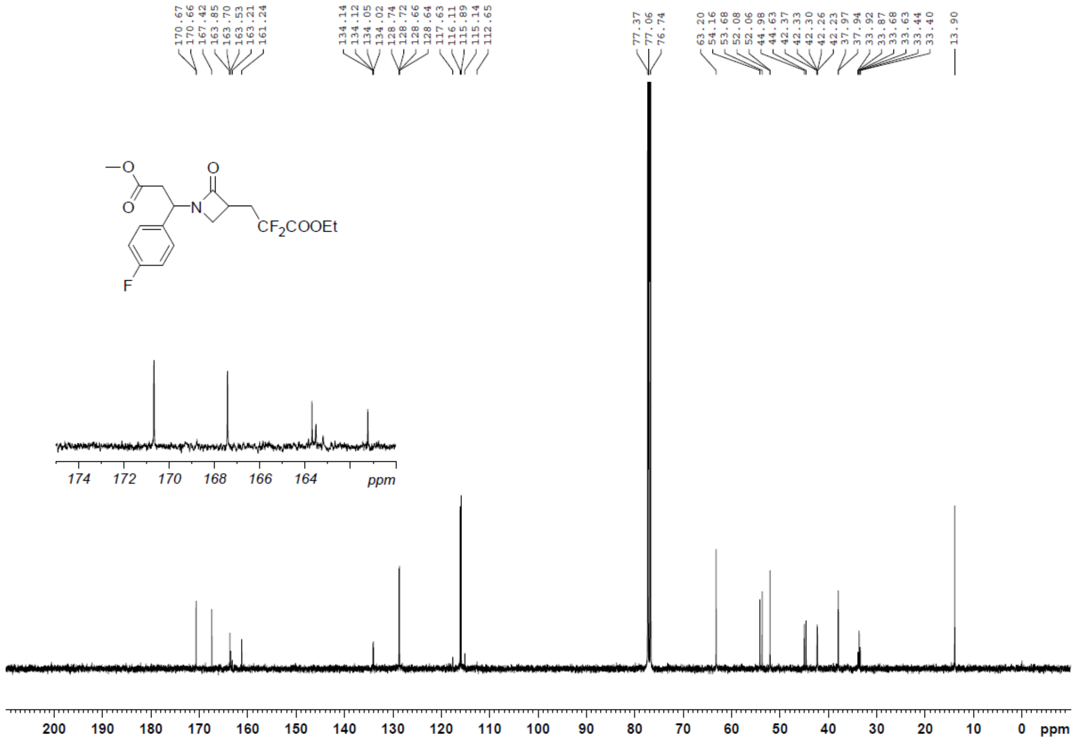

Supplementary Figure 113. ^{13}C NMR spectrum of **3u** in $CDCl_3$ (101 MHz)

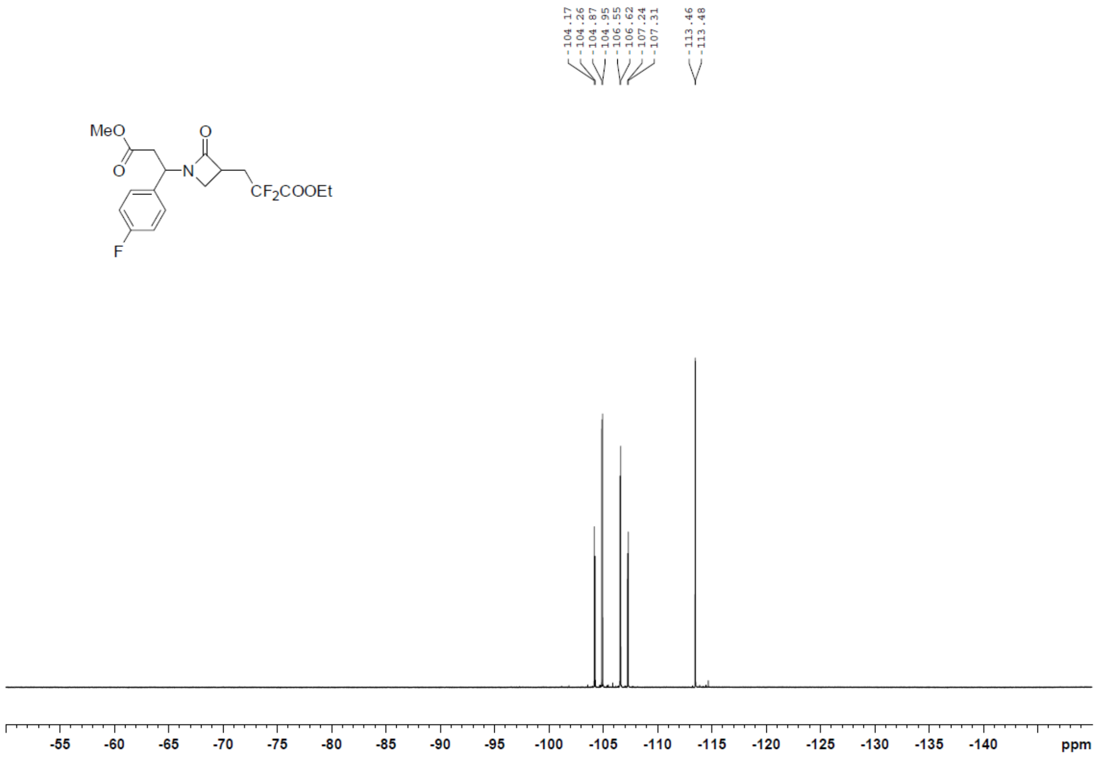

Supplementary Figure 114. ^{19}F NMR spectrum of **3u** in $CDCl_3$ (376 MHz)

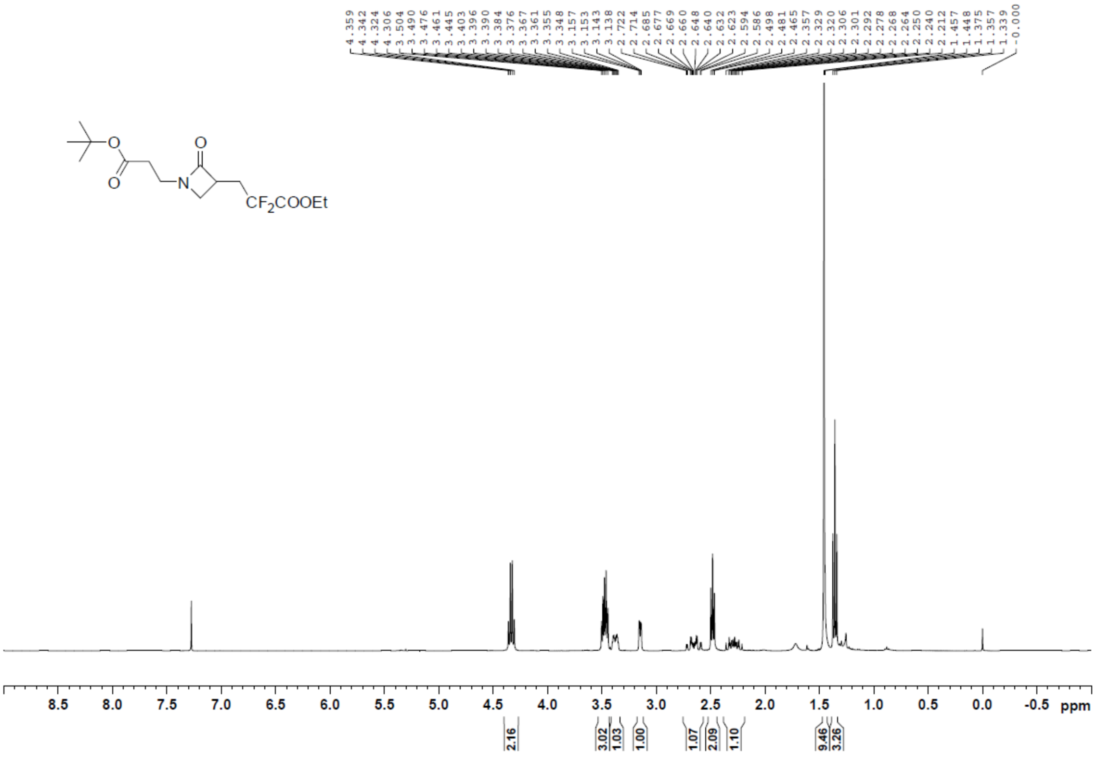

Supplementary Figure 115. ^{1}H NMR spectrum of **3v** in $CDCl_3$ (400 MHz)

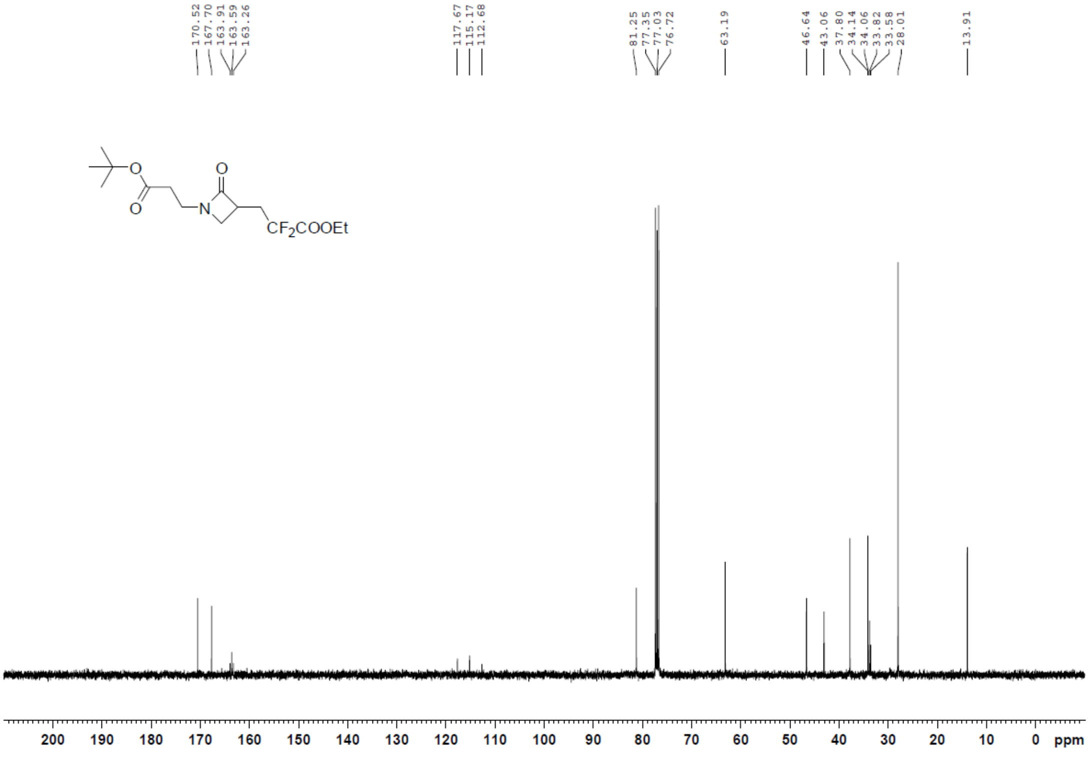

Supplementary Figure 116. ^{13}C NMR spectrum of **3v** in $CDCl_3$ (101 MHz)

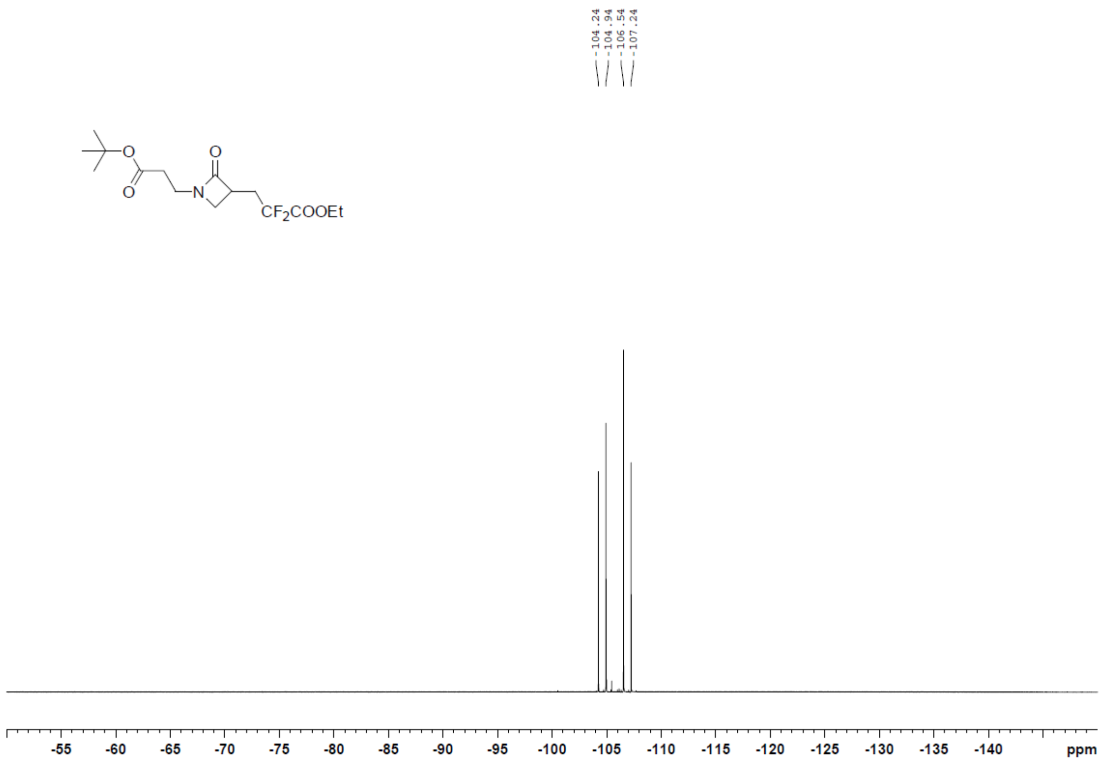

Supplementary Figure 117. ^{19}F NMR spectrum of **3v** in $CDCl_3$ (376 MHz)

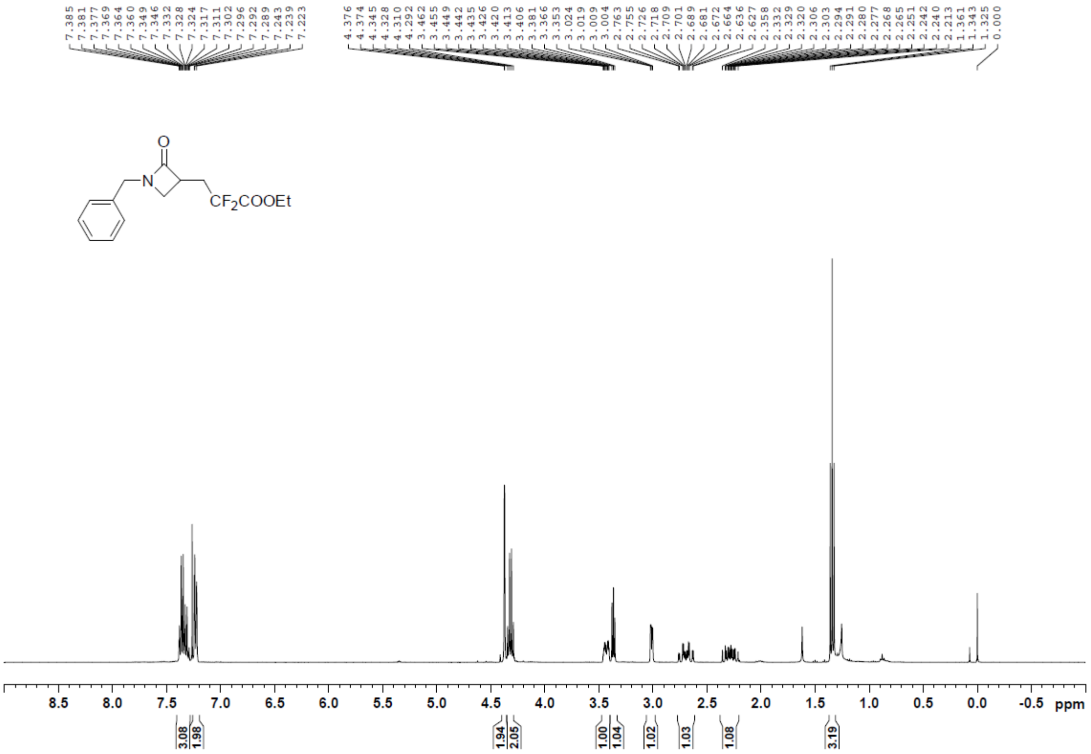

Supplementary Figure 118. ^{1}H NMR spectrum of **3w** in $CDCl_3$ (400 MHz)

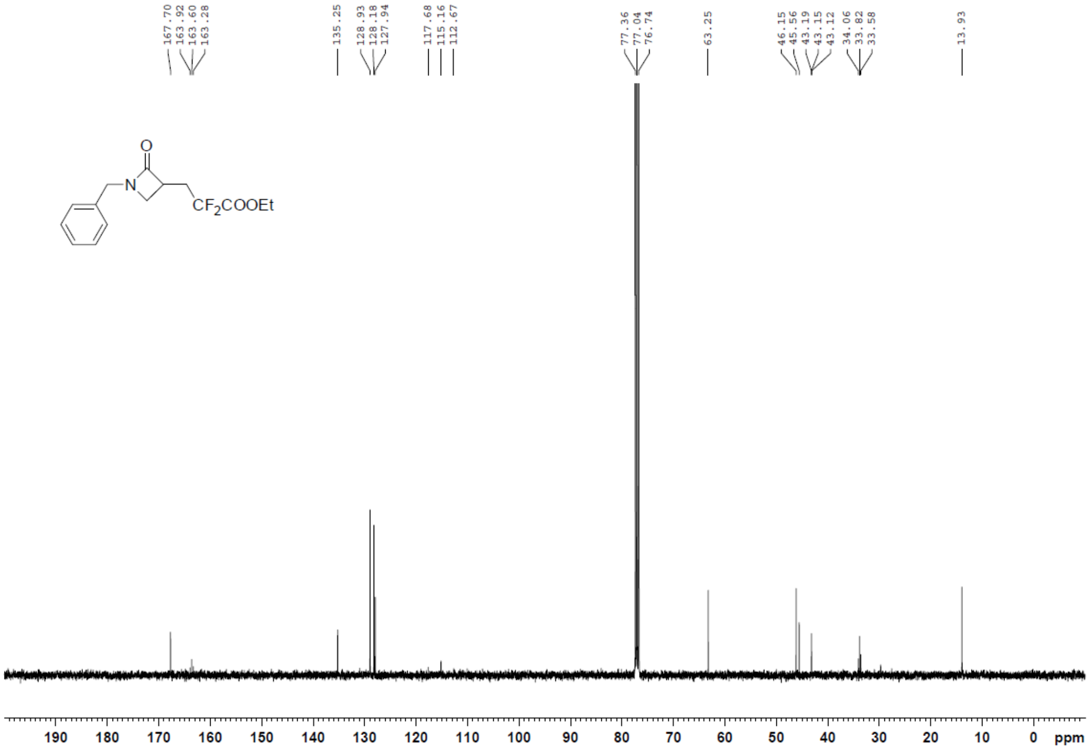

Supplementary Figure 119. ^{13}C NMR spectrum of **3w** in $CDCl_3$ (101 MHz)

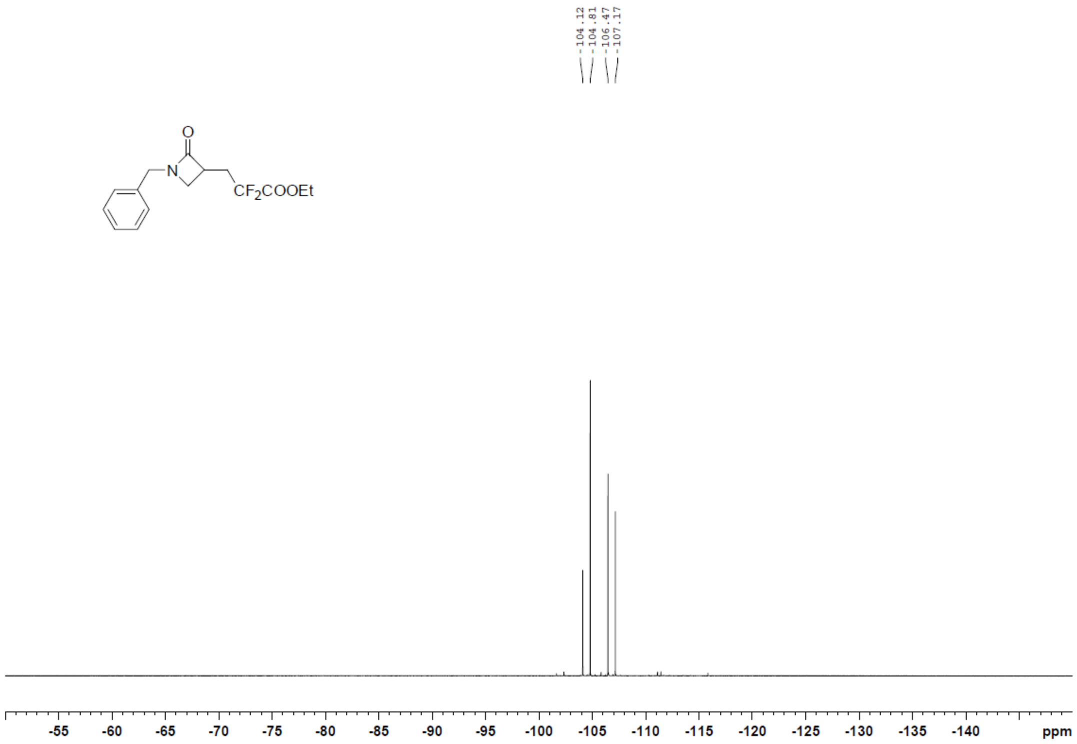

Supplementary Figure 120. ^{19}F NMR spectrum of **3w** in $CDCl_3$ (376 MHz)

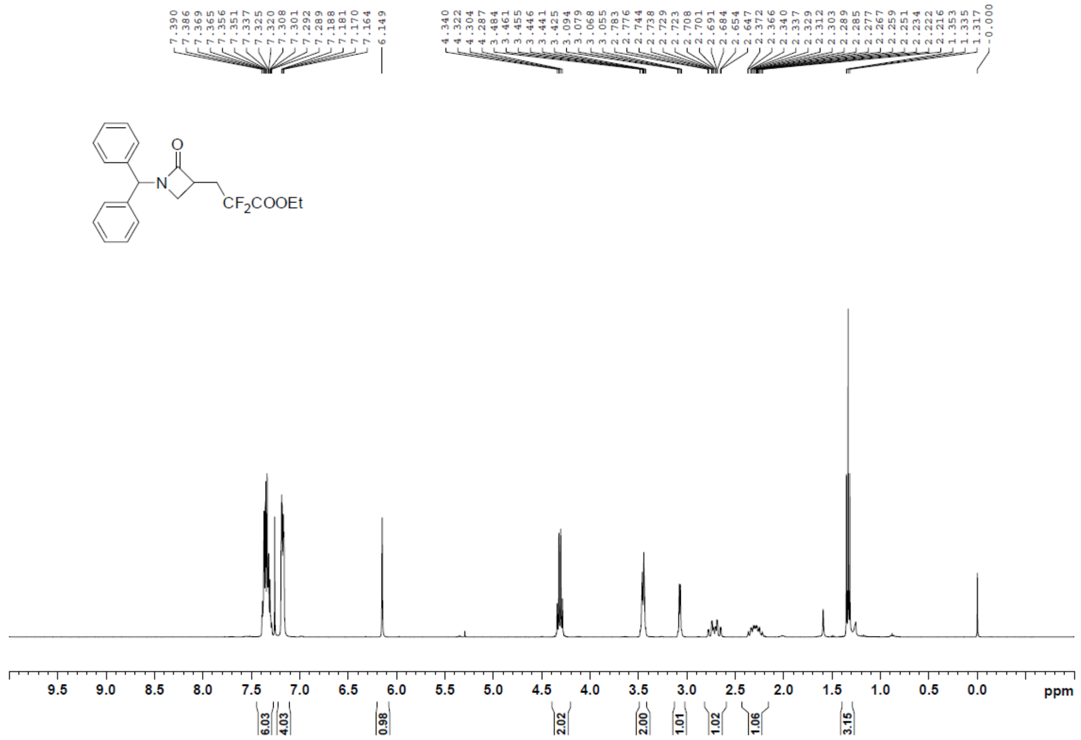

Supplementary Figure 121. ^{1}H NMR spectrum of **3x** in $CDCl_3$ (400 MHz)

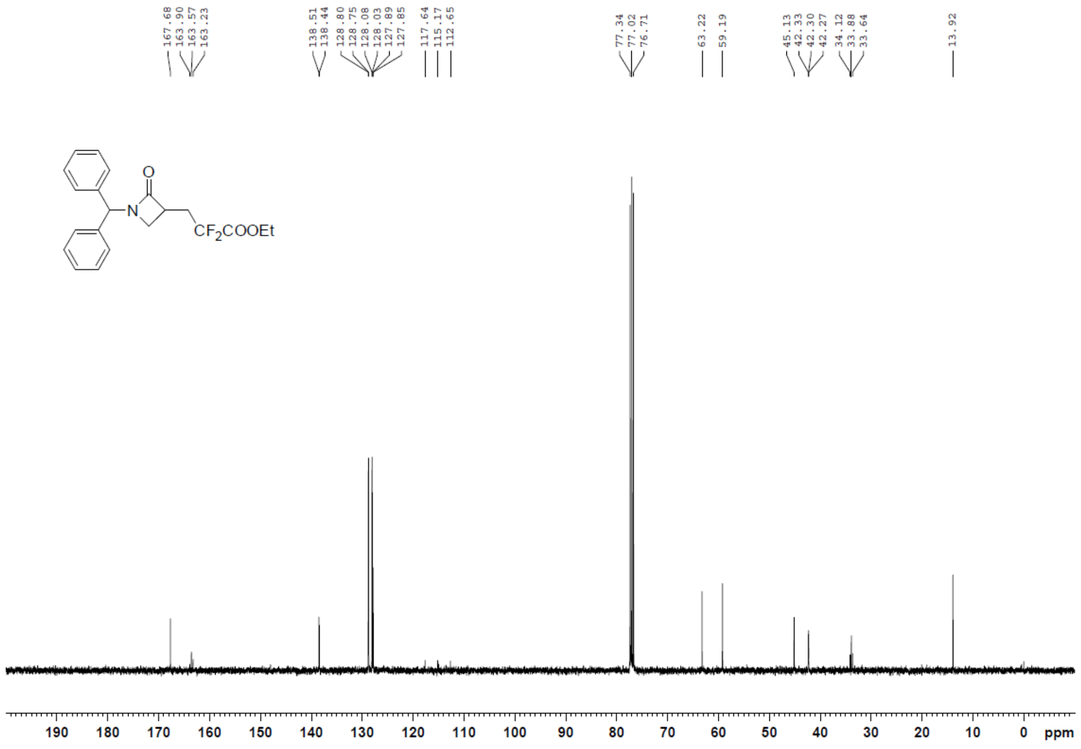

Supplementary Figure 122. ^{13}C NMR spectrum of **3x** in $CDCl_3$ (101 MHz)

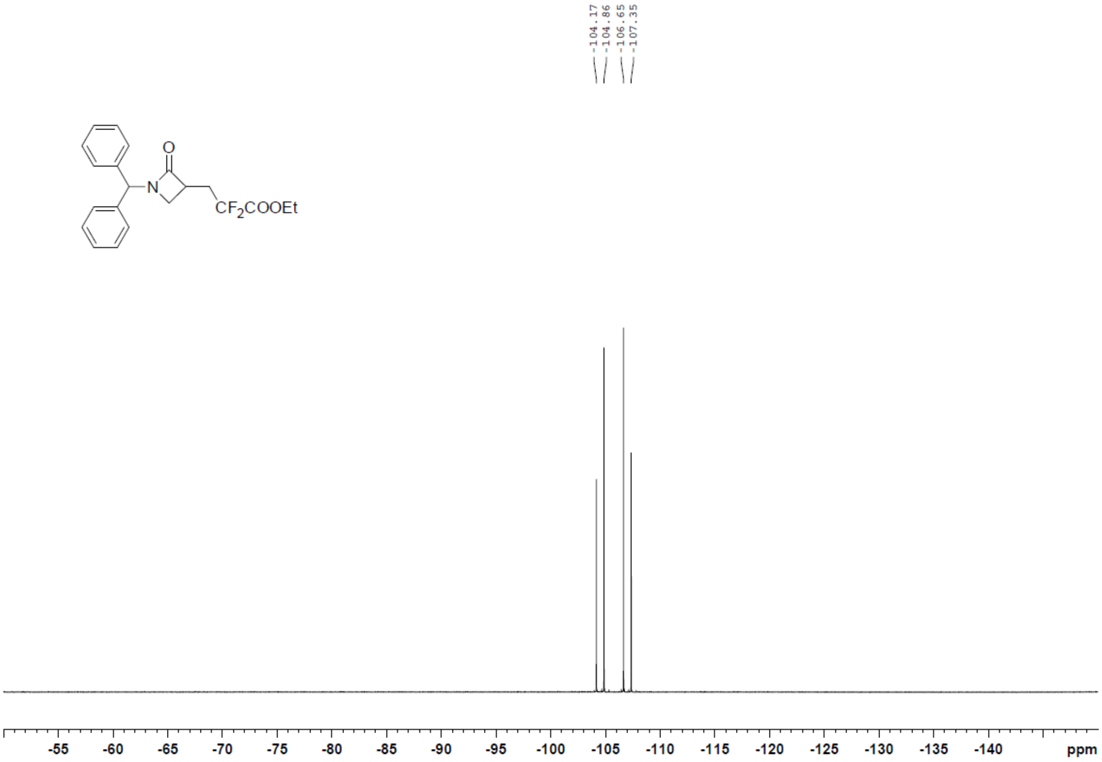

Supplementary Figure 123. ^{19}F NMR spectrum of **3x** in $CDCl_3$ (376 MHz)

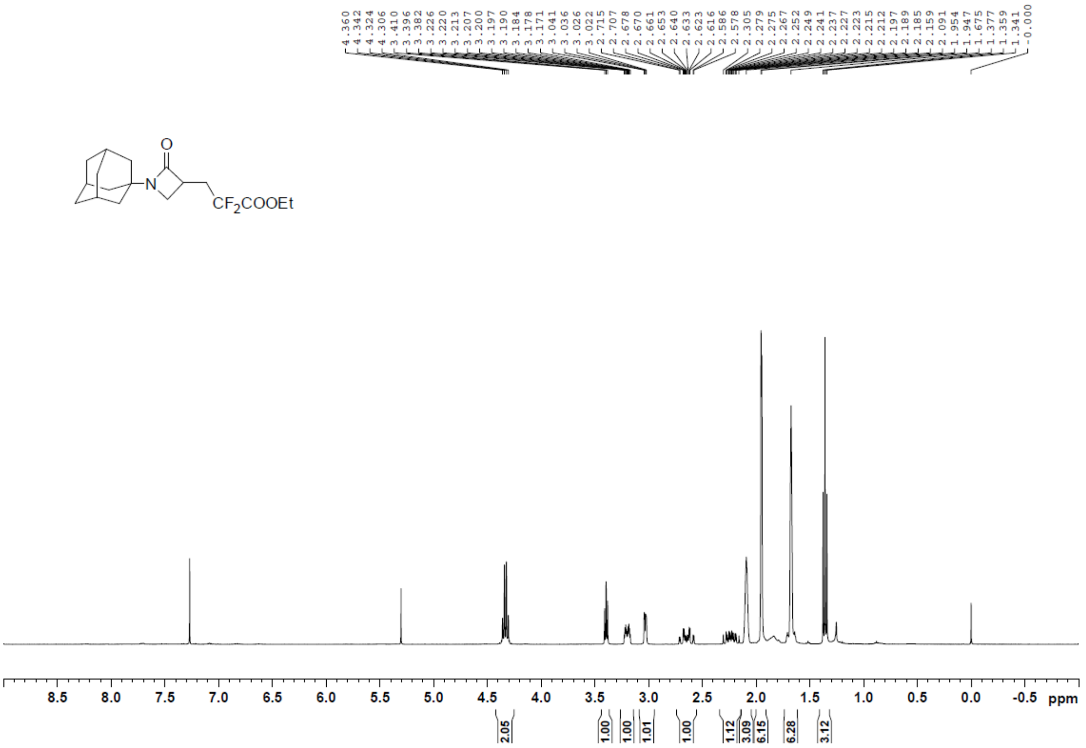

Supplementary Figure 124. ^{1}H NMR spectrum of **3y** in $CDCl_3$ (400 MHz)

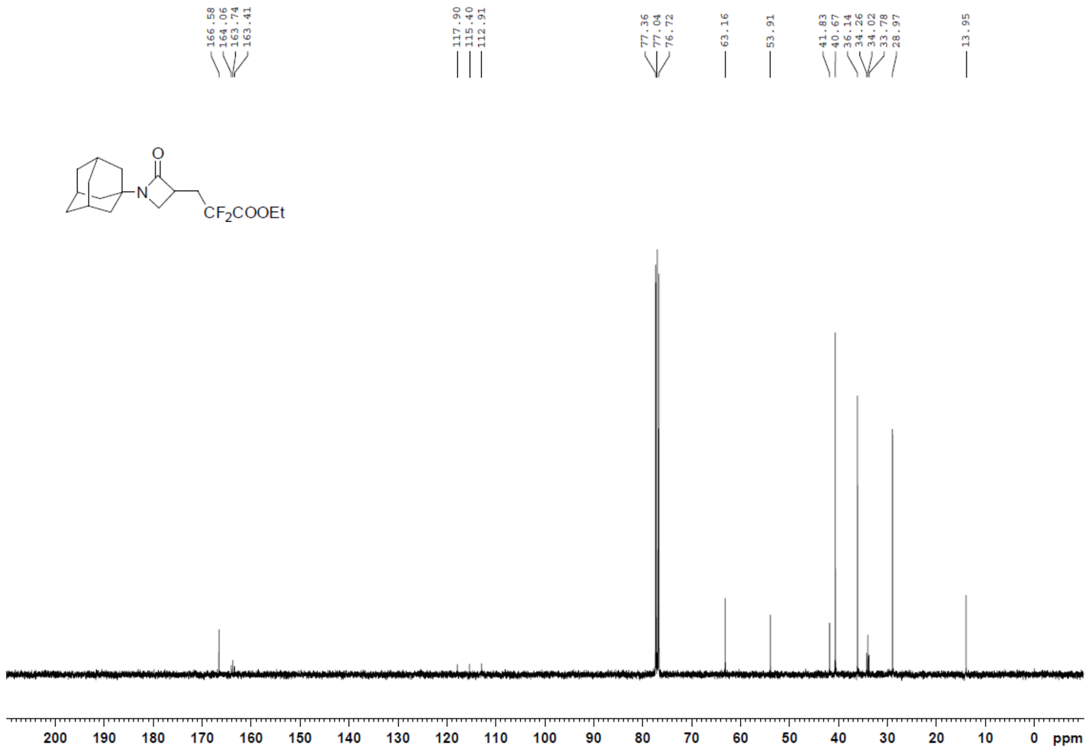

Supplementary Figure 125. ^{13}C NMR spectrum of **3y** in $CDCl_3$ (101 MHz)

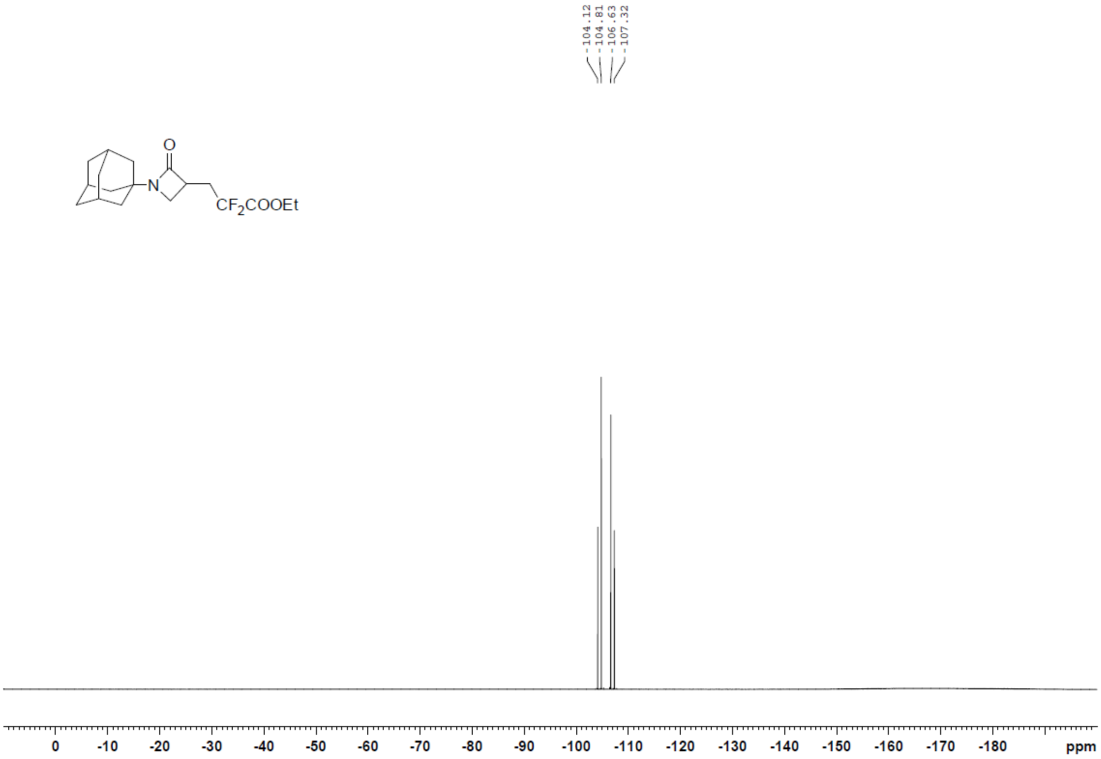

Supplementary Figure 126. ^{19}F NMR spectrum of **3y** in $CDCl_3$ (376 MHz)

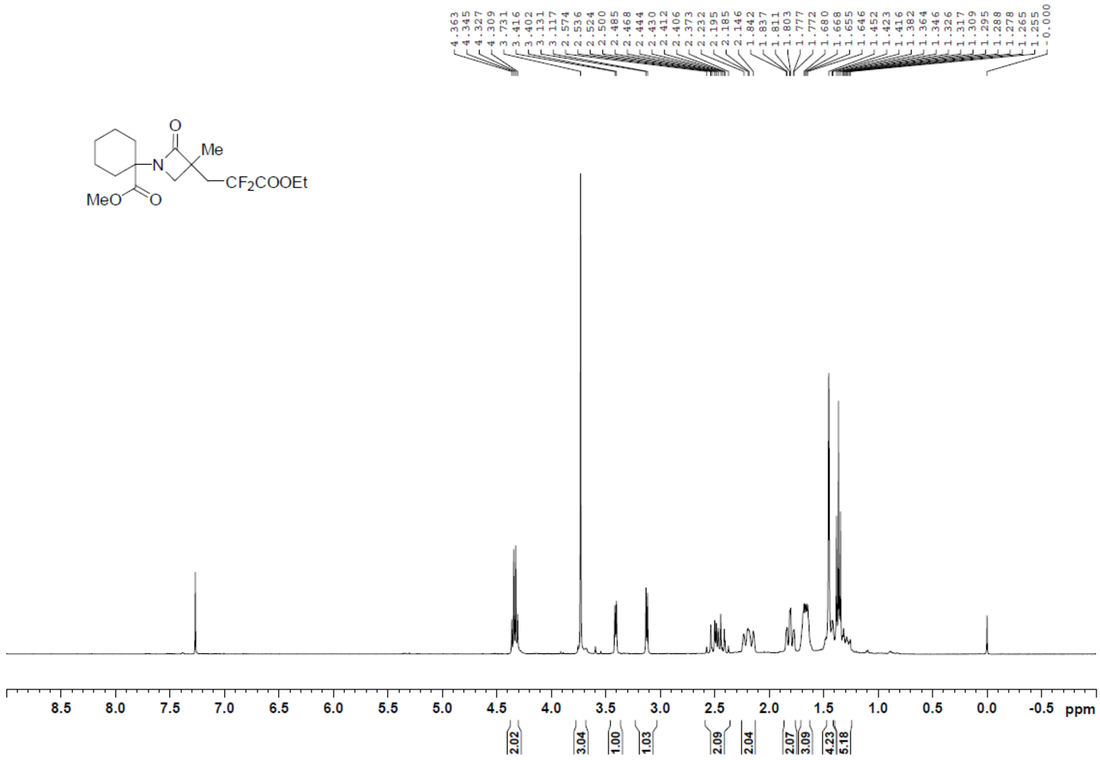

Supplementary Figure 127. ^{1}H NMR spectrum of **3z** in $CDCl_3$ (400 MHz)

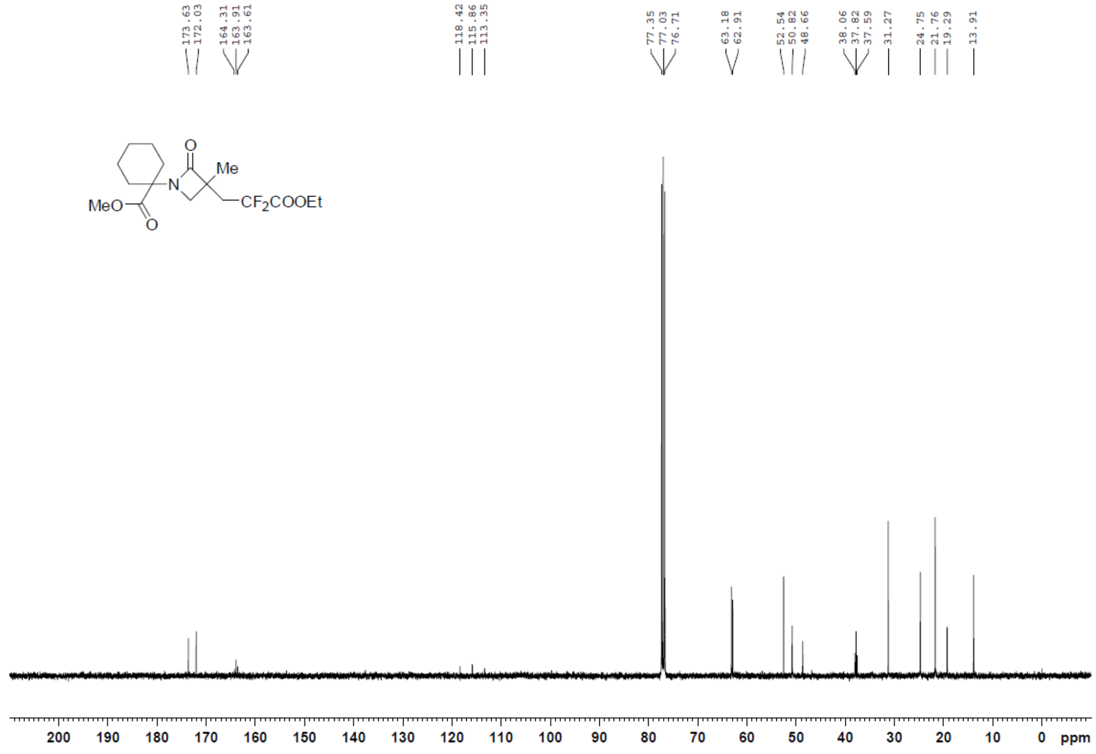

Supplementary Figure 128. ^{13}C NMR spectrum of **3z** in $CDCl_3$ (101 MHz)

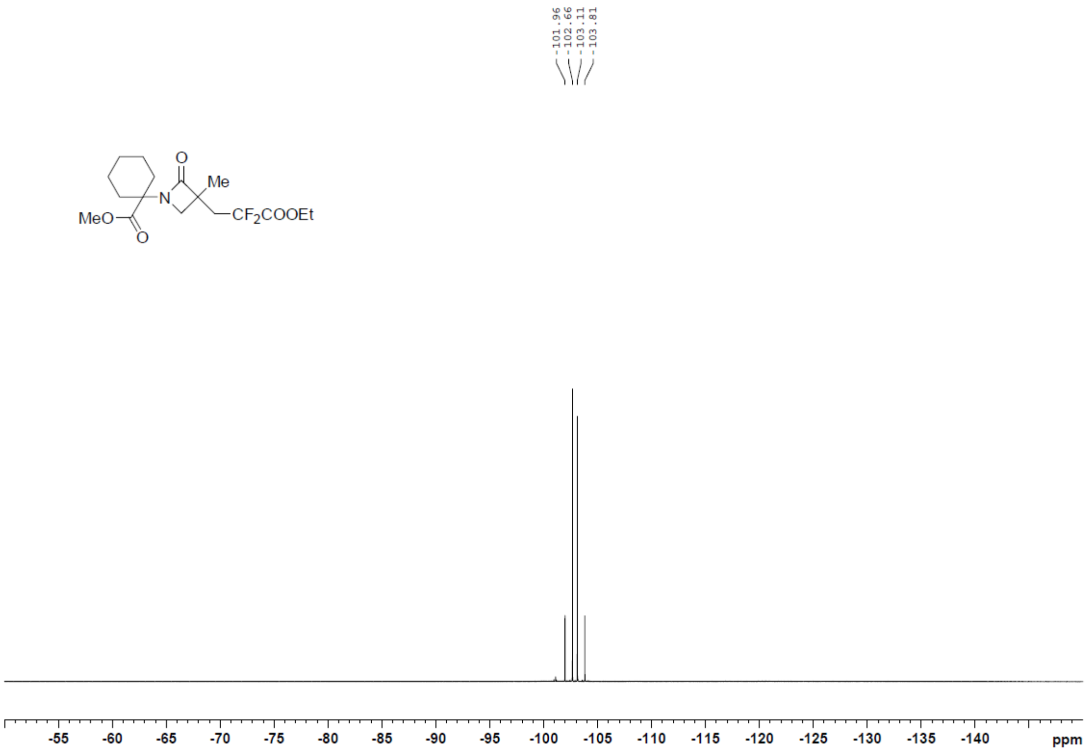

Supplementary Figure 129. ^{19}F NMR spectrum of **3z** in $CDCl_3$ (376 MHz)

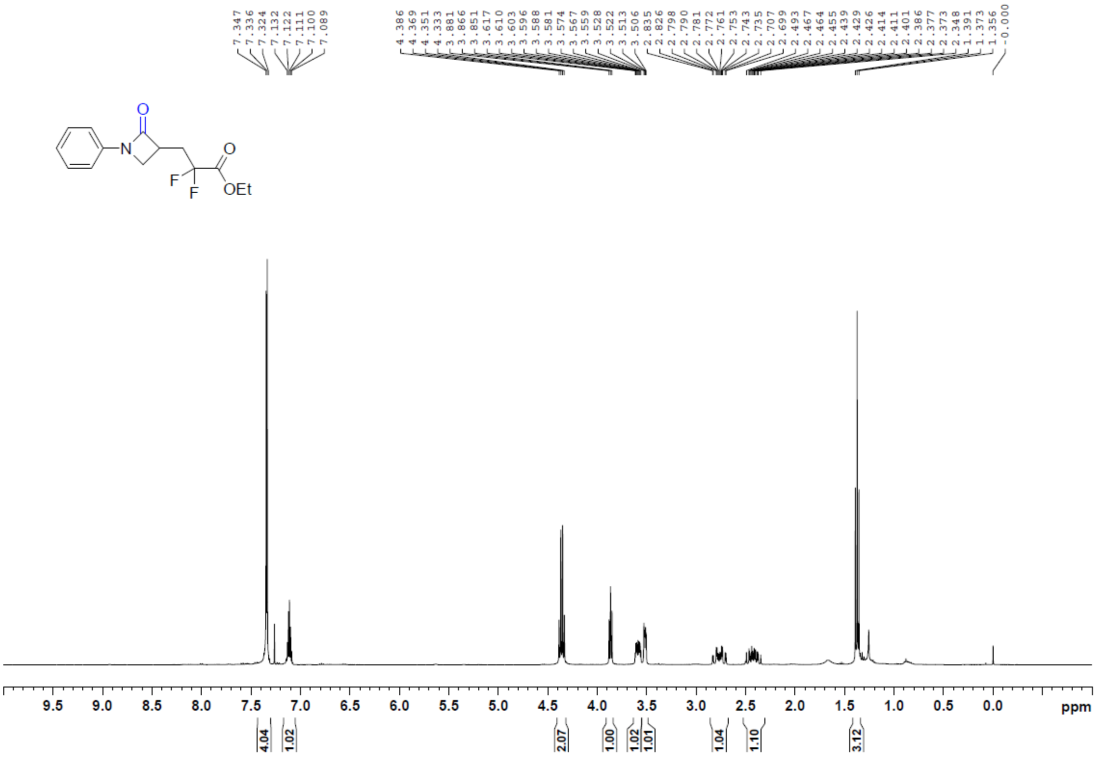

Supplementary Figure 130. ^{1}H NMR spectrum of **4a** in $CDCl_3$ (400 MHz)

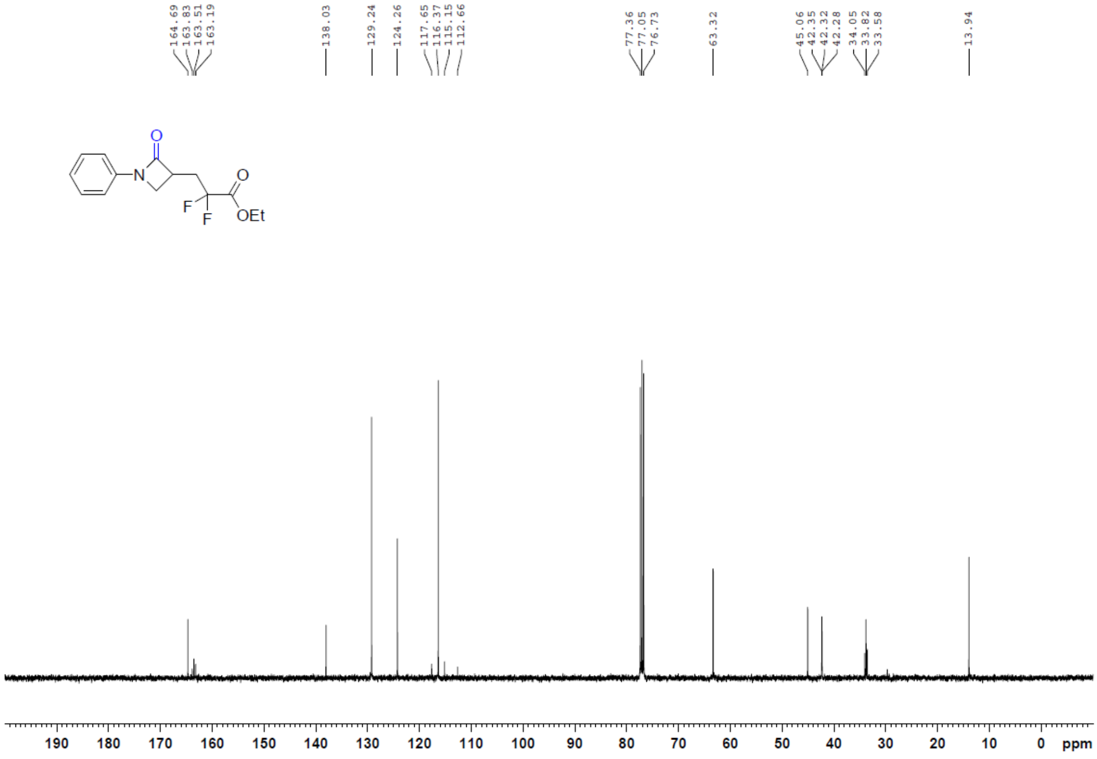

Supplementary Figure 131. ^{13}C NMR spectrum of 4**a** in $CDCl_3$ (101 MHz)

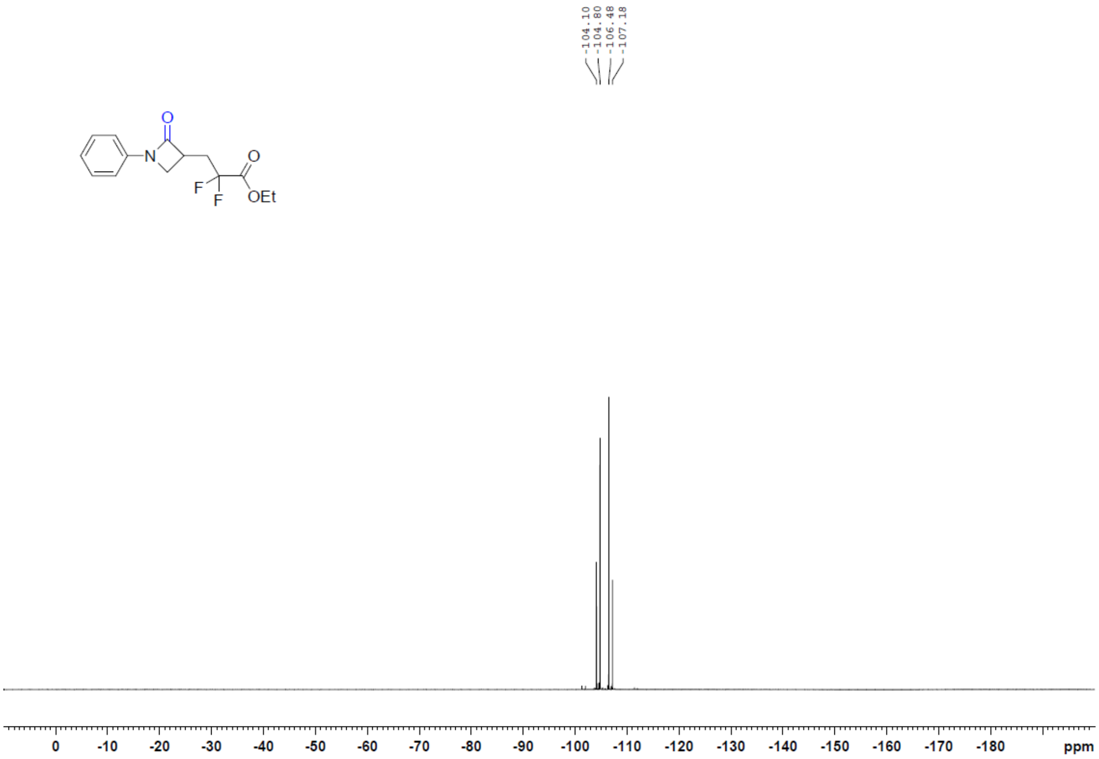

Supplementary Figure 132. ^{19}F NMR spectrum of **4a** in $CDCl_3$ (376 MHz)

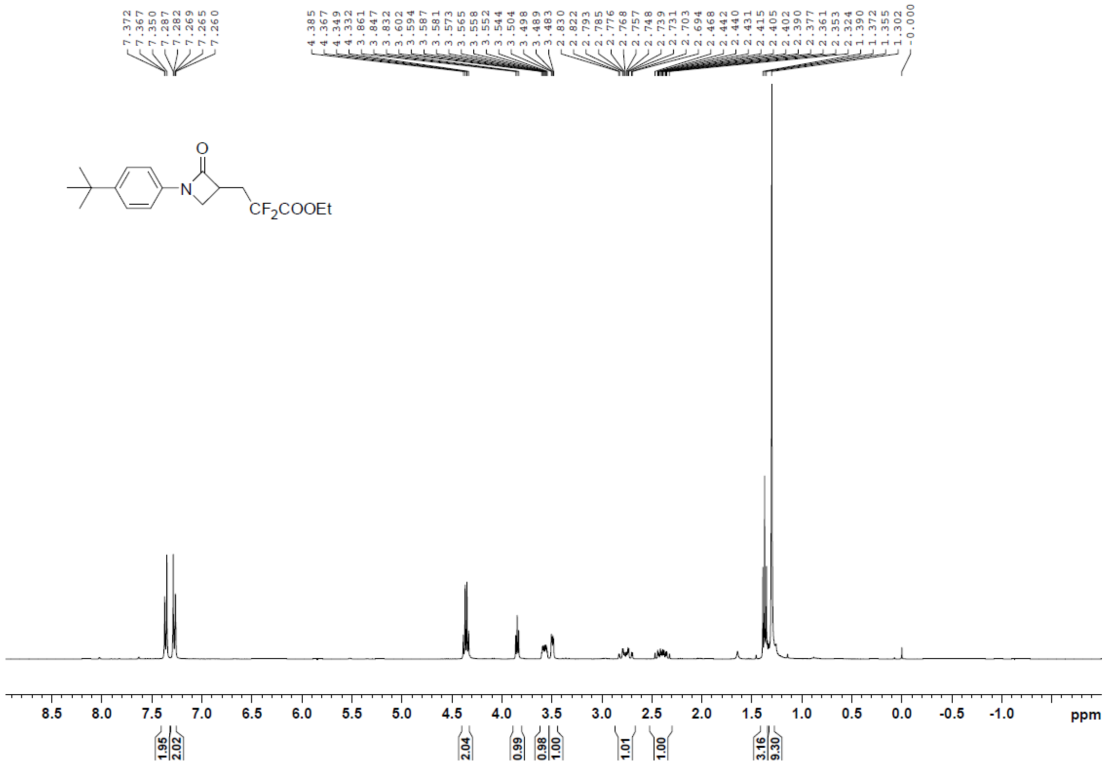

Supplementary Figure 133. ^{1}H NMR spectrum of **4b** in $CDCl_3$ (400 MHz)

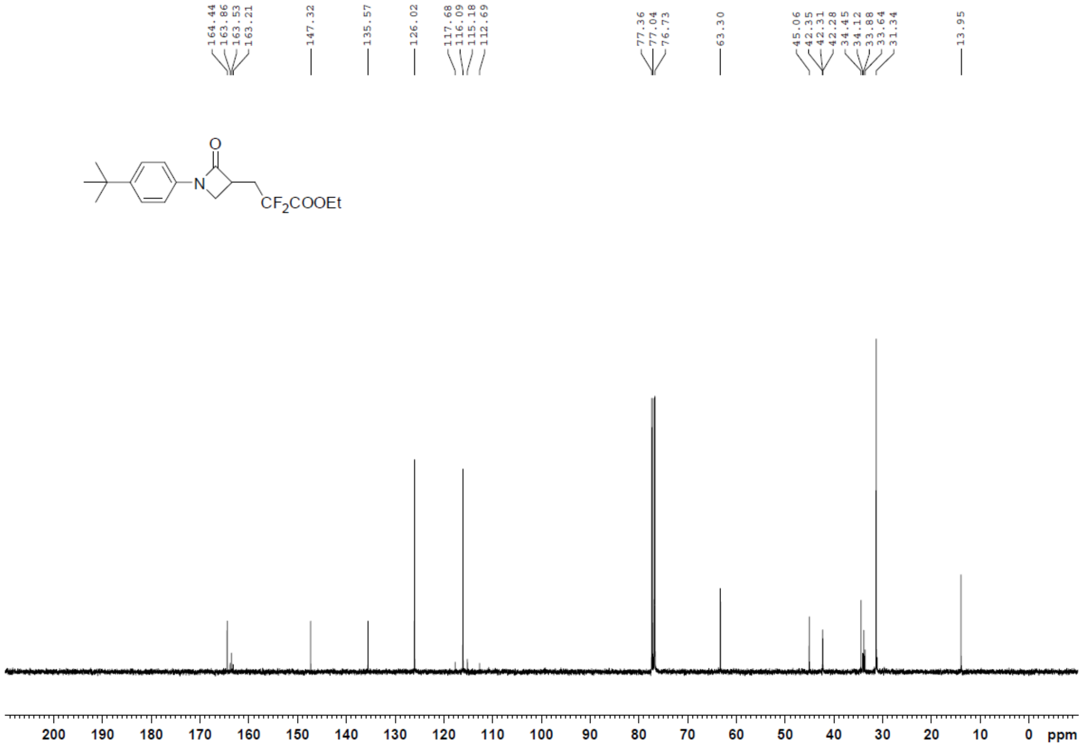

Supplementary Figure 134. ^{13}C NMR spectrum of **4b** in $CDCl_3$ (101 MHz)

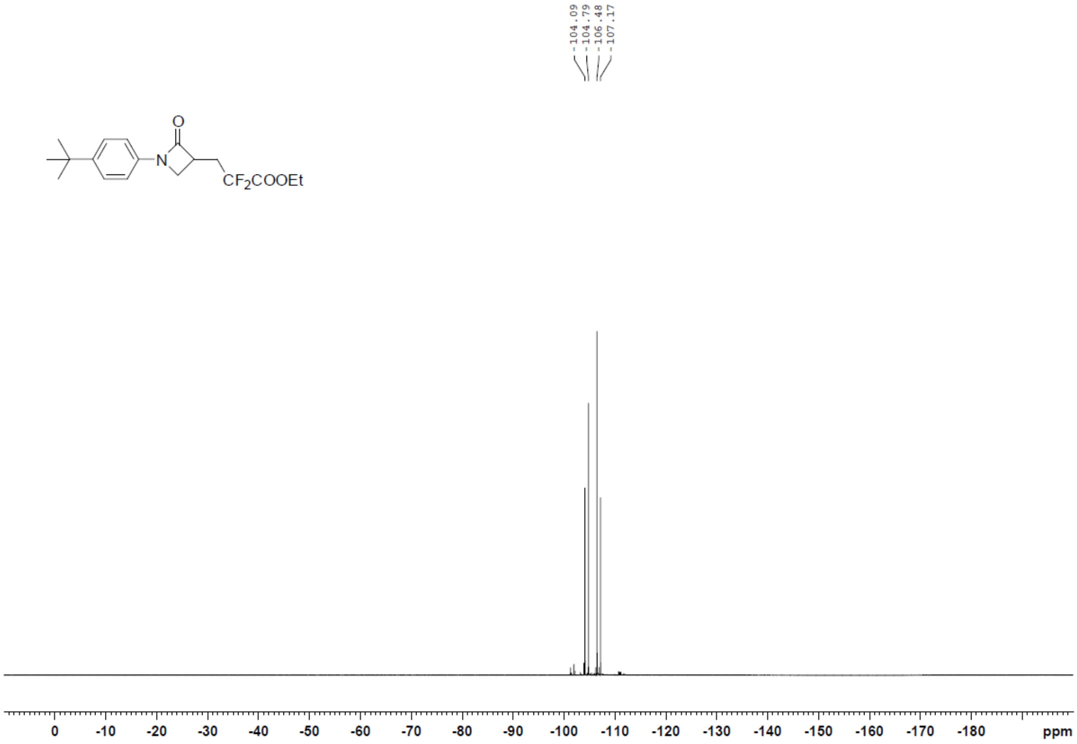

Supplementary Figure 135. ^{19}F NMR spectrum of **4b** in $CDCl_3$ (376 MHz)

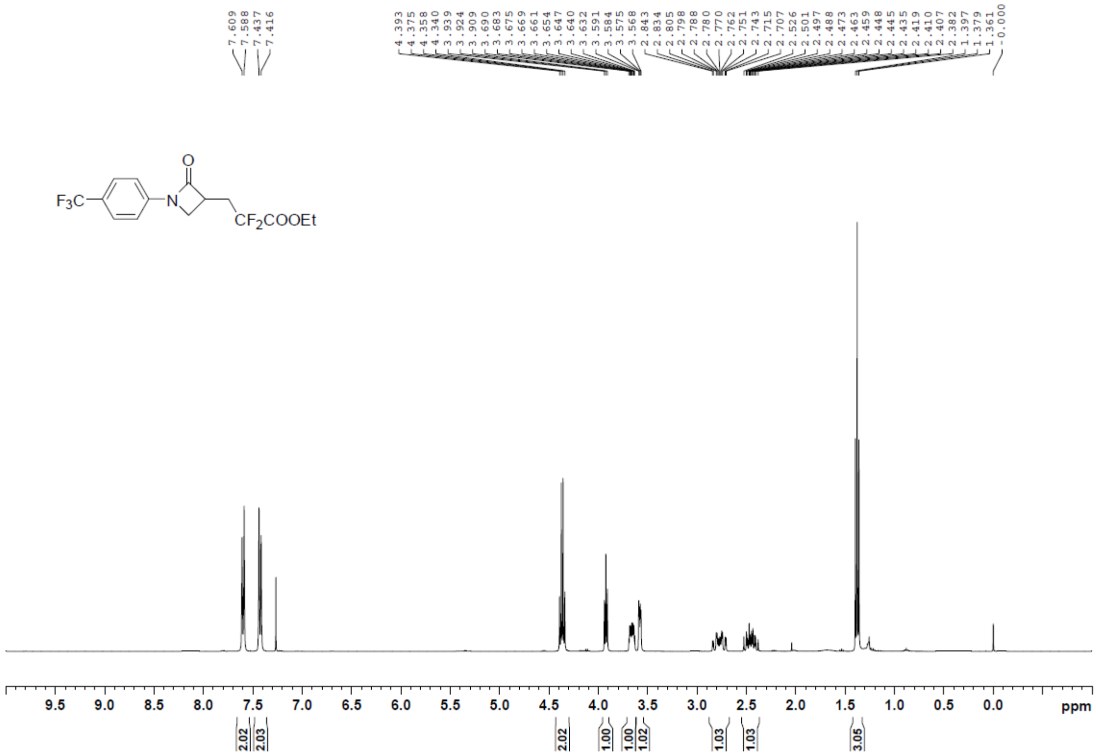

Supplementary Figure 136. ^{1}H NMR spectrum of **4c** in $CDCl_3$ (400 MHz)

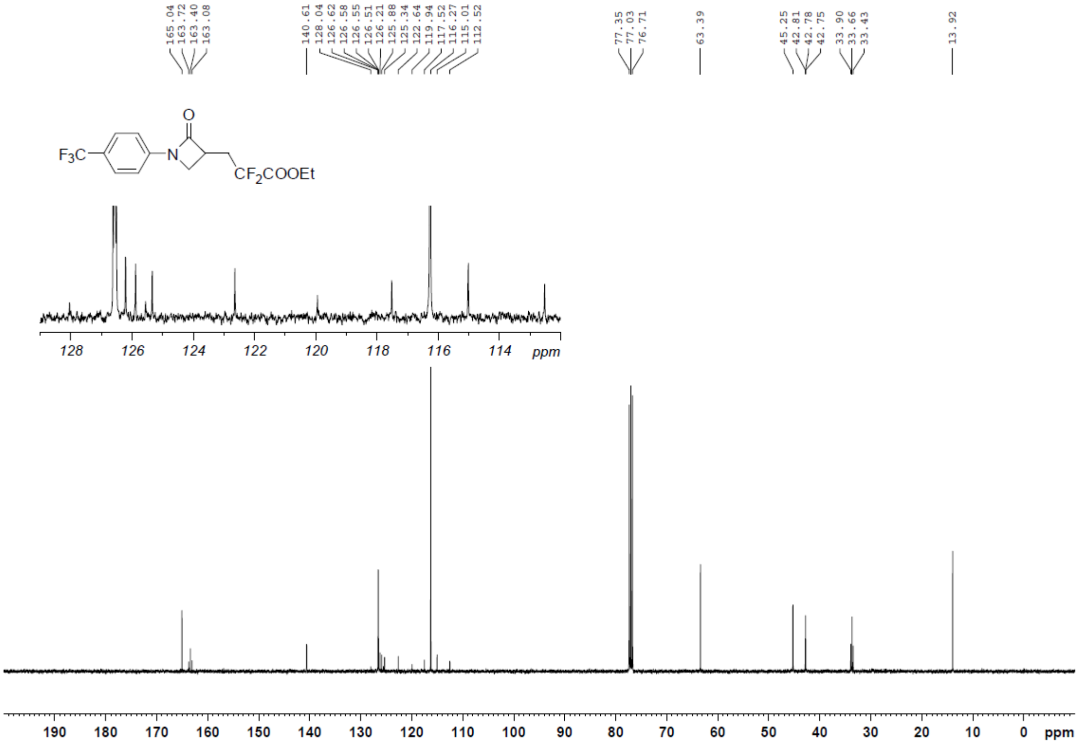

Supplementary Figure 137. ^{13}C NMR spectrum of **4c** in $CDCl_3$ (101 MHz)

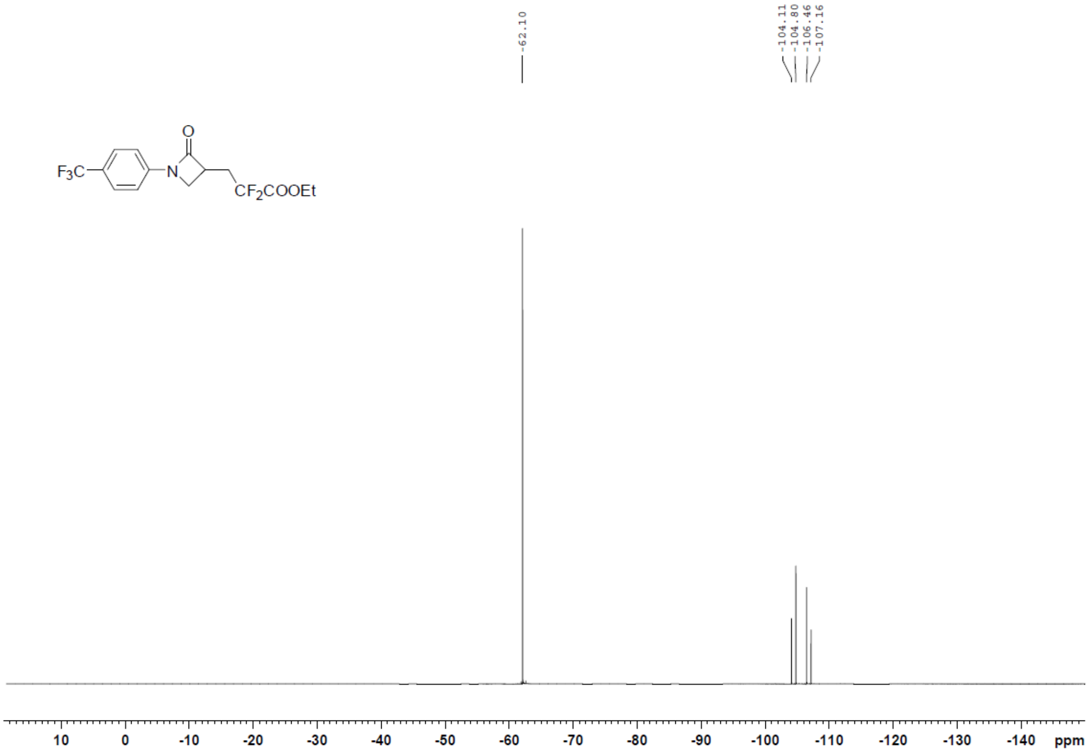

Supplementary Figure 138. ^{19}F NMR spectrum of **4c** in $CDCl_3$ (376 MHz)

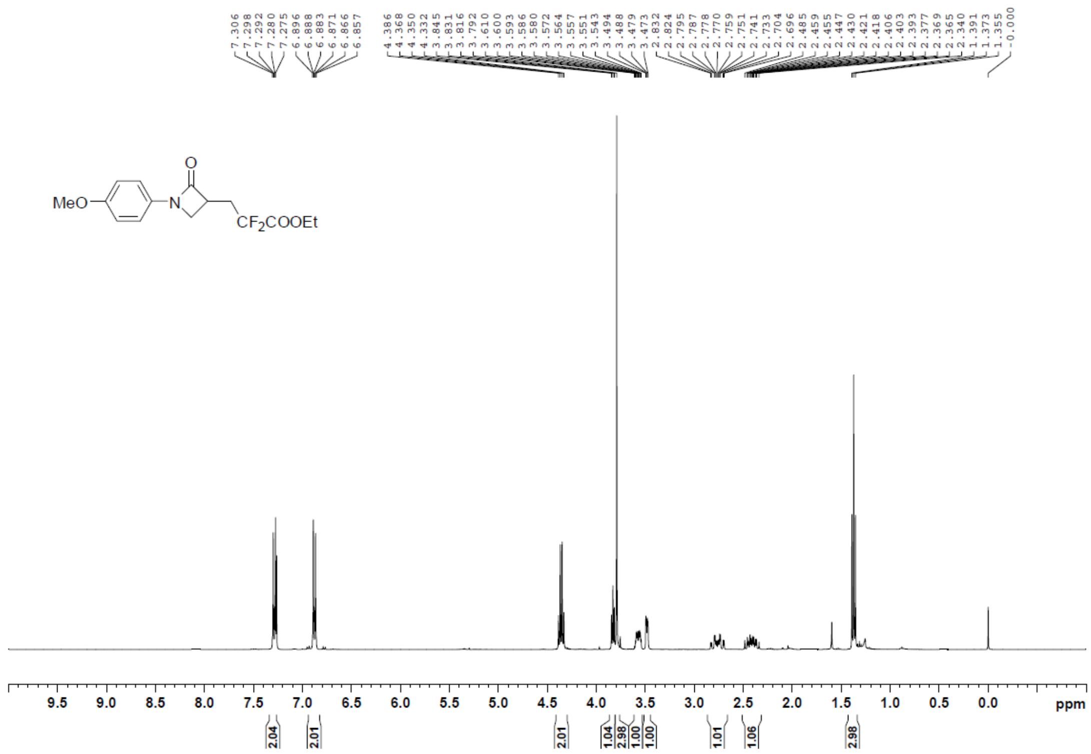

Supplementary Figure 139. ^{1}H NMR spectrum of **4d** in $CDCl_3$ (400 MHz)

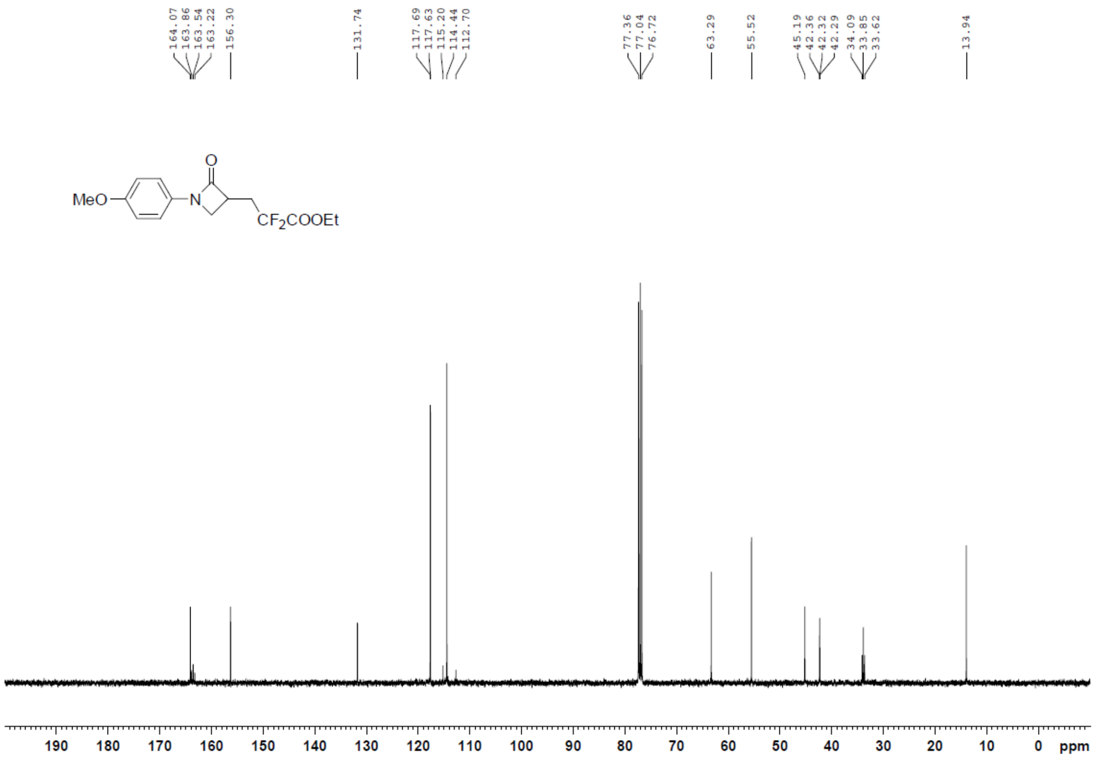

Supplementary Figure 140. ^{13}C NMR spectrum of **4d** in $CDCl_3$ (101 MHz)

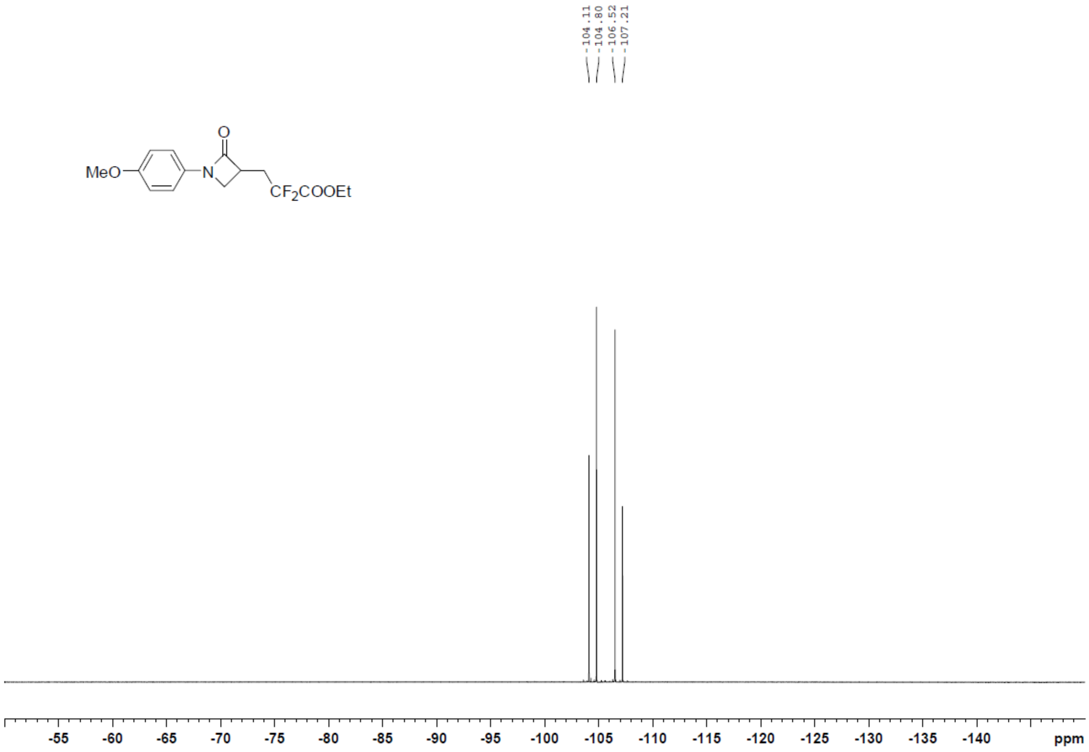

Supplementary Figure 141. ^{19}F NMR spectrum of **4d** in $CDCl_3$ (376 MHz)

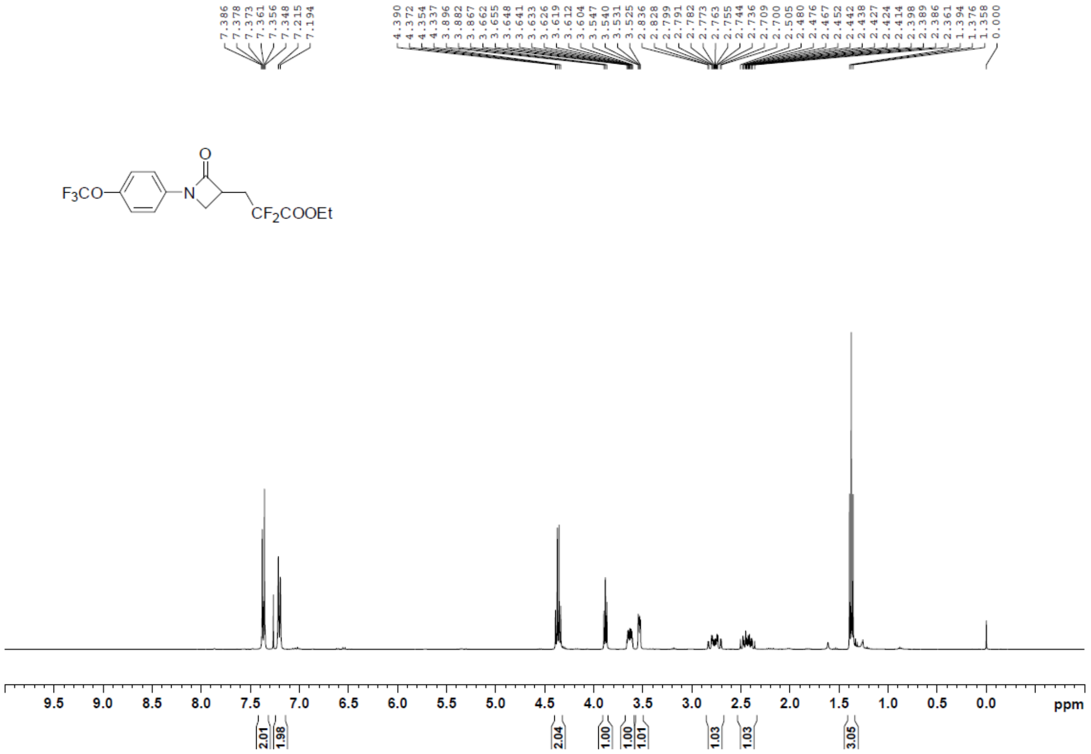

Supplementary Figure 142. ^{1}H NMR spectrum of **4e** in $CDCl_3$ (400 MHz)

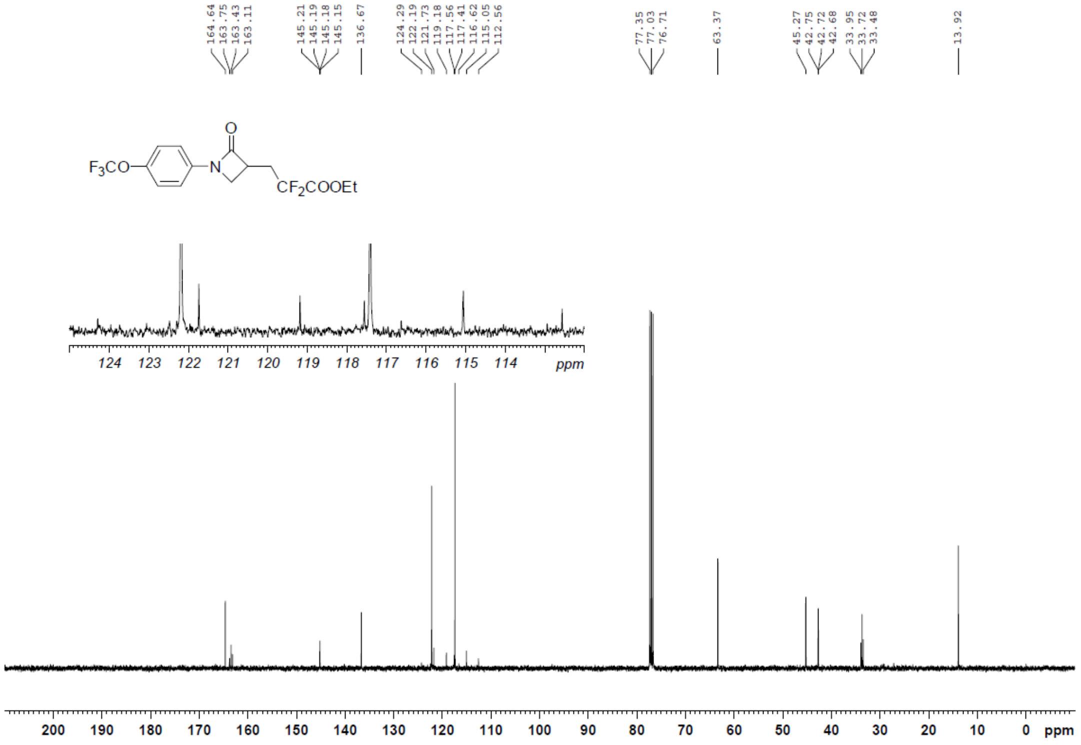

Supplementary Figure 143. ^{13}C NMR spectrum of **4e** in $CDCl_3$ (101 MHz)

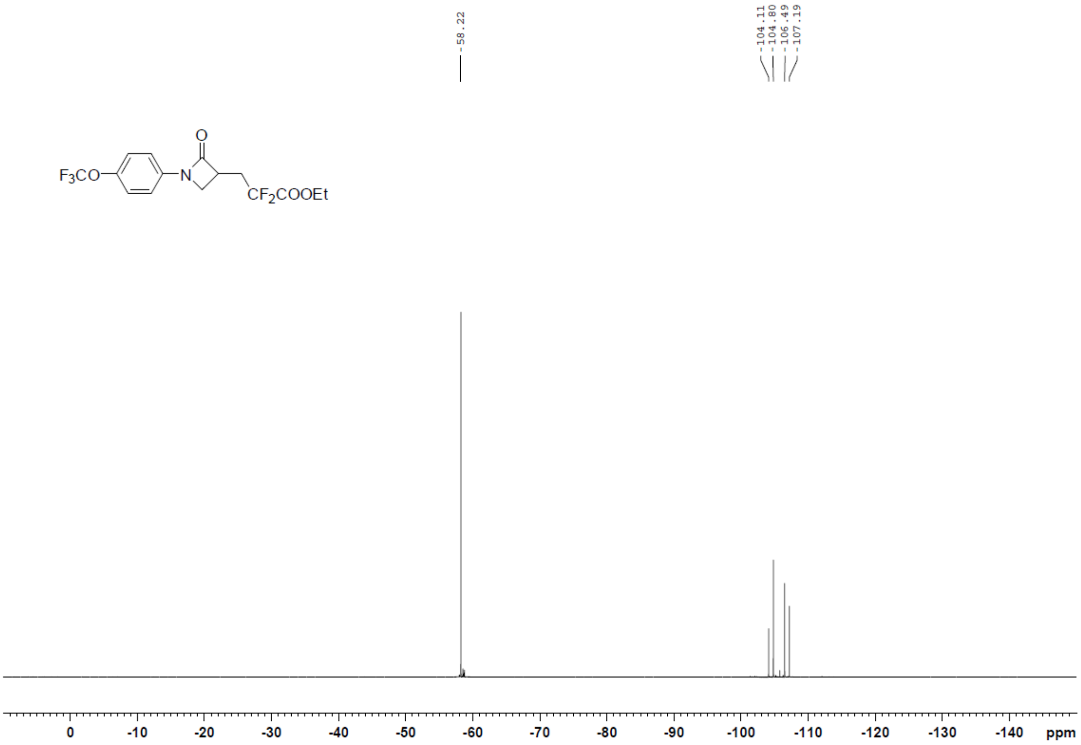

Supplementary Figure 144. ^{19}F NMR spectrum of **4e** in $CDCl_3$ (376 MHz)

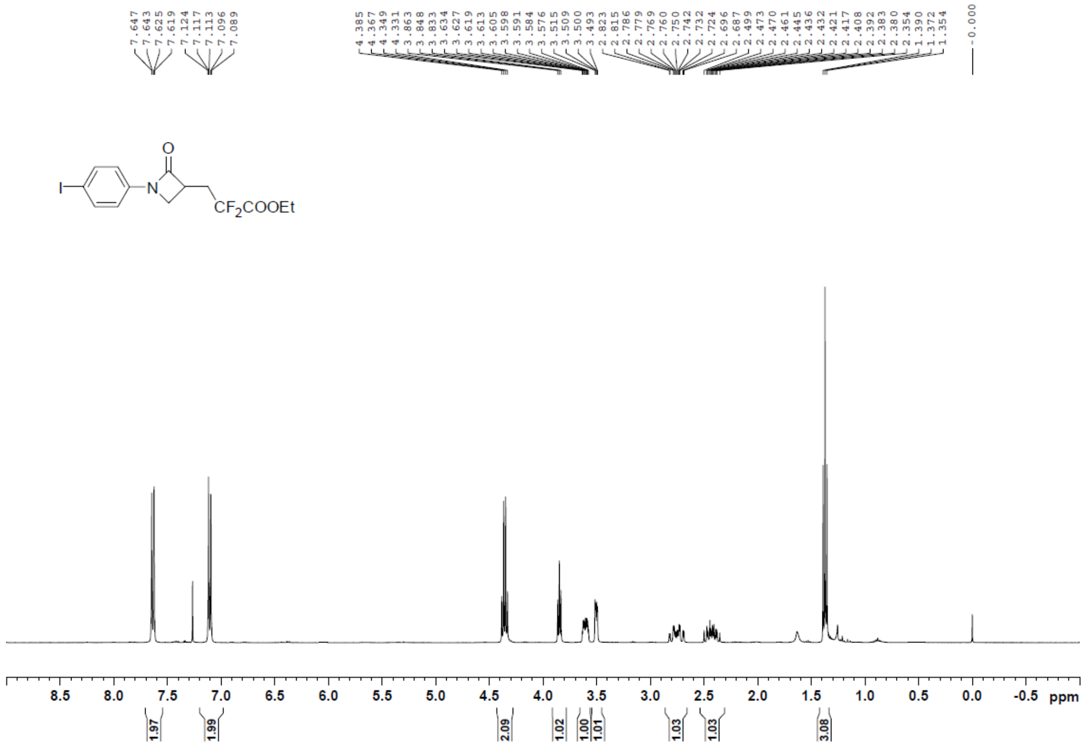

Supplementary Figure 145. ^{1}H NMR spectrum of **4f** in $CDCl_3$ (400 MHz)

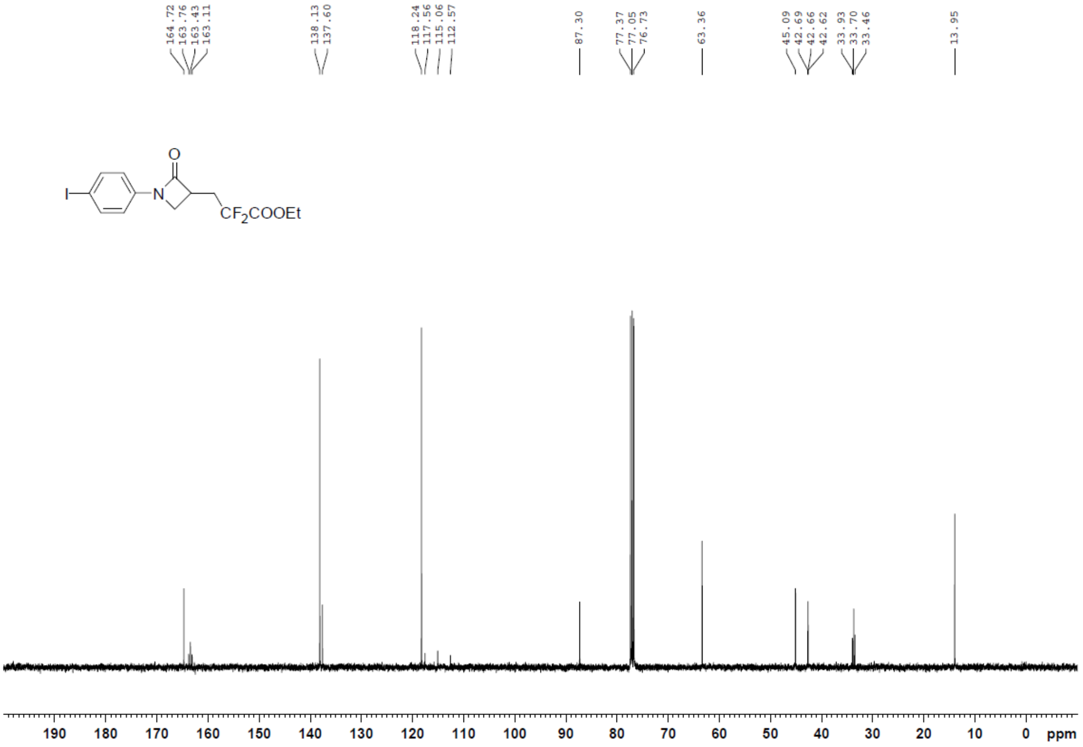

Supplementary Figure 146. ^{13}C NMR spectrum of **4f** in $CDCl_3$ (101 MHz)

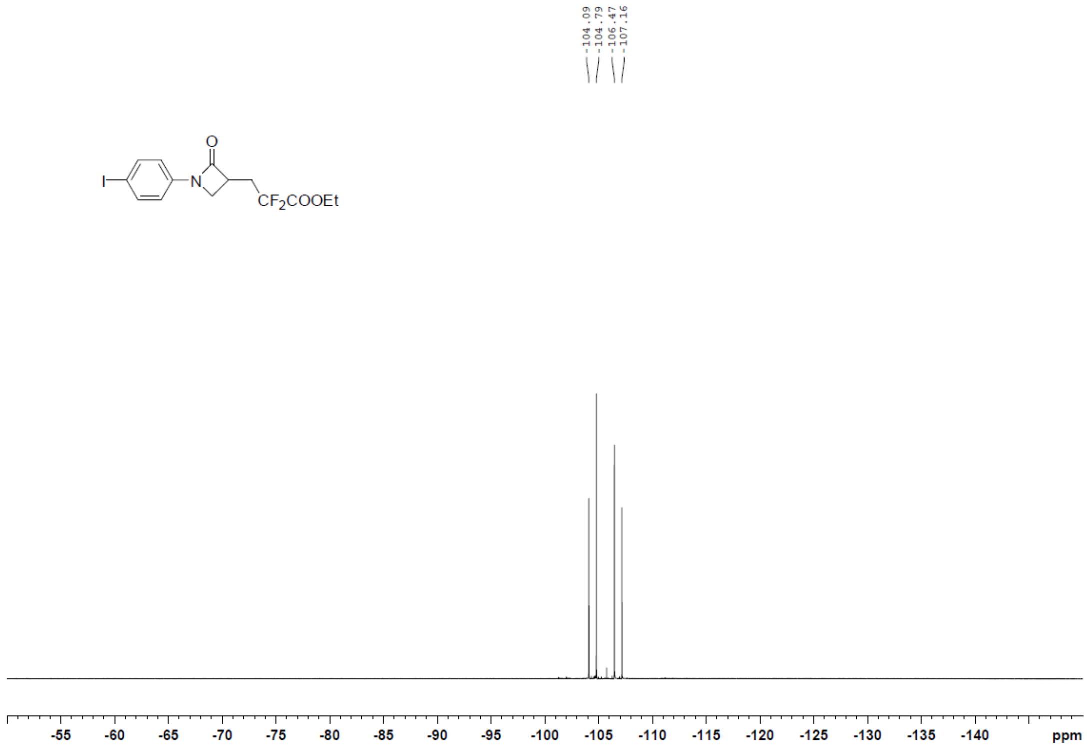

Supplementary Figure 147. ^{19}F NMR spectrum of **4f** in $CDCl_3$ (376 MHz)

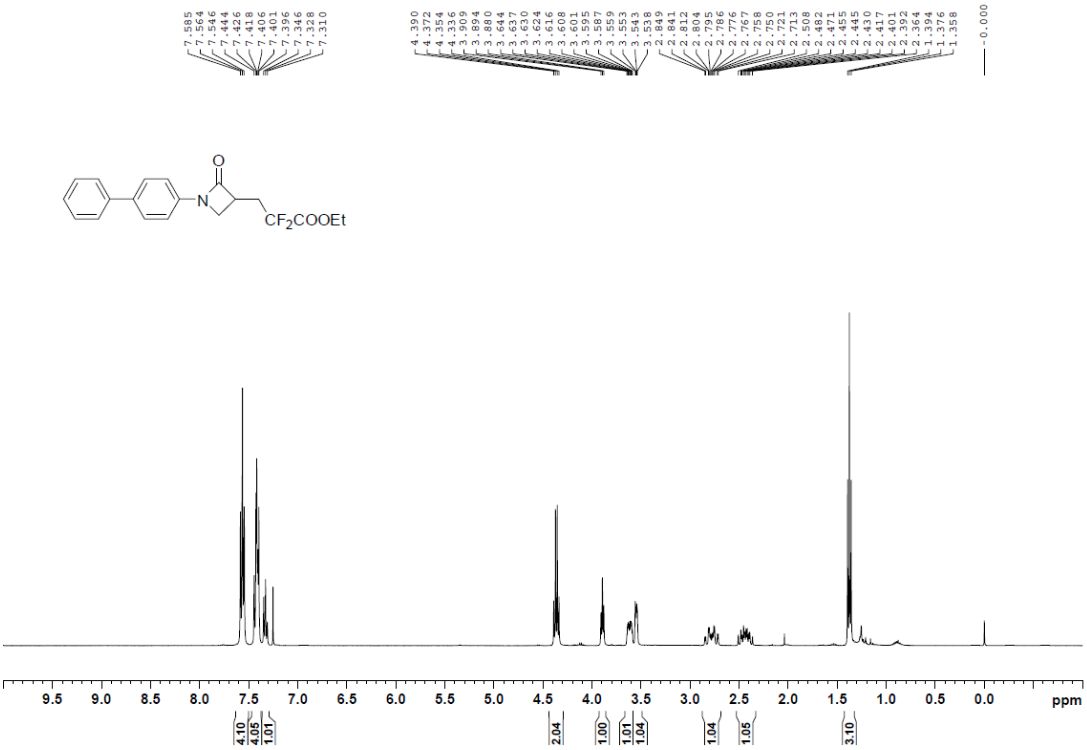

Supplementary Figure 148. ^{1}H NMR spectrum of **4g** in $CDCl_3$ (400 MHz)

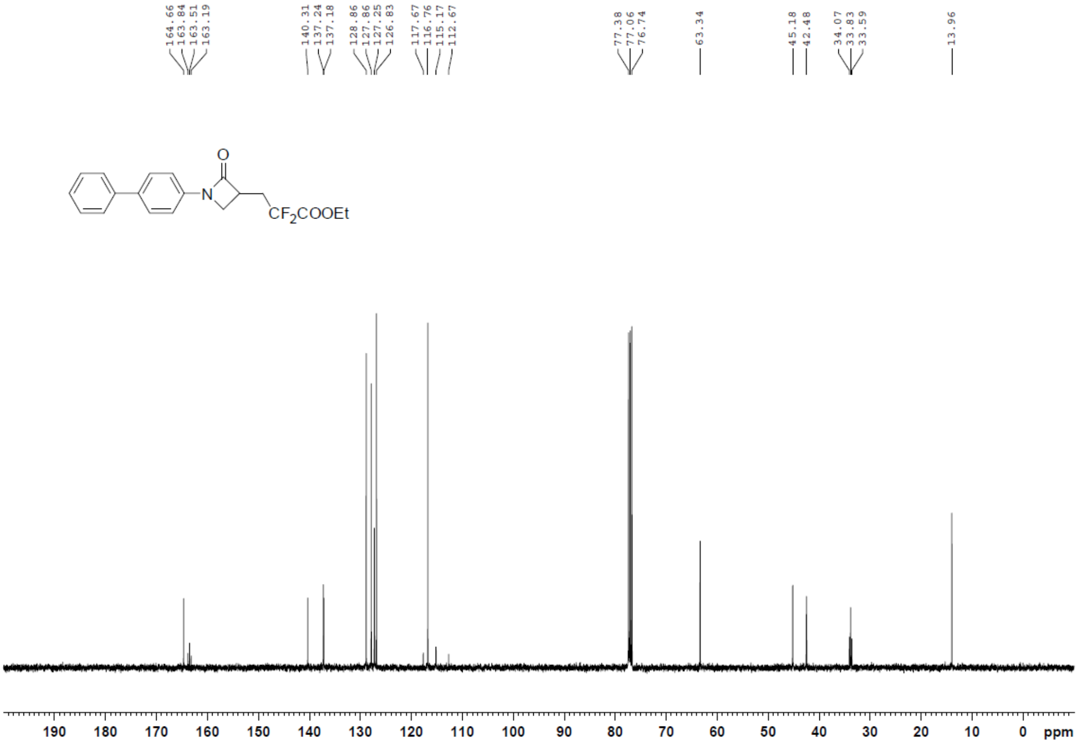

Supplementary Figure 149. ^{13}C NMR spectrum of **4g** in $CDCl_3$ (101 MHz)

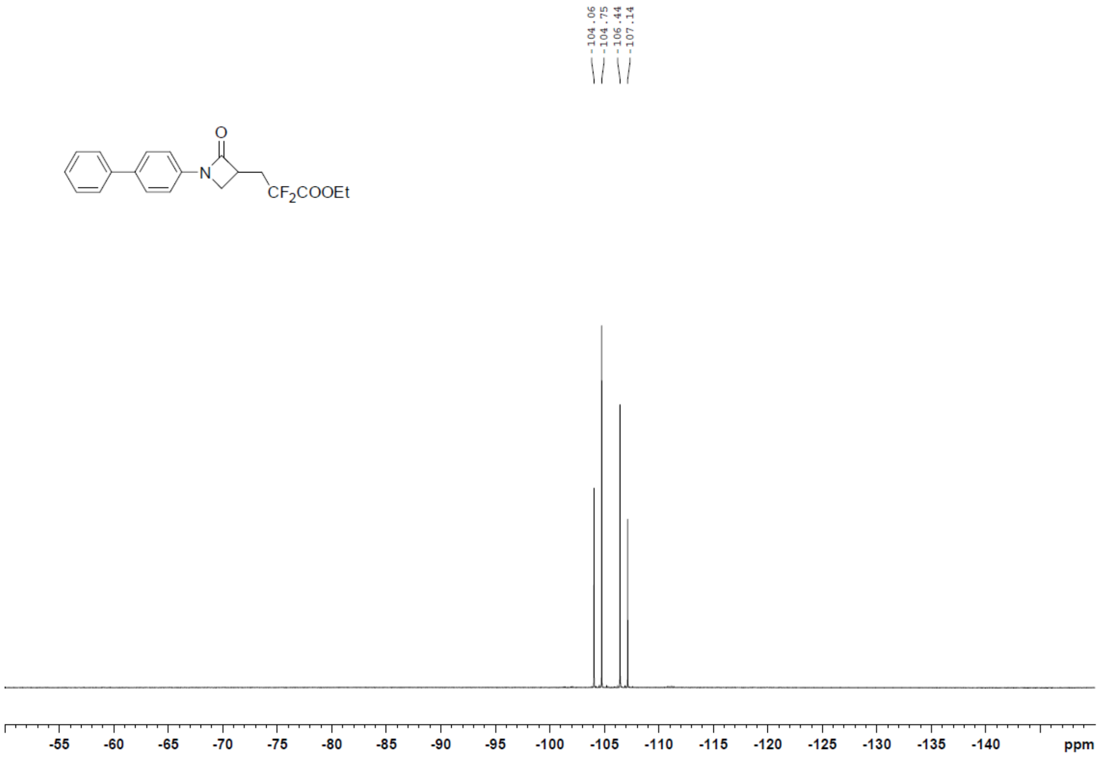

Supplementary Figure 150. ^{19}F NMR spectrum of **4g** in $CDCl_3$ (376 MHz)

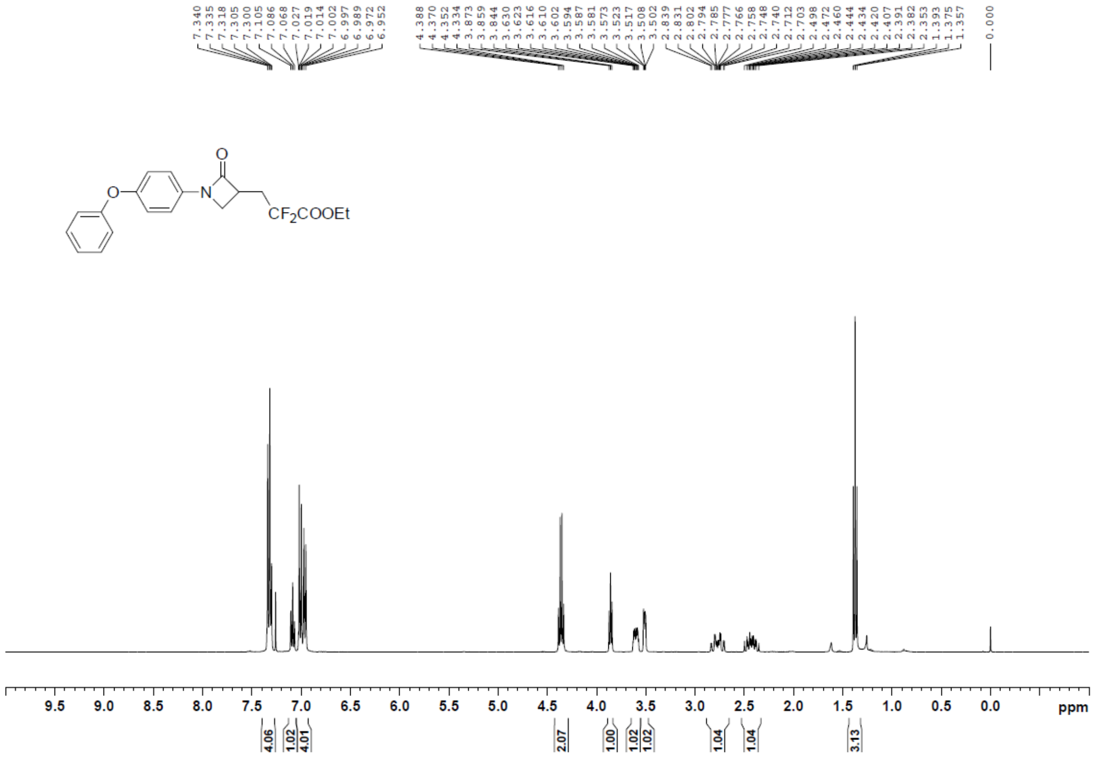

Supplementary Figure 151. ^{1}H NMR spectrum of **4h** in $CDCl_3$ (400 MHz)

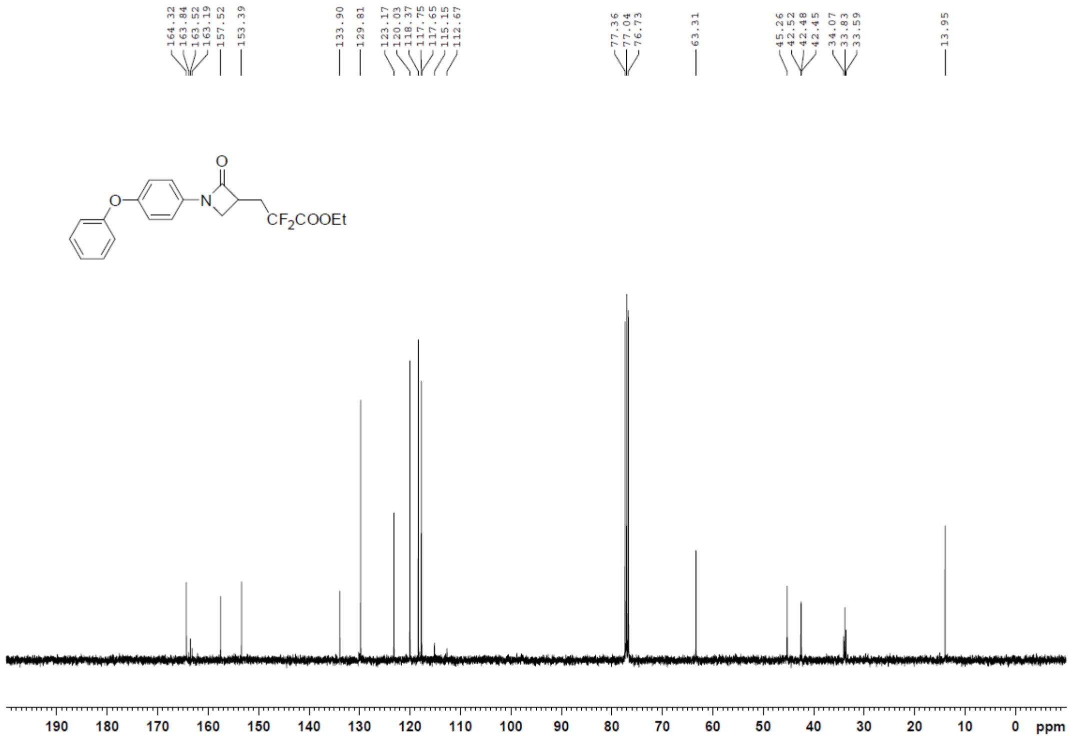

Supplementary Figure 152. ^{13}C NMR spectrum of **4h** in $CDCl_3$ (101 MHz)

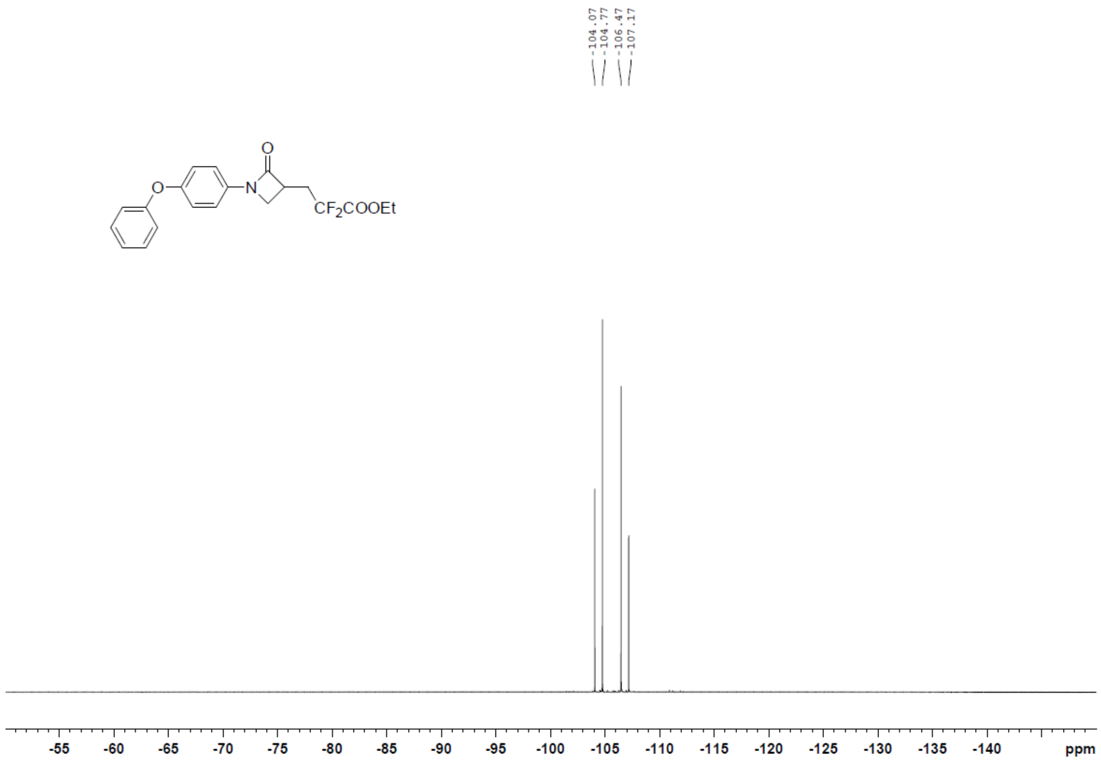

Supplementary Figure 153. ^{19}F NMR spectrum of **4h** in $CDCl_3$ (376 MHz)

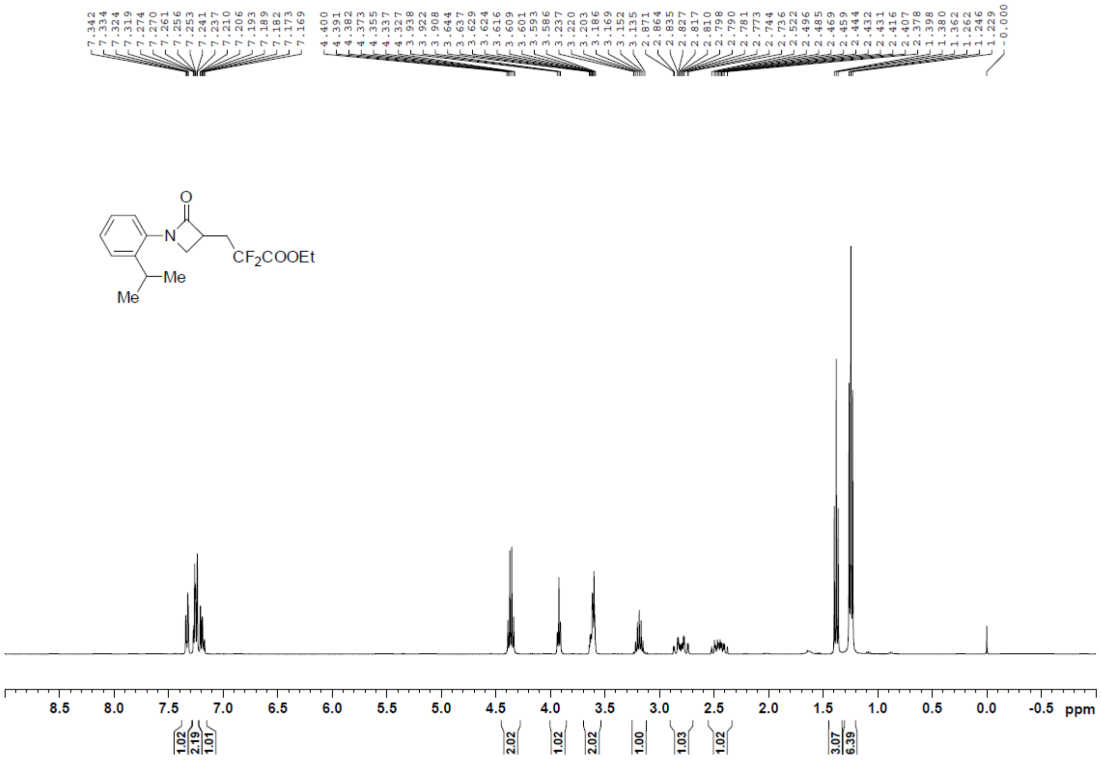

Supplementary Figure 154. ^{1}H NMR spectrum of **4i** in $CDCl_3$ (400 MHz)

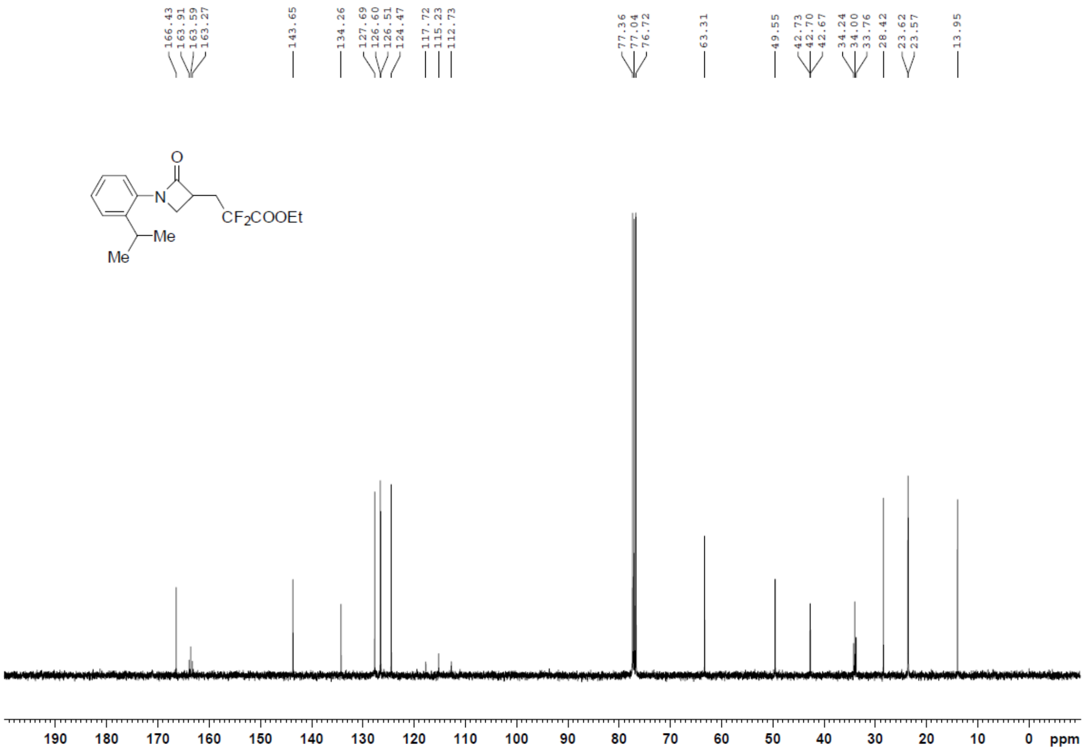

Supplementary Figure 155. ^{13}C NMR spectrum of **4i** in $CDCl_3$ (101 MHz)

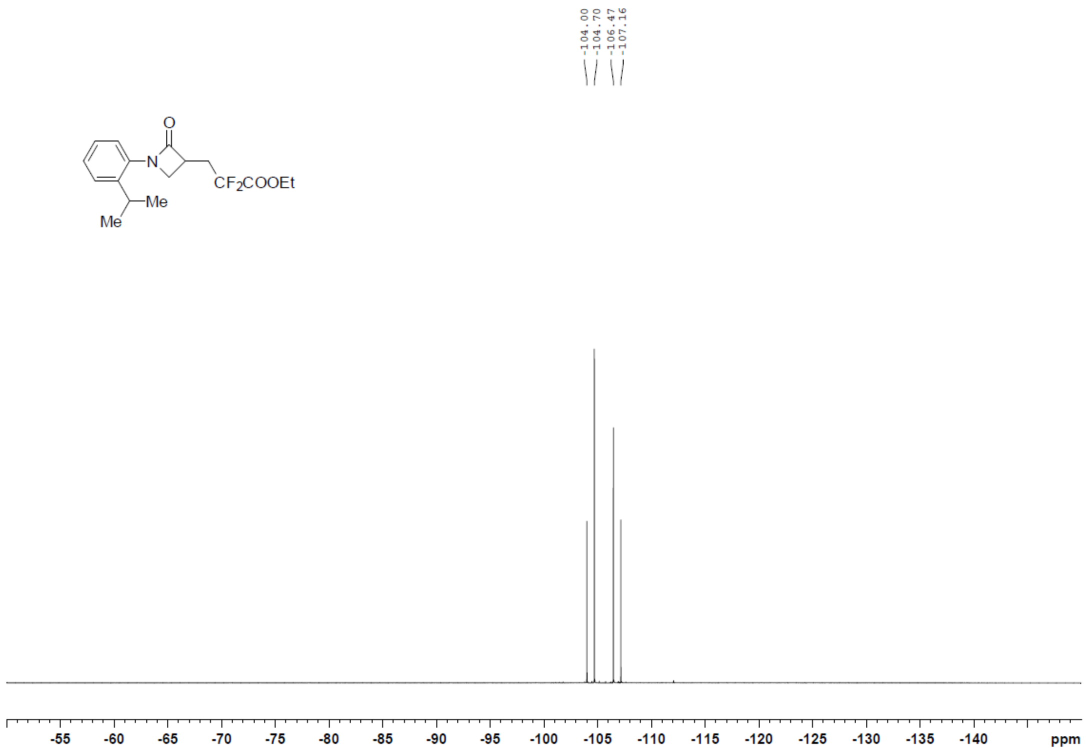

Supplementary Figure 156. ^{19}F NMR spectrum of **4i** in $CDCl_3$ (376 MHz)

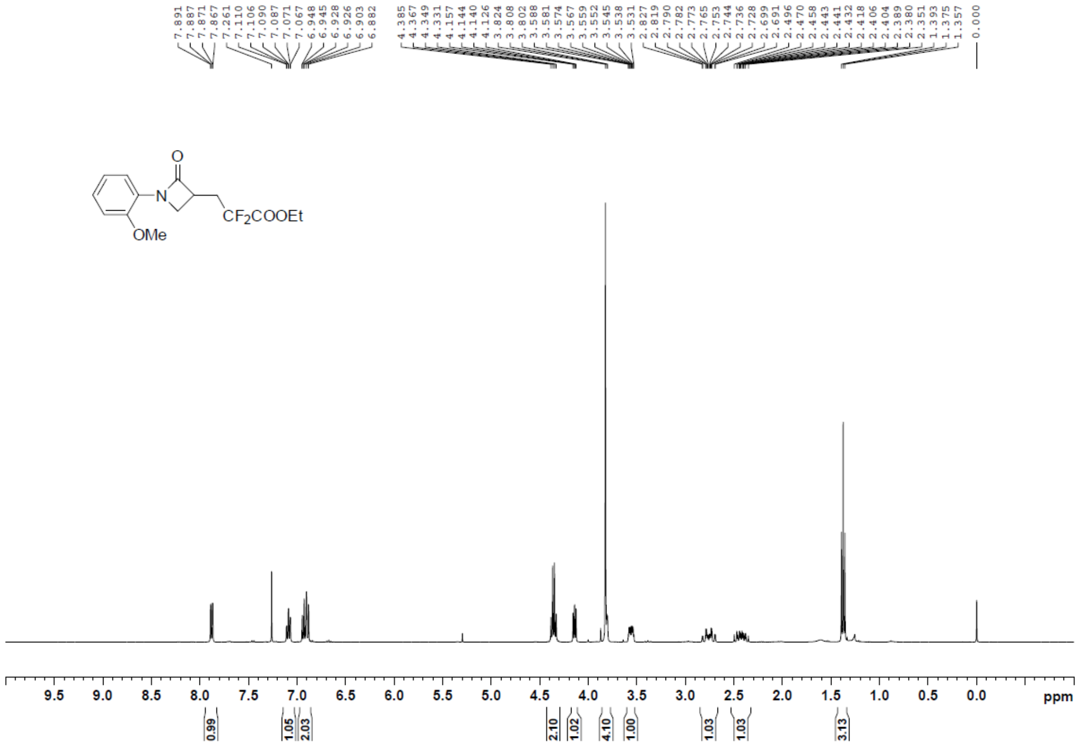

Supplementary Figure 157. ^{1}H NMR spectrum of **4j** in $CDCl_3$ (400 MHz)

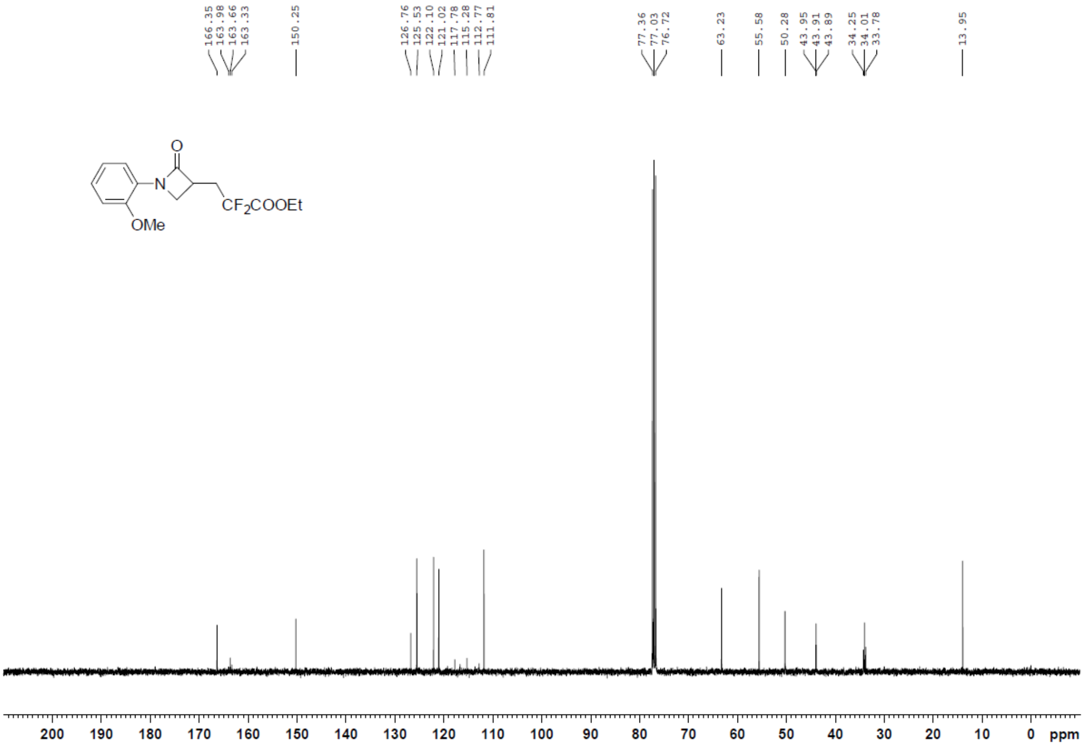

Supplementary Figure 158. ^{13}C NMR spectrum of **4j** in $CDCl_3$ (101 MHz)

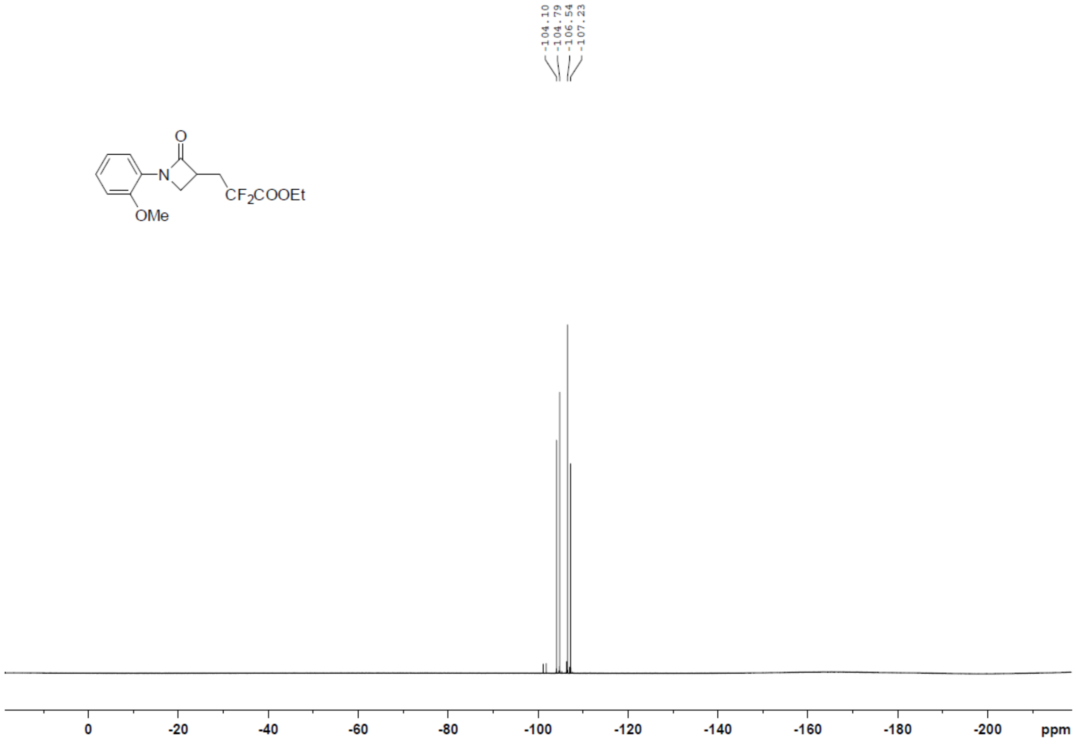

Supplementary Figure 159. ^{19}F NMR spectrum of **4j** in $CDCl_3$ (376 MHz)

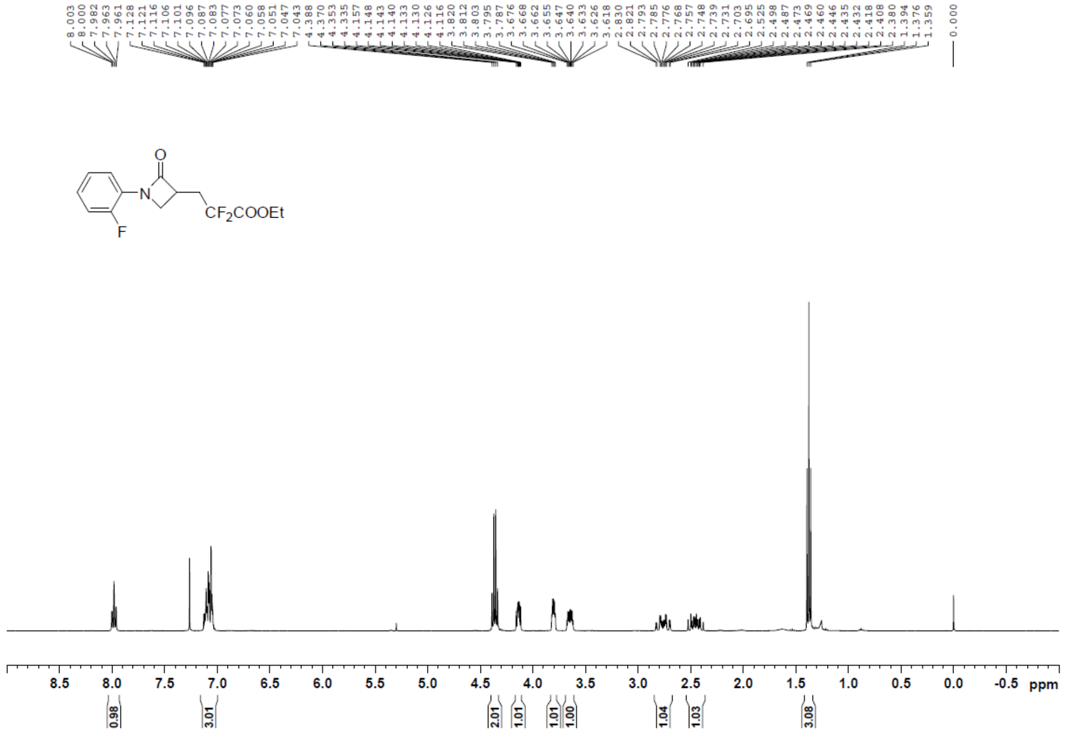

Supplementary Figure 160. ^{1}H NMR spectrum of **4k** in $CDCl_3$ (400 MHz)

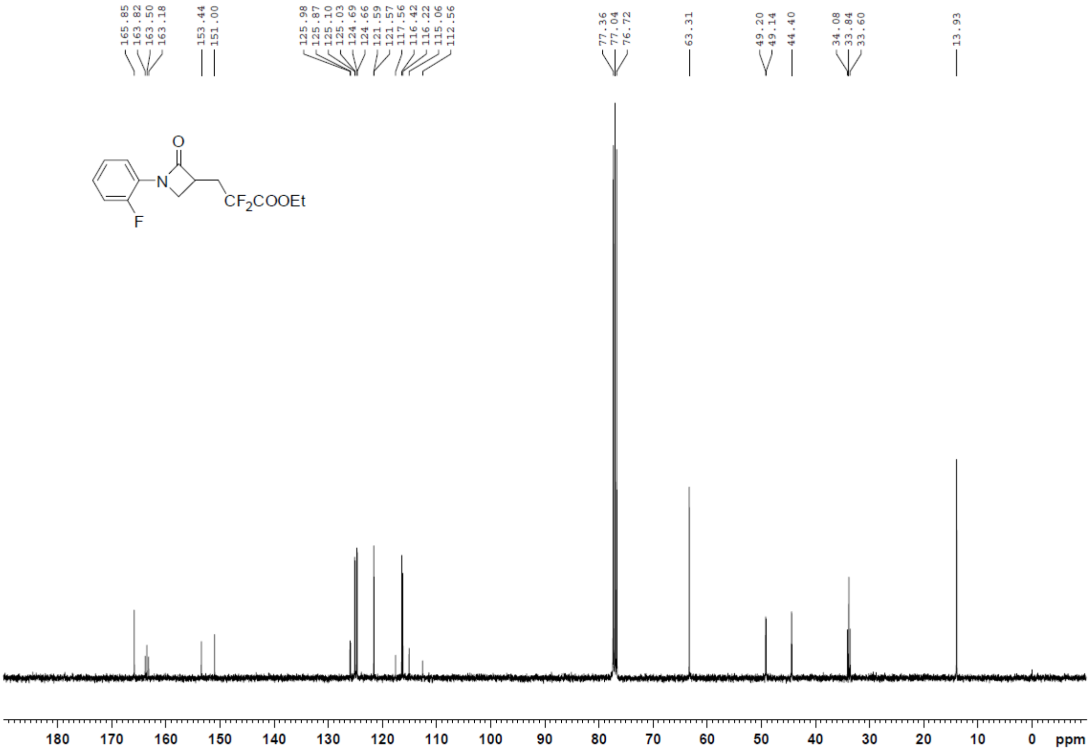

Supplementary Figure 161. ^{13}C NMR spectrum of **4k** in $CDCl_3$ (101 MHz)

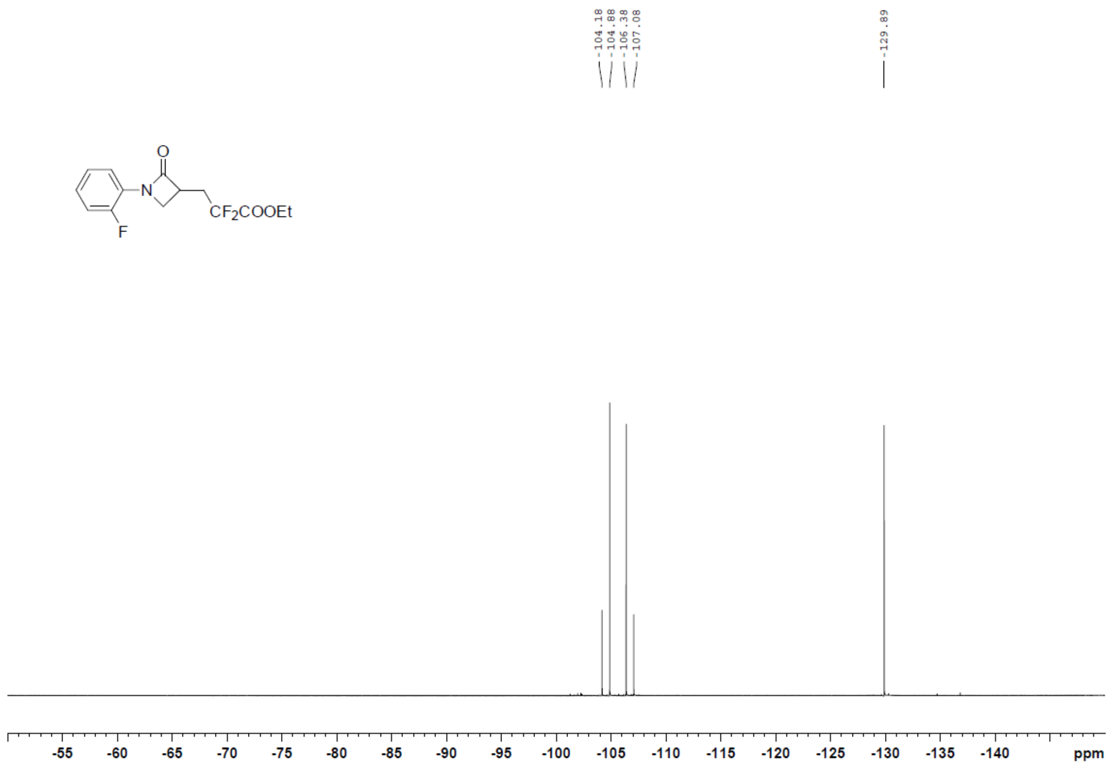

Supplementary Figure 162. ^{19}F NMR spectrum of **4k** in $CDCl_3$ (376 MHz)

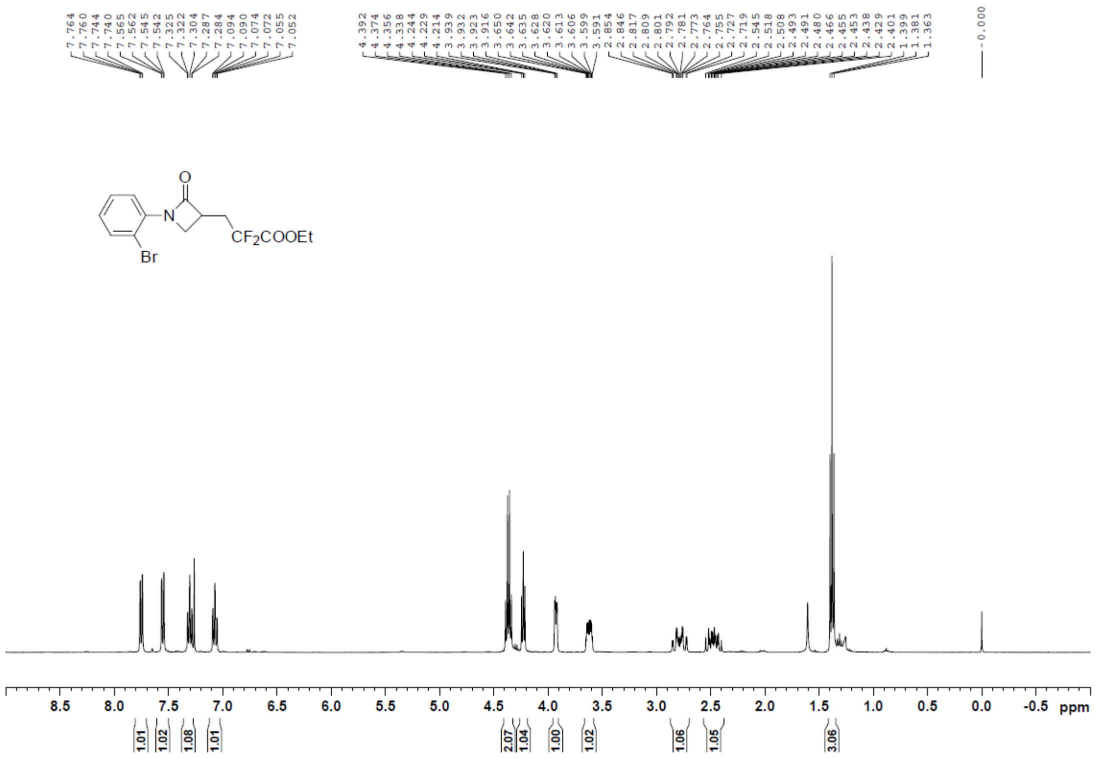

Supplementary Figure 163. ^{1}H NMR spectrum of **4l** in $CDCl_3$ (400 MHz)

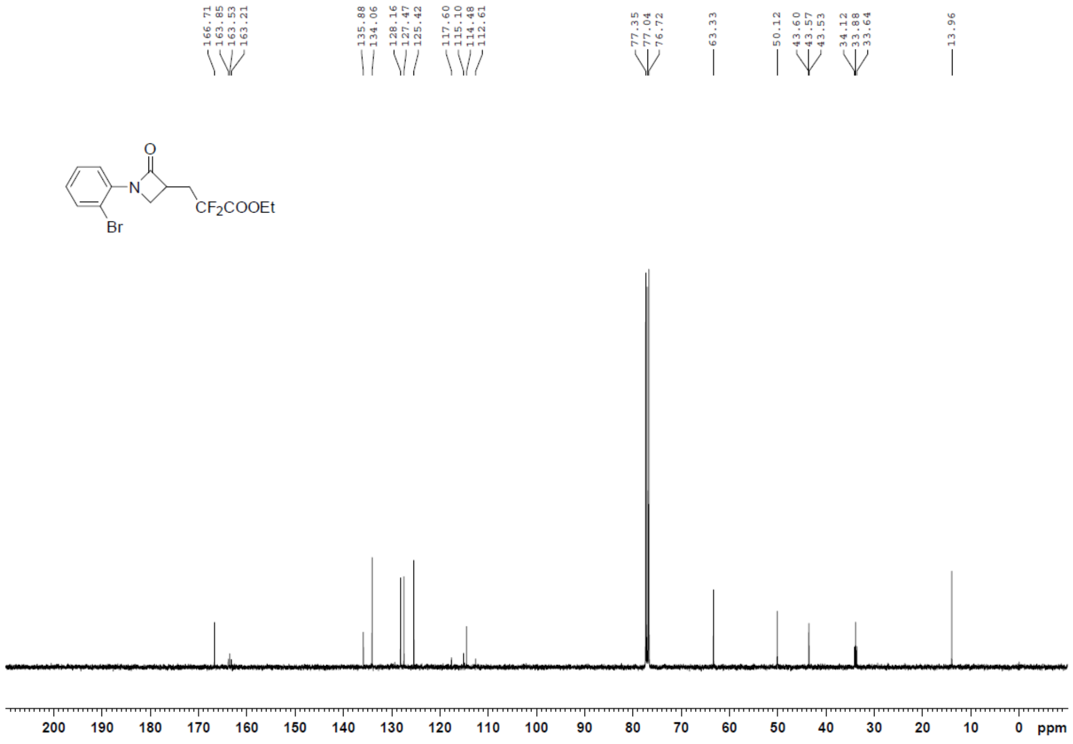

Supplementary Figure 164. ^{13}C NMR spectrum of **4l** in $CDCl_3$ (101 MHz)

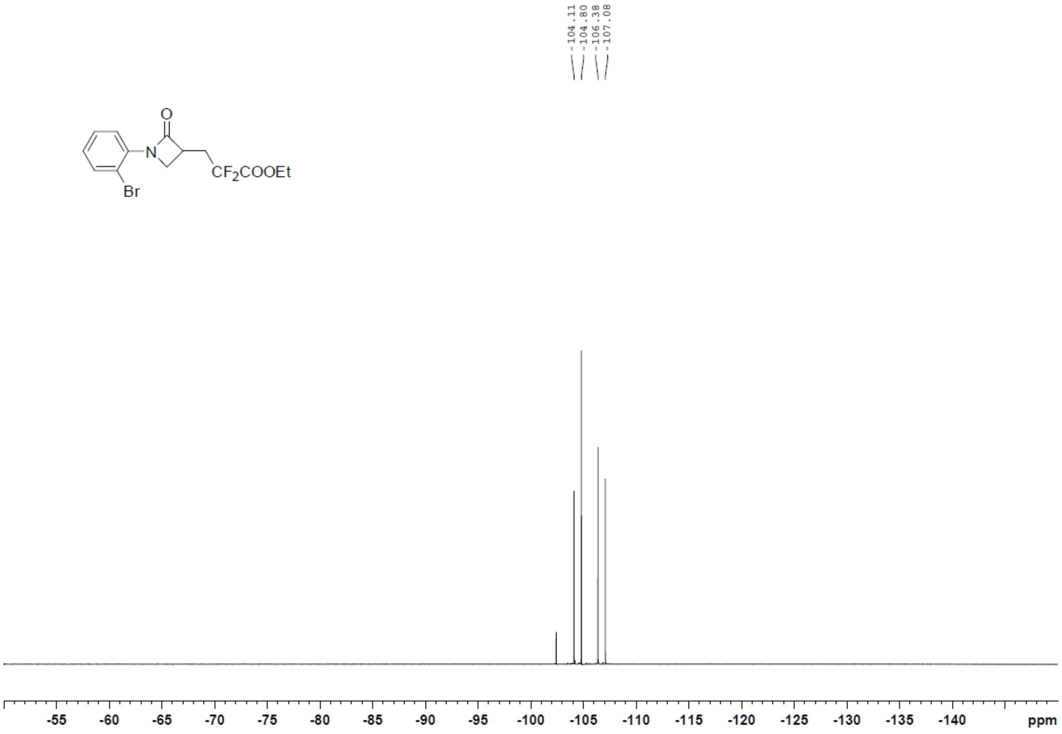

Supplementary Figure 165. ^{19}F NMR spectrum of **4l** in $CDCl_3$ (376 MHz)

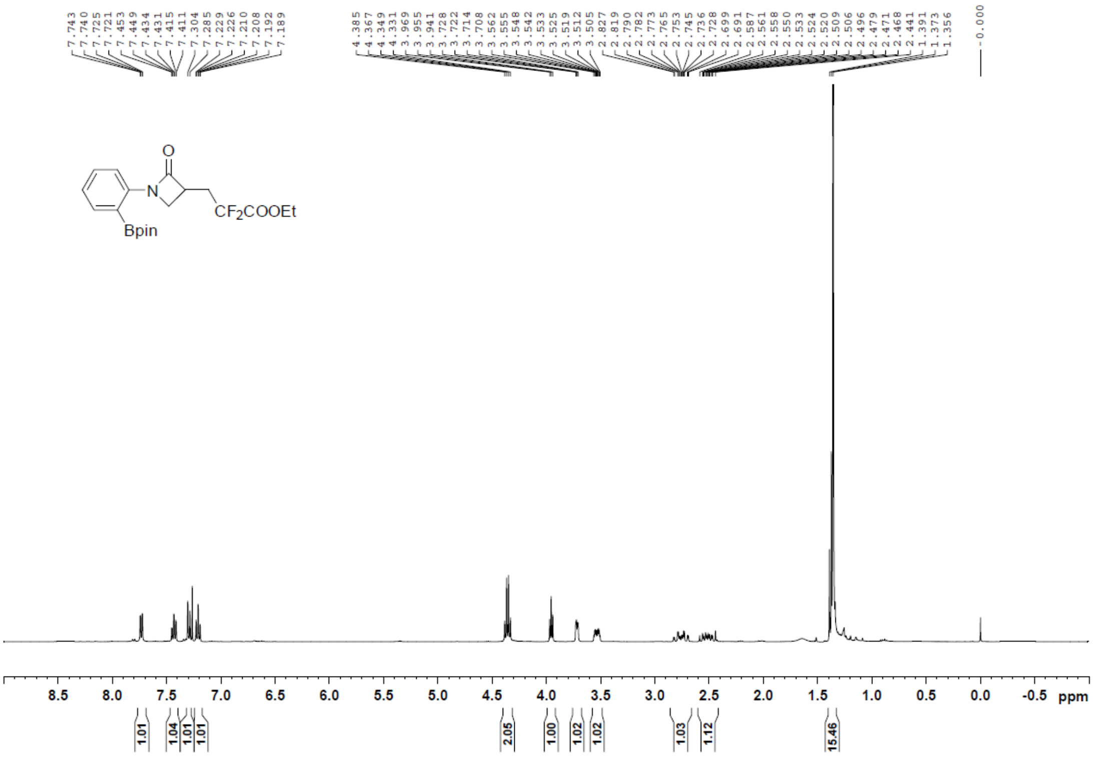

Supplementary Figure 166. ^{1}H NMR spectrum of **4m** in $CDCl_3$ (400 MHz)

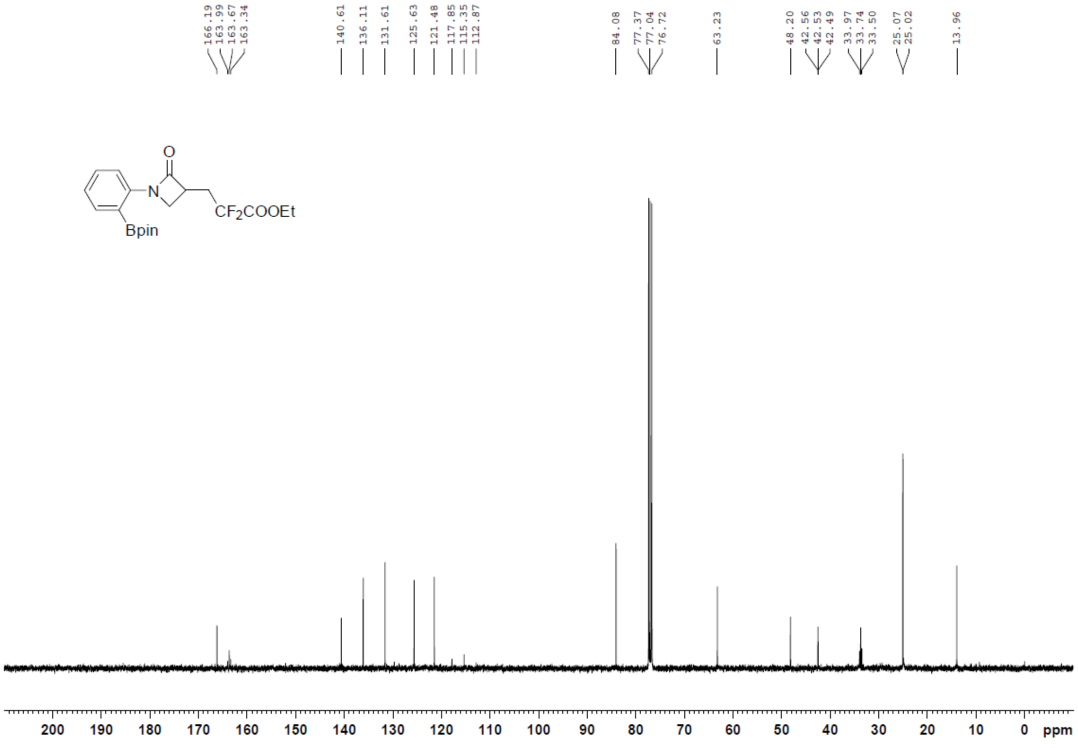

Supplementary Figure 167. ^{13}C NMR spectrum of **4m** in $CDCl_3$ (101 MHz)

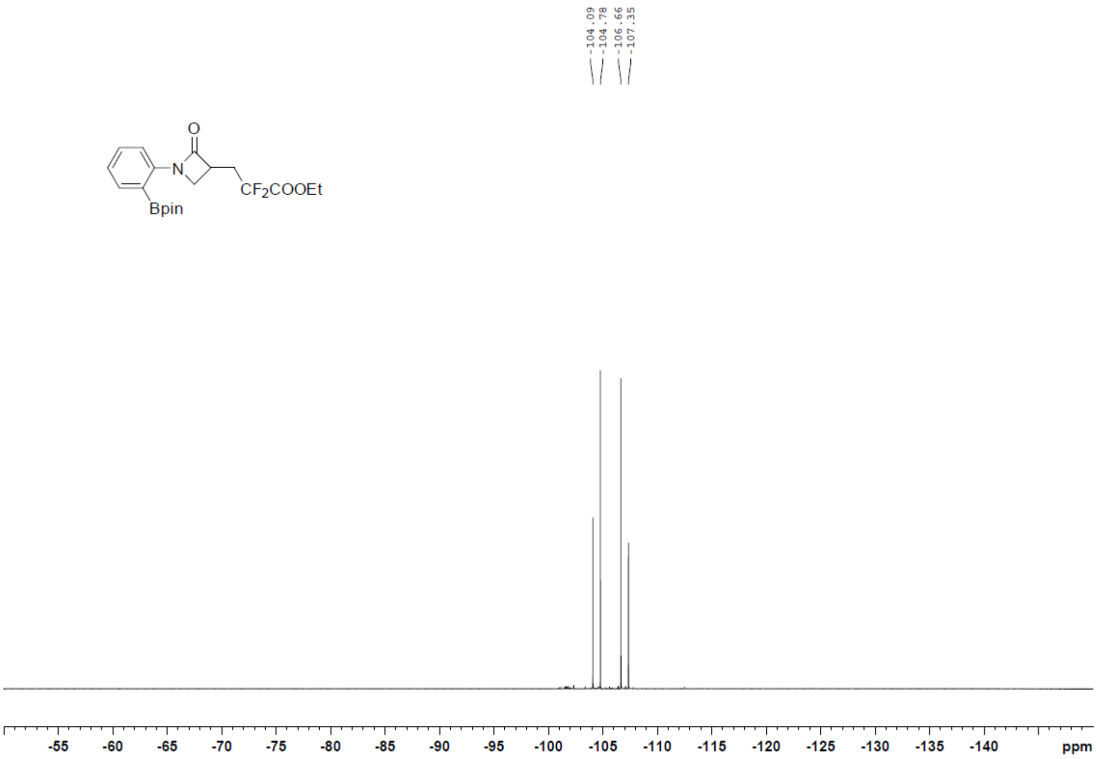

Supplementary Figure 168. ^{19}F NMR spectrum of **4m** in $CDCl_3$ (376 MHz)

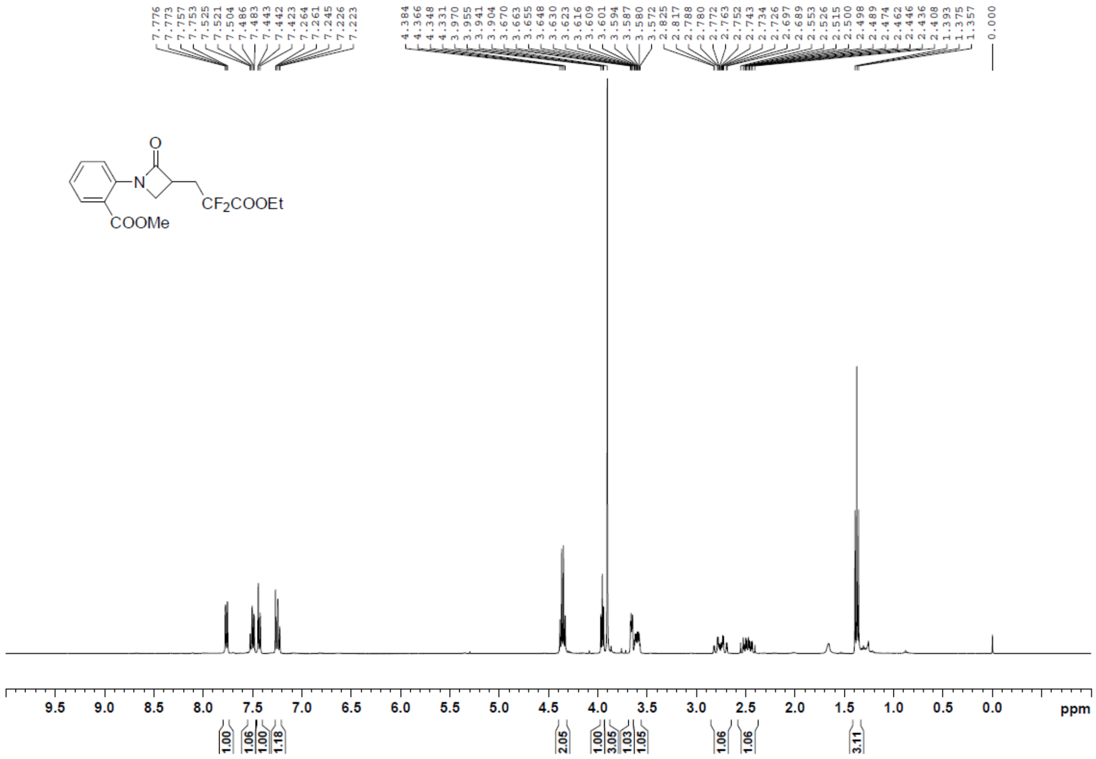

Supplementary Figure 169. ^{1}H NMR spectrum of **4n** in $CDCl_3$ (400 MHz)

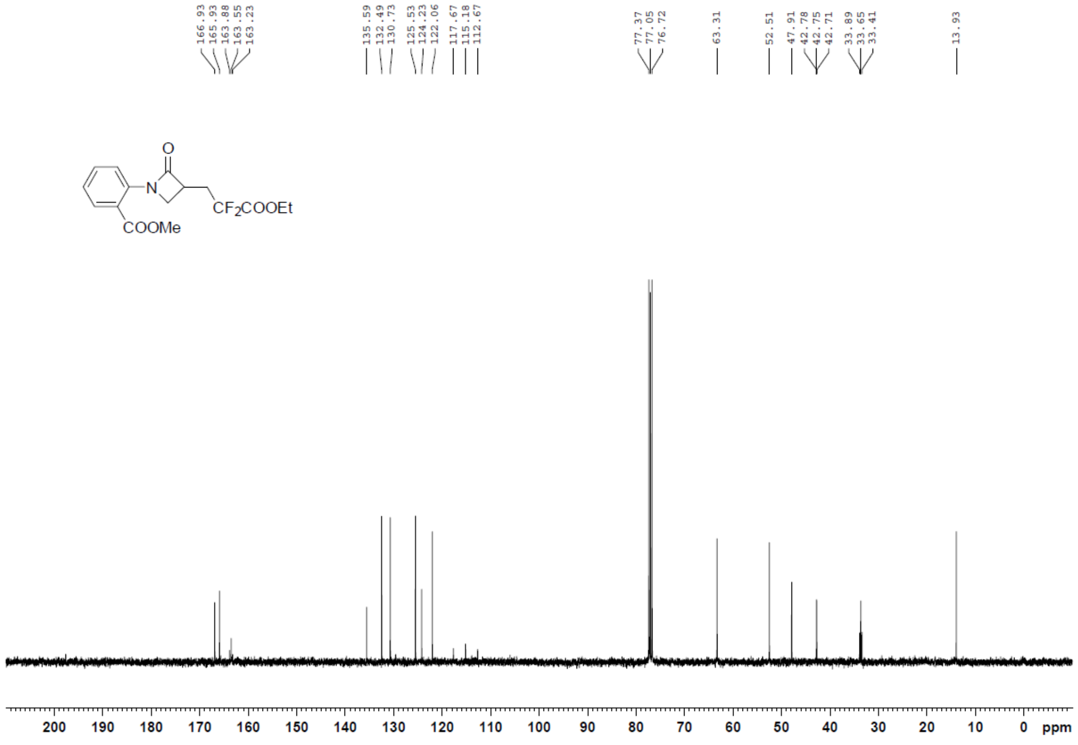

Supplementary Figure 170. ^{13}C NMR spectrum of **4n** in $CDCl_3$ (101 MHz)

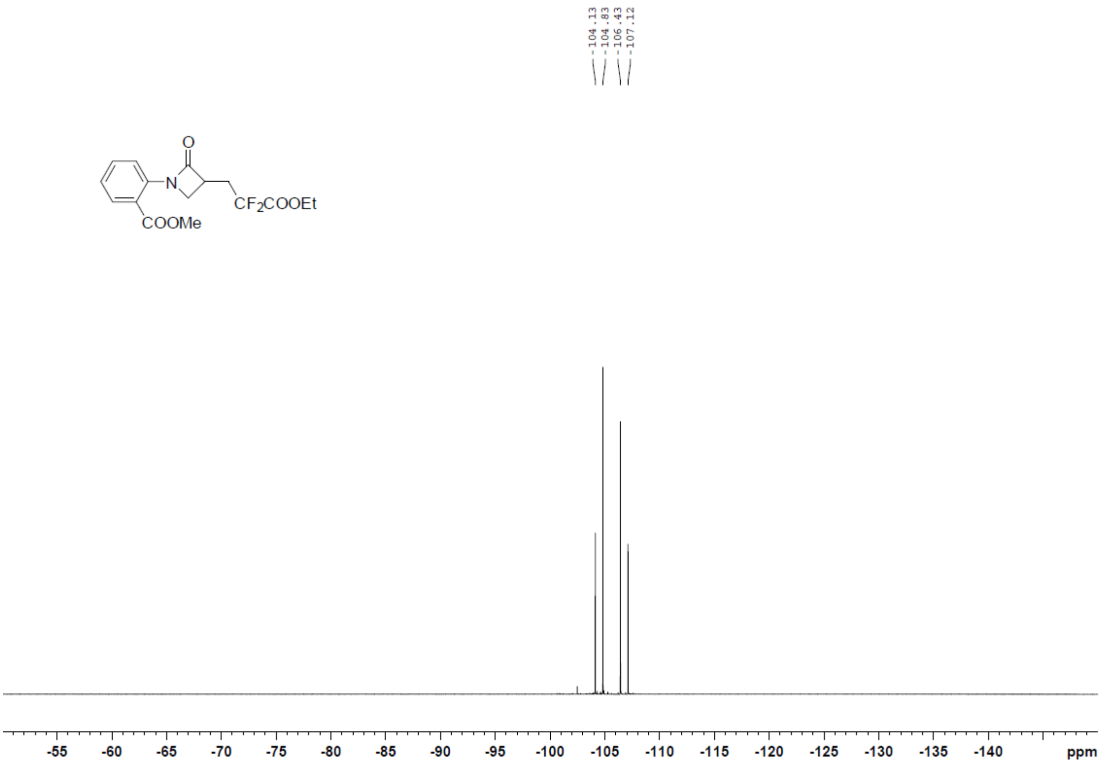

Supplementary Figure 171. ^{19}F NMR spectrum of **4n** in $CDCl_3$ (376 MHz)

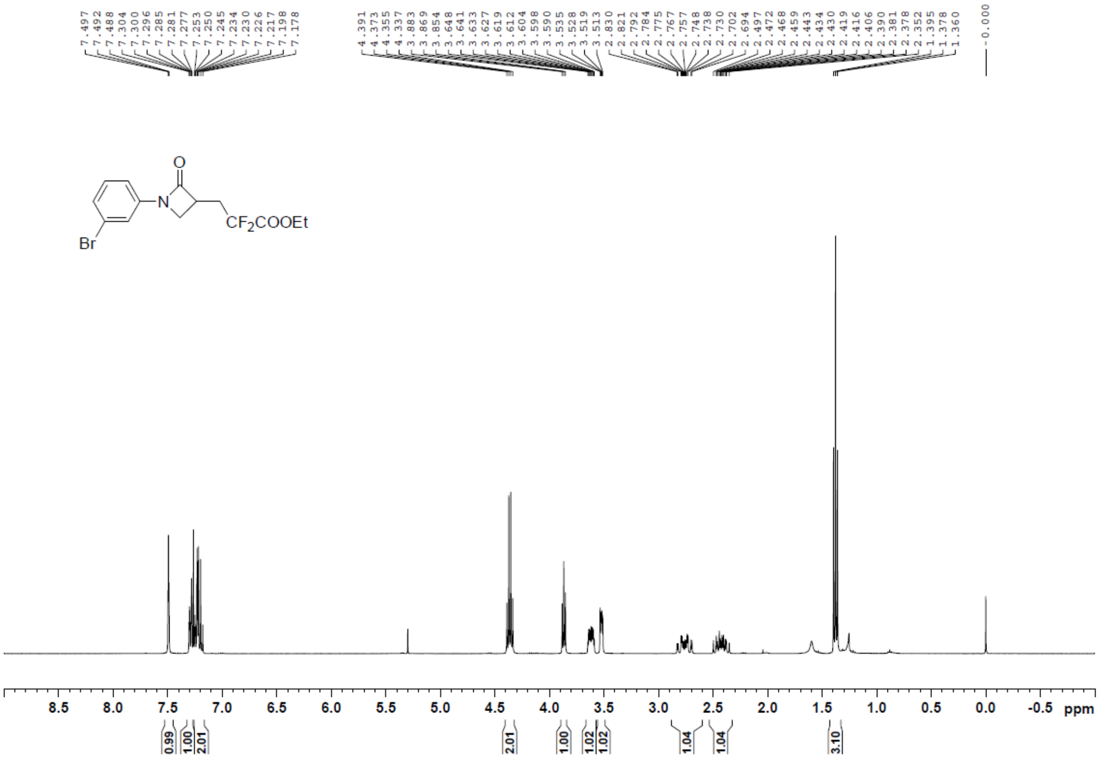

Supplementary Figure 172. ^{1}H NMR spectrum of **4o** in $CDCl_3$ (400 MHz)

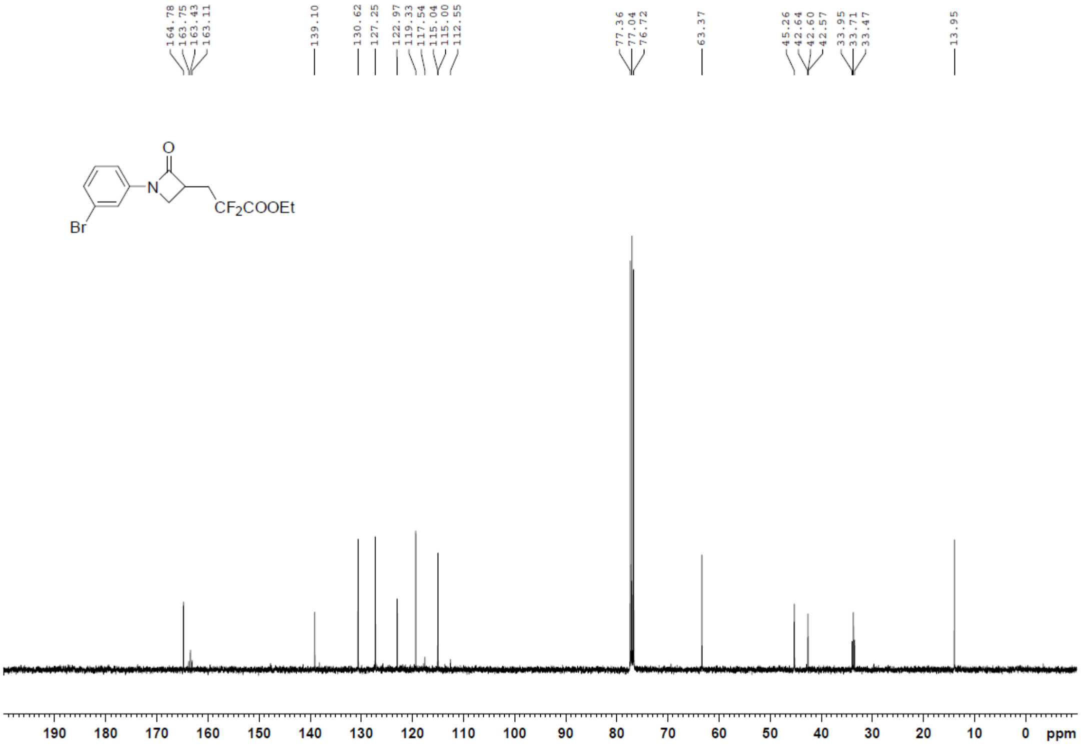

Supplementary Figure 173. ^{13}C NMR spectrum of **4o** in $CDCl_3$ (101 MHz)

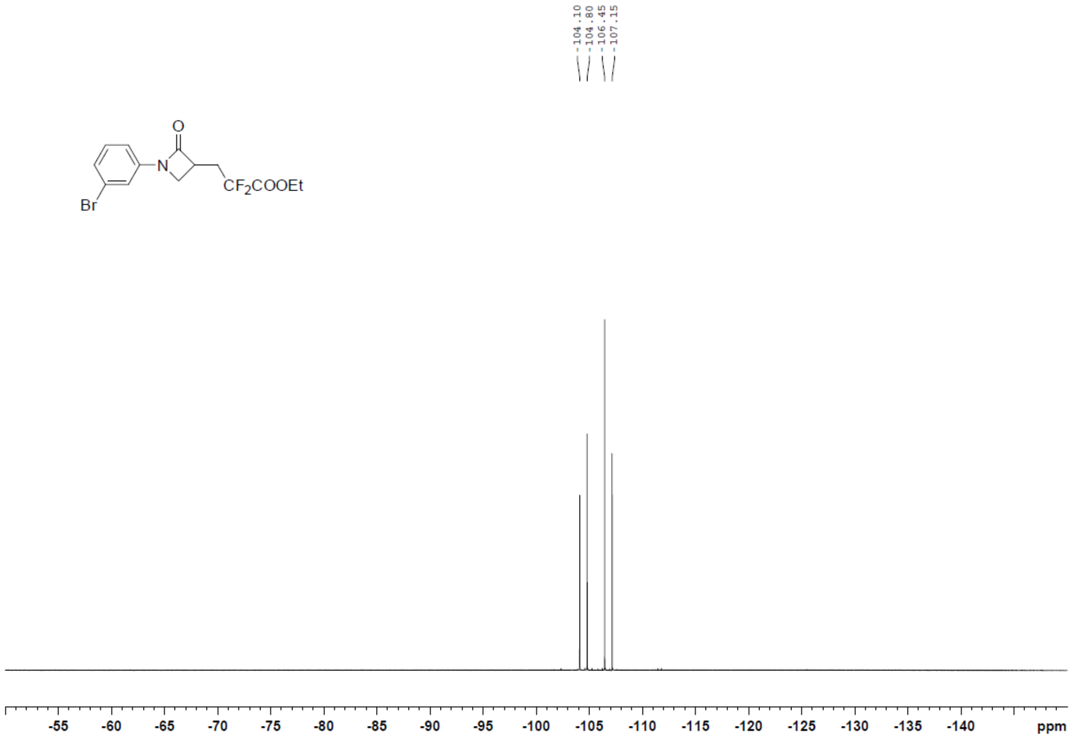

Supplementary Figure 174. ^{19}F NMR spectrum of **4o** in $CDCl_3$ (376 MHz)

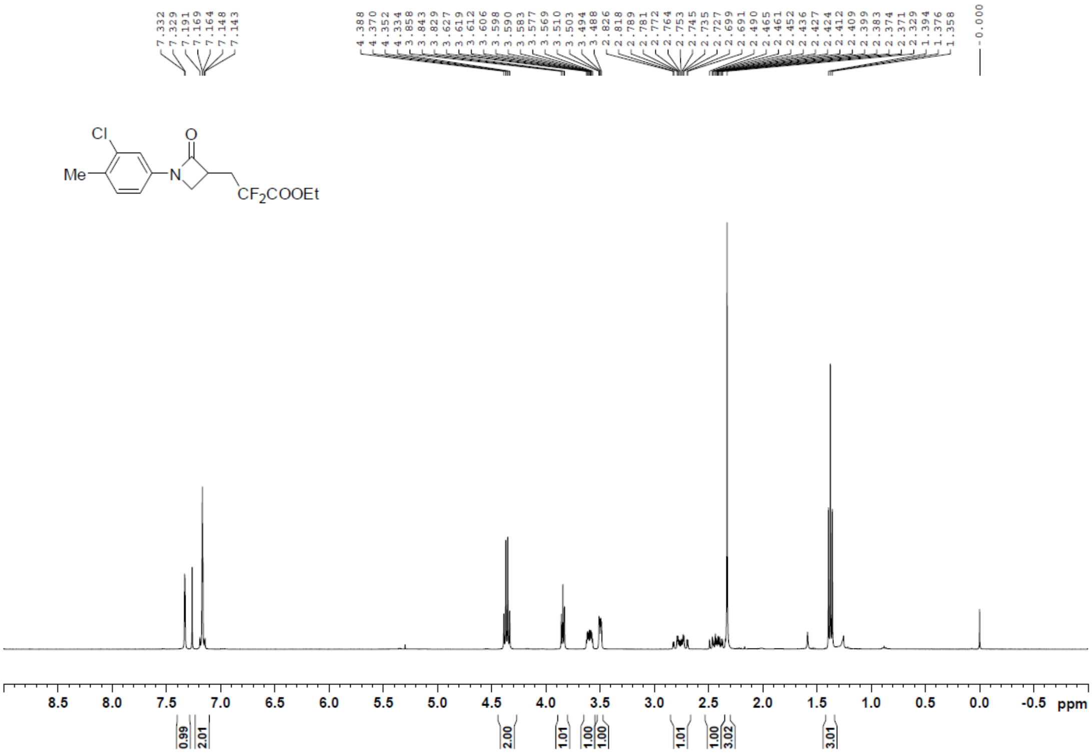

Supplementary Figure 175. ^{1}H NMR spectrum of **4p** in $CDCl_3$ (400 MHz)

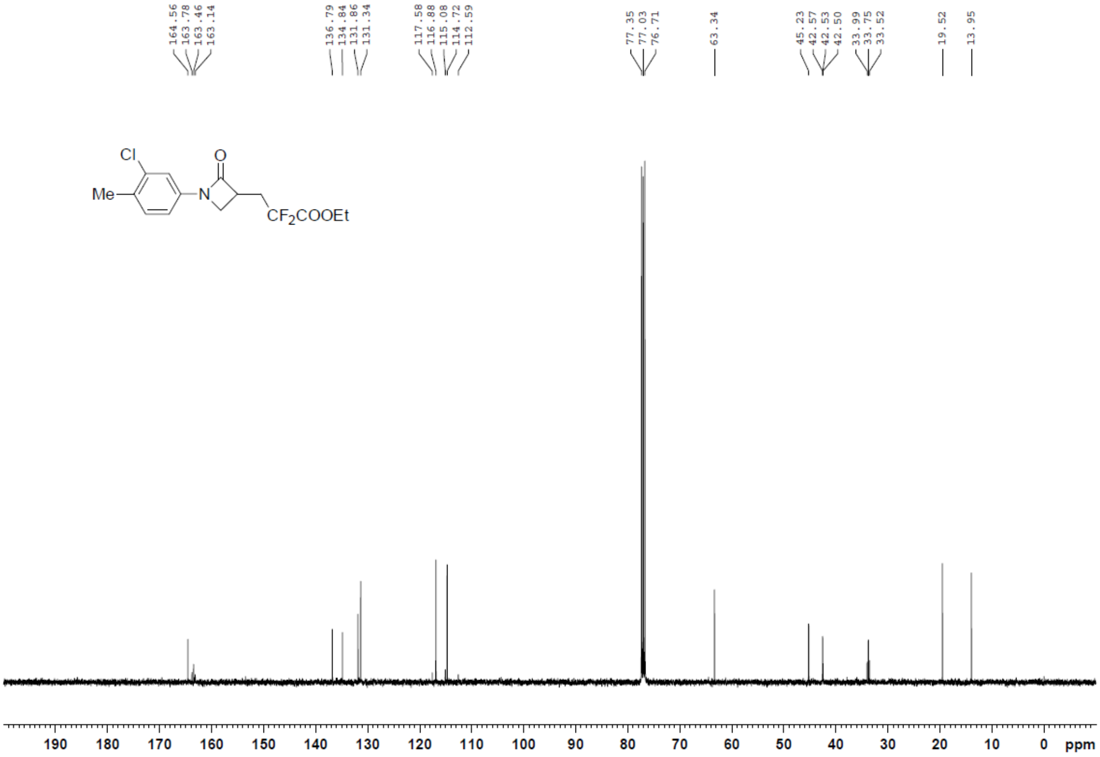

Supplementary Figure 176. ^{13}C NMR spectrum of **4p** in $CDCl_3$ (101 MHz)

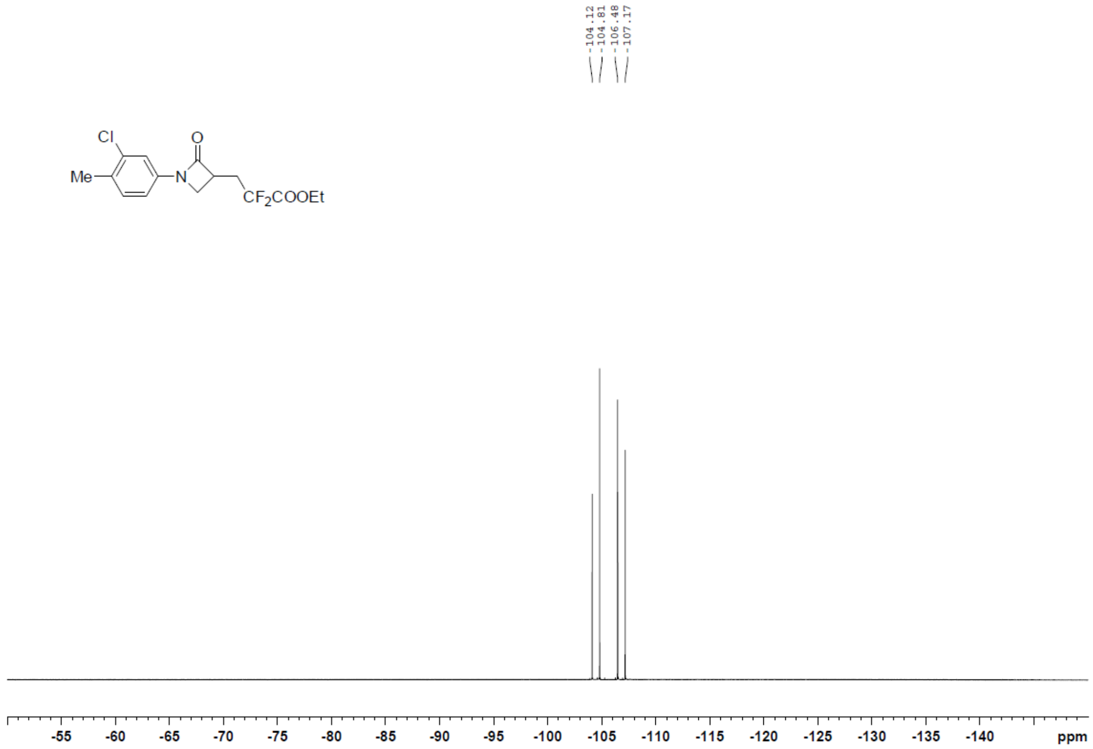

Supplementary Figure 177. ^{19}F NMR spectrum of **4p** in $CDCl_3$ (376 MHz)

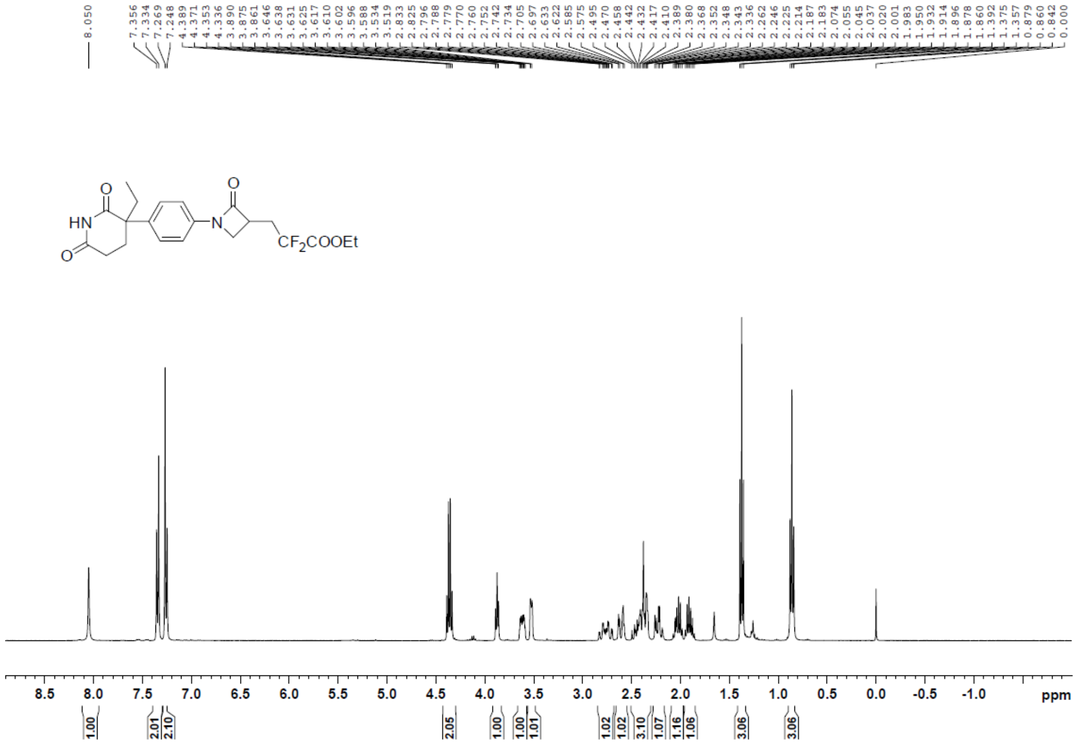

Supplementary Figure 178. ^{1}H NMR spectrum of **4q** in $CDCl_3$ (400 MHz)

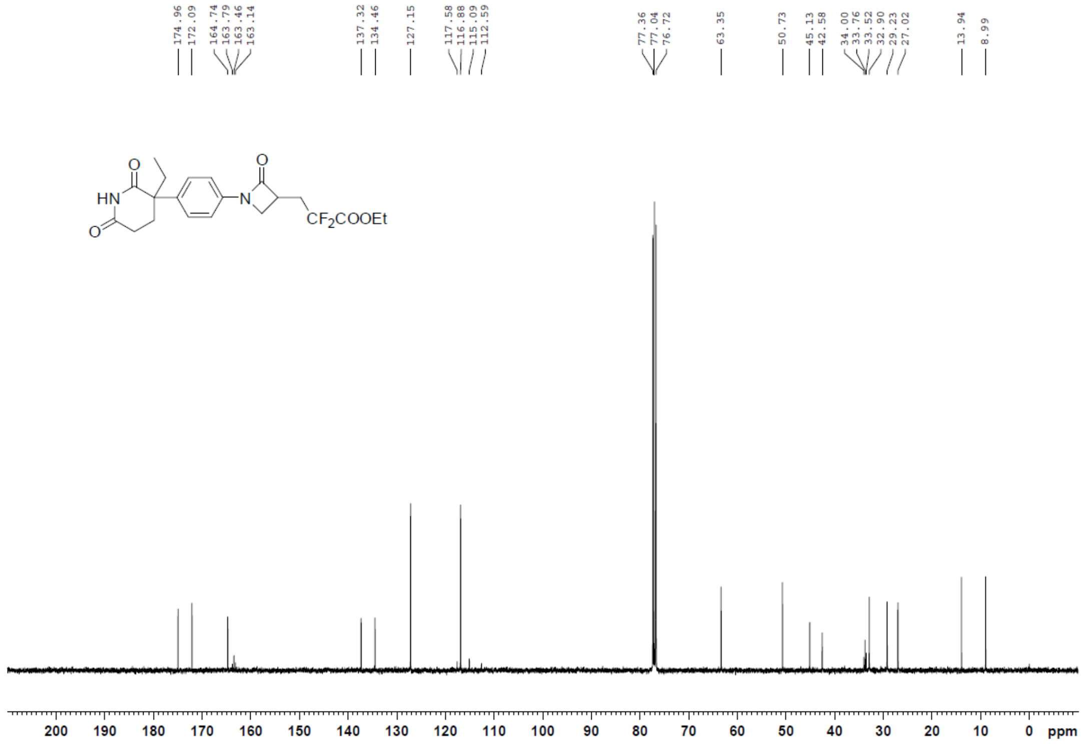

Supplementary Figure 179. ^{13}C NMR spectrum of **4q** in $CDCl_3$ (101 MHz)

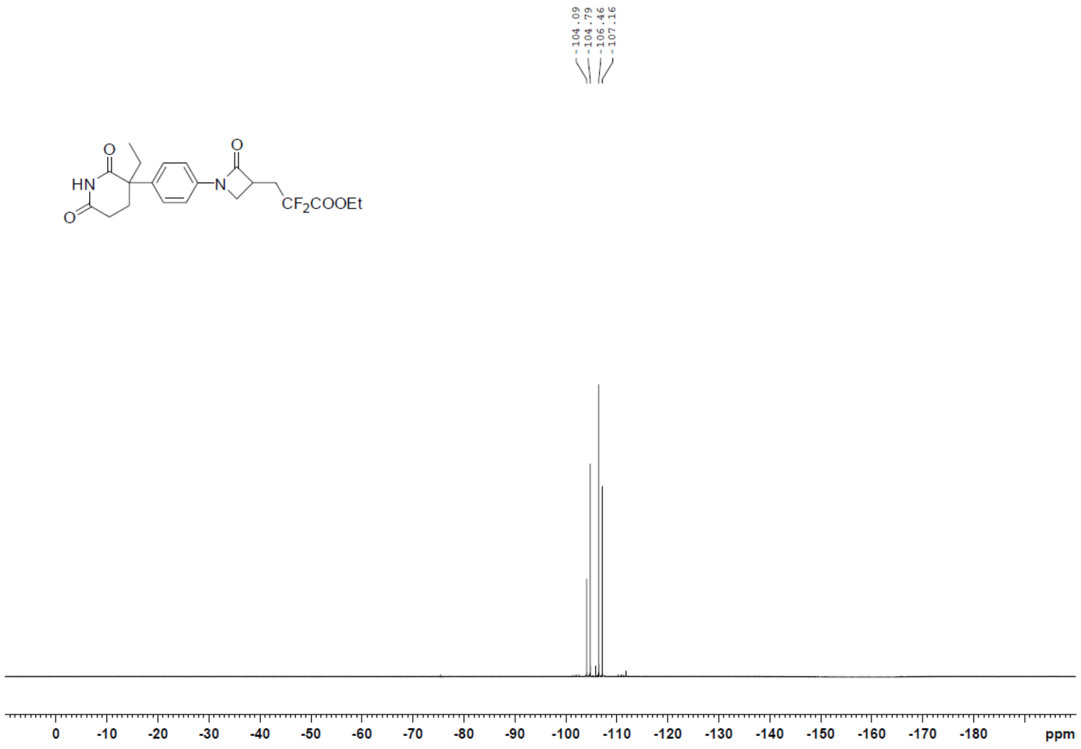

Supplementary Figure 180. ^{19}F NMR spectrum of **4q** in $CDCl_3$ (376 MHz)

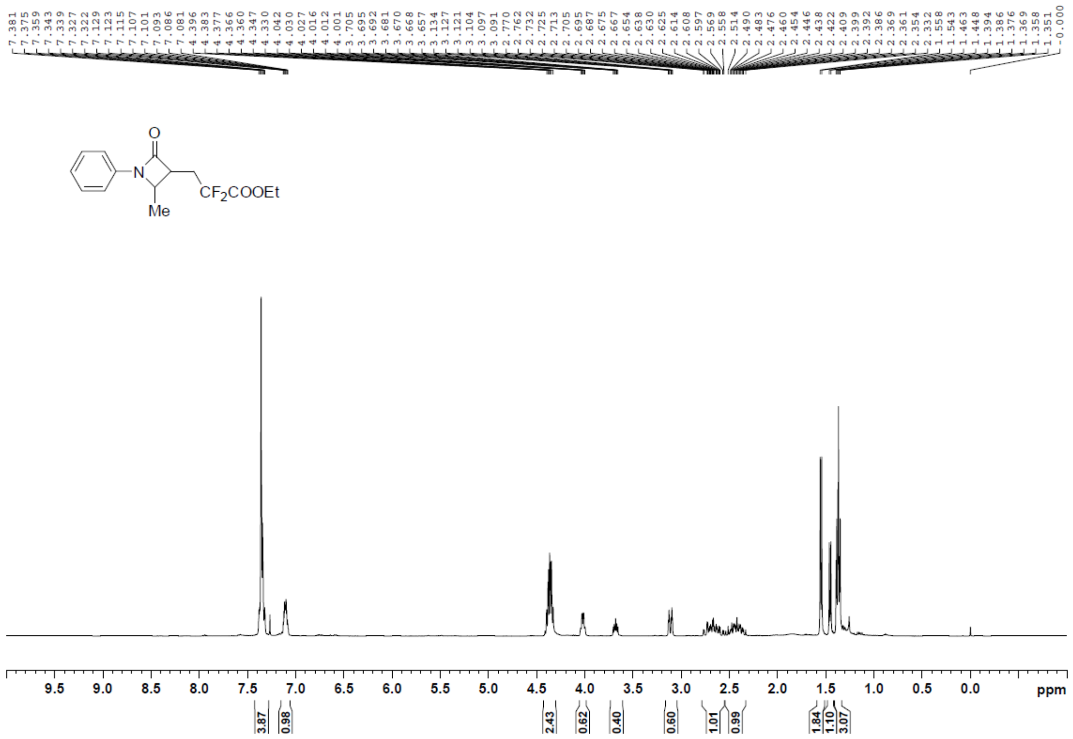

Supplementary Figure 181. ^{1}H NMR spectrum of **4r** in $CDCl_3$ (400 MHz)

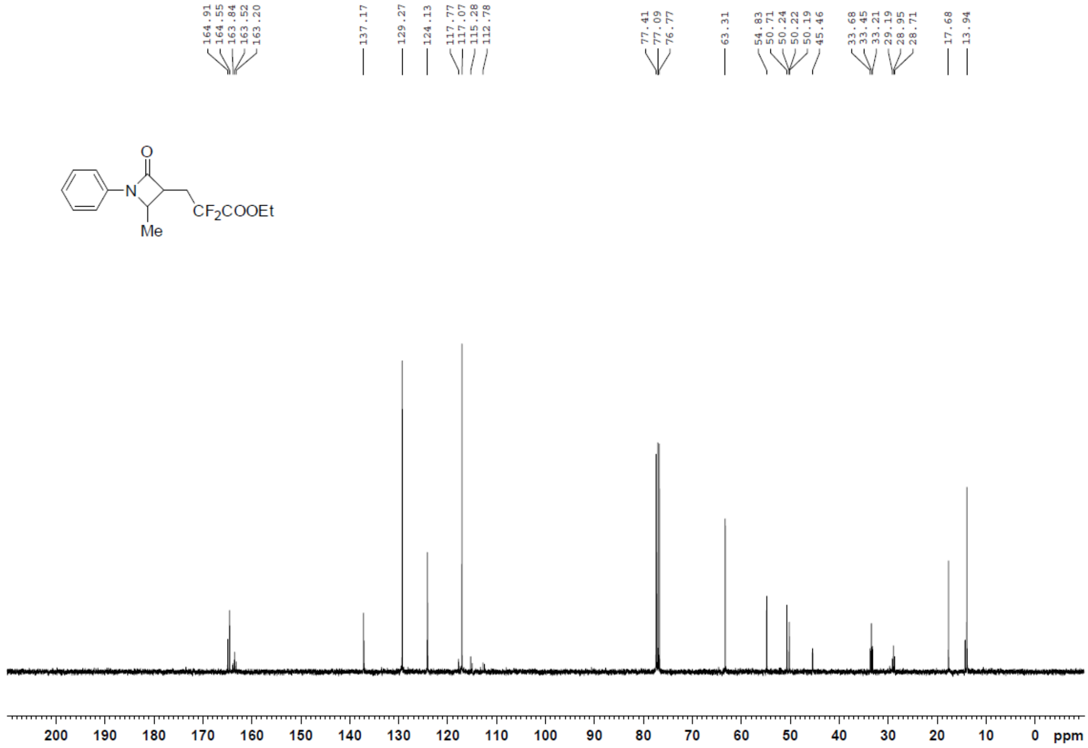

Supplementary Figure 182. ^{13}C NMR spectrum of **4r** in $CDCl_3$ (101 MHz)

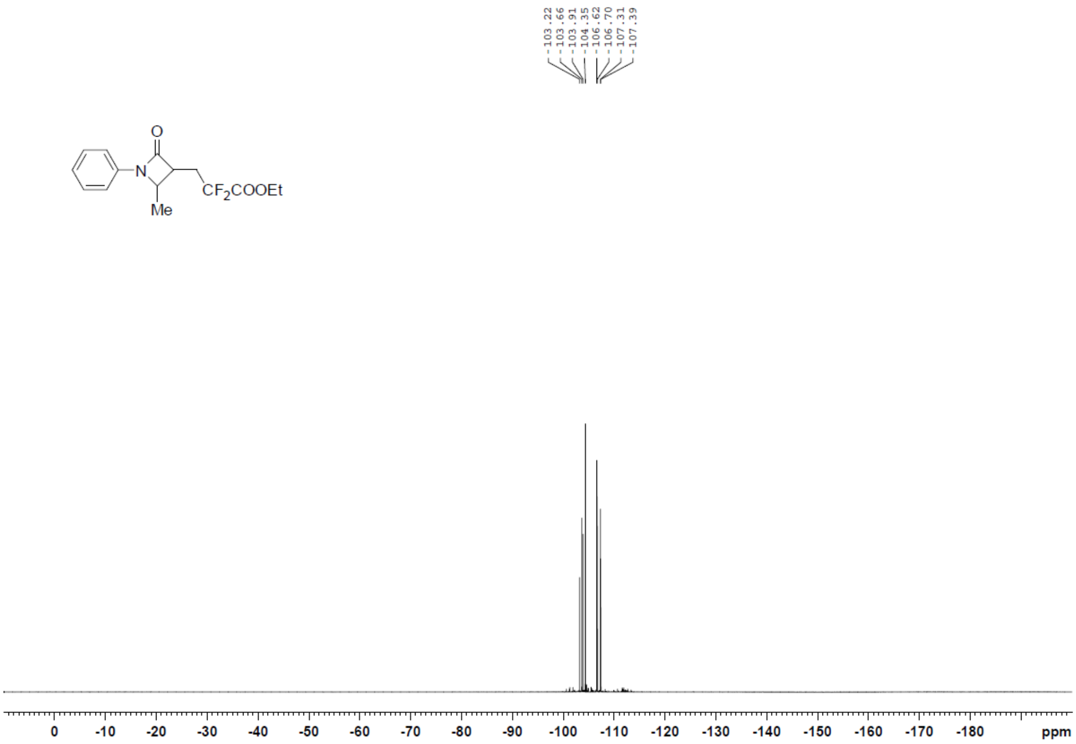

Supplementary Figure 183. ^{19}F NMR spectrum of **4r** in $CDCl_3$ (376 MHz)

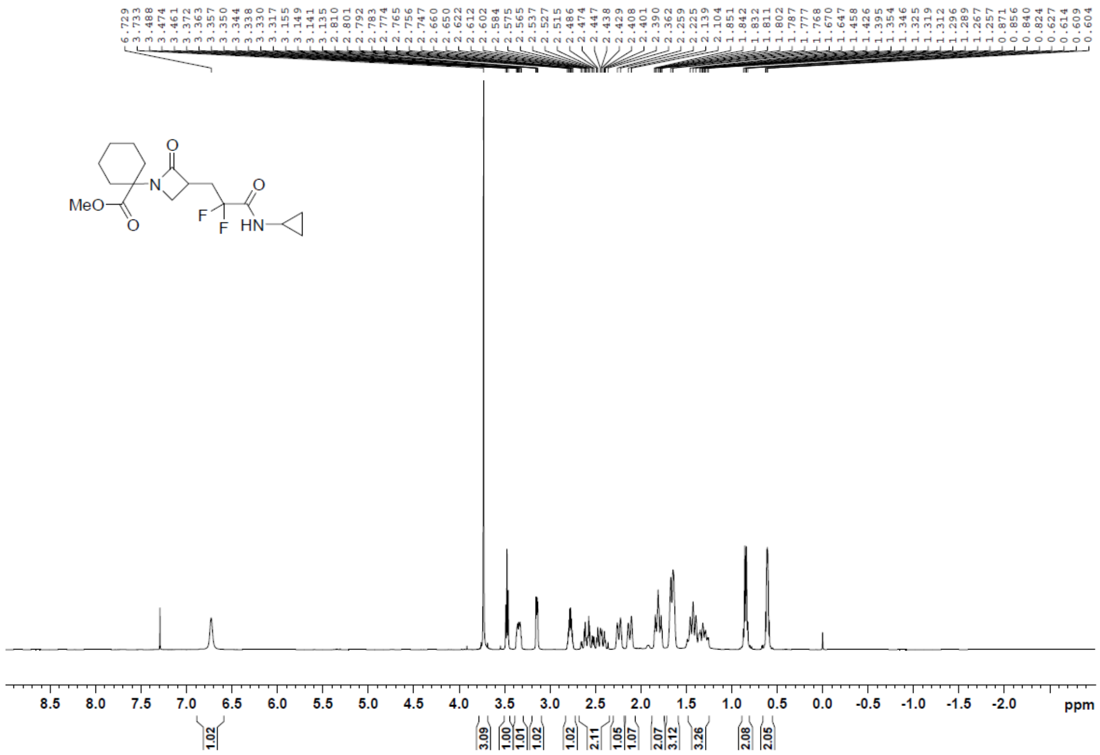

Supplementary Figure 184. ^{1}H NMR spectrum of **5a** in $CDCl_3$ (400 MHz)

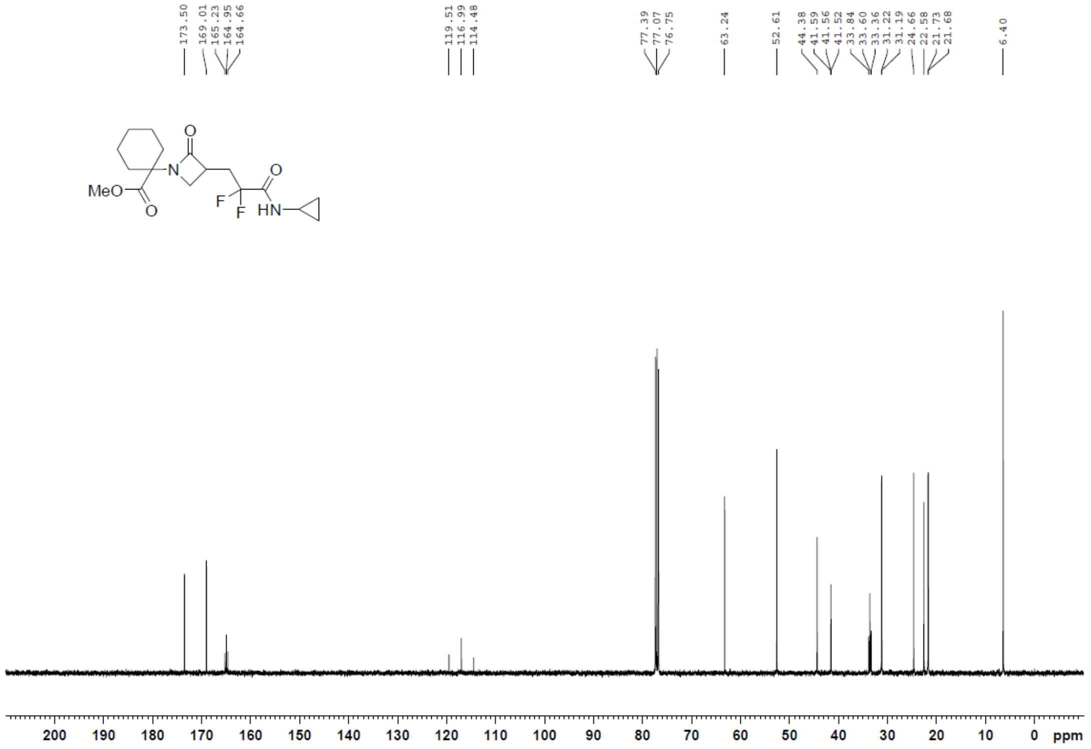

Supplementary Figure 185. ^{13}C NMR spectrum of **5a** in $CDCl_3$ (101 MHz)

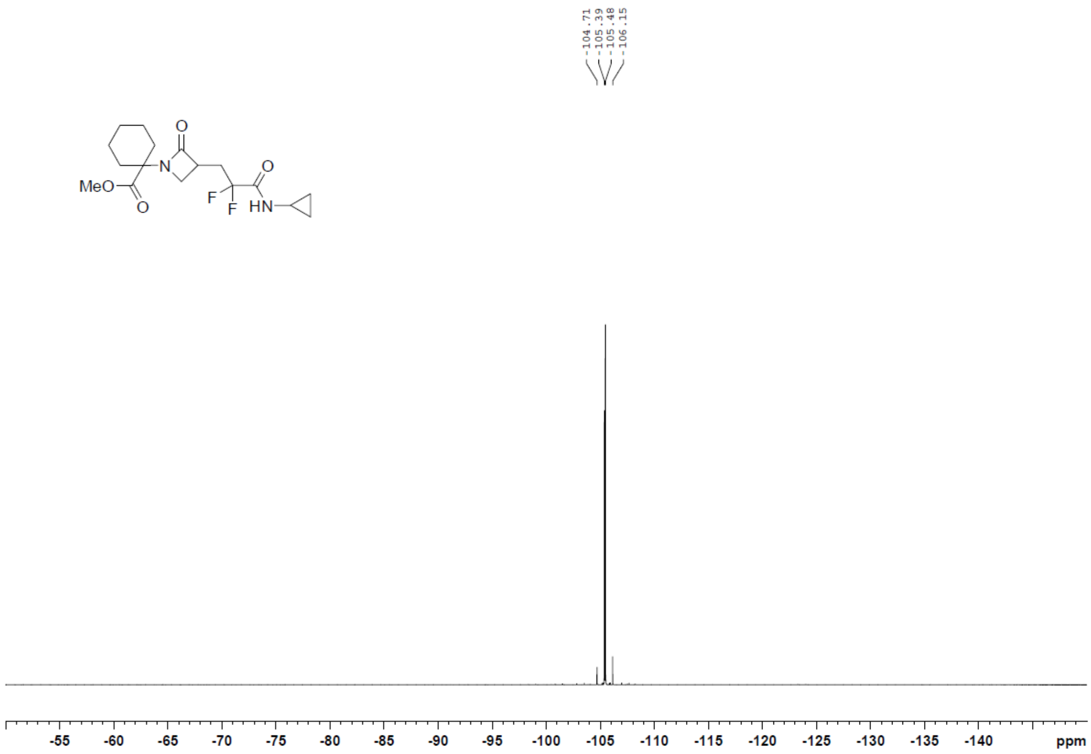

Supplementary Figure 186. ^{19}F NMR spectrum of **5a** in $CDCl_3$ (376 MHz)

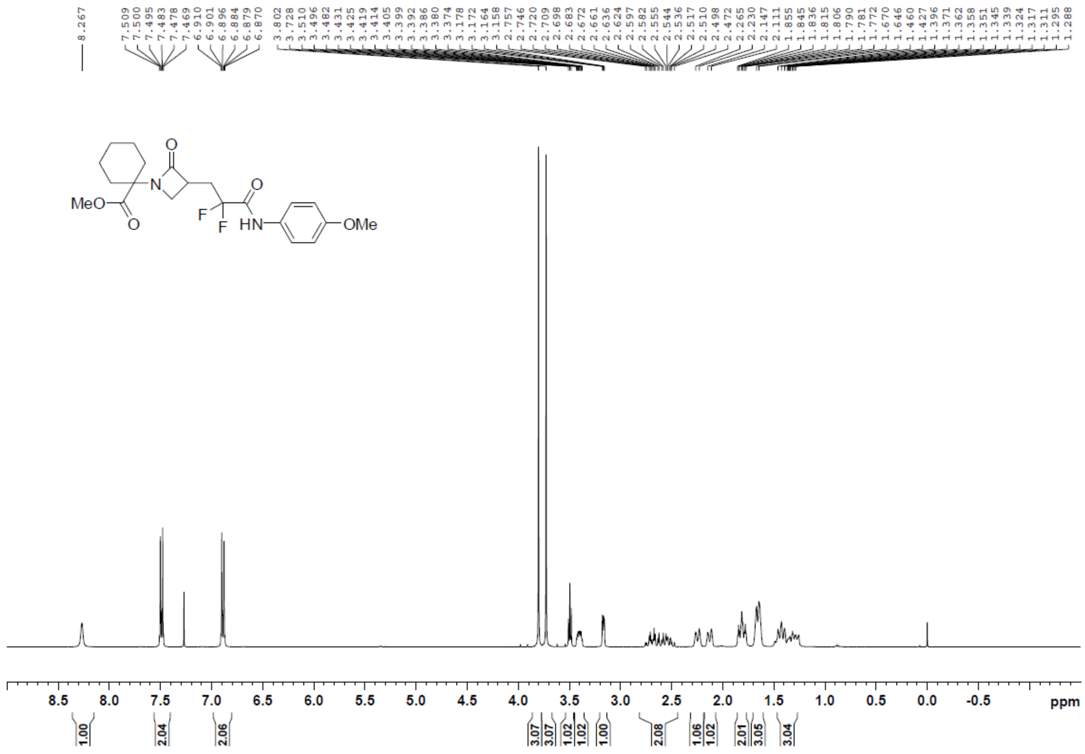

Supplementary Figure 187. ^{1}H NMR spectrum of **5b** in $CDCl_3$ (400 MHz)

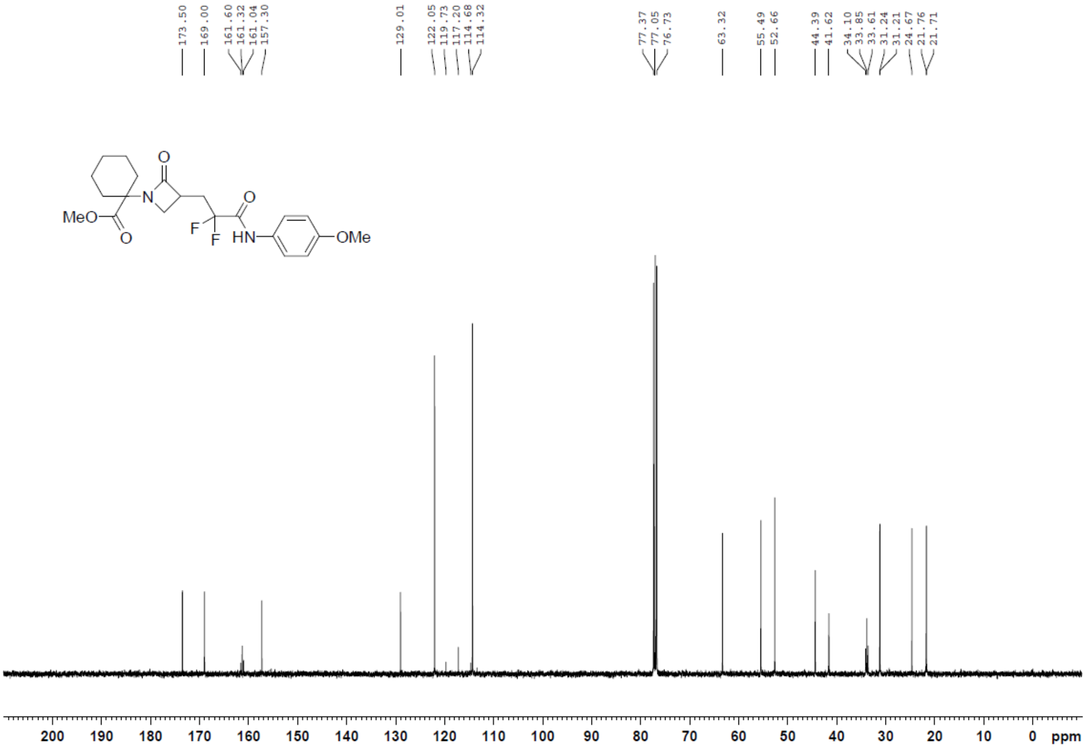

Supplementary Figure 188. ^{13}C NMR spectrum of **5b** in $CDCl_3$ (101 MHz)

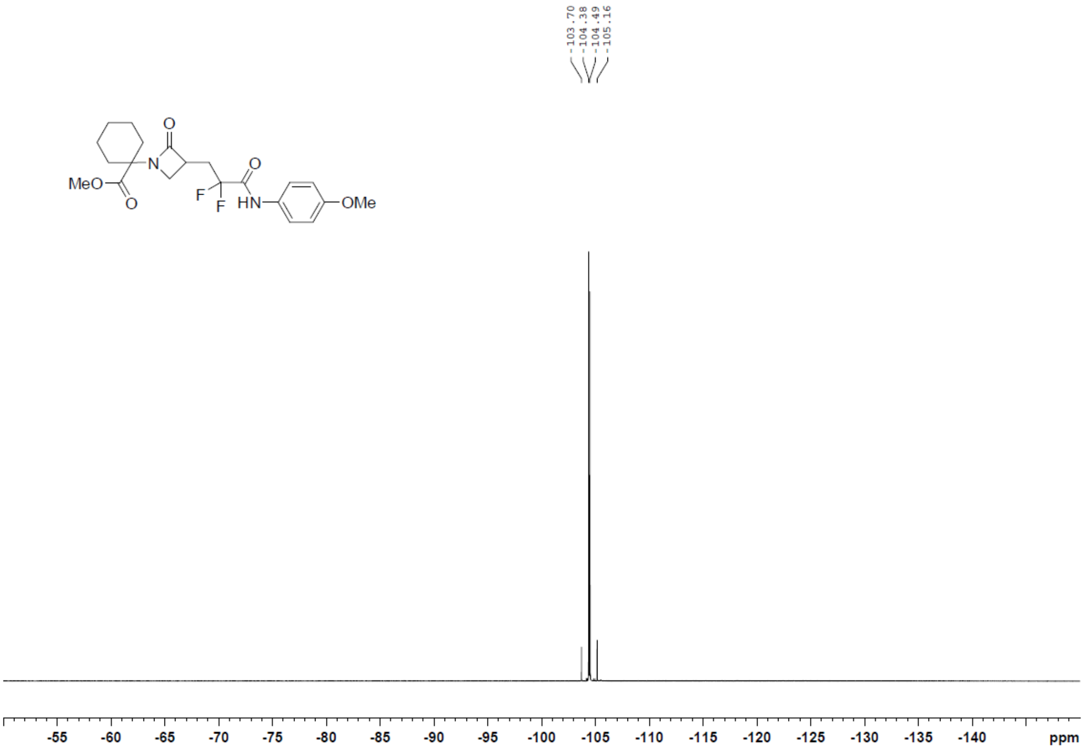

Supplementary Figure 189. ^{19}F NMR spectrum of **5b** in $CDCl_3$ (376 MHz)

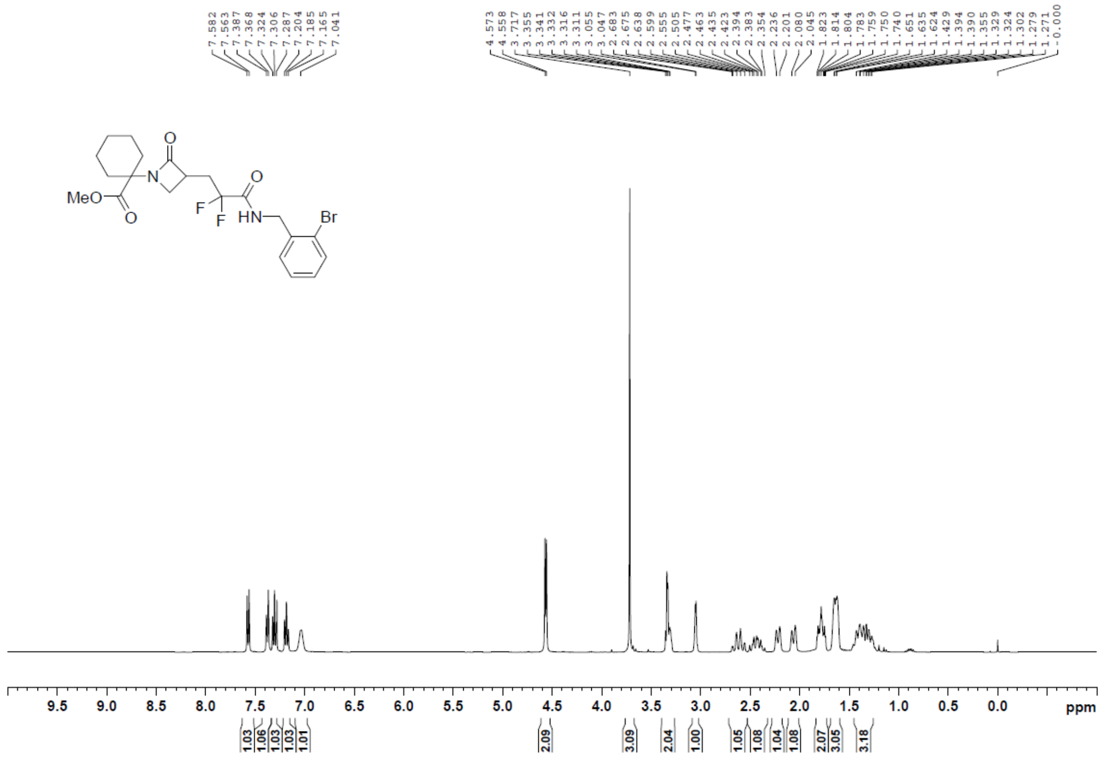

Supplementary Figure 190. ^{1}H NMR spectrum of **5c** in $CDCl_3$ (400 MHz)

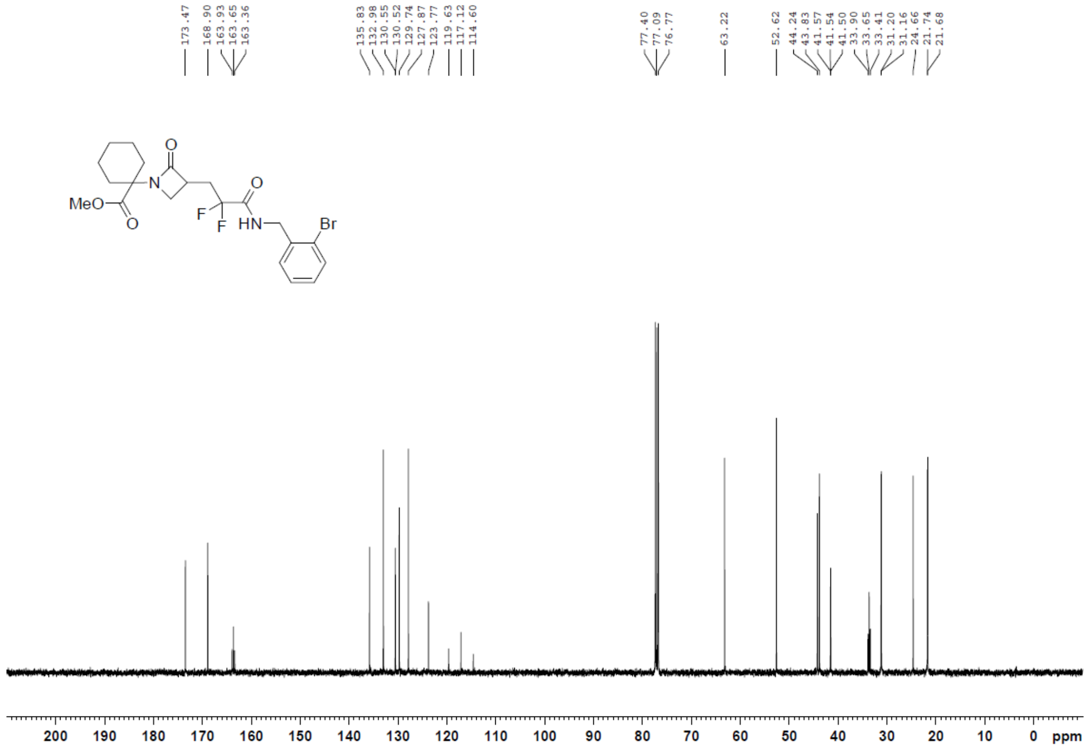

Supplementary Figure 191. ^{13}C NMR spectrum of **5c** in $CDCl_3$ (101 MHz)

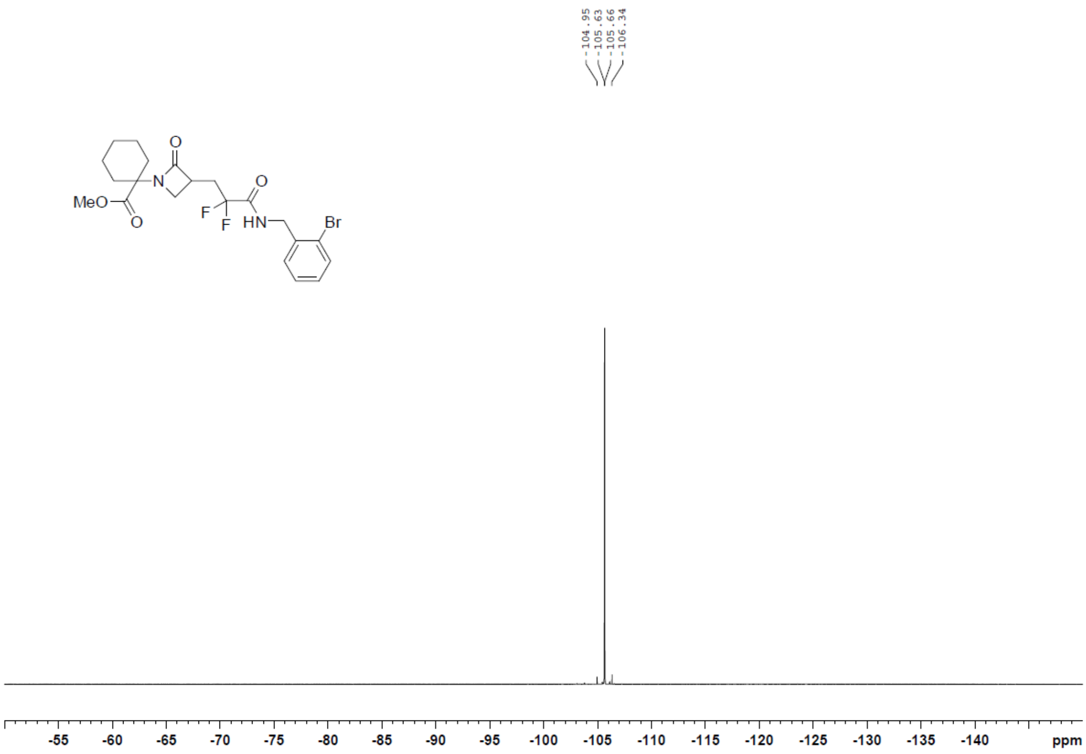

Supplementary Figure 192. ^{19}F NMR spectrum of **5c** in $CDCl_3$ (376 MHz)

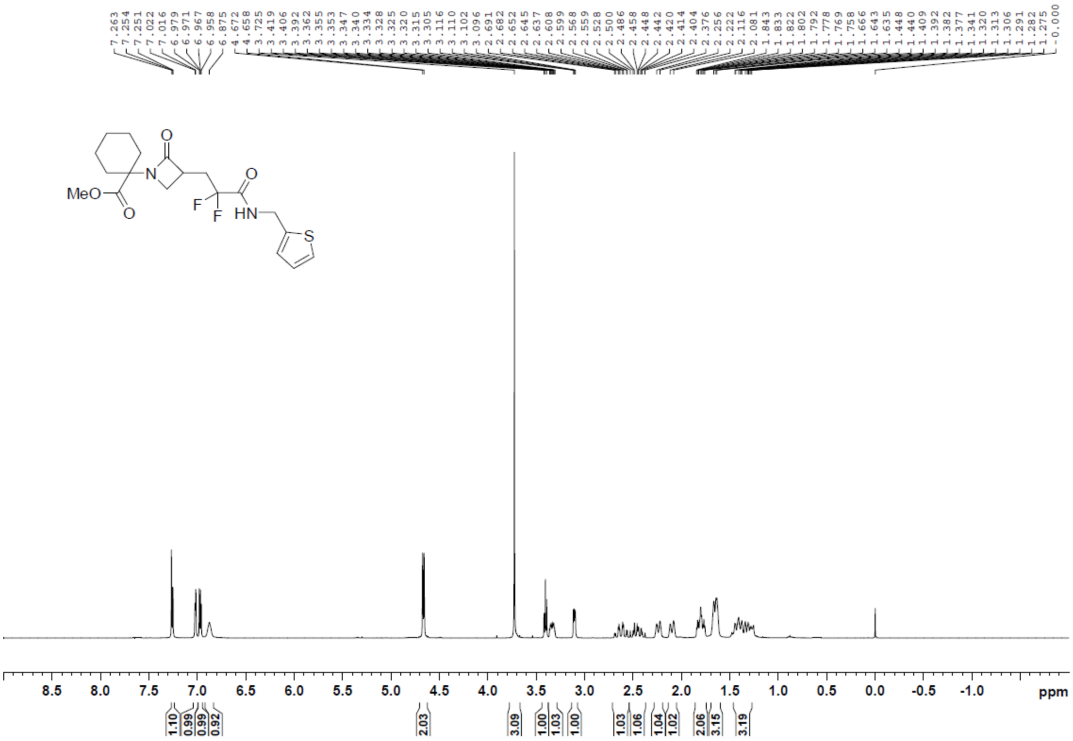

Supplementary Figure 193. ^{1}H NMR spectrum of **5d** in $CDCl_3$ (400 MHz)

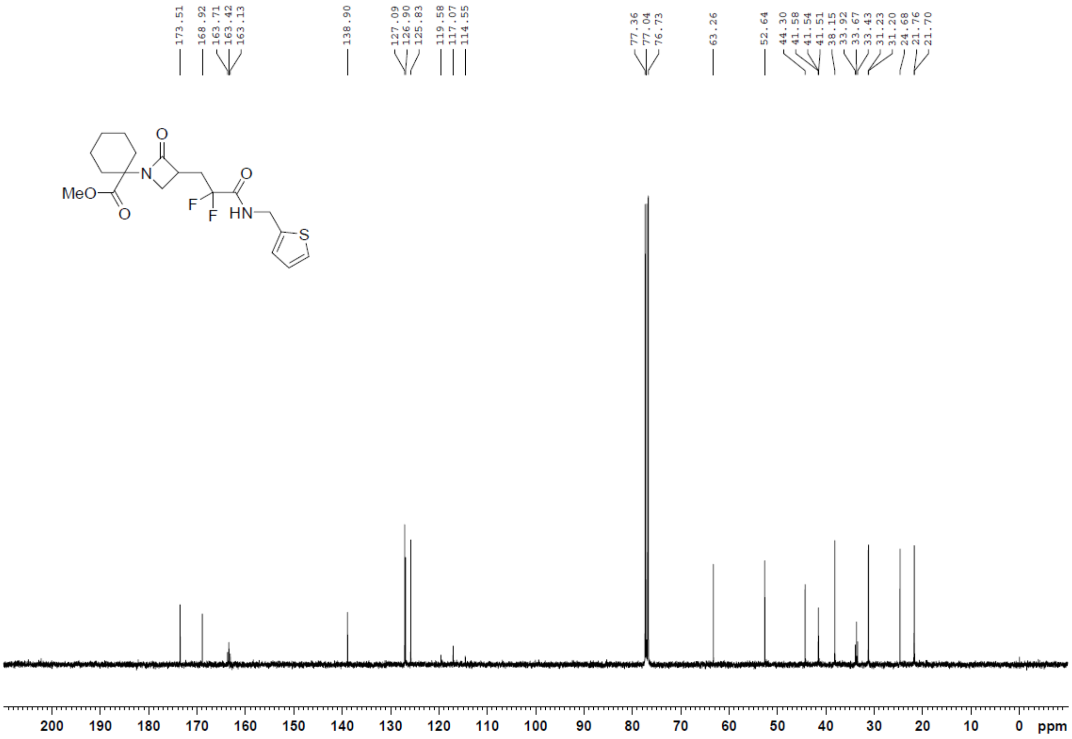

Supplementary Figure 194. ^{13}C NMR spectrum of **5d** in $CDCl_3$ (101 MHz)

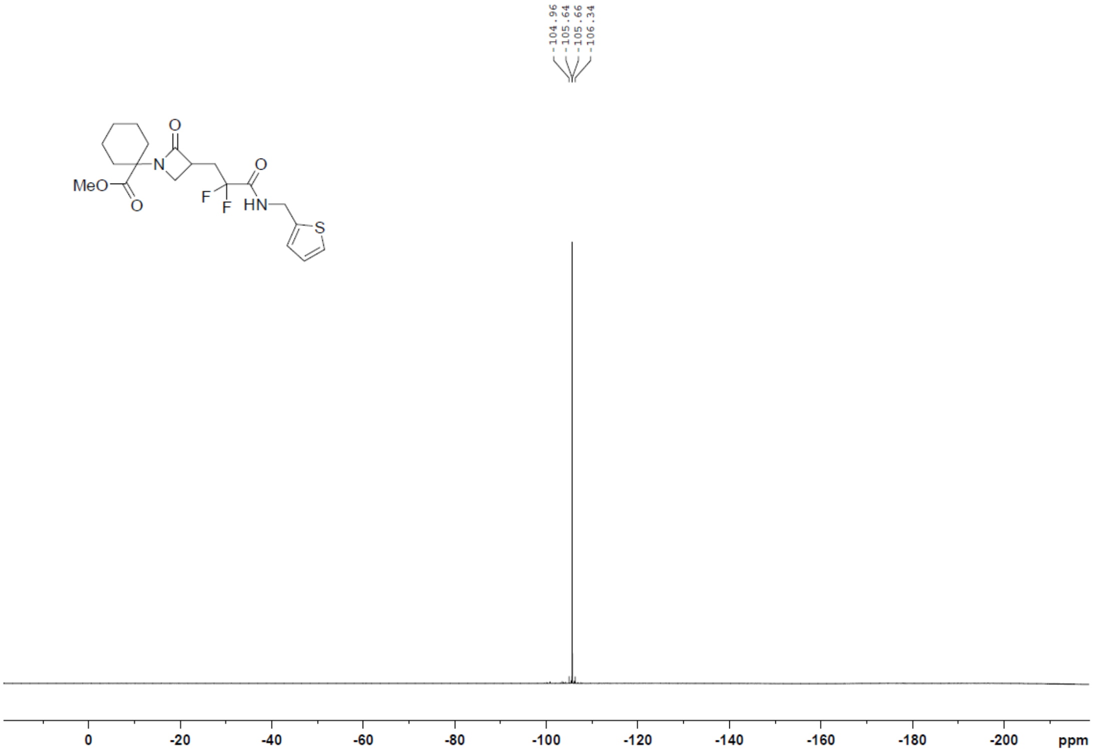

Supplementary Figure 195. ^{19}F NMR spectrum of **5d** in $CDCl_3$ (376 MHz)

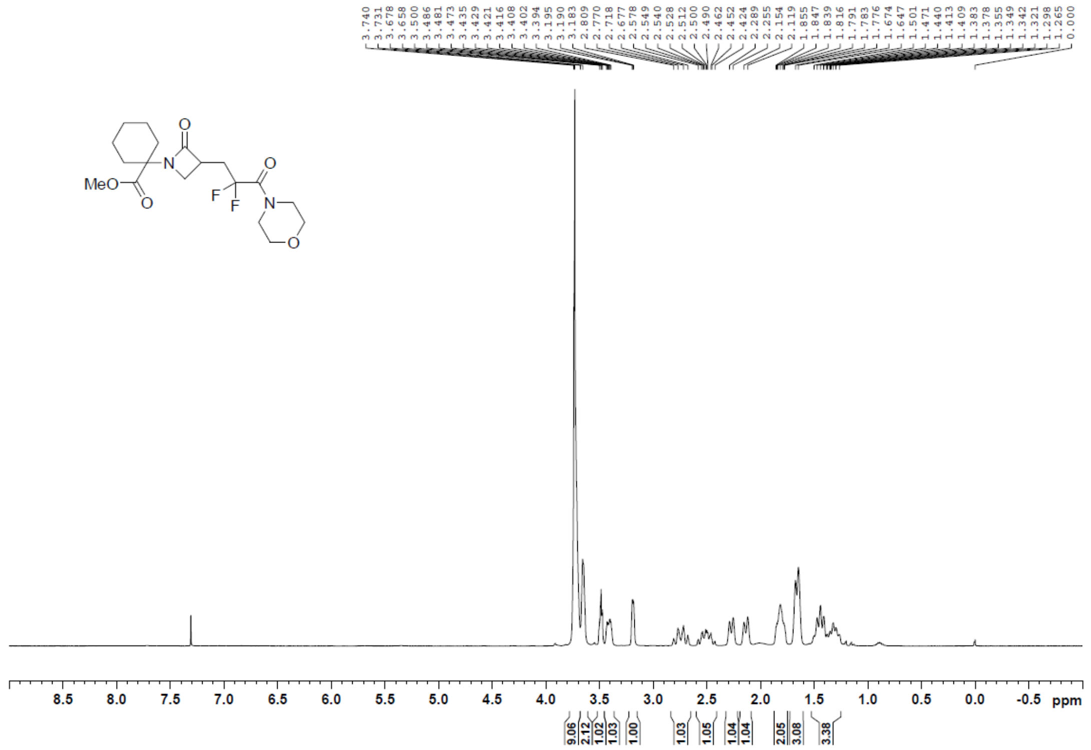

Supplementary Figure 196. ^{1}H NMR spectrum of **5e** in $CDCl_3$ (400 MHz)

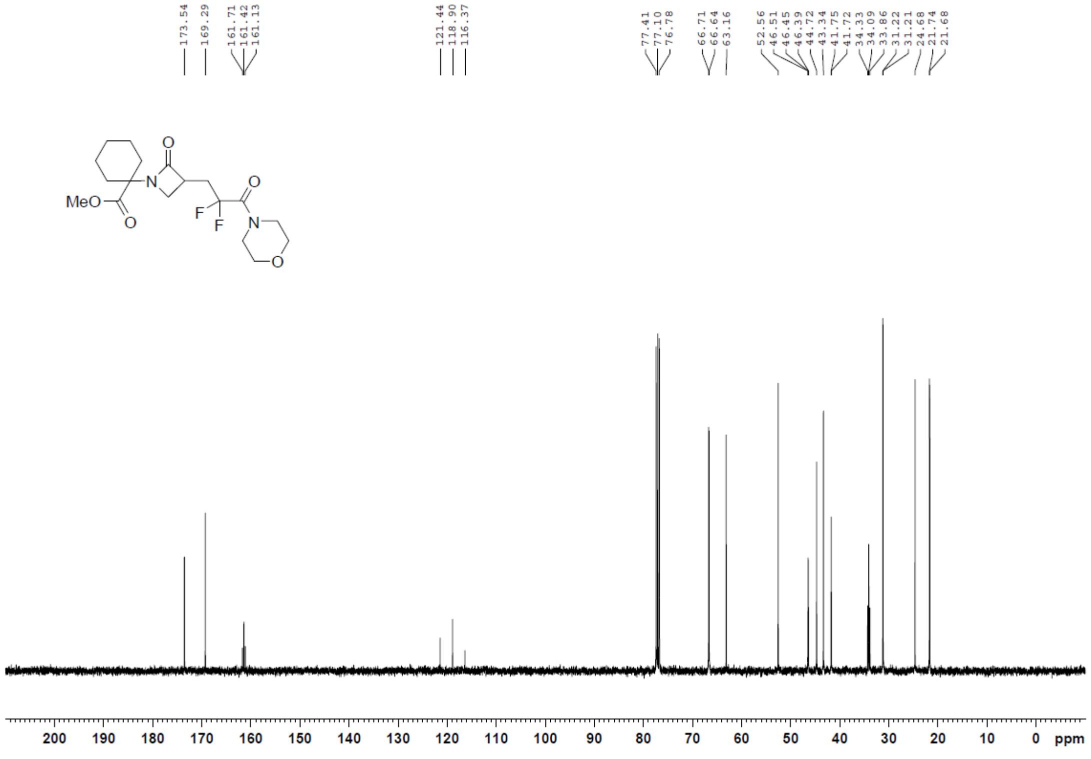

Supplementary Figure 197. ^{13}C NMR spectrum of **5e** in $CDCl_3$ (101 MHz)

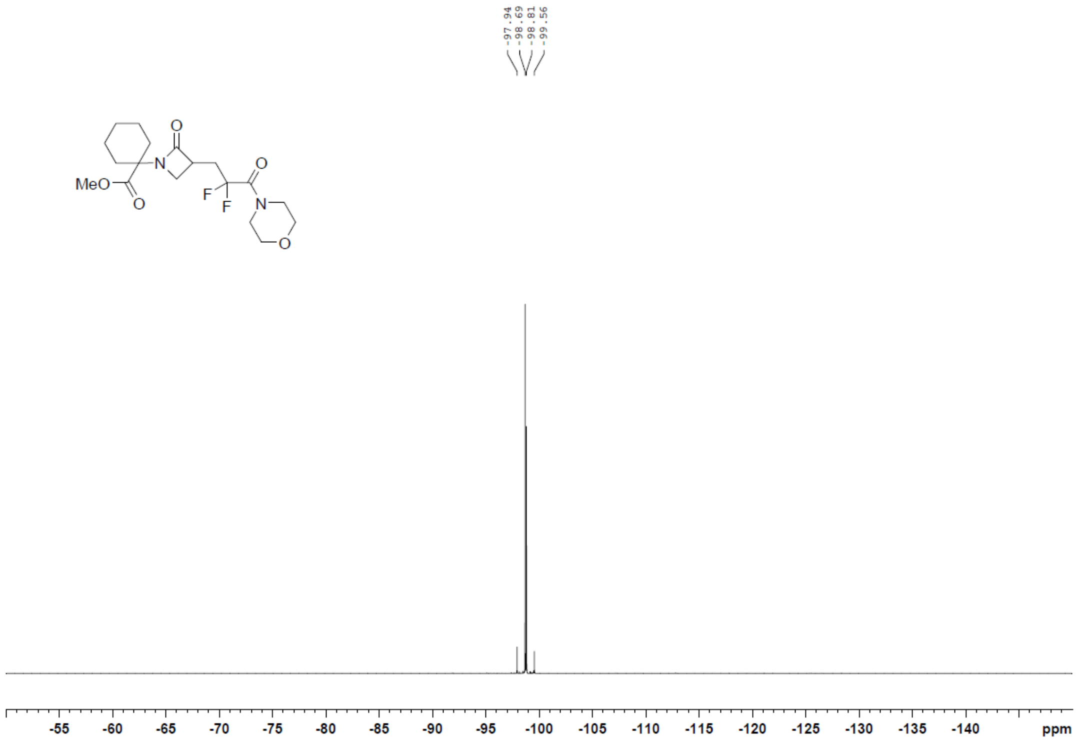

Supplementary Figure 198. ^{19}F NMR spectrum of **5e** in $CDCl_3$ (376 MHz)

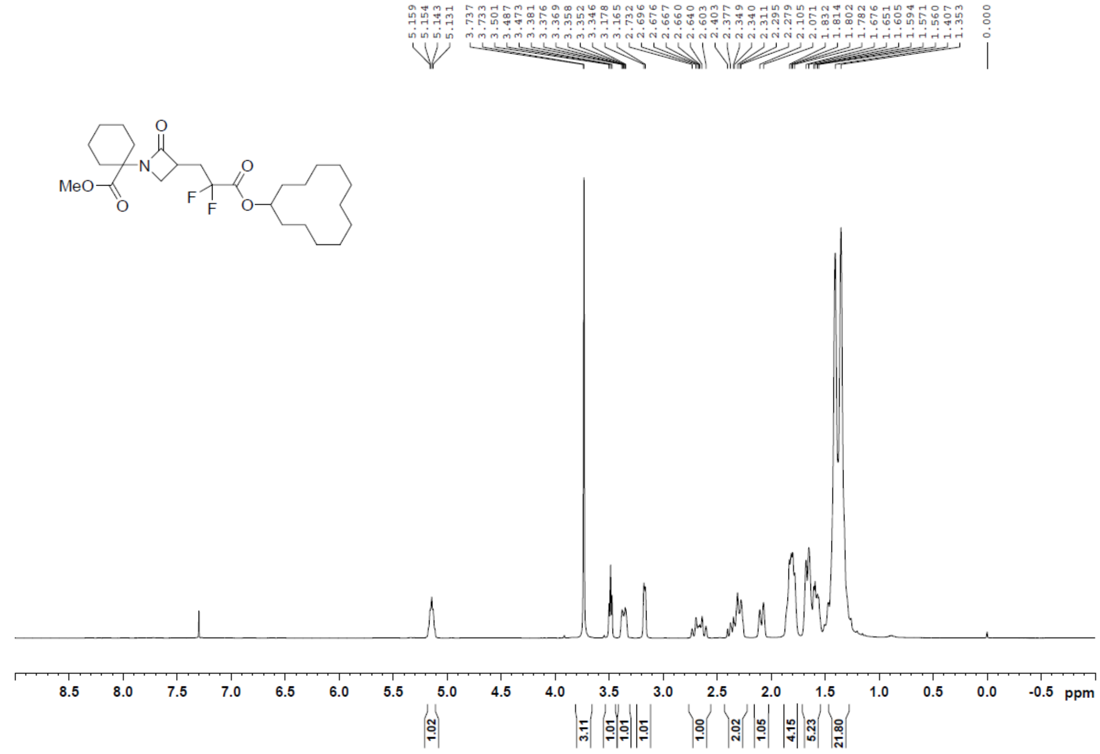

Supplementary Figure 199. ^{1}H NMR spectrum of **5f** in $CDCl_3$ (400 MHz)

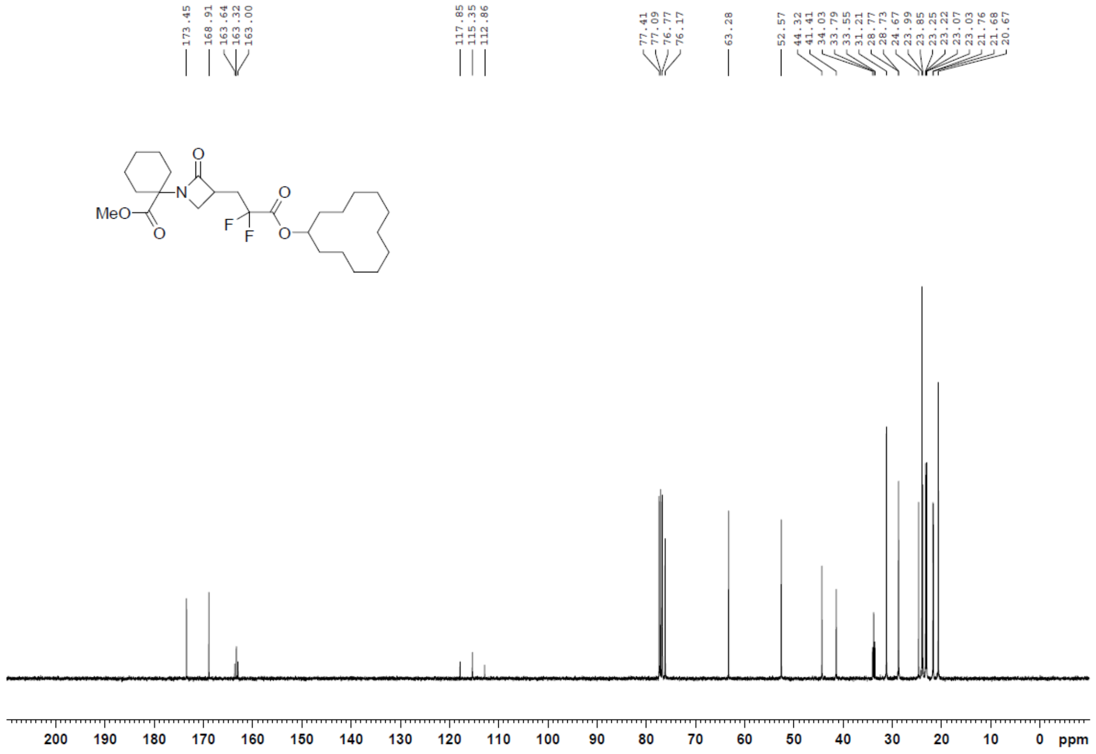

Supplementary Figure 200. ^{13}C NMR spectrum of **5f** in $CDCl_3$ (101 MHz)

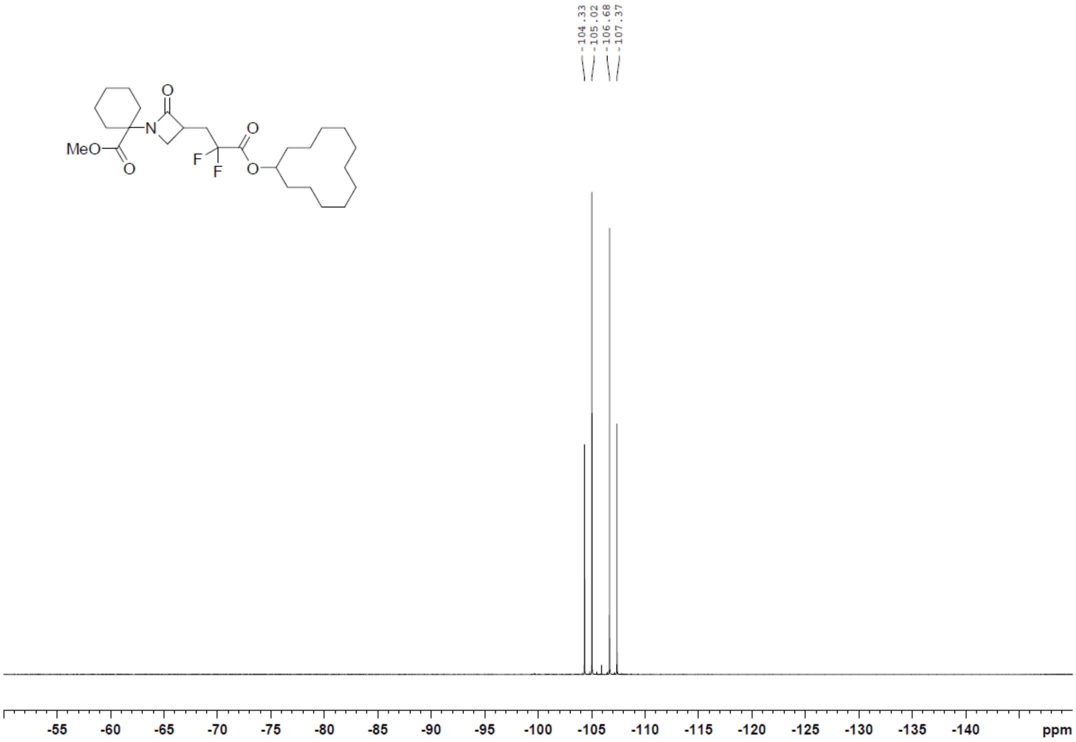

Supplementary Figure 201. ^{19}F NMR spectrum of **5f** in $CDCl_3$ (376 MHz)

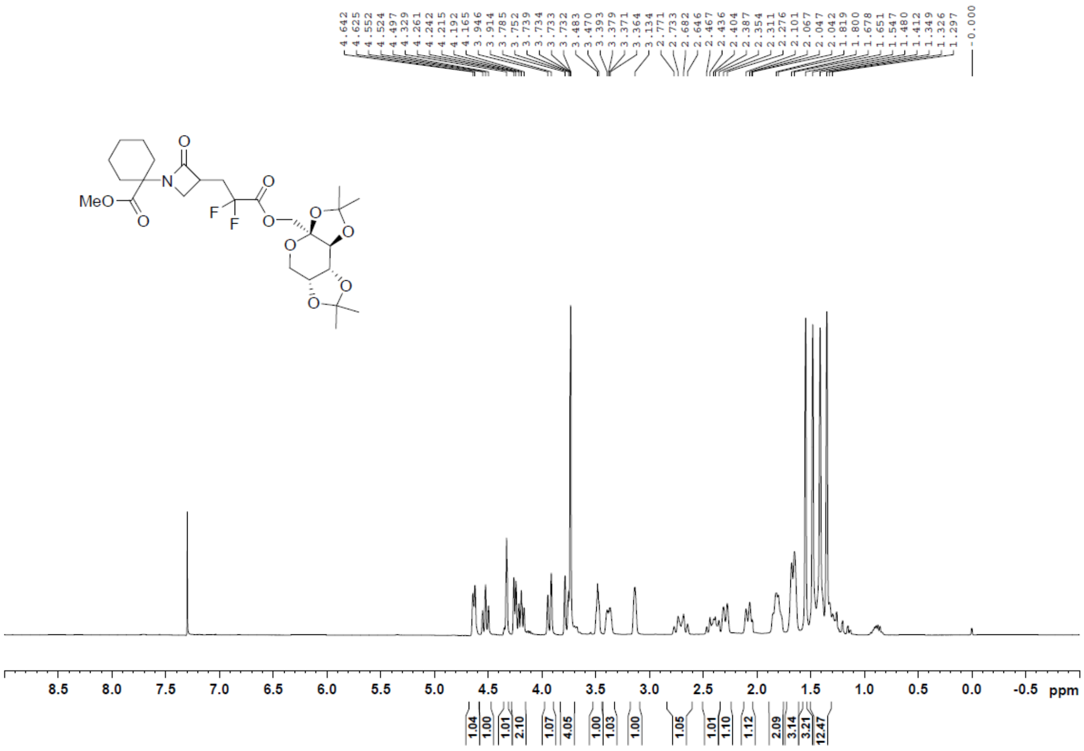

Supplementary Figure 202. ^{1}H NMR spectrum of **5g** in $CDCl_3$ (400 MHz)

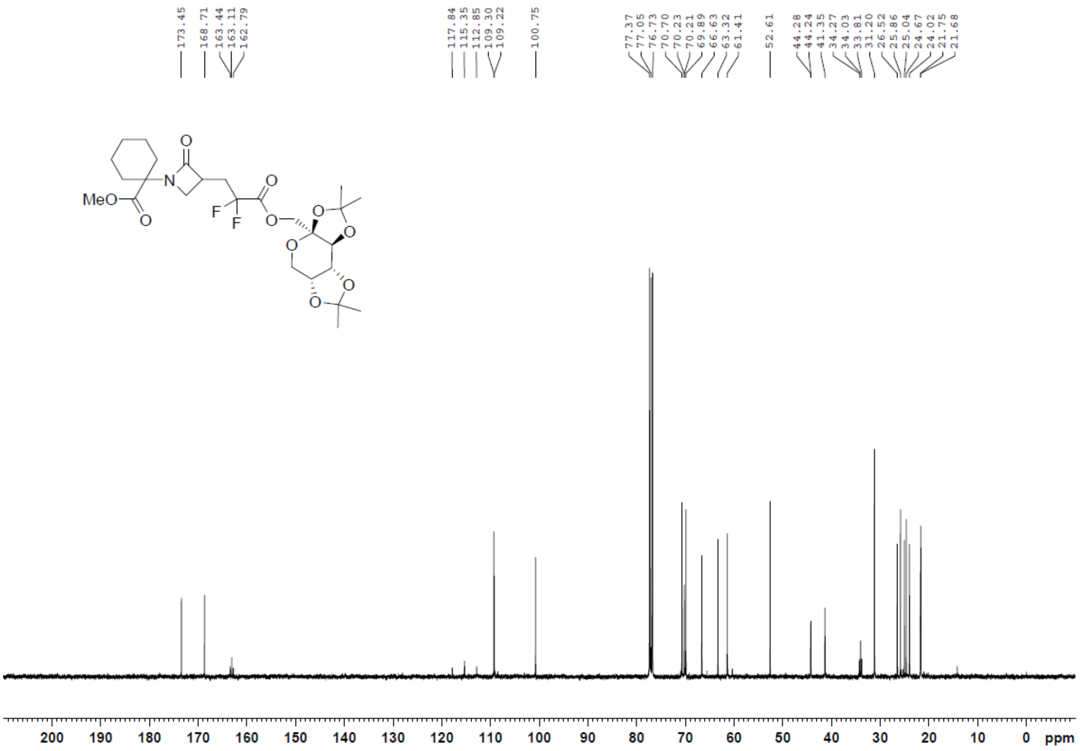

Supplementary Figure 203. ^{13}C NMR spectrum of **5g** in $CDCl_3$ (101 MHz)

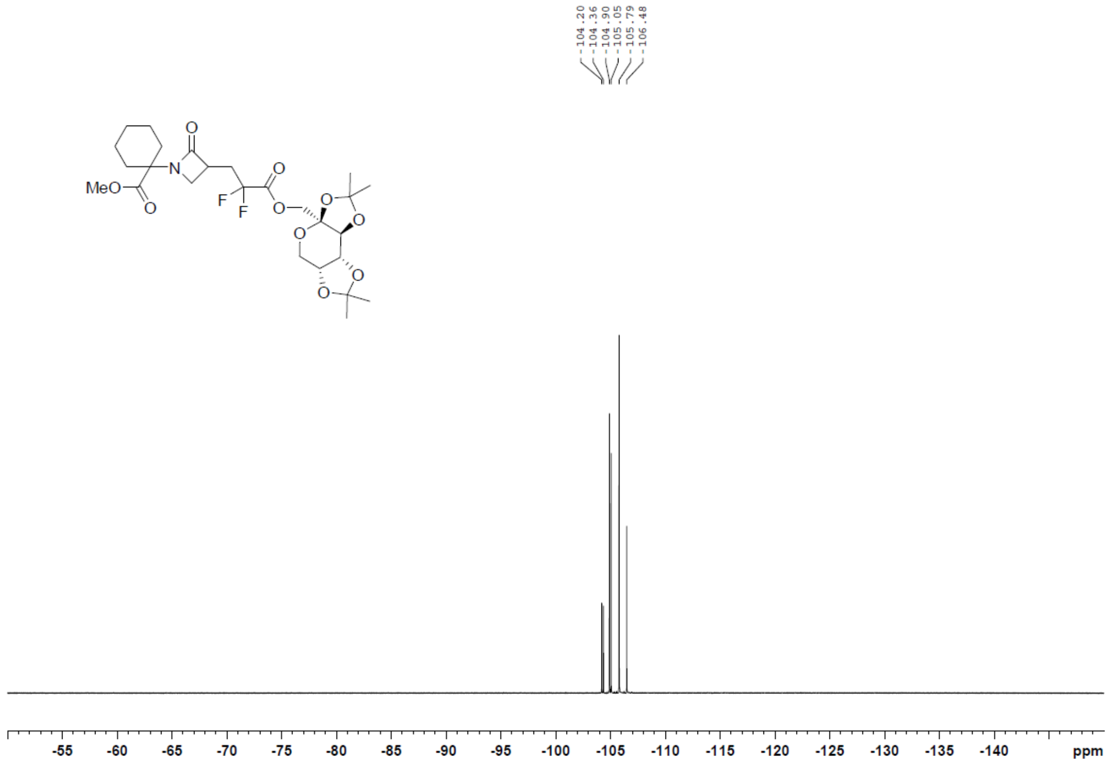

Supplementary Figure 204. ^{19}F NMR spectrum of **5g** in $CDCl_3$ (376 MHz)

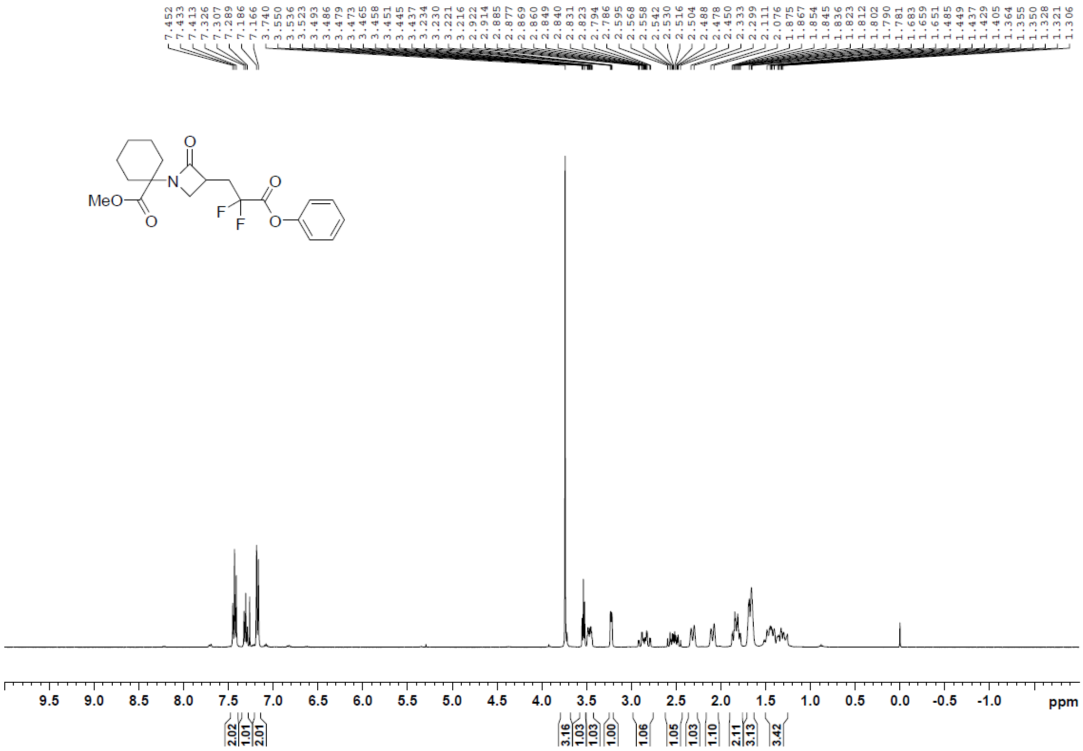

Supplementary Figure 205. ^{1}H NMR spectrum of **5h** in $CDCl_3$ (400 MHz)

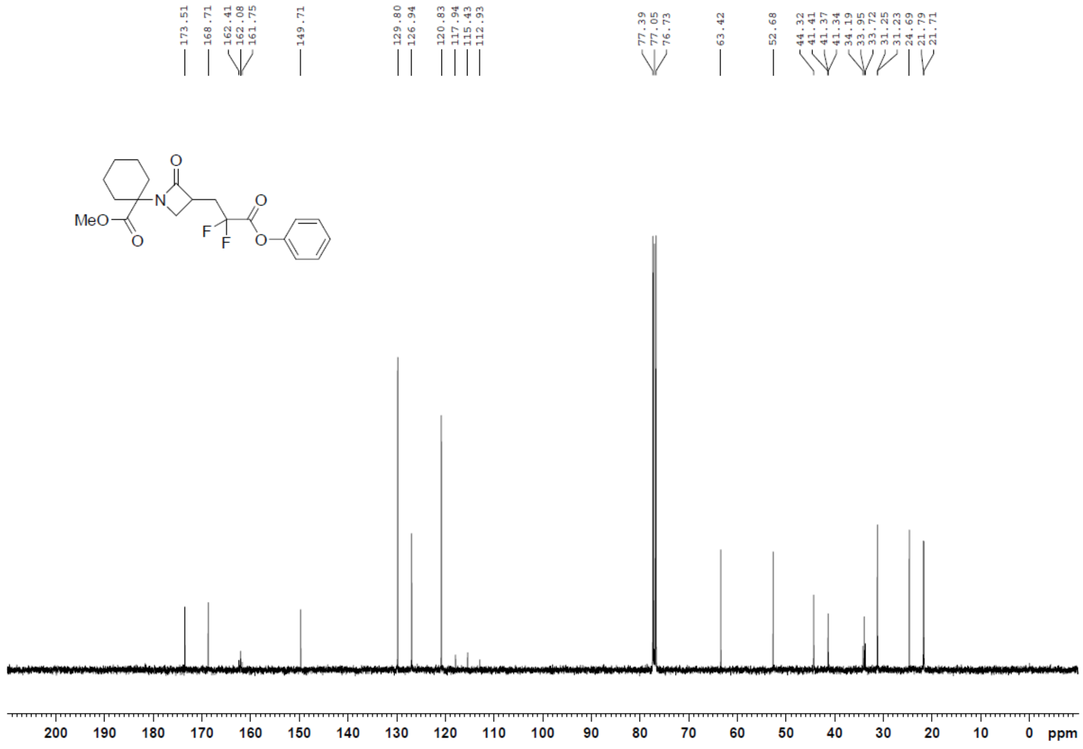

Supplementary Figure 206. ^{13}C NMR spectrum of **5h** in $CDCl_3$ (101 MHz)

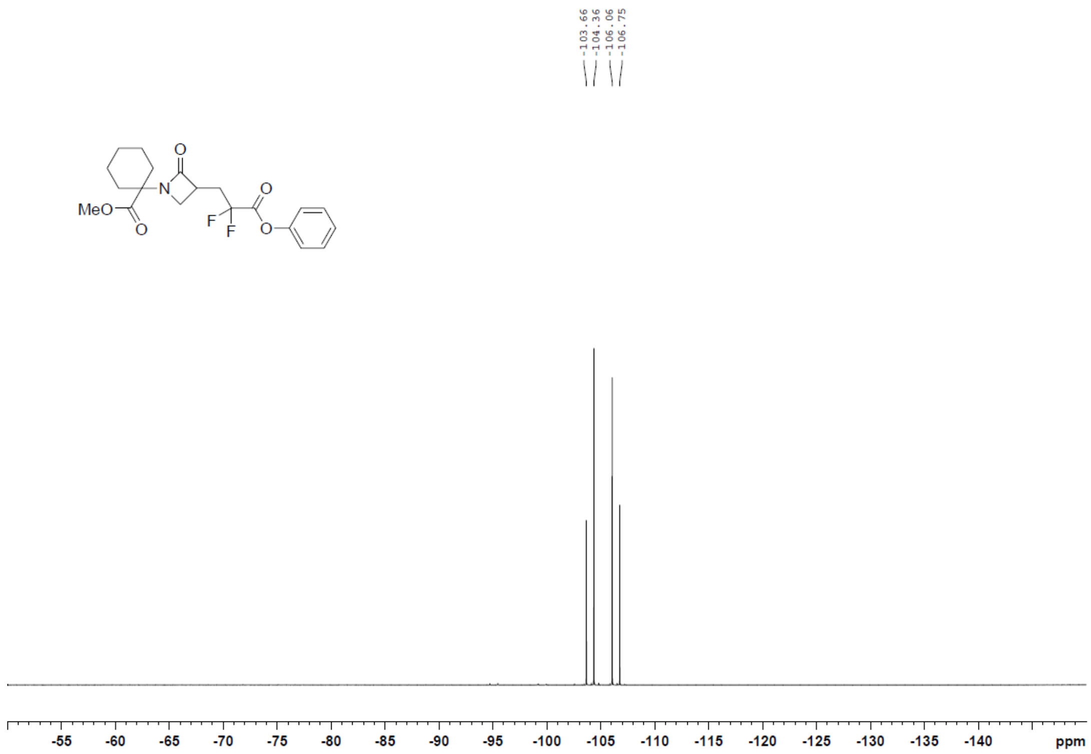

Supplementary Figure 207. ^{19}F NMR spectrum of **5h** in $CDCl_3$ (376 MHz)

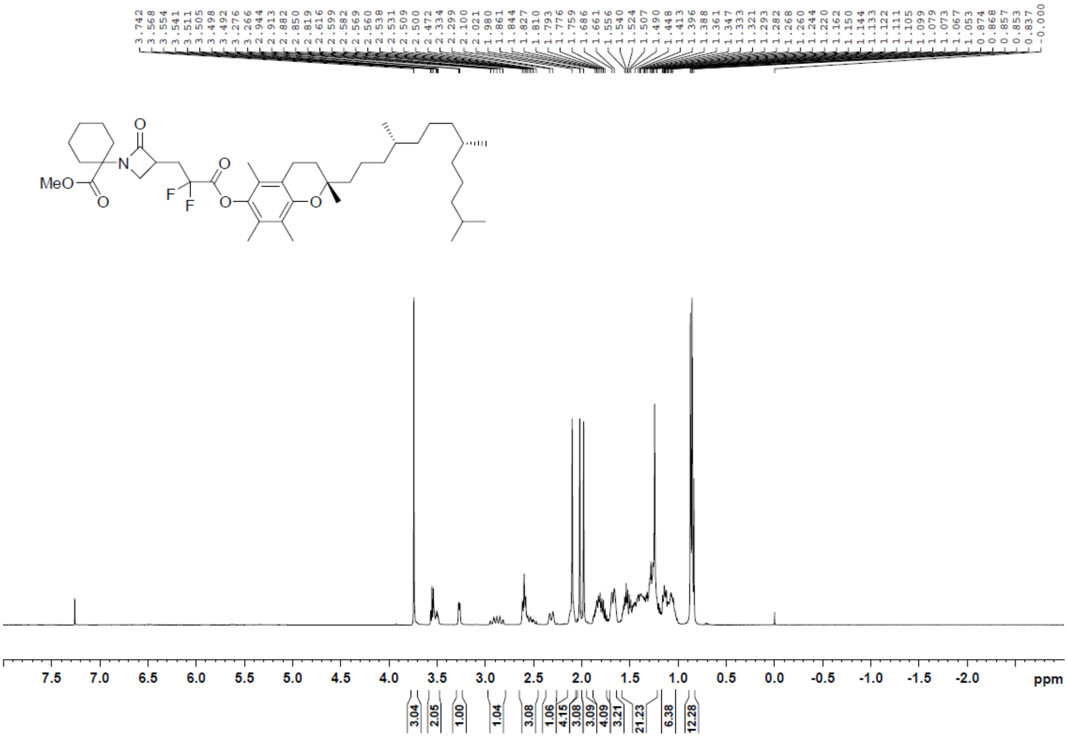

Supplementary Figure 208. ^{1}H NMR spectrum of **5i** in $CDCl_3$ (400 MHz)

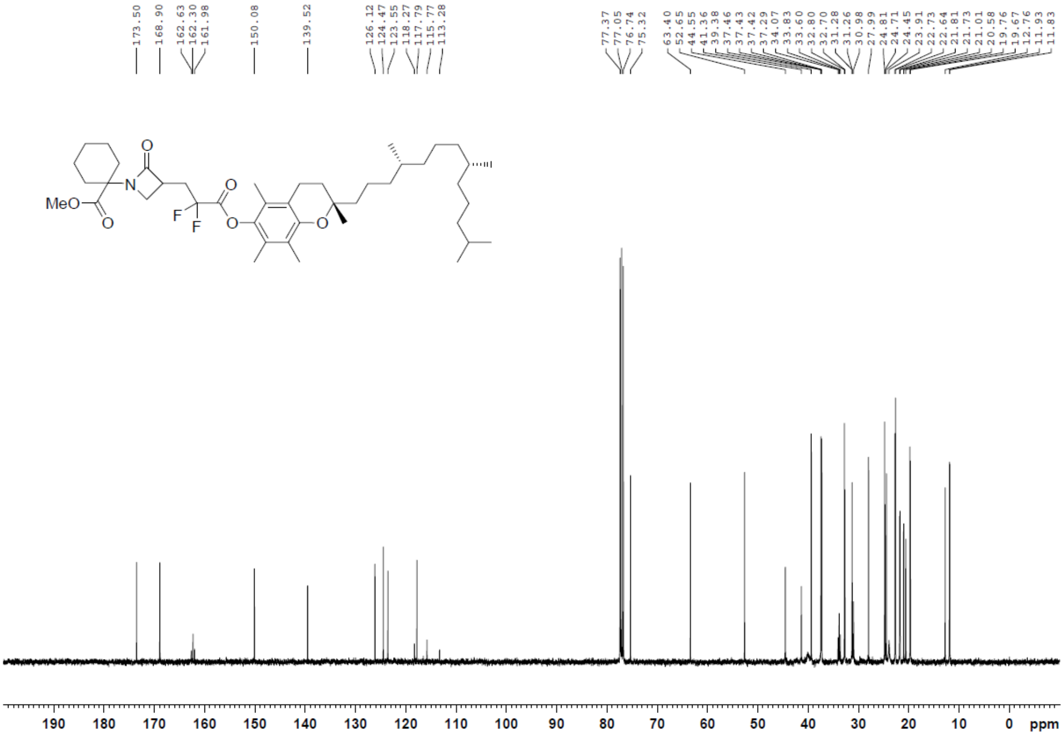

Supplementary Figure 209. ^{13}C NMR spectrum of **5i** in $CDCl_3$ (101 MHz)

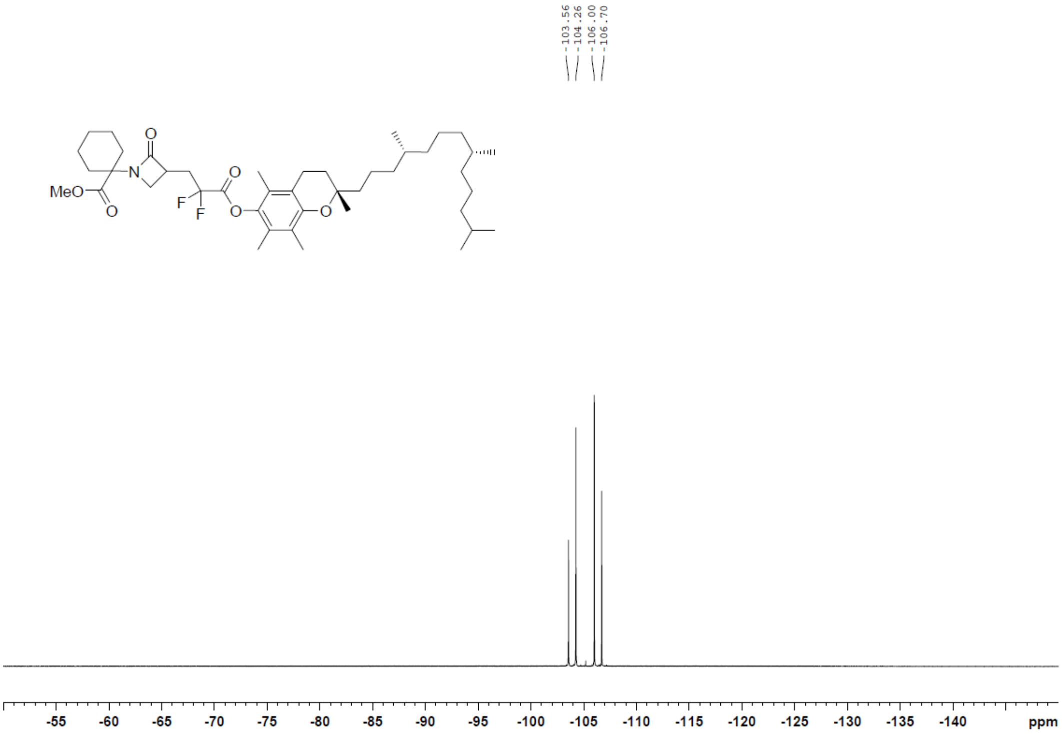

Supplementary Figure 210. ^{19}F NMR spectrum of **5i** in $CDCl_3$ (376 MHz)

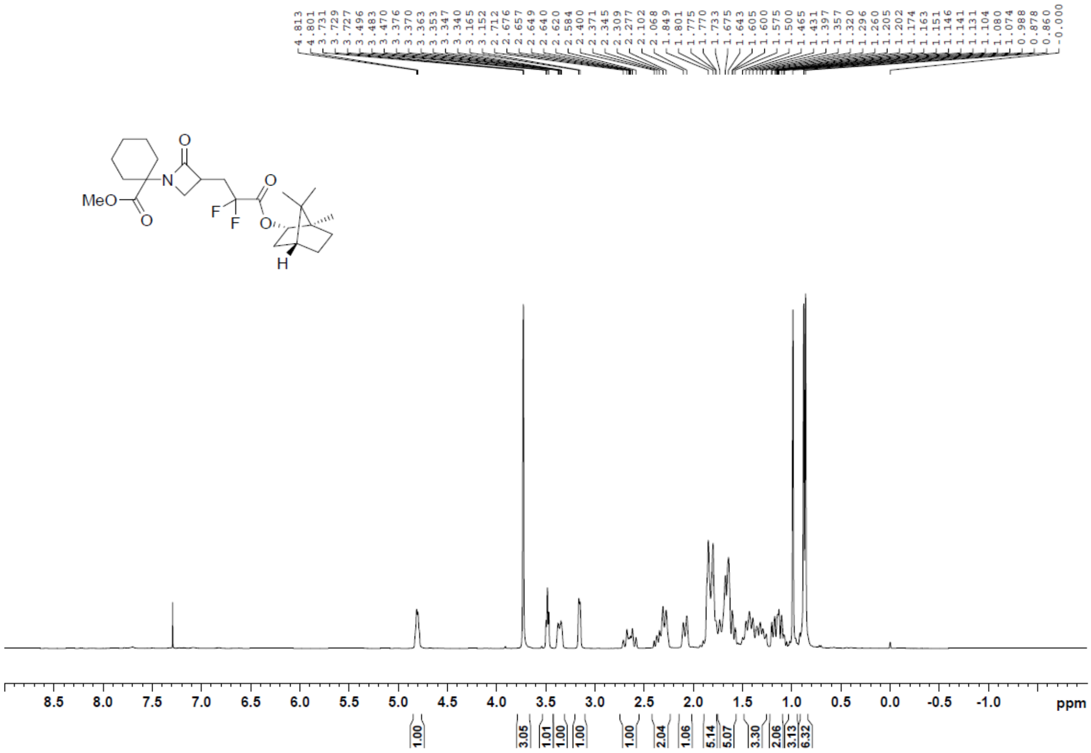

Supplementary Figure 211. ^{1}H NMR spectrum of **5j** in $CDCl_3$ (400 MHz)

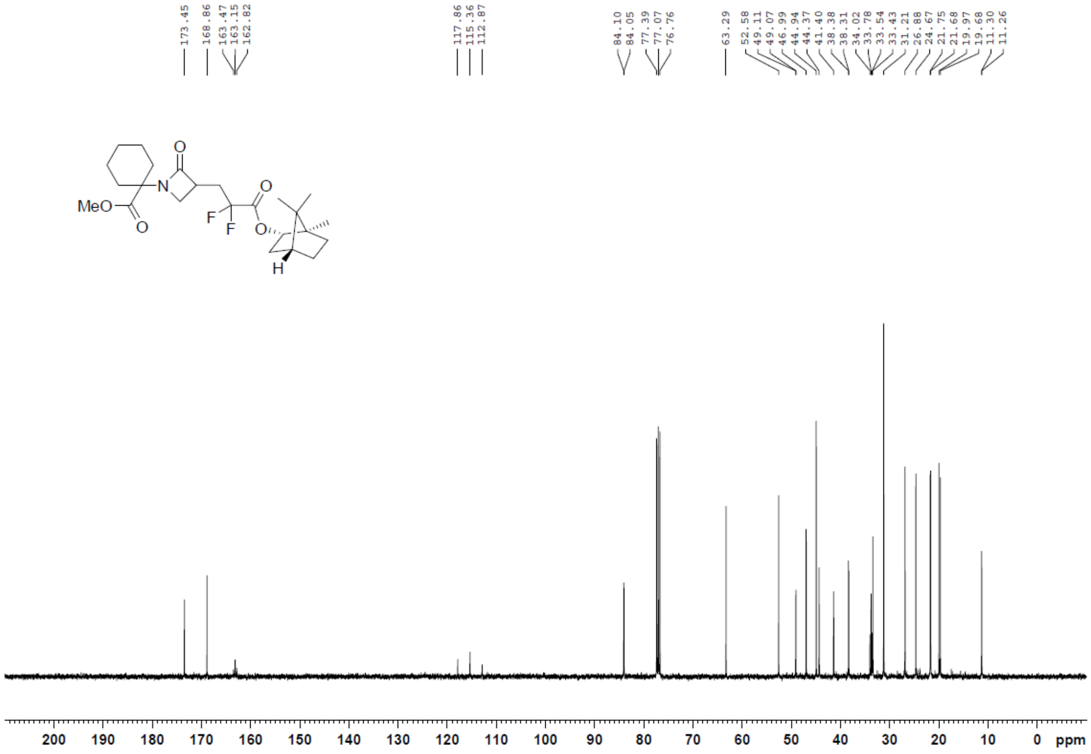

Supplementary Figure 212. ^{13}C NMR spectrum of **5j** in $CDCl_3$ (101 MHz)

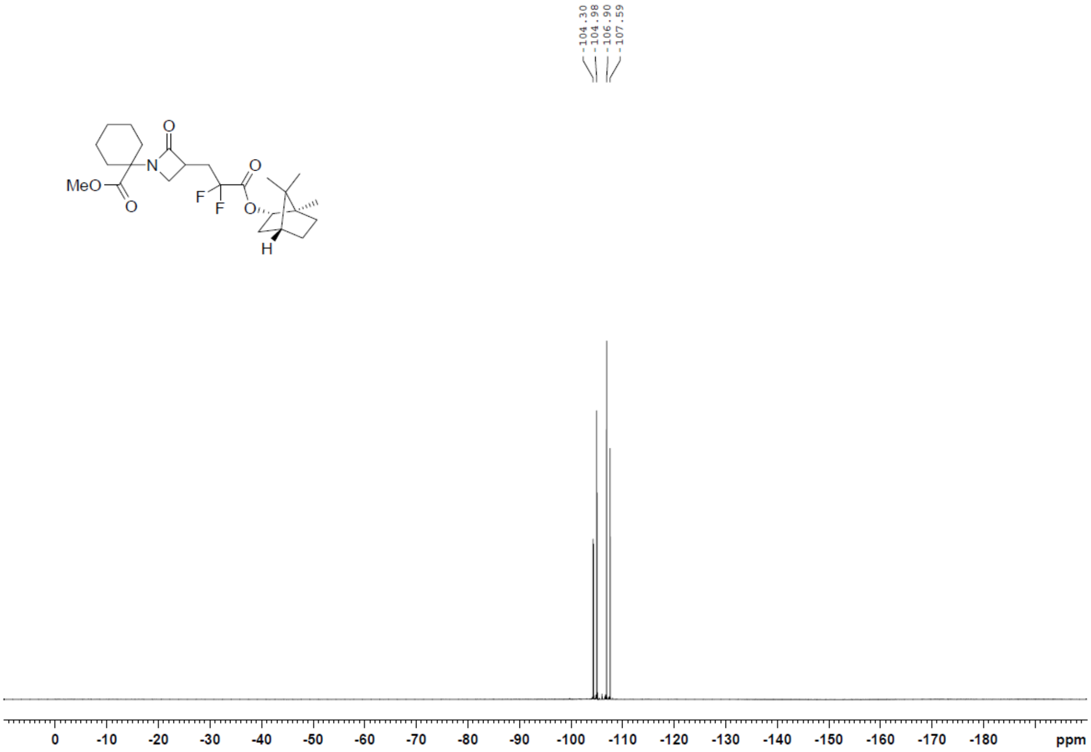

Supplementary Figure 213. ^{19}F NMR spectrum of **5j** in $CDCl_3$ (376 MHz)

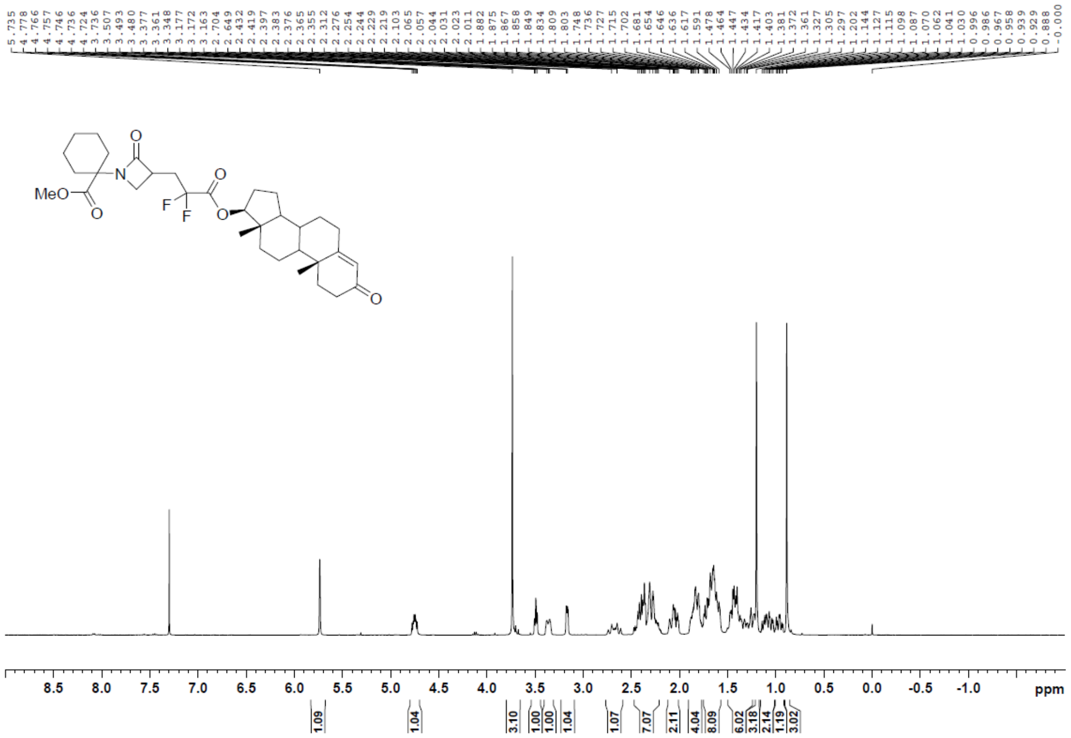

Supplementary Figure 214. ^{1}H NMR spectrum of **5k** in $CDCl_3$ (400 MHz)

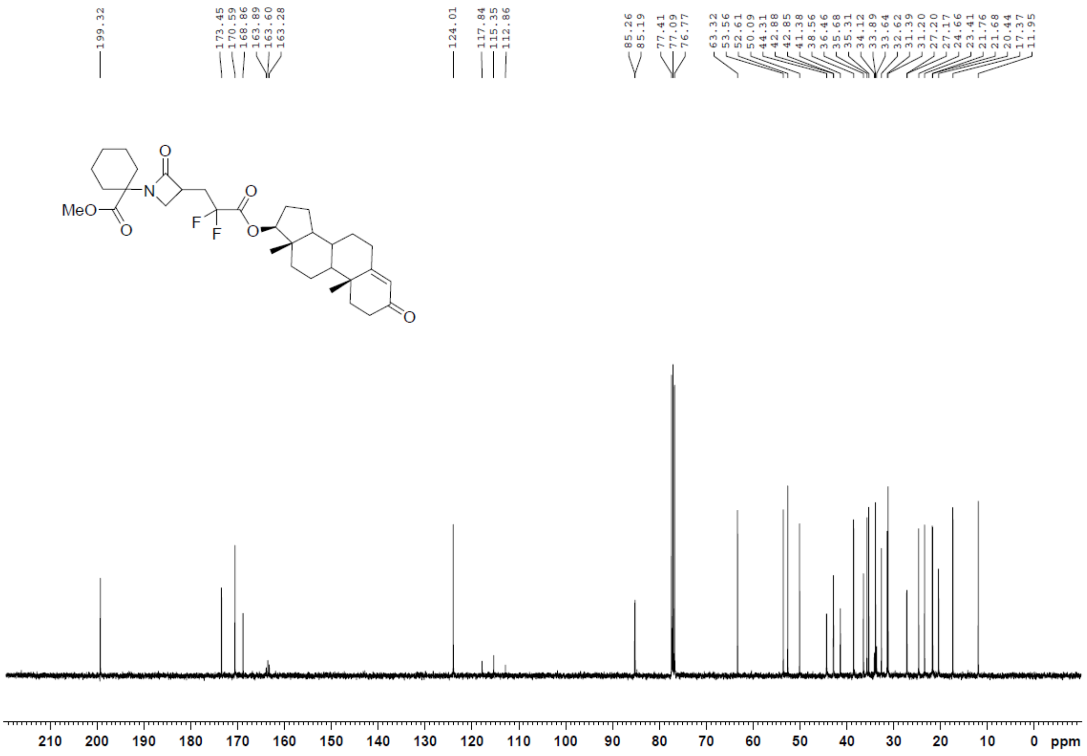

Supplementary Figure 215. ^{13}C NMR spectrum of **5k** in $CDCl_3$ (101 MHz)

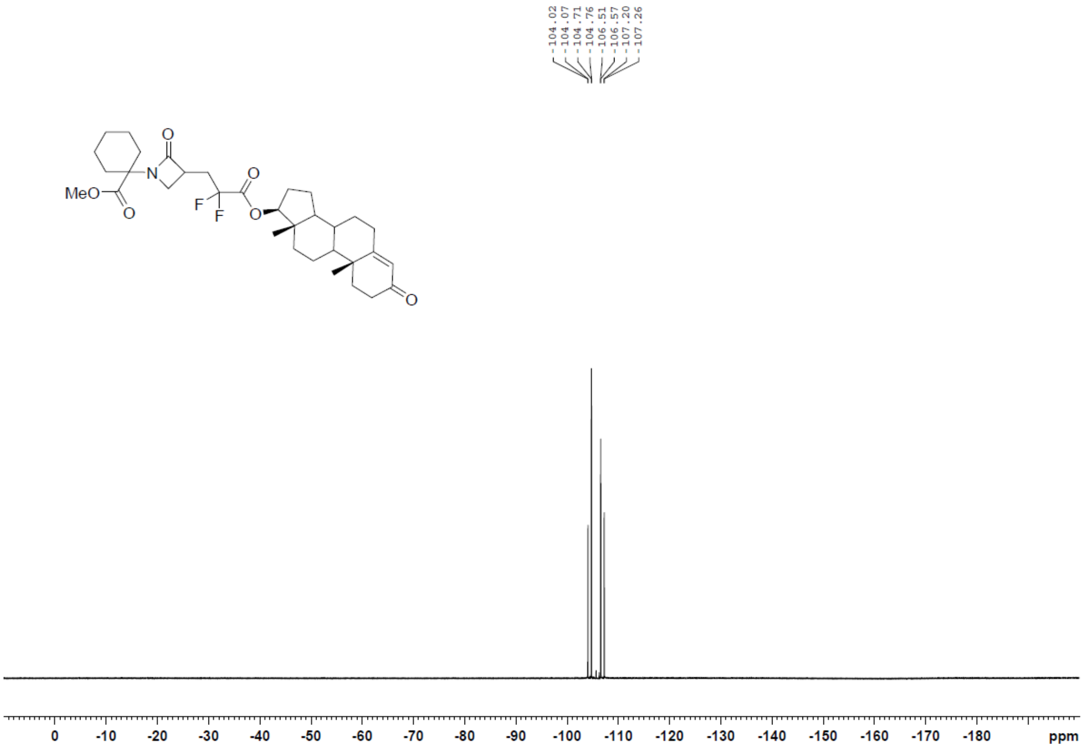

Supplementary Figure 216. ^{19}F NMR spectrum of **5k** in $CDCl_3$ (376 MHz)

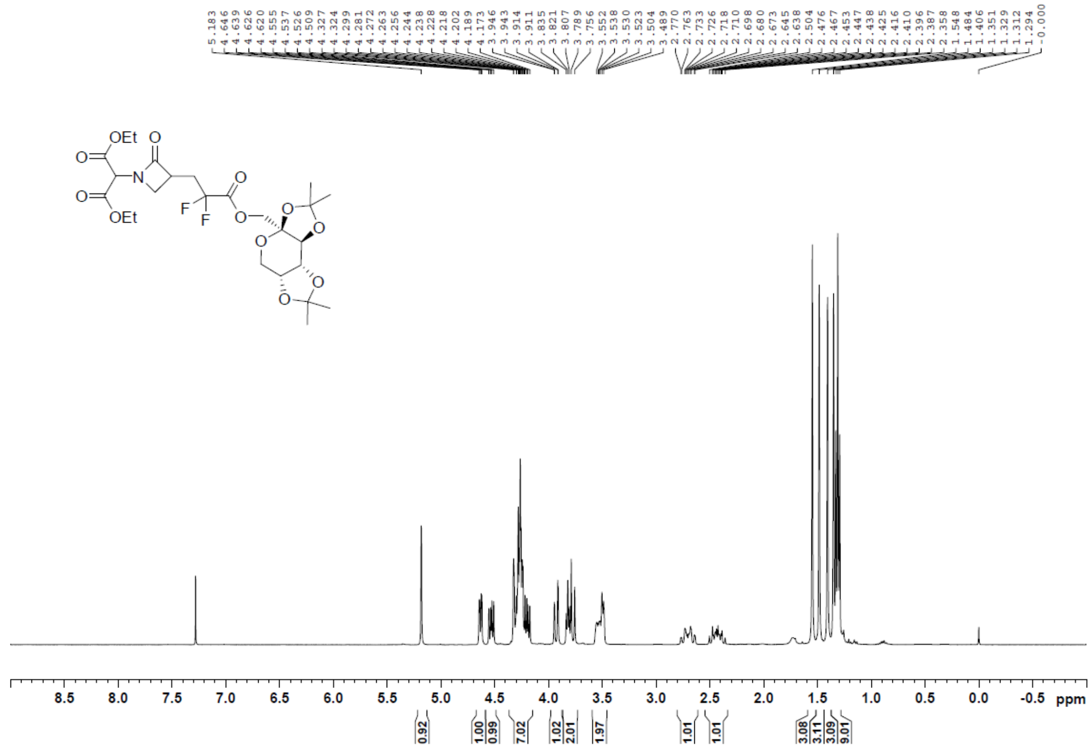

Supplementary Figure 217. ^{1}H NMR spectrum of **5l** in $CDCl_3$ (400 MHz)

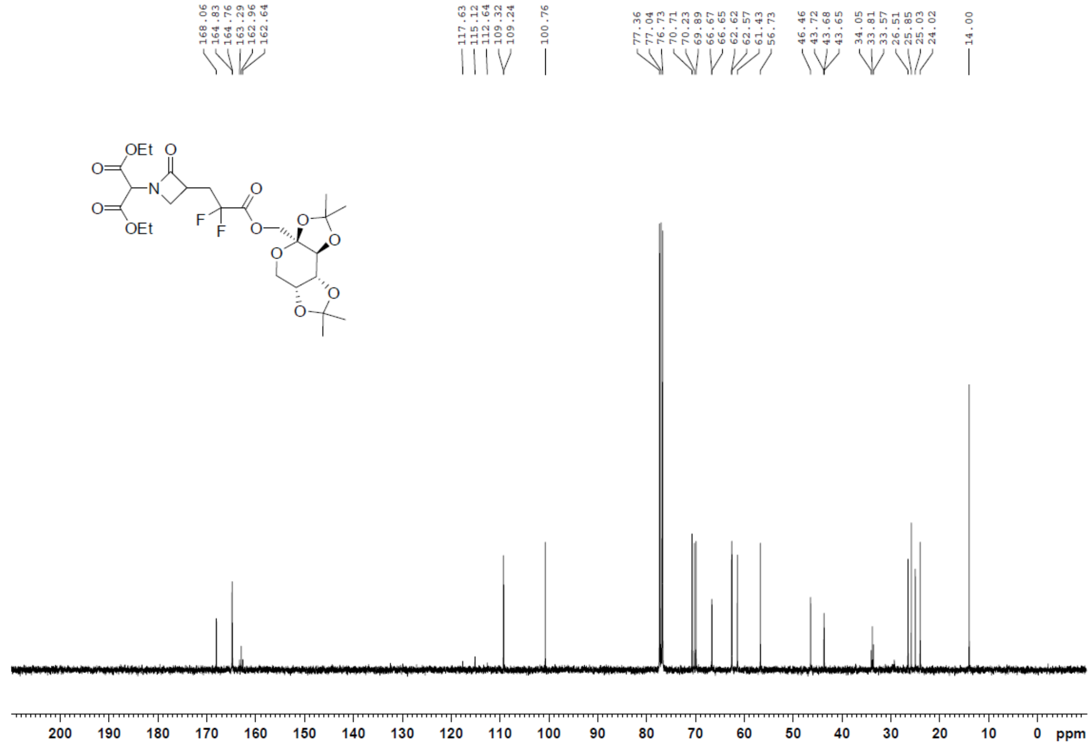

Supplementary Figure 218. ^{13}C NMR spectrum of **5l** in $CDCl_3$ (101 MHz)

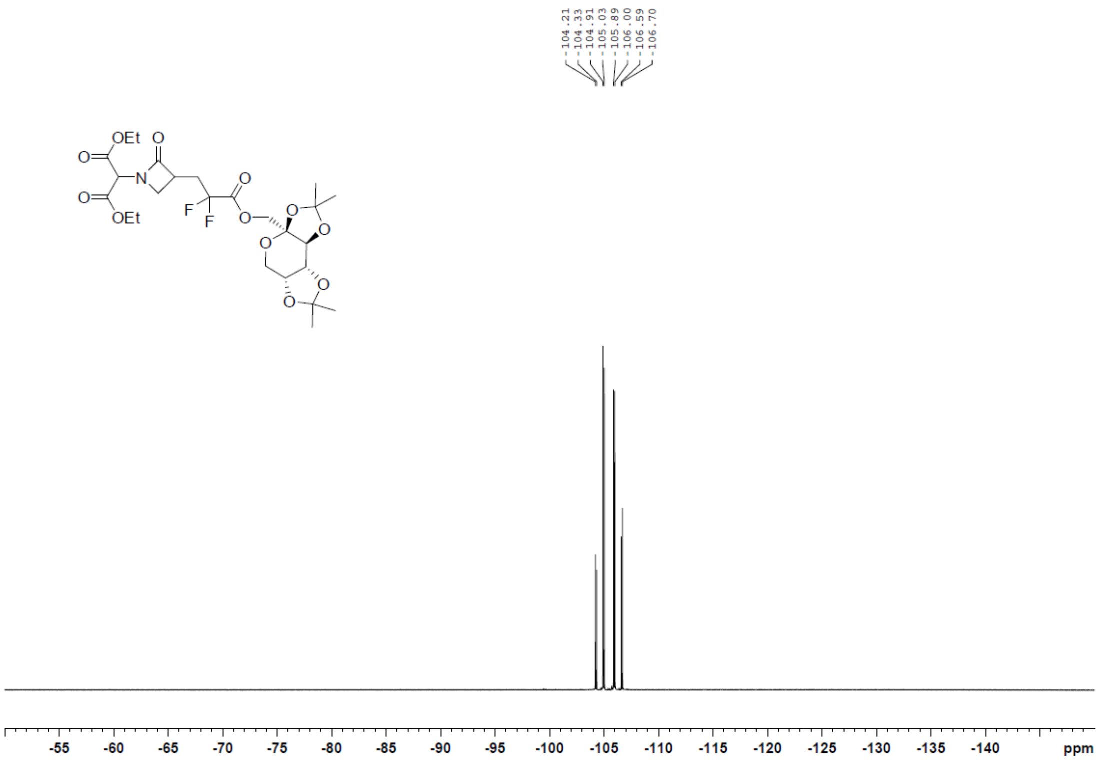

Supplementary Figure 219. ^{19}F NMR spectrum of **5l** in $CDCl_3$ (376 MHz)

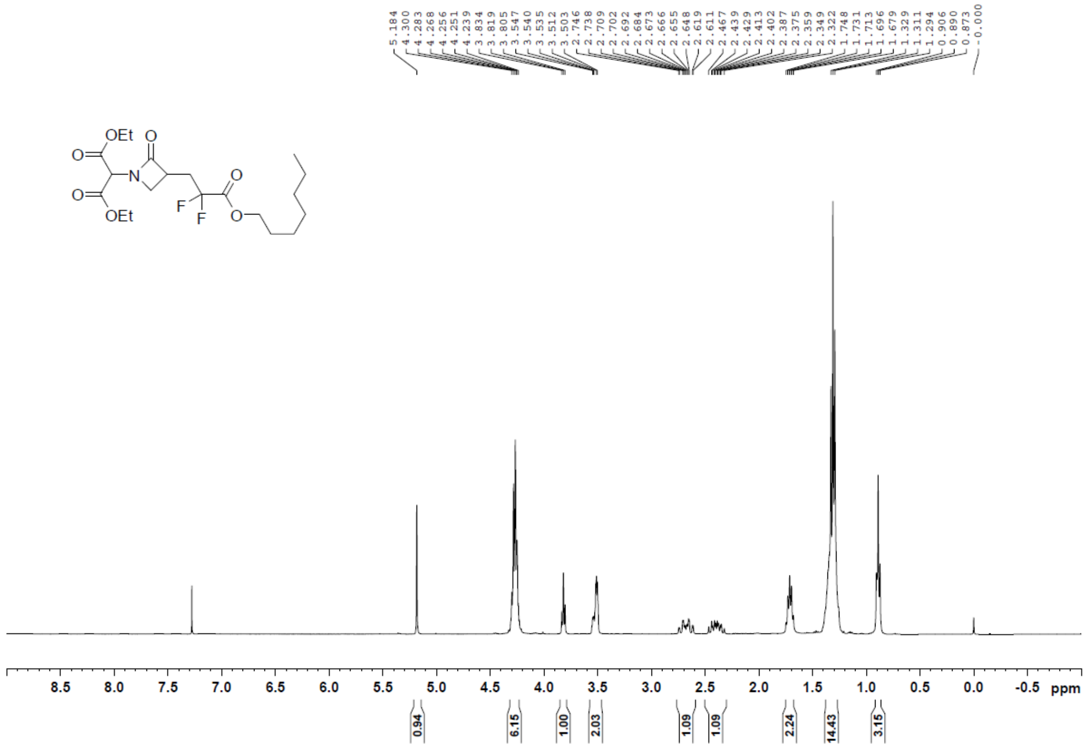

Supplementary Figure 220. ^{1}H NMR spectrum of **5m** in $CDCl_3$ (400 MHz)

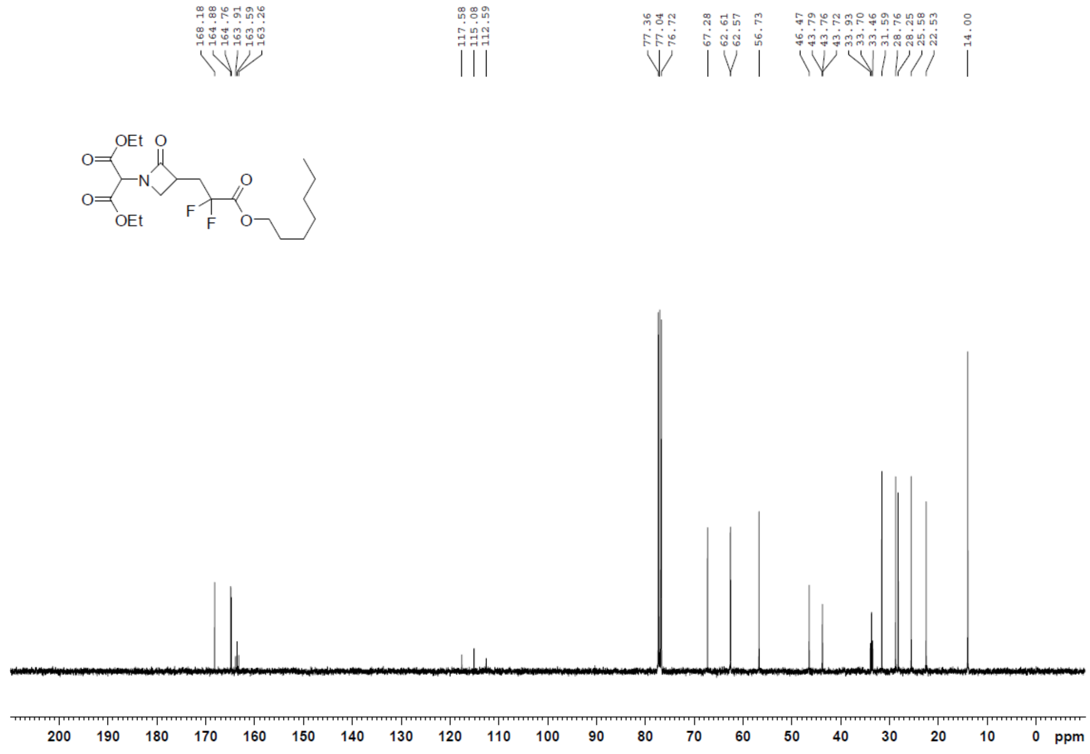

Supplementary Figure 221. ^{13}C NMR spectrum of **5m** in $CDCl_3$ (101 MHz)

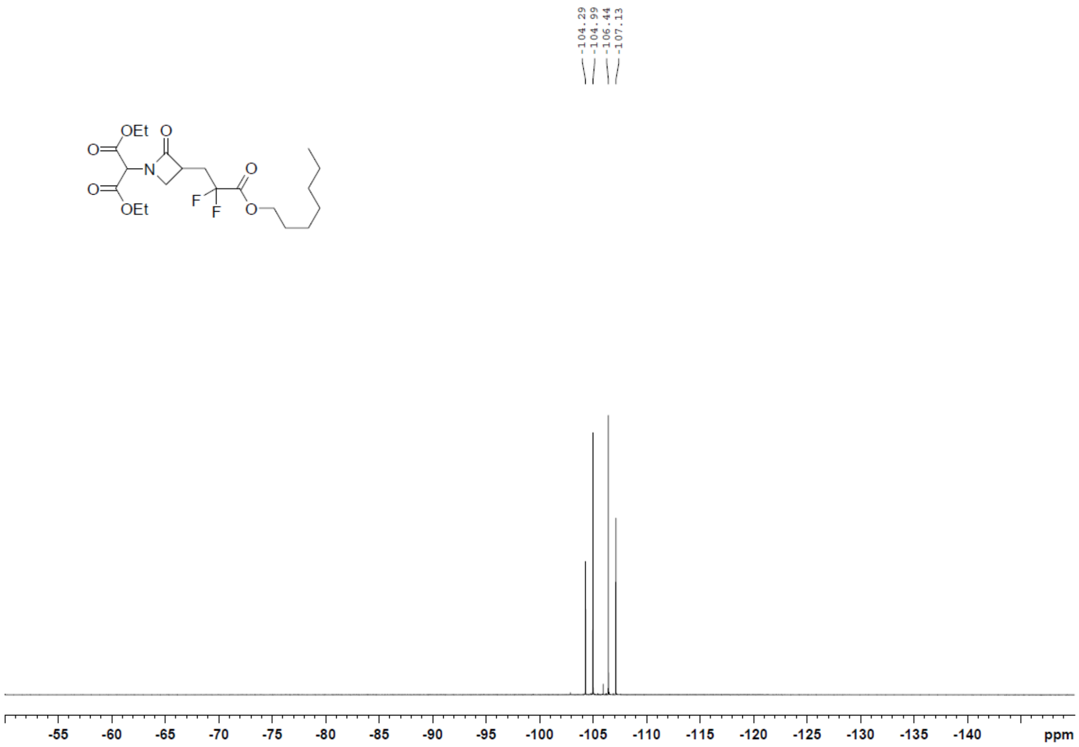

Supplementary Figure 222. ^{19}F NMR spectrum of **5m** in $CDCl_3$ (376 MHz)

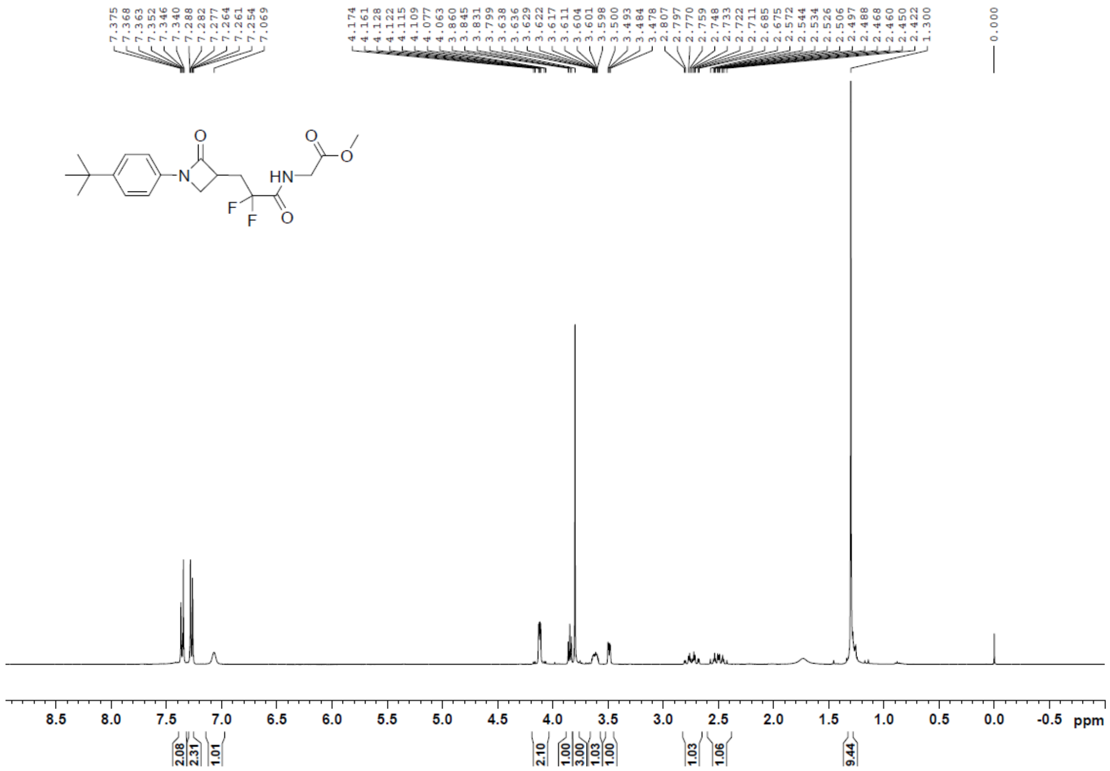

Supplementary Figure 223. ^{1}H NMR spectrum of **5n** in $CDCl_3$ (400 MHz)

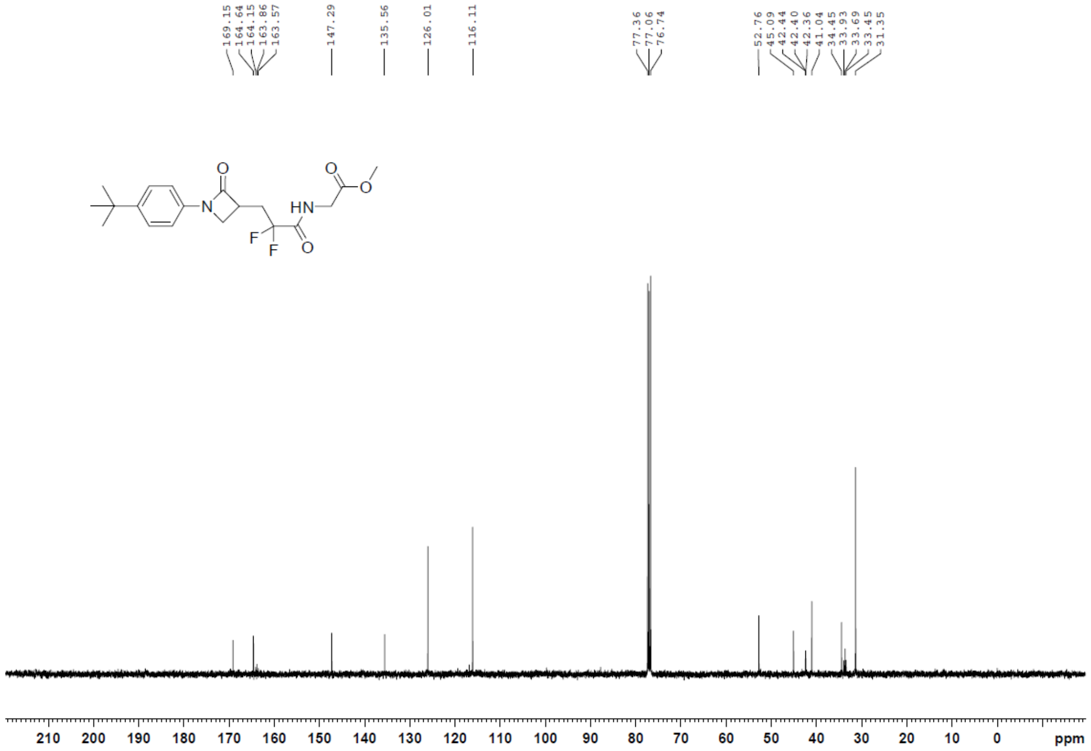

Supplementary Figure 224. ^{13}C NMR spectrum of **5n** in $CDCl_3$ (101 MHz)

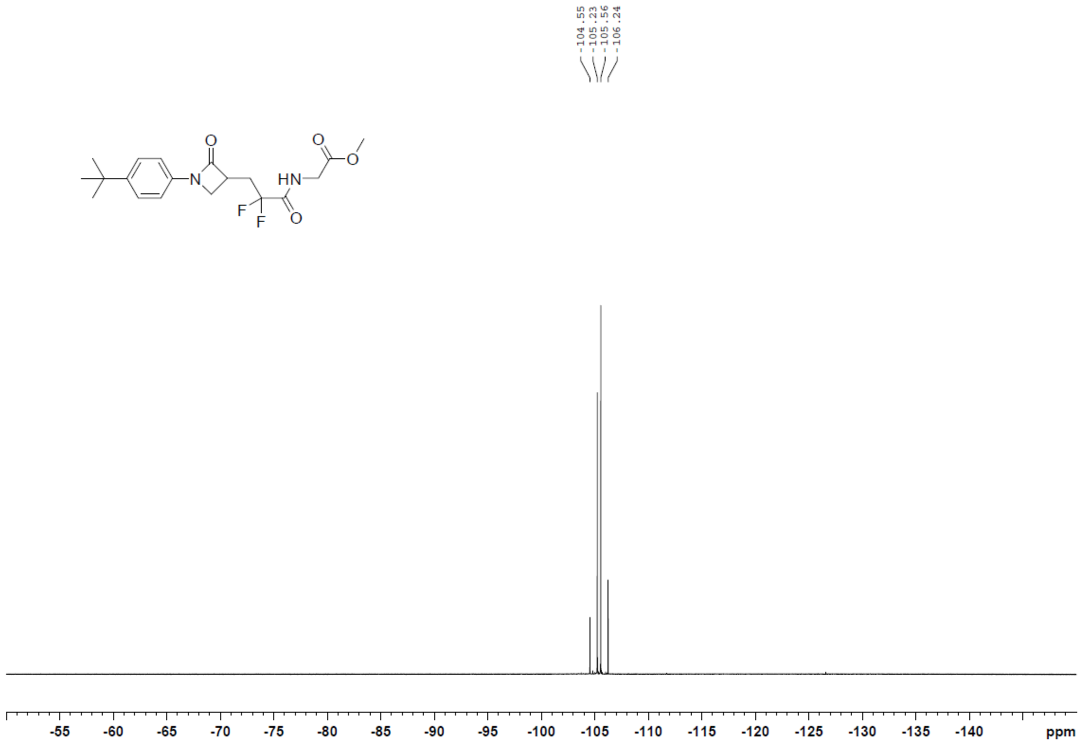

Supplementary Figure 225. ^{19}F NMR spectrum of **5n** in $CDCl_3$ (376 MHz)

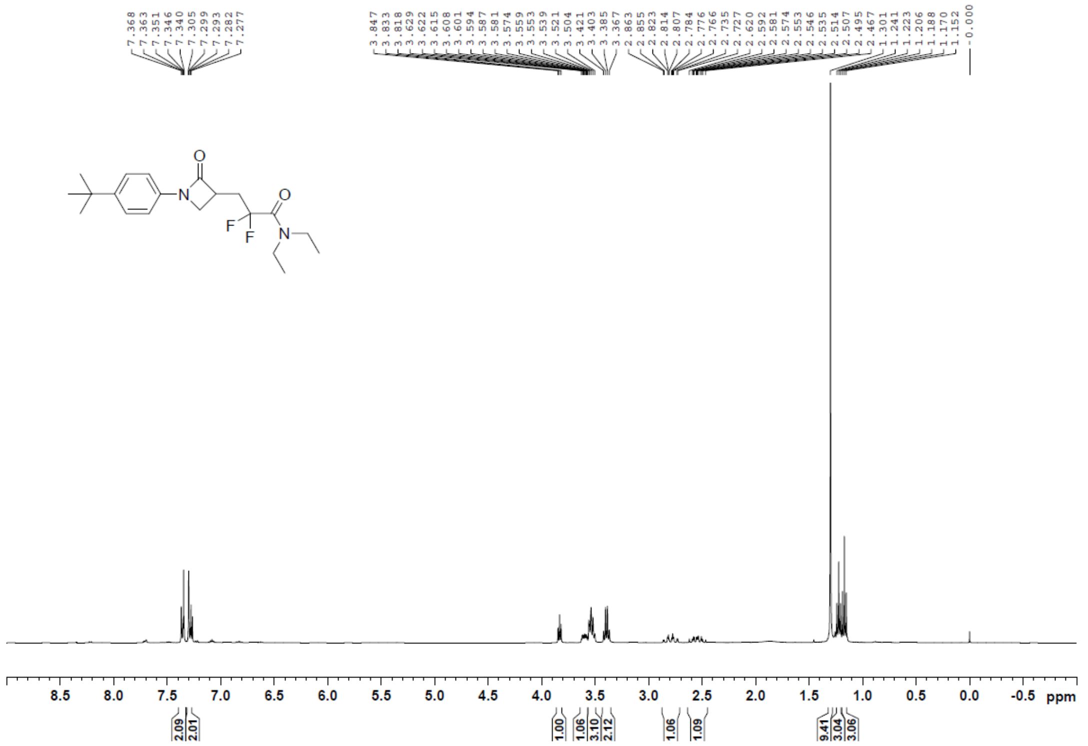

Supplementary Figure 226. ^{1}H NMR spectrum of **5o** in $CDCl_3$ (400 MHz)

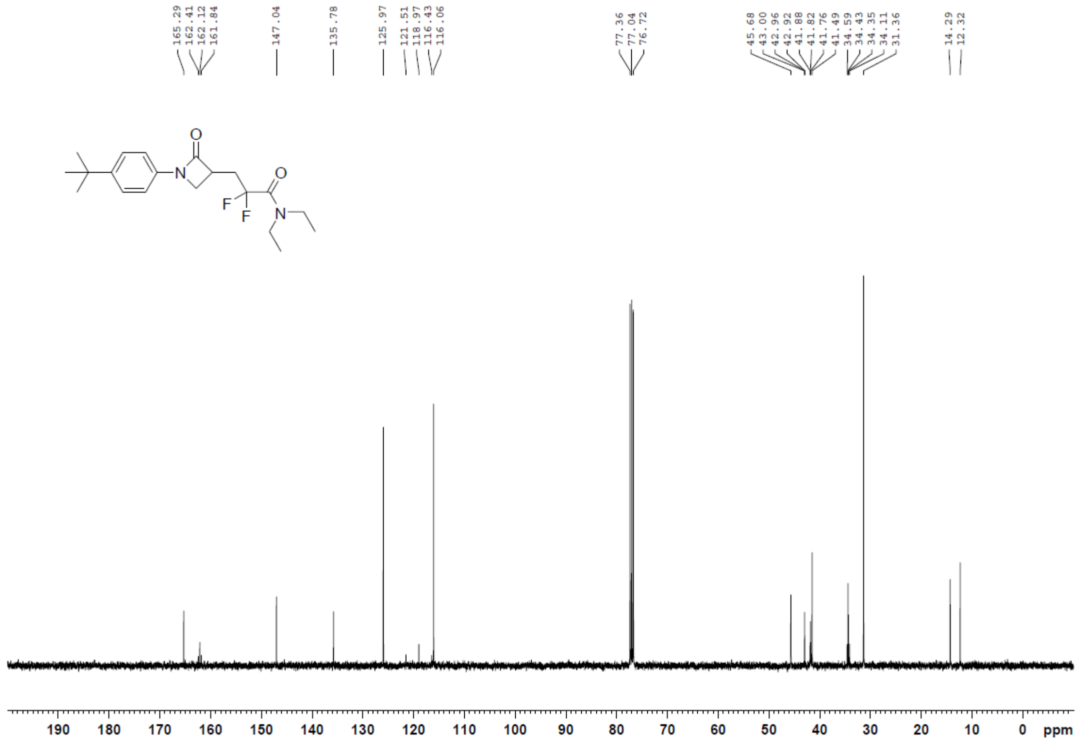

Supplementary Figure 227. ^{13}C NMR spectrum of **5o** in $CDCl_3$ (101 MHz)

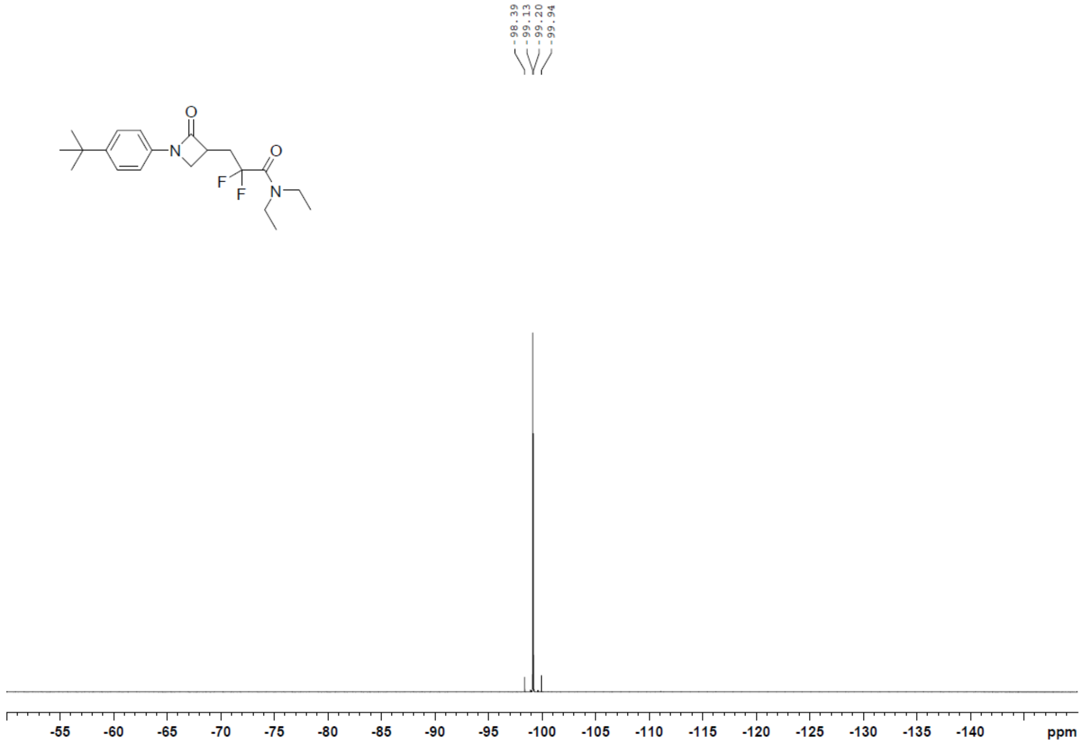

Supplementary Figure 228. ^{19}F NMR spectrum of **5o** in $CDCl_3$ (376 MHz)

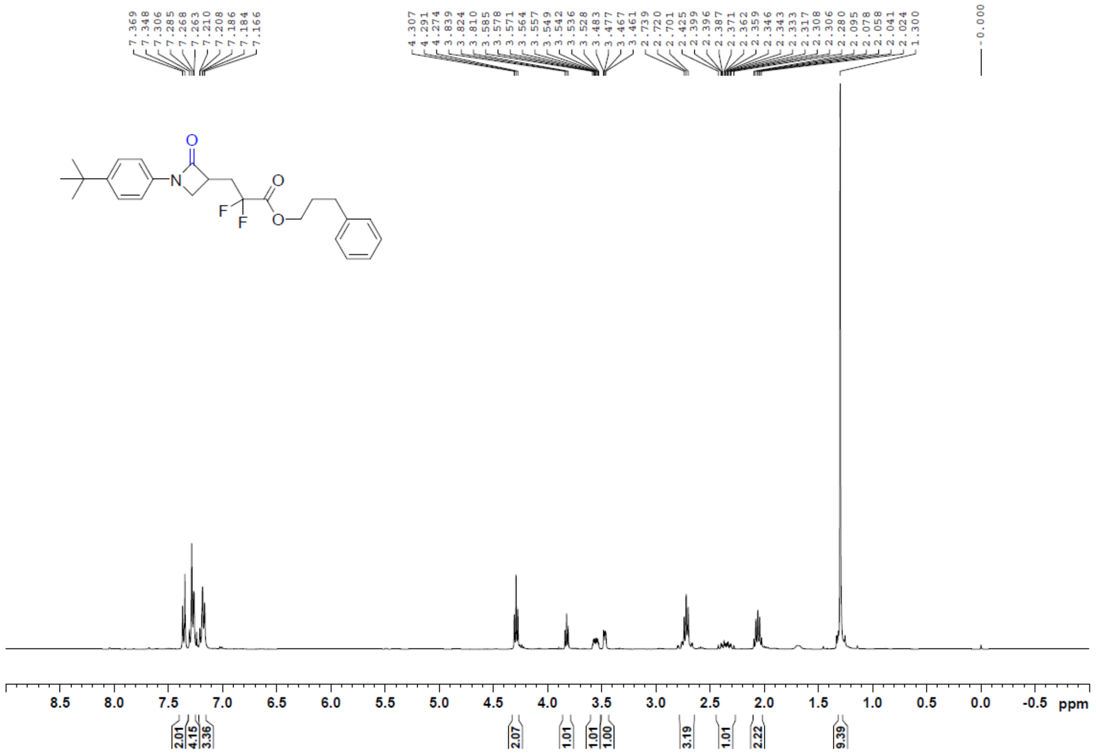

Supplementary Figure 229. ^{1}H NMR spectrum of **5p** in $CDCl_3$ (400 MHz)

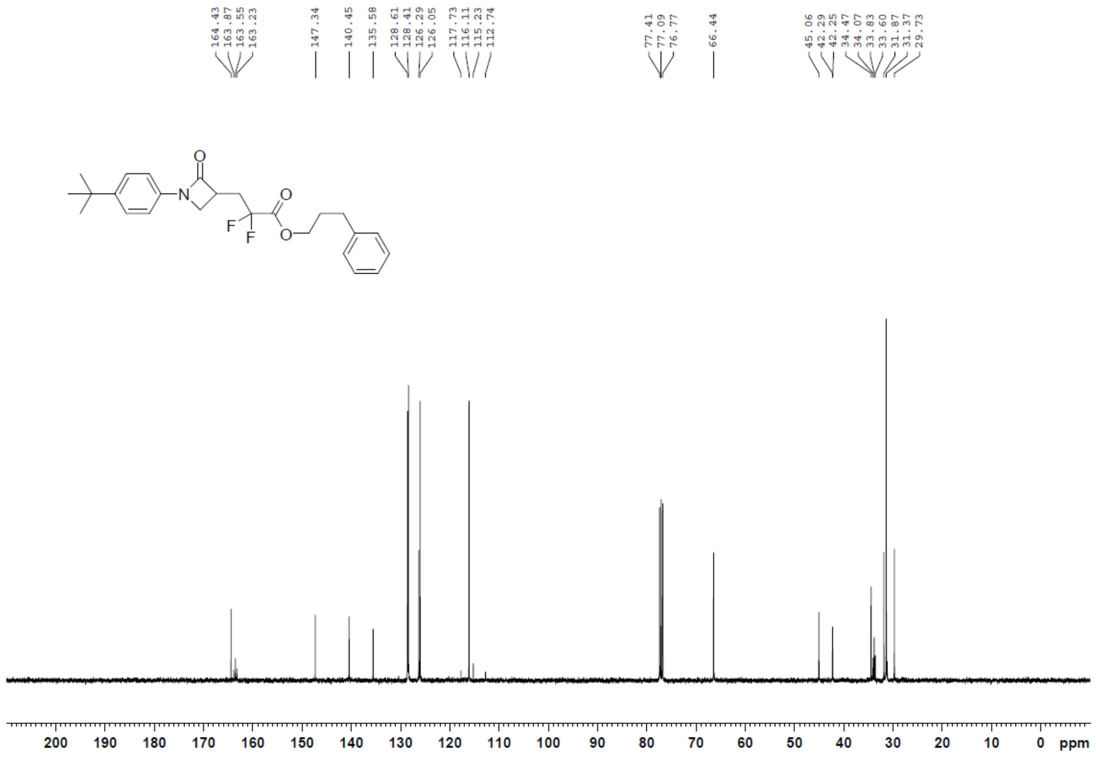

Supplementary Figure 230. ^{13}C NMR spectrum of **5p** in $CDCl_3$ (101 MHz)

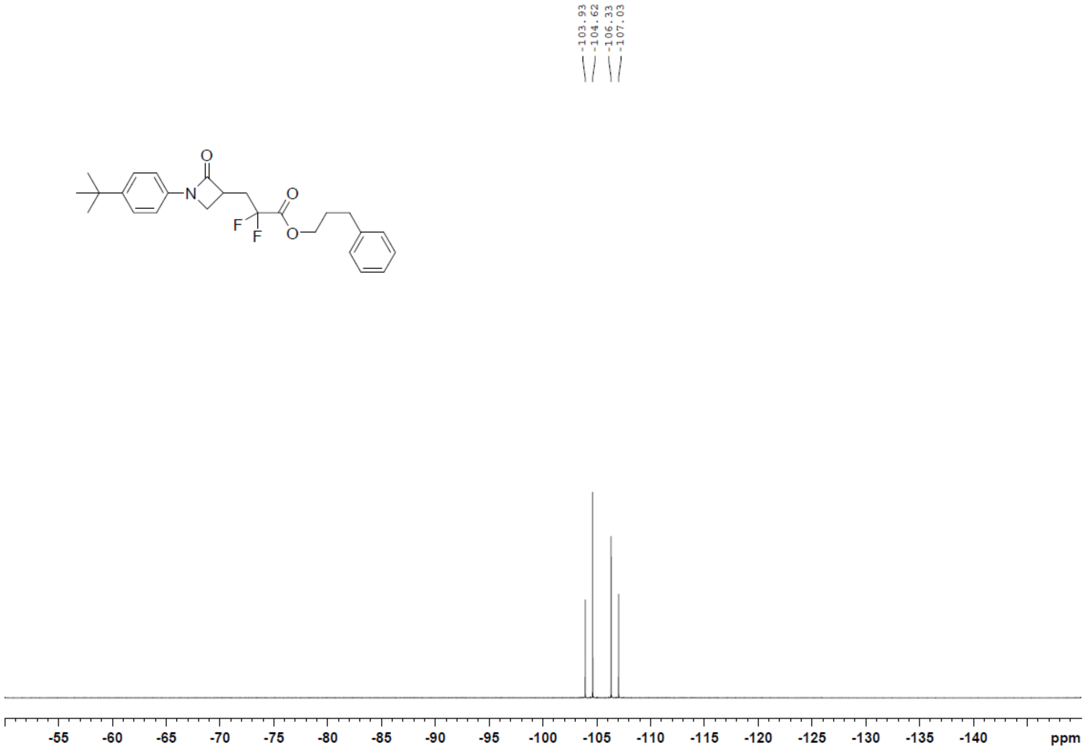

Supplementary Figure 231. ^{19}F NMR spectrum of **5p** in $CDCl_3$ (376 MHz)

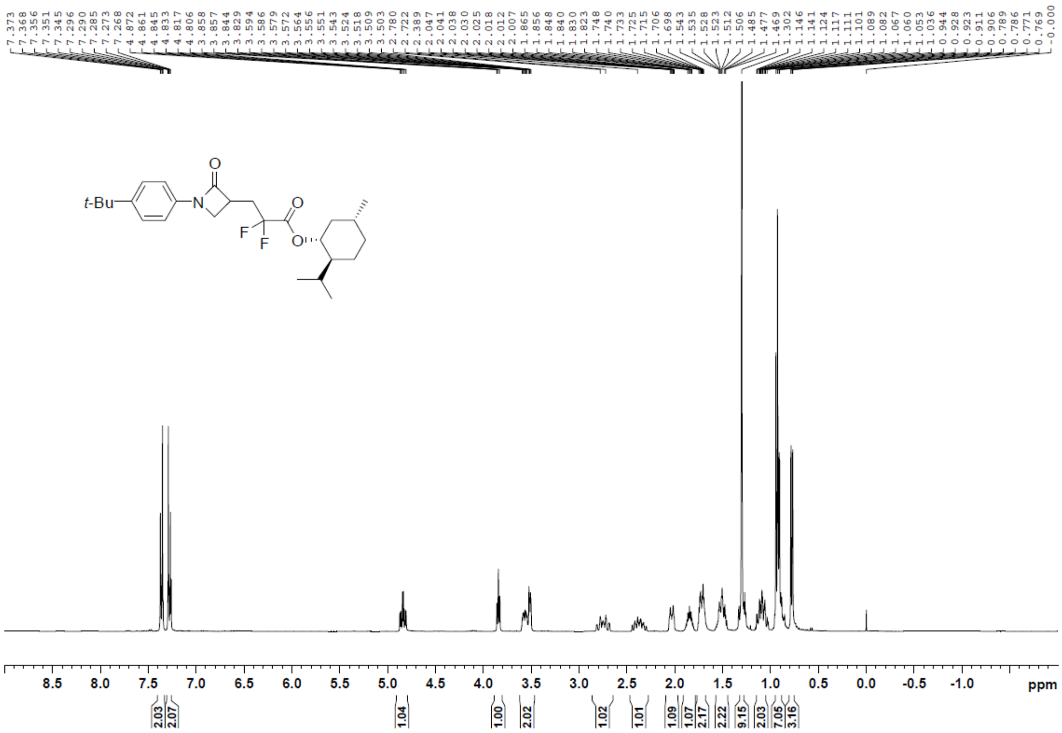

Supplementary Figure 232. ^{1}H NMR spectrum of **5q** in $CDCl_3$ (400 MHz)

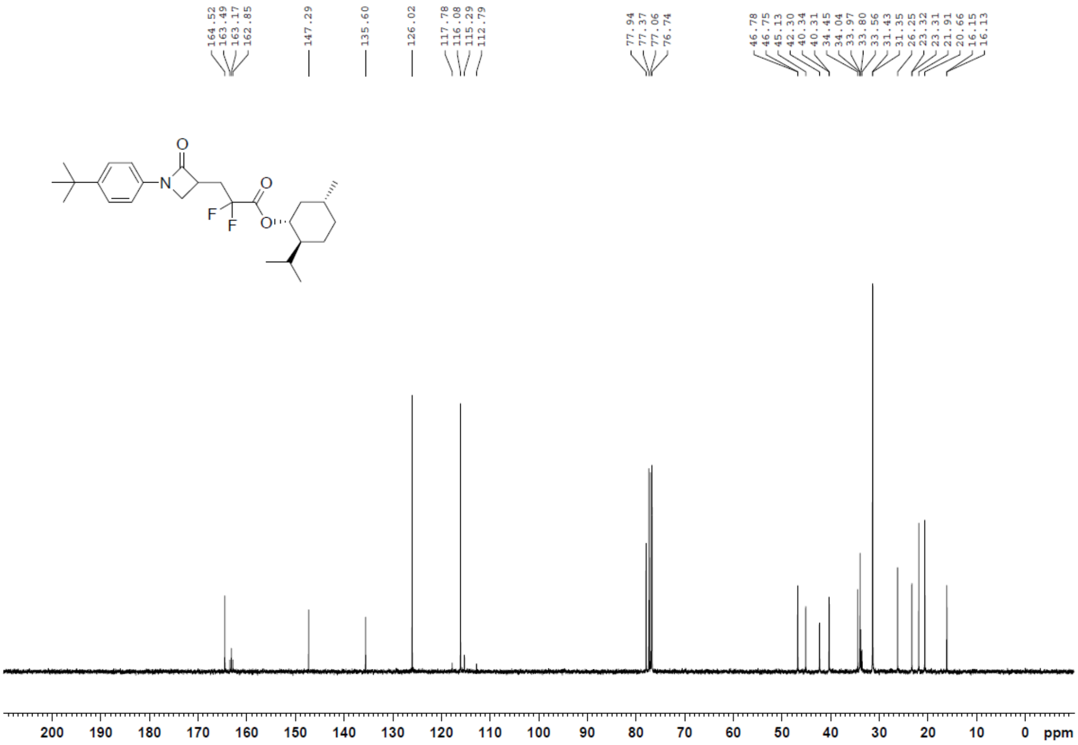

Supplementary Figure 233. ^{13}C NMR spectrum of **5q** in $CDCl_3$ (101 MHz)

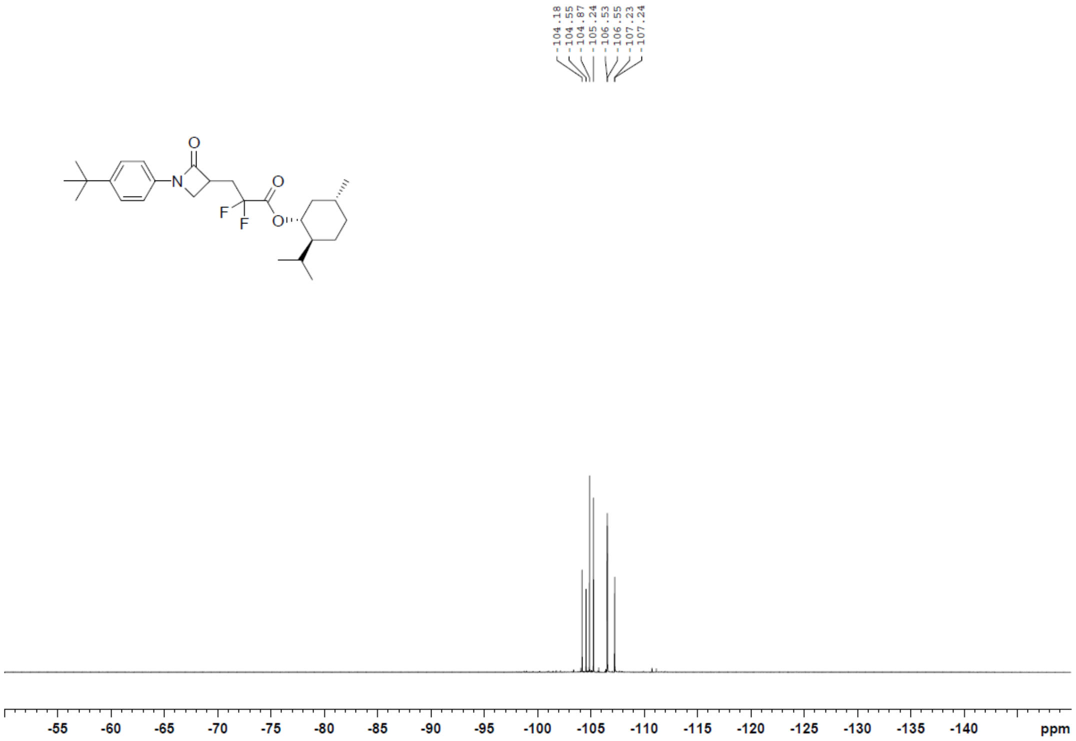

Supplementary Figure 234. ^{19}F NMR spectrum of **5q** in $CDCl_3$ (376 MHz)

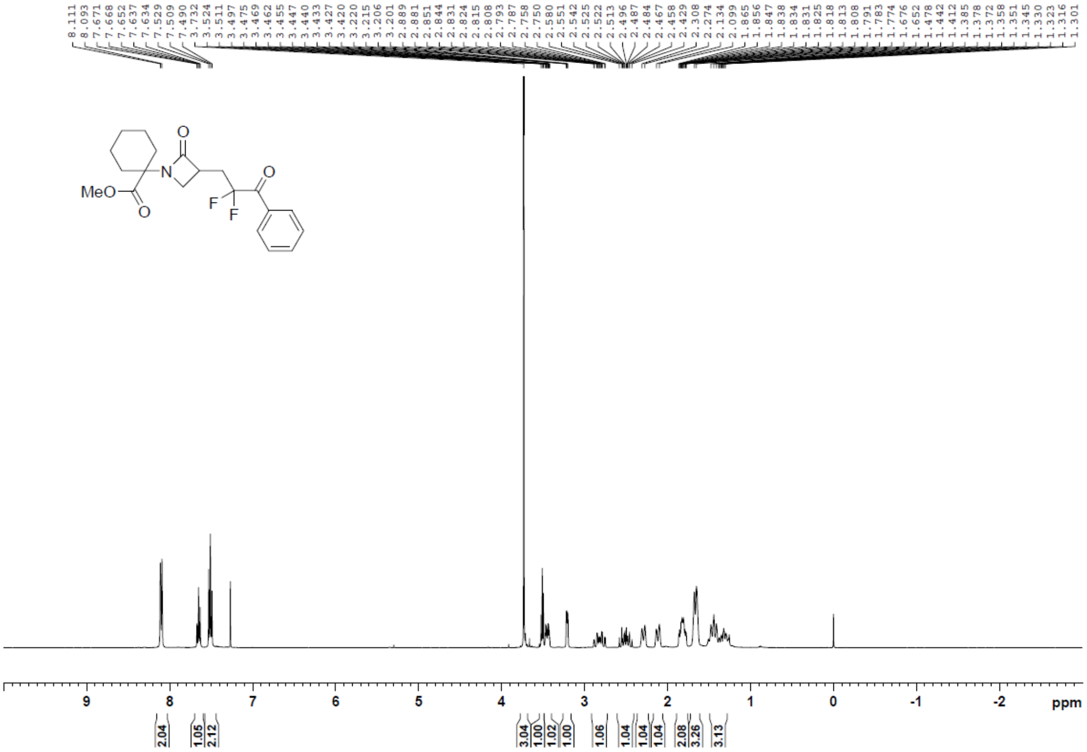

Supplementary Figure 235. ^{1}H NMR spectrum of **6a** in $CDCl_3$ (400 MHz)

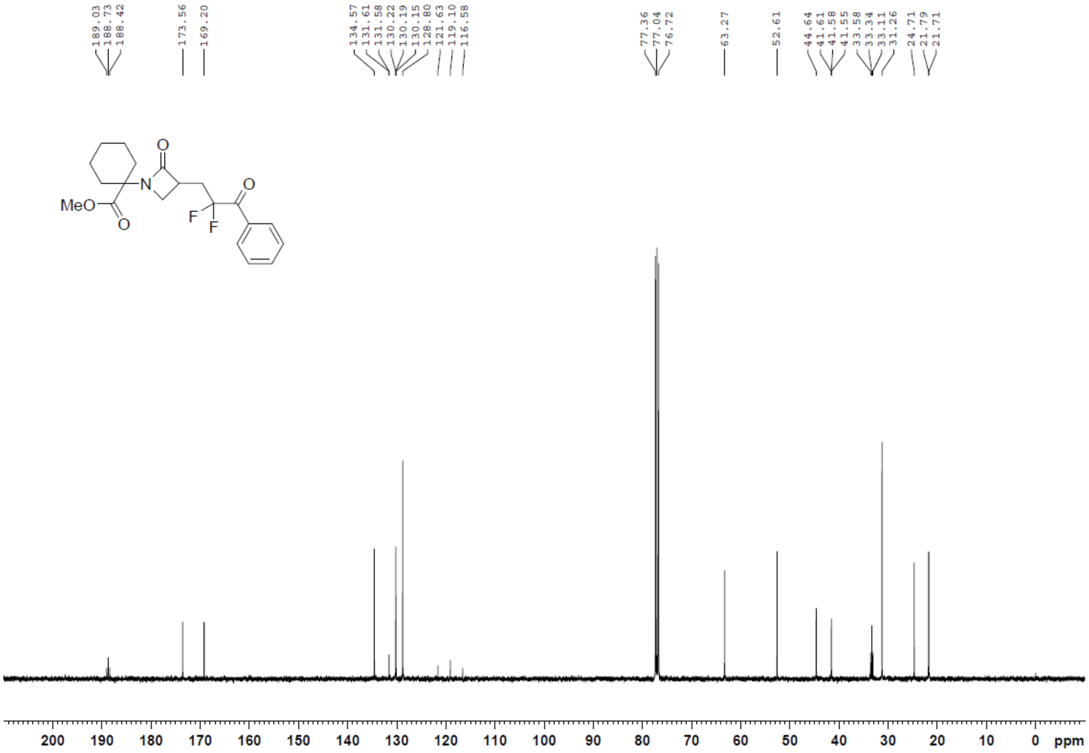

Supplementary Figure 236. ^{13}C NMR spectrum of **6a** in $CDCl_3$ (101 MHz)

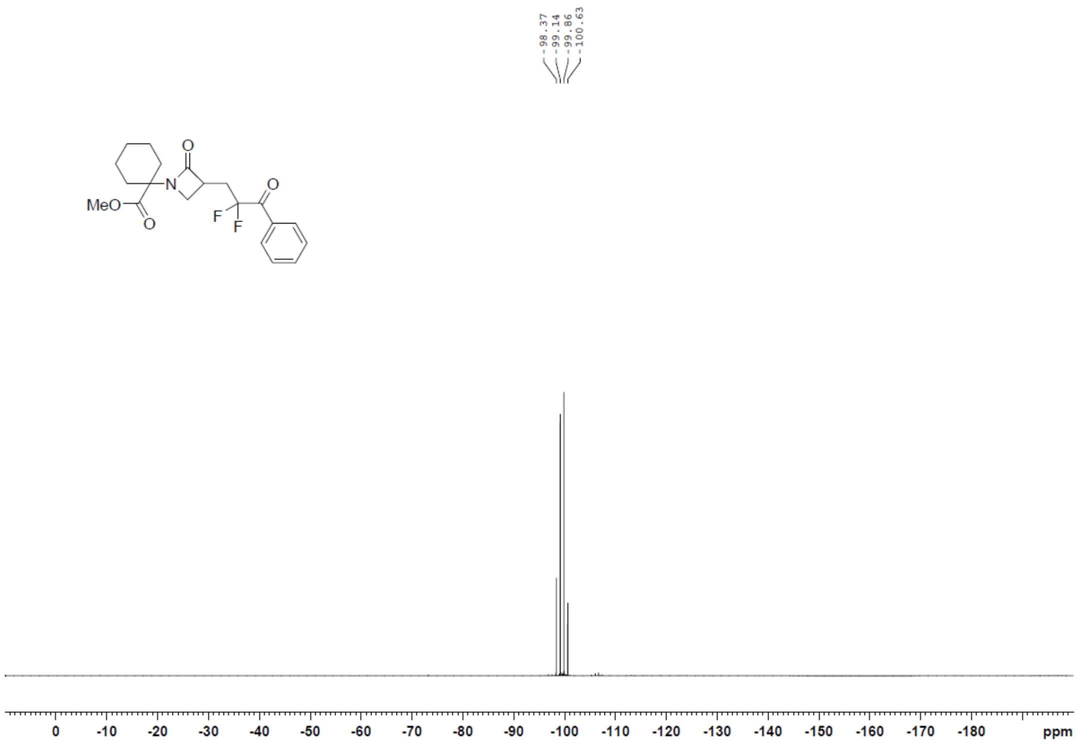

Supplementary Figure 237. ^{19}F NMR spectrum of **6a** in $CDCl_3$ (376 MHz)

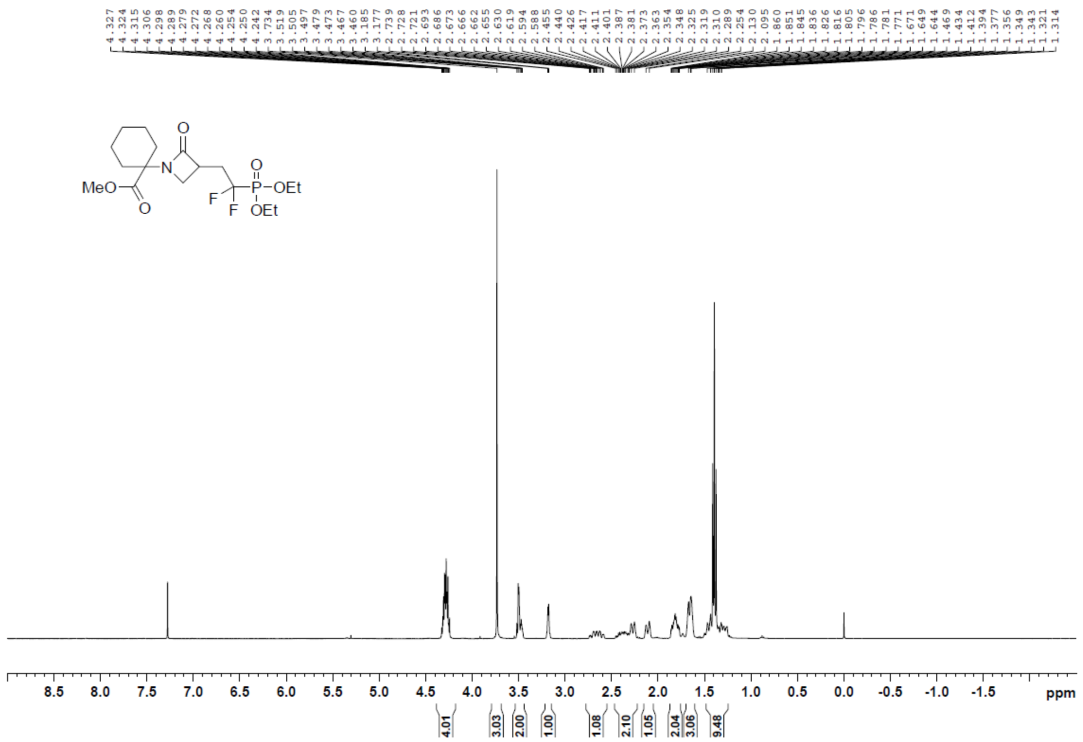

Supplementary Figure 238. ^{1}H NMR spectrum of 6**b** in $CDCl_3$ (400 MHz)

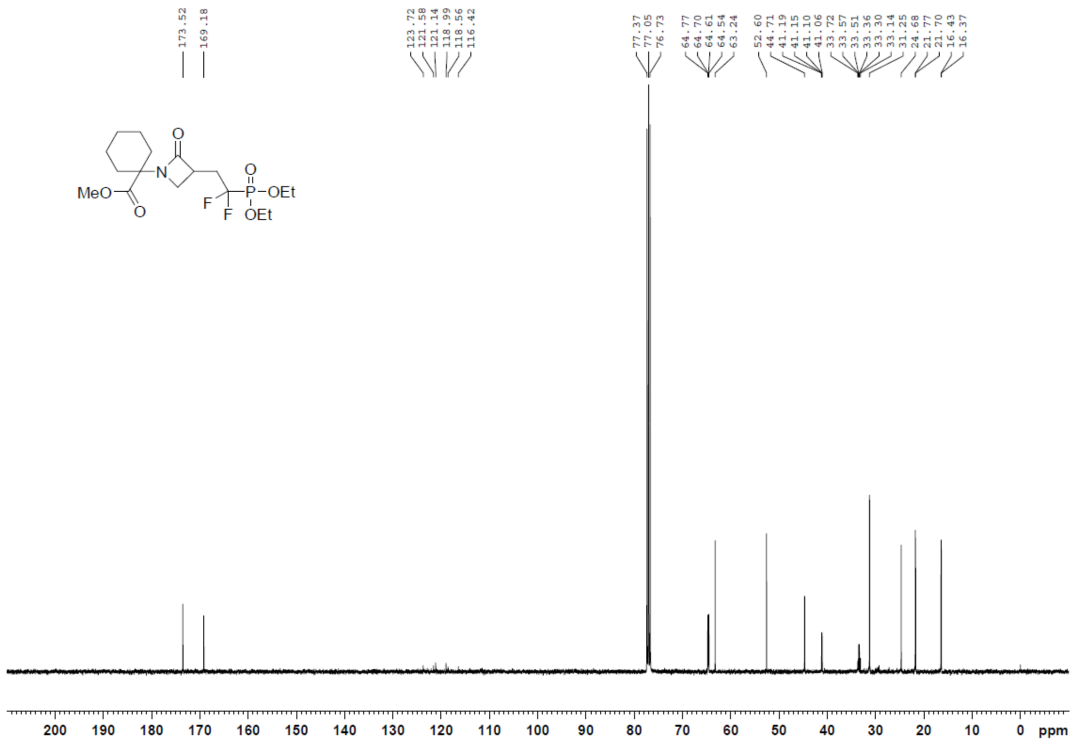

Supplementary Figure 239. ^{13}C NMR spectrum of **6b** in $CDCl_3$ (101 MHz)

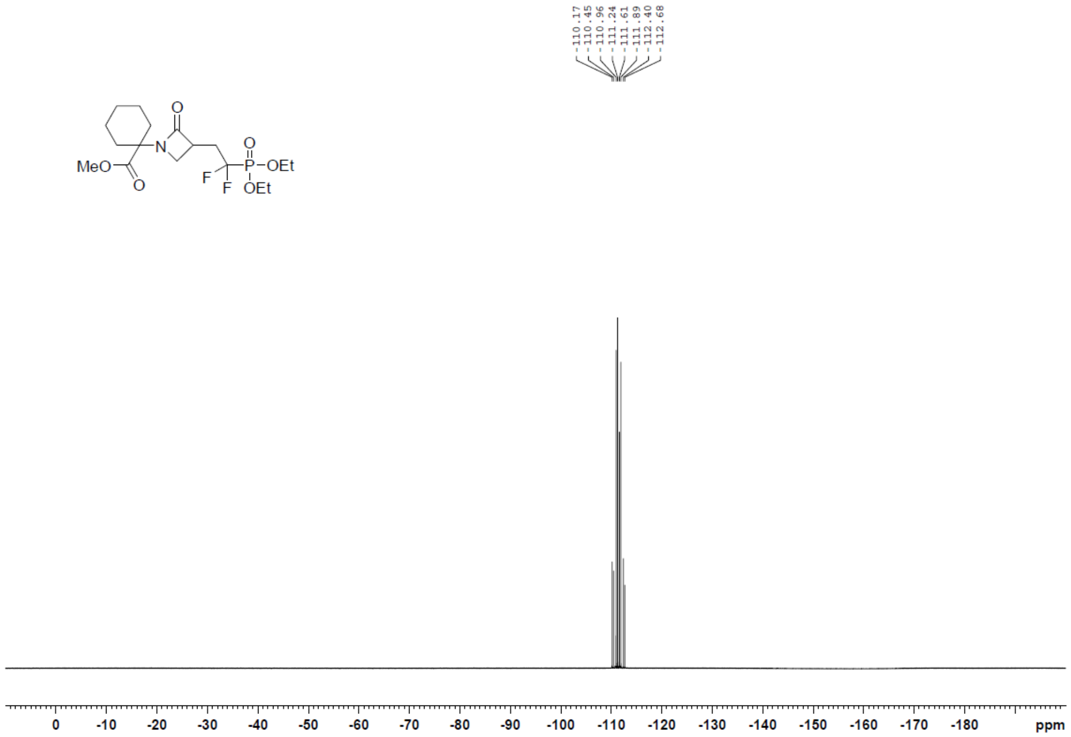

Supplementary Figure 240. ^{19}F NMR spectrum of **6b** in $CDCl_3$ (376 MHz)

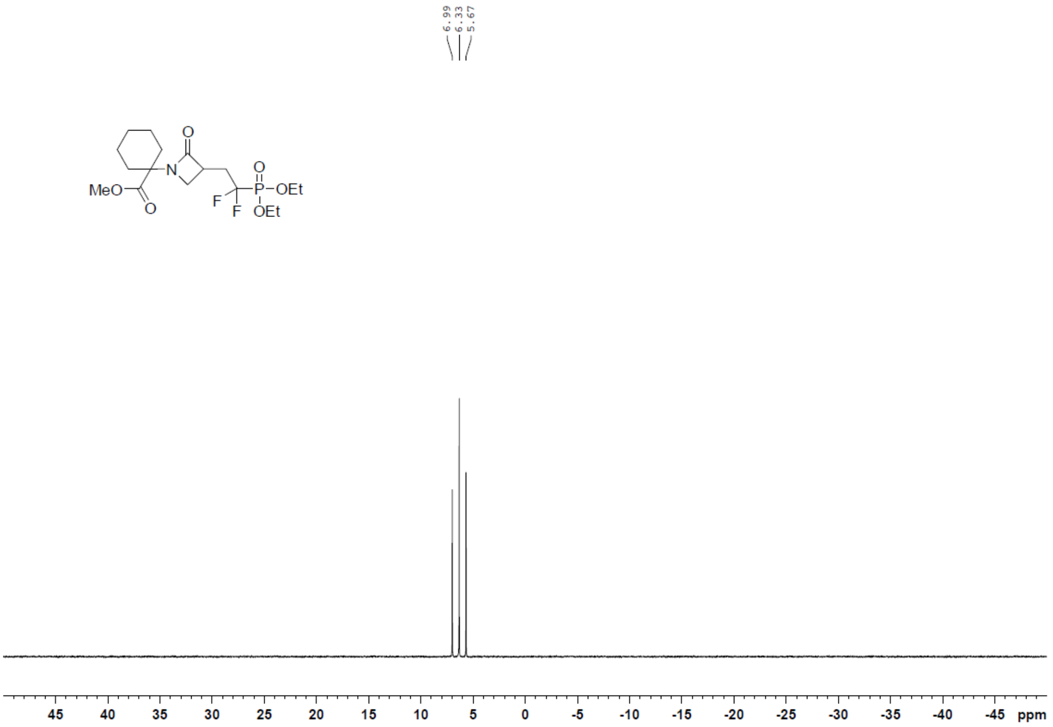

Supplementary Figure 241. ^{31}P NMR spectrum of **6b** in $CDCl_3$ (162 MHz)

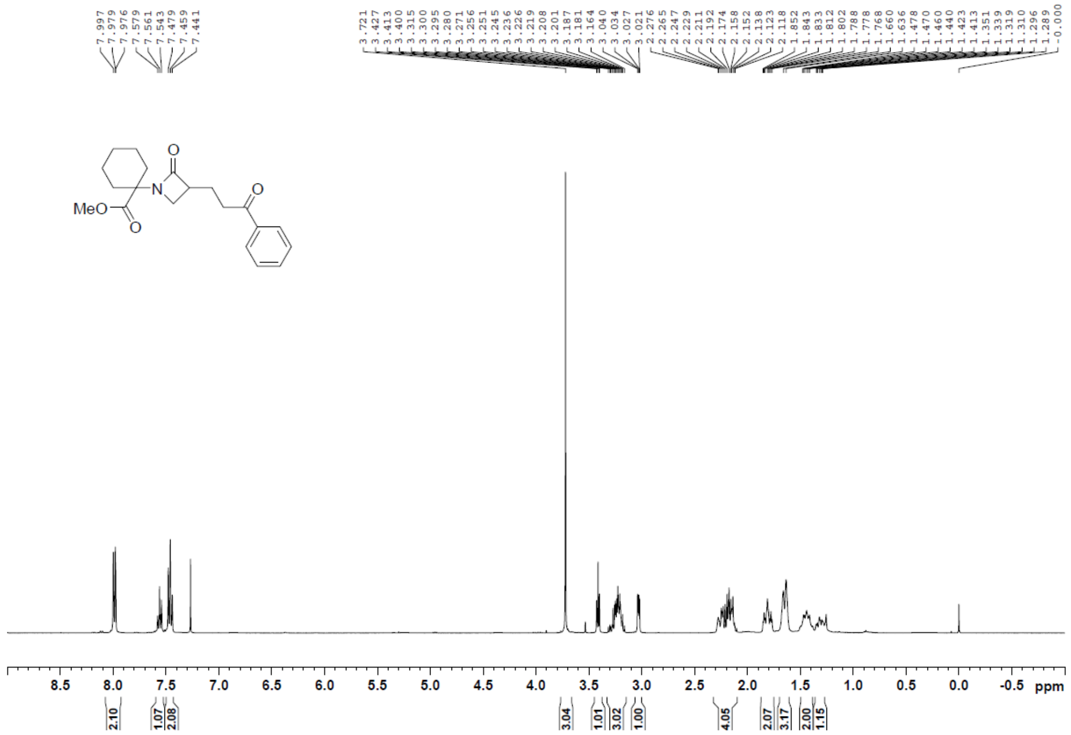

Supplementary Figure 242. ^{1}H NMR spectrum of **6c** in $CDCl_3$ (400 MHz)

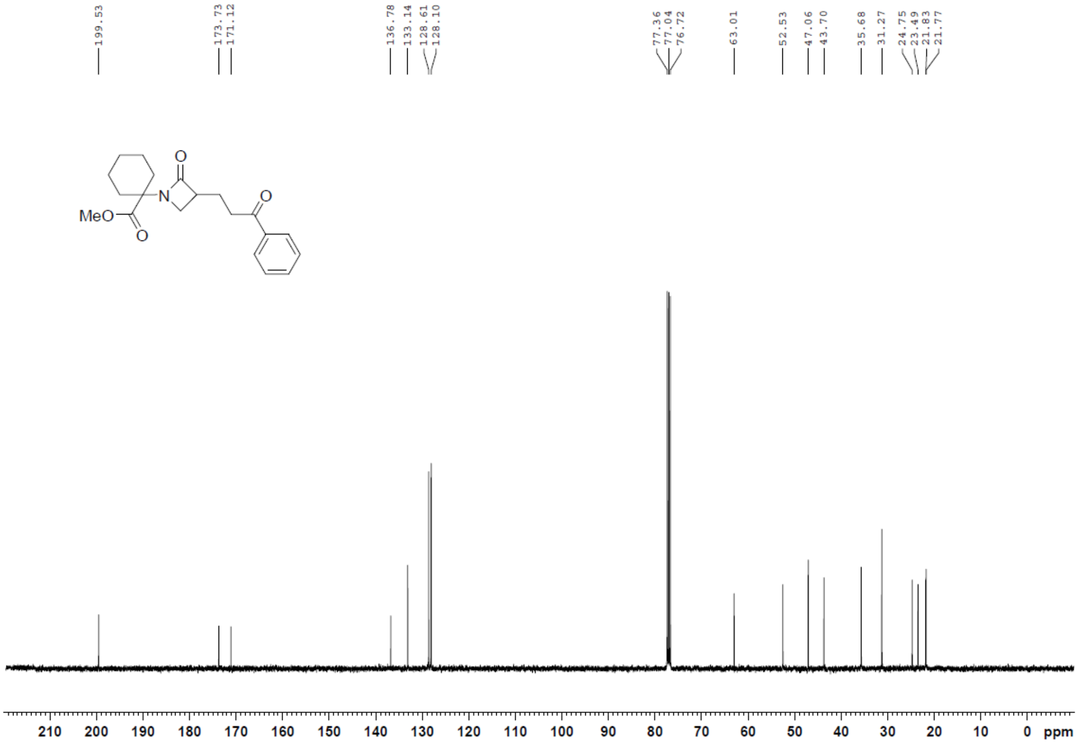

Supplementary Figure 243. ^{13}C NMR spectrum of **6c** in $CDCl_3$ (101 MHz)

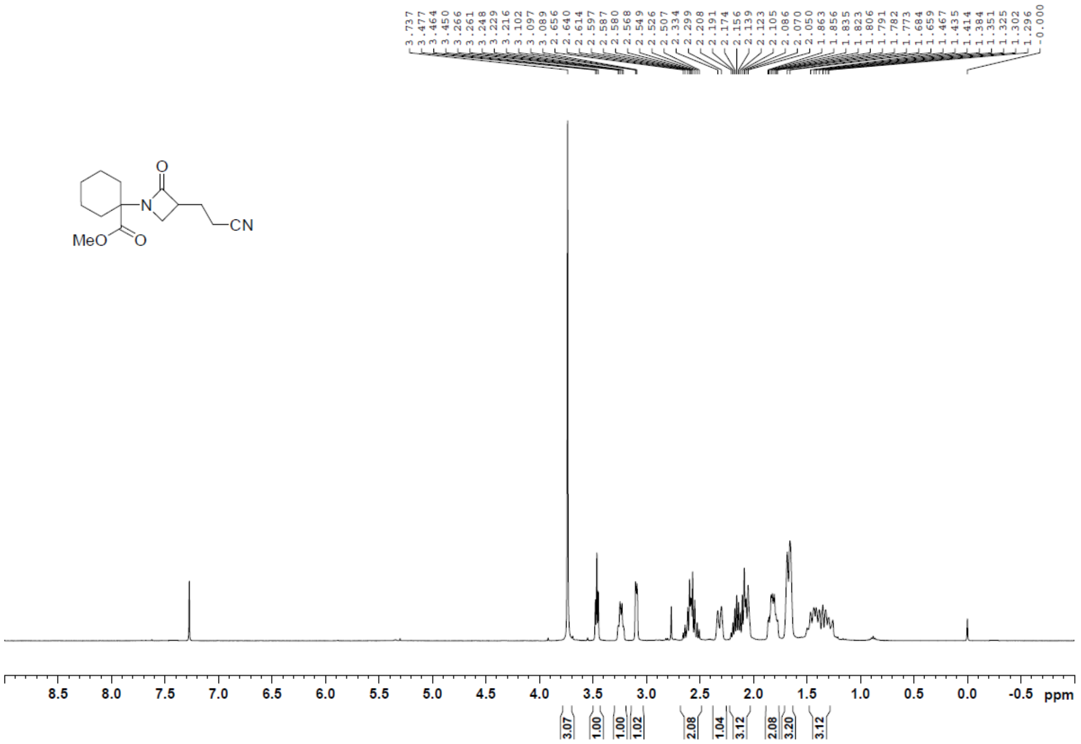

Supplementary Figure 244. ^{1}H NMR spectrum of **6d** in $CDCl_3$ (400 MHz)

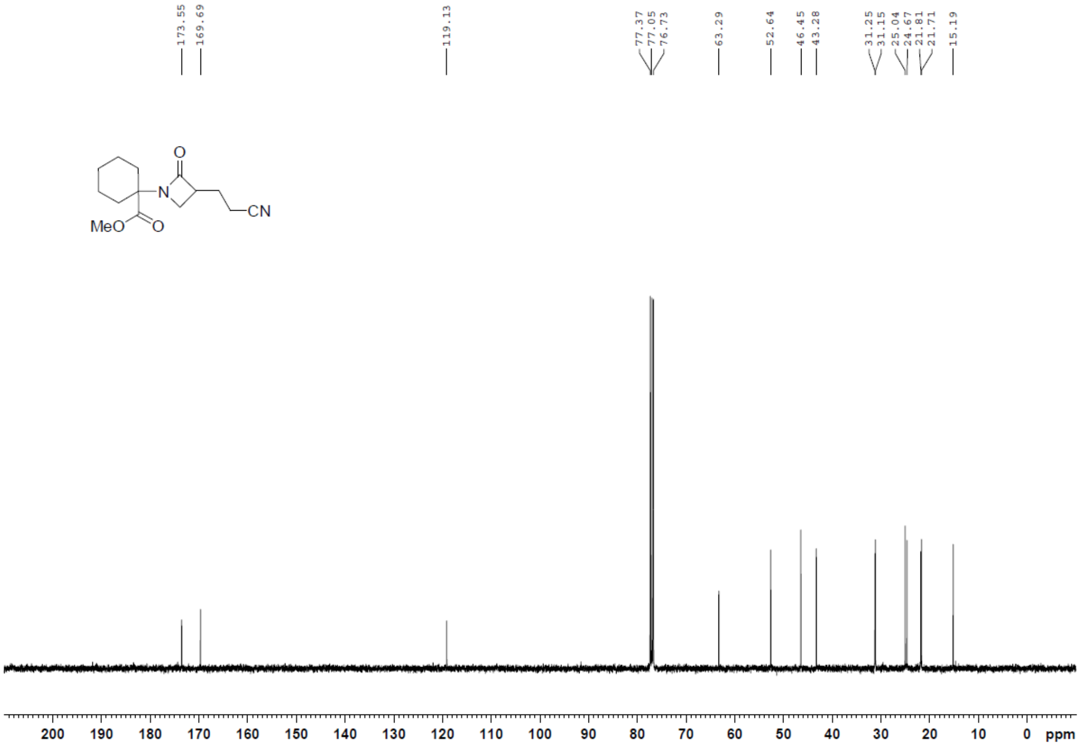

Supplementary Figure 245. ^{13}C NMR spectrum of **6d** in $CDCl_3$ (101 MHz)

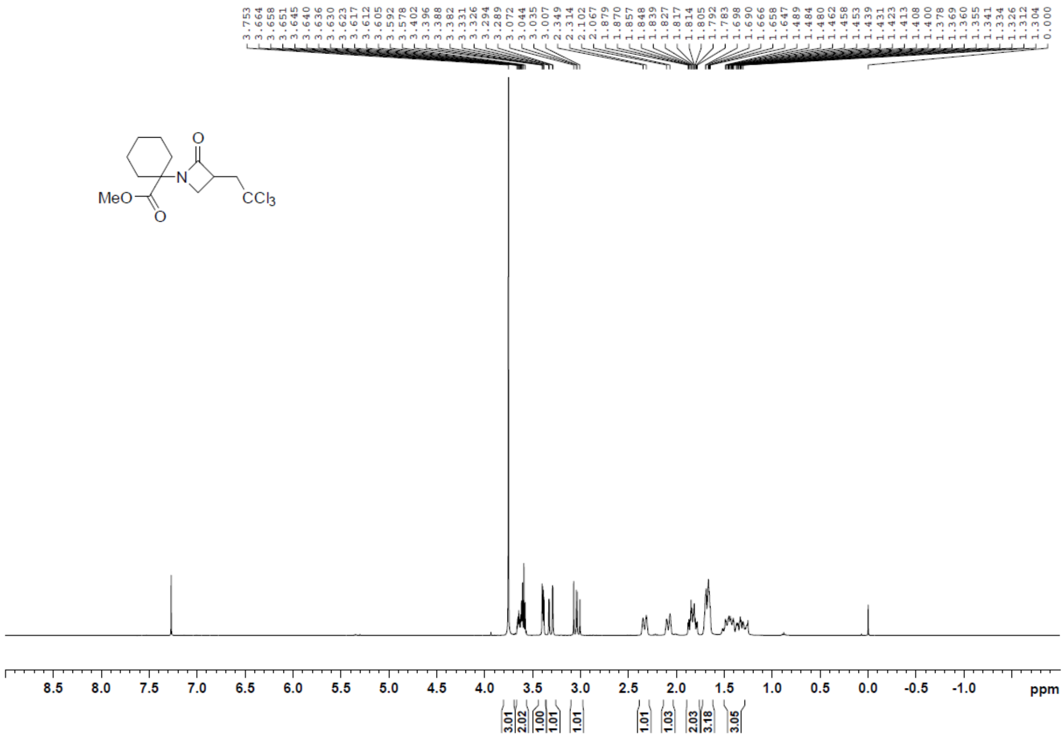

Supplementary Figure 246. ^{1}H NMR spectrum of **6e** in $CDCl_3$ (400 MHz)

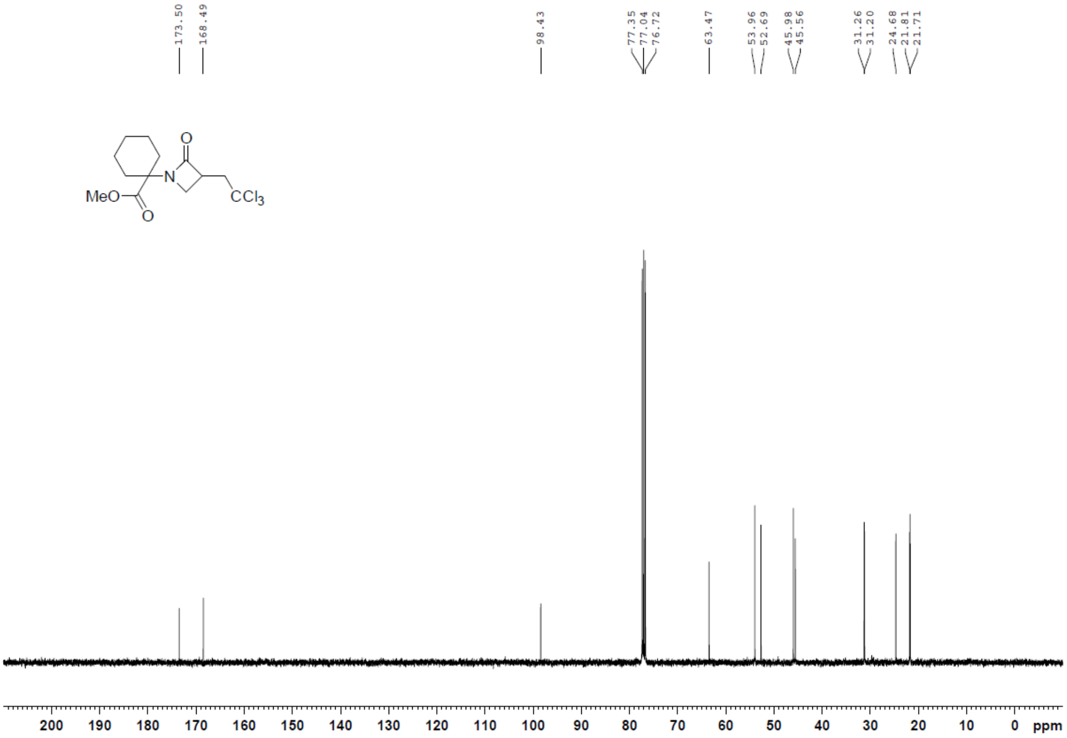

Supplementary Figure 247. ^{13}C NMR spectrum of **6e** in $CDCl_3$ (101 MHz)

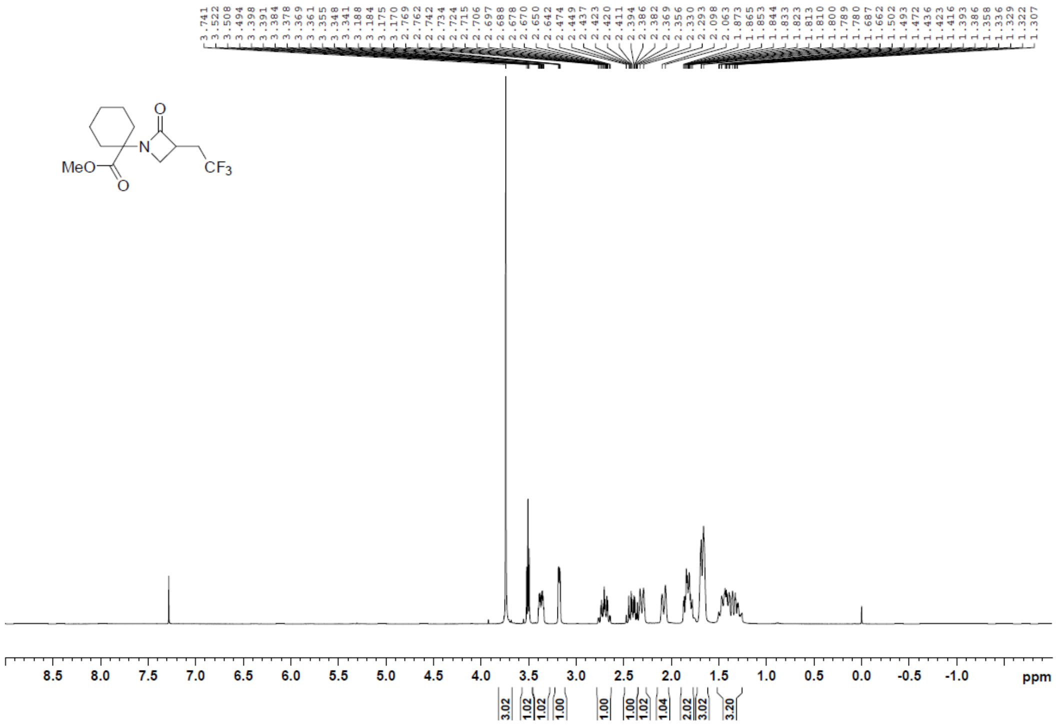

Supplementary Figure 248. ^{1}H NMR spectrum of **6f** in $CDCl_3$ (400 MHz)

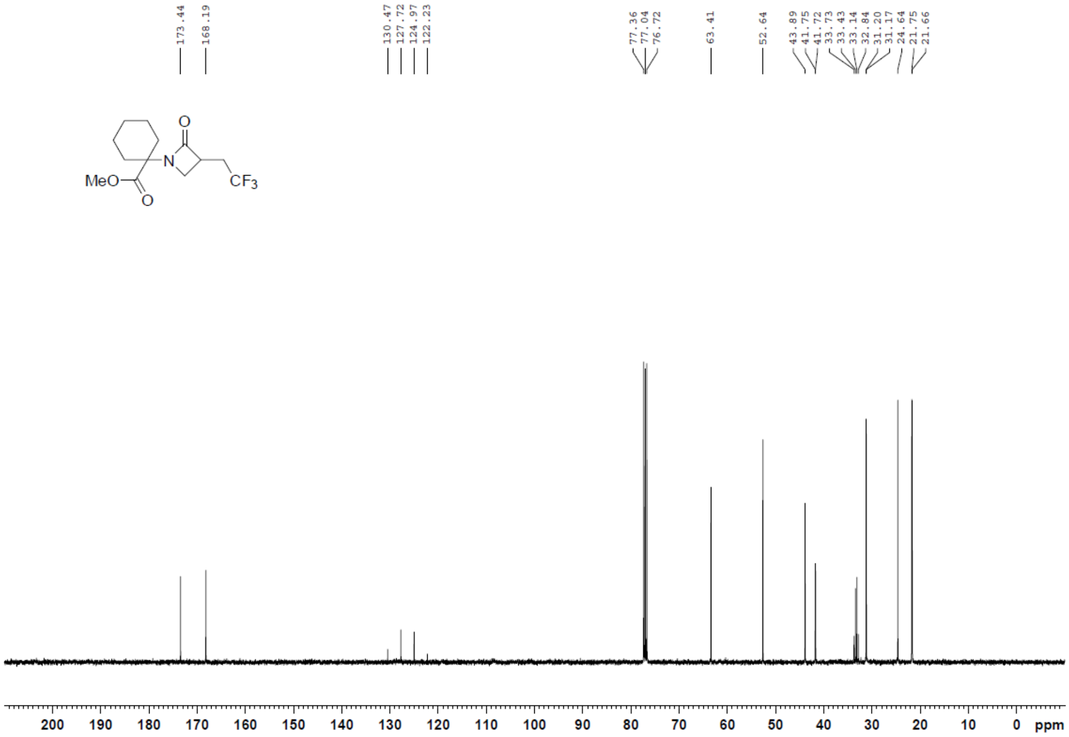

Supplementary Figure 249. ^{13}C NMR spectrum of **6f** in $CDCl_3$ (101 MHz)

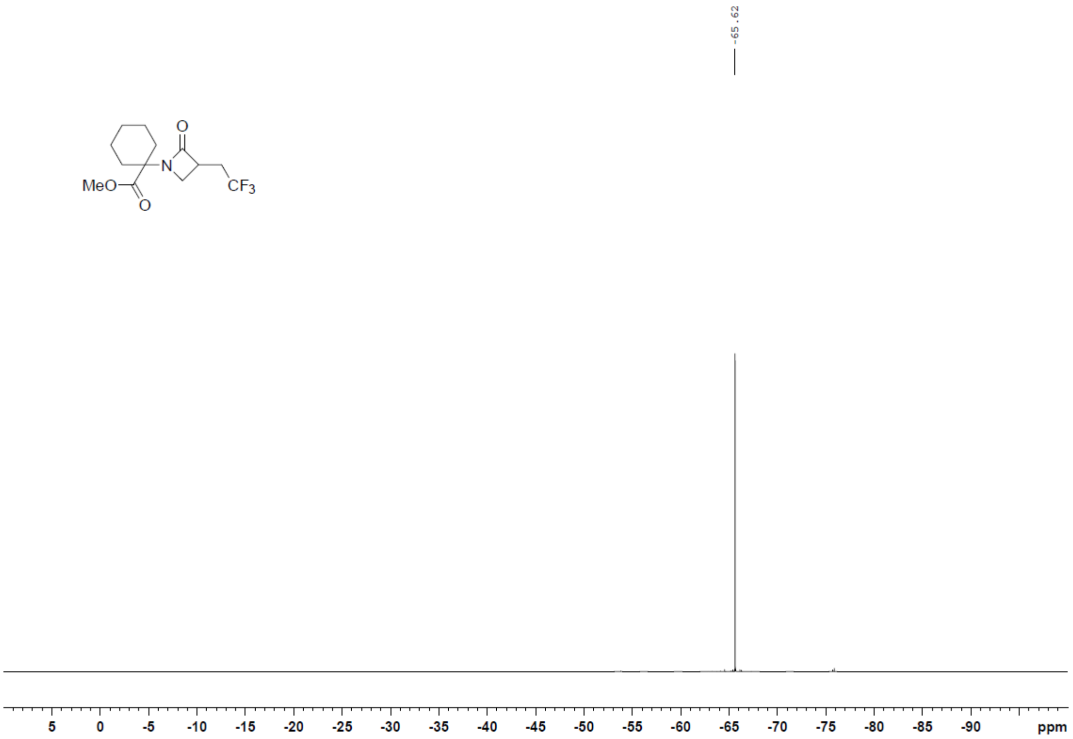

Supplementary Figure 250. ^{19}F NMR spectrum of **6f** in $CDCl_3$ (376 MHz)

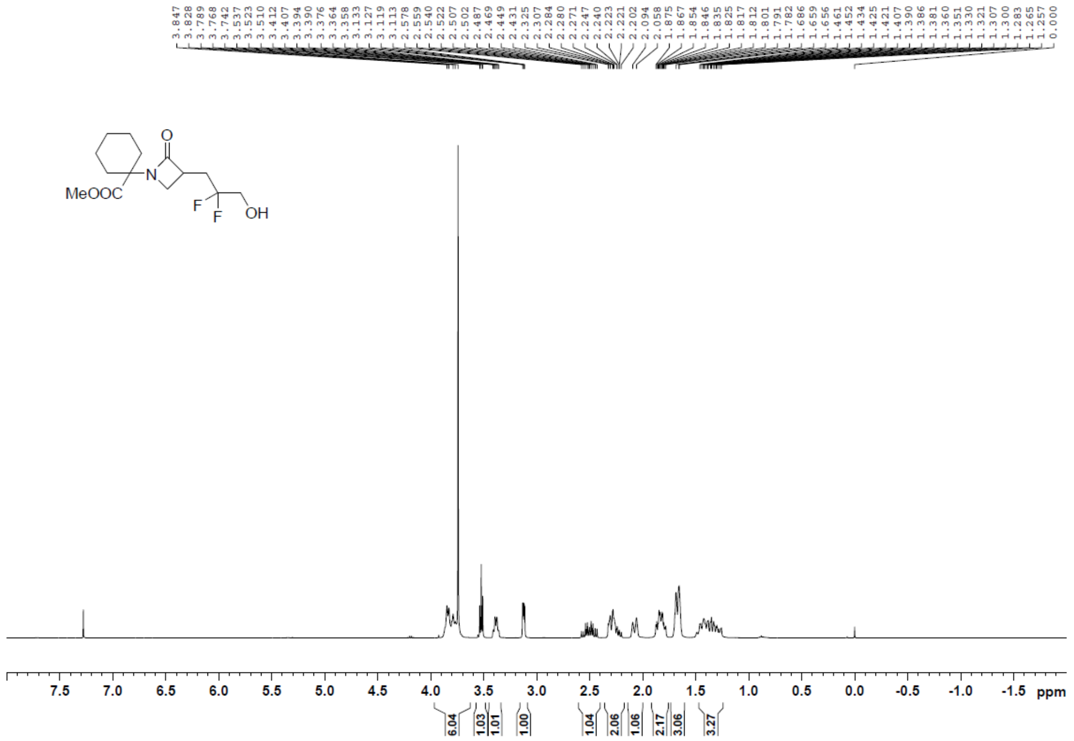

Supplementary Figure 251. ^{1}H NMR spectrum of **7** in $CDCl_3$ (400 MHz)

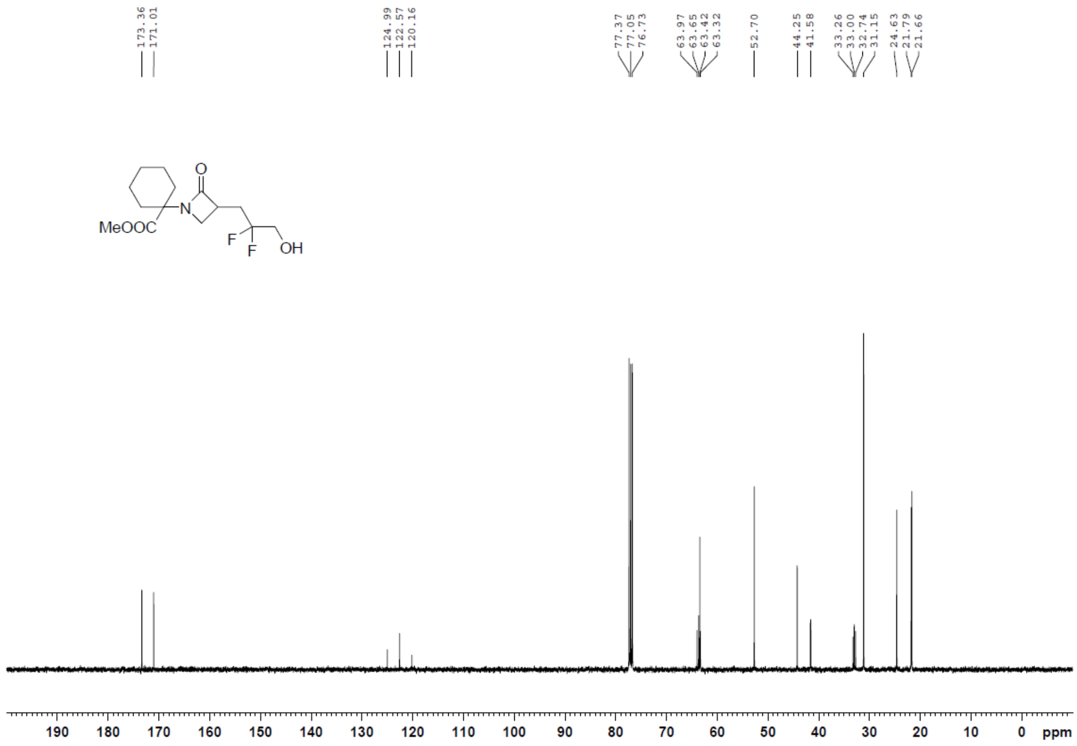

Supplementary Figure 252. ^{13}C NMR spectrum of **7** in $CDCl_3$ (101 MHz)

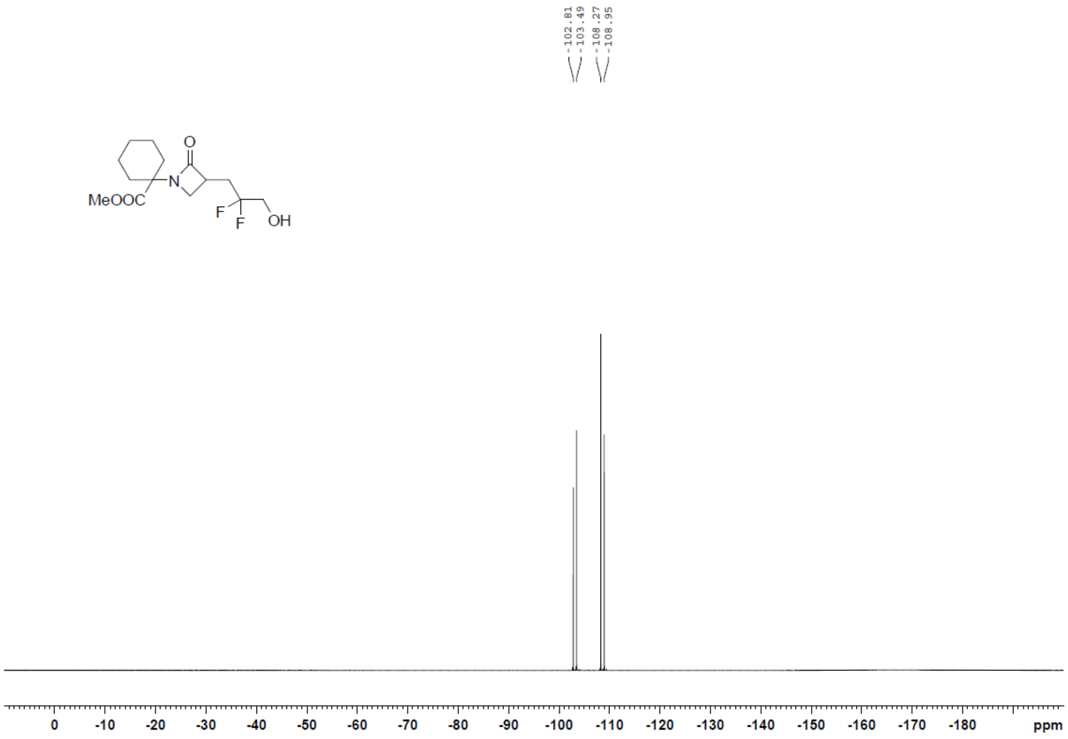

Supplementary Figure 253. ^{19}F NMR spectrum of **7** in $CDCl_3$ (376 MHz)

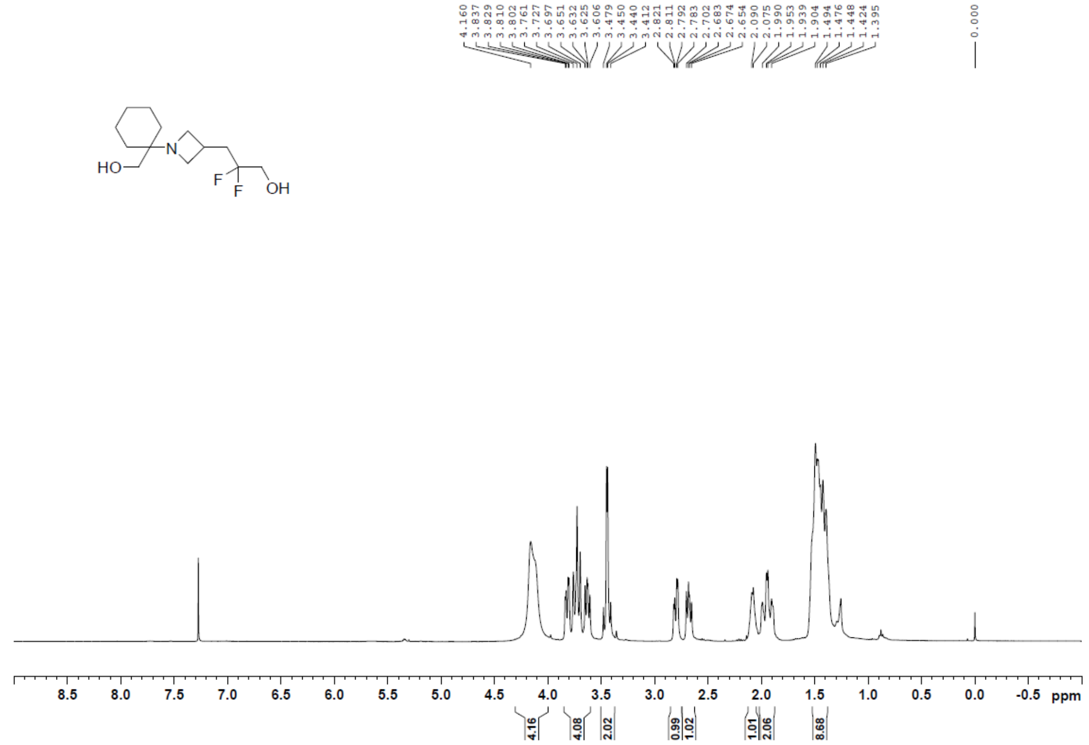

Supplementary Figure 254. ^{1}H NMR spectrum of **8** in $CDCl_3$ (400 MHz)

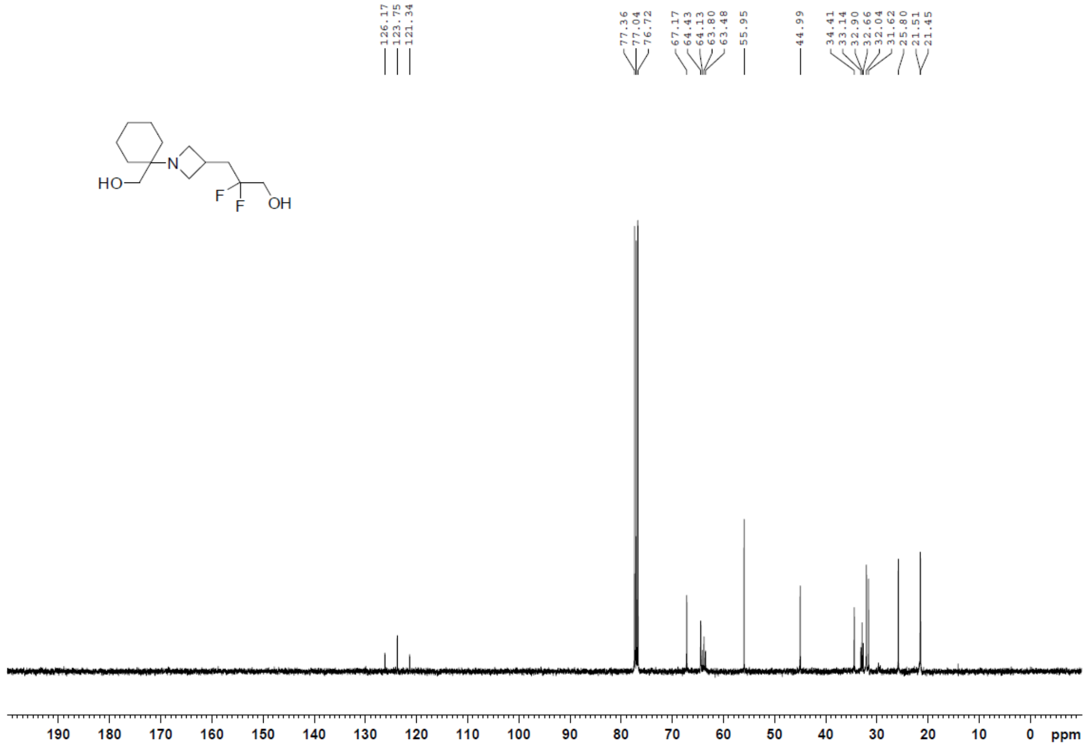

Supplementary Figure 255. ^{13}C NMR spectrum of **8** in $CDCl_3$ (101 MHz)

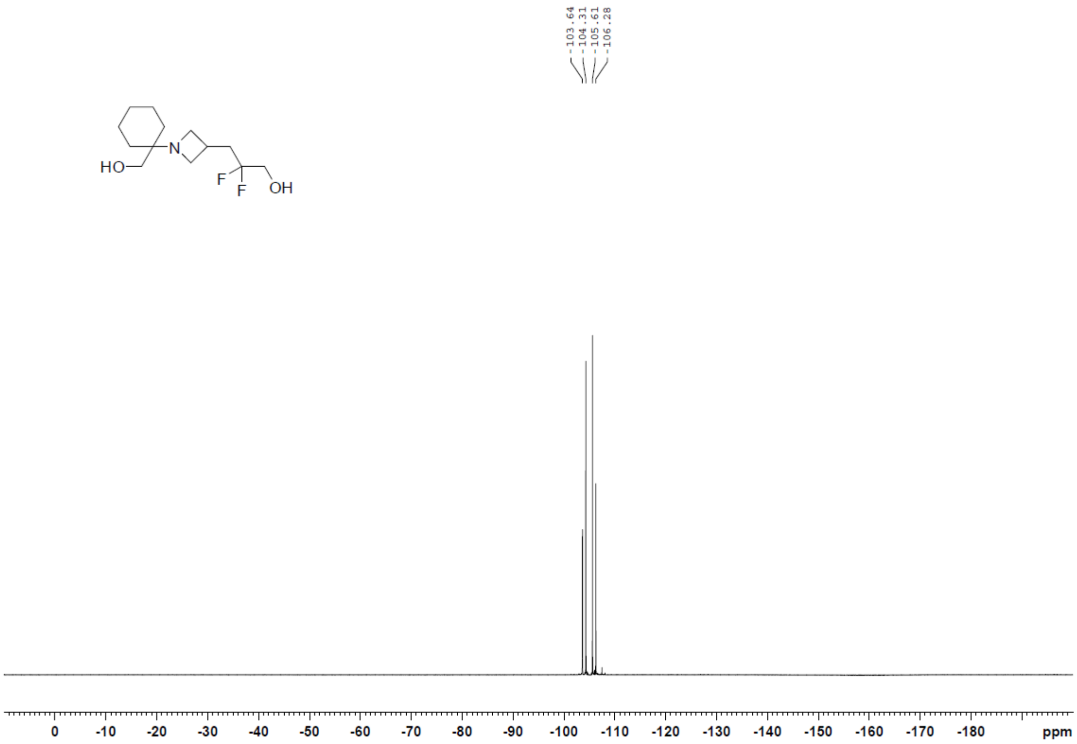

Supplementary Figure 256. ^{19}F NMR spectrum of **8** in $CDCl_3$ (376 MHz)

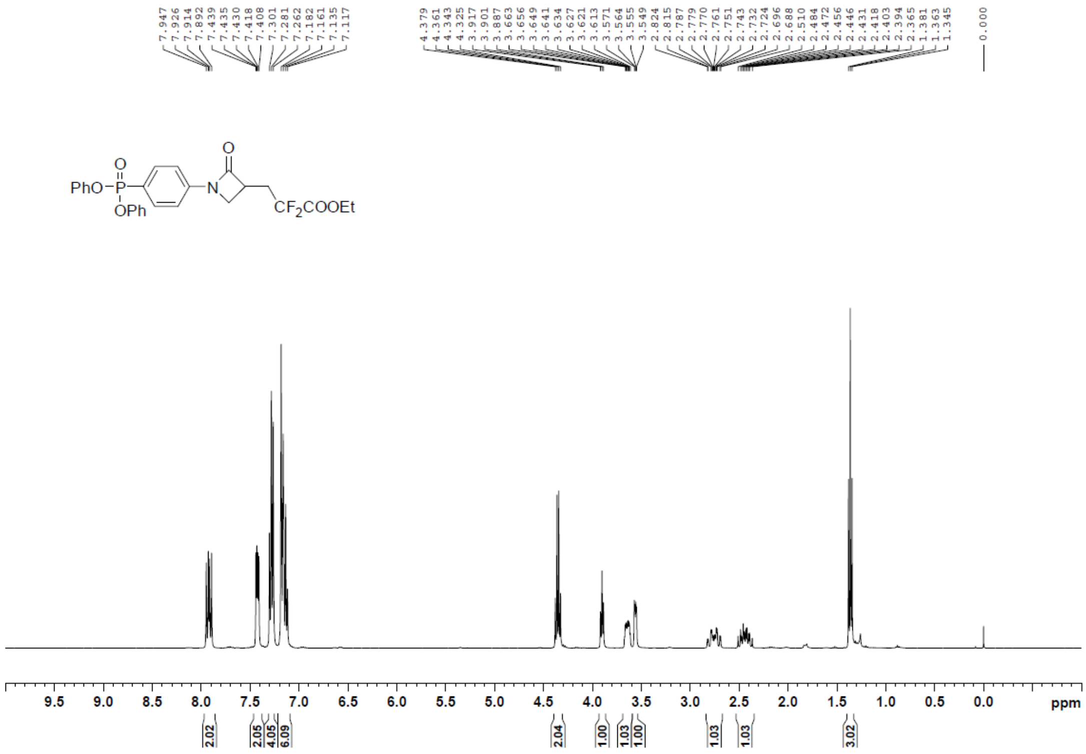

Supplementary Figure 257. ^{1}H NMR spectrum of **11** in $CDCl_3$ (400 MHz)

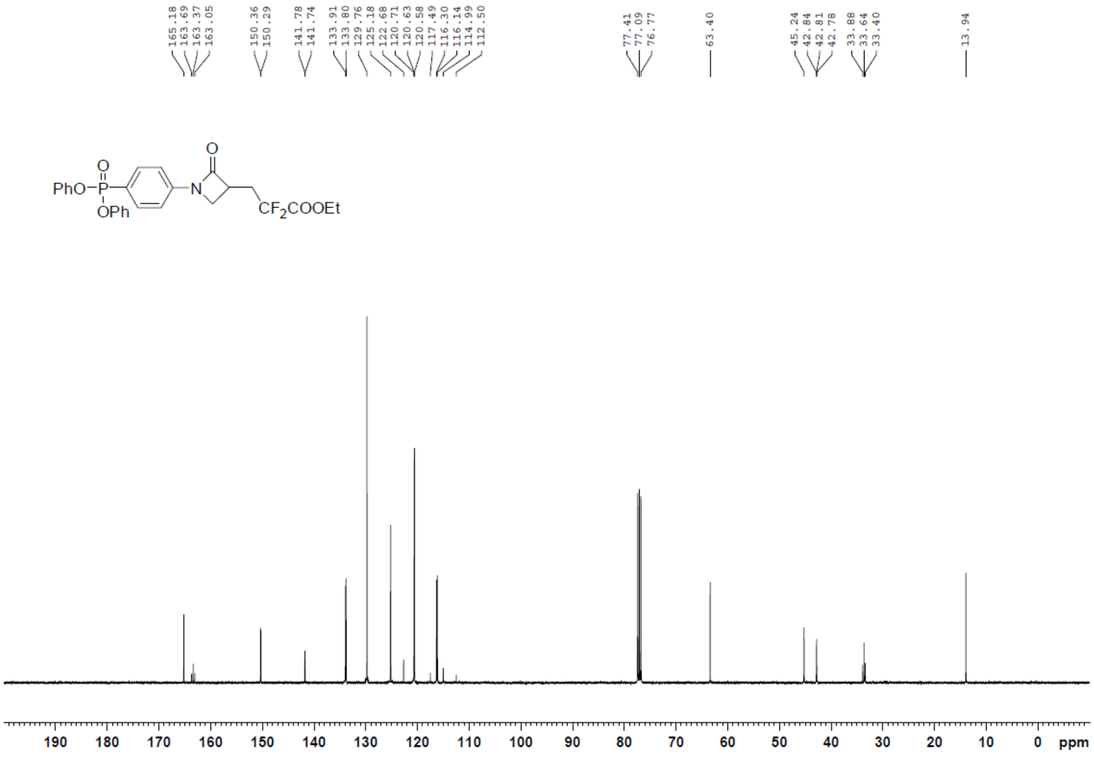

Supplementary Figure 258. ^{13}C NMR spectrum of **11** in $CDCl_3$ (101 MHz)

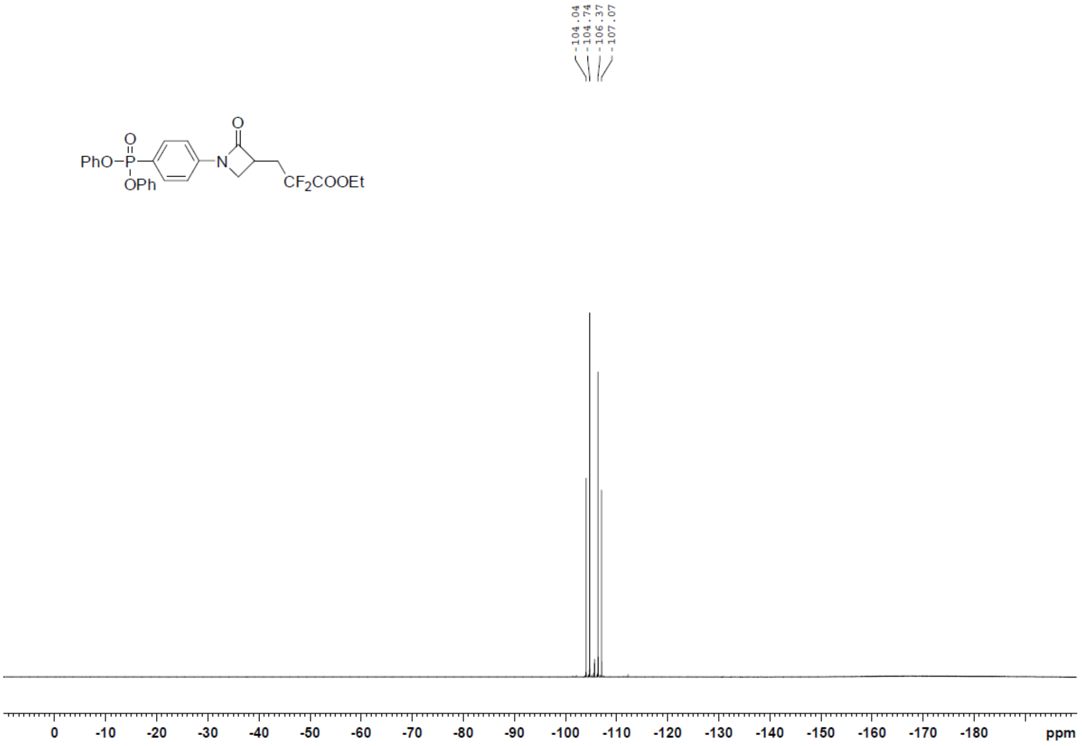

Supplementary Figure 259. ^{19}F NMR spectrum of **11** in $CDCl_3$ (376 MHz)

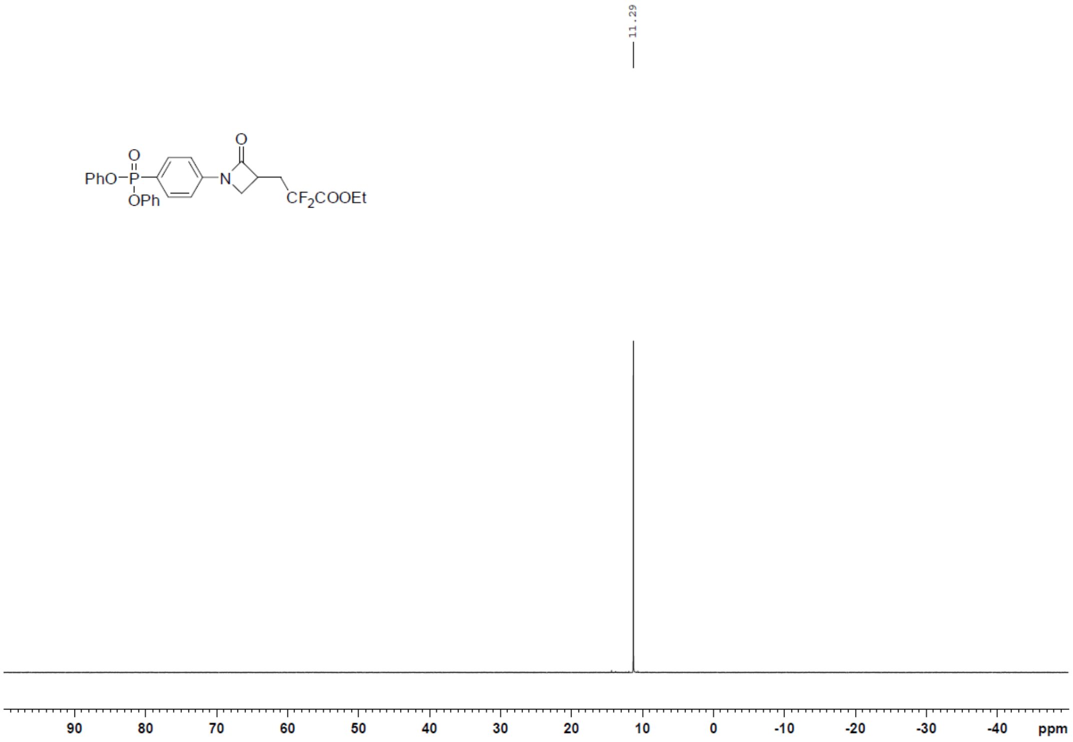

Supplementary Figure 260. ^{31}P NMR spectrum of **11** in $CDCl_3$ (162 MHz)

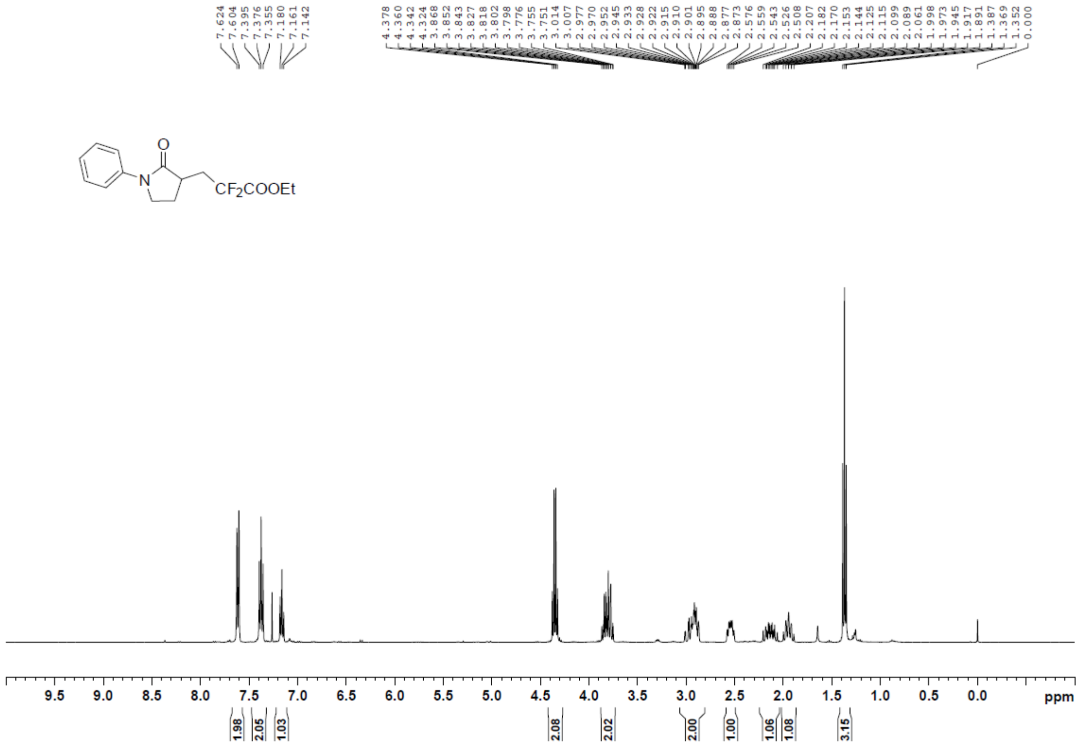

Supplementary Figure 261. ^{1}H NMR spectrum of **14** in $CDCl_3$ (400 MHz)

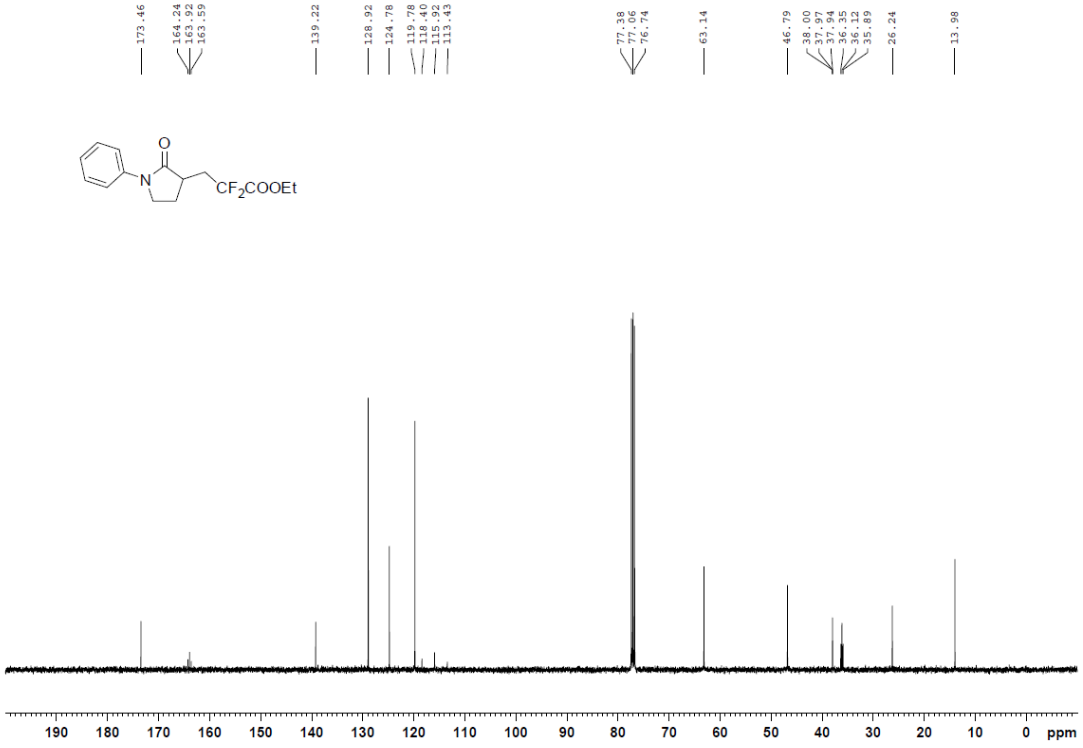

Supplementary Figure 262. ^{13}C NMR spectrum of **14** in $CDCl_3$ (101 MHz)

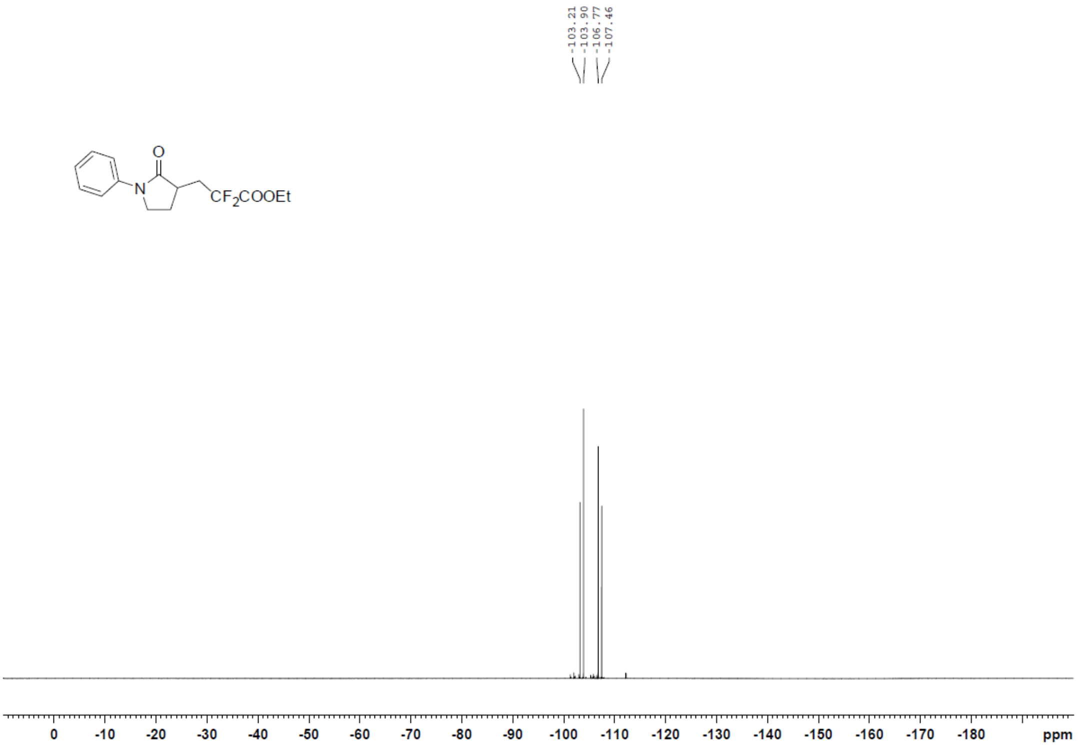

Supplementary Figure 263. ^{19}F NMR spectrum of **14** in $CDCl_3$ (376 MHz)

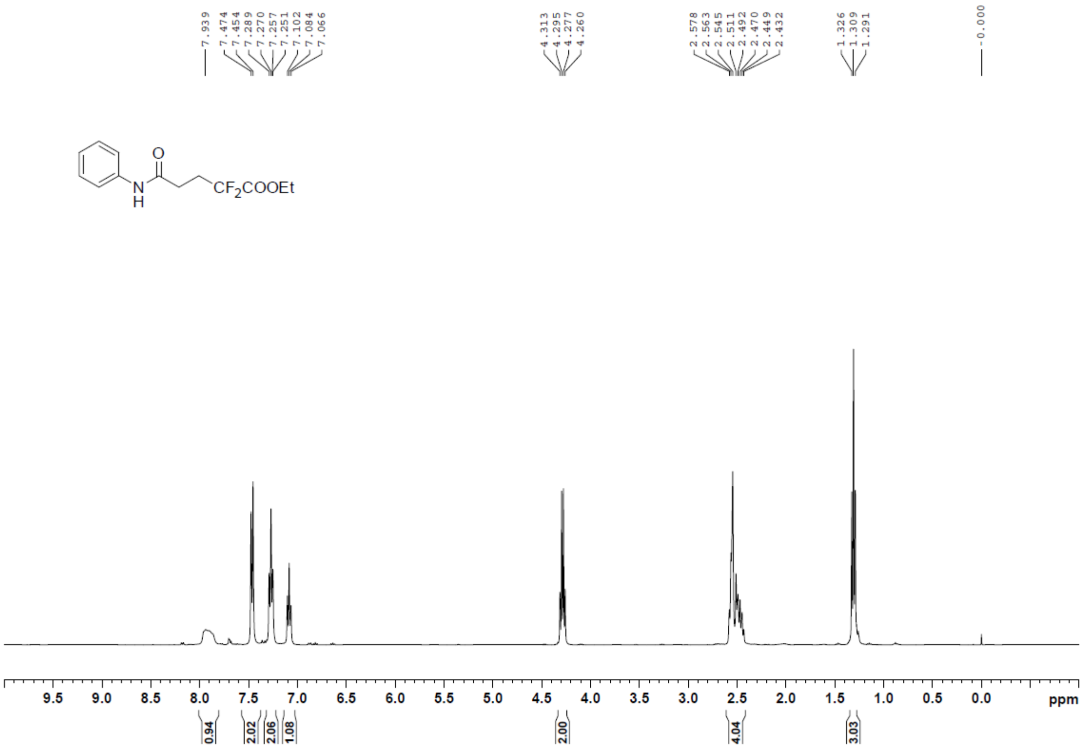

Supplementary Figure 264. ^{1}H NMR spectrum of **16** in $CDCl_3$ (400 MHz)

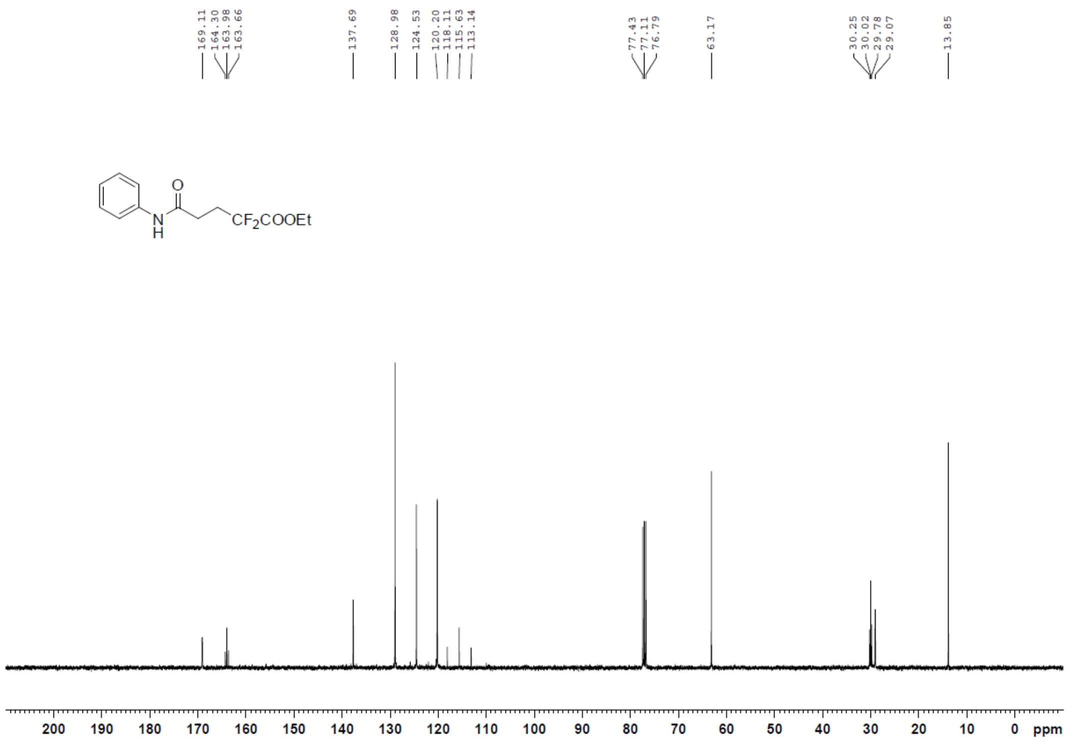

Supplementary Figure 265. ^{13}C NMR spectrum of **16** in $CDCl_3$ (101 MHz)

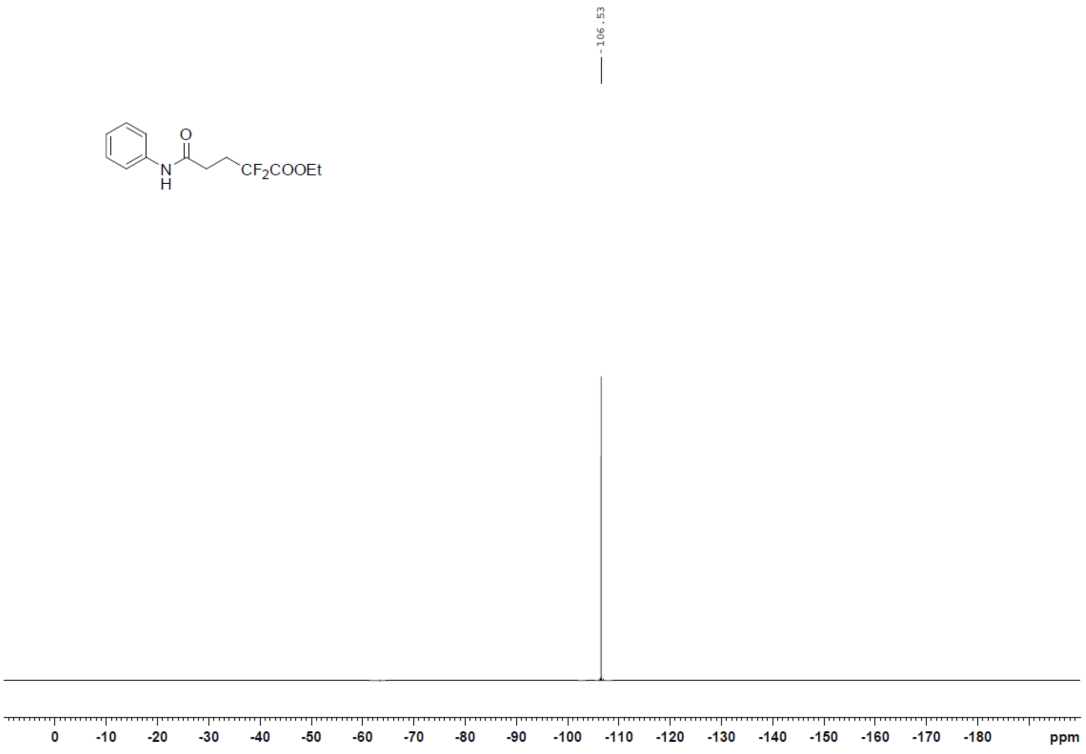

Supplementary Figure 266. ^{19}F NMR spectrum of **16** in $CDCl_3$ (376 MHz)

10. Supplementary References

1. Kawamata, Y., Yan, M., Liu, Z., Bao, D.-H., Chen, J., Starr, J. T. & Baran, P. S. Scalable, Electrochemical oxidation of unactivated C–H bonds. *J. Am. Chem. Soc*. **139**, 22, 7448-7451 (2017).
2. Li, J., Yang, Y., Liu, Y., Liu, Q., Zhang, L., Li, X., Dong, Y. & Liu, H. Palladium/norbornene catalyzed *ortho* amination/cyclization of aryl iodide: process to 3-Methyl-indole derivates and controllable reductive elimination against the second amination. *Org. Lett*. **23**, 2988-2993 (2021).
3. Wang, Y., Shen, S., He, C., Zhou, Y., Zhang, K., Rao, B., Han, T., Su, Y., Duan, X.-H. & Liu, L. Cu (II)-mediated direct intramolecular cyclopropanation of distal olefinic acetate: access to cyclopropane-fused γ-lactones. *Chem. Sci*. **14**, 6663-6668 (2023).
4. Rao, N., Li, Y.-Z., Luo, Y.-C., Zhang, Y. & Zhang. X. Nickel-catalyzed multicomponent carbodifluoroalkylation of electron-deficient alkenes. *ACS Catalysis*. **13**, 4111-4119 (2023).
5. Bao, Z.-P., Zhang, Y. & Wu, X.-F. Palladium-catalyzed four-component difluoroalkylative carbonylation of aryl olefins and ethylene. *J. Catal*. **413**, 163-167 (2022).
6. Zhang, J., Hu, W., Chen, Z., Wu, N., Li, C., Chen, T. & Han. L.-B. Water-promoted mild and general Michaelis–Arbuzov reaction of triaryl phosphites and aryl iodides by palladium catalysis. *Org. Lett*. **26**, 3386-3390 (2024).
